# A systematic review and network meta-analysis of psychological, psychosocial, pharmacological, physical and combined treatments for adults with a new episode of depression

**DOI:** 10.1016/j.eclinm.2024.102780

**Published:** 2024-08-16

**Authors:** Ifigeneia Mavranezouli, Odette Megnin-Viggars, Hugo Pedder, Nicky J. Welton, Sofia Dias, Edward Watkins, Neil Nixon, Caitlin H. Daly, Edna Keeney, Hilary Eadon, Deborah M. Caldwell, Katriona J.M. O'Donoghue, Sarah Stockton, Stephanie Arnold, James Thomas, Navneet Kapur, Stephen Pilling

**Affiliations:** a Centre for Outcomes Research and Effectiveness, Research Department of Clinical, Educational & Health Psychology, University College London, London, UK; b Population Health Sciences, Bristol Medical School, University of Bristol, Bristol, UK; c Centre for Reviews and Dissemination, University of York, York, UK; d Faculty of Health and Life Sciences, University of Exeter, Exeter, UK; e School of Medicine, University of Nottingham, Nottingham, UK; f National Institute for Health and Care Excellence, Manchester, UK; g Gateshead Health NHS Foundation Trust Library, Gateshead, UK; h EPPI-Centre, Social Research Institute, University College London, London, UK; i University of Manchester, Manchester, UK; j Mersey Care NHS Foundation Trust, Liverpool, UK

**Keywords:** Depression, Psychological interventions, Antidepressants, Physical interventions, Network meta-analysis, Clinical guidelines

## Abstract

**Background:**

Various effective treatments for depression exist. We aimed to identify the most effective first-line treatments for new episodes of less and more severe depression (defined by depression scale cut-off scores), to update NICE guidance on the management of Depression in Adults in England.

**Methods:**

Systematic review and network meta-analysis of randomised controlled trials (RCTs) published up to June 2020 (PROSPERO registration number CRD42019151328). We analysed interventions by class and individually. The primary efficacy outcome was depressive symptom change (expressed as standardised mean difference [SMD]). The review for this outcome was updated in November 2023.

**Findings:**

We included 676 RCTs, 105,477 participants and 63 treatment classes. For less severe depression, group cognitive/cognitive behavioural therapy (CT/CBT) class was efficacious versus treatment as usual [TAU], the reference treatment for this population [SMD −1.01 (95% Credible Interval [CrI] −1.76; −0.06)]. For more severe depression, efficacious classes versus pill placebo (reference treatment for this population) included combined individual CT/CBT with antidepressants [−1.18 (−2.07; −0.44)], individual behavioural therapies [−0.86 (−1.65; −0.16)], combined light therapy with antidepressants [−0.86 (−1.59; −0.12)], combined acupuncture with antidepressants [−0.78 (−1.12; −0.44)], individual CT/CBT [−0.78 (−1.42; −0.33)], mirtazapine [−0.35 (−0.48; −0.22)], serotonin and norepinephrine reuptake inhibitors [−0.32 (−0.43; −0.22)], tricyclic antidepressants [−0.29 (−0.50; −0.05)], and selective serotonin reuptake inhibitors [−0.24 (−0.32; −0.16)]. Additional treatments showed evidence of efficacy at the intervention level. Evidence for less and more severe depression was of low and low-to-moderate quality, respectively. In the 2023 update, group yoga and self-help without support emerged as efficacious for less severe depression. For more severe depression, combined group exercise with antidepressants emerged as efficacious, whereas combined light therapy with antidepressants failed to remain efficacious.

**Interpretation:**

Group CT/CBT (and possibly group yoga and self-help) appears efficacious in less severe depression, whereas antidepressants do not show evidence of effect. Combined antidepressants with individual CT/CBT, acupuncture and, possibly, group exercise, individual psychological therapies (behavioural therapies, CT/CBT) alone, and antidepressants alone appear efficacious in more severe depression. Quality of evidence, cost-effectiveness, applicability and implementation issues also need to be considered when formulating clinical practice recommendations.

**Funding:**

10.13039/100010377National Institute for Health and Care Excellence.

**Research in context**

**Evidence before this study**

Several published network meta-analyses (NMAs) have concluded that psychological, pharmacological and combined therapies are effective in the treatment of adults with depression, however none have made comparisons between specific psychological and pharmacological interventions and very few stratified populations by depressive symptom severity. We searched Embase, Emcare, MEDLINE, PsycINFO, CENTRAL and Cochrane Database of Systematic Reviews from inception to June 2020 to identify RCTs of psychological, psychosocial, pharmacological, physical and combined treatments used as first-line therapy for adults with a new episode of unipolar depression (not currently receiving treatment) and synthesised data using NMA techniques to address these gaps in evidence and update national guidance on the treatment and management of depression in adults in England, published by the National Institute for Health and Care Excellence (NICE). We undertook separate analyses for new episodes of less severe (subthreshold and mild) and more severe (moderate and severe) depression, as defined by clinical cut-off depression scale scores. The review was updated in 2023.

**Added value of this study**

Our NMA included 676 eligible RCTs (142 on less and 534 on more severe depression) assessing 63 treatment classes on 105,477 participants. Results differ from previous NMA findings in suggesting that (a) antidepressants do not show evidence of an effect against pill placebo in less severe depression; (b) group CBT may be the most effective treatment for less severe depression; (c) the mode of delivery (individual versus group) appears to impact on CBT effectiveness; and (d) some psychological interventions (such as individual CBT, individual behavioural activation, non-directive counselling, computerised CBT with or without support) may be more effective than antidepressants in more severe depression. Results remained largely unchanged following a review update in 2023.

**Implications of all the available evidence**

The findings of this NMA should be considered alongside other contextual factors when making practice recommendations on the treatment of adults with depression. Our results (including the size, uncertainty and limitations of the evidence) informed the updated NICE national guideline on the treatment and management of depression in adults. Other factors that were considered when formulating recommendations included cost-effectiveness evidence, results of pairwise meta-analysis of outcomes at follow-up, quality of life and functioning data, qualitative evidence on the facilitators and barriers to treatment choice, side effects and withdrawal symptoms associated with antidepressants, applicability of the evidence, implementation issues with regard to current structure of psychological treatment services in England, and the need to provide a wide range of interventions to meet individual needs and support patient choice.

## Introduction

Worldwide, major depression has a prevalence of 4.33% and is a leading cause of disability.^1^ Depression is associated with substantial costs to the health service and the wider society, mainly due to productivity losses.^2^^,^^3^ Providing evidence-based, effective interventions targeting depression will increase service costs but is expected to lead to overall cost-savings via increased productivity.^2^ Systematic reviews and pairwise meta-analyses have indicated that antidepressant medication and psychological interventions (e.g., cognitive behavioural therapy) are effective interventions for depression singly and in combination.^4^, ^5^, ^6^ However, there is limited evidence on the direct comparison of psychological and pharmacological interventions with evidence assessments tending to be siloed, with separate large network meta-analyses (NMAs) of antidepressant medication^7^^,^^8^ or psychotherapy^9^, ^10^, ^11^, ^12^, ^13^, ^14^ that do not fully address clinical choice. Of the many NMAs now available on the treatment for adults with depression,^7^, ^8^, ^9^, ^10^, ^11^, ^12^, ^13^, ^14^, ^15^, ^16^, ^17^, ^18^, ^19^ none has made comparisons between specific psychological and pharmacological interventions and few^17^, ^18^, ^19^ have assessed physical treatments. Furthermore, very few of the previous NMAs stratified populations by depressive symptom severity,^13^^,^^15^ a key factor in informing treatment choice.^20^

We employed NMA techniques to examine the relative effectiveness, acceptability and tolerability of psychological, psychosocial, pharmacological, physical and combined treatments, used as first-line therapy for adults with a new episode of unipolar depression (not currently receiving treatment), stratified by symptom severity level, to address these gaps and update national guidance on the management of depression in adults in England, published by the National Institute for Health and Care Excellence (NICE).^21^

## Methods

A systematic review of randomised controlled trials (RCTs) of treatments for adults with a new episode of unipolar depression was undertaken according to Preferred Reporting Items for Systematic reviews and Meta-Analyses (PRISMA) guidelines for NMAs.^22^ The study protocol (Appendix 1) was registered on PROSPERO (CRD42019151328). The review focused on first-line treatments for a new depressive episode, as separate reviews were conducted to identify suitable treatments for people who did not respond to previous treatment and those with chronic depression.

### Search strategy

Searches for RCTs (and systematic reviews of RCTs) of treatments for adults with depression were conducted in Embase, Emcare, MEDLINE, PsycINFO, CENTRAL and Cochrane Database of Systematic Reviews from inception, using relevant medical subject headings, free-text terms, and study type filters where appropriate (Appendix 1). The search was undertaken in May 2019 and updated in June 2020, with this date limit set to meet NICE guidance publication timelines (see also 2023 review update section below, specific to this publication). Additional search methods included checking reference lists of systematic reviews (identified through the electronic database search), and citation searches using Web-of-Science for included studies.

### Selection criteria

RCTs were eligible for inclusion if ≥80% of participants were adults not currently receiving treatment, initiated on first-line treatment for a new episode of unipolar depression (or subthreshold depressive symptoms) according to Diagnostic and Statistical Manual of Mental Disorders (DSM), International Classification of Diseases (ICD) or similar criteria, or by scoring above threshold on a validated depression scale. We excluded populations with perinatal depression, seasonal affective disorder, bipolar disorder, learning disabilities or in contact with the criminal justice system. Trials of further-line treatment, those specifically recruiting participants with depression and a physical health condition, or where ≥20% of participants had psychotic symptoms or a co-existing personality disorder or chronic depression were also excluded. We included only studies reporting data that could inform one or more outcomes of interest (described under ‘Analysis plan and data extraction’).

The review considered pharmacological, psychological, psychosocial, physical and combined treatments. Pharmacological treatments included selective serotonin reuptake inhibitors (SSRIs), serotonin–norepinephrine reuptake inhibitors (SNRIs), tricyclic antidepressants (TCAs), mirtazapine and trazodone. They were eligible if licensed in the UK and in routine clinical use as first-line treatments of depression. Psychological/psychosocial treatments included behavioural therapies, cognitive and cognitive behavioural therapies (CT/CBT), counselling, interpersonal psychotherapy (IPT), psychodynamic psychotherapies, psychoeducation, self-help with or without support, mindfulness, meditation or relaxation, and peer support. Physical treatments included acupuncture, exercise, yoga and light therapy. Combined treatments were also included (e.g., individual CT/CBT + antidepressants). Non-eligible treatments (such as drugs of no interest for decision-making, for example imipramine and bupropion, or broader classes and types of treatment rather than specific drug interventions, such as ‘SSRIs’, ‘TCAs’, and ‘antidepressants’), were included in the analysis if they had formed a combined treatment with an eligible psychological or physical intervention or if they had been compared against an eligible psychological or physical intervention, in order to enhance connectivity of eligible treatments in the networks. However, non-eligible treatments were not considered as part of the decision problem (that is, they were not considered when formulating recommendations). Controls included treatment as usual (TAU), waitlist, no treatment, attention placebo, sham treatments, and pill placebo. Appendix 1 provides a comprehensive description of eligible treatments, and details on the approach of considering ineligible pharmacological treatments in the analysis so as to enhance network connectivity.

Titles and abstracts of identified studies were screened by two reviewers for inclusion against pre-defined protocol criteria, until inter-rater reliability of ≥90% agreement was observed. Initially 10% of references were double-screened; as inter-rater agreement was >90%, the remaining references were screened by one reviewer. For studies considered relevant after title/abstract screening, the full text was acquired and checked for eligibility by both reviewers. Disagreements in study inclusion were resolved via discussion between the two reviewers, and consultation with a senior reviewer if necessary.

### Analysis plan and data extraction

Separate analyses were conducted for new episodes of less and more severe depression. Baseline mean scores on validated depression scales were used to classify the study population into one of these two levels of depressive symptom severity, using, as a starting point, default cut-off points for different depressive symptom levels, crosswalk tables of standardized depression measurements,^23^, ^24^, ^25^, ^26^ and clinical judgement. Emphasis was given to PHQ-9 as this is the most widely used screening tool for depression in primary care in the UK, although there were concerns that default thresholds may overstate depressive symptom severity compared with other validated scales.^27^, ^28^, ^29^ Provisional cut-off points were further calibrated to allow a clinically meaningful distinction between the two severity levels, aligned with treatment decisions in clinical practice. Full details are provided in Appendix 1. The final depression scale cut-off points used to classify populations into groups of adults with a new episode of less and more severe depression are shown in Table 1. Where baseline scores were unavailable, severity was determined according to the study inclusion criteria or description (if unambiguous, e.g. ‘severe’ or ‘subthreshold’).

**Table 1:** Cut-off points for depression scales, estimated using crosswalk tables of standardized depression measurements^23^, ^24^, ^25^, ^26^ (with a score at the threshold point and above indicating more severe depression, that is, of at least moderate severity).

Scale	Threshold
HAMD (17-item, 21-item and 24-item)	16
MADRS (10-item)	22
PHQ-9	16
BDI-I (21-item)	22
BDI-II (21-item)	30
CES-D (20-item)	36
QIDS (16-item)	12
HADS-D (7-item)	12

BDI: Beck's Depression Inventory; CES-D: Center for Epidemiologic Studies Depression Scale; HADS-D: Hospital Anxiety and Depression Scale—Depression sub-scale; HAMD: Hamilton Depression Rating Scale; QIDS: Quick Inventory of Depressive Symptomatology; MADRS: Montgomery–Åsberg Depression Rating Scale; PHQ-9: Patient Health Questionnaire.

Due to the high number of included interventions, these were grouped into treatment classes, according to common theoretical structure or hypothesized mechanism of action (see Appendix 1 for details).

Seven outcomes at treatment endpoint were analysed using NMA techniques:

• Efficacy:
  o Standardised mean difference (SMD) of depressive symptom change from baseline (primary efficacy measure)
  o Response (typically defined as ≥50% improvement on a depression scale score) in those randomised
  o Response in treatment completers (that is, in those who did not discontinue treatment early)
  o Remission (typically defined as a depression scale score below a threshold) in those randomised
  o Remission in treatment completers
• Acceptability: treatment discontinuation for any reason
• Tolerability: treatment discontinuation due to side effects from medication, in those who discontinued treatment.

Four of these outcomes (response and remission in treatment completers; discontinuation due to any reason and due to side effects) informed the model-based economic evaluation of first-line treatments for a new episode of depression undertaken to support the NICE guideline.

From each included study, we extracted: country, population, intervention details, outcome data, and potential risk of bias assessed using the Cochrane Risk-of-Bias tool.^30^ Data extraction was double-checked by a second reviewer. Disagreements were resolved via consultation with a senior reviewer. A large NMA of antidepressants for the acute treatment of adults with depression^7^ was used as a source of data from non-English language and unpublished studies.

### Statistical analysis

NMAs were conducted within a Bayesian framework using Markov Chain Monte Carlo simulation techniques implemented in OpenBUGS 3.1.2, which is a variant of WinBUGS.^31^, ^32^, ^33^ For the depressive symptom change, we pooled the SMD between treatments using a NMA model with normal likelihood and identity link function, accounting for different reporting formats between studies.^34^ For discontinuation, response and remission we pooled log-odds ratios (LORs) between pairs of treatments using a NMA model with binomial likelihood and logit link function^34^ (Appendix 1 provides method details). We used random study effects models to capture the expected between-study variability in intervention effects. Intervention effects were modelled using random class models, assuming that intervention effects within each class are distributed around a common class mean effect with a within-class variance.

For each analysis we estimated mean relative effects (SMD or LOR, as relevant) between classes and between interventions, with 95% credible intervals (CrI). We also estimated mean ranks with 95%CrI for every class and intervention, where a rank of 1 indicates the best outcome. Although all connected eligible classes and interventions were included in the NMAs, we only considered results for classes and interventions tested on ≥50 participants (or ≥50 completers, for the completer outcomes) across RCTs in each analysis, as the minimum adequate evidence base to support recommendations. We determined ‘evidence of effect’ based on whether the 95%CrI crossed the no effect line. We reviewed relative effects against a reference treatment, which was treatment as usual (TAU) and pill placebo for less and more severe depression, respectively, selected following consideration of their clinical significance, size of the evidence and connectivity in each population. TAU was relatively ill-defined in the included studies, with often little more detail than ‘usual care’. However, where a study did report the proportion of participants who received different treatments within the TAU arm, and if at least 80% of participants in that arm received a consistent treatment class (for example SSRIs), then the arm was coded as such rather than as TAU. As outlined in the review protocol, where a study compared ‘intervention + TAU versus TAU alone’ it was recoded as ‘intervention versus no treatment’. Pill placebo was originally preferred for use as the reference treatment for both less and more severe depression, because it is well-defined across RCTs. However, there was either very limited connectivity or lack of evidence for pill placebo in the less severe depression networks, so that it wasn't possible to use as the reference treatment for this population. On the other hand, TAU may be a heterogeneous condition but has been widely used as a control condition across RCTs in populations with a new episode of less severe depression; for this reason, TAU was selected as the reference treatment for less severe depression. For discontinuation due to side effects from medication, the reference treatment was pill placebo for both severity levels. We also made comparisons between active treatments at class and intervention level.

### Transitivity and inconsistency checks

NMA assumes that the distribution of effect modifiers is the same across treatment comparisons (‘transitivity’ assumption). To control for potential effect modifiers, we aimed to reduce heterogeneity by stratifying analyses by depressive symptom severity and assigning interventions to classes using detailed definitions and considering their mode of delivery. Violations of the transitivity assumption may lead to inconsistency between direct and indirect evidence estimates.^35^ This was assessed statistically by comparing the fit of a model assuming consistency with a model allowing for inconsistency at the intervention level, whilst still modelling class effects^36^ (see Appendix 1). Furthermore, we compared NMA and inconsistency model effects for all comparisons and estimated differences between direct and indirect effects to identify treatment comparisons where this difference was statistically meaningful and higher than the minimal clinically important difference, determined as ±0.5 for the SMD and ±0.25 for log-ORs.

We further explored the transitivity between pharmacological and non-pharmacological trial participants by conducting a sensitivity analysis on the depressive symptom change outcome (SMD) that excluded pharmacological trials, and comparing the effect size and ranking of non-pharmacological treatments between this analysis and the full analysis (which included pharmacological trials).

### Quality assessment of the evidence

We evaluated the quality of the evidence with a narrative summary using the standard GRADE approach domains (risk of bias, inconsistency, publication bias, indirectness and imprecision).^37^

### Bias adjustment models

Bias adjustment models^38^ were fitted for the outcomes of depressive symptom change (SMD), treatment discontinuation for any reason, and response in completers, testing for bias associated with small sample size^39^ (Appendix 1). Analyses assumed possible bias in comparisons of active interventions versus inactive controls and no bias in comparisons between inactive controls or between active interventions. As an exception we assumed bias against counselling in comparisons where counselling was the control intervention, as there were concerns that non-directive counselling, when used as control, is less likely to be manual-based and delivered in a comparable number of sessions by an equivalent healthcare professional as when it is assessed as the active intervention. Magnitude of bias was assumed to be the same across all potentially biased comparisons. Where bias was indicated, results from bias-adjusted models were considered.

### 2023 review update

We reran the full search on 28 November 2023 and updated the NMA of the primary outcome (SMD of depressive symptom change) for less and more severe depression to explore whether there were substantial changes in conclusions between our original analysis (2020) and the updated analysis (2023). Due to the high number of records captured in the search, we first used machine learning methods to eliminate records with high likelihood of being irrelevant, before proceeding to manual selection of the remaining, potentially eligible, studies. Appendix 2 provides the updated search strategy and a description of the machine learning methods employed for the review update.

### Role of the funding source

This work was funded by NICE, who had no active role in study design, data collection, data analysis, data interpretation, or writing of the report. All authors had full access to all the data in the study and had final responsibility for the decision to submit for publication.

## Results

### Studies and treatments

**Fig. 1:**
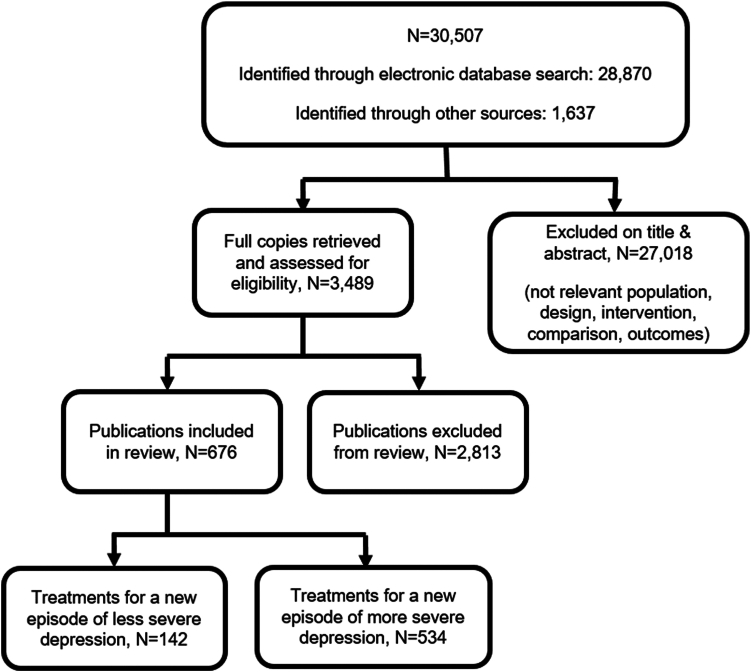
Flow diagram of study selection for the systematic review and network meta-analysis.

The systematic search identified 3451 potentially eligible publications, of which 676 RCTs (142 on less and 534 on more severe depression) that assessed 63 treatment classes on 105,477 participants met eligibility criteria for the NMA (Fig. 1). Participants included adults with a new episode of unipolar depression as defined according to DSM, ICD or similar criteria in 503 RCTs, or depressive symptoms as indicated by baseline depression scores on validated scales (including those with subthreshold depressive symptoms) in the remaining 173 RCTs. The median treatment duration was 6 weeks (interquartile range -IR- 6 to 8 weeks) for pharmacological interventions, 9 weeks (IR 6–12 weeks) for psychological interventions, 8 weeks (IR 6–12 weeks) for physical interventions and 12 weeks (IR 6–14 weeks) for combined interventions. Appendix 3 contains lists of included and excluded studies (with study characteristics for included studies).

**Fig. 2:**
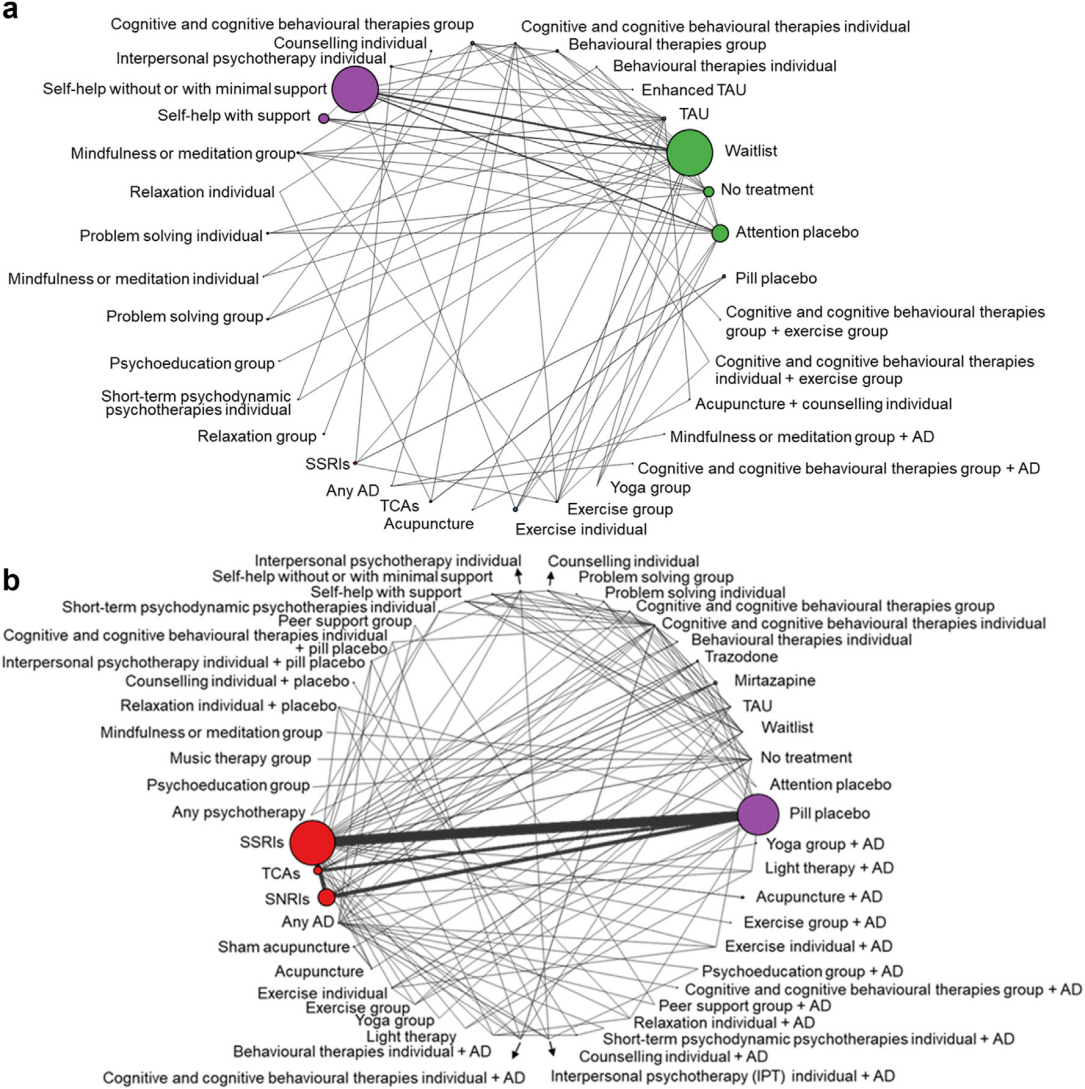
Networks of the NMA of standardised mean difference (SMD) of depressive symptom change in adults with a new episode of (a) less severe depression and (b) more severe depression—treatment class level. The width of lines is proportional to the number of trials in which each direct comparison is made. The size of each circle (treatment node) is proportional to the number of participants on each treatment class across RCTs. AD: antidepressant; SNRIs: serotonin and norepinephrine reuptake inhibitors; SSRIs: selective serotonin uptake inhibitors; TAU: treatment as usual; TCAs: tricyclic antidepressants.

The networks of depressive symptom change (SMD) included 127 RCTs, 16,829 participants, 34 classes and 76 interventions for less severe depression, and 352 RCTs, 59,350 participants, 50 classes and 99 interventions for more severe depression. See Fig. 2 for class level SMD network plots, Appendix 4 for all other network plots (class and intervention level) and information on classes/interventions, numbers of RCTs and participants on each outcome, and Appendix 5 for full data utilised and the number of RCTs per comparison in each NMA.

### Assessment of heterogeneity, inconsistency and bias

In less severe depression, heterogeneity (measured by the posterior median between-study standard deviation) was high for response outcomes, and moderate for depressive symptom change (SMD), discontinuation for any reason, and remission outcomes. In more severe depression, heterogeneity was low for remission in treatment completers and moderate for all other outcomes. In less severe depression, evidence of inconsistency was identified only for response in completers. In more severe depression, there was evidence of inconsistency in SMD, response in those randomised, and discontinuation for any reason (Appendix 6). Comparison of NMA and inconsistency model estimates identified only a small number of treatment comparisons with clinically important differences between direct and indirect effects (Appendix 7).

Evidence of small-study bias was identified for depressive symptom change (less and more severe depression), response in treatment completers (more severe depression) and treatment discontinuation for any reason (more severe depression), so results from bias-adjusted models were considered (Appendix 6).

For less severe depression, most risk-of-bias domains were rated as low or unclear except for blinding of participants and personnel due to lack of blinding in trials assessing psychological interventions alone or in combination. For more severe depression, the majority of risk-of-bias domains were rated as low or unclear, with the exception of selective reporting bias and other bias (which included potential conflict of interest based on the funding source). Differences in types of bias and quality ratings between less and more severe depression are attributed to the considerably larger proportion of pharmacological trials for more severe depression compared with less severe depression.

Following quality assessment using the narrative summary of the standard GRADE approach domains, evidence was judged to be of low and low-to-moderate quality for less and more severe depression, respectively (Appendix 6).

### Treatment outcomes

**Fig. 3:**
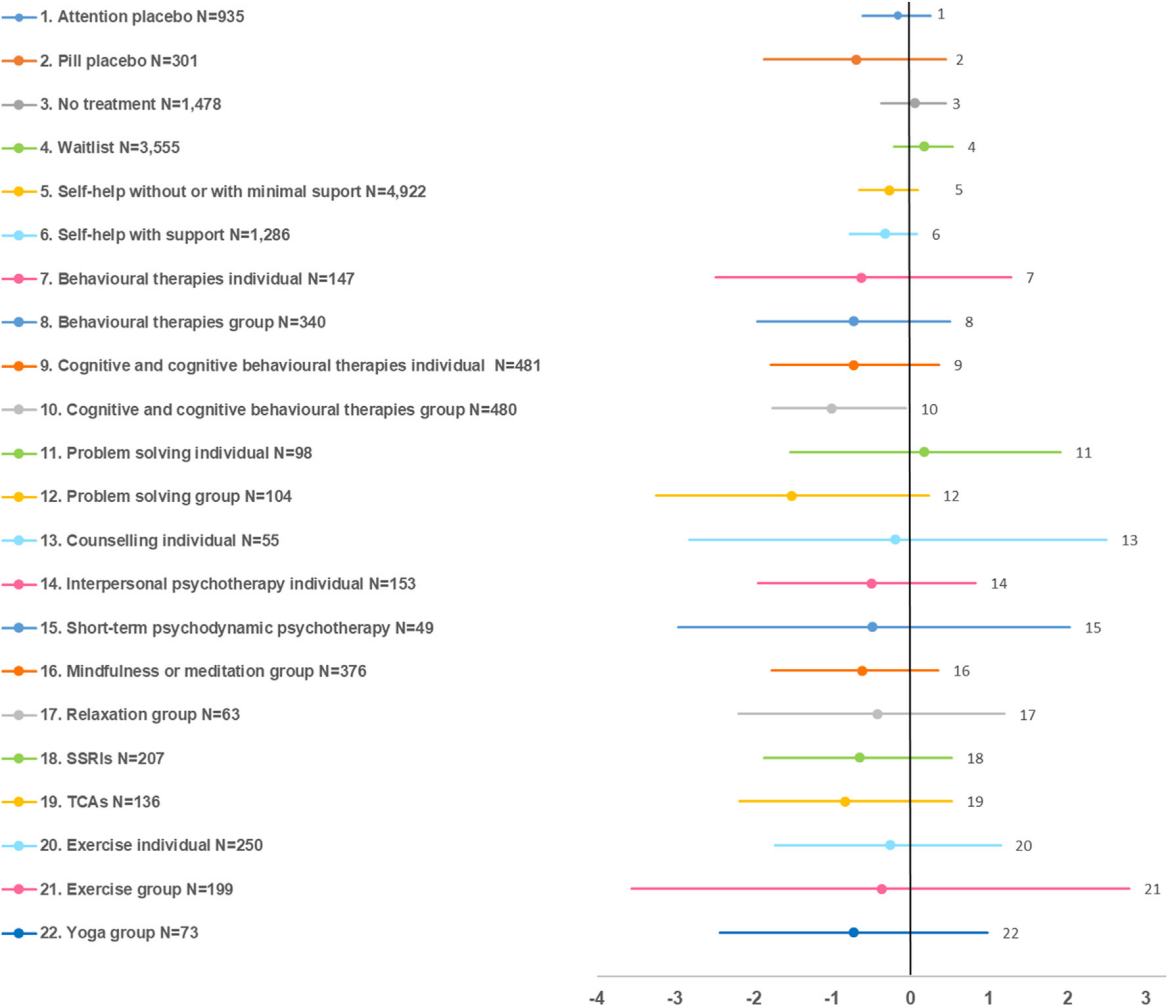
Bias-adjusted forest plots of standardised mean difference (SMD) of depressive symptom change in adults with a new episode of less severe depression: effects of treatment classes versus treatment as usual (TAU, N = 815). Values on the left side of the vertical axis indicate better effect compared with TAU. Effects are shown only for treatment classes with N ≥ 50, plus short-term psychodynamic psychotherapy (N = 49). SSRIs: selective serotonin uptake inhibitors; TCAs: tricyclic antidepressants.

**Fig. 4:**
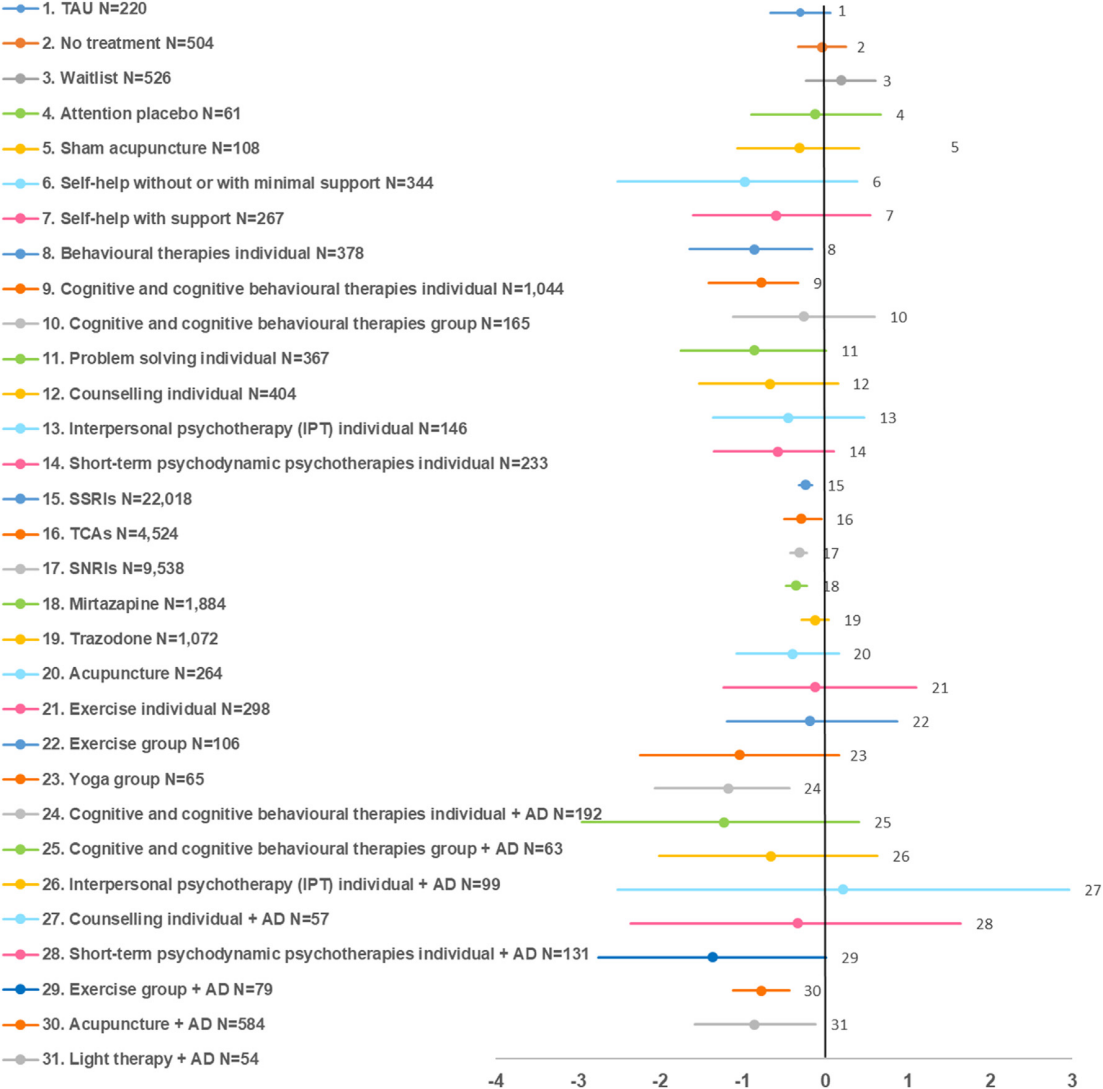
Bias-adjusted forest plots of standardised mean difference (SMD) of depressive symptom change in adults with a new episode of more severe depression: effects of treatment classes versus pill placebo (N = 12,554). Values on the left side of the vertical axis indicate better effect compared with pill placebo. Effects are shown only for treatment classes with N ≥ 50. AD: antidepressant; SNRIs: serotonin and norepinephrine reuptake inhibitors; SSRIs: selective serotonin uptake inhibitors; TAU: treatment as usual; TCAs: tricyclic antidepressants.

Bias-adjusted results on the SMD of depressive symptom change of each class versus the reference treatment are shown as forest-like plots in Fig. 3, Fig. 4, for less and more severe depression, respectively. Full results of treatment effects versus the reference treatment for all outcomes are shown in Appendix 6 (classes) and Appendix 8 (interventions). Relative effects between active treatments (classes and interventions) for all outcomes, including inconsistency model comparisons, are reported in Appendix 7.

For less severe depression, group CT/CBT was the only class with clear evidence of efficacy versus TAU on depressive symptom change (SMD -1.01, 95%CrI −1.76; −0.06). At the intervention level, group interventions (behavioural activation, problem solving, MBCT, mindfulness meditation), individual third-wave cognitive therapy, and, marginally (with upper 95%CrI just crossing the no effect line), individual CBT (≥15 sessions), cognitive bibliotherapy with or without support, and a number of computerised interventions with or without support, were effective versus TAU. Most other classes and interventions showed improvements in outcomes, but 95%CrI were wide and crossed the no effect line. Group treatments showed higher effects compared with TAU regarding response and remission. No differential effects between classes were found. SSRIs and TCAs showed very small effects with high uncertainty compared with pill placebo. At the intervention level, group interventions (behavioural activation, CBT and problem solving) showed higher effect than self-help interventions with or without support.

For more severe depression, classes with evidence of efficacy on depressive symptom change versus pill placebo included combined individual CT/CBT with antidepressants (SMD −1.18, 95%CrI −2.07; −0.44), individual behavioural therapies (−0.86, 95%CrI −1.65; −0.16), combined light therapy with antidepressants (−0.86, 95%CrI −1.59; −0.12), combined acupuncture with antidepressants (−0.78, 95%CrI −1.12; −0.44), individual CT/CBT (−0.78, 95%CrI −1.42; −0.33), mirtazapine (−0.35, 95%CrI −0.48; −0.22), SNRIs (−0.32, 95%CrI −0.43; −0.22), TCAs (−0.29, 95%CrI −0.50; −0.05) and SSRIs (−0.24, 95%CrI −0.32; −0.16). Effective interventions belonging to other classes included cognitive bibliotherapy, computerised CBT with or without support, various individual psychotherapies (problem solving, non-directive counselling, short-term psychodynamic psychotherapy [PDPT]), various combinations of psychological or physical interventions (group CBT, IPT, group exercise) with antidepressants, and group yoga. The majority of other classes and interventions showed improvements in outcomes, but with wide 95%CrI that crossed the no effect line. Similar results were found for response and remission. Long term PDPT alone or combined with antidepressants showed higher remission than pill placebo. Combined treatment classes (individual CT/CBT or acupuncture) with antidepressants showed higher effects than antidepressant classes alone. At the intervention level, self-help (cognitive bibliotherapy, computerised CBT with or without support) and individual psychological interventions (problem solving, behavioural activation, CBT, non-directive counselling) were overall more effective than antidepressants. Combined traditional or electro-acupuncture with SSRIs was more effective than traditional acupuncture or antidepressants alone.

No class showed higher risk of discontinuation versus reference in either depressive symptom level. Other than SSRIs in less severe depression, all assessed antidepressant classes showed higher risk of discontinuation due to side effects compared with pill placebo.

The sensitivity analysis conducted to explore the transitivity assumption between pharmacological and non-pharmacological trial participants suggested only small changes in non-pharmacological treatment effects on the SMD after exclusion of pharmacological RCTs for less severe depression, probably because there were few pharmacological trials in this population. For more severe depression, exclusion of pharmacological trials resulted in some changes and higher uncertainty in effects and rankings of non-pharmacological treatments, apparently because most evidence in this population came from pharmacological trials (Appendix 6). Overall, non-pharmacological treatment effects were not substantially affected after exclusion of pharmacological trials.

### Results from the 2023 review update

The search update identified 26,024 records. Following machine learning methods that eliminated records with high likelihood of being irrelevant, 5656 publications remained as potentially eligible, of which 165 were assessed at full text for eligibility. The selection process resulted in 16 and 10 new RCTs for less and more severe depression, respectively (see flow diagram in Appendix 9). Conclusions on the SMD (primary outcome) remained largely unchanged. However, in contrast to the original analysis for less severe depression, group yoga and self-help without support showed evidence of effect versus TAU. Group yoga also showed evidence of effect versus self-help with support in this population. At the intervention level, in addition to group interventions, individual behavioural activation, individual CBT interventions and a wider range of self-help interventions without support showed evidence of effect versus TAU. The updated analysis for more severe depression differed from the original results in that combined group exercise with antidepressants showed evidence of efficacy versus pill placebo, whereas combined light therapy with antidepressants failed to show evidence of efficacy versus pill placebo (however, interventions within this class retained evidence of efficacy versus pill placebo). At the intervention level, combined group CBT with antidepressants appeared to be more efficacious than group CBT alone, antidepressants alone, individual exercise interventions and acupuncture. Appendix 9 and Appendix 10 show full data and results on the depressive symptom change (SMD) outcome following the review update.

## Discussion

This large NMA, which was undertaken specifically to inform recommendations on first-line treatments of a new depressive episode as part of national guidance on the treatment and management of depression on adults,^21^ compared psychological, psychosocial, pharmacological, physical and combined first-line treatments for a new depressive episode stratified by symptom severity level and synthesised evidence from 676 RCTs, 105,477 participants and 63 classes in 5 efficacy and two discontinuation outcomes.

For less severe depression, predominantly the group CT/CBT class, other group interventions (e.g. behavioural activation, MBCT), individual third-wave cognitive therapy, and, marginally, individual CBT (≥15 sessions), cognitive bibliotherapy with or without support, and a number of computerised interventions with or without support, appeared to be effective versus TAU. Most other classes and interventions showed improvements in outcomes compared with TAU, but results were uncertain. Antidepressants did not show evidence of effect compared with either TAU or pill placebo. Results may be explained by benefits associated with group cohesion and normalization effects, learning and support from others and functioning as co-therapists^40^^,^^41^ The lack of a clear, distinguishable effect for most other treatments versus TAU may be attributable to the increased likelihood for non-specific effects, such as natural recovery, in less severe depression. Group CT/CBT, the only class with clear evidence of effect versus TAU, was tested on N = 480 on the SMD.

For more severe depression, combined treatments (antidepressants combined with individual CT/CBT, light therapy, or acupuncture) were more effective than antidepressants, psychological treatments, acupuncture, or exercise alone. Individual psychological treatment classes (behavioural therapies, CT/CBT), other psychological interventions (problem solving, non-directive counselling, short-term PDPT, cognitive bibliotherapy, computerised CBT with or without support) and antidepressants (mirtazapine, SNRIs, TCAs and SSRIs) were effective versus pill placebo. Some self-help and high-intensity psychological interventions showed higher effects than antidepressants. Most other classes and interventions showed improvements in outcomes but with considerable uncertainty. Antidepressants showed higher risk of discontinuation due to side effects relative to pill placebo. Antidepressants had by far the largest evidence base (ranging from N = 1884 for mirtazapine to N = 22,018 for SSRIs on the SMD outcome), followed by individual CT/CBT class (N = 1044), combined acupuncture with antidepressants (N = 584), and individual behavioural therapies class (N = 378).

Previous NMAs concluded that (a) antidepressants were more effective than pill placebo with little differences among them^7^^,^^8^; (b) guided and unguided self-help CBT^9^^,^^11^^,^^13^ and high-intensity psychological therapies^10^, ^11^, ^12^ were more effective than TAU, waitlist and/or pill placebo; (c) individual, group, telephone, and guided self-help CBT had similar effectiveness^9^ and (d) combined psychotherapy with pharmacotherapy was more effective than psychotherapy or pharmacotherapy alone, with no differences being found between psychotherapy and pharmacotherapy.^15^^,^^16^^,^^18^ However, no previous NMA made comparisons between specific psychological and pharmacological treatment classes or interventions. Our results differ from previous NMA findings in that, according to our analyses, (a) antidepressants did not show evidence of an effect against pill placebo in less severe depression; (b) group CBT appears to be the most effective treatment for less severe depression; (c) the mode of delivery appears to impact on CBT effectiveness; and (d) some psychological interventions may be more effective than antidepressants in more severe depression (see Appendix 7 for full results on comparisons between interventions). Differences between our NMA and previous NMA results may be attributable to different inclusion criteria (e.g., we included only RCTs of people treated for a new depressive episode not currently receiving treatment) and our stratification by depressive symptom severity. We also synthesised a much larger evidence base than previously published NMAs by including a wider range of treatment modalities. Compared with other published NMAs in the field, we considered a substantially larger number of distinct, clearly defined pharmacological and psychological interventions grouped into homogeneous treatment classes by taking also into account their mode of delivery (we included approximately 150 active interventions grouped into 51 active treatment classes), and, for the first time, we employed class models for analyses, which allowed us to capture subtle differences in efficacy between treatment classes and interventions within and across classes. Furthermore, we conducted analyses adjusted for bias due to small study size, which, to our knowledge, has not been done by any other NMA in the area so far.

Our conclusions on the antidepressant treatment effects in less severe depression differ from those of a recent large individual participant data (IPD) meta-analysis that assessed the effects of SSRIs for non-severe and severe depression.^42^ These differences may be further attributable to different cut-off points used to define less severe depression in our study (HAMD<16) compared with the cut-offs for the non-severe depression in the IPD meta-analysis (HAMD<19),^42^ and the use of IPD by the other study, which enabled assessment of antidepressant treatment effects on the core symptoms of depression (expressed in the 6-item Bech HAMD sub-scale).^42^ On the other hand, our findings on antidepressant effects versus pill placebo are in line with those of an older IPD meta-analysis, which concluded that the magnitude of antidepressant effects compared with pill placebo increases with depressive symptom severity and may be overall minimal or non-existent in people with mild or moderate symptoms (defined by a HAMD score of 8–13 and 14–18, respectively).^43^

We used strict criteria to identify a homogeneous population experiencing a new depressive episode and excluded studies where >20% of participants were already receiving treatment (most commonly antidepressants) at trial initiation or had chronic depression or depression not responding to previous treatment, and studies specifically recruiting participants with depression and physical health problems. To stratify analyses by symptom severity level we categorised studies using the study sample's mean baseline depressive symptom score. However, some study categorisations may have been incorrect if baseline score distributions were largely skewed. Moreover, the approach we used to categorise the study population into the levels of less and more severe depression is novel, using partly clinical judgement, and has not been validated, but recognises some of the inherent challenges in depression symptom scale measurements, such as (a) the default thresholds of depression symptom scales may not necessarily correspond to each other, as suggested by the use of cross-walk tables; (b) some scales may have more than one threshold sets reported in the published literature and used in routine practice; and (c) for some scales, default thresholds may overstate depressive symptom severity. The choice of threshold may have a crucial impact on the analysis results, as it affects the number of studies and trial participants as well as the availability of treatment classes and interventions in each network, and may therefore also have an impact on the estimation of bias in bias-adjusted models and the estimation of within-class variance in class models (which depends on the availability of treatment classes and interventions in the network). An alternative approach to explore the impact of depressive symptom severity on treatments effects would be by employing IPD NMA^44^; however, we did not have access to IPD data.

We employed class models^34^ to gain precision on treatment effects, connect networks disconnected at the intervention level, and aide interpretability. We used TAU (instead of the well-defined pill placebo) as the reference treatment for less severe depression, due to the very limited evidence and connectivity of pill placebo in the respective evidence networks. We reduced the heterogeneity of the TAU node by recoding comparisons of ‘intervention plus TAU versus TAU alone’ as ‘intervention versus no treatment’, and coding TAU arms where TAU was described as a well-defined intervention or control condition accordingly. Nevertheless, the remaining TAU arms forming the TAU node may still comprise a heterogeneous condition, which is acknowledged as a limitation of the analyses for less severe depression.

Analyses for more severe depression showed some inconsistency between direct and indirect evidence. Notably, across analyses, between-study heterogeneity was lower in more severe depression compared with less severe depression. This could be explained by the larger number of pharmacological trials included in more severe depression, which typically recruit more homogeneous populations. However, exclusion of pharmacological trials had only a small impact on the SMD results, suggesting that the transitivity assumption between non-pharmacological and pharmacological trial populations is acceptable. Pairwise sub-group analysis assessing age and setting as potential effect modifiers did not find evidence suggestive of a differential effect between older (aged ≥60 years) versus younger (aged <60 years) adults and between inpatient and outpatient care settings (although the latter was based on a very limited evidence base). Similar planned analyses to assess gender and ethnicity as potential effect modifiers were not possible to undertake due to lack of relevant data. In addition to those factors, it is possible that other, unknown effect modifiers (which were therefore not possible to control for) were not evenly spread across comparisons, thus increasing heterogeneity and compromising transitivity. Bias-adjusted analyses suggested the presence of small study effects favouring active versus control treatments in some outcomes, however it is unclear whether this is due to publication bias or study design aspects that may differ between smaller and larger studies. Small study effects were assumed to be common across comparisons of active interventions versus control (or non-directive counselling) in each analysis, though in reality there may be groups of active treatments (e.g. pharmacological, psychological) in which this bias is more pronounced.

In addition to the above analyses, we also conducted post-hoc pairwise meta-analyses of the available evidence, to compare their effect sizes to those obtained from the NMA. We considered the effects between the NMA and the pairwise meta-analysis to be ‘significantly different’ if the NMA effect estimate was not within the 95% confidence interval (CI) of the pairwise meta-analysis effect estimate. For less severe depression, out of 93 available comparisons between NMA and pairwise meta-analysis results, we found 11 comparisons (12%) where results were ‘significantly different’. For more severe depression, out of 160 available comparisons between NMA and pairwise meta-analysis results, we found 17 comparisons (11%) where effects were ‘significantly different’. For most differences identified, the difference was in the magnitude rather than the direction of effect and could probably be accounted for by the smaller evidence base contributing to the pairwise effect estimates.

The quality of the NMA evidence, narratively assessed using the GRADE approach domains,^37^ was judged to be low and low-to-moderate for less and more severe depression, respectively. This overall low quality of the evidence in this research area has been reported in other published NMAs.^7^^,^^10^^,^^18^ Originally, a threshold analysis was planned to test the robustness of the NMA-based treatment recommendations to potential biases or sampling variation in included evidence.^45^ However, this analysis was ultimately not considered feasible or helpful to conduct due to the lack of clear decision-making rules linking recommendations to the NMA results, as other pragmatic criteria were factored in (discussed below). CINeMA, a method used to evaluate the confidence in the NMA results^46^ was also not possible to undertake due to the implementation of class models.

Our systematic review included evidence published in English language unless data could be extracted from an existing review.^7^ Evidence suggests that use of language restrictions in systematic review-based meta-analyses in conventional medicine does not introduce systematic bias.^47^ Final NICE guideline evidence searches were conducted in June 2020, due to the complexity and time required to run the analyses prior to guideline publication. Given the possibility of new evidence becoming available since then, we updated our searches and analyses on the depressive symptom change (SMD) outcome in November 2023, using novel machine learning methods to speed up the study selection process. Results remained largely unchanged. In less severe depression, group yoga, self-help without support and some individual psychological interventions (behavioural activation, CBT) emerged as efficacious versus TAU. Results for group yoga were attributable to the inclusion of one small RCT (N = 62) with a substantial positive effect for laughter group yoga (Armat 2022). In more severe depression, combined group exercise with antidepressants emerged as efficacious versus pill placebo (albeit based on a more limited evidence base than other efficacious classes in this population), whereas light therapy combined with antidepressants failed to show efficacy as in the original analysis, which is likely attributable to its limited evidence base, which apparently could not ensure stability in the results. The similarity between the updated and original results on the SMD outcome is not surprising given the particularly large evidence base that was synthesised in our original analysis (so that a small body of newly identified evidence was unlikely to shift conclusions to a considerable extent) and increases our confidence in our original conclusions. A search update conducted in July 2024 identified 13 eligible studies for our review and NMA of the primary outcome of the SMD of depressive symptom change (Appendix 11). The newly identified evidence for less severe depression (9 RCTs) might potentially reduce the uncertainty in findings for group exercise, group mindfulness or meditation, self-help with support, and IPT, possibly weaken the findings for group CBT, and strengthen the updated findings for yoga and self-help, although the size of this new evidence is very small compared with the evidence base synthesised in our NMA and thus unlikely to be influential to our reported results. Some new evidence was also found for light therapy in less severe depression. For more severe depression, there was very limited evidence (4 RCTs) on self-help (with or without support) and SSRIs that is highly unlikely to alter our reported conclusions. However, it is likely that, as new evidence accumulates, especially for currently less well-researched treatments, some treatments with currently uncertain findings may show evidence of efficacy in the future.

In terms of developing clinical recommendations, the findings presented here were not the only data considered by the guideline committee. The full guideline process is described elsewhere^21^ but, in making recommendations, the guideline committee took into account the size, uncertainty and limitations of both the clinical and cost-effectiveness evidence, results of pairwise meta-analysis of outcomes at follow-up, quality of life and functioning data, qualitative evidence on the facilitators and barriers to treatment choice, side effects and withdrawal symptoms associated with antidepressants, the applicability of the evidence to the UK context (e.g. evidence on acupuncture came predominantly from Chinese studies and there may be differences between Chinese and Western-style acupuncture techniques), implementation issues with regard to current structure of psychological treatment services in England, and the need to provide a wide range of interventions to meet individual needs and support patient choice.

Based on the NMA findings and the above considerations, the NICE updated guideline on Depression in adults^21^ recommends a wide range of first-line treatments for adults with a new episode of depression, arranged in a suggested order in which they should be considered within a shared decision-making context (see NICE visual summaries for new episodes of less severe depression and more severe depression). Recommendations generally reflect current practice, but may reduce variation in practice across the National Health Service in England. Further research was recommended on peer support, combined acupuncture with antidepressants, differential effects of psychological interventions and withdrawal effects of antidepressants for which evidence was more limited, less applicable, or uncertain. Finally, this analysis is limited through the essentially unitary concept of ‘depression’ assessed within clinical trials, despite clinical wisdom and research evidence continuing to point out its heterogeneity.^48^, ^49^, ^50^ Clearer subtyping, associated with causal mechanisms (from basic biomarkers to higher order reinforcement learning), may provide enhanced predictive signals for more targeted clinical interventions and we recommend this as an area for future research investment.

## Contributors

OMV, IM, SP, SD, NJW and HP drafted the study protocol. SS and SA performed the literature searches. OMV and KJMOD assessed the eligibility of the studies for inclusion, extracted the data and assessed the risk of bias. SD, NJW, HP, CHD, EK, and DMC developed the statistical code and ran the statistical analyses. JT performed the machine learning methods of the 2023 review update and 2024 search update. All authors (IM, OMV, HP, NJW, SD, EW, NN, CHD, EK, HE, DMC, KJMOD, SS, SA, JT, NK, SP) contributed to the design of the study and the selection of studies for inclusion in the analysis and contributed to the interpretation of the findings. IM drafted the first draft of the manuscript, which all authors critically reviewed and contributed to, and approved its final version submitted for publication. All authors had full access to all the data in the study and had final responsibility for the decision to submit for publication.

## Data sharing statement

All data used in the study are within the paper and its Supporting Information files.

## Declaration of interests

HE, KJMOD, IM, OMV, SA, SS and SP received support from NICE for the submitted work. CHD, DMC, HP, NJW, EK, and SD received support from the NICE Guidelines TSU for the submitted work. EW, NK and NN declared the following interests based on NICE's policy on conflicts of interests: https://www.nice.org.uk/guidance/ng222/documents/register-of-interests. The authors report no other relationships or activities that could appear to have influenced the submitted work.

## Appendix A Supplementary data

Appendix 1 Methods

Appendix 2 2023 Review update methods

Appendix 3 Included & excluded studies

Appendix 4 Networks, treatments, Nrandomised

Appendix 5 Data files and Nstudies per comparison

Appendix 6 Results

Appendix 7 All NMA effects and direct comparisons

Appendix 8 Effects vs ref all outcomes intervention level

Appendix 9 2023 Review update results

Appendix 10 2023 Review update NMA data & SMD effects

Appendix 11 2024 Search update results
