# Supplementary material for: A systematic review and network meta-analysis of psychological, psychosocial, pharmacological, physical and combined treatments for adults with a new episode of depression
Source: eClinicalMedicine. 2024 Aug 16;75:102780. doi: 10.1016/j.eclinm.2024.102780 (PMC11377144; doi:10.1016/j.eclinm.2024.102780)
Supplement: Appendix 1 Methods [file mmc1.pdf]

APPENDIX 1

CONTENTS

Study protocol..3
Search strategy..14
Treatment classes and interventions considered in the NMA..20
Psychological interventions..20
Behavioural therapies..20
Cognitive and cognitive behavioural therapies..21
Problem solving..22
Counselling..22
Interpersonal psychotherapy..23
Short-term psychodynamic psychotherapies..23
Long-term psychodynamic psychotherapies..24
Psychoeducational interventions..24
Self-help (without support or with minimal support)..24
Self-help with support..25
Music therapy..25
Pharmacological interventions..26
Physical interventions..26
Acupuncture..26
Exercise..27
Yoga..27
Light therapy..27
Psychosocial interventions..28
Mindfulness, meditation or relaxation..28
Peer support..28
Control classes and interventions..28
Categorising depressive symptom severity into less and more severe depression..**31**
Methods of the statistical analysis and codes for data synthesis..**36**
Methods of the statistical analysis..36
Class effect models..38
Selection of reference treatments for presentation of results..42
Model assumptions..43
SMD analysis: methods..45
Response analysis: methods..53
Information on within-study correlation and standard deviation at follow-up..55
Discontinuation and remission analyses..57

OpenBUGS codes..57
Methods of inconsistency checks and statistical codes...66
Methods of inconsistency checks 66
Statistical codes for global inconsistency checks..68
Methods of bias adjustment models and statistical codes.......................................79
Methods of bias adjustment models 79
Statistical codes for bias adjustment 81
References 97

Study protocol

Topic	First-line treatment for adults with depression
Review questions	RQ. 2.1 For adults with a new episode of less severe depression, what are the relative benefits and harms of psychological, psychosocial, pharmacological and physical interventions alone or in combination?<br><br>RQ. 2.2. For adults with a new episode of more severe depression, what are the relative benefits and harms of psychological, psychosocial, pharmacological and physical interventions alone or in combination?
Objectives	To identify the most effective first-line interventions for the treatment of a new episode of depression
Population	Adults receiving first-line treatment for a new episode of depression, as defined by a diagnosis of depression according to DSM, ICD or similar criteria, or depressive symptoms as indicated by baseline depression scores on validated scales (and including those with subthreshold [just below threshold] depressive symptoms)<br>If some, but not all, of a study's participants are eligible for the review, for instance, mixed anxiety and depression diagnoses, then we will include a study if at least 80% of its participants are eligible for this review.<br>Baseline mean scores are used to classify study population severity according to less severe (RQ 2.1) or more severe (RQ 2.2) using the thresholds outlined below. These thresholds are derived using standardization of depression measurement crosswalk tables (Wahl 2014; Rush 2003; Carmody 2006; Uher 2008). An anchor point of 16 on the PHQ-9 was selected on the basis of alignment with the clinical judgement of the committee and eligibility criteria in published studies. If baseline mean scores are not available, severity will be classified according to the inclusion criteria of the study or the description given by the study authors (but only in cases where this is unambiguous, for example 'severe' or 'subthreshold' or 'mild').<br><br>Severity thresholds:

Scale	Threshold
HAMD (17-item, 21-item and 24-item)	16
MADRS (10-item)	22
PHQ-9	16
BDI-I (21-item)	22
BDI-II (21-item)	30
CES-D (20-item)	36
QIDS (16-item)	12
HADS-D (7-item)	12

Topic	First-line treatment for adults with depression
Exclude	• Trials of women with antenatal or postnatal depression<br>• Trials of children and young people (mean age under 18 years)<br>• Trials of people with learning disabilities<br>• Trials of people with bipolar disorder<br>• Trials of adults in contact with the criminal justice system (not solely as a result of being a witness or victim)<br>• Trials where more than 20% of the population have psychotic symptoms<br>• Trials where more than 20% of the population have a coexisting personality disorder<br>• Trials where more than 20% of the population have chronic depression (chronic depression defined as depression for at least 2 years, or persistent subthreshold symptoms [dysthymia], or double depression [an acute episode of major depressive disorder superimposed on dysthymia])<br>• Trials of further-line treatment<br>• Trials of people with Seasonal Affective Disorder (SAD)<br>Trials that specifically recruit participants with a physical health condition in addition to depression (e.g. depression in people with diabetes)
Intervention	The following interventions will be included:<br><br>**Psychological interventions**:<br><br>• Behavioural therapies (including behavioural activation, behavioural therapy [Lewinsohn 1976], coping with depression group)<br>• Cognitive and cognitive behavioural therapies (including CBT individual or group [defined as under or over 15 sessions], problem solving, rational emotive behaviour therapy [REBT] and third-wave cognitive therapies individual or group)<br>• Counselling (including emotion-focused therapy [EFT], non-directive/supportive/ person-centred counselling and relational client-centred therapy)<br>• Interpersonal psychotherapy<br>• Psychodynamic psychotherapies (including individual or group-based short-term psychodynamic psychotherapy, long-term psychodynamic psychotherapy and psychodynamic counselling)<br>• Psychoeducational interventions (including psychoeducational group programmes)<br>• Self-help with or without support (including cognitive bibliotherapy with or without support, computerised CBT [CCBT] with or without support, computerised psychodynamic therapy with or without support)<br>• Art therapy<br>• Music therapy<br>• Eye movement desensitization and reprocessing (EMDR) (for depression, not PTSD)

Topic	First-line treatment for adults with depression
	The following interventions are more appropriate for subgroups of adults with depression and as such will be considered only in pairwise comparisons (and not included in the NMA):<br><br>• Couple interventions, including behavioural couples therapy (for people with problems in the relationship with their partner)<br><br>**Pharmacological interventions**:<br><br>To be included, pharmacological interventions needed to be licensed in the UK and in routine clinical use for the first-line treatment of depression.<br><br>SSRIs<br><br>• Citalopram<br>• Escitalopram<br>• Paroxetine<br>• Sertraline<br>• Fluoxetine<br><br>TCAs<br><br>• Amitriptyline<br>• Clomipramine<br>• Lofepramine<br>• Nortriptyline<br>• Note: To improve connectivity, imipramine will be included in the network (because it has been used as a control in many trials) however it will not be considered as part of the decision problem<br><br>SNRIs<br><br>• Venlafaxine<br>• Duloxetine

Topic	First-line treatment for adults with depression
	Other antidepressant drugs:<br><br>• Mirtazapine<br>• Trazodone<br><br>Note that if necessary for connectivity in the network specific drugs that are excluded and 'any antidepressant' or 'any SSRI' or 'any TCA' nodes will be added where they have been compared against a psychological or physical intervention and/or combined with a psychological or physical intervention but they will not be considered as part of the decision problem.<br><br>**Physical interventions**:<br><br>• Acupuncture<br>• Exercise (including yoga)<br>• Light therapy (for depression, not SAD)<br><br>**Psychosocial interventions**:<br><br>• Peer support (including befriending, mentoring, and community navigators)<br>• Mindfulness, meditation or relaxation (including mindfulness-based stress reduction [MBSR])
Comparison	• Other active intervention (must also meet inclusion criteria above)<br>• Treatment as usual (TAU)<br>• Waitlist<br>• No treatment<br>• Placebo<br><br>If a study compares 'intervention + TAU vs TAU alone' it will be recoded as 'intervention vs no treatment'
Outcomes	**Critical outcomes:**<br><br>**Efficacy**<br><br>• Depression symptomatology (mean endpoint score or change in depression score from baseline)

Topic	First-line treatment for adults with depression
	• Remission (usually defined as a cut off on a depression scale), this will be analysed for those randomised and for completers<br>• Response (usually defined as at least 50% improvement from the baseline score on a depression scale), this will be analysed for those randomised and for completers<br><br>The following depression scales will be included in the following hierarchy:<br><br>• MADRS<br>• HAMD<br>• QIDS<br>• PHQ<br>• CGI (for dichotomous outcomes only)<br>• CES-D<br>• BDI<br>• HADS-D (depression subscale)<br>• HADS (full scale)<br>Only one continuous scale will be used per study<br><br>• For studies reporting response and/or remission, the scale used in the study to define cut-offs for response and/or remission will be used<br>• If more than one definition is used, a hierarchy of scales will be adopted (hierarchy listed above)<br><br>For studies not reporting dichotomous data, a hierarchy of scales (see above) will be adopted for continuous outcomes<br><br>**Acceptability/tolerability**<br><br>• Discontinuation due to side effects (for pharmacological trials)<br>• Discontinuation due to any reason (including side effects)<br><br>**Important, but not critical, outcomes**:<br><br>• Quality of life<br>• Quality of life (as assessed with a validated scale, including the 12-item/36-item Short-Form Survey [SF-12/SF-36], 26-item short version of the World Health Organization Quality of Life assessment

Topic	First-line treatment for adults with depression
	[WHOQOL-BREF], EuroQoL [EQ5D], Quality of Life Depression Scale [QLDS], Quality of Life Enjoyment and Satisfaction Questionnaire [Q-LES-Q], Quality of Life Inventory [QoLI], and World Health Organization 5-item Well-Being Index [WHO-5])<br>• Personal, social, and occupational functioning<br>• Global functioning (as assessed with a validated scale, including Global Assessment of Functioning [GAF], Global Assessment Scale [GAS], and Social and Occupational Functioning Assessment Scale [SOFAS])<br>• Functional impairment (as assessed with a validated scale, including Sheehan Disability Scale [SDS], Social Adjustment Scale [SAS], and Work and Social Adjustment Scale [WSAS])<br>• Sleeping difficulties (as assessed with a validated scale, including Insomnia Severity Index [ISI] and Pittsburgh Sleep Quality Index [PSQI])<br>• Employment (for instance, % unemployed)<br>• Interpersonal problems (as assessed with a validated scale, including Inventory of Interpersonal Problems [IIP])<br>Outcomes will be assessed at endpoint and follow-up (data for all available follow-up periods of at least 1-month post-intervention will be extracted and will be grouped into categories for analysis, for instance, 1-3 months, 4-6 months, 7-9 months, 10-12 months, 13-18 months, 19-24 months, and >2 years).
Study design	• RCTs<br>• Systematic reviews of RCTs
Include unpublished data?	Conference abstracts, dissertations and unpublished data will not be included unless the data can be extracted from elsewhere (for instance, from the previous guideline)
Restriction by date	All relevant studies from existing reviews from the 2009 guideline and from previous searches (pre-2016) will be carried forward. Studies published between 2016 and the date the searches are run will be sought.
Minimum sample size	N = 10 in each arm<br>Studies with <50% completion data (drop out of >50%) will be excluded.
Study setting	Primary, secondary, tertiary and social care settings.<br>Non-English-language papers will be excluded (unless data can be obtained from an existing review).
The review strategy	**Data Extraction (selection and coding)**<br>Citations from each search will be downloaded into EndNote and duplicates removed. Titles and abstracts of identified studies will be screened by two reviewers for inclusion against criteria, until a good inter-rater reliability has been observed (percentage agreement =>90%). Initially 10% of references will be double-screened. If inter-rater agreement is good then the remaining references will be screened by one reviewer. All primary-level studies included after the first scan of citations will be acquired in full and re-evaluated for eligibility at the time

Topic	First-line treatment for adults with depression
	they are being entered into a study database (standardised template created in Microsoft Excel). At least 10% of data extraction will be double-coded. Discrepancies or difficulties with coding will be resolved through discussion between reviewers or the opinion of a third reviewer will be sought.<br><br>**Data Analysis**<br>Pairwise comparisons (meta-analyses using random-effects models) will be conducted to combine results from similar studies. An intention to treat (ITT) approach will be taken where possible.<br><br>Network meta-analysis (NMA) in a Bayesian framework will also be used to synthesise the data for all eligible interventions which are connected in a network of RCT comparisons. Interventions with similar effects (as determined by the committee) will be grouped into classes and class effects models will be fitted [Dias 2018]. The relative effects of the interventions within each class will be assumed to be distributed around a common class mean with a within-class variance, permitting the borrowing of strength across interventions within each class.<br><br>Classes which do not have enough evidence to estimate within-class variability of effects (i.e., a class with just 1 or 2 interventions) will share within-class variability with similar classes (as determined by the committee) where the variance can be estimated. For example, the individual cognitive and CBT class may borrow the within-class variance from the individual behavioural therapies class. If no such similar class is identified, we will assume zero variance in classes with only 1 or 2 interventions. In addition, the attention placebo, no treatment and TAU classes will share a within-class variance. If an ‘any antidepressant’ class is required to connect otherwise disconnected/excluded drugs to the network (as described under Intervention topic), its within-class variance will be equal to the maximum of the SSRI and TCA within-class variances.<br><br>The random class effects assumption will be assessed by comparing the fit of fixed and random class effects models, where the former assumes the intervention effects within each class are the same (i.e., no within-class variability of effects).<br><br>Continuous outcomes (SMDs) will be combined with dichotomous data to estimate intervention effects, using the methods described in the Appendix. The NMA will probably be restricted to critical outcomes at endpoint due to the likelihood of a lack of connectivity in a follow-up data network or in a network for important (but not critical) outcomes.<br><br>The consistency of direct and indirect evidence will be assessed by fitting and comparing the fit of the NMA and unrelated mean effects (UME) models, the latter of which is equivalent to having separate, unrelated, meta-

Topic	First-line treatment for adults with depression
	analyses for every pairwise contrast [Dias 2011]. Each data point's contribution to the posterior mean residual deviance for the NMA model will be plotted against that for the UME model, to visually assess if specific data points are contributing to inconsistency. If the UME suggests there is evidence of inconsistency, node-split models will be fitted to assist in identifying loops of evidence with inconsistency [Dias 2010].<br><br>Risk of bias will be assessed at the study level using the Cochrane risk of bias tool. This assessment includes: adequacy of randomisation (sufficient description of randomisation method, allocation concealment and any baseline difference between groups); blinding (of participants, intervention administrators and outcome assessors); attrition ('at risk of attrition bias' defined as a dropout of more than 20% and completer analysis used, or a difference of >20% between the groups); selective reporting bias (is the protocol registered, are all outcomes reported); other bias (for instance, conflict of interest in funding).<br><br>Risk of bias will also be assessed at the outcome level using GRADE. For heterogeneity, outcomes will be downgraded once if I2>50%, twice if I2 >80%. For imprecision, outcomes will be downgraded using rules of thumb. If the 95% CI is imprecise i.e. crosses the line of no effect and the threshold for clinical benefit/harm, 0.8 or 1.25 (dichotomous) or -0.5 or 0.5 SMD (for continuous), the outcome will be downgraded. Outcomes will be downgraded one or two levels depending on how many lines it crosses. If the 95% CI is not imprecise, we will consider whether the criterion for Optimal Information Size is met (for dichotomous outcomes, 300 events; for continuous outcomes, 400 participants), if not we will downgrade one level.
Heterogeneity<br>(sensitivity analysis and subgroups)	Where possible, the influence of the following subgroups will be considered:<br>• Primary care compared to secondary care<br>• Inpatient compared to outpatient settings<br>• Older adults (60 years and older) compared to younger adults (younger than 60 years)<br>• BME populations<br>• Men<br><br>If the network structure allows, sensitivity analyses will be considered for depression symptoms (SMD, the primary outcome for the clinical analysis) and discontinuation for any reason and response in completers (the main outcomes for economic analysis), as follows:<br>• Risk of bias as reflected by publication bias and study size using methods described in [Dias 2010]. We will assume possible bias in comparisons of active interventions vs inactive control and no bias between inactive control comparisons, as well as active intervention comparisons, except in comparisons where counselling is the control intervention (in which case bias against counselling will be assumed)

Topic	First-line treatment for adults with depression
	• Validity of transitivity assumption will be explored by sensitivity analysis on SMD outcome that includes non-pharmacological trials only and examines any differences in magnitude of effects and ranking of non-pharmacological interventions compared to results from the mixed psychological, psychosocial, pharmacological and physical model<br><br>Threshold analysis will be performed to assess the robustness of intervention recommendations due to bias [Phillippo 2018].
Notes	For interventions in the NMA it is assumed that any patient that meets all inclusion criteria is, in principle, equally likely to be randomised to any of the interventions in the synthesis comparator set.<br><br>For defining routine usage of drugs, the national prescription cost data for England in 2017 - the most recent year for which relevant data existed - (Prescribing & Medicines Team, Health and Social Care Information Centre, 2017) was used. If a drug appeared in the top 15 it was included, with the exception of dosulepin which the BNF indicates should be initiated by a specialist.<br><br>Cipriani 2018 network meta-analysis will be used as a source for studies and data.<br><br>References for crosswalk tables:<br>Carmody, T. J., Rush, A. J., Bernstein, I., et al. (2006). The Montgomery Äsberg and the Hamilton ratings of depression: a comparison of measures. European Neuropsychopharmacology, 16(8), 601-611.<br><br>Rush, A. J., Trivedi, M. H., Ibrahim, H. M., et al. (2003). The 16-Item Quick Inventory of Depressive Symptomatology (QIDS), clinician rating (QIDS-C), and self-report (QIDS-SR): a psychometric evaluation in patients with chronic major depression. Biological psychiatry, 54(5), 573-583.<br><br>Uher, R., Farmer, A., Maier, W., et al. (2008). Measuring depression: comparison and integration of three scales in the GENDEP study. Psychological medicine, 38(2), 289-300.<br><br>Wahl, I., Löwe, B., Bjorner, J. B., et al. (2014). Standardization of depression measurement: a common metric was developed for 11 self-report depression measures. Journal of clinical epidemiology, 67(1), 73-86.<br><br>Assuming a normal distribution and using baseline mean and standard deviation data, we will explore the categorisation of less and more severe, including the percentage of studies 'definitely' within the correct category (≥70% of the study sample above cut-off) in order to aid the committee in interpreting the results.

Topic	First-line treatment for adults with depression
	References for data analysis:<br>Dias, S., Ades, A.E., Welton, N.J., et al. (2018). Network meta-analysis for decision making. Hoboken, NJ: Wiley.<br><br>Dias, S., Welton, N.J., Sutton, A.J., et al. (2011). NICE DSU Technical Support Document 4: Inconsistency in networks of evidence based on randomised controlled trials.<br><br>Dias, S., Welton, N.J., Caldwell, D.M., Ades A.E. (2010a). Checking consistency in mixed treatment comparison meta-analysis. Statistics in Medicine, 29(7-8), 932-44.<br><br>References for heterogeneity:<br>Dias, S., Welton, N.J., Marinho, V.C.C., et al. (2010b). Estimation and adjustment of bias in randomised evidence by using mixed treatment comparison meta-analysis. Journal of the Royal Statistical Society: Series A (Statistics in Society), 173(3), 613-29.<br><br>Phillippo, D.M., Welton, N.J., Dias, S., et al. (2018). Sensitivity of treatment recommendations to bias in network meta-analysis. Journal of the Royal Statistical Society: Series A (Statistics in Society), 181(3), 843-67.
Information sources – databases and dates	Database(s): Embase 1974 to Present, Ovid MEDLINE(R) In-Process & Other Non-Indexed Citations and Ovid MEDLINE(R) 1946 to Present; Cochrane Library; WEB OF SCIENCE
Identify if an update	Update of CG90 (2009)
Author contacts	For details please see the guideline in development web site.
Highlight if amendment to previous protocol	For details please see section 4.5 of Developing NICE guidelines: the Manual, 2014
Search strategy – for one database	For details please see appendix.
Data collection process – forms/duplicate	A standardised evidence table format will be used.
Data items – define all variables to be collected	For details please see evidence tables in appendix.
Methods for assessing bias at outcome/study level	Standard study checklists were used to critically appraise individual studies. For details please see section 6.2 of Developing NICE guidelines: the Manual, 2014.

Topic	First-line treatment for adults with depression
	The risk of bias across all available evidence was evaluated for each outcome using an adaptation of the 'Grading of Recommendations Assessment, Development and Evaluation (GRADE) toolbox' developed by the international GRADE working group.
Criteria for quantitative synthesis	For details please see section 6.4 of Developing NICE guidelines: the Manual, 2014
Methods for quantitative analysis – combining studies and exploring (in)consistency	For details please see the methods chapter.
Meta-bias assessment – publication bias, selective reporting bias	For details please see section 6.2 of Developing NICE guidelines: the Manual, 2014.
Confidence in cumulative evidence	For details please see sections 6.4 and 9.1 of Developing NICE guidelines: the Manual, 2014
Rationale/context – what is known	For details please see the introduction to the evidence review.
Describe contributions of authors and guarantor	A multidisciplinary committee developed the evidence review. The committee was convened by the National Guideline Alliance (NGA) and chaired by Dr Navneet Kapur in line with section 3 of Developing NICE guidelines: the Manual, 2014.<br>Staff from the NGA undertook systematic literature searches, appraised the evidence, conducted meta-analysis and cost effectiveness analysis where appropriate, and drafted the guideline in collaboration with the committee. For details please see the methods chapter.
Sources of funding/support	The NGA is funded by NICE and hosted by the Royal College of Obstetricians and Gynaecologists.
Name of sponsor	The NGA is funded by NICE and hosted by the Royal College of Obstetricians and Gynaecologists.
Roles of sponsor	NICE funds NGA to develop guidelines for those working in the NHS, public health and social care in England
PROSPERO registration number	CRD42019151328

BDI: Beck depression inventory; BME: black minority ethnic; BNF: British national formulary; (C)CBT: (computerised) cognitive behavioural therapy; CDSR: Cochrane Database of Systematic Reviews; CENTRAL: Cochrane Central Register of Controlled Trials; CES-D: Centre of epidemiology studies – depression; CGI: clinical global impressions; CI: confidence interval; DARE: Database of Abstracts of Reviews of Effects; DSM: Diagnostic and statistical manual; EFT: emotion-focused therapy; EMDR: eye movement desensitization and reprocessing; EQ-5D: European quality of life 5 dimensions; GAF: global assessment of functioning; GAS: global assessment scale; GRADE: Grading of Recommendations Assessment, Development and Evaluation; HADS-D: hospital anxiety and depression scale – depression; HAMD: Hamilton Depression Rating Scale; ICD: International classification of diseases; IIP: inventory of interpersonal problems; ISI: insomnia severity index; ITT: intention to treat; MADRS: Montgomery–Åsberg Depression Rating Scale; MBSR: Mindfulness-based stress reduction; MID: minimally important difference; NGA: National Guideline Alliance; NHS: National health service; NICE: National Institute for Health and Care Excellence; NMA: network meta-analysis; PHQ-9: patient health questionnaire-9; PSQI: Pittsburgh sleep quality index; PTSD: post-traumatic stress disorder; QIDS: quick inventory of depressive symptomatology; QLDS: quality of life depression scale; Q-LES-Q: quality of life enjoyment and satisfaction questionnaire QOLI: quality of life inventory RCT: randomised controlled trial; REBT: rational emotive behaviour therapy; RoB: risk of bias; SAD: seasonal affective disorder; SAS: Spielberger state/trait anxiety scale; SDS: Sheehan disability scale; SMD: standardised mean difference; SNRI: serotonin-noradrenaline reuptake inhibitor; SOFAS: social and occupational functioning assessment scale; SSRI: selective serotonin reuptake inhibitor; TAU: treatment as usual; TCA: tricyclic antidepressant; UME: unrelated mean effects; WHOQOL-BRIEF: World health organization quality of life assessment (brief); WHO-5: world health organization 5-item wellbeing index; WSAS: work and social adjustment scale

Search strategy

Database(s): Embase 1974 to 2019 Week 19, Emcare 1995 to present, Ovid MEDLINE(R) and Epub Ahead of Print, In-Process & Other Non-Indexed Citations and Daily 1946 to May 14, 2019, PsycINFO 1806 to May Week 1 2019

Database segment codes:
oemez = Embase
emcr = Emcare
ppez = Ovid MEDLINE(R) and Epub Ahead of Print, In-Process & Other Non-Indexed Citations and Daily
psyh = PsycINFO

Date of first search: 16/05/2019

Date of search updated: 04/06/2020

#	Searches
1	(depression/ or agitated depression/ or atypical depression/ or depressive psychosis/ or dysthymia/ or endogenous depression/ or involutional depression/ or late life depression/ or major depression/ or masked depression/ or melancholia/ or "mixed anxiety and depression"/ or reactive depression/ or recurrent brief depression/ or treatment resistant depression/) use oemezd,emcr
2	(Depression/ or Depressive Disorder/ or Depressive Disorder, Major/ or Depressive Disorder, Treatment-Resistant/ or Disorders, Psychotic/ or Dysthymic Disorder/) use ppez
3	("depression (emotion)"/ or exp major depression/ or affective disorders/ or atypical depression/) use psyh
4	(depress* or dysthym* or melanchol* or ((affective or mood) adj disorder*)).tw.
5	((sever* or serious* or major* or chronic* or complex* or critical* or endur* or persist* or resist* or acute) adj2 (anxiety or (mental adj2 (disorder* or health or illness* or ill-health)) or (obsessive adj2 disorder*) or OCD or panic attack* or panic disorder* or phobi* or personality disorder* or psychiatric disorder* or psychiatric illness* or psychiatric ill-health*)).tw.
6	or/1-5
7	(exp psychotherapy/ or exp counseling/ or mindfulness/ or problem solving/ or psychiatric treatment/ or psychoeducation/ or self help/ or exp support group/) use oemezd,emcr
8	(exp Psychotherapy/ or Bibliotherapy/ or exp Cognitive Behavioral Therapy/ or exp Counseling/ or Problem Solving/ or Self Care/ or Self Efficacy/ or Self-Help Groups/) use ppez
9	(exp psychotherapy/ or behavioral activation system/ or bibliotherapy/ or cognitive therapy/ or exp counseling/ or group intervention/ or mindfulness/ or exp problem solving/ or psychoeducation/ or exp self-help techniques/ or support groups/) use psyh
10	((behavio* or abreact* or act* out* or age regression or assertive or autogenic or experiential) adj2 (activation or analys* or cathar* or condition* or intervention* or modification* or therap* or training or treatment*)).tw.
11	((cognitive adj2 (behavior* or therap*)) or (CBT* or CBASP or biofeedback or contingency management or covert conditioning or covert sensiti?ation or defusion or MBCT* or neurofeedback or problem focus* or problem solving or rational emotive or REBT or schema or solution focus*) or ((third wave or 3rd wave) adj2 (intervention* or therap* or treatment*))).tw.
12	(counsel* or ((art or creative or compassion* or conversation* or dialectic* or emotion* or group* or insight or narrative or non-directive or nondirective or non-specific or nonspecific or rational or client-centred or client-centered or humanistic or integrative or interpersonal or person-centred or person-centered or personal construct or persuasion or Rogerian or talking or time-limited) adj2 (intervention* or therap* or training or treatment*))).tw.
13	(psychotherap* or (psycho* adj (aid* or help* or intervention* or support* or therap* or training or treatment*)) or (balint group or group program* or mindfulness* or mind training or role play* or support group*)).tw.
14	(self-help or bibliotherap* or meditat* or self-analy* or self-esteem or self-control or self-imag* or self-validat* or stress manag* or (computer* adj2 (intervention* or program* or therap* or treatment*)) or CCBT).tw.
15	or/7-14
16	drug therapy/ or drug therapy.fs.
17	psychopharmacotherapy/ use oemezd,emcr,psyh

#	Searches
18	antidepressant agent/ use oemezd,emcr
19	Antidepressive Agents/ use ppez
20	antidepressant drugs/ use psyh
21	serotonin uptake inhibitor/ use oemezd,emcr
22	Serotonin Uptake Inhibitors/ use ppez
23	serotonin reuptake inhibitors/ use psyh
24	serotonin noradrenalin reuptake inhibitor/ use oemezd,emcr
25	"Serotonin and Noradrenaline Reuptake Inhibitors"/ use ppez
26	serotonin norepinephrine reuptake inhibitors/ use psyh
27	tricyclic antidepressant agent/ use oemezd,emcr
28	Antidepressive Agents, Tricyclic/ use ppez
29	tricyclic antidepressant drugs/ use psyh
30	monoamine oxidase inhibitor/ use oemezd,emcr
31	monoamine oxidase inhibitors/ use ppez,psyh
32	tetracyclic antidepressive agent/ use oemezd,emcr
33	amfebutamone/ or amineptine/ or amitriptyline/ or bupropion/ or clomipramine/ or chlorimipramine/ or citalopram/ or desipramine/ or duloxetine/ or Duloxetine Hydrochloride/ or escitalopram/ or fluvoxamine/ or fluoxetine/ or imipramine/ or lofepramine/ or mianserin/ or mirtazapine/ or moclobemide/ or nefazadone/ or nortriptyline/ or paroxetine/ or phenelzine/ or sertraline/ or venlafaxine/ or Venlafaxine Hydrochloride/
34	(antidepress* or amfebutamone or amineptin* or amitr?ptylin* or bupropion or chlorimipramine or clomipramin* or citalopram or desipramin* or duloxetin* or escitalopram or fluvoxamin* or fluoxetin* or imipramin* or lofepramin* or mianserin or mirtazapin* or moclobemide or nefazadon* or nortriptylin* or paroxetin* or phenelzin* or psychopharmacologic* or psychopharmacotherap* or sertralin* or venlafaxin* or SNRI* or SSRI* or TCA* or TeCA* or tetracyclic or tricyclic or ((monoamine or serotonin) adj2 inhibitor*)).tw.
35	or/16-34
36	(anticonvulsive agent/ or anticonvulsant therapy/) use oemezd,emcr
37	Anticonvulsants/ use ppez
38	anticonvulsive drugs/ use psyh
39	lamotrigine/ or (lamotrigine or anticonvul* or anti-convul*).tw.
40	or/38-39
41	neuroleptic agent/ use oemezd,emcr
42	Antipsychotic Agents/ use ppez
43	neuroleptic drugs/ use psyh
44	amisulpride/ or aripiprazole/ or olanzapine/ or quetiapine/ or Quetiapine Fumarate/ or risperidone/ or ziprasidone/
45	(antipsychotic* or anti-psychotic* or amisulpride or aripiprazole or olanzapine or psychotropic* or quetiapine or risperidone or ziprasidone).tw.
46	or/41-45
47	anxiolytic agent/ use oemezd,emcr
48	Anti-Anxiety Agents/ use ppez
49	tranquilizing drugs/ use psyh
50	buspirone/
51	(anxiolytic* or antianxiet* or anti-anxiet* or tranquili* or buspirone).tw.
52	or/47-51
53	central stimulant agent/ use oemezd,emcr
54	Central Nervous System Stimulants/ use ppez
55	CNS stimulating drugs/ use psyh
56	methylphenidate/ or (methylphenidate or ritalin).tw.
57	or/53-56
58	lithium/ or lithium.tw.
59	omega 3 fatty acid/ use oemezd,emcr
60	Fatty Acids, Omega-3/ use ppez
61	fatty acids/ use psyh
62	(omega adj ("fatty acid*" or "polyunsaturated fatty acid*" or PUFA*)).tw.
63	thyroid hormone/ use oemezd,emcr
64	Thyroid Hormones/ use ppez
65	exp thyroid hormones/ use psyh
66	(thyroid hormone* or calcitonin or dextrothyroxine or diiodotyrosine or monoiodotyrosine or thyronines or thyroxine).tw.
67	or/58-66

#	Searches
68	acupuncture/ or acupuncture.tw.
69	electroconvulsive therapy/ use oemezd,emcr,ppez
70	electroconvulsive shock therapy/ use psyh
71	(ECT or ((electroconvuls* or electro-convuls*) adj2 (therap* or treatment*)) or electroshock* or (shock adj (therap* or treatment*))).tw.
72	exp exercise/
73	(exp Exercise Therapy/ or Physical Exertion/ or exp Physical Fitness/ or Bicycling/ or exp Running/ or Swimming/ or Walking/) use ppez
74	(exp kinesiotherapy/ or exp physical activity/ or fitness/ or exp sport/) use oemezd,emcr
75	(exp physical fitness/ or exp sports/) use psyh
76	yoga/
77	(exercis* or yoga or cycling or bicycling or jogging or running or sport* or swimming or walking).tw.
78	or/68-77
79	peer group/ or mentoring/
80	peer relations/ use psyh
81	friendship/
82	Friends/ use ppez
83	(befriend* or friend* or mentor* or peer group* or peer support or (communit* adj (navigat* or support*))).tw.
84	or/79-83
85	or/15,35,40,46,52,57,67,78,84
86	6 and 85
87	Letter/ use ppez
88	letter.pt. or letter/ use oemezd,emcr
89	note.pt.
90	editorial.pt.
91	Editorial/ use ppez
92	News/ use ppez
93	exp Historical Article/ use ppez
94	Anecdotes as Topic/ use ppez
95	Comment/ use ppez
96	Case Report/
97	case study/ use oemezd,emcr
98	(letter or comment*).ti.
99	or/87-98
100	randomized controlled trial/
101	random*.ti,ab.
102	100 or 101
103	99 not 102
104	(animals/ not humans/) use ppez
105	(animal/ not human/) use oemezd,emcr
106	nonhuman/ use oemezd,emcr
107	exp animals/ use psyh
108	"primates (nonhuman)"/ use psyh
109	exp Animals, Laboratory/ use ppez
110	exp Animal Experimentation/ use ppez
111	exp animal experiment/ use oemezd,emcr
112	exp experimental animal/ use oemezd,emcr
113	exp Models, Animal/ use ppez
114	animal model/ use oemezd,emcr
115	animal models/ use psyh
116	animal research/ use psyh
117	exp Rodentia/ use ppez
118	exp rodent/ use oemezd,emcr
119	exp rodents/ use psyh
120	(rat or rats or mouse or mice).ti.
121	or/103-120
122	86 not 121
123	clinical Trials as topic.sh. or (controlled clinical trial or pragmatic clinical trial or randomized controlled trial).pt. or (placebo or randomi?ed or randomly).ab. or trial.ti.
124	123 use ppez

#	Searches
125	(controlled clinical trial or pragmatic clinical trial or randomized controlled trial).pt. or drug therapy.fs. or (groups or placebo or randomi?ed or randomly or trial).ab.
126	125 use ppez
127	crossover procedure/ or double blind procedure/ or randomized controlled trial/ or single blind procedure/ or (assign* or allocat* or crossover* or cross over* or ((doubl* or singl*) adj blind*) or factorial* or placebo* or random* or volunteer*).ti,ab.
128	127 use oemezd,emcr
129	clinical trials/ or (placebo or randomi?ed or randomly).ab. or trial.ti.
130	129 use psyh
131	124 or 126
132	128 or 130 or 131
133	Meta-Analysis/
134	exp Meta-Analysis as Topic/
135	systematic review/
136	meta-analysis/
137	(meta analy* or metanaly* or metaanaly*).ti,ab.
138	((systematic or evidence) adj2 (review* or overview*)).ti,ab.
139	((systematic* or evidence*) adj2 (review* or overview*)).ti,ab.
140	(reference list* or bibliograph* or hand search* or manual search* or relevant journals).ab.
141	(search strategy or search criteria or systematic search or study selection or data extraction).ab.
142	(search* adj4 literature).ab.
143	(medline or pubmed or cochrane or embase or psychlit or psyclit or psychinfo or psycinfo or cinahl or science citation index or bids or cancerlit).ab.
144	cochrane.jw.
145	((pool* or combined) adj2 (data or trials or studies or results)).ab.
146	(or/133-135,137,139-144) use ppez
147	(or/135-138,140-145) use oemezd,emcr
148	(or/133,137,139-144) use psyh
149	or/146-148
150	network meta-analysis/
151	((network adj (MA or MAs)) or (NMA or NMAs)).tw.
152	((indirect or mixed or multiple or multi-treatment* or simultaneous) adj1 comparison*).tw.
153	or/150-152
154	or/132,149,153
155	122 and 154
156	limit 155 to english language

Database: Cochrane Database of Systematic Reviews, Issue 5 of 12, May 2019; Cochrane Central Register of Controlled Trials, Issue 5 of 12, May 2019.

Date of first search: 21/05/2019

Date of search updated: 04/06/2020

ID	Search
#1	MeSH descriptor: [Depression] this term only
#2	MeSH descriptor: [Depressive Disorder] this term only
#3	MeSH descriptor: [Depressive Disorder, Major] this term only
#4	MeSH descriptor: [Depressive Disorder, Treatment-Resistant] this term only
#5	MeSH descriptor: [Affective Disorders, Psychotic] this term only
#6	MeSH descriptor: [Dysthymic Disorder] this term only
#7	(depress* or dysphori* or dysthym* or melanchol* or ((affective or mood) next disorder*)):ti,ab
#8	((sever* or serious* or major* or acute or chronic* or complex* or endur* or persist* or resist*) next/2 anxiety or (mental next/2 (disorder* or health or illness* or ill-health)) or (obsessive next/2 disorder*) or OCD or "panic attack*" or "panic disorder*" or phobi* or "personality disorder*" or "psychiatric disorder*" or "psychiatric illness*" or "psychiatric ill-health*"):ti,ab
#9	{or #1-#8}
#10	MeSH descriptor: [Psychotherapy] explode all trees
#11	MeSH descriptor: [Bibliotherapy] this term only
#12	MeSH descriptor: [Cognitive Behavioral Therapy] explode all trees
#13	MeSH descriptor: [Counseling] explode all trees

ID	Search
#14	MeSH descriptor: [Problem Solving] this term only
#15	MeSH descriptor: [Self Care] this term only
#16	MeSH descriptor: [Self Efficacy] this term only
#17	MeSH descriptor: [Self-Help Groups] this term only
#18	((behaviour* or behavior* or abreact* or "act* out*" or "age regression" or assertive or autogenic or experiential) next/2 (activation or analys* or cathar* or condition* or intervention* or modification* or therap* or training or treatment*)):ti,ab
#19	((cognitive next/2 (behavio* or therap*)) or (CBT* or CBASP or biofeedback or "contingency management" or "covert conditioning" or "covert sensitisation" or "covert sensiitization" or defusion or MBCT* or neurofeedback or "problem focus*" or "problem solving" or "rational emotive" or REBT or schema or "solution focus*") or (("third wave" or "3rd wave") next (intervention* or therap* or treatment*))):ti,ab
#20	(counsel* or ((art or creative or compassion* or conversation* or dialectic* or emotion* or group* or insight or narrative or non-directive or nondirective or non-specific or nonspecific or rational or client-centred or client-centered or humanistic or integrative or interpersonal or person-centred or person-centered or "personal construct*" or persuasion or Rogerian or talking or time-limited) next (intervention* or therap* or training or treatment*))):ti,ab
#21	(psychotherap* or (psycho* next (aid* or help* or intervention* or support* or therap* or training or treatment*)) or ("balint group*" or "group program*" or mindfulness* or "mind training" or "role play*" or "support group*")):ti,ab
#22	(self-help or bibliotherap* or meditat* or self-analy* or self-esteem or self-control or self-imag* or self-validat* or "stress manag*" or (computer* next/2 (intervention* or program* or therap* or treatment*)) or CCBT):ti,ab
#23	MeSH descriptor: [Drug Therapy] this term only
#24	MeSH descriptor: [Antidepressive Agents] this term only
#25	MeSH descriptor: [Serotonin Uptake Inhibitors] this term only
#26	MeSH descriptor: [Serotonin and Noradrenaline Reuptake Inhibitors] this term only
#27	MeSH descriptor: [Antidepressive Agents, Tricyclic] this term only
#28	MeSH descriptor: [Monoamine Oxidase Inhibitors] this term only
#29	MeSH descriptor: [Bupropion] this term only
#30	MeSH descriptor: [Amitriptyline] this term only
#31	MeSH descriptor: [Bupropion] this term only
#32	MeSH descriptor: [Clomipramine] this term only
#33	MeSH descriptor: [Clomipramine] this term only
#34	MeSH descriptor: [Citalopram] this term only
#35	MeSH descriptor: [Desipramine] this term only
#36	MeSH descriptor: [Duloxetine Hydrochloride] this term only
#37	MeSH descriptor: [Citalopram] this term only
#38	MeSH descriptor: [Fluvoxamine] this term only
#39	MeSH descriptor: [Fluoxetine] this term only
#40	MeSH descriptor: [Imipramine] this term only
#41	MeSH descriptor: [Lofepramine] this term only
#42	MeSH descriptor: [Mianserin] this term only
#43	MeSH descriptor: [Mirtazapine] this term only
#44	MeSH descriptor: [Moclobemide] this term only
#45	MeSH descriptor: [Nortriptyline] this term only
#46	MeSH descriptor: [Paroxetine] this term only
#47	MeSH descriptor: [Phenelzine] explode all trees
#48	MeSH descriptor: [Sertraline] this term only
#49	MeSH descriptor: [Venlafaxine Hydrochloride] this term only
#50	(antidepress* or amfebutamone or amineptin* or amitriptylin* or amitryptylin* or bupropion or chlorimipramine or clomipramin* or citalopram or desipramin* or duloxetin* or escitalopram or fluvoxamin* or fluoxetin* or imipramin* or lofepramin* or mianserin or mirtazapin* or moclobemide or nefazadon* or nortriptylin* or paroxetin* or phenelzin* or psychopharmacologic* or psychopharmacotherap* or sertralin* or venlafaxin* or SNRI* or SSRI* or TCA* or TeCA* or tetracyclic or tricyclic or ((monoamine or serotonin) next/2 inhibitor*)):ti,ab
#51	MeSH descriptor: [Anticonvulsants] this term only
#52	MeSH descriptor: [Lamotrigine] this term only
#53	(lamotrigine or anticonvul* or anti-convul*):ti,ab
#54	MeSH descriptor: [Antipsychotic Agents] this term only
#55	MeSH descriptor: [Amisulpride] this term only
#56	MeSH descriptor: [Aripiprazole] this term only
#57	MeSH descriptor: [Olanzapine] this term only

ID	Search
#58	MeSH descriptor: [Quetiapine Fumarate] this term only
#59	MeSH descriptor: [Risperidone] this term only
#60	(antipsychotic* or anti-psychotic* or amisulpride or aripiprazole or olanzapine or psychotropic* or quetiapine or risperidone or ziprasidone):ti,ab
#61	MeSH descriptor: [Anti-Anxiety Agents] this term only
#62	MeSH descriptor: [Buspirone] this term only
#63	(anxiolytic* or antianxiet* or anti-anxiet* or tranquilis* or tranquiliz* or buspirone):ti,ab
#64	MeSH descriptor: [Central Nervous System Stimulants] this term only
#65	MeSH descriptor: [Methylphenidate] this term only
#66	(methylphenidate or ritalin):ti,ab
#67	MeSH descriptor: [Lithium] this term only
#68	lithium:ti,ab
#69	MeSH descriptor: [Fatty Acids, Omega-3] explode all trees
#70	(omega next/2 ("fatty acid*" or "polyunsaturated fatty acid*" or PUFA*)):ti,ab
#71	MeSH descriptor: [Thyroid Hormones] explode all trees
#72	("thyroid hormone*" or calcitonin or dextrothyroxine or diiodotyrosine or monoiodotyrosine or thyronines or thyroxine):ti,ab
#73	MeSH descriptor: [Acupuncture] this term only
#74	acupuncture:ti,ab
#75	MeSH descriptor: [Electroconvulsive Therapy] this term only
#76	(ECT or ((electroconvuls* or electro-convuls*) next/2 (therap* or treatment*)) or electroshock* or (shock next (therap* or treatment*))):ti,ab
#77	MeSH descriptor: [Exercise Therapy] explode all trees
#78	MeSH descriptor: [Physical Exertion] this term only
#79	MeSH descriptor: [Physical Fitness] explode all trees
#80	MeSH descriptor: [Bicycling] this term only
#81	MeSH descriptor: [Running] explode all trees
#82	MeSH descriptor: [Swimming] this term only
#83	MeSH descriptor: [Walking] this term only
#84	MeSH descriptor: [Yoga] this term only
#85	(exercis* or yoga or cycling or bicycling or jogging or running or sport* or swimming or walking):ti,ab
#86	MeSH descriptor: [Peer Group] this term only
#87	MeSH descriptor: [Mentoring] this term only
#88	MeSH descriptor: [Friends] this term only
#89	(befriend* or friend* or mentor* or "peer group*" or "peer support" or (communit* next (navigat* or support*))):ti,ab
#90	{or #10-#89}

Treatment classes and interventions considered in the NMA

Due to the large number of different treatment options considered in this review, they have been grouped into classes to allow comparison between classes of treatment. Table 1 shows the classes and interventions included in each class (note that only classes and interventions with included data are listed in the table, although a comprehensive list of eligible interventions is outlined in the study protocol presented earlier in this Appendix). Combinations of interventions were also included in the review and were categorised in line with the classes and interventions outlined below.

Psychological interventions

Psychological therapies were grouped according to common theoretical structure and methodological approach. Separate classes were formed, where appropriate, for group versus individually delivered psychological interventions. We also aimed to explore potential differences in effects by intervention intensity, as reflected in the intended number of therapy sessions. This was only possible to explore for cognitive and cognitive behavioural therapies (CT/CBT), because there was large variation in the number of sessions reported across RCTs, and there was also a large evidence base that allowed a distinction between CBT ≥15 sessions and CBT<15 sessions, which were considered as separate interventions within the classes of both individual and group CT/CBT.

Behavioural therapies

In behavioural therapies, depression is seen as the result of a low rate of positive reinforcement and is maintained through negative reinforcement.[1] Most commonly, people use avoidance to minimise negative emotions and situations they worry will be unpleasant in the short-term, which may produce difficulties in the long-term. Behavioural therapies focus on behavioural activation aimed at encouraging people to develop more rewarding and task-focused behaviours as well as stepping out of patterns of negative reinforcement. The approach was developed by Lewinsohn[2] and there are still a group of therapies based on this traditional approach (referred to as ‘behavioural therapy [Lewinsohn 1976]’ in this

review). However, more recently there has also been a renewed interest in behavioural activation, as it is now known, as a therapy in its own right.

Another example of a specific intervention in this category that is linked by a common underlying philosophy is the Coping with Depression (CWD) course most frequently delivered in group format. The CWD course has similarities with psychoeducational group programmes but it was originally developed by Lewinsohn and colleagues[3] and has its roots in social learning theory, according to which depression is associated with a decrease in pleasant and an increase in unpleasant person-environment interactions.

Cognitive and cognitive behavioural therapies

The cognitive model[4] describes how, when depressed, people focus on negative views of themselves, the world, and the future. CBT takes an educative approach where, through collaboration, the person with depression learns to recognise his or her negative thinking patterns and to re-evaluate his or her thinking. This approach also requires people to practise re-evaluating their thoughts and new behaviours (homework). There is also an important emphasis on increasing activity and engaging in rewarding behaviours, as per behavioural activation, as well as the use of behavioural experiments to test underlying beliefs. As with any psychological treatment, cognitive behavioural therapy is not static and has been evolving, and in addition to the continued individual-format high-intensity CBT, CBT has also been delivered in a group format and in a low-intensity format. This review used the cut-off of 15 sessions to distinguish between a longer course of CBT (over 15 sessions) and briefer courses of CBT (under 15 sessions).

The principles of CBT also form the basis of a number of other stand-alone interventions that are grouped under this class. Drawing on common cognitive and cognitive behavioural principles although with a different emphasis (towards acceptance rather than directly targeting change) and with some different techniques are a newer wave or so-called third wave of cognitive therapies including Acceptance and Commitment Therapy (ACT) and Meta-Cognitive Therapy (MCT). Another, albeit older, variant of the traditional Beckian

cognitive behavioural approach is rational emotive behaviour therapy (REBT) which was developed by Ellis in the 1950s,[5] and which proponents believe may promote a deeper change through advocating unconditional self-acceptance, focusing explicitly on reducing secondary problems such as depression about depression (meta-emotions) and explicitly targeting demandingness (imperative or absolutistic demands on self, others, and life), the latter of which is considered the crucial component of depression. Positive psychotherapy (PPT) was also included in this class as cognitive restructuring techniques and behavioural exercises are used, although here the focus is shifted away from directly targeting depressive symptoms towards an emphasis on increasing positive emotion, engagement and meaning.[6]

Problem solving

Problem solving interventions, delivered both individually and in groups, are based on the theory that depression is associated with social problem-solving difficulties[7] which may relate to the effects of the depressed state, lack of knowledge, and/or rumination[8] and aims to help people solve problems and develop problem-solving skills[9] in order to improve depression symptoms. In some conceptualisations problem solving is considered a variant or dimension of CBT. However, it was categorised in a separate class as there are a number of studies (mainly from the US) which examine problem solving as a distinct stand-alone intervention.

Counselling

Counselling was developed by Carl Rogers[10] who believed that people had the means for self-healing, problem resolution and growth if the right conditions could be created. These conditions include the provision of positive regard, genuineness and empathy. Rogers's original model was developed into structured counselling approaches by Truax and Carkhuff[11] and, independently, by Egan[12] who developed the three stage model: exploration, personalizing, and action. Although many other therapies now use the basic ingredients of client-centred counselling,[13] there are differences in how they are used, for instance, emotion-focused therapy (EFT) and relational client-centred therapy. Counselling has

become a generic term used to describe a broad range of interventions that may include psychodynamic, systemic or cognitive behavioural elements.[14] More recently approaches to counselling have been developed which focus particularly on depression (for example, Counselling for Depression/Person-Centred Experiential Therapy [PCET]). However, all the evidence for counselling that was included in the review was non-directive counselling.

Interpersonal psychotherapy

Interpersonal therapy (IPT) was developed by Klerman and colleagues[15] initially for depression although it has now been extended to other disorders.[16] IPT focuses on current relationships, not past ones, and on interpersonal processes rather than intra-psychic ones (such as negative core beliefs or automatic thoughts as in CBT, or unconscious conflicts as in psychodynamic psychotherapy). It is time limited and focused on difficulties arising in the daily experience of maintaining relationships and resolving difficulties during an episode of major depression. Early in the treatment, patient and therapist agree to work on a particular focal area that would include: interpersonal role transitions, interpersonal roles/conflicts, grief and/or interpersonal deficits. The character of the therapy sessions is, largely, facilitating understanding of recent events in interpersonal terms and exploring alternative ways of handling interpersonal situations. Although there is not an explicit emphasis on ‘homework’, there is an emphasis on effecting changes in interpersonal relationships and tasks towards this end may be undertaken between sessions. Interpersonal counselling (IPC) is a brief intervention derived from IPT that was originally developed for people presenting in primary care with distress relating to current life stressors,[17] but IPC has since been tested as a stand-alone intervention for people with depression.

Short-term psychodynamic psychotherapies

Short-term psychodynamic psychotherapies are based on psychoanalytic techniques but may often be considerably briefer than psychoanalysis proper. Short-term psychodynamic psychotherapy considers the symptoms of depression as the result of core relationship conflicts predominately based on early experience and aims to help the person become

aware of the link between conflicts and symptoms using the therapeutic relationship as a central vehicle for insight and change. As with other schools of psychological therapy, there are a number of variations on the original model of psychodynamic psychotherapy. Some approaches focus on the dynamic of drives (for example, aggression) while others focus on relationships.[18] Other forms of this therapy have been influenced by attachment theory.[19] Dynamic Interpersonal Therapy (DIT)[20] is a time-limited psychodynamic therapy informed by attachment and mentalization theory, developed in response to the Improving Access to Psychological Therapies (IAPT) programme in order to provide a psychoanalytically informed intervention that could be delivered in a fixed number of sessions and offered as an alternative to CBT. Short-term psychodynamic psychotherapy is usually delivered individually but has also been tested in a group format.[21]

Long-term psychodynamic psychotherapies

A number of recent trials have examined a longer-term version of psychodynamic psychotherapy with treatment durations of up to three years. Long-term psychodynamic psychotherapy is an intensive, transference-based therapeutic approach and acts in a supportive-interpretive continuum (depending on the therapeutic needs of the patient) in order to explore and work through a broad range of intrapsychic and interpersonal conflicts.[22]

Psychoeducational interventions

Psychoeducation is a structured educational treatment (often offered in groups) that provides people with information about depression, often through a didactic format. These interventions are often informed by psychological principles and as such techniques from CBT and/or IPT are used, such as cognitive restructuring, pleasant event scheduling, role play, guided relaxation, and homework exercises.

Self-help (without support or with minimal support)

Self-help (without support or with minimal support, also called unguided self-help) covers a range of psychological interventions typically based on cognitive behavioural principles that

seek to equip people with strategies and techniques to begin to overcome and manage their psychological difficulties. Self-help can include the provision of information in the form of books or other written materials or audio-recordings that include psychoeducation about the problem and describe techniques to overcome it (for instance, cognitive bibliotherapy). Computerised self-administered versions of psychological therapies have also been developed including computerised-CBT [cCBT], computerised behavioural activation, and computerised problem solving therapy. A taxonomy has been identified that distinguishes between self-administered work, in which an individual uses the self-help materials exclusively on his or her own (self-help without support), versus minimal contact in which the individual works through the self-help materials with irregular, often non face-to-face contact with a practitioner whose role is to check on progress and motivate the user (self-help with minimal support), versus self-help with support, see below, in which the individual receives regular and scheduled meetings with a practitioner whose role is to support and guide him or her in using the self-help materials and provide therapeutic feedback.

Self-help with support

Self-help with support (also called guided self-help) is generally accepted as being more efficacious and engaging than simply giving people literature to read. Intervention content may overlap with that used in self-help (without or with minimal) support, for instance, cognitive bibliotherapy and computerised psychological therapies (including computerised-CBT [cCBT], computerised psychodynamic therapy, computerised-problem solving therapy and cognitive bias modification), the difference being the addition of regular scheduled support from a healthcare practitioner for the purposes of supporting and/or facilitating the individual to complete work with the self-administered materials by introducing, monitoring, providing feedback, and reviewing the outcome of such treatment.

Music therapy

Improvisational music therapy has been tested as an intervention for depression, and active music-making has been associated with three dimensions[23] that may be potential

mechanisms of action for improving depressed mood: aesthetic (creating shared experience of meaningfulness and pleasure); physical (playing an instrument is physical and physical activity has recognised benefits for depression); relational (actively participating and communicating musically).

Pharmacological interventions

Pharmacological treatments were grouped according to mechanism of action or chemical structure.

For inclusion in this review, pharmacological interventions needed to be licensed in the UK and in routine clinical use for the first-line treatment of depression. The national prescription data for England in 2017[24] were used to define routine usage of drugs: if a drug appeared in the top 15 antidepressants prescribed by volume it was included, with the exception of dosulepin which the BNF indicates should be initiated by a specialist.

Some interventions were included in the evidence review to improve connectivity within the network meta-analysis but were not considered as part of the decision problem. If necessary for connectivity in the network, excluded pharmacological interventions were added as 'any antidepressant' or 'any SSRI' or 'any TCA' nodes but only where the pharmacological interventions had been compared against an included psychological or physical intervention and/or combined with an included psychological or physical intervention. To improve connectivity, imipramine was also included in the network (because it has been used as a control in many trials) however it was not considered as part of the decision problem.

Physical interventions

Acupuncture

The medical use of acupuncture combines theoretical principles of traditional Chinese medicine, such as re-balancing bodily energy, with knowledge of physiology and anatomy to determine the appropriate site of application. There are several styles of treatment including classical, auricular, trigger point and single point acupuncture. Variations on the traditional insertion of needles include electroacupuncture and laser acupuncture.[25] It has been

suggested that the therapeutic effects of acupuncture may be mediated by its action on limbic brain structures, including the cingulate cortex.[26]

Exercise

The effect of physical activity on mental health has been the subject of research for several decades. There is a growing body of literature examining the effects of physical activity in the treatment of depression. The aerobic forms of physical activity, especially jogging or running, have been most frequently investigated. Physical activity may act as a diversion from negative thoughts and the mastery of a new skill may be important. Social contact may also be an important benefit, and physical activity may have physiological effects such as changes in endorphin and monoamine concentrations.[27,28]

Exercise interventions were classified according to the format (individual or group), the intensity (high intensity described aerobic forms and low intensity described anaerobic forms), and whether the physical activity was supervised or unsupervised.

Yoga

Yoga is a method based on traditional Indian philosophical and spiritual practices with modern yoga forms used in the western world being mostly associated with physical postures, breathing techniques, and meditation. Evidence was only identified for a yoga group for the treatment of depression (and not for individual yoga).

Light therapy

Light therapy (that is, increasing the amount or duration of light exposure) has more commonly been investigated for depression with a seasonal pattern (also known as seasonal affective disorder [SAD]), however, there are some trials that have tested bright light therapy for (nonseasonal) depression. Proposed mechanisms of action are that light may correct disturbed circadian rhythms which have been implicated in the pathophysiology of depression.[29]

Psychosocial interventions

Mindfulness, meditation or relaxation

Mindfulness and meditation approaches were combined into group and individual classes, and progressive muscle relaxation (individual and group) interventions were considered as distinct classes.

Mindfulness or meditation classes included the use of mindfulness meditation as derived from mindfulness-based stress reduction,[30] and mindfulness-based cognitive therapy (MBCT) that was developed with a specific focus on preventing relapse/recurrence of depression.[31-33] These approaches use guided meditative practice, to decentre from negative thoughts and feelings, accept difficulties using a stance of self-compassion and use bodily awareness to ground and transform experience.

Peer support

Support groups provide an opportunity for peer support. Peer support groups are usually facilitated by a healthcare professional with discussions structured around a series of pre-defined topic areas. However, the primary goal of these interventions is to enable mutual support by bringing people with depression into contact with other people who are having similar experiences and providing opportunities for sharing problems and solutions.

Control classes and interventions

Control classes and interventions included: treatment as usual (TAU; that included both TAU and enhanced TAU); any psychotherapy (as defined by study); waitlist; no treatment; placebo (pill placebo); attention placebo; sham acupuncture (traditional non-specific point acupuncture; sham electrostimulation at non-specific points with no current; inactive laser acupuncture). Studies that compared ‘intervention + TAU vs TAU alone’ were recoded as ‘intervention vs no treatment’, assuming an additive effect for TAU (i.e. the addition of TAU to an active arm vs TAU alone was equivalent to the active arm vs no treatment). A sensitivity analysis was carried out to examine the assumption that the effect of TAU was additive versus the assumption that it was multiplicative. The analysis found no meaningful impact on treatment effects, confirming the appropriateness of our approach.

Table 1: Treatment classes and interventions

Treatment class	Interventions
Behavioural therapies individual	Behavioural activation (BA) individual
	Behavioural therapy (Lewinsohn 1976) individual
Behavioural therapies group	Coping with Depression course (group)
	Behavioural activation (BA) group
Cognitive and cognitive behavioural therapies individual	CBT individual (under 15 sessions)
	CBT individual (15 sessions or over)
	Third-wave cognitive therapy individual
	Dialectical behavioural therapy (DBT) individual
Cognitive and cognitive behavioural therapies group	CBT group (under 15 sessions)
	CBT group (15 sessions or over)
	Rational emotive behaviour therapy (REBT) group
	Third-wave cognitive therapy group
	Positive psychotherapy (PPT) group
Problem solving individual	Problem solving individual
Problem solving group	Problem solving group
Counselling individual	Non-directive/supportive/person-centred counselling
Interpersonal psychotherapy (IPT) individual	Interpersonal psychotherapy (IPT) individual
	Interpersonal counselling individual
Short-term psychodynamic psychotherapies individual	Short-term psychodynamic psychotherapy individual
	Dynamic interpersonal therapy (DIT) individual
Short-term psychodynamic psychotherapies group	Short-term psychodynamic psychotherapy group
Long-term psychodynamic psychotherapies individual	Long-term psychodynamic psychotherapy individual
Psychoeducation group	Psychoeducational group programme
Self-help (with no or minimal support)	Cognitive bibliotherapy
	Behavioural bibliotherapy
	Computerised-CBT (CCBT)
	Computerised Coping with Depression course
	Computerised behavioural activation
	Computerised positive psychological intervention
	Computerised attentional bias modification
	Computerised cognitive bias modification
	Computerised mindfulness intervention
	Computerised problem solving therapy
	Computerised expressive writing
	Computerised third-wave cognitive therapy
	Expressive writing
	Psychoeducational website
	Mindfulness meditation CD
Self-help with support	Cognitive bibliotherapy with support
	Behavioural bibliotherapy with support
	Computerised-CBT (CCBT) with support
	Computerised Coping with Depression course with support

	Computerised behavioural activation with support
	Computerised third-wave cognitive therapy with support
	Cognitive bias modification with support
	Computerised problem solving therapy with support
	Expressive writing with support
	Computerised exercise promotion with support
	Mindfulness meditation CD with support
	Relaxation training CD with support
	Third-wave cognitive therapy CD with support
Music therapy group	Music therapy group
Selective serotonin re-uptake inhibitors (SSRIs)	Citalopram
	Escitalopram
	Paroxetine
	Sertraline
	Fluoxetine
	Any SSRI
Tricyclic antidepressants (TCAs)	Amitriptyline
	Clomipramine
	Imipramine
	Lofepramine
	Nortriptyline
	Any TCA
Serotonin and noradrenaline reuptake inhibitors (SNRIs)	Duloxetine
	Venlafaxine
Mirtazapine	Mirtazapine
Trazodone	Trazodone
Any antidepressant (AD)	Any AD
Acupuncture	Traditional acupuncture
	Electroacupuncture
	Laser acupuncture
Exercise individual	Supervised low intensity exercise individual
	Supervised high intensity exercise individual
	Unsupervised low intensity exercise individual
	Unsupervised high intensity exercise individual
Exercise group	Supervised low intensity exercise group
	Supervised high intensity exercise group
Yoga group	Yoga group
Light therapy	Bright light therapy
Mindfulness or meditation individual	Mindfulness-based stress reduction (MBSR) individual
Mindfulness or meditation group	Mindfulness-based cognitive therapy (MBCT) group
	Mindfulness meditation group
	Mindfulness-based stress reduction (MBSR) group
	Meditation-relaxation group
Relaxation individual	Progressive muscle relaxation individual
Relaxation group	Progressive muscle relaxation group
Peer support group	Peer support group

Categorising depressive symptom severity into less and more severe depression

The studies were stratified into less and more severe depression populations according to their depressive symptom severity as indicated by baseline mean scores on validated depression scales. The guideline committee were keen to move away from the traditional categories of mild-to-moderate and moderate-to-severe depression because of the ambiguity of including ‘moderate’ in both severity levels, and the potential advantage of defining a clearer cut-off point that was clinically meaningful. As a first step, for each depression scale used, default (as defined by the developers or widely accepted) cut-off points for different depressive symptom levels (such as mild, moderate or severe) were identified (Table 2).[34-44] Subsequently, the committee used crosswalk tables of standardized depression measurements[45-48] to map across depression scales, using different default cut-off scores of each scale as starting points, for example, a score of 14 and 19 on HAMD, 20 on MADRS and 19 on BDI-I. The committee noted an overall consistency across the depressive symptom severity levels of several rating scales obtained from this mapping exercise as, following crosswalking, a MADRS score of 20 as a threshold point of moderate depression corresponded to a HAMD score of 14 (also indicating moderate depression), a BDI-I score of 19 (which was the cut-off point between mild-to-moderate and moderate-to-severe depression), and a QIDS score of 11 (indicating moderate depression). The committee, therefore, expressed the view that these cut-off scores could be used as provisional anchor points to distinguish between less and more severe depression (see

Table 2). On the other hand, it was noted that the mapping exercise using the default cut-off scores of the above scales (that is, 20 for MADRS, 14 for HAMD, 19 for BDI-I 19 and 11 for QIDS) did not correspond to other rating scales' default cut-off scores (for example, the mapped cut-off score of 25 for BDI-II obtained from the mapping exercise using the above default cut-offs lies towards the middle of the default score range of 20-28 for moderate depression; the mapped cut-off score of 14 for PHQ-9 is just below the 15 threshold score for moderately-severe depression). Moreover, it was noted that more recent validation studies had suggested different thresholds for differentiating across depressive symptom severity levels for some scales; for example, higher thresholds have been suggested and widely implemented in routine practice for HAMD, based on a large study of psychiatric outpatients with major depressive disorder[49] and alternative score ranges have been suggested for BDI-II to differentiate between different symptom levels.[50] Most importantly, the committee placed an emphasis on PHQ-9, because this is the most widely used screening tool for depression in primary care in the UK, but noted potential problems with differentiating moderate (PHQ-9 scores of 10-14) and moderately severe (PHQ-9 scores of 15-19) depression, and had concerns based on their clinical experience and published literature[44,51,52] that the default PHQ-9 cut-off points may overstate depressive symptom severity compared with other validated scales. According to the mapping exercise, the corresponding cross-walked threshold value of PHQ-9 to the other provisional anchor points was 14, which is just one point lower than the 'moderately severe depression', suggesting that PHQ-9 default cut-offs may indeed overstate depressive symptom severity compared with the other 3 depression symptom scales.

Following this mapping exercise, and considering that a) the default severity cut-offs for some scales do not correspond to each other; b) for some scales different cut-offs from the default ones have been suggested based on further validation studies and c) some treatments (for example self-help) have been assessed mostly in milder forms of depression, whereas other treatments (for example antidepressants alone or combined with high

intensity psychological therapies) have been focused on more severe forms of depression, the next step was to explore the clustering of relevant eligible RCTs (identified during scoping searches) around the provisional anchor points reported above, based on the study samples' baseline depression scale scores, to calibrate the anchor points, if needed. It was observed that in several RCTs on self-help for populations with milder forms of depression the baseline mean scores (which were often measured on the PHQ-9) were clustered very close to the provisional cut-off points, whereas treatments for more severe depression were clustered far above the cut-off points defined by the developers.

Based on the above considerations, inspection of RCT baseline depressive symptom score data and their clinical experience, the committee agreed that raising the PHQ-9 threshold score to 16 was a reasonable adjustment to the provisional cut-off scores for differentiating between less and more severe depression. This only slightly moved most of the other scales' provisional anchor points by a score of 1-3, following use of the mapping algorithm. This adjustment allowed a clinically meaningful distinction, appropriate clustering of treatments around cut-off scores (for example, using this threshold most RCTs on self-help moved to the less severe depression category which was considered clinically appropriate and consistent with the study descriptions of the populations), and aligned with treatment decisions in clinical practice, without deviating substantially from the developer-defined thresholds. The final depression scale cut-off points used in this exercise are shown in Table 2.

Table 2: Validated depression symptom scales, default cut-off scores of different symptom severity levels, provisional and final thresholds (“anchor points”), estimated using crosswalk tables of standardized depression measurements[45-48] (a score at an anchor point and above indicates more severe depression, that is, of at least moderate severity)

Depression symptom scale	Default severity cut-off scores	Anchor points	
		Provisional	Final
HAMD (17- 21- and 24-item)	0 to 7: no depression [34]<br>8-13: mild depression<br>14-18: moderate depression<br>19-22: moderately severe depression<br>23+: very severe depression<br><br>Alternative suggested cut-off scores: [49]<br>0 to 7: no depression<br>8-16: mild depression<br>17-23: moderate depression<br>24+: severe depression	14	16
MADRS (10-item)	0 to 6: no depression [35-37]<br>7 to 19: mild depression<br>20 to 34: moderate depression<br>35 to 59: severe depression<br>60+: very severe depression	20	22
PHQ-9	0 to 4: no depression [38]<br>5 to 9: mild depression<br>10 to 14: moderate depression<br>15 to 19: moderately severe depression<br>20 to 27: severe depression	14	16
BDI-I (21-item)	0 to 9: none or minimal depression [39]<br>10 to 18: mild to moderate depression<br>19 to 29: moderate to severe depression<br>30 to 63: severe depression	19	22
BDI-II (21-item)	0 to 13: minimal or no depression [40]<br>14 to 19: mild depression<br>20 to 28: moderate depression<br>29 to 63: severe depression<br><br>Alternative suggested cut-off scores: [50]<br>0 to 10: no depression<br>11 to 16: mild mood disturbance<br>17 to 20: borderline clinical depression<br>21 to 30: moderate depression<br>31 to 40: severe depression<br>40+: extreme depression	25	30
CES-D (20-item)	≥16: caseness [41]	31	36
QIDS (16-item)	0 to 5: no depression [42]<br>6 to 10: mild depression<br>11 to 15: moderate depression<br>16 to 20: severe depression<br>21 to 27: very severe depression	11	12

HADS-D (7-item)	<8: no depression [43,44]<br>8 to 10: doubtful / mild depression<br>11 to 15: definite / moderate depression<br>16+: severe depression	10	12

Methods of the statistical analysis and codes for data synthesis

Methods of the statistical analysis

NMAs were conducted within a Bayesian framework using Markov Chain Monte Carlo simulation techniques implemented in OpenBUGS 3.1.2, which is a variant of WinBUGS 1.4.3.[53-55] Three different sets of initial values were used when running each model; convergence was assessed by visually inspecting the mixing of the two chains in the history plots and the Brooks-Gelman-Rubin diagram and satisfactory by 80,000 simulations for all outcomes.[56,57] A further simulation sample of at least 20,000 iterations post-convergence was obtained on which all reported results were based.

In the situation where a study compared two treatments that were coded the same way (based on the review protocol), these were included as separate arms. Any differences between the treatments in these arms therefore contributed to between-study variation.

For binary data, studies with zero or 100% events in all arms were excluded from the analysis because these studies provide no evidence on relative effects.[58]. For studies with zero or 100% events in one arm only, we planned to analyse the data without continuity corrections where computationally possible. Where this was not possible, we used a continuity correction where we added 0.5 to both the number of events and the number of non-events, which has shown to perform well when there is an approximate 1:1 randomisation ratio across intervention arms.[59] For the small number of studies in which there was not an approximate 1:1 randomisation ratio, a continuity correction that was weighted by the reciprocal of the opposite group arm size was used.[59] For studies with >2 arms we extended this weighted continuity correction by using a weighting that was a sum of the sample size in the other treatment arms in the study, and then standardised the weights so that they summed to 1.

Relative intervention effects are reported as posterior median log-odds ratios (log-OR) or standardised mean differences (SMD) and 95% Credible Intervals (CrIs). Posterior mean

ranks and 95% CrIs are also reported. Only interventions and classes of interest were included in the calculations of the rankings. The interventions that were included in the NMA in order to provide links to the networks but were deemed not part of the decision problem and were therefore excluded from the rankings were:

- No treatment
- Any psychotherapy
- CBT individual (15 sessions or over) + pill placebo
- CBT individual (under 15 sessions) + pill placebo
- Interpersonal psychotherapy individual + pill placebo
- Non-directive/supportive/person-centred counselling + pill placebo
- Computerised-CBT + TAU
- Progressive muscle relaxation individual + pill placebo
- Any SSRI
- Any TCA
- Imipramine
- Any AD

The classes that were included in the NMA in order to provide links to the networks but were not of interest for decision-making and were therefore excluded from the rankings were

- No treatment
- Any psychotherapy
- Cognitive and cognitive behavioural therapies individual + placebo
- Interpersonal psychotherapy individual + placebo
- Counselling individual + placebo
- Self-help + TAU
- Relaxation individual + placebo
- Any AD

Class effect models

Classes are groups of interventions which are thought to have similar effects. Class models[60] were used so that strength could be borrowed across treatments in the same class and to reconnect disconnected networks. For all outcomes, random class effect models were used which assume that the effects of treatments in a class are distributed around a common class mean, m_{class}, with a within-class variance, τ^2_{class}. In this way treatment effects are shrunk towards a class mean and can borrow strength from other elements of the class, whilst still estimating distinct effects for each treatment.

The pooled relative treatment effects were assumed to be exchangeable within class:

$$d_{1,k} \sim N\left(m_{D_k}, \tau^2{}_{D_k}\right)$$

where $d_{1,k}$ is the effect of intervention k relative to intervention 1, and D_k indicates the class to which treatment *k* belongs.

For treatments belonging to a class with only one or two treatments in a particular analysis there is insufficient evidence to estimate the within-class variance, however we would still expect there to be heterogeneity between the within class treatment effects. For this reason, the within-class variance was shared with another similar class in the model, where the variability between treatment effects might be expected to be similar. The following rules applied where there was limited information with which to estimate separate class variances (e.g. where classes had only one or two treatments) but variance could be shared with another class for which it could be more reliably estimated. The following variance sharing rules were used when necessary:

- The following classes shared variance with Behavioural therapies individual:
    - Cognitive and cognitive behavioural therapies individual
- The following classes shared variance with Cognitive and cognitive behavioural therapies individual:

- Behavioural therapies individual
- Behavioural therapies group
- Cognitive and cognitive behavioural therapies group
- Problem solving individual
- Problem solving group
- Counselling individual
- Interpersonal psychotherapy (IPT) individual
- Psychoeducation group
- Self-help
- Self-help with support
- Long-term psychodynamic psychotherapies individual
- Short-term psychodynamic psychotherapies individual
- Short-term psychodynamic psychotherapies group
- Mindfulness or meditation individual
- Relaxation individual
- Cognitive and cognitive behavioural therapies individual + placebo
- Interpersonal psychotherapy (IPT) individual + placebo
- Counselling individual + placebo
- Relaxation individual + placebo
- Acupuncture
- Cognitive and cognitive behavioural therapies individual + AD
- Acupuncture + counselling individual

* The following classes shared variance with Cognitive and cognitive behavioural therapies group:
  - Music therapy group

- Mindfulness or meditation group
- Relaxation group
- Peer support group
- Yoga group

- The following classes shared variance with Self-help with support:
  - Exercise individual
  - Exercise group
- The following classes shared variance with SSRIs:
  - TCAs
  - SNRIs
- The following classes shared variance with Acupuncture:
  - Sham acupuncture
  - Light therapy
  - Acupuncture + AD
  - Sham acupuncture + AD
  - Light therapy + AD
- The following classes shared variance with Cognitive and cognitive behavioural therapies individual + AD:
  - Self-help + TAU
  - Behavioural therapies individual + AD
  - Cognitive and cognitive behavioural therapies group + AD
  - Problem solving individual + AD
  - Long-term psychodynamic psychotherapy individual + AD
  - Interpersonal psychotherapy (IPT) individual + AD
  - Counselling individual + AD

- Self-help + AD
  - Short-term psychodynamic psychotherapies individual + AD
  - Psychoeducation group + AD
  - Peer support group + AD
  - Mindfulness or meditation group + AD
  - Relaxation individual + AD
  - Exercise individual + AD
  - Exercise group + AD
  - Yoga group + AD
  - Cognitive and cognitive behavioural therapies individual + exercise group
  - Cognitive and cognitive behavioural therapies group + exercise group
- The following class used the maximum of either the SSRI class variance or the TCA class variance:
  - Any AD
- The following class used the maximum of either the Cognitive and cognitive behavioural therapies individual class variance or the Cognitive and cognitive behavioural therapies group class variance:
  - Any psychotherapy

The following treatments were not allocated to a class, and a single intervention effect estimated (equivalent to a class-effect model with within-class variability ($\tau^2_{D_k} = 0$)):

- Pill placebo
- Attention placebo
- No treatment
- Waitlist
- TAU

- Enhanced TAU
- Mirtazapine
- Trazodone

These assumptions were based on the committee's expert opinion.

If class variances could not be estimated for any psychological/physical/combined therapies (i.e. the absence of class variance information on both Behavioural therapies individual *and* Cognitive and cognitive behavioural therapies individual), then the class variance was shared with the class that had the maximum class variance.

The within-class mean treatment effects were given vague priors m_{class} ~ N(0,100^2) and the within-class standard deviations (SD) were given vague uniform priors τ_{class} ~ Uniform(0,5). In cases where there was evidence that the prior constrained the posterior, the upper limit was extended to 7.

For treatments connected only by a single, small study with zero responders in one of the connecting arms, this sometimes led to convergence issues, even after making a continuity correction, which could not be resolved without making additional strong assumptions. In these cases, the treatments were effectively disconnected from the network, meaning that relative effects for them compared to other treatments in the network could not be estimated, and thus are not presented in the results.

Treatment effects have been reported at both the class and intervention level.

Selection of reference treatments for presentation of results

For the purpose of performing the NMA analysis, the network reference treatment for each outcome was selected based on its connectivity and the number and size of studies that included it in order to improve computation and model convergence. However, the basic treatment effect parameters estimated from this model can be used to estimate treatment effects versus more clinically relevant reference treatments, as desired.

The selection of reference treatments for presentation of results for less and more severe depression was therefore also based on their clinical relevance, in addition to ensuring there would be sufficient precision when using them for estimating relative effects (based on the size of the evidence of control treatments and their connectivity in the networks created for each population). Control treatments with their own established effects were prioritised. The committee expressed a preference for pill placebo as it is well-defined across trials. On the other hand, the definition of TAU may vary across trials, although it has been widely used as the control treatment in meta-analyses of psychological trials. The committee considered the comparisons of psychological treatment classes and interventions with pill placebo as an advantage of conducting the NMAs, because psychological therapies are not routinely compared with pill placebo, unless active drug arms are included in the trial. A further advantage of selecting pill placebo is that it provides a more conservative estimate and convincing comparison for clinical effect and addresses treatment expectancy effects for interventions. Nevertheless, pill placebo was tested on a very small number of people in less severe depression and it had limited connectivity (or was completely absent) in most network plots in this population. Therefore, its use as a reference for this population was considered inappropriate and TAU was selected instead as the next best option to serve as reference in NMAs of treatments for less severe depression. The only exception was for the outcome of treatment discontinuation due to side effects from medication in those who discontinued treatment in less severe depression, where pill placebo was used as the relevant (and only available) reference treatment.

Model assumptions

When considering models for NMA, there are several aspects of the data that will impact the choice of parameters included in the model. Two important assumptions must be made in NMA regarding heterogeneity and consistency. Heterogeneity concerns the differences in treatment effects between trials within each treatment contrast, while consistency concerns the differences between the direct and indirect evidence informing the treatment contrasts.[60]

A further assumption concerned the within-class variability, where the treatment effects within a class may be assumed to be identical or exchangeable.

Two types of class effect models were considered for the base-case analyses, all of which assumed random study-specific treatment effects and consistency:

1) **Random study-specific treatment, fixed class effects** model. Treatments within classes are assumed to have identical effects, but any beyond chance differences between trial-specific estimates of the same treatment contrasts are captured by the between-study standard deviation (SD).
2) **Random study-specific treatment, random class effects** model. Treatments within classes are assumed to have exchangeable effects and any beyond chance differences between trial-specific estimates of the same treatment contrasts are captured by the between-study SD.

When assessing class effect NMA models, it is good practice to assess and compare the fit of both fixed and random class effects models, as differences may provide evidence of potential within-class variability in treatment effects. The posterior median between-study standard deviation measures the heterogeneity of treatment effects estimated by trials within contrasts, and the posterior median within-class standard deviation measures the variability of treatment effects within a class. When fitting random effects models, it is important to assess whether there is enough evidence informing the between-study/within-class standard deviation by comparing the prior and posterior distributions for these parameters. Note that in the absence of sufficient treatments within a class to reliably estimate within-class standard deviation, within-class standard deviation was shared between classes following variance-sharing rules agreed by the committee (see Class effect models).

The posterior mean of the residual deviance, which measures the magnitude of the differences between the observed data and the model predictions of the data, was used to assess the goodness of fit of each model.[61,62] Smaller values are preferred, and in a well-

fitting model the posterior mean residual deviance should be close to the number of data points in the network (each study arm contributes 1 data point).[58,61]

In addition to comparing how well the models fit the data using the posterior mean of the residual deviance, models were compared using the deviance information criterion (DIC). This is equal to the sum of the posterior mean deviance and the effective number of parameters, and thus penalizes model fit with model complexity.[61] Lower values are preferred and typically differences of at least 3 points are considered meaningful.[61]

SMD analysis: methods

We wished to include as many trials and information as possible in each analysis even when data were reported in different ways. This meant transforming the data in some cases. For the SMD analysis we wanted to conduct a NMA on the mean difference in change from baseline (CFB) (for which standard methods are available, see [58]). The data required for each arm of each study are the mean CFB, the standard deviation in CFB and the total number of individuals in that arm (or the standard error of the mean change from baseline).

However, some studies did not report these data, and instead reported

a) the baseline and endpoint means, standard deviations and number of individuals, for each arm of the study;

b) the number of individuals responding to treatment in each arm of each study, out of the total number of individuals, defined as those improving by more than a certain percentage from baseline;

Studies reporting outcomes a) or b) above also provide information on the mean change from baseline, through the relationship between the underlying continuous scale and the measurements that can be derived from it.

For our analysis, if CFB data were available in a study we used those data. If a study did not report CFB but reported baseline and endpoint data then these were included in the model (see OpenBUGS codes - SMD analysis) with an additional step to transform them to CFB

using a correlation coefficient (“rho”) of 0.31 estimated from the data. If a study reported neither CFB nor baseline and endpoint data but did report response, we used the response data and transformed it to CFB. For using intention-to-treat data we required that the number of participants randomised be reported, whilst for per-protocol data we required that the number of completers be reported. If these were not reported consistently for continuous data on CFB, baseline or endpoint, then we preferred to use the number of individuals responding to treatment and derive the continuous results from this.

Analysis was carried out on all patients randomized where possible. However, if trials only reported the number of completers then these were also included.

Notation

To transform the data we assumed that n_{ik} individuals are randomised to each arm k (k>1) of study i=1,…,M, on which the following outcomes are recorded for individual j=1,…,n_{ik}:

x_{jik} - the score at baseline for individual j in arm k of trial i, on a given continuous scale;

y_{jik} - the score at follow-up for individual j in arm k of trial i, on a given continuous scale;

c_{jik} - the change from baseline for individual j in arm k of trial i, on a given continuous scale, where $c_{jik} = y_{jik} - x_{jik}$;

R_{jik} - response status at endpoint for individual j in arm k of trial i, defined as **at least a *q_i*100*% reduction** of the endpoint measurement on a given continuous scale, compared to baseline, i.e.

$$R_{jik} = \begin{cases} 1 & \text{if } y_{jik} - x_{jik} \le -q_i x_{jik} \\ 0 & \text{otherwise} \end{cases} \quad (1)$$

Note that different studies may have used a different cut-off *q* (although they would be expected to be the same for all arms of a study), and these are therefore indexed by study.

Reported outcomes

Studies may report all or some of the following observed outcomes

$m_{X,ik}$ - the observed mean at baseline in arm *k* of trial *i*, on a given continuous scale;

$sd_{X,ik}$ - the observed standard deviation at baseline in arm *k* of trial *i*, on a given continuous scale;

$m_{Y,ik}$ - the observed mean at endpoint in arm *k* of trial *i*, on a given continuous scale;

$sd_{Y,ik}$ - the observed standard deviation at endpoint in arm *k* of trial *i*, on a given continuous scale;

$m_{C,ik}$ - the observed mean change from baseline in arm *k* of trial *i*, on a given continuous scale;

$sd_{C,ik}$ - the observed standard deviation in change from baseline in arm *k* of trial *i*, on a given continuous scale;

ρ_{ik} - the observed correlation between baseline and endpoint scores measured on the same individual in arm *k* of trial *i*. (Although this is rarely reported directly, it can be calculated when the means and standard deviations at baseline, endpoint and from the CFB are provided);

$r_{resp,ik}$ - the number of individuals achieving response in arm *k* of trial *i*, with response defined in equation (1).

Relationship between different outcomes

We assume that for each patient the baseline and endpoint measurements are sampled from a bivariate Normal distribution. Thus for all patients in arm *k* of trial *i*, we assume that their baseline, X_{ik}, and endpoint measurements Y_{ik}, are independent and identically distributed as

$$\begin{pmatrix} X_{ik} \\ Y_{ik} \end{pmatrix} \sim \mathrm{N}_2\left(\begin{pmatrix} \mu_{X,ik} \\ \mu_{Y,ik} \end{pmatrix}, \begin{pmatrix} \sigma^2_{X,ik} & \rho_{ik}\sigma_{X,ik}\sigma_{Y,ik} \\ \rho_{ik}\sigma_{X,ik}\sigma_{Y,ik} & \sigma^2_{Y,ik} \end{pmatrix} \right) \quad (2)$$

with $\mu_{X,ik}$ and $\mu_{Y,ik}$ representing the means and $\sigma^2_{X,ik}$ and $\sigma^2_{Y,ik}$ the variances at baseline and endpoint for individuals in arm *k* of trial *i*, respectively, and ρ_{ik} being the within arm and study correlation between baseline and endpoint measurements on the same individuals.

We define the mean change from baseline in arm *k* of trial *i* as $\theta_{ik} = \mu_{Y,ik} - \mu_{X,ik}$ as the parameter of interest.

NMA model for continuous outcomes

With continuous outcome data, meta-analysis is usually based on the sample means, with standard errors assumed known. Here we are interested in modelling the mean changes from baseline, which are assumed to be approximately normally distributed, with likelihood

$$m_{C,ik} \sim N\left(\theta_{ik}, se^2_{C,ik}\right)$$

The parameter of interest is the mean, θ_{ik}, of this distribution. For a random effects model we write

$$\theta_{ik} = \gamma_i + \delta_{ik} \quad (3)$$

where γ_i are the trial-specific effects of the treatment in arm 1 of trial *i*, treated as unrelated nuisance parameters, and the δ_{ik} are the trial-specific treatment effects of the treatment in arm *k* relative to the treatment in arm 1 in that trial, where $\delta_{i1} = 0$. The trial-specific random effects δ_{ik}, represent the mean differences between the change from baseline for the treatment in arm *k* and the treatment in arm 1 of trial *i* and, in a random effects model,

$$\delta_{ik} \sim \mathrm{Normal}(d_{t_{i1},t_{ik}}, \tau^2_{study}) \tag{4}$$

where τ^2_{study} denotes the between-study heterogeneity, assumed common to all treatment comparisons and $d_{t_{i1}t_{ik}} = d_{1,t_{ik}} - d_{1,t_{i1}}$ are the pooled mean differences, defined by the consistency equations ($d_{11} = 0$). The fixed effect model is obtained by replacing equation (3) with $\theta_{ik} = \gamma_i + d_{1,t_{ik}} - d_{1,t_{i1}}$. Where studies with more than 2 arms are present, a correlation is induced in the trial specific effects δ_{ik} so equation (4) is replaced by a multivariate normal distribution with correlation equal to 0.5 (Dias 2011; Higgins 1996).

Likelihood and link functions for studies reporting other outcomes

- Studies reporting mean and variance at endpoint

From the joint bivariate normal distribution in equation (2) we know that

$$\left(Y_{ik} - X_{ik}\right) \sim N\left(\theta_{ik}, \sigma^2_{X,ik} + \sigma^2_{Y,ik} - 2\rho_{ik}\sigma_{X,ik}\sigma_{Y,ik}\right) \tag{5}$$

Therefore, studies not reporting change from baseline but reporting the mean and variance at baseline and endpoint also provide information on the parameter of interest θ_{ik}, the mean change from baseline.

For these studies we can calculate the mean change from baseline as $m_{C,ik} = m_{Y,ik} - m_{X,ik}$.

Using equation (5), the likelihood can be written as

$$m_{C,ik} \sim N\left(\theta_{ik}, se^2_{X,ik} + se^2_{Y,ik} - 2\rho_{ik} se_{X,ik} se_{Y,ik}\right)$$

Provided the standard errors at baseline and endpoint can be obtained and that we have information on the within-study correlation, the remaining model is given in equations (3) and (4) can be used to pool the mean differences in change from baseline.

- Studies reporting number of responders

Using equation (1), the probability of response for individuals in arm *k* of trial *i* is defined as

$$R_{ik} = \Pr(Y_{ik} - X_{ik} \leq -qX_{ik}) \qquad (6)$$

Conditioning on the baseline value X_{ik} we have

$$Y_{ik} \mid X_{ik} \sim N\left(\mu_{X,ik}(1-\rho_{ik}) + \theta_{ik} + \rho_{ik} X_{jik}, (1-\rho^2_{ik})\sigma^2_{X,ik}\right) \qquad (7)$$

thus,

$$\begin{aligned} R_{ik} \mid X_{ik} &= \Pr_{Y|X}\left(Y_{ik} < (1-q)X_{ik}\right) \\ &= \Phi\left(aX_{ik} + b\right) \end{aligned} \qquad (8)$$

with

$$a = \frac{1-q-\rho_{ik}}{\sigma_{X,ik}\sqrt{1-\rho^2_{ik}}} \ , \ b = -\frac{\mu_{X,ik}(1-\rho_{ik}) + \theta_{ik}}{\sigma_{X,ik}\sqrt{1-\rho^2_{ik}}}$$

Therefore the unconditional probability of response in arm *k* of trial *i* is

$$R_{ik} = E_{X_{ik}}\left[\Phi\left(aX_{ik} + b\right)\right] \qquad (9)$$

It can be shown that

$$E_X\left[\Phi\left(aX+b\right)\right]=\Phi\left(\frac{aE(X)+b}{\sqrt{1+a^2Var(X)}}\right) \tag{10}$$

thus the probability of response for individuals in arm *k* of trial *i* can be written as

$$R_{ik}=\Phi\left(\frac{-(q\mu_{X,ik}+\theta_{ik})}{\sigma_{X,ik}\sqrt{1+(1-q)(1-q-2\rho_{ik})}}\right) \tag{11}$$

Therefore, studies not reporting the change from baseline or endpoint measures, but providing information on the probability of response, also provide information on the parameter of interest, the mean change from baseline θ_{ik}.

These studies have a binomial likelihood

$$r_{resp,ik} \sim \text{Binomial}(R_{ik}, n_{ik})$$

Provided the baseline mean and standard deviation for each study are reported and that we also have information on the correlation between baseline and endpoint scores in each arm of each study, we can replace these as if they are known into equation (11) and then use equations (3) and (4), as before.

Prior distributions and computation

In this case non-informative prior distributions are chosen for the pooled treatment effects, relative to treatment 1, d_{1k}, k=2,…,nt , where nt is the number of treatments in the network

$$d_{1k} \sim \text{Normal}(0,100^2) \tag{12}$$

and a Uniform prior between 0 and 5 is chosen for the between-study heterogeneity, which is thought to be sufficiently wide to capture the variability in difference in mean change from baseline across trials making the same comparisons.

Prior distributions for the within-class standard deviation are given as detailed under ‘Class effect models’.

Analysis on the SMD scale

In this case, studies also used different underlying continuous scales on which they report the means or the number of responders. As the methods noted above are study and arm specific, they apply regardless of which scale was used in that trial, although care needs to be taken to ensure that the pre-specified cut-offs *q* and *h* are appropriate for the scale used in a particular study.

Pooling of the difference in means across different scales is not appropriate. A common approach is to use the SMD, where the mean difference is divided by a standardising constant, which can be the population standard deviation for each scale (if known), or its estimate, s_i. We use the baseline SD as the standardising constant because it is not influenced by treatment, so better reflects the SD of the outcome scale in the RCT population.[63]

The standardising constant can be adjusted in different ways (Cooper 2009). We use Cohen’s d,[64] but the analysis using another standardising constant can be done following the same principles.

The SMD for arm *k* of study *i* compared to arm 1 of study i, λ_{ik} , is given as

$$\lambda_{ik} = \frac{m_{ik} - m_{i1}}{s_i} \qquad (13)$$

where s_i in a two arm study is given as

$$s_i = \sqrt{\frac{(n_{i1} - 1)sd^2_{i1} + (n_{i2} - 1)sd^2_{i2}}{n_{i1} + n_{i2} - 2}} \qquad (14)$$

and in a three arm study is given as

$$s_i = \sqrt{\frac{(n_{i1}-1)sd^2{}_{i1} + (n_{i2}-1)sd^2{}_{i2} + (n_{i3}-1)sd^2{}_{i3}}{n_{i1}+n_{i2}+n_{i3}-3}} \quad (15)$$

The likelihood for each study reporting the various outcomes are as before, but the parameter of interest is now the SMD λ_{ik} . Thus the model is defined as

$$\lambda_{ik} = \gamma_i + \delta_{ik} \quad (16)$$

This model is linked to the mean change from baseline through the following relationship

$$\theta_{ik} = \lambda_{ik} s_i \quad (17)$$

Prior distributions can be defined as before.

Response analysis: methods

The response outcomes (both response in those randomised and response in treatment completers) on each treatment were informed both by studies reporting response and studies reporting continuous measures. Three types of outcomes were used to inform response outcomes, prioritised in the following order:

a) Number of individuals responding to treatment in each arm of each study, defined as those improving by more than a certain percentage from baseline, out of the total number of individuals randomised (or out of the total number of individuals who completed treatment, as relevant).

b) The mean CFB, the standard deviation in CFB and the total number of individuals in that arm (or the standard error of the mean change from baseline);

c) The baseline and endpoint means, standard deviations and number of individuals, for each arm of the study.

Studies reporting outcomes b) or c) above also provide information on the probability of response through the relationship between the underlying continuous scale and the measurements that can be derived from it.

For this analysis, if response data were available in a study we used those data. If that study did not report response but reported CFB we used the CFB data and transformed these to response. If a study reported neither response nor CFB but did report baseline and endpoint data, we used the baseline and endpoint data and transformed these to response.

Continuous SMD data were converted to LOR following the approach recommended by the Cochrane collaboration.[65] For trials reporting response the following model was used:

$$r_{ik} \sim \text{Binomial}(p_{ik}, n_{ik})$$

where r_{jk} is the number of individuals achieving response in arm k of trial j, n_{jk} is the total number of individuals in arm k of trial j, and p_{jk} is the probability of response in arm k of trial j. These probabilities are modelled on the log-odds scale as:

$$\text{logit}(p_{ik}) = \alpha_i + \eta_{ik}$$

where η_{ik} represents the relative treatment effect of the treatment in arm k compared with the treatment in arm 1 in trial i, on the log-odds ratio (LOR) scale and $\eta_{i1} = 0$. Thus $\eta_{ik} > 0$ favours the treatment in arm k and $\eta_{ik} < 0$ favours the treatment in arm 1.

The LOR of response can be related to a notional SMD for response using the formula:[66]

$$LOR_{\text{Response}} = \frac{\pi}{\sqrt{3}} SMD_{\text{Response}} \qquad (18)$$

noting the change in sign to retain the interpretation of a positive LOR favouring treatment k.

The LOR was obtained by transforming the treatment effect from the SMD scale using equation (18). So, the treatment effect on response is informed by the treatment effect in studies on the pooled scale of symptoms as:

$$\eta_{ik} = \left(-\frac{\pi}{\sqrt{3}} \delta_{ik} \right)$$

Standard NMA random and fixed effects model can used to pool η, as described in previous section [‘*SMD analysis: methods’* under subsection ‘*NMA model for continuous outcomes*’]. Prior distributions can also be defined as before.

Information on within-study correlation and standard deviation at follow-up

To apply the methods described in sub-sections of ‘*Likelihood and link functions for studies reporting other outcomes*’ within section ‘*SMD analysis: methods*’ we needed information on a) the correlation between baseline and endpoint scores and b) the relationship between standard deviations (SDs) at baseline and endpoint.

For a) we identified 35 studies in our dataset that provided information on mean and SD at baseline, mean and SD at endpoint and the mean and SD of change from baseline. The correlations had a median of 0.31 (Inter-Quartile Range: 0.18-0.47), and this value was used for subsequent calculations.

For b) we plotted the SDs at baseline and endpoint from every study that reported both by group of intervention and population (Figure 1 and Figure 2). The blue line on these plots is the regression line with 95% confidence interval and the red line is the line of equality where y=x. The regression equation is also shown. We used the regression equation to predict SD at endpoint from SD at baseline in studies where SD at endpoint was not reported using the regression equations given.

Figure 1. Plot of SDs at baseline and endpoint – Less severe depression.

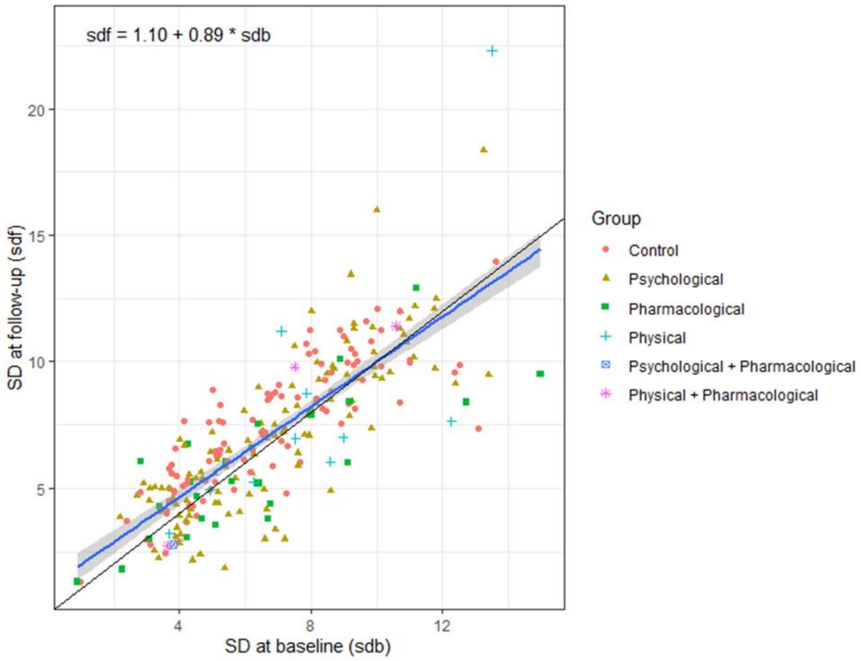

Figure 2. Plot of SDs at baseline and endpoint – More severe depression.

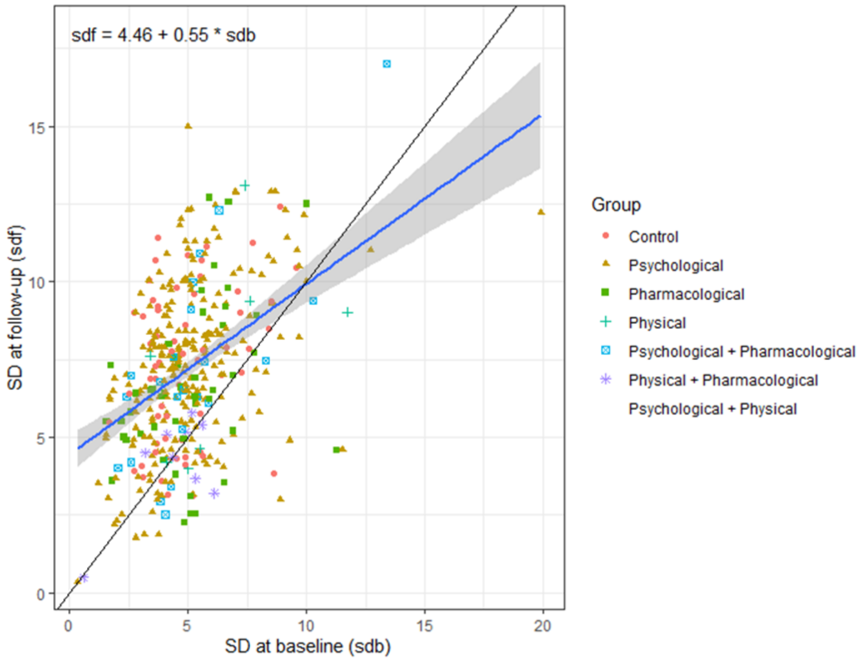

Discontinuation and remission analyses

These analyses were conducted using a binomial likelihood and logit link model using relevant published NMA code.[58] For 'treatment discontinuation for any reason' and 'remission in those randomised' the denominator was the total number of patients randomized. For 'treatment discontinuation due to side effects from medication, in those who discontinued treatment' the denominator was the total number of patients who discontinued treatment. For 'remission in completers' the denominator was the total number of patients who completed treatment.

OpenBUGS codes

Sample OpenBUGS codes for the SMD and the response outcomes are provided below.

Additional codes used in the NMA are available upon request.

Sample OpenBUGS code – SMD analysis

```
# Normal likelihood, identity link: SMD with arm-based means
# Random effects model for multi-arm trials
model{                                      # *** PROGRAM STARTS
for(i in 1:ns){                             #   LOOP THROUGH STUDIES
  w[i,1] <- 0   # adjustment for multi-arm trials is zero for control arm
  delta[i,1] <- 0                           # trt effect zero for control arm
  mu[i] ~ dnorm(0,.0001)                    # vague priors for all trial
baselines
 }
# (1) CFB DATA
for(i in 1:nsCFB){
  # calculate pooled.sd and adjustment for SMD
  df[i] <- sum(nCFB[i,1:naCFB[i]]) - naCFB[i] # denominator for
pooled.var
  Pooled.var[i] <- sum(nvar[i,1:naCFB[i]])/df[i]
  # pooled sd for study i, for SMD
  Pooled.sd[i] <- sqrt(Pooled.var[i])
  H[i] <- 1                                 # use Cohen's d (ie no adjustment)
  for (k in 1:naCFB[i]){
    se[i,k] <- sdCFB[i,k]/sqrt(nCFB[i,k])
    var[i,k] <- pow(se[i,k],2)              # calcultate variances
    prec[i,k] <- 1/var[i,k]                 # set precisions
    yCFB[i,k] ~ dnorm(phi[i,k], prec[i,k]) # normal likelihood
    phi[i,k] <- theta[i,k] * (Pooled.sd[i]/H[i]) # theta is stand mean
    theta[i,k] <- mu[i] + delta[i,k] # model for linear pred, delta is
SMD
    dev[i,k] <- (yCFB[i,k]-phi[i,k])*(yCFB[i,k]-phi[i,k])*prec[i,k]
    nvar[i,k] <- (nCFB[i,k]-1) * pow(sdCFB[i,k],2) # for pooled.sd
   }
  # summed residual deviance contribution for this trial
  resdev[i] <- sum(dev[i,1:naCFB[i]])
 }
# (2) BASELINE + FOLLOW-UP DATA (no CFB)
for(i in 1:nsBF){                            #   LOOP THROUGH STUDIES
```

```
  # calculate pooled.sd and adjustment for SMD
  df[i+nsCFB] <- sum(n[i,1:naBF[i]]) - naBF[i] # denominator for
pooled.var
  Pooled.var[i+nsCFB] <- sum(nvarBF[i,1:naBF[i]])/df[i+nsCFB]
  # pooled sd for study i, for SMD
  Pooled.sd[i+nsCFB] <- sqrt(Pooled.var[i+nsCFB])
  H[i+nsCFB] <- 1                    # use Cohen's d (ie no adjustment)
  for (k in 1:naBF[i]){
    yBF[i,k] <- yF[i,k] - yB[i,k]      # calculate mean CFB
    seF[i,k] <- sdF[i,k]/sqrt(n[i,k]) # se at followup
    seB[i,k] <- sdB[i,k]/sqrt(n[i,k]) # se at baseline
    # variance of mean CFB, assuming correlation rho
    var[i+nsCFB,k] <- pow(seF[i,k],2)+ pow(seB[i,k],2)
-2*(seF[i,k]*seB[i,k]*rho)
    prec[i+nsCFB,k] <- 1/var[i+nsCFB,k] # set CFB precisions
    yBF[i,k] ~ dnorm(phi[i+nsCFB,k], prec[i+nsCFB,k]) # normal likelihood
    # theta is standardised mean
    phi[i+nsCFB,k] <- theta[i+nsCFB,k] * (Pooled.sd[i+nsCFB]/H[i+nsCFB])
    # model for linear predictor, delta is SMD
    theta[i+nsCFB,k] <- mu[i+nsCFB] + delta[i+nsCFB,k]
    # residual deviance contribution
    dev[i+nsCFB,k] <- (yBF[i,k]-phi[i+nsCFB,k]) * (yBF[i,k]-
phi[i+nsCFB,k])                      * prec[i+nsCFB,k]
    # Variance of baseline for pooled SD
    varBF[i,k] <- pow(sdB[i,k],2)
    nvarBF[i,k] <- (n[i,k]-1) * varBF[i,k] # for pooled.sd
   }
  # summed residual deviance contribution for this trial
  resdev[i+nsCFB] <- sum(dev[i+nsCFB,1:naBF[i]])
 }
# (3) RESPONSE DATA (no CFB or BL+follow-up)
for(i in 1:nsR){                      #   LOOP THROUGH STUDIES
  # calculate pooled.sd and adjustment for SMD
  df[i+nsCFB+nsBF] <- sum(nR[i,1:naR[i]]) - naR[i] # denominator for
pooled.var
  Pooled.var[i+nsCFB+nsBF] <- sum(nvarR[i,1:naR[i]])/df[i+nsCFB+nsBF]
  # pooled sd for study i, for SMD
  Pooled.sd[i+nsCFB+nsBF] <- sqrt(Pooled.var[i+nsCFB+nsBF])
  H[i+nsCFB+nsBF] <- 1              # use Cohen's d (ie no adjustment)
  for (k in 1:naR[i]){
    r[i,k] ~ dbin(R[i,k], nR[i,k])    # binomial likelihood
    R[i,k] <- phi.adj[i,k]
    x[i,k] <- -(q[i]*yBR[i,k]+ phi[i+nsCFB+nsBF,k])/(sdBR[i,k] *
sqrt(1+(1-q[i])*(1-q[i]-2*rho)))
    # adjust link function phi(x) for extreme values that can give
numerical
    # errors when x< -5, phi(x)=0, when x> 5, phi(x)=1
    phi.adj[i,k] <- (step(5+x[i,k]) * step(x[i,k]-5)
           + step(5-x[i,k])* step(x[i,k]+5) * phi(x[i,k]))*(1-
equals(x[i,k],5))
           + equals(x[i,k],5)    # correct for x=5
    # theta is standardised mean
    phi[i+nsCFB+nsBF,k] <- theta[i+nsCFB+nsBF,k]
                 * (Pooled.sd[i+nsCFB+nsBF]/H[i+nsCFB+nsBF])
    # model for linear predictor, delta is SMD
    theta[i+nsCFB+nsBF,k] <- mu[i+nsCFB+nsBF] + delta[i+nsCFB+nsBF,k]
    # residual deviance contribution
    rhat[i,k] <- R[i,k] * nR[i,k]
    dev[i+nsCFB+nsBF,k] <- 2 * (r[i,k] * (log(r[i,k])-log(rhat[i,k]))
         + (nR[i,k]-r[i,k]) * (log(nR[i,k]-r[i,k]) - log(nR[i,k]-
rhat[i,k])))
```

```
    # sd for follow-up from baseline using estimated regression model
parameters
    # current values for more severe depression
    sdR[i,k] <-  4.46 + (sdBR[i,k] * 0.55)
    # Variance of baseline for pooled SD
    nvarR[i,k] <- (nR[i,k]-1) * pow(sdBR[i,k],2) # for pooled.sd
   }
  # summed residual deviance contribution for this trial
  resdev[i+nsCFB+nsBF] <- sum(dev[i+nsCFB+nsBF,1:naR[i]])
 }
#
# RE MODEL (CFB data)
for(i in 1:nsCFB){                     # LOOP THROUGH STUDIES WITH CFB
DATA
  for (k in 2:naCFB[i]){               # LOOP THROUGH ARMS
    # trial-specific RE distributions
    delta[i,k] ~ dnorm(md[i,k], taud[i,k])
    md[i,k] <- d[tCFB[i,k]] - d[tCFB[i,1]] + sw[i,k]
    # precision of RE distributions (with multi-arm trial correction)
    taud[i,k] <- tau *2*(k-1)/k
    #adjustment, multi-arm RCTs
    w[i,k] <- delta[i,k] - d[tCFB[i,k]] + d[tCFB[i,1]]
    # cumulative adjustment for multi-arm trials
    sw[i,k] <-sum(w[i,1:k-1])/(k-1)
   }
 }
# RE MODEL (BL and F-up data)
for(i in 1:nsBF){                      # LOOP THROUGH STUDIES WITH BL+FUP
DATA
  for (k in 2:naBF[i]){                  # LOOP THROUGH ARMS
    # trial-specific RE distributions
    delta[i+nsCFB,k] ~ dnorm(md[i+nsCFB,k], taud[i+nsCFB,k])
    md[i+nsCFB,k] <- d[tBF[i,k]] - d[tBF[i,1]] + sw[i+nsCFB,k]
    # precision of RE distributions (with multi-arm trial correction)
    taud[i+nsCFB,k] <- tau *2*(k-1)/k
    #adjustment, multi-arm RCTs
    w[i+nsCFB,k] <- delta[i+nsCFB,k] - d[tBF[i,k]] + d[tBF[i,1]]
    # cumulative adjustment for multi-arm trials
    sw[i+nsCFB,k] <-sum(w[i+nsCFB,1:k-1])/(k-1)
   }
 }
# RE MODEL (Response data)
for(i in 1:nsR){                       # LOOP THROUGH STUDIES WITH
RESPONSE DATA
  for (k in 2:naR[i]){                 # LOOP THROUGH ARMS
    # trial-specific RE distributions
    delta[i+nsCFB+nsBF,k] ~ dnorm(md[i+nsCFB+nsBF,k],
taud[i+nsCFB+nsBF,k])
    md[i+nsCFB+nsBF,k] <- d[tR[i,k]] - d[tR[i,1]] + sw[i+nsCFB+nsBF,k]
    # precision of RE distributions (with multi-arm trial correction)
    taud[i+nsCFB+nsBF,k] <- tau *2*(k-1)/k
    #adjustment, multi-arm RCTs
    w[i+nsCFB+nsBF,k] <- delta[i+nsCFB+nsBF,k] - d[tR[i,k]] + d[tR[i,1]]
    # cumulative adjustment for multi-arm trials
    sw[i+nsCFB+nsBF,k] <-sum(w[i+nsCFB+nsBF,1:k-1])/(k-1)
  }
 }
#
totresdev <- sum(resdev[])               # Total Residual Deviance (all
data)
```

```
# Partial Residual Deviance
totresdev.p[1] <- sum(resdev[1:nsCFB])                    # CFB data
totresdev.p[2] <- sum(resdev[nsCFB+1:nsCFB+nsBF])         # BL + Fup
data
totresdev.p[3] <- sum(resdev[nsCFB+nsBF+1:nsCFB+nsBF+nsR]) # Response
data
#
# Priors and model assumptions (classes)
d[1] <- 0                  # treatment effect is zero for reference
treatment
# treatment effects from Class
for (k in 2:7) {
  d[k] <- m[D[k]]
}
for (k in 8:nt) {
  d[k] ~ dnorm(m[D[k]], prec2[D[k]])
}
for (k in 8:nc) {
  prec2[k] <- pow(sd2[k],-2)
}
for (k in 1:ncvar) {
  sd2[cvar[k]] ~ dunif(0,5)
}

# VARIANCE SHARING RULES
sd2[8] <- sd2[9]
sd2[10] <- sd2[9]
sd2[11] <- sd2[9]
sd2[12] <- sd2[9]
sd2[13] <- sd2[9]
sd2[14] <- sd2[9]
sd2[15] <- sd2[9]
sd2[18] <- sd2[9]
sd2[19] <- sd2[10]
sd2[20] <- sd2[10]
sd2[21] <- sd2[10]
sd2[23] <- sd2[9]
sd2[24] <- sd2[9]
sd2[25] <- sd2[9]
sd2[26] <- sd2[9]
sd2[29] <- sd2[27]
sd2[31] <- sd2[32]
sd2[32] <- sd2[9]
sd2[34] <- sd2[17]
sd2[35] <- sd2[10]
sd2[36] <- sd2[32]
sd2[37] <- sd2[38]
sd2[39] <- sd2[38]
sd2[40] <- sd2[38]
sd2[41] <- sd2[38]
sd2[42] <- sd2[38]
sd2[43] <- sd2[38]
sd2[44] <- sd2[38]
sd2[45] <- sd2[38]
sd2[46] <- sd2[38]
sd2[47] <- sd2[38]
sd2[48] <- sd2[38]
sd2[50] <- sd2[32]
sd2[22] <- max(sd2[9], sd2[10])
sd2[30] <- max(sd2[27], sd2[28])
```

```
m[1] <- 0
# prior for mean class effect
for (k in 2:nc){  m[k] ~ dnorm(0, .0001)   }

#
sdev ~ dunif(0,5)                      # vague prior for between-trial SD
tau <- pow(sdev,-2)                     # between-trial precision
# all pairwise differences
for (c in 1:(nt-1)) {  for (k in (c+1):nt)  { diff[c,k] <- d[k] - d[c]  }

# pairwise SMDs for all possible class comparisons
for (c in 1:(nc-1)){
  for (k in (c+1):nc){ diffClass[c,k] <- (m[k]-m[c]) }
 }
# treatments of interest to rank
for (k in 1:ntR) {
      dR[k] <- d[tRcode[k]]
}
# classes of interest to rank
for (k in 1:ncR) {
      mR[k] <- m[cRcode[k]]
}

for (k in 1:ntR){
  rk[k] <- rank(dR[],k)         # lower values are "good"
  best[k]  <- equals(rk[k],1)   # Smallest is best (i.e. rank 1)
  # prob treat k is h-th best, prob[1,k]=best[k]
  for (h in 1:ntR) { prob[h,k] <- equals(rk[k],h) }
 }
#
for (k in 1:ncR){
  rkClass[k]  <- rank(mR[],k)   # lower values are "good"
  bestClass[k]  <- equals(rkClass[k],1)    # Smallest is best (i.e. rank
1)
  # prob class k is h-th best, prob[1,k]=best[k]
  for (h in 1:ncR) { probClass[h,k] <- equals(rkClass[k],h) }
 }
}                                 # *** PROGRAM ENDS
```

Sample OpenBUGS code – Response analysis

```
# Random effects model for multi-arm trials
model{                                 # *** PROGRAM STARTS
for(i in 1:ns){                        #   LOOP THROUGH STUDIES
  w[i,1] <- 0   # adjustment for multi-arm trials is zero for control arm
  delta[i,1] <- 0                      # treatment effect is zero for
control arm
  deltaX[i,1] <- 0                     # treatment effect is zero for
control arm
  mu[i] ~ dnorm(0,.0001)               # vague priors for all trial
baselines
  muX[i] ~ dnorm(0,.0001)              # vague priors for all trial
baselines
 }
# (1) Response DATA
for(i in 1:nsR){
  for (k in 1:naR[i]){
    r[i,k] ~ dbin(p[i,k],nR[i,k])       # binomial likelihood
    logit(p[i,k]) <- mu[i] + delta[i,k] # model for linear predictor
```

```
    rhat[i,k] <- p[i,k] * nR[i,k]       # expected value of the 
numerators
    #Deviance contribution
    dev[i,k] <- 2 * (r[i,k] * (log(r[i,k])-log(rhat[i,k]))
         +  (nR[i,k]-r[i,k]) * (log(nR[i,k]-r[i,k]) - log(nR[i,k]-
rhat[i,k])))
   }
#Summed residual deviance contribution for this trial
  resdev[i] <- sum(dev[i,1:naR[i]])
 }
# (2) CFB DATA
for(i in 1:nsCFB){
  # calculate pooled.sd and adjustment for SMD
  df[i] <- sum(nCFB[i,1:naCFB[i]]) - naCFB[i] # denominator for 
pooled.var
  Pooled.var[i] <- sum(nvar[i,1:naCFB[i]])/df[i]
  # pooled sd for study i, for SMD
  Pooled.sd[i] <- sqrt(Pooled.var[i])
  H[i] <- 1                          # use Cohen's d (ie no adjustment)
  for (k in 1:naCFB[i]){
    se[i,k] <- sdCFB[i,k]/sqrt(nCFB[i,k])
    var[i,k] <- pow(se[i,k],2)       # calcultate variances
    prec[i,k] <- 1/var[i,k]          # set precisions
    yCFB[i,k] ~ dnorm(phi[i,k], prec[i,k]) # normal likelihood
    phi[i,k] <- theta[i,k] * (Pooled.sd[i]/H[i]) # theta is SMD
    theta[i,k] <- muX[i] + deltaX[i,k] # model for linear predictor
    dev[i+nsR,k] <- (yCFB[i,k]-phi[i,k])*(yCFB[i,k]-phi[i,k])*prec[i,k]
    nvar[i,k] <- (nCFB[i,k]-1) * pow(sdCFB[i,k],2) # for pooled.sd
   }
  # summed residual deviance contribution for this trial
  resdev[i+nsR] <- sum(dev[i+nsR,1:naCFB[i]])
 }
# (3) BASELINE + FOLLOW-UP DATA (no CFB)
for(i in 1:nsBF){                        #   LOOP THROUGH STUDIES
  # calculate pooled.sd and adjustment for SMD
  df[i+nsCFB] <- sum(n[i,1:naBF[i]]) - naBF[i] # denominator for 
pooled.var
  Pooled.var[i+nsCFB] <- sum(nvarBF[i,1:naBF[i]])/df[i+nsCFB]
  # pooled sd for study i, for SMD
  Pooled.sd[i+nsCFB] <- sqrt(Pooled.var[i+nsCFB])
  H[i+nsCFB] <- 1                      # use Cohen's d (ie no adjustment)
  for (k in 1:naBF[i]){
     yBF[i,k] <- yF[i,k] - yB[i,k]      # calculate mean CFB
    seF[i,k] <- sdF[i,k]/sqrt(n[i,k]) # se at followup
    seB[i,k] <- sdB[i,k]/sqrt(n[i,k]) # se at baseline
    # variance of mean CFB, assuming correlation rho
    var[i+nsCFB,k] <- pow(seF[i,k],2)+ pow(seB[i,k],2)
-2*(seF[i,k]*seB[i,k]*rho)
    prec[i+nsCFB,k] <- 1/var[i+nsCFB,k] # set CFB precisions
    yBF[i,k] ~ dnorm(phi[i+nsCFB,k], prec[i+nsCFB,k]) # normal likelihood
    # theta is SMD
    phi[i+nsCFB,k] <- theta[i+nsCFB,k] * (Pooled.sd[i+nsCFB]/H[i+nsCFB])
    # model for linear predictor
    theta[i+nsCFB,k] <- muX[i+nsCFB] + deltaX[i+nsCFB,k]
    # residual deviance contribution
    dev[i+nsR+nsCFB,k] <- (yBF[i,k]-phi[i+nsCFB,k]) * (yBF[i,k]-
phi[i+nsCFB,k])                            * prec[i+nsCFB,k]
    # Variance of baseline for pooled SD
    varBF[i,k] <- pow(sdB[i,k],2)
    nvarBF[i,k] <- (n[i,k]-1) * varBF[i,k] # for pooled.sd
   }
```

```
   # summed residual deviance contribution for this trial
   resdev[i+nsR+nsCFB] <- sum(dev[i+nsR+nsCFB,1:naBF[i]])
  }
# RE MODEL (Response data)
for(i in 1:nsR){                          # LOOP THROUGH STUDIES WITH
RESPONSE DATA
  for (k in 2:naR[i]){                    # LOOP THROUGH ARMS
    # trial-specific RE distributions
    delta[i,k] ~ dnorm(md[i,k], taud[i,k])
    md[i,k] <- d[tR[i,k]] - d[tR[i,1]] + sw[i,k]
    # precision of RE distributions (with multi-arm trial correction)
    taud[i,k] <- tau *2*(k-1)/k
    #adjustment, multi-arm RCTs
    w[i,k] <- delta[i,k] - d[tR[i,k]] + d[tR[i,1]]
    # cumulative adjustment for multi-arm trials
    sw[i,k] <-sum(w[i,1:k-1])/(k-1)
  }
 }
# RE MODEL (CFB data)
for(i in 1:nsCFB){                        # LOOP THROUGH STUDIES WITH CFB
DATA
  for (k in 2:naCFB[i]){                  # LOOP THROUGH ARMS
    # convert SMD to LOR
    deltaX[i,k] <- delta[i+nsR,k]*((sqrt(3))/-3.1416)*beta
    # trial-specific RE distributions
    delta[i+nsR,k] ~ dnorm(md[i+nsR,k], taud[i+nsR,k])
    md[i+nsR,k] <- d[tCFB[i,k]] - d[tCFB[i,1]] + sw[i+nsR,k]
    # precision of RE distributions (with multi-arm trial correction)
    taud[i+nsR,k] <- tau *2*(k-1)/k
    #adjustment, multi-arm RCTs
    w[i+nsR,k] <- delta[i+nsR,k] - d[tCFB[i,k]] + d[tCFB[i,1]]
    # cumulative adjustment for multi-arm trials
    sw[i+nsR,k] <-sum(w[i+nsR,1:k-1])/(k-1)
   }
 }
# RE MODEL (BL and F-up data)
for(i in 1:nsBF){                         # LOOP THROUGH STUDIES WITH BL+FUP
DATA
  for (k in 2:naBF[i]){                     # LOOP THROUGH ARMS
    # convert SMD to LOR
    deltaX[i+nsCFB,k] <- delta[i+nsR+nsCFB,k]*((sqrt(3))/-3.1416)*beta
    # trial-specific RE distributions
    delta[i+nsR+nsCFB,k] ~ dnorm(md[i+nsR+nsCFB,k], taud[i+nsR+nsCFB,k])
    md[i+nsR+nsCFB,k] <- d[tBF[i,k]] - d[tBF[i,1]] + sw[i+nsR+nsCFB,k]
    # precision of RE distributions (with multi-arm trial correction)
    taud[i+nsR+nsCFB,k] <- tau *2*(k-1)/k
    #adjustment, multi-arm RCTs
    w[i+nsR+nsCFB,k] <- delta[i+nsR+nsCFB,k] - d[tBF[i,k]] + d[tBF[i,1]]
    # cumulative adjustment for multi-arm trials
    sw[i+nsR+nsCFB,k] <-sum(w[i+nsR+nsCFB,1:k-1])/(k-1)
   }
 }

totresdev <- sum(resdev[])                  # Total Residual Deviance (all
data)
# Partial Residual Deviance
totresdev.p[1] <- sum(resdev[1:nsR])                      # Response data
totresdev.p[2] <- sum(resdev[nsR+1:nsR+nsCFB])           # CFB data
totresdev.p[3] <- sum(resdev[nsR+nsCFB+1:nsR+nsCFB+nsBF]) # Response data

# Priors and model assumptions (classes)
```

```
d[1] <- 0                   # treatment effect is zero for reference
treatment
# treatment effects from Class
for (k in 2:7) {
  d[k] <- m[D[k]]
}
for (k in 8:nt) {
  d[k] ~ dnorm(m[D[k]], prec2[D[k]])
}
for (k in 8:nc) {
  prec2[k] <- pow(sd2[k],-2)
}
for (k in 1:ncvar) {
  sd2[cvar[k]] ~ dunif(0,7)
}

# Class variance sharing
sd2[8] <- sd2[9]
sd2[10] <- sd2[9]
sd2[11] <- sd2[9]
sd2[12] <- sd2[9]
sd2[13] <- sd2[9]
sd2[14] <- sd2[9]
sd2[15] <- sd2[9]
sd2[18] <- sd2[9]
sd2[19] <- sd2[10]
sd2[21] <- sd2[9]
sd2[22] <- sd2[9]
sd2[23] <- sd2[9]
sd2[25] <- sd2[24]
sd2[26] <- sd2[24]
sd2[28] <- sd2[29]
sd2[29] <- sd2[9]
sd2[30] <- sd2[17]
sd2[31] <- sd2[17]
sd2[32] <- sd2[10]
sd2[33] <- sd2[29]
sd2[34] <- sd2[35]
sd2[36] <- sd2[35]
sd2[37] <- sd2[35]
sd2[38] <- sd2[35]
sd2[39] <- sd2[35]
sd2[40] <- sd2[35]
sd2[41] <- sd2[35]
sd2[42] <- sd2[35]
sd2[43] <- sd2[29]
sd2[44] <- sd2[29]
sd2[20] <- max(sd2[9], sd2[10])
sd2[27] <- max(sd2[24], sd2[25])

m[1] <- 0
# prior for mean class effect
for (k in 2:nc){  m[k] ~ dnorm(0, .0001)  }

#
beta <- 1
sdev ~ dunif(0,5)                   # vague prior for between-trial SD
tau <- pow(sdev,-2)                  # between-trial precision
# all pairwise differences
for (c in 1:(nt-1)) {
      for (k in (c+1):nt)  {
```

```
            or[c,k] <- exp(d[k] - d[c])
    lor[c,k] <- (d[k]-d[c])
            }
      }
# pairwise SMDs for all possible class comparisons
for (c in 1:(nc-1)){
  for (k in (c+1):nc){
            lorClass[c,k] <- m[k] - m[c]
    orClass[c,k] <- exp(m[k] - m[c])
            }
 }

# treatments of interest to rank
for (k in 1:ntR) {
      dR[k] <- d[tRcode[k]]
}
# classes of interest to rank
for (k in 1:ncR) {
      mR[k] <- m[cRcode[k]]
}

for (k in 1:ntR){
  rk[k] <- ntR+1-rank(dR[],k)  # lower values are "bad"
  best[k]  <- equals(rk[k],1)  # Smallest is best (i.e. rank 1)
  # prob treat k is h-th best, prob[1,k]=best[k]
  for (h in 1:ntR) { prob[h,k] <- equals(rk[k],h) }
 }
#
for (k in 1:ncR){
  rkClass[k]  <- ncR+1-rank(mR[],k)   # lower values are "bad"
  bestClass[k]  <- equals(rkClass[k],1)    # Smallest is best (i.e. rank
1)
  # prob class k is h-th best, prob[1,k]=best[k]
  for (h in 1:ncR) { probClass[h,k] <- equals(rkClass[k],h) }
 }
}                                 # *** PROGRAM ENDS
```

Methods of inconsistency checks and statistical codes

Methods of inconsistency checks

The assumption of consistency between direct and indirect evidence was explored by comparing the fit of a model assuming consistency with a model which allowed for inconsistency (also known as an 'unrelated mean effects' model) at the intervention level, whilst still modelling class effects.[67] The latter is equivalent to having separate, unrelated meta-analyses for every pair-wise contrast while assuming a common between-study heterogeneity across all comparisons in the case of random treatment effects models. Improvement in model fit or a substantial reduction in heterogeneity in the inconsistency model compared with the NMA consistency model indicates evidence of inconsistency. Inconsistency can only be assessed when there are closed loops of direct evidence on 3 treatments that are informed by at least 3 distinct trials.[68] The consistency and inconsistency models were compared based on their posterior residual deviance, which is a measure of the magnitude of the difference between the observed data and their model predictions.[61] Smaller values are preferred, and in a well-fitting model the posterior mean residual deviance should be close to the number of data points.[61] In addition, the deviance information criterion (DIC) was checked, which penalises model fit with model complexity.[61] Differences of ≥5 points for posterior mean residual deviance and DIC were considered meaningful.[61] Heterogeneity (measured by the posterior median between-study SD) was compared between the two models; smaller heterogeneity in the inconsistency model compared to the consistency model was an indication of potential inconsistency in the data.

Deviance plots, in which the posterior mean deviance of the individual data points in the inconsistency model were plotted against their posterior mean deviance in the consistency model, were inspected for each outcome, in order to identify studies which may have contributed to loops of evidence where inconsistency may be present. Points lying below the line of equality indicated data points contributing to inconsistency.

Direct estimates from the unrelated mean effect model were compared to NMA estimates from the consistency models. To identify comparisons for which there was likely to be a discrepancy between direct and indirect estimates, we estimated the indirect evidence contributions by subtracting the direct evidence contributions estimated using the unrelated mean effects model from the NMA estimates estimated using the consistency model, assuming normality of the posterior distributions:

$$d_{ind} = \frac{d_{nma}(w_{dir} + w_{ind}) - w_{dir} d_{dir}}{w_{ind}}$$

Where d_{ind} is the indirect relative effect, d_{nma} is the mixed relative effect estimated from the NMA, d_{dir} is the direct relative effect estimated from the inconsistency model, for a given treatment comparison. w_{nma}, w_{dir} and w_{ind} are the inverse-variance weights, calculated as $\frac{1}{\sigma_{nma}^2}$, $\frac{1}{\sigma_{dir}^2}$ and $\frac{1}{\sigma_{ind}^2}$ for the mixed, direct and indirect effects respectively; σ_{nma} and σ_{dir} are the standard deviations of the posterior distributions for the corresponding relative effects; σ_{ind} is the standard error for the indirect relative effect, calculated as:

$$\sigma_{ind} = \sqrt{\frac{\sigma_{nma}^2 \sigma_{dir}^2}{\sigma_{dir}^2 - \sigma_{nma}^2}}$$

The difference between direct and indirect estimates can then be estimated, and a Wald test can be used to test whether direct and indirect evidence are in agreement. We acknowledge that the posterior distributions may not be normally distributed, and hence we used this approach as a heuristic to identify comparisons in which direct and indirect evidence are likely to strongly disagree, given the large number of comparisons in many of the networks.

When evidence of inconsistency was found, studies contributing to loops of evidence with potential inconsistency were checked for data accuracy and analyses were repeated if data corrections were made. However, if evidence of inconsistency was still present following

data corrections, no studies were excluded from analysis. Given that inconsistency within a loop could be caused by any of the comparisons in that loop rather than a specific study/comparison, their results could not be considered as less valid than those of other studies solely based on the inconsistency findings. Nevertheless, the presence of inconsistency in the NMA was highlighted and results were interpreted accordingly.

Sample statistical OpenBUGS codes used to perform global inconsistency checks are provided below. Further codes used for global inconsistency checks are available upon request.

Statistical codes for global inconsistency checks

Sample OpenBUGS code – SMD unrelated mean effects model

```
# Normal likelihood, identity link: SMD with arm-based means
# Random effects model for multi-arm trials
model{                                     # *** PROGRAM STARTS
for(i in 1:ns){                            #   LOOP THROUGH STUDIES
  delta[i,1] <- 0                          # treatment effect is zero for
control arm
  mu[i] ~ dnorm(0,.0001)                   # vague priors for all trial
baselines
 }
# (1) CFB DATA
for(i in 1:nsCFB){
  # calculate pooled.sd and adjustment for SMD
  df[i] <- sum(nCFB[i,1:naCFB[i]]) - naCFB[i] # denominator for
pooled.var
  Pooled.var[i] <- sum(nvar[i,1:naCFB[i]])/df[i]
  # pooled sd for study i, for SMD
  Pooled.sd[i] <- sqrt(Pooled.var[i])
  H[i] <- 1                                # use Cohen's d (ie no
adjustment)
  for (k in 1:naCFB[i]){
    se[i,k] <- sdCFB[i,k]/sqrt(nCFB[i,k])
    var[i,k] <- pow(se[i,k],2)             # calcultate variances
    prec[i,k] <- 1/var[i,k]                # set precisions
    yCFB[i,k] ~ dnorm(phi[i,k], prec[i,k]) # normal likelihood
    phi[i,k] <- theta[i,k] * (Pooled.sd[i]/H[i]) # theta is stand
mean
    theta[i,k] <- mu[i] + delta[i,k] # model for linear predictor,
delta is SMD
    dev[i,k] <- (yCFB[i,k]-phi[i,k])*(yCFB[i,k]-phi[i,k])*prec[i,k]
    nvar[i,k] <- (nCFB[i,k]-1) * pow(sdCFB[i,k],2) # for pooled.sd
   }
  # summed residual deviance contribution for this trial
  resdev[i] <- sum(dev[i,1:naCFB[i]])
 }
```

```
# (2) BASELINE + FOLLOW-UP DATA (no CFB)
for(i in 1:nsBF){                          #   LOOP THROUGH STUDIES
  # calculate pooled.sd and adjustment for SMD
  df[i+nsCFB] <- sum(n[i,1:naBF[i]]) - naBF[i] # denominator for
pooled.var
  Pooled.var[i+nsCFB] <- sum(nvarBF[i,1:naBF[i]])/df[i+nsCFB]
  # pooled sd for study i, for SMD
  Pooled.sd[i+nsCFB] <- sqrt(Pooled.var[i+nsCFB])
  H[i+nsCFB] <- 1                          # use Cohen's d (ie no
adjustment)
  for (k in 1:naBF[i]){
    yBF[i,k] <- yF[i,k] - yB[i,k]      # calculate mean CFB
           seF[i,k] <- sdF[i,k]/sqrt(n[i,k]) # se at followup
    seB[i,k] <- sdB[i,k]/sqrt(n[i,k]) # se at baseline
    # variance of mean CFB, assuming correlation corr[i]
    var[i+nsCFB,k] <- pow(seF[i,k],2)+ pow(seB[i,k],2)
-2*(seF[i,k]*seB[i,k]*rho)
    prec[i+nsCFB,k] <- 1/var[i+nsCFB,k] # set CFB precisions
    yBF[i,k] ~ dnorm(phi[i+nsCFB,k], prec[i+nsCFB,k]) # normal
likelihood
    # theta is standardised mean
    phi[i+nsCFB,k] <- theta[i+nsCFB,k] *
(Pooled.sd[i+nsCFB]/H[i+nsCFB])
    # model for linear predictor, delta is SMD
    theta[i+nsCFB,k] <- mu[i+nsCFB] + delta[i+nsCFB,k]
    # residual deviance contribution
    dev[i+nsCFB,k] <- (yBF[i,k]-phi[i+nsCFB,k]) * (yBF[i,k]-
phi[i+nsCFB,k])                                * prec[i+nsCFB,k]
    # Variance of baseline for pooled SD
    varBF[i,k] <- pow(sdB[i,k],2)
    nvarBF[i,k] <- (n[i,k]-1) * varBF[i,k] # for pooled.sd
   }
  # summed residual deviance contribution for this trial
  resdev[i+nsCFB] <- sum(dev[i+nsCFB,1:naBF[i]])
 }
# (3) RESPONSE DATA (no CFB or BL+follow-up)
for(i in 1:nsR){                          #   LOOP THROUGH STUDIES
  # calculate pooled.sd and adjustment for SMD
  df[i+nsCFB+nsBF] <- sum(nR[i,1:naR[i]]) - naR[i] # denominator
for pooled.var
  Pooled.var[i+nsCFB+nsBF] <-
sum(nvarR[i,1:naR[i]])/df[i+nsCFB+nsBF]
  # pooled sd for study i, for SMD
  Pooled.sd[i+nsCFB+nsBF] <- sqrt(Pooled.var[i+nsCFB+nsBF])
  H[i+nsCFB+nsBF] <- 1                    # use Cohen's d (ie no
adjustment)
  for (k in 1:naR[i]){
    r[i,k] ~ dbin(R[i,k], nR[i,k])    # binomial likelihood
    R[i,k] <- phi.adj[i,k]
    x[i,k] <- -(q[i]*yBR[i,k]+ phi[i+nsCFB+nsBF,k])/(sdBR[i,k] *
sqrt(1+(1-q[i])*(1-q[i]-2*rho)))
    # adjust link function phi(x) for extreme values that can give
numerical
    # errors when x< -5, phi(x)=0, when x> 5, phi(x)=1
    phi.adj[i,k] <- (step(5+x[i,k]) * step(x[i,k]-5)
```

```
           + step(5-x[i,k])* step(x[i,k]+5) * phi(x[i,k]))*(1-
equals(x[i,k],5))
           + equals(x[i,k],5)   # correct for x=5
    # theta is standardised mean
    phi[i+nsCFB+nsBF,k] <- theta[i+nsCFB+nsBF,k]
                * (Pooled.sd[i+nsCFB+nsBF]/H[i+nsCFB+nsBF])
    # model for linear predictor, delta is SMD
    theta[i+nsCFB+nsBF,k] <- mu[i+nsCFB+nsBF] +
delta[i+nsCFB+nsBF,k]
    # residual deviance contribution
    rhat[i,k] <- R[i,k] * nR[i,k]
    dev[i+nsCFB+nsBF,k] <- 2 * (r[i,k] * (log(r[i,k])-
log(rhat[i,k]))
         + (nR[i,k]-r[i,k]) * (log(nR[i,k]-r[i,k]) - log(nR[i,k]-
rhat[i,k])))
    sdR[i,k] <-  4.46 + (sdBR[i,k] * 0.55) # sd for response in
severe group
    # Variance of baseline for pooled SD
    nvarR[i,k] <- (nR[i,k]-1) * pow(sdBR[i,k],2) # for pooled.sd
   }
  # summed residual deviance contribution for this trial
  resdev[i+nsCFB+nsBF] <- sum(dev[i+nsCFB+nsBF,1:naR[i]])
 }
#
# RE MODEL (CFB data)
for(i in 1:nsCFB){                   # LOOP THROUGH STUDIES WITH
CFB DATA
  for (k in 2:naCFB[i]){             # LOOP THROUGH ARMS
    # trial-specific RE distributions
    delta[i,k] ~ dnorm(md[i,k], tau)
    md[i,k] <- d[tCFB[i,k], tCFB[i,1]]
   }
 }
# RE MODEL (BL and F-up data)
for(i in 1:nsBF){                    # LOOP THROUGH STUDIES WITH
BL+FUP DATA
  for (k in 2:naBF[i]){               # LOOP THROUGH ARMS
    # trial-specific RE distributions
    delta[i+nsCFB,k] ~ dnorm(md[i+nsCFB,k], tau)
    md[i+nsCFB,k] <- d[tBF[i,k], tBF[i,1]]
   }
 }
# RE MODEL (Response data)
for(i in 1:nsR){                    # LOOP THROUGH STUDIES WITH
RESPONSE DATA
  for (k in 2:naR[i]){               # LOOP THROUGH ARMS
    # trial-specific RE distributions
    delta[i+nsCFB+nsBF,k] ~ dnorm(md[i+nsCFB+nsBF,k], tau)
    md[i+nsCFB+nsBF,k] <- d[tR[i,k], tR[i,1]]
   }
 }
#
totresdev <- sum(resdev[])            # Total Residual Deviance
(all data)
# Partial Residual Deviance
```

```
totresdev.p[1] <- sum(resdev[1:nsCFB])                    # CFB
data
totresdev.p[2] <- sum(resdev[nsCFB+1:nsCFB+nsBF])         # BL +
Fup data
totresdev.p[3] <- sum(resdev[nsCFB+nsBF+1:nsCFB+nsBF+nsR]) #
Response data
#
# treatment effects from Class (unrelated mean effects)
for (c in 1:(nt-1)){
      d[c,c] <- 0
      for (k in (c+1):nt) {
            # Ensures d is fixed if class has 0 variance for ALL
comparisons
            d[c,k] <- m[D[c],D[k]] + (step(step(D[c]-7) + step(D[k]-
7)) * dvar[c,k])
            dvar[c,k] ~ dnorm(0, tau.class[D[c],D[k]])
            d[k,c] <- -d[c,k]
      }
}

m[nc,nc] <- 0
for (c1 in 1:(nc-1)){
      m[c1,c1] <- 0
      for (c2 in (c1+1):nc){
            m[c1,c2] ~ dnorm(0,.0001)
      }
}
for (c1 in 1:nc){
      for (c2 in 1:nc){
            tau.class[c1,c2] <- pow(sd.class[c1,c2], -2)
            sd.class[c1,c2] <- max(sd2[c1], sd2[c2])
      }
}

for (k in 1:7) {
      sd2[k] <- 0.0001 # v.low sd for ~0 class variance
}
for (k in 8:nc) {
  prec2[k] <- pow(sd2[k],-2)
}
for (k in 1:ncvar) {
  sd2[cvar[k]] ~ dunif(0,5)
}

# Variance sharing rules
sd2[8] <- sd2[9]
sd2[10] <- sd2[9]
sd2[11] <- sd2[9]
sd2[12] <- sd2[9]
sd2[13] <- sd2[9]
sd2[14] <- sd2[9]
sd2[15] <- sd2[9]
sd2[18] <- sd2[9]
sd2[19] <- sd2[10]
sd2[20] <- sd2[10]
```

```
sd2[21] <- sd2[10]
sd2[23] <- sd2[9]
sd2[24] <- sd2[9]
sd2[25] <- sd2[9]
sd2[26] <- sd2[9]
sd2[29] <- sd2[27]
sd2[31] <- sd2[32]
sd2[32] <- sd2[9]
sd2[34] <- sd2[17]
sd2[35] <- sd2[10]
sd2[36] <- sd2[32]
sd2[37] <- sd2[38]
sd2[39] <- sd2[38]
sd2[40] <- sd2[38]
sd2[41] <- sd2[38]
sd2[42] <- sd2[38]
sd2[43] <- sd2[38]
sd2[44] <- sd2[38]
sd2[45] <- sd2[38]
sd2[46] <- sd2[38]
sd2[47] <- sd2[38]
sd2[48] <- sd2[38]
sd2[50] <- sd2[32]
sd2[22] <- max(sd2[9], sd2[10])
sd2[30] <- max(sd2[27], sd2[28])

#
sdev ~ dunif(0,5)      # vague prior for between-trial SD
tau <- pow(sdev,-2)    # between-trial precision = (1/between-trial
variance)
# pairwise ORs for all possible pair-wise comparisons
#for (c in 1:(nt-1)){
#   for (k in (c+1):nt){
#     or[c,k] <- exp(d[c,k])
#    }
# }

# Pairwise ORs for all possible pair-wise class combinations
#for (c in 1:(nc-1)){
#   for (k in (c+1):nc){
#     orClass[c,k] <- exp(m[c,k])
#    }
# }
}                                # *** PROGRAM ENDS
```

Sample OpenBUGS code – Response unrelated mean effects model

```
# Random effects model for multi-arm trials
model{                                    # *** PROGRAM STARTS
for(i in 1:ns){                           #   LOOP THROUGH STUDIES
  delta[i,1] <- 0                         # treatment effect is zero for
control arm
  deltaX[i,1] <- 0                     # treatment effect is zero for
control arm
```

```
  mu[i] ~ dnorm(0,.0001)              # vague priors for all trial
baselines
  muX[i] ~ dnorm(0,.0001)          # vague priors for all trial
baselines
 }
# (1) Response DATA
for(i in 1:nsR){
  for (k in 1:naR[i]){
    r[i,k] ~ dbin(p[i,k],nR[i,k])        # binomial likelihood
    logit(p[i,k]) <- mu[i] + delta[i,k] # model for linear
predictor
    rhat[i,k] <- p[i,k] * nR[i,k]        # expected value of the
numerators
    #Deviance contribution
    dev[i,k] <- 2 * (r[i,k] * (log(r[i,k])-log(rhat[i,k]))
          +  (nR[i,k]-r[i,k]) * (log(nR[i,k]-r[i,k]) - log(nR[i,k]-
rhat[i,k])))
   }
#Summed residual deviance contribution for this trial
  resdev[i] <- sum(dev[i,1:naR[i]])
 }
# (2) CFB DATA
for(i in 1:nsCFB){
  # calculate pooled.sd and adjustment for SMD
  df[i] <- sum(nCFB[i,1:naCFB[i]]) - naCFB[i] # denominator for
pooled.var
  Pooled.var[i] <- sum(nvar[i,1:naCFB[i]])/df[i]
  # pooled sd for study i, for SMD
  Pooled.sd[i] <- sqrt(Pooled.var[i])
  H[i] <- 1                          # use Cohen's d (ie no
adjustment)
  for (k in 1:naCFB[i]){
    se[i,k] <- sdCFB[i,k]/sqrt(nCFB[i,k])
    var[i,k] <- pow(se[i,k],2)       # calcultate variances
    prec[i,k] <- 1/var[i,k]          # set precisions
    yCFB[i,k] ~ dnorm(phi[i,k], prec[i,k]) # normal likelihood
    phi[i,k] <- theta[i,k] * (Pooled.sd[i]/H[i]) # theta is SMD
    theta[i,k] <- muX[i] + deltaX[i,k] # model for linear predictor
    dev[i+nsR,k] <- (yCFB[i,k]-phi[i,k])*(yCFB[i,k]-
phi[i,k])*prec[i,k]
    nvar[i,k] <- (nCFB[i,k]-1) * pow(sdCFB[i,k],2) # for pooled.sd
   }
  # summed residual deviance contribution for this trial
  resdev[i+nsR] <- sum(dev[i+nsR,1:naCFB[i]])
 }
# (3) BASELINE + FOLLOW-UP DATA (no CFB)
for(i in 1:nsBF){                      #   LOOP THROUGH STUDIES
  # calculate pooled.sd and adjustment for SMD
  df[i+nsCFB] <- sum(n[i,1:naBF[i]]) - naBF[i] # denominator for
pooled.var
  Pooled.var[i+nsCFB] <- sum(nvarBF[i,1:naBF[i]])/df[i+nsCFB]
  # pooled sd for study i, for SMD
  Pooled.sd[i+nsCFB] <- sqrt(Pooled.var[i+nsCFB])
  H[i+nsCFB] <- 1                      # use Cohen's d (ie no
adjustment)
```

```
  for (k in 1:naBF[i]){
     yBF[i,k] <- yF[i,k] - yB[i,k]      # calculate mean CFB
    seF[i,k] <- sdF[i,k]/sqrt(n[i,k]) # se at followup
    seB[i,k] <- sdB[i,k]/sqrt(n[i,k]) # se at baseline
# variance of mean CFB, assuming correlation corr[i]
    var[i+nsCFB,k] <- pow(seF[i,k],2)+ pow(seB[i,k],2)
-2*(seF[i,k]*seB[i,k]*rho)
    prec[i+nsCFB,k] <- 1/var[i+nsCFB,k] # set CFB precisions
    yBF[i,k] ~ dnorm(phi[i+nsCFB,k], prec[i+nsCFB,k]) # normal
likelihood
# theta is SMD
    phi[i+nsCFB,k] <- theta[i+nsCFB,k] *
(Pooled.sd[i+nsCFB]/H[i+nsCFB])
# model for linear predictor
    theta[i+nsCFB,k] <- muX[i+nsCFB] + deltaX[i+nsCFB,k]
# residual deviance contribution
    dev[i+nsR+nsCFB,k] <- (yBF[i,k]-phi[i+nsCFB,k]) * (yBF[i,k]-
phi[i+nsCFB,k])                             * prec[i+nsCFB,k]
    # Variance of baseline for pooled SD
    varBF[i,k] <- pow(sdB[i,k],2)
    nvarBF[i,k] <- (n[i,k]-1) * varBF[i,k] # for pooled.sd
   }
  # summed residual deviance contribution for this trial
  resdev[i+nsR+nsCFB] <- sum(dev[i+nsR+nsCFB,1:naBF[i]])
 }
# RE MODEL (Response data)
for(i in 1:nsR){                      # LOOP THROUGH STUDIES WITH
RESPONSE DATA
  for (k in 2:naR[i]){                # LOOP THROUGH ARMS
    # trial-specific RE distributions
    delta[i,k] ~ dnorm(md[i,k], tau)
    md[i,k] <- d[tR[i,k], tR[i,1]]
  }
 }
# RE MODEL (CFB data)
for(i in 1:nsCFB){                    # LOOP THROUGH STUDIES WITH
CFB DATA
  for (k in 2:naCFB[i]){              # LOOP THROUGH ARMS
    # convert SMD to LOR
    deltaX[i,k] <- delta[i+nsR,k]*((sqrt(3))/-3.1416)*beta
    # trial-specific RE distributions
    delta[i+nsR,k] ~ dnorm(md[i+nsR,k], tau)
    md[i+nsR,k] <- d[tCFB[i,k], tCFB[i,1]]
   }
 }
# RE MODEL (BL and F-up data)
for(i in 1:nsBF){                     # LOOP THROUGH STUDIES WITH
BL+FUP DATA
  for (k in 2:naBF[i]){                 # LOOP THROUGH ARMS
    # convert SMD to LOR
    deltaX[i+nsCFB,k] <- delta[i+nsR+nsCFB,k]*((sqrt(3))/-
3.1416)*beta
    # trial-specific RE distributions
    delta[i+nsR+nsCFB,k] ~ dnorm(md[i+nsR+nsCFB,k], tau)
    md[i+nsR+nsCFB,k] <- d[tBF[i,k], tBF[i,1]]
```

```
    }
  }
#
totresdev <- sum(resdev[])              # Total Residual Deviance
(all data)
# Partial Residual Deviance
totresdev.p[1] <- sum(resdev[1:nsR])                     # Response
data
totresdev.p[2] <- sum(resdev[nsR+1:nsR+nsCFB])          # CFB data
totresdev.p[3] <- sum(resdev[nsR+nsCFB+1:nsR+nsCFB+nsBF]) #
Response data
#
# Priors and model assumptions (classes)
# treatment effects from Class (unrelated mean effects)
for (c in 1:(nt-1)){
      d[c,c] <- 0
      for (k in (c+1):nt) {
            # Ensures d is fixed if class has 0 variance for ALL
comparisons
            d[c,k] <- m[D[c],D[k]] + (step(step(D[c]-7) + step(D[k]-
7)) * dvar[c,k])
            dvar[c,k] ~ dnorm(0, tau.class[D[c],D[k]])
            d[k,c] <- -d[c,k]
      }
}

m[nc,nc] <- 0
for (c1 in 1:(nc-1)){
      m[c1,c1] <- 0
      for (c2 in (c1+1):nc){
            m[c1,c2] ~ dnorm(0,.0001)
      }
}
for (c1 in 1:nc){
      for (c2 in 1:nc){
            tau.class[c1,c2] <- pow(sd.class[c1,c2], -2)
            sd.class[c1,c2] <- max(sd2[c1], sd2[c2])
      }
}

for (k in 1:7) {
      sd2[k] <- 0.0001 # v.low sd for ~0 class variance
}
for (k in 8:nc) {
  prec2[k] <- pow(sd2[k],-2)
}
for (k in 1:ncvar) {
  sd2[cvar[k]] ~ dunif(0,5)
}

# Variance sharing rules
sd2[8] <- sd2[9]
sd2[10] <- sd2[9]
sd2[11] <- sd2[9]
sd2[12] <- sd2[9]
```

```
sd2[13] <- sd2[9]
sd2[15] <- sd2[9]
sd2[16] <- sd2[9]
sd2[17] <- sd2[10]
sd2[18] <- sd2[10]
sd2[19] <- sd2[10]
sd2[21] <- sd2[9]
sd2[22] <- sd2[9]
sd2[25] <- sd2[23]
sd2[27] <- sd2[28]
sd2[30] <- sd2[15]
sd2[31] <- sd2[10]
sd2[32] <- sd2[28]
sd2[33] <- sd2[34]
sd2[35] <- sd2[34]
sd2[36] <- sd2[34]
sd2[37] <- sd2[34]
sd2[38] <- sd2[34]
sd2[39] <- sd2[34]
sd2[40] <- sd2[34]
sd2[41] <- sd2[34]
sd2[43] <- sd2[28]
sd2[20] <- max(sd2[9], sd2[10])
sd2[26] <- max(sd2[23], sd2[24])

#
beta <- 1
sdev ~ dunif(0,5)                    # vague prior for between-
trial SD
tau <- pow(sdev,-2)                   # between-trial precision

}                                # *** PROGRAM ENDS
```

Sample OpenBUGS code – Discontinuation unrelated mean effects model (also applicable to other outcomes with binomial likelihood)

```
# Binomial likelihood, logit link
# Random effects model for multi-arm trials
model{
for(i in 1:ns){
  delta[i,1] <- 0    # treatment effect is zero for control arm
  mu[i] ~ dnorm(0,.0001) # vague priors for all trial baselines
  for (k in 1:na[i]) {
    r[i,k] ~ dbin(p[i,k],n[i,k])                  # binomial
likelihood
    logit(p[i,k]) <- mu[i] + delta[i,k]          # model for
linear predictor
    rhat[i,k] <- p[i,k] * n[i,k]                  # expected value
of the numerators
#Deviance contribution
    dev[i,k] <- 2 * (r[i,k] * (log(r[i,k])-log(rhat[i,k]))
         +  (n[i,k]-r[i,k]) * (log(n[i,k]-r[i,k]) - log(n[i,k]-
rhat[i,k])))
   }
#Summed residual deviance contribution for this trial
```

```
  resdev[i] <- sum(dev[i,1:na[i]])
  for (k in 2:na[i]) {
    delta[i,k] ~ dnorm(md[i,k],tau)          # trial-specific LOR
distributions
    # mean of LOR distributions (with multi-arm trial correction)
    md[i,k] <-  d[t[i,1], t[i,k]]
   }
 }
totresdev <- sum(resdev[])            # Total Residual Deviance

# treatment effects from Class (unrelated mean effects)
for (c in 1:(nt-1)){
      d[c,c] <- 0
      for (k in (c+1):nt) {
            # Ensures d is fixed if class has 0 variance for ALL
comparisons
            d[c,k] <- m[D[c],D[k]] + (step(step(D[c]-8) + step(D[k]-
8)) * dvar[c,k])
            dvar[c,k] ~ dnorm(0, tau.class[D[c],D[k]])
            d[k,c] <- -d[c,k]
      }
}

m[nc,nc] <- 0
for (c1 in 1:(nc-1)){
      m[c1,c1] <- 0
      for (c2 in (c1+1):nc){
            m[c1,c2] ~ dnorm(0,.0001)
      }
}
for (c1 in 1:nc){
      for (c2 in 1:nc){
            tau.class[c1,c2] <- pow(sd.class[c1,c2], -2)
            sd.class[c1,c2] <- max(sd2[c1], sd2[c2])
      }
}

for (k in 1:8) {
      sd2[k] <- 0.0001 # v.low sd for ~0 class variance
}
for (k in 9:nc) {
  prec2[k] <- pow(sd2[k],-2)
}
for (k in 1:ncvar) {
  sd2[cvar[k]] ~ dunif(0,5)
}

# Variance sharing rules
sd2[9] <- sd2[11]
sd2[10] <- sd2[11]
sd2[12] <- sd2[11]
sd2[13] <- sd2[11]
sd2[14] <- sd2[11]
sd2[15] <- sd2[11]
sd2[16] <- sd2[11]
```

```
sd2[19] <- sd2[11]
sd2[20] <- sd2[11]
sd2[21] <- sd2[11]
sd2[22] <- sd2[11]
sd2[25] <- sd2[23]
sd2[26] <- sd2[27]
sd2[27] <- sd2[11]
sd2[28] <- sd2[18]
sd2[29] <- sd2[18]
sd2[30] <- sd2[12]
sd2[31] <- sd2[27]
sd2[33] <- sd2[32]
sd2[34] <- sd2[32]
sd2[35] <- sd2[32]
sd2[36] <- sd2[32]
sd2[37] <- sd2[32]
sd2[38] <- sd2[27]
sd2[39] <- sd2[27]

#
sd ~ dunif(0,5)      # vague prior for between-trial SD
tau <- pow(sd,-2)    # between-trial precision = (1/between-trial
variance)
# pairwise ORs for all possible pair-wise comparisons
for (c in 1:(nt-1)){
  for (k in (c+1):nt){
    or[c,k] <- exp(d[c,k])
   }
 }

# Pairwise ORs for all possible pair-wise class combinations
for (c in 1:(nc-1)){
  for (k in (c+1):nc){
    orClass[c,k] <- exp(m[c,k])
   }
 }
}                               # *** PROGRAM ENDS
```

Methods of bias adjustment models and statistical codes

Methods of bias adjustment models

Publication bias is known to affect results of meta-analyses in several clinical areas, including depression.[69-73] Small sample size studies are associated with publication bias as small studies with positive results are more likely to be published compared with small studies with negative results, and may also be associated with lower study quality. Published smaller studies tend to overestimate the relative treatment effect of interventions versus control, compared to larger studies.[71,74] Furthermore, small studies are often of poorer quality, and may be at higher risk of bias, which can lead to inflated estimates of efficacy and violate the transitivity assumption. Bias analysis of small study effects allows retaining all studies in the analysis, thus increasing power to detect any effect.

As the NMAs included a significant number of small studies, sensitivity analyses were carried out on selected outcomes, which adjusted for bias associated with small study size effects. The analyses, which were based on the assumption that the smaller the study the greater the bias, attempted to estimate the “true” treatment effect that would be obtained in a study of infinite size. The analyses assumed possible bias in comparisons of active interventions versus inactive control and no bias between inactive control comparisons, as well as between active intervention comparisons. The exception to this was in comparisons where non-directive counselling was the control intervention (in which case bias against non-directive counselling was assumed). This exception was based on concerns that non-directive counselling when used as a control intervention may be less likely to be manual-based, and to be delivered in a comparable number of sessions by an equivalent healthcare professional as when non-directive counselling is included as an active intervention in trials. Bias adjustment assumptions were supported by empirical evidence of the direction and magnitude of small study bias in meta-analyses of psychological interventions versus control[69] and of antidepressants versus pill placebo.[73]

Bias adjustment models were developed for the following outcomes synthesised in NMAs:

- SMD of depressive symptom change scores (primary efficacy outcome)
- Treatment discontinuation for any reason
- Response in completers

The latter two outcomes were selected for bias adjustment because they were the main NMA outcomes that informed the economic analysis, with the highest anticipated impact on the cost-effectiveness results.

The bias model acts to change the relative treatment effects of the treatment in arm *k* compared to the treatment in arm 1, for each study *i* on the outcome scale being modelled (SMD or logOR). This applies to the relative effects estimated from all included studies, whether the data are reported as change from baseline in measures of depression, depression measured at endpoint or as the number of responders to treatment. The only change required to incorporate the bias adjustment is to change

$\theta_{ik} = \gamma_i + \delta_{ik}$ (see Methods section of the Appendix)

to

$$\theta_{ik} = \gamma_i + \delta_{ik} + \left(\beta_{ik} \times V_{ik}\right)$$

where $\delta_{i1} = \beta_{i1} = V_{i1} = 0$, V_{ik} is the variance of the relative effect measure calculated for arm *k* of study *i* compared to arm 1, and β_{ik} represents the bias coefficient for the comparison of the treatment in arm *k* to the treatment in arm 1 of study *i* which is assumed to follow a Normal distribution

$$\beta_{ik} \sim \text{Normal}(B, \kappa^2_{SMD})$$

where $B=b$ if the treatment in arm 1 of trial i is a control and the treatment in arm k is not and $B=0$ if the comparison of treatment 1 to treatment k is active vs active or control vs control.

Bias-adjusted models were compared to random effects consistency models using DIC. If the bias-adjusted model had a DIC that was lower by ≥5 then results from this were reported over the unadjusted model.[61]

We used priors that are very diffuse on the relevant scale. For the SMD, the mean and standard deviation of the trial-specific bias due to study size were assigned Normal(0, 10000) and Uniform(0,50) prior distributions, respectively. For the discontinuation and response outcomes, the mean and standard deviation of the trial-specific bias due to outcome measurement were assigned Normal(0, 10000) and Uniform(0,5) prior distributions, respectively.

Sample statistical OpenBUGS codes used for bias adjustment are provided below. All codes tested are available upon request.

Statistical codes for bias adjustment

Sample OpenBUGS code – SMD bias analysis

```
 # Normal likelihood, identity link: SMD with arm-based means
# Random effects model for multi-arm trials
model{                                   # *** PROGRAM STARTS
for(i in 1:ns){                          #   LOOP THROUGH STUDIES
  w[i,1] <- 0   # adjustment for multi-arm trials is zero for
control arm
       beta[i,1]<-0                                #no bias term in
baseline arm
       V[i,1]<-0                              #no variance term in
baseline arm
  delta[i,1] <- 0                        # treatment effect is zero for
control arm
  mu[i] ~ dnorm(0,.0001)                 # vague priors for all trial
baselines
 }
# (1) CFB DATA
for(i in 1:nsCFB){
  # calculate pooled.sd and adjustment for SMD
  df[i] <- sum(nCFB[i,1:naCFB[i]]) - naCFB[i] # denominator for
pooled.var
  Pooled.var[i] <- sum(nvar[i,1:naCFB[i]])/df[i]
      # pooled sd for study i, for SMD
  Pooled.sd[i] <- sqrt(Pooled.var[i])
```

```
#  H[i] <- 1 - 3/(4*df[i]-1)          # use Hedges' g
  H[i] <- 1                          # use Cohen's d (ie no
adjustment)
  for (k in 1:naCFB[i]){
    se[i,k] <- sdCFB[i,k]/sqrt(nCFB[i,k])
    var[i,k] <- pow(se[i,k],2)       # calcultate variances
    prec[i,k] <- 1/var[i,k]          # set precisions
    yCFB[i,k] ~ dnorm(phi[i,k], prec[i,k]) # normal likelihood
    phi[i,k] <- theta[i,k] * (Pooled.sd[i]/H[i]) # theta is stand
mean
    theta[i,k] <- mu[i] + delta[i,k] + (beta[i,k]*V[i,k])
    dev[i,k] <- (yCFB[i,k]-phi[i,k])*(yCFB[i,k]-phi[i,k])*prec[i,k]
    nvar[i,k] <- (nCFB[i,k]-1) * pow(sdCFB[i,k],2) # for pooled.sd
   }
  # summed residual deviance contribution for this trial
  resdev[i] <- sum(dev[i,1:naCFB[i]])
 }
# (2) BASELINE + FOLLOW-UP DATA (no CFB)
for(i in 1:nsBF){                    #   LOOP THROUGH STUDIES
  # calculate pooled.sd and adjustment for SMD
  df[i+nsCFB] <- sum(n[i,1:naBF[i]]) - naBF[i] # denominator for
pooled.var
  Pooled.var[i+nsCFB] <- sum(nvarBF[i,1:naBF[i]])/df[i+nsCFB]
      # pooled sd for study i, for SMD
  Pooled.sd[i+nsCFB] <- sqrt(Pooled.var[i+nsCFB])
#  H[i] <- 1 - 3/(4*df[i]-1)           # use Hedges' g
  H[i+nsCFB] <- 1                      # use Cohen's d (ie no
adjustment)
  for (k in 1:naBF[i]){
    yBF[i,k] <- yF[i,k] - yB[i,k]      # calculate mean CFB
    seF[i,k] <- sdF[i,k]/sqrt(n[i,k]) # se at followup
    seB[i,k] <- sdB[i,k]/sqrt(n[i,k]) # se at baseline
    # variance of mean CFB, assuming correlation corr[i]
    var[i+nsCFB,k] <- pow(seF[i,k],2)+ pow(seB[i,k],2)
-2*(seF[i,k]*seB[i,k]*rho)
    prec[i+nsCFB,k] <- 1/var[i+nsCFB,k] # set CFB precisions
    yBF[i,k] ~ dnorm(phi[i+nsCFB,k], prec[i+nsCFB,k]) # normal
likelihood
    # theta is standardised mean
    phi[i+nsCFB,k] <- theta[i+nsCFB,k] *
(Pooled.sd[i+nsCFB]/H[i+nsCFB])
    # model for linear predictor, delta is SMD
    theta[i+nsCFB,k] <- mu[i+nsCFB] + delta[i+nsCFB,k] +
           (beta[i+nsCFB,k]*V[i+nsCFB,k])
    # residual deviance contribution
    dev[i+nsCFB,k] <- (yBF[i,k]-phi[i+nsCFB,k]) * (yBF[i,k]-
phi[i+nsCFB,k])                             * prec[i+nsCFB,k]
    # variance of CFB, assuming correlation corrBF[i] (var is sd
squared)
    #varBF[i,k] <- pow(sdF[i,k],2) + pow(sdB[i,k],2)
#             - 2*(sdF[i,k]*sdB[i,k]*rho)
           # Variance of baseline for pooled SD
           varBF[i,k] <- pow(sdB[i,k],2)
    nvarBF[i,k] <- (n[i,k]-1) * varBF[i,k] # for pooled.sd
   }
```

```
  # summed residual deviance contribution for this trial
  resdev[i+nsCFB] <- sum(dev[i+nsCFB,1:naBF[i]])
 }
# (3) RESPONSE DATA (no CFB or BL+follow-up)
for(i in 1:nsR){                          #   LOOP THROUGH STUDIES
  # calculate pooled.sd and adjustment for SMD
  df[i+nsCFB+nsBF] <- sum(nR[i,1:naR[i]]) - naR[i] # denominator
for pooled.var
  Pooled.var[i+nsCFB+nsBF] <-
sum(nvarR[i,1:naR[i]])/df[i+nsCFB+nsBF]
      # pooled sd for study i, for SMD
  Pooled.sd[i+nsCFB+nsBF] <- sqrt(Pooled.var[i+nsCFB+nsBF])
#  H[i] <- 1 - 3/(4*df[i]-1)           # use Hedges' g
  H[i+nsCFB+nsBF] <- 1                # use Cohen's d (ie no
adjustment)
  for (k in 1:naR[i]){
    r[i,k] ~ dbin(R[i,k], nR[i,k])   # binomial likelihood
    R[i,k] <- phi.adj[i,k]
    x[i,k] <- -(q[i]*yBR[i,k]+ phi[i+nsCFB+nsBF,k])/(sdBR[i,k] *
sqrt(1+(1-q[i])*(1-q[i]-2*rho)))
    # adjust link function phi(x) for extreme values that can give
numerical
    # errors when x< -5, phi(x)=0, when x> 5, phi(x)=1
    phi.adj[i,k] <- (step(5+x[i,k]) * step(x[i,k]-5)
           + step(5-x[i,k])* step(x[i,k]+5) * phi(x[i,k]))*(1-
equals(x[i,k],5))
           + equals(x[i,k],5)   # correct for x=5
    # theta is standardised mean
    phi[i+nsCFB+nsBF,k] <- theta[i+nsCFB+nsBF,k]
                  * (Pooled.sd[i+nsCFB+nsBF]/H[i+nsCFB+nsBF])
    # model for linear predictor, delta is SMD
    theta[i+nsCFB+nsBF,k] <- mu[i+nsCFB+nsBF] +
delta[i+nsCFB+nsBF,k] + (beta[i+nsCFB+nsBF,k]*V[i+nsCFB+nsBF,k])
    # residual deviance contribution
    rhat[i,k] <- R[i,k] * nR[i,k]
    dev[i+nsCFB+nsBF,k] <- 2 * (r[i,k] * (log(r[i,k])-
log(rhat[i,k]))
          + (nR[i,k]-r[i,k]) * (log(nR[i,k]-r[i,k]) - log(nR[i,k]-
rhat[i,k])))
    sdR[i,k] <-  4.46 + (sdBR[i,k] * 0.55) # sd for response in
severe group
           # Variance of baseline for pooled SD
    nvarR[i,k] <- (nR[i,k]-1) * pow(sdBR[i,k],2) # for pooled.sd
#   # nvarR[i,k] <- (nR[i,k]-1) * pow(sdR[i,k],2) # for pooled.sd
   }
  # summed residual deviance contribution for this trial
  resdev[i+nsCFB+nsBF] <- sum(dev[i+nsCFB+nsBF,1:naR[i]])
 }
#
# RE MODEL (CFB data)
for(i in 1:nsCFB){                         # LOOP THROUGH STUDIES WITH
CFB DATA
  for (k in 2:naCFB[i]){                   # LOOP THROUGH ARMS
        # model for bias parameter beta
    beta[i,k] ~ dnorm(mb[i,k], Pkappa)
```

```
    mb[i,k] <- A[CCFB[i,k]]
    V[i,k] <- (var[i,k]+var[i,1])/Pooled.var[i]
    # trial-specific RE distributions
    delta[i,k] ~ dnorm(md[i,k], taud[i,k])
    md[i,k] <- d[tCFB[i,k]] - d[tCFB[i,1]] + sw[i,k]
    # precision of RE distributions (with multi-arm trial
correction)
    taud[i,k] <- tau *2*(k-1)/k
    #adjustment, multi-arm RCTs
    w[i,k] <- delta[i,k] - d[tCFB[i,k]] + d[tCFB[i,1]]
    # cumulative adjustment for multi-arm trials
    sw[i,k] <-sum(w[i,1:k-1])/(k-1)
   }
 }
# RE MODEL (BL and F-up data)
for(i in 1:nsBF){                    # LOOP THROUGH STUDIES WITH
BL+FUP DATA
  for (k in 2:naBF[i]){                 # LOOP THROUGH ARMS
         # model for bias parameter beta
    beta[i+nsCFB,k] ~ dnorm(mb[i+nsCFB,k], Pkappa)
    mb[i+nsCFB,k] <- A[CBF[i,k]]
    V[i+nsCFB,k] <-
(var[i+nsCFB,k]+var[i+nsCFB,1])/Pooled.var[i+nsCFB]
    # trial-specific RE distributions
    delta[i+nsCFB,k] ~ dnorm(md[i+nsCFB,k], taud[i+nsCFB,k])
    md[i+nsCFB,k] <- d[tBF[i,k]] - d[tBF[i,1]] + sw[i+nsCFB,k]
    # precision of RE distributions (with multi-arm trial
correction)
    taud[i+nsCFB,k] <- tau *2*(k-1)/k
    #adjustment, multi-arm RCTs
    w[i+nsCFB,k] <- delta[i+nsCFB,k] - d[tBF[i,k]] + d[tBF[i,1]]
    # cumulative adjustment for multi-arm trials
    sw[i+nsCFB,k] <-sum(w[i+nsCFB,1:k-1])/(k-1)
   }
 }
# RE MODEL (Response data)
for(i in 1:nsR){                     # LOOP THROUGH STUDIES WITH
RESPONSE DATA
  for (k in 2:naR[i]){               # LOOP THROUGH ARMS
    # model for bias parameter beta
    beta[i+nsCFB+nsBF,k] ~ dnorm(mb[i+nsCFB+nsBF,k], Pkappa)
    mb[i+nsCFB+nsBF,k] <- A[C[i,k]]
    #
    # calculate variance of log odds ratio for comparisons with arm
1
    # check for zero or 100% events in arm k
    aux.a[i,k] <- equals(r[i,k],0)+equals(r[i,k],nR[i,k])
    # check for zero or 100% events in arm 1
    aux.b[i,k] <- equals(r[i,1],0)+equals(r[i,1],nR[i,1])
    aux[i,k] <- max(aux.a[i,k],aux.b[i,k]) # any zero or 100%
events?
    # add 0.5 if zero or 100% events
    VLOR[i,k] <- 1/(r[i,k]+(0.5*aux[i,k])) +
1/(r[i,1]+(0.5*aux[i,k]))                          + 1/(nR[i,k]-
```

```
r[i,k]+(0.5*aux[i,k]))
+ 1/(nR[i,1]-r[i,1]+(0.5*aux[i,k]))
    V[i+nsCFB+nsBF,k] <- 0.30396 * VLOR[i,k]  # convert to var of
SMD
    # trial-specific RE distributions
    delta[i+nsCFB+nsBF,k] ~ dnorm(md[i+nsCFB+nsBF,k],
taud[i+nsCFB+nsBF,k])
    md[i+nsCFB+nsBF,k] <- d[tR[i,k]] - d[tR[i,1]] +
sw[i+nsCFB+nsBF,k]
    # precision of RE distributions (with multi-arm trial
correction)
    taud[i+nsCFB+nsBF,k] <- tau *2*(k-1)/k
    #adjustment, multi-arm RCTs
    w[i+nsCFB+nsBF,k] <- delta[i+nsCFB+nsBF,k] - d[tR[i,k]] +
d[tR[i,1]]
    # cumulative adjustment for multi-arm trials
    sw[i+nsCFB+nsBF,k] <-sum(w[i+nsCFB+nsBF,1:k-1])/(k-1)
  }
 }
#
totresdev <- sum(resdev[])              # Total Residual Deviance
(all data)
# Partial Residual Deviance
totresdev.p[1] <- sum(resdev[1:nsCFB])                    # CFB
data
totresdev.p[2] <- sum(resdev[nsCFB+1:nsCFB+nsBF])         # BL +
Fup data
totresdev.p[3] <- sum(resdev[nsCFB+nsBF+1:nsCFB+nsBF+nsR]) #
Response data
#
# Priors and model assumptions (classes)
d[1] <- 0                    # treatment effect is zero for reference
treatment
# treatment effects from Class
for (k in 2:7) {
  d[k] <- m[D[k]]
}
for (k in 8:nt) {
  d[k] ~ dnorm(m[D[k]], prec2[D[k]])
}
for (k in 8:nc) {
  prec2[k] <- pow(sd2[k],-2)
}
for (k in 1:ncvar) {
  sd2[cvar[k]] ~ dunif(0,5)
}

sd2[8] <- sd2[9]
sd2[10] <- sd2[9]
sd2[11] <- sd2[9]
sd2[12] <- sd2[9]
sd2[13] <- sd2[9]
sd2[14] <- sd2[9]
sd2[15] <- sd2[9]
sd2[18] <- sd2[9]
```

```
sd2[19] <- sd2[10]
sd2[20] <- sd2[10]
sd2[21] <- sd2[10]
sd2[23] <- sd2[9]
sd2[24] <- sd2[9]
sd2[25] <- sd2[9]
sd2[26] <- sd2[9]
sd2[29] <- sd2[27]
sd2[31] <- sd2[32]
sd2[32] <- sd2[9]
sd2[34] <- sd2[17]
sd2[35] <- sd2[10]
sd2[36] <- sd2[32]
sd2[37] <- sd2[38]
sd2[39] <- sd2[38]
sd2[40] <- sd2[38]
sd2[41] <- sd2[38]
sd2[42] <- sd2[38]
sd2[43] <- sd2[38]
sd2[44] <- sd2[38]
sd2[45] <- sd2[38]
sd2[46] <- sd2[38]
sd2[47] <- sd2[38]
sd2[48] <- sd2[38]
sd2[50] <- sd2[32]
sd2[22] <- max(sd2[9], sd2[10])
sd2[30] <- max(sd2[27], sd2[28])


m[1] <- 0
# prior for mean class effect
for (k in 2:nc){  m[k] ~ dnorm(0, .0001)   }

#
sdev ~ dunif(0,5)                    # vague prior for between-
trial SD
tau <- pow(sdev,-2)                   # between-trial precision

# mean bias: assumptions
A[1] <- 0             # control v control
A[2] <- b             # control v Active
A[3] <- 0             # Active v Active
# bias model prior for variance
kappa ~ dunif(0,50)
kappa.sq <- pow(kappa,2)
Pkappa <- 1/kappa.sq
# bias model prior for mean
b ~ dnorm(0,.0001)

# all pairwise differences
for (c in 1:(nt-1)) {  for (k in (c+1):nt)  { diff[c,k] <- d[k] -
d[c] }   }
#diff[2,1] <- d[1]-d[2]                 #Get diff relative to
waitlist
#Desired treatment effects (relative to waitlist)
```

```
#tx.out[2]<-diff[2,1]
for(k in 3:nt){ tx.out[k]<-diff[2,k] }
# pairwise SMDs for all possible class comparisons
for (c in 1:(nc-1)){
  for (k in (c+1):nc){ diffClass[c,k] <- (m[k]-m[c]) }
 }
diffClass[2,1] <- m[1]-m[2]           #Get diffs relative to no
treatment
#Desired class effects (relative to no treatment)
#class.out[2]<-diffClass[2,1]
#for(k in 3:nc){ class.out[k]<-diffClass[2,k] }
#
# treatments of interest to rank
for (k in 1:ntR) {
      dR[k] <- d[tRcode[k]]
}
# classes of interest to rank
for (k in 1:ncR) {
      mR[k] <- m[cRcode[k]]
}

for (k in 1:ntR){
#  rk2[k] <- ntR+1-rank(dR[],k)   # lower values are "bad"
  rk[k] <- rank(dR[],k)            # lower values are "good"
  best[k]  <- equals(rk[k],1)  # Smallest is best (i.e. rank 1)
  # prob treat k is h-th best, prob[1,k]=best[k]
  for (h in 1:ntR) { prob[h,k] <- equals(rk[k],h) }
 }
#
for (k in 1:ncR){
  rkClass[k]  <- rank(mR[],k)   # lower values are "good"
  bestClass[k]  <- equals(rkClass[k],1)    # Smallest is best (i.e.
rank 1)
  # prob class k is h-th best, prob[1,k]=best[k]
  for (h in 1:ncR) { probClass[h,k] <- equals(rkClass[k],h) }
 }
}                                # *** PROGRAM ENDS
```

Sample OpenBUGS code – response bias analysis

```
 # Random effects model for multi-arm trials
model{                                         # *** PROGRAM STARTS
for(i in 1:ns){                                #   LOOP THROUGH STUDIES
  w[i,1] <- 0   # adjustment for multi-arm trials is zero for
control arm
      beta[i,1] <- 0                 # no bias term in baseline
arm
  V[i,1] <- 0                    # no variance term in baseline
arm
  delta[i,1] <- 0                    # treatment effect is zero for
control arm
      deltaX[i,1] <- 0                                # treatment
effect is zero for control arm
  mu[i] ~ dnorm(0,.0001)             # vague priors for all trial
baselines
```

```
      muX[i] ~ dnorm(0,.0001)                           # vague
priors for all trial baselines
  }
# (1) Response DATA
for(i in 1:nsR){
  for (k in 1:naR[i]){
    r[i,k] ~ dbin(p[i,k],nR[i,k])       # binomial likelihood
    logit(p[i,k]) <- mu[i] + delta[i,k] + (beta[i,k]*V[i,k]) #
model for linear predictor
    rhat[i,k] <- p[i,k] * nR[i,k]       # expected value of the
numerators
    #Deviance contribution
    dev[i,k] <- 2 * (r[i,k] * (log(r[i,k])-log(rhat[i,k]))
          +  (nR[i,k]-r[i,k]) * (log(nR[i,k]-r[i,k]) - log(nR[i,k]-
rhat[i,k])))
   }
#Summed residual deviance contribution for this trial
  resdev[i] <- sum(dev[i,1:naR[i]])
  }
# (2) CFB DATA
for(i in 1:nsCFB){
  # calculate pooled.sd and adjustment for SMD
  df[i] <- sum(nCFB[i,1:naCFB[i]]) - naCFB[i] # denominator for
pooled.var
  Pooled.var[i] <- sum(nvar[i,1:naCFB[i]])/df[i]
      # pooled sd for study i, for SMD
  Pooled.sd[i] <- sqrt(Pooled.var[i])
#  H[i] <- 1 - 3/(4*df[i]-1)          # use Hedges' g
  H[i] <- 1                          # use Cohen's d (ie no
adjustment)
  for (k in 1:naCFB[i]){
    se[i,k] <- sdCFB[i,k]/sqrt(nCFB[i,k])
    var[i,k] <- pow(se[i,k],2)       # calcultate variances
    prec[i,k] <- 1/var[i,k]          # set precisions
    yCFB[i,k] ~ dnorm(phi[i,k], prec[i,k]) # normal likelihood
    phi[i,k] <- theta[i,k] * (Pooled.sd[i]/H[i]) # theta is SMD
    theta[i,k] <- muX[i] + deltaX[i,k] # model for linear predictor
    dev[i+nsR,k] <- (yCFB[i,k]-phi[i,k])*(yCFB[i,k]-
phi[i,k])*prec[i,k]
    nvar[i,k] <- (nCFB[i,k]-1) * pow(sdCFB[i,k],2) # for pooled.sd
   }
  # summed residual deviance contribution for this trial
  resdev[i+nsR] <- sum(dev[i+nsR,1:naCFB[i]])
  }
# (3) BASELINE + FOLLOW-UP DATA (no CFB)
for(i in 1:nsBF){                    #  LOOP THROUGH STUDIES
  # calculate pooled.sd and adjustment for SMD
  df[i+nsCFB] <- sum(n[i,1:naBF[i]]) - naBF[i] # denominator for
pooled.var
  Pooled.var[i+nsCFB] <- sum(nvarBF[i,1:naBF[i]])/df[i+nsCFB]
      # pooled sd for study i, for SMD
  Pooled.sd[i+nsCFB] <- sqrt(Pooled.var[i+nsCFB])
#  H[i] <- 1 - 3/(4*df[i]-1)           # use Hedges' g
  H[i+nsCFB] <- 1                     # use Cohen's d (ie no
adjustment)
```

```
   for (k in 1:naBF[i]){
      yBF[i,k] <- yF[i,k] - yB[i,k]      # calculate mean CFB
     seF[i,k] <- sdF[i,k]/sqrt(n[i,k]) # se at followup
     seB[i,k] <- sdB[i,k]/sqrt(n[i,k]) # se at baseline
# variance of mean CFB, assuming correlation corr[i]
     var[i+nsCFB,k] <- pow(seF[i,k],2)+ pow(seB[i,k],2)
-2*(seF[i,k]*seB[i,k]*rho)
     prec[i+nsCFB,k] <- 1/var[i+nsCFB,k] # set CFB precisions
     yBF[i,k] ~ dnorm(phi[i+nsCFB,k], prec[i+nsCFB,k]) # normal
likelihood
# theta is SMD
     phi[i+nsCFB,k] <- theta[i+nsCFB,k] *
(Pooled.sd[i+nsCFB]/H[i+nsCFB])
# model for linear predictor
     theta[i+nsCFB,k] <- muX[i+nsCFB] + deltaX[i+nsCFB,k]
# residual deviance contribution
     dev[i+nsR+nsCFB,k] <- (yBF[i,k]-phi[i+nsCFB,k]) * (yBF[i,k]-
phi[i+nsCFB,k])                             * prec[i+nsCFB,k]
     # variance of CFB, assuming correlation corrBF[i] (var is sd
squared)
     #varBF[i,k] <- pow(sdF[i,k],2) + pow(sdB[i,k],2)
#             - 2*(sdF[i,k]*sdB[i,k]*rho)
           # Variance of baseline for pooled SD
           varBF[i,k] <- pow(sdB[i,k],2)
     nvarBF[i,k] <- (n[i,k]-1) * varBF[i,k] # for pooled.sd
    }
   # summed residual deviance contribution for this trial
   resdev[i+nsR+nsCFB] <- sum(dev[i+nsR+nsCFB,1:naBF[i]])
  }
# RE MODEL (Response data)
for(i in 1:nsR){                       # LOOP THROUGH STUDIES WITH
RESPONSE DATA
   for (k in 2:naR[i]){                # LOOP THROUGH ARMS
           # calculate variance of log odds ratio for comparisons
with arm 1
     # check for zero or 100% events in arm k
     aux.a[i,k] <- equals(r[i,k],0)+equals(r[i,k],nR[i,k])
     # check for zero or 100% events in arm 1
     aux.b[i,k] <- equals(r[i,1],0)+equals(r[i,1],nR[i,1])
     aux[i,k] <- max(aux.a[i,k],aux.b[i,k]) # any zero or 100%
events?
     # add 0.5 if zero or 100% events
     V[i,k] <- 1/(r[i,k]+(0.5*aux[i,k])) + 1/(r[i,1]+(0.5*aux[i,k]))
+ 1/(nR[i,k]-r[i,k]+(0.5*aux[i,k]))
+ 1/(nR[i,1]-r[i,1]+(0.5*aux[i,k]))
     # model for bias parameter beta
     beta[i,k] ~ dnorm(mb[i,k], Pkappa)
     mb[i,k] <- A[C[i,k]]

     # trial-specific RE distributions
     delta[i,k] ~ dnorm(md[i,k], taud[i,k])
     md[i,k] <- d[tR[i,k]] - d[tR[i,1]] + sw[i,k]
     # precision of RE distributions (with multi-arm trial
correction)
     taud[i,k] <- tau *2*(k-1)/k
```

```
    #adjustment, multi-arm RCTs
    w[i,k] <- delta[i,k] - d[tR[i,k]] + d[tR[i,1]]
    # cumulative adjustment for multi-arm trials
    sw[i,k] <-sum(w[i,1:k-1])/(k-1)
  }
 }
# RE MODEL (CFB data)
for(i in 1:nsCFB){                      # LOOP THROUGH STUDIES WITH
CFB DATA
  for (k in 2:naCFB[i]){                # LOOP THROUGH ARMS
    # convert SMD to LOR
    deltaX[i,k] <-
(delta[i+nsR,k]+beta[i+nsR,k]*V[i+nsR,k])*((sqrt(3))/-3.1416)

           # convert variance of SMD to variance of LOR for bias
model
    VSMD[i,k] <- (var[i,k]+var[i,1])/Pooled.var[i]
    V[i+nsR,k] <- 3.2899 * VSMD[i,k]
    # model for bias parameter beta
    beta[i+nsR,k] ~ dnorm(mb[i+nsR,k], Pkappa)
    mb[i+nsR,k] <- A[CCFB[i,k]]

           # trial-specific RE distributions
    delta[i+nsR,k] ~ dnorm(md[i+nsR,k], taud[i+nsR,k])
    md[i+nsR,k] <- d[tCFB[i,k]] - d[tCFB[i,1]] + sw[i+nsR,k]
    # precision of RE distributions (with multi-arm trial
correction)
    taud[i+nsR,k] <- tau *2*(k-1)/k
    #adjustment, multi-arm RCTs
    w[i+nsR,k] <- delta[i+nsR,k] - d[tCFB[i,k]] + d[tCFB[i,1]]
    # cumulative adjustment for multi-arm trials
    sw[i+nsR,k] <-sum(w[i+nsR,1:k-1])/(k-1)
   }
 }
# RE MODEL (BL and F-up data)
for(i in 1:nsBF){                       # LOOP THROUGH STUDIES WITH
BL+FUP DATA
  for (k in 2:naBF[i]){                   # LOOP THROUGH ARMS
    # convert SMD to LOR
    deltaX[i+nsCFB,k] <-
(delta[i+nsR+nsCFB,k]+beta[i+nsR+nsCFB,k]*V[i+nsR+nsCFB,k]) *
((sqrt(3))/-3.1416)

    # convert variance of SMD to variance of LOR for bias model
    VSMD[i+nsCFB,k] <-
(var[i+nsCFB,k]+var[i+nsCFB,1])/Pooled.var[i+nsCFB]
    V[i+nsR+nsCFB,k] <- 3.2899 * VSMD[i+nsCFB,k]
    # model for bias parameter beta
    beta[i+nsR+nsCFB,k] ~ dnorm(mb[i+nsR+nsCFB,k], Pkappa)
    mb[i+nsR+nsCFB,k] <- A[CBF[i,k]]

           # trial-specific RE distributions
    delta[i+nsR+nsCFB,k] ~ dnorm(md[i+nsR+nsCFB,k],
taud[i+nsR+nsCFB,k])
```

```
    md[i+nsR+nsCFB,k] <- d[tBF[i,k]] - d[tBF[i,1]] +
sw[i+nsR+nsCFB,k]
    # precision of RE distributions (with multi-arm trial
correction)
    taud[i+nsR+nsCFB,k] <- tau *2*(k-1)/k
    #adjustment, multi-arm RCTs
    w[i+nsR+nsCFB,k] <- delta[i+nsR+nsCFB,k] - d[tBF[i,k]] +
d[tBF[i,1]]
    # cumulative adjustment for multi-arm trials
    sw[i+nsR+nsCFB,k] <-sum(w[i+nsR+nsCFB,1:k-1])/(k-1)
   }
 }
#
totresdev <- sum(resdev[])              # Total Residual Deviance
(all data)
# Partial Residual Deviance
totresdev.p[1] <- sum(resdev[1:nsR])                     # Response
data
totresdev.p[2] <- sum(resdev[nsR+1:nsR+nsCFB])          # CFB data
totresdev.p[3] <- sum(resdev[nsR+nsCFB+1:nsR+nsCFB+nsBF]) #
Response data
#
# Priors and model assumptions (classes)
d[1] <- 0                # treatment effect is zero for reference
treatment
# treatment effects from Class
for (k in 2:7) {
  d[k] <- m[D[k]]
}
for (k in 8:nt) {
  d[k] ~ dnorm(m[D[k]], prec2[D[k]])
}
for (k in 8:nc) {
  prec2[k] <- pow(sd2[k],-2)
}
for (k in 1:ncvar) {
  sd2[cvar[k]] ~ dunif(0,7)
}

sd2[8] <- sd2[9]
sd2[10] <- sd2[9]
sd2[11] <- sd2[9]
sd2[12] <- sd2[9]
sd2[13] <- sd2[9]
sd2[14] <- sd2[9]
sd2[15] <- sd2[9]
sd2[18] <- sd2[9]
sd2[19] <- sd2[10]
sd2[21] <- sd2[9]
sd2[22] <- sd2[9]
sd2[23] <- sd2[9]
sd2[25] <- sd2[24]
sd2[26] <- sd2[24]
sd2[28] <- sd2[29]
sd2[29] <- sd2[9]
```

```
sd2[30] <- sd2[17]
sd2[31] <- sd2[17]
sd2[32] <- sd2[10]
sd2[33] <- sd2[29]
sd2[34] <- sd2[35]
sd2[36] <- sd2[35]
sd2[37] <- sd2[35]
sd2[38] <- sd2[35]
sd2[39] <- sd2[35]
sd2[40] <- sd2[35]
sd2[41] <- sd2[35]
sd2[42] <- sd2[35]
sd2[43] <- sd2[29]
sd2[44] <- sd2[29]
sd2[20] <- max(sd2[9], sd2[10])
sd2[27] <- max(sd2[24], sd2[25])


m[1] <- 0
# prior for mean class effect
for (k in 2:nc){  m[k] ~ dnorm(0, .0001)  }

#
sdev ~ dunif(0,5)                    # vague prior for between-
trial SD
tau <- pow(sdev,-2)                   # between-trial precision

# mean bias: assumptions
A[1] <- 0              # control v control
A[2] <- b              # control v Active
A[3] <- 0              # Active v Active
# bias model prior for variance
kappa ~ dunif(0,5)
kappa.sq <- pow(kappa,2)
Pkappa <- 1/kappa.sq
# bias model prior for mean
b ~ dnorm(0,.0001)

# all pairwise differences
for (c in 1:(nt-1)) {
     for (k in (c+1):nt)  {
          or[c,k] <- exp(d[k] - d[c])
    lor[c,k] <- (d[k]-d[c])
          }
     }
#diff[2,1] <- d[1]-d[2]                #Get diff relative to
waitlist
#Desired treatment effects (relative to waitlist)
#tx.out[2]<-diff[2,1]
#for(k in 3:nt){ tx.out[k]<-diff[2,k] }
# pairwise SMDs for all possible class comparisons
for (c in 1:(nc-1)){
  for (k in (c+1):nc){
          lorClass[c,k] <- m[k] - m[c]
    orClass[c,k] <- exp(m[k] - m[c])
```

```
            }
 }
#diffClass[2,1] <- m[1]-m[2]                #Get diffs relative to
no treatment
#Desired class effects (relative to no treatment)
#class.out[2]<-diffClass[2,1]
#for(k in 3:nc){ class.out[k]<-diffClass[2,k] }
#
# treatments of interest to rank
for (k in 1:ntR) {
     dR[k] <- d[tRcode[k]]
}
# classes of interest to rank
for (k in 1:ncR) {
     mR[k] <- m[cRcode[k]]
}

for (k in 1:ntR){
  rk[k] <- ntR+1-rank(dR[],k)     # lower values are "bad"
#  rk[k] <- rank(dR[],k)           # lower values are "good"
  best[k]  <- equals(rk[k],1)  # Smallest is best (i.e. rank 1)
  # prob treat k is h-th best, prob[1,k]=best[k]
  for (h in 1:ntR) { prob[h,k] <- equals(rk[k],h) }
 }
#
for (k in 1:ncR){
  rkClass[k]  <- ncR+1-rank(mR[],k)   # lower values are "bad"
#     rkClass[k]  <- rank(mR[],k)   # lower values are "good"
  bestClass[k]  <- equals(rkClass[k],1)    # Smallest is best (i.e.
rank 1)
  # prob class k is h-th best, prob[1,k]=best[k]
  for (h in 1:ncR) { probClass[h,k] <- equals(rkClass[k],h) }
 }
}                                 # *** PROGRAM ENDS
```

Sample OpenBUGS code – discontinuation bias analysis

```
 model{
for(i in 1:ns){
     beta[i,1] <- 0                    # no bias term in baseline
arm
  V[i,1] <- 0                       # no variance term in baseline
arm
  w[i,1] <- 0                     # adjustment for multi-arm trials
is zero for control arm
  delta[i,1] <- 0    # treatment effect is zero for control arm
  mu[i] ~ dnorm(0,.0001) # vague priors for all trial baselines
  for (k in 1:na[i]) {
    r[i,k] ~ dbin(p[i,k],n[i,k])                                #
binomial likelihood
    logit(p[i,k]) <- mu[i] + delta[i,k] + (beta[i,k]*V[i,k])
      # model for linear predictor
    rhat[i,k] <- p[i,k] * n[i,k]                                #
expected value of the numerators
#Deviance contribution
```

```
    dev[i,k] <- 2 * (r[i,k] * (log(r[i,k])-log(rhat[i,k]))
          +  (n[i,k]-r[i,k]) * (log(n[i,k]-r[i,k]) - log(n[i,k]-
rhat[i,k])))
   }
#Summed residual deviance contribution for this trial
  resdev[i] <- sum(dev[i,1:na[i]])
  for (k in 2:na[i]) {
           # calculate variance of log odds ratio for comparisons
with arm 1
    # check for zero or 100% events in arm k
    aux.a[i,k] <- equals(r[i,k],0)+equals(r[i,k],n[i,k])
    # check for zero or 100% events in arm 1
    aux.b[i,k] <- equals(r[i,1],0)+equals(r[i,1],n[i,1])
    aux[i,k] <- max(aux.a[i,k],aux.b[i,k]) # any zero or 100%
events?
    # add 0.5 if zero or 100% events
    V[i,k] <- 1/(r[i,k]+(0.5*aux[i,k])) + 1/(r[i,1]+(0.5*aux[i,k]))
+ 1/(n[i,k]-r[i,k]+(0.5*aux[i,k]))
+ 1/(n[i,1]-r[i,1]+(0.5*aux[i,k]))
    # model for bias parameter beta
    beta[i,k] ~ dnorm(mb[i,k], Pkappa)
    mb[i,k] <- A[C[i,k]]

    delta[i,k] ~ dnorm(md[i,k],taud[i,k])         # trial-specific
LOR distributions
    # mean of LOR distributions (with multi-arm trial correction)
    md[i,k] <-  d[t[i,k]] - d[t[i,1]] + sw[i,k]
    # precision of LOR distributions (with multi-arm trial
correction)
    taud[i,k] <- tau *2*(k-1)/k
    # adjustment for multi-arm RCTs
    w[i,k] <- (delta[i,k] - d[t[i,k]] + d[t[i,1]])
    # cumulative adjustment for multi-arm trials
    sw[i,k] <- sum(w[i,1:k-1])/(k-1)
   }
 }
totresdev <- sum(resdev[])           # Total Residual Deviance
d[1]<-0       # treatment effect is zero for reference treatment
# treatment effects from Class
#for (k in 2:nt){ d[k] ~ dnorm(m[D[k]], prec2[D[k]]) }
for (k in 2:8) {
  d[k] <- m[D[k]]
}
for (k in 9:nt) {
  d[k] ~ dnorm(m[D[k]], prec2[D[k]])
}
for (k in 9:nc) {
  prec2[k] <- pow(sd2[k],-2)
}
for (k in 1:ncvar) {
  sd2[cvar[k]] ~ dunif(0,5)
}

sd2[9] <- sd2[11]
sd2[10] <- sd2[11]
```

```
sd2[12] <- sd2[11]
sd2[13] <- sd2[11]
sd2[14] <- sd2[11]
sd2[15] <- sd2[11]
sd2[16] <- sd2[11]
sd2[19] <- sd2[11]
sd2[20] <- sd2[11]
sd2[21] <- sd2[11]
sd2[22] <- sd2[11]
sd2[25] <- sd2[23]
sd2[26] <- sd2[27]
sd2[27] <- sd2[11]
sd2[28] <- sd2[18]
sd2[29] <- sd2[18]
sd2[30] <- sd2[12]
sd2[31] <- sd2[27]
sd2[33] <- sd2[32]
sd2[34] <- sd2[32]
sd2[35] <- sd2[32]
sd2[36] <- sd2[32]
sd2[37] <- sd2[32]
sd2[38] <- sd2[27]
sd2[39] <- sd2[27]

m[1] <- 0
#m[4] <- d[10]
# prior for mean class effect
for (k in 2:nc){  m[k] ~ dnorm(0, .0001)   }

#
sd ~ dunif(0,5)      # vague prior for between-trial SD
tau <- pow(sd,-2)    # between-trial precision = (1/between-trial
variance)

# mean bias: assumptions
A[1] <- 0               # control v control
A[2] <- b               # control v Active
A[3] <- 0               # Active v Active
# bias model prior for variance
kappa ~ dunif(0,5)
kappa.sq <- pow(kappa,2)
Pkappa <- 1/kappa.sq
# bias model prior for mean
b ~ dnorm(0,.0001)

# pairwise ORs and LORs for all possible pair-wise comparisons
for (c in 1:(nt-1)){
  for (k in (c+1):nt){
    or[c,k] <- exp(d[k] - d[c])
    lor[c,k] <- (d[k]-d[c])
   }
 }
# treatments of interest to rank
for (k in 1:ntR) {
      dR[k] <- d[tRcode[k]]
```

```
}
# classes of interest to rank
for (k in 1:ncR) {
      mR[k] <- m[cRcode[k]]
}
for (k in 1:ntR){
  rk[k] <- rank(dR[],k)                     # assumes events are
"bad"
  best[k] <- equals(rk[k],1)                           #calculate
probability that treat k is best
  # calculates probability that treat k is h-th best
  for (h in 1:ntR){ prob[h,k] <- equals(rk[k],h) }
 }
#
# pairwise differences for classes
for (c in 1:(nc-1)){
  for (k in (c+1):nc){
    lorClass[c,k] <- m[k] - m[c]
    orClass[c,k] <- exp(m[k] - m[c])
   }
 }
# rank classes
for (k in 1:ncR)  {
    rkClass[k]  <- rank(mR[],k)
    bestClass[k]  <- equals(rkClass[k],1)    # Smallest is best
(i.e. rank 1)
# prob class k is h-th best, prob[1,k]=best[k]
    for (h in 1:ncR) { probClass[h,k] <- equals(rkClass[k],h) }
    }
}

}                                # *** PROGRAM ENDS
```

References

1. Ferster CB. A functional anlysis of depression. *Am Psychol* 1973; **28**(10): 857-70.
2. Lewinsohn PM. Activity schedules in the treatment of depression. In: Krumboltz JD, Thoresen CE, eds. Counselling methods. New York: Holt, Rine-hart & Winston; 1976: 74-83.
3. Lewinsohn PM, Antonuccio DO, Steinmetz JL, Teri L. The coping with depression course: A psychoeducational intervention for unipolar depression: Castalia Pub Co; 1984.
4. Beck AT, Rush AJ, Shaw BF, Emery G. Cognitive therapy of depression. New York: Guilford Press; 1979.
5. Ellis A. New approaches to psychotherapy techniques. *J Clin Psychol* 1955; **11**(3): 207-60.
6. Seligman MEP, Rashid T, Parks AC. Positive psychotherapy. *Am Psychol* 2006; **61**(8): 774-88.
7. Nezu AM. A problem-solving formulation of depression: A literature review and proposal of a pluralistic model. *Clinical Psychology Review* 1987; **7**(2): 121-44.
8. Watkins ER. Constructive and unconstructive repetitive thought. *Psychol Bull* 2008; **134**(2): 163-206.
9. Nezu AM, Nezu CM, Perri MG. Problem-solving therapy for depression: Theory, research, and clinical guidelines: John Wiley & Sons; 1989.
10. Rogers CR. The necessary and sufficient conditions of therapeutic personality change. *J Consult Psychol* 1957; **21**(2): 95-103.
11. Truax CB, Carkhuff R. Toward effective counseling and psychotherapy: Training and practice. New Brunswick and London: Transaction Publishers; 1967.
12. Egan G. The skilled helper: A systematic approach to effective helping (4th ed.) Thomson Brooks/Cole Publishing Co.; 1990.
13. Roth A, Fonagy P. What works for whom: A critical review of psychotherapy research (2nd ed.). : Guilford Publications; 2005.
14. Bower P, Rowland N, Hardy R. The clinical effectiveness of counselling in primary care: a systematic review and meta-analysis. *Psychol Med* 2003; **33**(2): 203-15.
15. Klerman GL, Weissman MM, Rounsaville BJ, Chevron ES. Interpersonal psychotherapy of depression. : Basic Books; 1984.
16. Weissman MM, Markowitz JC, Klerman GL. Comprehensive guide to interpersonal psychotherapy: Basic Books; 2000.
17. Klerman GL, Budman S, Berwick D, et al. Efficacy of a brief psychosocial intervention for symptoms of stress and distress among patients in primary care. *Med Care* 1987; **25**(11): 1078-88.
18. Greenberg JR, Mitchell SA. Object Relations in Psychoanalytic Theory. Cambridge, MA: Harvard University Press; 1983.
19. Holmes J. The search for the secure base: attachment theory and psychotherapy. London: Brunner-Routledge, Taylor & Francis; 2001.
20. Lemma A, Target M, Fonagy P. Brief dynamic interpersonal therapy: A clinician's guide. Oxford: Oxford University Press; 2011.
21. Covi L, Lipman RS. Cognitive behavioral group psychotherapy combined with imipramine in major depression. *Psychopharmacol Bull* 1987; **23**(1): 173-6.
22. Gabbard GO. Long-term psychodynamic psychotherapy: A basic text (3rd edition): American Psychiatric Pub; 2017.
23. Maratos A, Crawford MJ, Procter S. Music therapy for depression: it seems to work, but how? *Br J Psychiatry* 2011; **199**(2): 92-3.
24. Prescribing & Medicines Team, Health and Social Care Information Centre. Prescription Cost Analysis: England 2016, 2017.
25. Smith CA, Armour M, Lee MS, Wang LQ, Hay PJ. Acupuncture for depression. *Cochrane Database Syst Rev* 2018; **3**(3): CD004046.
26. Napadow V, Makris N, Liu J, Kettner NW, Kwong KK, Hui KK. Effects of electroacupuncture versus manual acupuncture on the human brain as measured by fMRI. *Hum Brain Mapp* 2005; **24**(3): 193-205.

27. Thoren P, Floras JS, Hoffmann P, Seals DR. Endorphins and exercise: physiological mechanisms and clinical implications. *Med Sci Sports Exerc* 1990; **22**(4): 417-28.
28. Leith LM. Foundations of exercise and mental health. Morgantown (WV): Fitness Information Technology 1994.
29. Lam RW, Levitt AJ, Levitan RD, et al. Efficacy of Bright Light Treatment, Fluoxetine, and the Combination in Patients With Nonseasonal Major Depressive Disorder: A Randomized Clinical Trial. *JAMA Psychiatry* 2016; **73**(1): 56-63.
30. Kabat-Zinn J. Full catastrophe living: using the wisdom of your body and mind to face stress, pain, and illness. New York: Delacorte Press; 1990.
31. Segal ZV, Williams JMG, Teasdale JD. Mindfulness-based cognitive therapy for depression: a new approach to preventing relapse: Guilford Press; 2002.
32. Kuyken W, Byford S, Taylor RS, et al. Mindfulness-based cognitive therapy to prevent relapse in recurrent depression. *J Consult Clin Psychol* 2008; **76**(6): 966-78.
33. Kuyken W, Hayes R, Barrett B, et al. Effectiveness and cost-effectiveness of mindfulness-based cognitive therapy compared with maintenance antidepressant treatment in the prevention of depressive relapse or recurrence (PREVENT): a randomised controlled trial. *Lancet* 2015; **386**(9988): 63-73.
34. Hamilton M. A rating scale for depression. *J Neurol Neurosurg Psychiatry* 1960; **23**(1): 56-62.
35. Montgomery SA. Clinically relevant effect sizes in depression. *European Neuropsychopharmacology* 1994; **4**(3): 283-4.
36. Muller MJ, Himmerich H, Kienzle B, Szegedi A. Differentiating moderate and severe depression using the Montgomery-Asberg depression rating scale (MADRS). *J Affect Disord* 2003; **77**(3): 255-60.
37. Muller-Thomsen T, Arlt S, Mann U, Mass R, Ganzer S. Detecting depression in Alzheimer's disease: evaluation of four different scales. *Arch Clin Neuropsychol* 2005; **20**(2): 271-6.
38. Kroenke K, Spitzer RL. The PHQ-9: A new depression diagnostic and severity measure. *Psychiatric Annals* 2002; **32**(9).
39. Beck AT, Steer RA, Garbin MG. Psychometric properties of the Beck Depression Inventory Twenty-five years of evaluation. *Clinical Psychology Review* 1988; **8**: 77-100.
40. Beck AT, Steer RA, Brown GK. BDI-II: Beck Depression Inventory Manual. San Antonio: Psychological Corporation, 1996.
41. Radloff LS. The CES-D scale: A self report depression scale for research in the general population. *Applied Psychological Measurement* 1977; **1**(3): 385-401.
42. Reilly TJ, MacGillivray SA, Reid IC, Cameron IM. Psychometric properties of the 16-item Quick Inventory of Depressive Symptomatology: a systematic review and meta-analysis. *J Psychiatr Res* 2015; **60**: 132-40.
43. Zigmond AS, Snaith RP. The hospital anxiety and depression scale. *Acta Psychatr Scan* 1983; **67**(6): 361-70.
44. Cameron IM, Crawford JR, Lawton K, Reid IC. Psychometric comparison of PHQ-9 and HADS for measuring depression severity in primary care. *Br J Gen Pract* 2008; **58**(546): 32-6.
45. Carmody TJ, Rush AJ, Bernstein I, et al. The Montgomery Asberg and the Hamilton ratings of depression: a comparison of measures. *Eur Neuropsychopharmacol* 2006; **16**(8): 601-11.
46. Rush AJ, Trivedi MH, Ibrahim HM, et al. The 16-Item Quick Inventory of Depressive Symptomatology (QIDS), clinician rating (QIDS-C), and self-report (QIDS-SR): a psychometric evaluation in patients with chronic major depression. *Biol Psychiatry* 2003; **54**(5): 573-83.
47. Uher R, Farmer A, Maier W, et al. Measuring depression: comparison and integration of three scales in the GENDEP study. *Psychol Med* 2008; **38**(2): 289-300.
48. Wahl I, Lowe B, Bjorner JB, et al. Standardization of depression measurement: a common metric was developed for 11 self-report depression measures. *J Clin Epidemiol* 2014; **67**(1): 73-86.

49. Zimmerman M, Martinez JH, Young D, Chelminski I, Dalrymple K. Severity classification on the Hamilton Depression Rating Scale. *J Affect Disord* 2013; **150**(2): 384-8.
50. Interpreting the Beck Depression Inventory (BDI-II); 2013. Available from: https://drsarahallen.com/wp-content/uploads/2013/09/Beck-Depression-Inventory-and-Scoring-Key1.pdf. Accessed 28 November 2023.
51. Manea L, Gilbody S, McMillan D. Optimal cut-off score for diagnosing depression with the Patient Health Questionnaire (PHQ-9): a meta-analysis. *CMAJ* 2012; **184**(3): E191-6.
52. Lowe B, Spitzer RL, Grafe K, et al. Comparative validity of three screening questionnaires for DSM-IV depressive disorders and physicians' diagnoses. *J Affect Disord* 2004; **78**(2): 131-40.
53. Lunn D, Jackson C, Best N, Thomas A, Spiegelhalter D. The BUGS book. Boca Raton, FL: CRC Press.; 2013.
54. Lunn DJ, Thomas A, Best N, Spiegelhalter D. WinBUGS-A Bayesian modelling framework: Concepts, structure, and extensibility. . *Stat Comput* 2000; **10**: 325-37.
55. Spiegelhalter D, Thomas A, Best N, Lunn DJ. WinBUGS user manual: version 1.4. . Cambridge: MRC Biostatistics Unit; 2003.
56. Brooks S, Gelman A. General methods for monitoring convergence of iterative simulations. *J Comput Graph Stat* 1998; **7**(4): 434-55.
57. Gelman A, D.B. R. Inferences from iterative simulation using multiple sequences. *Stat Sci* 1992; **7**: 457-72.
58. Dias S, Sutton AJ, Ades AE, Welton NJ. Evidence synthesis for decision making 2: a generalized linear modeling framework for pairwise and network meta-analysis of randomized controlled trials. *Med Decis Making* 2013; **33**(5): 607-17.
59. Sweeting MJ, Sutton AJ, Lambert PC. What to add to nothing? Use and avoidance of continuity corrections in meta-analysis of sparse data. *Stat Med* 2004; **23**(9): 1351-75.
60. Dias S, Ades AE, Welton NJ, Jansen JP, Sutton AJ. Network Meta-analysis for Decision-Making. Hoboken, NJ: Wiley; 2018.
61. Spiegelhalter DJ, Best NG, Carlin BP, Van Der Linde A. Bayesian measures of model complexity and fit. *J R Stat Soc Ser B Stat Soc* 2002; **64**(1): 583-639.
62. Dempster A. The direct use of likelihood for significance testing. *Stat Comput* 1997; **7**: 247-52.
63. Daly C, Welton NJ, Dias S, Anwer S, Ades AE. Meta-Analysis of Continuous Outcomes. Guideline Methodology Document 2. Version 1. NICE Guidelines Technical Support Unit; 2021. Available from: http://www.bristol.ac.uk/media-library/sites/social-community-medicine/documents/mpes/gmd-2-continuous-jan2021.pdf Accessed 24 April 2022.
64. Cohen J. Statistical power analysis for the behavioral sciences. New York: Academic Press; 1969.
65. Higgins JPT, Thomas J, Chandler J, et al. Cochrane Handbook for Systematic Reviews of Interventions. 2nd Edition. Chichester (UK): John Wiley & Sons; 2019.
66. Chinn S. A simple method for converting an odds ratio to effect size for use in meta-analysis. *Stat Med* 2000; **19**(22): 3127-31.
67. Dias S, Welton NJ, Sutton AJ, Caldwell DM, Lu G, Ades AE. Evidence synthesis for decision making 4: inconsistency in networks of evidence based on randomized controlled trials. *Med Decis Making* 2013; **33**(5): 641-56.
68. van Valkenhoef G, Dias S, Ades AE, Welton NJ. Automated generation of node-splitting models for assessment of inconsistency in network meta-analysis. *Res Synth Methods* 2016; **7**(1): 80-93.
69. Driessen E, Hollon SD, Bockting CL, Cuijpers P, Turner EH. Does Publication Bias Inflate the Apparent Efficacy of Psychological Treatment for Major Depressive Disorder? A Systematic Review and Meta-Analysis of US National Institutes of Health-Funded Trials. *PLoS One* 2015; **10**(9): e0137864.

70. Moreno SG, Sutton AJ, Turner EH, et al. Novel methods to deal with publication biases: secondary analysis of antidepressant trials in the FDA trial registry database and related journal publications. *BMJ* 2009; **339**: b2981.
71. Moreno SG, Sutton AJ, Ades AE, Cooper NJ, Abrams KR. Adjusting for publication biases across similar interventions performed well when compared with gold standard data. *J Clin Epidemiol* 2011; **64**(11): 1230-41.
72. Trinquart L, Abbe A, Ravaud P. Impact of reporting bias in network meta-analysis of antidepressant placebo-controlled trials. *PLoS One* 2012; **7**(4): e35219.
73. Turner EH, Matthews AM, Linardatos E, Tell RA, Rosenthal R. Selective publication of antidepressant trials and its influence on apparent efficacy. *N Engl J Med* 2008; **358**(3): 252-60.
74. Chaimani A, Vasiliadis HS, Pandis N, Schmid CH, Welton NJ, Salanti G. Effects of study precision and risk of bias in networks of interventions: a network meta-epidemiological study. *Int J Epidemiol* 2013; **42**(4): 1120-31.
