# Supplementary material for: A systematic review and network meta-analysis of psychological, psychosocial, pharmacological, physical and combined treatments for adults with a new episode of depression
Source: eClinicalMedicine. 2024 Aug 16;75:102780. doi: 10.1016/j.eclinm.2024.102780 (PMC11377144; doi:10.1016/j.eclinm.2024.102780)
Supplement: Appendix 2 2023 Review update methods [file mmc2.pdf]

APPENDIX 2 – 2023 REVIEW UPDATE METHODS

CONTENTS

Search strategy update **2**
Machine learning methods **11**
Methods and data 11
Results 11

Search strategy update

Databases: Ovid Embase 2019 May 15 to 2023 November 27; Ovid Emcare 2019 Week 19 to 2023 Week 46

Date of search update: 28/11/2023

#	Searches
1	depression/ or agitated depression/ or atypical depression/ or depressive psychosis/ or dysthymia/ or endogenous depression/ or involutional depression/ or late life depression/ or major depression/ or masked depression/ or melancholia/ or "mixed anxiety and depression"/ or reactive depression/ or recurrent brief depression/ or treatment resistant depression/
2	(depress* or dysthym* or melanchol* or ((affective or mood) adj1 disorder*)).tw.
3	((sever* or serious* or major* or chronic* or complex* or critical* or endur* or persist* or resist* or acute) adj2 (anxiety or (mental adj2 (disorder* or health or illness* or ill-health)) or (obsessive adj2 disorder*) or OCD or panic attack* or panic disorder* or phobi* or personality disorder* or psychiatric disorder* or psychiatric illness* or psychiatric ill-health*)).tw.
4	or/1-3
5	exp psychotherapy/ or exp counseling/ or mindfulness/ or problem solving/ or psychiatric treatment/ or psychoeducation/ or self help/ or exp support group/
6	((behavio* or behavior* or abreact* or act* out* or age regression or assertive or autogenic or experiential) adj2 (activation or analy* or cathar* or conditioning or intervention* or modification* or therap* or training or treatment*)).tw.
7	((cognitive adj2 (behavior* or therap*)) or (CBT* or CBASP or biofeedback or contingency management or covert conditioning or covert sensiti?ation or defusion or MBCT* or neurofeedback or problem focus* or problem solving or rational emotive or REBT or schema or solution focus*) or ((third wave or 3rd wave) adj2 (intervention* or therap* or treatment*))).tw.
8	(counsel* or ((art or creative or compassion* or conversation* or dialectic* or emotion* or group* or insight or narrative or non-directive or nondirective or non-specific or nonspecific or rational or client-centred or client-centered or humanistic or integrative or interpersonal or person-centred or person-centered or personal construct or persuasion or Rogerian or talking or time-limited) adj2 (intervention* or therap* or training or treatment*))).tw.
9	(psychotherap* or (psycho* adj1 (aid* or help* or intervention* or support* or therap* or training or treatment*)) or (balint group or group program* or mindfulness* or mind training or role play* or support group*)).tw.
10	(self-help or bibliotherap* or meditat* or self-analy* or self-esteem or self-control or self-imag* or self-validat* or stress manag* or (computer* adj2 (intervention* or program* or therap* or treatment*)) or CCBT).tw.
11	or/5-10
12	drug therapy/ or drug therapy.fs.
13	psychopharmacotherapy/
14	antidepressant agent/
15	serotonin uptake inhibitor/
16	serotonin noradrenalin reuptake inhibitor/
17	tricyclic antidepressant agent/
18	monoamine oxidase inhibitor/
19	tetracyclic antidepressive agent/
20	amfebutamone/ or amineptine/ or amitriptyline/ or bupropion/ or clomipramine/ or chlorimipramine/ or citalopram/ or desipramine/ or duloxetine/ or Duloxetine Hydrochloride/ or escitalopram/ or fluvoxamine/ or fluoxetine/ or imipramine/ or lofepramine/ or mianserin/ or mirtazapine/ or moclobemide/ or nefazadone/ or nortriptyline/ or paroxetine/ or phenelzine/ or sertraline/ or venlafaxine/ or Venlafaxine Hydrochloride/
21	(antidepress* or amfebutamone or amineptin* or amitr?ptylin* or bupropion or chlorimipramine or clomipramin* or citalopram or desipramin* or duloxetin* or escitalopram or fluvoxamin* or fluoxetin* or imipramin* or lofepramin* or mianserin or mirtazapin* or moclobemide or nefazadon* or nortriptylin* or paroxetin* or phenelzin* or psychopharmacologic* or psychopharmacotherap* or sertralin* or venlafaxin* or SNRI* or SSRI* or TCA* or TeCA* or tetracyclic or tricyclic or ((monoamine or serotonin) adj2 inhibitor*)).tw.
22	or/12-21
23	anticonvulsive agent/ or anticonvulsant therapy/
24	lamotrigine/ or (lamotrigine or anticonvul* or anti convul*).tw.
25	or/23-24
26	neuroleptic agent/

#	Searches
27	amisulpride/ or aripiprazole/ or olanzapine/ or quetiapine/ or Quetiapine Fumarate/ or risperidone/ or ziprasidone/
28	(antipsychotic* or anti-psychotic* or amisulpride or aripiprazole or olanzapine or psychotropic* or quetiapine or risperidone or ziprasidone).tw.
29	or/26-28
30	anxiolytic agent/
31	buspirone/
32	(anxiolytic* or antianxiet* or anti-anxiet* or tranquili* or buspirone).tw.
33	or/30-32
34	central stimulant agent/
35	methylphenidate/ or (methylphenidate or ritalin).tw.
36	or/34-35
37	lithium/ or lithium.tw.
38	omega 3 fatty acid/
39	(omega adj1 ("fatty acid*" or PUFA*)).tw.
40	thyroid hormone/
41	(thyroid hormone* or calcitonin or dextrothyroxine or diiodotyrosine or monoiodotyrosine or thyronine* or thyroxine).tw.
42	or/37-41
43	acupuncture/ or acupuncture.tw.
44	electroconvulsive therapy/
45	(ECT or ((electroconvulsive or electro-convulsive) adj2 (therap* or treatment*)) or electroshock or (shock adj1 (therap* or treatment*))).tw.
46	exp exercise/
47	exp kinesiotherapy/ or exp physical activity/ or fitness/ or exp sport/
48	yoga/
49	(exercis* or yoga or cycling or bicycling or jogging or running or sport* or swimming or walking).tw.
50	or/43-49
51	peer group/ or mentoring/
52	friendship/
53	(befriend* or friend* or mentor* or peer support or (communit* adj1 (navigat* or support*))).tw.
54	or/51-53
55	or/11,22,25,29,33,36,42,50,54
56	4 and 55
57	letter.pt. or letter/
58	note.pt.
59	editorial.pt.
60	case report/ or case study/
61	(letter or comment*).ti.
62	or/57-61
63	randomized controlled trial/ or random*.ti,ab.
64	62 not 63
65	animal/ not human/
66	nonhuman/
67	exp Animal Experiment/
68	exp Experimental Animal/
69	animal model/
70	exp Rodent/
71	(rat or rats or rodent* or mouse or mice).ti.
72	or/64-71
73	56 not 72
74	limit 73 to english language
75	random*.ti,ab.
76	factorial*.ti,ab.
77	(crossover* or cross over*).ti,ab.
78	((doubl* or singl*) adj blind*).ti,ab.
79	(assign* or allocat* or volunteer* or placebo*).ti,ab.
80	crossover procedure/
81	single blind procedure/
82	randomized controlled trial/
83	double blind procedure/
84	or/75-83

#	Searches
85	systematic review/
86	meta-analysis/
87	(meta analy* or metanaly* or metaanaly*).ti,ab.
88	((systematic or evidence) adj2 (review* or overview*)).ti,ab.
89	(reference list* or bibliograph* or hand search* or manual search* or relevant journals).ab.
90	(search strategy or search criteria or systematic search or study selection or data extraction).ab.
91	(search* adj4 literature).ab.
92	(medline or pubmed or cochrane or embase or psychlit or psyclit or psychinfo or psycinfo or cinahl or science citation index or bids or cancerlit).ab.
93	((pool* or combined) adj2 (data or trials or studies or results)).ab.
94	cochrane.jw.
95	or/85-94
96	network meta-analysis/
97	((network adj1 (MA or MAs)) or (NMA or NMAs)).tw.
98	((indirect or mixed or multiple or multi-treatment* or simultaneous) adj1 comparison*).tw.
99	or/96-98
100	74 and 99
101	74 and 95
102	74 and 84
103	100 or 101 or 102
104	(conference abstract* or conference review or conference paper or conference proceeding).db,pt,su.
105	103 not 104

Databases: Cochrane Database of Systematic Reviews (CDSR), from Issue 6 of 12, June 2019 to Issue 11 of 12, November 2023; Cochrane Central Register of Controlled Trials (CENTRAL), from Issue 6 of 12, June 2019 to Issue 11 of 12, November 2023.

Date of search update: 28/11/2023

ID	Search
#1	MeSH descriptor: [Depression] this term only
#2	MeSH descriptor: [Depressive Disorder] this term only
#3	MeSH descriptor: [Depressive Disorder, Major] this term only
#4	MeSH descriptor: [Depressive Disorder, Treatment-Resistant] this term only
#5	MeSH descriptor: [Affective Disorders, Psychotic] this term only
#6	MeSH descriptor: [Dysthymic Disorder] this term only
#7	(depress* or dysphori* or dysthym* or melanchol* or ((affective or mood) next disorder*)):ti,ab
#8	((sever* or serious* or major* or acute or chronic* or complex* or endur* or persist* or resist*) next/2 (anxiety or (mental next/2 (disorder* or health or illness* or "ill health")) or (obsessive next/2 disorder*) or OCD or "panic attack" or "panic attacks" or "panic disorder" or "panic disorders" or phobi* or "personality disorder" or "personality disorders" or "psychiatric disorder" or "psychiatric disorders" or "psychiatric illness" or "psychiatric illnesses" or "psychiatric ill health")):ti,ab
#9	{or #1-#8}
#10	MeSH descriptor: [Psychotherapy] explode all trees
#11	MeSH descriptor: [Bibliotherapy] this term only
#12	MeSH descriptor: [Cognitive Behavioral Therapy] explode all trees
#13	MeSH descriptor: [Counseling] explode all trees
#14	MeSH descriptor: [Problem Solving] this term only
#15	MeSH descriptor: [Self Care] this term only
#16	MeSH descriptor: [Self Efficacy] this term only
#17	MeSH descriptor: [Self-Help Groups] this term only
#18	((behaviour* or behavior* or abreact* or "act out" or "acting out" or "age regression" or assertive or autogenic or experiential) next/2 (activation or analy* or cathar* or condition* or intervention* or modification* or therap* or training or treatment*)):ti,ab
#19	((cognitive next/2 (behavio* or therap*)) or (CBT* or CBASP or biofeedback or "contingency management" or "covert conditioning" or "covert sensitisation" or "covert sensiitization" or defusion or MBCT* or neurofeedback or "problem focus" or "problem focused" or "problem focusing" or "problem solving" or "rational emotive" or REBT or schema or "solution focus" or "solution focused" or "solution focusing") or (("third wave" or "3rd wave") next (intervention* or therap* or treatment*))):ti,ab
#20	(counsel* or ((art or creative or compassion* or conversation* or dialectic* or emotion* or group* or insight or narrative or "non directive" or nondirective or "non specific" or nonspecific or rational or "client

ID	Search
	centred" or "client centered" or humanistic or integrative or interpersonal or "person centred" or "person centered" or "personal construct" or "personal constructs" or persuasion or Rogerian or talking or "time limited") next (intervention* or therap* or training or treatment*))):ti,ab
#21	(psychotherap* or (psycho* next (aid* or help* or intervention* or support* or therap* or training or treatment*)) or ("balint group" or "balint groups" or "group program" or "group programs" or "group programme" or "group programmes" or mindfulness* or "mind training" or "role play" or "role playing" or "support group" or "support groups")):ti,ab
#22	("self help" or bibliotherap* or meditat* or (self next (analy* or imag* or validat*)) or "self esteem" or "self control" or (stress next manag*) or (computer* next/2 (intervention* or program* or therap* or treatment*)) or CCBT):ti,ab
#23	MeSH descriptor: [Drug Therapy] this term only
#24	MeSH descriptor: [Antidepressive Agents] this term only
#25	MeSH descriptor: [Selective Serotonin Reuptake Inhibitors] this term only
#26	MeSH descriptor: [Serotonin and Noradrenaline Reuptake Inhibitors] this term only
#27	MeSH descriptor: [Antidepressive Agents, Tricyclic] this term only
#28	MeSH descriptor: [Monoamine Oxidase Inhibitors] this term only
#29	MeSH descriptor: [Amitriptyline] this term only
#30	MeSH descriptor: [Clomipramine] this term only
#31	MeSH descriptor: [Citalopram] this term only
#32	MeSH descriptor: [Desipramine] this term only
#33	MeSH descriptor: [Duloxetine Hydrochloride] this term only
#34	MeSH descriptor: [Fluvoxamine] this term only
#35	MeSH descriptor: [Fluoxetine] this term only
#36	MeSH descriptor: [Imipramine] this term only
#37	MeSH descriptor: [Lofepramine] this term only
#38	MeSH descriptor: [Mianserin] this term only
#39	MeSH descriptor: [Mirtazapine] this term only
#40	MeSH descriptor: [Moclobemide] this term only
#41	MeSH descriptor: [Nortriptyline] this term only
#42	MeSH descriptor: [Paroxetine] this term only
#43	MeSH descriptor: [Phenelzine] this term only
#44	MeSH descriptor: [Sertraline] this term only
#45	MeSH descriptor: [Venlafaxine Hydrochloride] this term only
#46	(antidepress* or anti depress* or amfebutamone or amineptin* or amitriptylin* or amitryptylin* or bupropion or chlorimipramine or clomipramin* or citalopram or desipramin* or duloxetin* or escitalopram or fluvoxamin* or fluoxetin* or imipramin* or lofepramin* or mianserin or mirtazapin* or moclobemide or nefazadon* or nortriptylin* or paroxetin* or phenelzin* or psychopharmacologic* or psychopharmacotherap* or sertralin* or venlafaxin* or SNRI* or SSRI* or TCA* or TeCA* or tetracyclic or tricyclic or ((monoamine or serotonin) next/2 inhibitor*)):ti,ab
#47	MeSH descriptor: [Anticonvulsants] this term only
#48	MeSH descriptor: [Lamotrigine] this term only
#49	(lamotrigine or anticonvul* or anti convul*):ti,ab
#50	MeSH descriptor: [Antipsychotic Agents] this term only
#51	MeSH descriptor: [Amisulpride] this term only
#52	MeSH descriptor: [Aripiprazole] this term only
#53	MeSH descriptor: [Olanzapine] this term only
#54	MeSH descriptor: [Quetiapine Fumarate] this term only
#55	MeSH descriptor: [Risperidone] this term only
#56	(antipsychotic* or anti psychotic* or amisulpride or aripiprazole or olanzapine or psychotropic* or quetiapine or risperidone or ziprasidone):ti,ab
#57	MeSH descriptor: [Anti-Anxiety Agents] this term only
#58	MeSH descriptor: [Buspirone] this term only
#59	(anxiolytic* or antianxiet* or anti anxiet* or tranquilis* or tranquiliz* or buspirone):ti,ab
#60	MeSH descriptor: [Central Nervous System Stimulants] this term only
#61	MeSH descriptor: [Methylphenidate] this term only
#62	(methylphenidate or ritalin):ti,ab
#63	MeSH descriptor: [Lithium] this term only
#64	lithium:ti,ab
#65	MeSH descriptor: [Fatty Acids, Omega-3] explode all trees
#66	(omega next/2 ("fatty acid" or "fatty acids" or PUFA*)):ti,ab
#67	MeSH descriptor: [Thyroid Hormones] explode all trees

ID	Search
#68	("thyroid hormone" or "thyroid hormones" or calcitonin or dextrothyroxine or diiodotyrosine or monoiodotyrosine or thyronines or thyroxine):ti,ab
#69	MeSH descriptor: [Acupuncture] this term only
#70	acupuncture:ti,ab
#71	MeSH descriptor: [Electroconvulsive Therapy] this term only
#72	(ECT or ((electroconvuls* or electro convuls*) next/2 (therap* or treatment*)) or electroshock* or (shock next (therap* or treatment*))):ti,ab
#73	MeSH descriptor: [Exercise Therapy] explode all trees
#74	MeSH descriptor: [Physical Exertion] this term only
#75	MeSH descriptor: [Physical Fitness] explode all trees
#76	MeSH descriptor: [Bicycling] this term only
#77	MeSH descriptor: [Running] explode all trees
#78	MeSH descriptor: [Swimming] this term only
#79	MeSH descriptor: [Walking] this term only
#80	MeSH descriptor: [Yoga] this term only
#81	(exercis* or yoga or cycling or bicycling or jogging or running or sport* or swimming or walking):ti,ab
#82	MeSH descriptor: [Peer Group] this term only
#83	MeSH descriptor: [Mentoring] this term only
#84	MeSH descriptor: [Friends] this term only
#85	(befriend* or friend* or mentor* or "peer group" or "peer groups" or “peer support" or (communit* next (navigat* or support*))):ti,ab
#86	{or #10-#85}
#87	#9 and #86 with Cochrane Library publication date Between Mar 2021 and Nov 2023, in Cochrane Reviews, Cochrane Protocols
#88	#9 and #86 with Publication Year from 2020 to 2023, in Trials
#89	"conference":pt or (clinicaltrials or trialsearch):so
#90	#88 not #89

Databases: Ovid MEDLINE ALL from May 15, 2019 to November 27, 2023

Date of search update: 28/11/2023

#	Searches
1	Depression/ or Depressive Disorder/ or Depressive Disorder, Major/ or Depressive Disorder, Treatment-Resistant/ or Disorders, Psychotic/ or Dysthymic Disorder/
2	(depress* or dysthym* or melanchol* or ((affective or mood) adj1 disorder*)).tw.
3	((sever* or serious* or major* or chronic* or complex* or critical* or endur* or persist* or resist* or acute) adj2 (anxiety or (mental adj2 (disorder* or health or illness* or ill-health)) or (obsessive adj2 disorder*) or OCD or panic attack* or panic disorder* or phobi* or personality disorder* or psychiatric disorder* or psychiatric illness* or psychiatric ill-health*)).tw.
4	or/1-3
5	exp Psychotherapy/ or Bibliotherapy/ or exp Cognitive Behavioral Therapy/ or exp Counseling/ or Problem Solving/ or Self Care/ or Self Efficacy/ or Self-Help Groups/
6	((behavio* or abreact* or act* out* or age regression or assertive or autogenic or experiential) adj2 (activation or analy* or cathar* or conditioning or intervention* or modif* or therap* or training or treatment*)).tw.
7	((cognitive adj2 (behavio* or therap*)) or (CBT* or CBASP or biofeedback or contingency management or covert conditioning or covert sensiti?ation or defusion or MBCT* or neurofeedback or problem focus* or problem solving or rational emotive or REBT or schema or solution focus*) or ((third wave or 3rd wave) adj2 (intervention* or therap* or treatment*))).tw.
8	(counsel* or ((art or creative or compassion* or conversation* or dialectic* or emotion* or group* or insight or narrative or non-directive or nondirective or non-specific or nonspecific or rational or client-centred or client-centered or humanistic or integrative or interpersonal or person-centred or person-centered or personal construct or persuasion or Rogerian or talking or time-limited) adj2 (intervention* or therap* or training or treatment*))).tw.
9	(psychotherap* or (psycho* adj1 (aid* or help* or intervention* or support* or therap* or training or treatment*)) or (balint group or group program* or mindfulness* or mind training or role play* or support group*)).tw.
10	(self-help or bibliotherap* or meditat* or self-analy* or self-esteem or self-control or self-imag* or self-validat* or stress manag* or (computer* adj2 (intervention* or program* or therap* or treatment*)) or CCBT).tw.
11	or/5-10

#	Searches
12	drug therapy/ or drug therapy.fs.
13	Antidepressive Agents/
14	Serotonin Uptake Inhibitors/
15	"Serotonin and Noradrenaline Reuptake Inhibitors"/
16	Antidepressive Agents, Tricyclic/
17	monoamine oxidase inhibitors/
18	amfebutamone/ or amineptine/ or amitriptyline/ or bupropion/ or clomipramine/ or chlorimipramine/ or citalopram/ or desipramine/ or duloxetine/ or Duloxetine Hydrochloride/ or escitalopram/ or fluvoxamine/ or fluoxetine/ or imipramine/ or lofepramine/ or mianserin/ or mirtazapine/ or moclobemide/ or nefazadone/ or nortriptyline/ or paroxetine/ or phenelzine/ or sertraline/ or venlafaxine/ or Venlafaxine Hydrochloride/
19	(antidepress* or amfebutamone or amineptin* or amitr?ptylin* or bupropion or chlorimipramine or clomipramin* or citalopram or desipramin* or duloxetin* or escitalopram or fluvoxamin* or fluoxetin* or imipramin* or lofepramin* or mianserin or mirtazapin* or moclobemide or nefazadon* or nortriptylin* or paroxetin* or phenelzin* or psychopharmacologic* or psychopharmacotherap* or sertralin* or venlafaxin* or SNRI* or SSRI* or TCA* or TeCA* or tetracyclic or tricyclic or ((monoamine or serotonin) adj2 inhibitor*)).tw.
20	or/12-19
21	Anticonvulsants/
22	lamotrigine/ or (lamotrigine or anticonvul* or anti convul*).tw.
23	or/21-22
24	Antipsychotic Agents/
25	amisulpride/ or aripiprazole/ or olanzapine/ or quetiapine/ or Quetiapine Fumarate/ or risperidone/ or ziprasidone/
26	(antipsychotic* or anti-psychotic* or amisulpride or aripiprazole or olanzapine or psychotropic* or quetiapine or risperidone or ziprasidone).tw.
27	or/24-26
28	Anti-Anxiety Agents/
29	buspirone/
30	(anxiolytic* or antianxiet* or anti-anxiet* or tranquili* or buspirone).tw.
31	or/28-30
32	Central Nervous System Stimulants/
33	methylphenidate/ or (methylphenidate or ritalin).tw.
34	or/32-33
35	lithium/ or lithium.tw.
36	Fatty Acids, Omega-3/
37	(omega adj1 (fatty acid* or PUFA*)).tw.
38	Thyroid Hormones/
39	(thyroid hormone* or calcitonin or dextrothyroxine or diiodotyrosine or monoiodotyrosine or thyronine* or thyroxine).tw.
40	or/35-39
41	acupuncture/ or acupuncture.tw.
42	electroconvulsive therapy/
43	(ECT or ((electroconvulsive or electro-convulsive) adj2 (therap* or treatment*)) or electroshock or (shock adj1 (therap* or treatment*))).tw.
44	exp Exercise/ or exp Exercise Therapy/ or Physical Exertion/ or exp Physical Fitness/ or Bicycling/ or exp Running/ or Swimming/ or Walking/
45	yoga/
46	(exercis* or yoga or cycling or bicycling or jogging or running or sport* or swimming or walking).tw.
47	or/41-46
48	peer group/ or mentoring/
49	Friends/ or Friendship/
50	(befriend* or friend* or mentor* or peer support or (communit* adj1 (navigat* or support*))).tw.
51	or/48-50
52	or/11,20,23,27,31,34,40,47,51
53	4 and 52
54	letter/
55	editorial/
56	news/
57	exp historical article/
58	Anecdotes as topic/
59	comment/

#	Searches
60	case reports/
61	(letter or comment*).ti.
62	or/54-61
63	randomized controlled trial/ or random*.ti,ab.
64	62 not 63
65	animals/ not humans/
66	exp Animals, Laboratory/
67	exp Animal Experimentation/
68	exp Models, Animal/
69	exp Rodentia/
70	(rat or rats or rodent* or mouse or mice).ti.
71	or/64-70
72	53 not 71
73	limit 72 to english language
74	randomized controlled trial.pt.
75	controlled clinical trial.pt.
76	pragmatic clinical trial.pt.
77	randomi#ed.ab.
78	placebo.ab.
79	randomly.ab.
80	Clinical Trials as topic.sh.
81	trial.ti.
82	or/74-81
83	meta-analysis/
84	meta-analysis as topic/
85	(meta analy* or metanaly* or metaanaly*).ti,ab.
86	((systematic* or evidence*) adj2 (review* or overview*)).ti,ab.
87	(reference list* or bibliograph* or hand search* or manual search* or relevant journals).ab.
88	(search strategy or search criteria or systematic search or study selection or data extraction).ab.
89	(search* adj4 literature).ab.
90	(medline or pubmed or cochrane or embase or psychlit or psyclit or psychinfo or psycinfo or cinahl or science citation index or bids or cancerlit).ab.
91	cochrane.jw.
92	or/83-91
93	network meta-analysis/
94	((network adj1 (MA or MAs)) or (NMA or NMAs)).tw.
95	((indirect or mixed or multiple or multi-treatment* or simultaneous) adj1 comparison*).tw.
96	or/93-95
97	73 and 96
98	73 and 92
99	73 and 82
100	or/97-99

Databases: Ovid APA PsycInfo from May Week 1 2019 to November Week 2 2023

Date of search update: 28/11/2023

#	Searches
1	"depression (emotion)"/ or exp major depression/ or affective disorders/ or atypical depression/
2	(depress* or dysthym* or melanchol* or ((affective or mood) adj1 disorder*)).tw.
3	((sever* or serious* or major* or chronic* or complex* or critical* or endur* or persist* or resist* or acute) adj2 (anxiety or (mental adj2 (disorder* or health or illness* or ill-health)) or (obsessive adj2 disorder*) or OCD or panic attack* or panic disorder* or phobi* or personality disorder* or psychiatric disorder* or psychiatric illness* or psychiatric ill-health*)).tw.
4	or/1-3
5	exp psychotherapy/ or behavioral activation system/ or bibliotherapy/ or cognitive therapy/ or exp counseling/ or group intervention/ or mindfulness/ or exp problem solving/ or psychoeducation/ or exp self-help techniques/ or support groups/
6	((behavio* or behavior* or abreact* or act* out* or age regression or assertive or autogenic or experiential) adj2 (activation or analy* or cathar* or conditioning or intervention* or modification* or therap* or training or treatment*)).tw.

#	Searches
7	((cognitive adj2 (behavior* or therap*)) or (CBT* or CBASP or biofeedback or contingency management or covert conditioning or covert sensiti?ation or defusion or MBCT* or neurofeedback or problem focus* or problem solving or rational emotive or REBT or schema or solution focus*) or ((third wave or 3rd wave) adj2 (intervention* or therap* or treatment*))).tw.
8	(counsel* or ((art or creative or compassion* or conversation* or dialectic* or emotion* or group* or insight or narrative or non-directive or nondirective or non-specific or nonspecific or rational or client-centred or client-centered or humanistic or integrative or interpersonal or person-centred or person-centered or personal construct or persuasion or Rogerian or talking or time-limited) adj2 (intervention* or therap* or training or treatment*))).tw.
9	(psychotherap* or (psycho* adj1 (aid* or help* or intervention* or support* or therap* or training or treatment*)) or (balint group or group program* or mindfulness* or mind training or role play* or support group*)).tw.
10	(self-help or bibliotherap* or meditat* or self-analy* or self-esteem or self-control or self-imag* or self-validat* or stress manag* or (computer* adj2 (intervention* or program* or therap* or treatment*)) or CCBT).tw.
11	or/5-10
12	drug therapy/
13	psychopharmacology/
14	antidepressant drugs/
15	serotonin reuptake inhibitors/
16	serotonin norepinephrine reuptake inhibitors/
17	tricyclic antidepressant drugs/
18	monoamine oxidase inhibitors/
19	amfebutamone/ or amineptine/ or amitriptyline/ or bupropion/ or clomipramine/ or chlorimipramine/ or citalopram/ or desipramine/ or duloxetine/ or Duloxetine Hydrochloride/ or escitalopram/ or fluvoxamine/ or fluoxetine/ or imipramine/ or lofepramine/ or mianserin/ or mirtazapine/ or moclobemide/ or nefazadone/ or nortriptyline/ or paroxetine/ or phenelzine/ or sertraline/ or venlafaxine/ or Venlafaxine Hydrochloride/
20	(antidepress* or amfebutamone or amineptin* or amitr?ptylin* or bupropion or chlorimipramine or clomipramin* or citalopram or desipramin* or duloxetin* or escitalopram or fluvoxamin* or fluoxetin* or imipramin* or lofepramin* or mianserin or mirtazapin* or moclobemide or nefazadon* or nortriptylin* or paroxetin* or phenelzin* or psychopharmacologic* or psychopharmacotherap* or sertralin* or venlafaxin* or SNRI* or SSRI* or TCA* or TeCA* or tetracyclic or tricyclic or ((monoamine or serotonin) adj2 inhibitor*)).tw.
21	or/12-20
22	anticonvulsive drugs/
23	lamotrigine/ or (lamotrigine or anticonvul* or anti convul*).tw.
24	or/22-23
25	neuroleptic drugs/
26	amisulpride/ or aripiprazole/ or olanzapine/ or quetiapine/ or Quetiapine Fumarate/ or risperidone/ or ziprasidone/
27	(antipsychotic* or anti-psychotic* or amisulpride or aripiprazole or olanzapine or psychotropic* or quetiapine or risperidone or ziprasidone).tw.
28	or/25-27
29	tranquilizing drugs/
30	buspirone/
31	(anxiolytic* or antianxiet* or anti-anxiet* or tranquili* or buspirone).tw.
32	or/29-31
33	CNS stimulating drugs/
34	methylphenidate/ or (methylphenidate or ritalin).tw.
35	or/33-34
36	lithium/ or lithium.tw.
37	fatty acids/
38	(omega adj1 ("fatty acid*" or PUFA*)).tw.
39	exp thyroid hormones/
40	(thyroid hormone* or calcitonin or dextrothyroxine or diiodotyrosine or monoiodotyrosine or thyronine* or thyroxine).tw.
41	or/36-40
42	acupuncture/ or acupuncture.tw.
43	electroconvulsive shock therapy/
44	(ECT or ((electroconvulsive or electro-convulsive) adj2 (therap* or treatment*)) or electroshock or (shock adj1 (therap* or treatment*))).tw.
45	exp exercise/

#	Searches
46	exp physical fitness/ or exp sports/
47	yoga/
48	(exercis* or yoga or cycling or bicycling or jogging or running or sport* or swimming or walking).tw.
49	or/42-48
50	peers/ or mentor/
51	peer relations/
52	friendship/
53	(befriend* or friend* or mentor* or peer support or (communit* adj1 (navigat* or support*))).tw.
54	or/50-53
55	or/11,21,24,28,32,35,41,49,54
56	4 and 55
57	(letter or editorial or comment reply).dt. or case report/
58	(letter or comment*).ti.
59	or/57-58
60	exp randomized controlled trial/
61	random*.ti,ab.
62	or/60-61
63	59 not 62
64	animal.po.
65	(rat or rats or rodent* or mouse or mice).ti.
66	or/63-65
67	56 not 66
68	limit 67 to english language
69	clinical trial.md.
70	Clinical trials/
71	Randomized controlled trials/
72	Randomized clinical trials/
73	assign*.ti,ab.
74	allocat*.ti,ab.
75	crossover*.ti,ab.
76	cross over*.ti,ab.
77	((doubl* or singl*) adj blind*).ti,ab.
78	factorial*.ti,ab.
79	placebo*.ti,ab.
80	random*.ti,ab.
81	volunteer*.ti,ab.
82	trial?.ti,ab.
83	or/69-82
84	(meta analysis or "systematic review").md.
85	META ANALYSIS/
86	SYSTEMATIC REVIEW/
87	(meta analy* or metanaly* or metaanaly*).ti,ab.
88	((systematic* or evidence*) adj2 (review* or overview*)).ti,ab.
89	(reference list* or bibliograph* or hand search* or manual search* or relevant journals).ab.
90	(search strategy or search criteria or systematic search or study selection or data extraction).ab.
91	(search* adj4 literature).ab.
92	((pool* or combined) adj2 (data or trials or studies or results)).ab.
93	(medline or pubmed or cochrane or embase or psychlit or psyclit or cinahl or science citation index or bids or cancerlit).ab.
94	or/84-93
95	((network adj1 (MA or MAs)) or (NMA or NMAs)).tw.
96	((indirect or mixed or multiple or multi-treatment* or simultaneous) adj1 comparison*).tw.
97	95 or 96
98	68 and 97
99	68 and 94
100	68 and 83
101	98 or 99 or 100

Machine learning methods

Methods and data

The data used for machine learning were titles and abstracts of the records screened in the original review. We had the following available for machine learning:

- 93 records previously included in the review
- 579 records that had been excluded on full text assessment
- 41,927 records that had been excluded based on title and abstract assessment

Based on this dataset, we needed to reduce the manual workload involved in screening the 26,024 records retrieved in the 2023 update search.

To maximise the number of relevant (or near relevant) records, we combined the 93 included references with the 579 that had passed initial eligibility assessment before being excluded at full text screening. This gave us a dataset of 672 ‘positive class’ records, and 41,927 ‘negative class’ records.

We split the data in an 80:20 ratio, using 80% of the records for training the machine learning model, and reserving 20% to assess its performance.

We built a logistic regression machine learning model using tri-gram ‘bag-of-words’ features based on the titles and abstracts of the training data, and then scored all the records reserved for testing.

Results

Figure 1 summarises the distribution of scores found in the test dataset. It shows an apparently well-performing model, correctly predicting that the majority of records are highly unlikely to be relevant, and a comparatively small number of possibly relevant records.

Table 1 shows the distribution of scores in more detail, examining where the positive and negative class records lie. Here, the picture is a little more complicated with the vast majority of negative class records (excluded on title and abstract) correctly given the lowest scores, but the records that were included were also rather evenly distributed. However, none of the records included in the review scored below 10% and, when examined in more detail, it was clear that most of the irrelevant records scored very low indeed (less than 5) giving assurance that it would be possible to set a threshold for excluding records below a given score with very little risk of missing any eligible records.

Figure 1: Distribution of scores in the test dataset

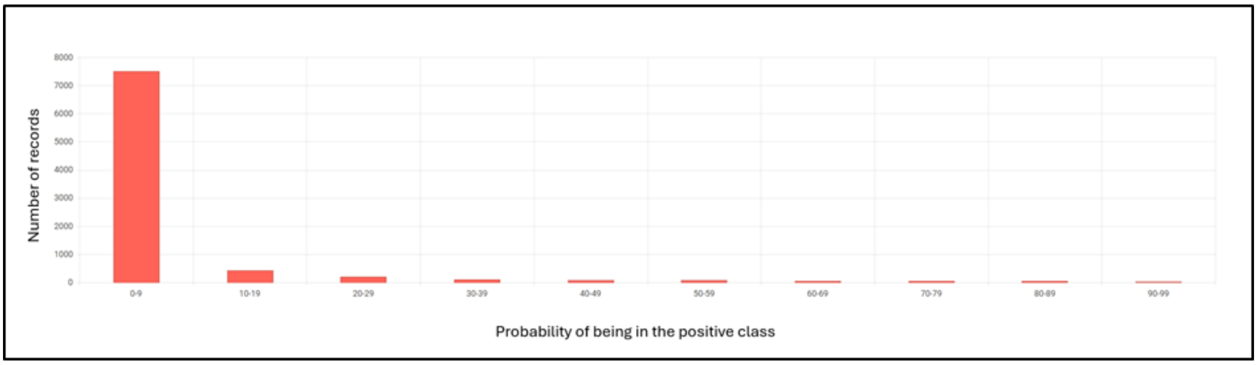

Table 1: Distribution of scores of included / excluded records

	Included in review	Excluded at full text	Excluded on title and abstract
**0-9 range**	0	18	7487
**10-19 range**	3	13	401
**20-29 range**	2	12	195
**30-39 range**	2	10	92
**40-49 range**	3	10	53
**50-59 range**	1	12	55
**60-69 range**	2	8	33
**70-79 range**	6	7	31
**80-89 range**	2	14	28
**90-99 range**	3	6	10

We then scored the 26,024 records from the 2023 update using the above model. The distribution of scores looked exactly the same as Figure 1, with the majority scoring very low, and a few records scoring > 20. Given the similarity seen in the scores between the initial dataset, and the update, we considered it justifiable to use the model at the above threshold, excluding everything that scored below 5 (i.e. all records where $p < 0.05$). This left a a total of 5,656 records for manual assessment out of the 26,024; a workload saving of 78%.
