# Supplementary material for: A systematic review and network meta-analysis of psychological, psychosocial, pharmacological, physical and combined treatments for adults with a new episode of depression
Source: eClinicalMedicine. 2024 Aug 16;75:102780. doi: 10.1016/j.eclinm.2024.102780 (PMC11377144; doi:10.1016/j.eclinm.2024.102780)
Supplement: Appendix 4 Networks, treatments, Nrandomised [file mmc4.pdf]

APPENDIX 4

CONTENTS

Networks, treatment classes, interventions and numbers of participants tested on each, for all outcomes considered in the network meta-analysis 2

Less severe depression.. 2

SMD of depressive symptom scale change scores 2

Response in those randomised... 6

Remission in those randomised 11

Treatment discontinuation for any reason 14

Treatment discontinuation due to side effects from medication, in those who discontinued treatment.. 18

Response in treatment completers.. 19

Remission in treatment completers 23

More severe depression 26

SMD of depressive symptom scale change scores 26

Response in those randomised.. 31

Remission in those randomised 35

Treatment discontinuation for any reason 39

Treatment discontinuation due to side effects from medication, in those who discontinued treatment.. 43

Response in treatment completers.. 46

Remission in treatment completers 51

Networks, treatment classes, interventions and numbers of participants tested on each, for all outcomes considered in the network meta-analysis

Less severe depression

SMD of depressive symptom scale change scores

Network - treatment class level

See Figure 2 in main article

Network - intervention level

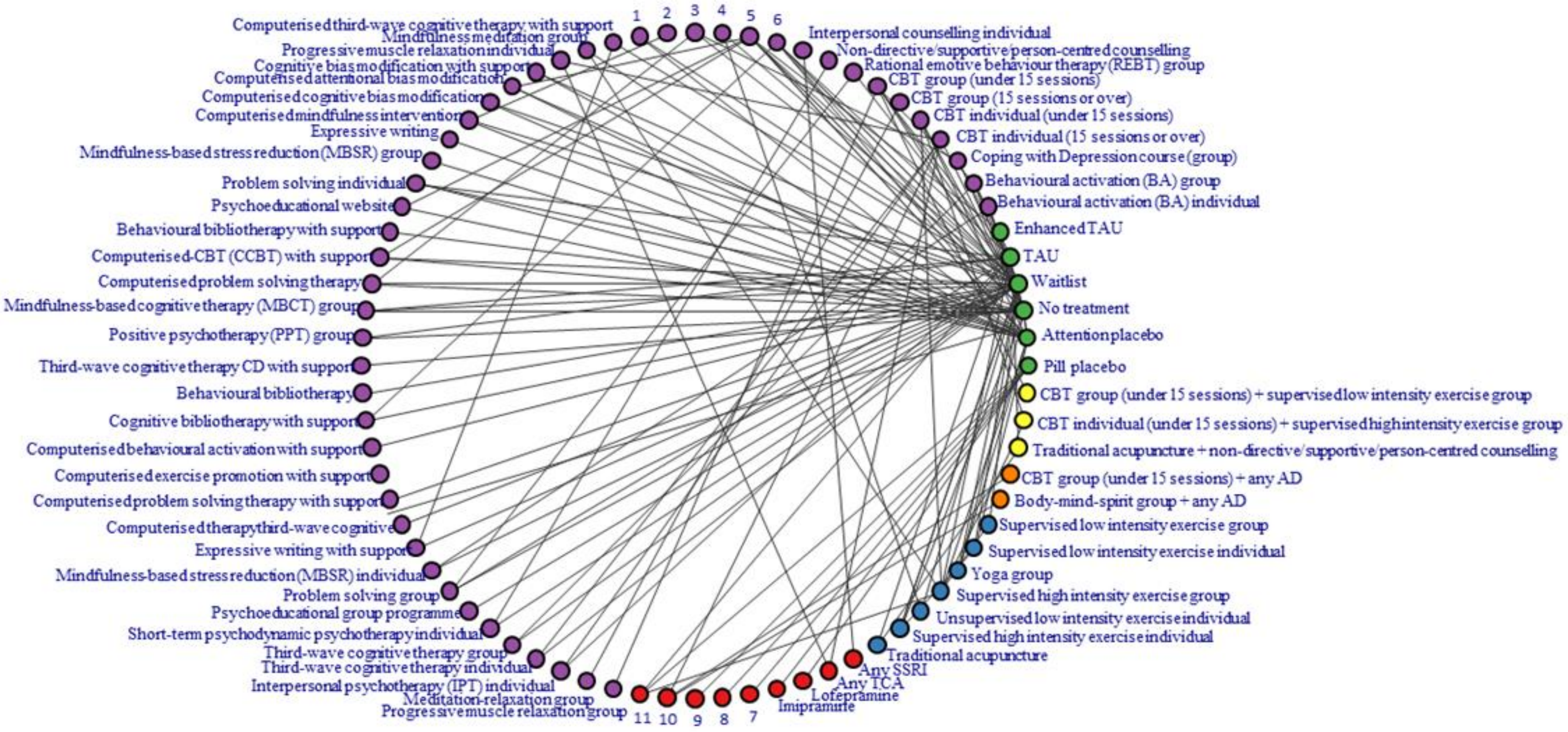

AD: antidepressant; CBT: cognitive behavioural therapy; CT: cognitive therapy; IPT: interpersonal psychotherapy; MBCT: mindfulness-based cognitive therapy; PDPT: psychodynamic psychotherapy; SSRI: selective serotonin uptake inhibitor; TAU: treatment as usual; TCA: tricyclic antidepressant

1 Computerised positive psychological intervention; 2 Computerised expressive writing; 3 Computerised Coping with Depression course; 4 Computerised behavioural activation; 5 Computerised-CBT (CCBT); 6 Cognitive bibliotherapy; 7 Fluoxetine; 8 Citalopram; 9 Amitriptyline; 10 Any AD; 11 Sertraline

Without the use of a class network CBT group (15 sessions or over), Interpersonal counselling individual, Meditation-relaxation group and Any SSRI would be disconnected from the rest of the network and would have to be excluded from the analysis.

Classes, interventions and numbers of participants tested on each
The NMA included 127 RCTs, 76 interventions grouped in 34 treatment classes, and 16,829 participants. Of the 127 RCTs, 10 reported change from baseline (CFB) depression symptom score data; 115 reported baseline and endpoint depression symptom score data; and 2 reported dichotomous response data and baseline symptom scores.

Treatment class	N	Intervention	N	Variance Sharing*
Attention placebo	935	Attention placebo	935	
Placebo	301	Pill placebo	301	
No treatment	1,478	No treatment	1,478	
Waitlist	3,555	Waitlist	3,555	
TAU	815	TAU	815	
Enhanced TAU	36	Enhanced TAU	36	
Self-help without/with minimal support	4,922	Behavioural bibliotherapy	13	3
		Cognitive bibliotherapy	516	
		Computerised-CBT (CCBT)	2,619	
		Computerised attentional bias modification	230	
		Computerised behavioural activation	122	
		Computerised cognitive bias modification	75	
		Computerised Coping with Depression course	257	
		Computerised expressive writing	36	
		Computerised mindfulness intervention	174	
		Computerised positive psychological intervention	439	
		Computerised problem solving therapy	232	
		Computerised third-wave CT	31	
		Expressive writing	13	
		Psychoeducational website	165	
Self-help with support	1,286	Behavioural bibliotherapy with support	67	4
		Cognitive bias modification with support	20	
		Cognitive bibliotherapy with support	125	
		Computerised-CBT (CCBT) with support	396	
		Computerised behavioural activation with support	40	
		Computerised exercise promotion with support	24	
		Computerised problem solving therapy with support	124	
		Computerised third-wave CT with support	82	
		Expressive writing with support	125	
		Third-wave CT CD with support	283	
Behavioural therapies individual	147	Behavioural activation (BA) individual	147	1
Behavioural therapies group	340	Behavioural activation (BA) group	117	
		Coping with Depression course (group)	223	
CT/CBT individual	481	CBT individual (15 sessions or over)	123	1
		CBT individual (under 15 sessions)	233	
		Third-wave CT individual	125	
CT/CBT group	480	CBT group (15 sessions or over)	10	2
		CBT group (under 15 sessions)	316	
		Positive psychotherapy (PPT) group	76	
		Rational emotive behaviour therapy (REBT) group	14	
		Third-wave CT group	64	
Problem solving individual	98	Problem solving individual	98	1
Problem solving group	104	Problem solving group	104	1
Counselling individual	55	Non-directive/supportive/person-centred counselling	55	1
IPT individual	153	Interpersonal counselling individual	17	1
		IPT individual	136	
Short-term PDPT individual	49	Short-term PDPT individual	49	1

Psychoeducation group	22	Psychoeducational group programme	22	1
Mindfulness or meditation individual	20	Mindfulness-based stress reduction (MBSR) individual	20	1
Mindfulness or meditation group	376	Meditation-relaxation group	13	5
		MBCT group	149	
		Mindfulness-based stress reduction (MBSR) group	85	
		Mindfulness meditation group	129	
Relaxation individual	13	Progressive muscle relaxation individual	13	1
Relaxation group	63	Progressive muscle relaxation group	63	2
SSRIs	207	Any SSRI	24	6
		Citalopram	24	
		Fluoxetine	78	
		Sertraline	81	
TCAs	136	Amitriptyline	67	6
		Any TCA	10	
		Imipramine	36	
		Lofepramine	23	
Any AD	65	Any AD	65	6
Acupuncture	40	Traditional acupuncture	40	1
Exercise individual	250	Supervised high intensity exercise individual	43	7
		Supervised low intensity exercise individual	86	
		Unsupervised low intensity exercise individual	121	
Exercise group	199	Supervised high intensity exercise group	147	8
		Supervised low intensity exercise group	52	
Yoga group	73	Yoga group	73	2
CT/CBT group + AD	32	CBT group (under 15 sessions) + any AD	32	1
Mindfulness or meditation group + AD	15	Body-mind-spirit group + any AD	15	1
Acupuncture + counselling individual	40	Traditional acupuncture + non-directive/supportive/person-centred counselling	40	1
CT/CBT individual + exercise group	18	CBT individual (under 15 sessions) + supervised high intensity exercise group	18	1
CT/CBT group + exercise group	25	CBT group (under 15 sessions) + supervised low intensity exercise group	25	1

AD: antidepressant; CBT: cognitive behavioural therapy; CT: cognitive therapy; IPT: interpersonal psychotherapy; MBCT: mindfulness-based cognitive therapy; PDPT: psychodynamic psychotherapy; SSRIs: selective serotonin uptake inhibitors; TAU: treatment as usual; TCAs: tricyclic antidepressants
** Classes with the same number share a common class variance*

Response in those randomised

Network - treatment class level

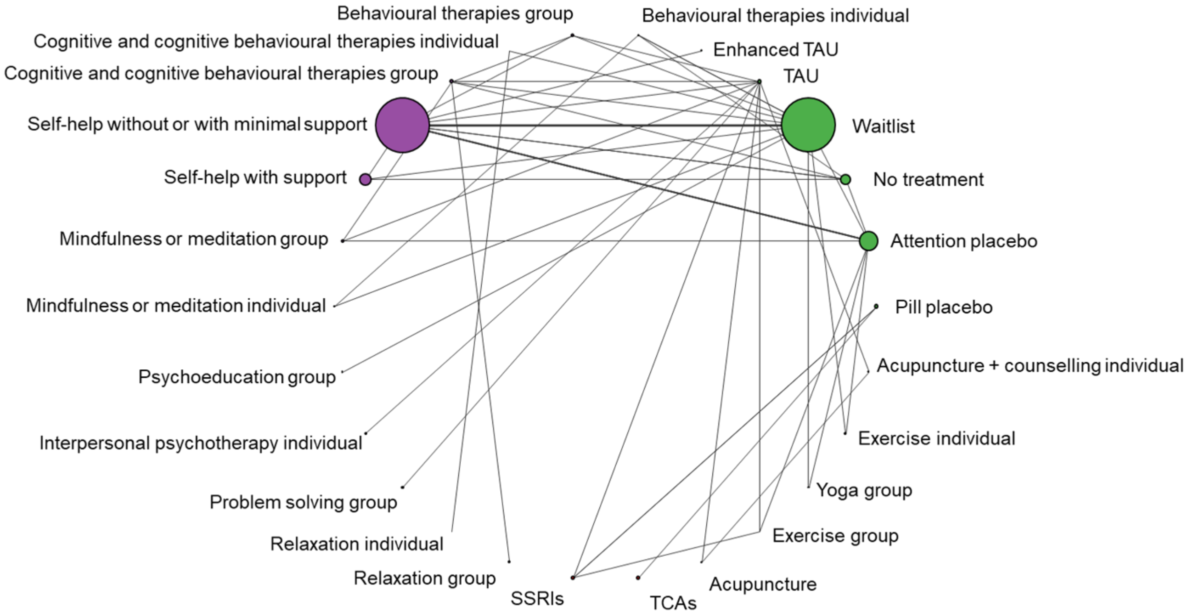

SSRIs: selective serotonin uptake inhibitors; TAU: treatment as usual; TCAs: tricyclic antidepressants

Network - intervention level

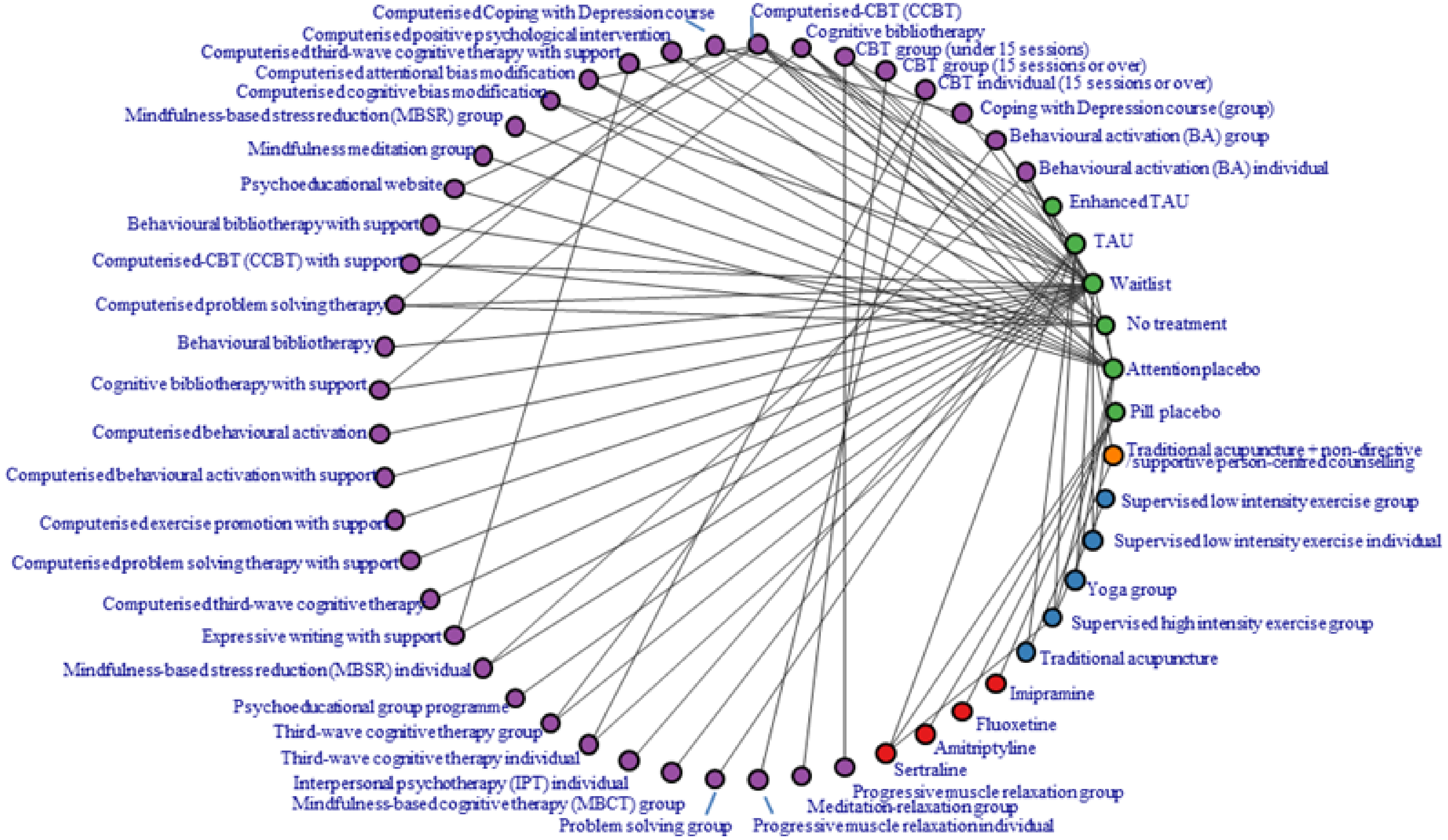

CBT: cognitive behavioural therapy; CT: cognitive therapy; IPT: interpersonal psychotherapy; MBCT: mindfulness-based cognitive therapy; PDPT: psychodynamic psychotherapy; SSRIs: selective serotonin uptake inhibitors; TAU: treatment as usual; TCAs: tricyclic antidepressants
Without the use of a class network CBT group (15 sessions or over) and Meditation-relaxation group would be disconnected from the rest of the network and would have to be excluded from the analysis. Any AD, Mindfulness group + AD, Non-directive/supportive/person-centred counselling and Short-term psychodynamic psychotherapy individual were excluded from the NMA as they were disconnected from the network.

Classes, interventions and numbers of participants tested on each

The NMA included 75 RCTs, 53 interventions grouped in 26 treatment classes and 12,549 participants. Of the 75 RCTs, 11 reported dichotomous response data, 6 reported CFB depression symptom score data; and 58 reported baseline and endpoint depression symptom score data.

Treatment class	N	Intervention	N	Variance Sharing*
Waitlist	3,144	Waitlist	3,144	
Placebo	303	Pill placebo	303	
Attention placebo	727	Attention placebo	727	
No treatment	718	No treatment	718	
TAU	623	TAU	623	
Enhanced TAU	36	Enhanced TAU	36	
Self-help	4,373	Behavioural bibliotherapy	13	2
		Cognitive bibliotherapy	516	
		Computerised-CBT (CCBT)	2,541	
		Computerised attentional bias modification	181	
		Computerised behavioural activation	10	
		Computerised cognitive bias modification	55	
		Computerised Coping with Depression course	190	
		Computerised positive psychological intervention	439	
		Computerised problem solving therapy	232	
		Computerised third-wave CT	31	
		Psychoeducational website	165	
Self-help with support	849	Behavioural bibliotherapy with support	67	3
		Cognitive bibliotherapy with support	125	
		Computerised-CBT (CCBT) with support	262	
		Computerised behavioural activation with support	40	
		Computerised exercise promotion with support	24	
		Computerised problem solving therapy with support	124	
		Computerised third-wave CT with support	82	
		Expressive writing with support	125	
Behavioural therapies individual	65	Behavioural activation (BA) individual	65	1
Behavioural therapies group	184	Behavioural activation (BA) group	85	1
		Coping with Depression course (group)	99	
CT/CBT individual	121	CBT individual (15 sessions or over)	56	1
		Third-wave CT individual	65	
CT/CBT group	341	CBT group (15 sessions or over)	10	1
		CBT group (under 15 sessions)	267	
		Third-wave CT group	64	
Problem solving group	89	Problem solving group	89	1
IPT individual	69	IPT individual	69	1
Psychoeducation group	22	Psychoeducational group programme	22	1
Mindfulness or meditation individual	20	Mindfulness-based stress reduction (MBSR) individual	20	1
Mindfulness or meditation group	197	Meditation-relaxation group	13	1
		MBCT group	76	
		Mindfulness-based stress reduction (MBSR) group	70	
		Mindfulness meditation group	38	
Relaxation individual	15	Progressive muscle relaxation individual	15	1
Relaxation group	63	Progressive muscle relaxation group	63	1
SSRIs	159	Fluoxetine	78	4
		Sertraline	81	
TCAs	163	Amitriptyline	90	4
		Imipramine	73	
Acupuncture	40	Traditional acupuncture	40	1

Exercise individual	71	Supervised low intensity exercise individual	71	3
Exercise group	52	Supervised high intensity exercise group	42	3
		Supervised low intensity exercise group	10	
Yoga group	65	Yoga group	65	1
Acupuncture + counselling individual	40	Traditional acupuncture + non-directive/ supportive/ person-centred counselling	40	1

CBT: cognitive behavioural therapy; CT: cognitive therapy; IPT: interpersonal psychotherapy; MBCT: mindfulness-based cognitive therapy; SSRIs: selective serotonin uptake inhibitors; TAU: treatment as usual; TCAs: tricyclic antidepressants
** Classes with the same number share a common class variance*

Remission in those randomised

Network - treatment class level

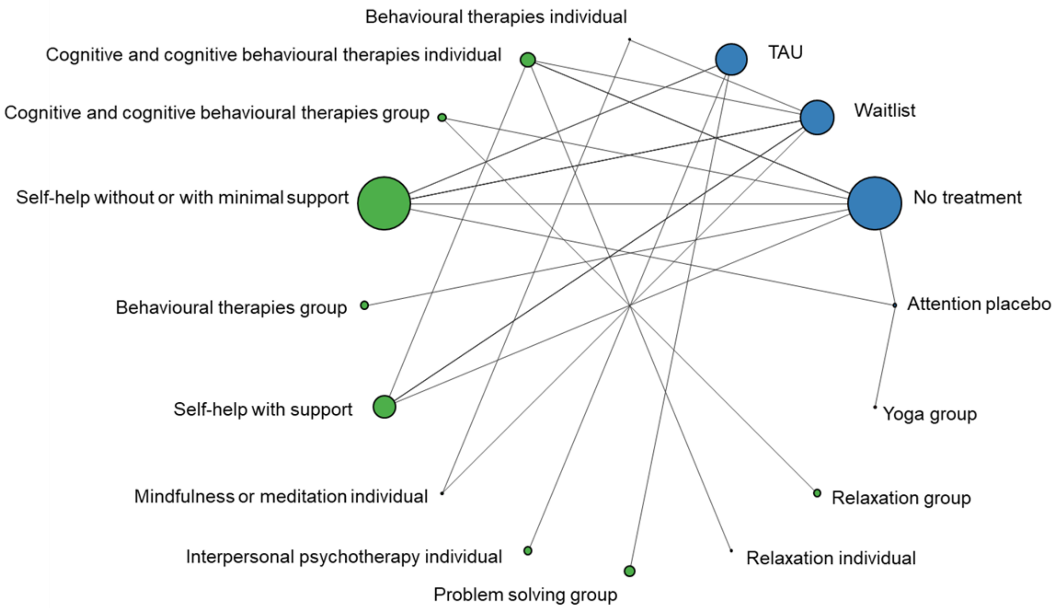

TAU: treatment as usual

Network - intervention level

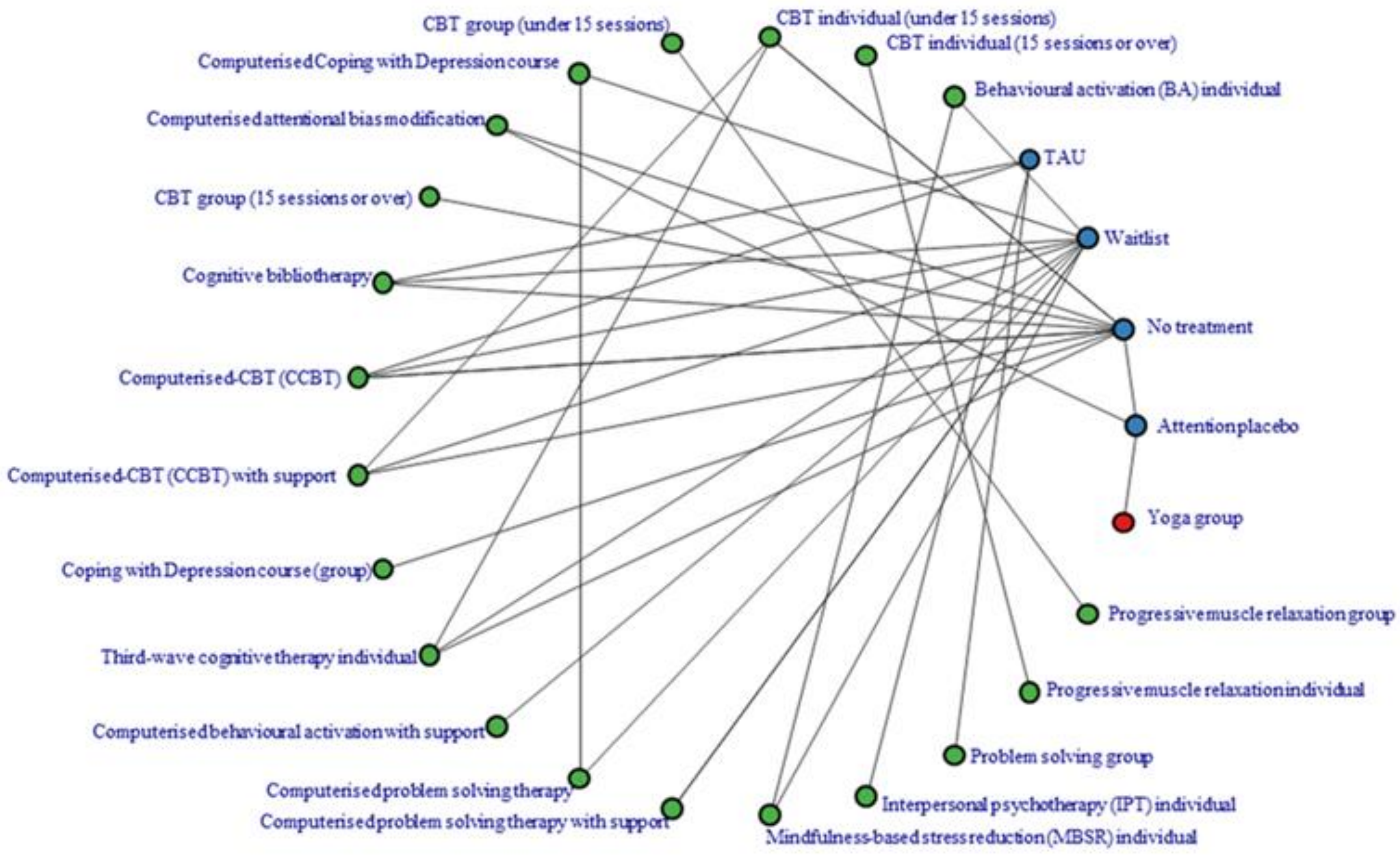

CBT: cognitive behavioural therapy
Without the use of a class network CBT group (under 15 sessions), CBT individual (15 sessions or over), Progressive muscle relaxation group and Progressive muscle relaxation individual would be disconnected from the rest of the network and would have to be excluded from the analysis.

Classes, interventions and numbers of participants tested on each

The NMA included 26 RCTs reporting dichotomous remission data, 25 interventions grouped in 16 treatment classes and 3,810 participants.

Treatment class	N	Intervention	N	Variance Sharing*
No treatment	751	No treatment	751	
Attention placebo	46	Attention placebo	46	
Waitlist	468	Waitlist	468	
TAU	437	TAU	437	
Self-help without/with minimal support	1,050	Cognitive bibliotherapy	287	1
		Computerised-CBT (CCBT)	559	
		Computerised attentional bias modification	28	
		Computerised Coping with Depression course	88	
		Computerised problem solving therapy	88	
Self-help with support	348	Computerised-CBT (CCBT) with support	184	1
		Computerised behavioural activation with support	40	
		Computerised problem solving therapy with support	124	
Behavioural therapies individual	16	Behavioural activation (BA) individual	16	1
Behavioural therapies group	68	Coping with Depression course (group)	68	1
CT/CBT individual	233	CBT individual (15 sessions or over)	12	1
		CBT individual (under 15 sessions)	116	
		Third-wave CT individual	105	
CT/CBT group	117	CBT group (15 sessions or over)	47	1
		CBT group (under 15 sessions)	70	
Problem solving group	89	Problem solving group	89	1
IPT individual	69	IPT individual	69	1
Mindfulness or meditation individual	20	Mindfulness-based stress reduction (MBSR) individual	20	1
Relaxation individual	15	Progressive muscle relaxation individual	15	1
Relaxation group	63	Progressive muscle relaxation group	63	1
Yoga group	20	Yoga group	20	1

CBT: cognitive behavioural therapy; CT: cognitive therapy; IPT: interpersonal psychotherapy; TAU: treatment as usual

** Classes with the same number share a common class variance*

Treatment discontinuation for any reason

Network - treatment class level

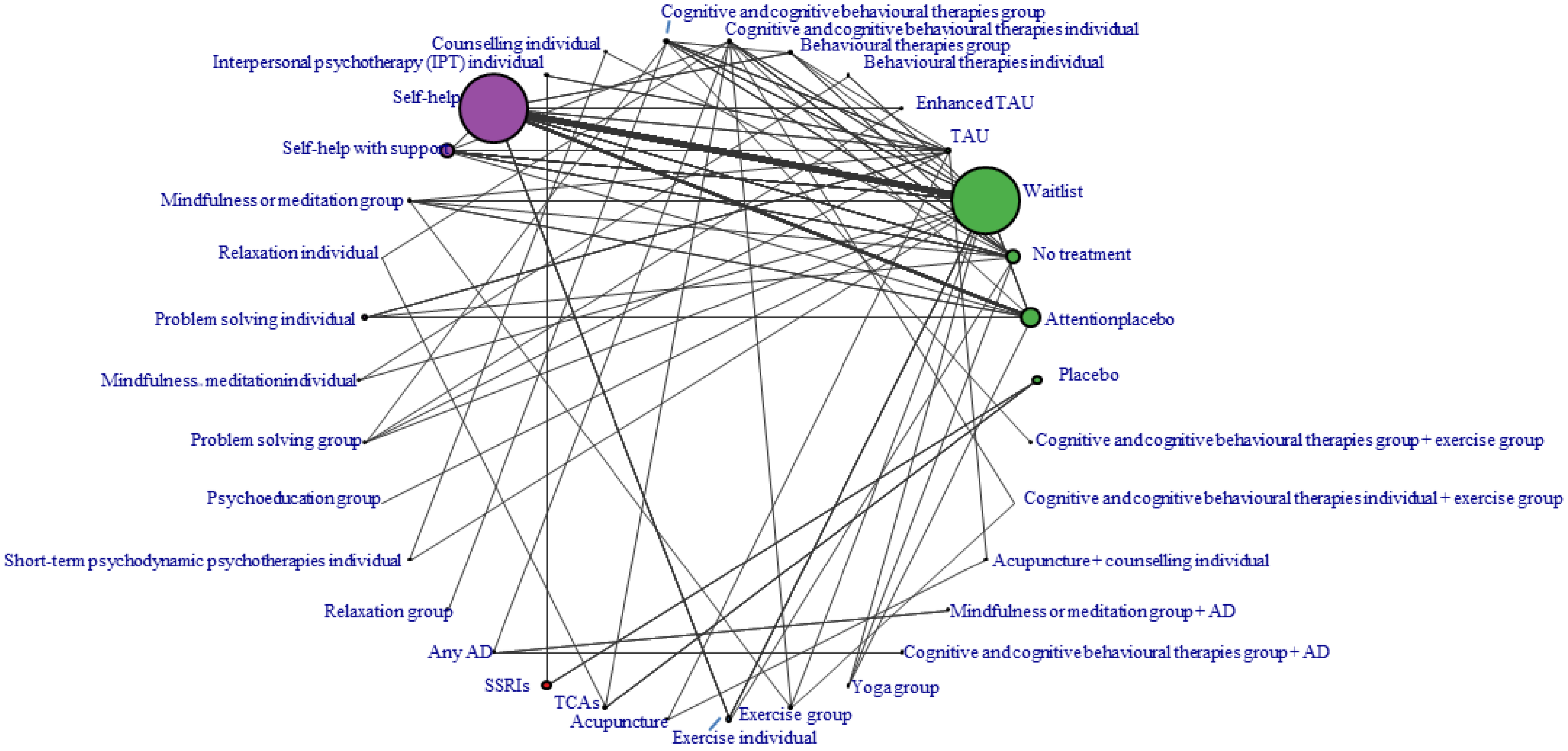

AD: antidepressant; SSRIs: selective serotonin uptake inhibitors; TAU: treatment as usual; TCAs: tricyclic antidepressants

Network - intervention level

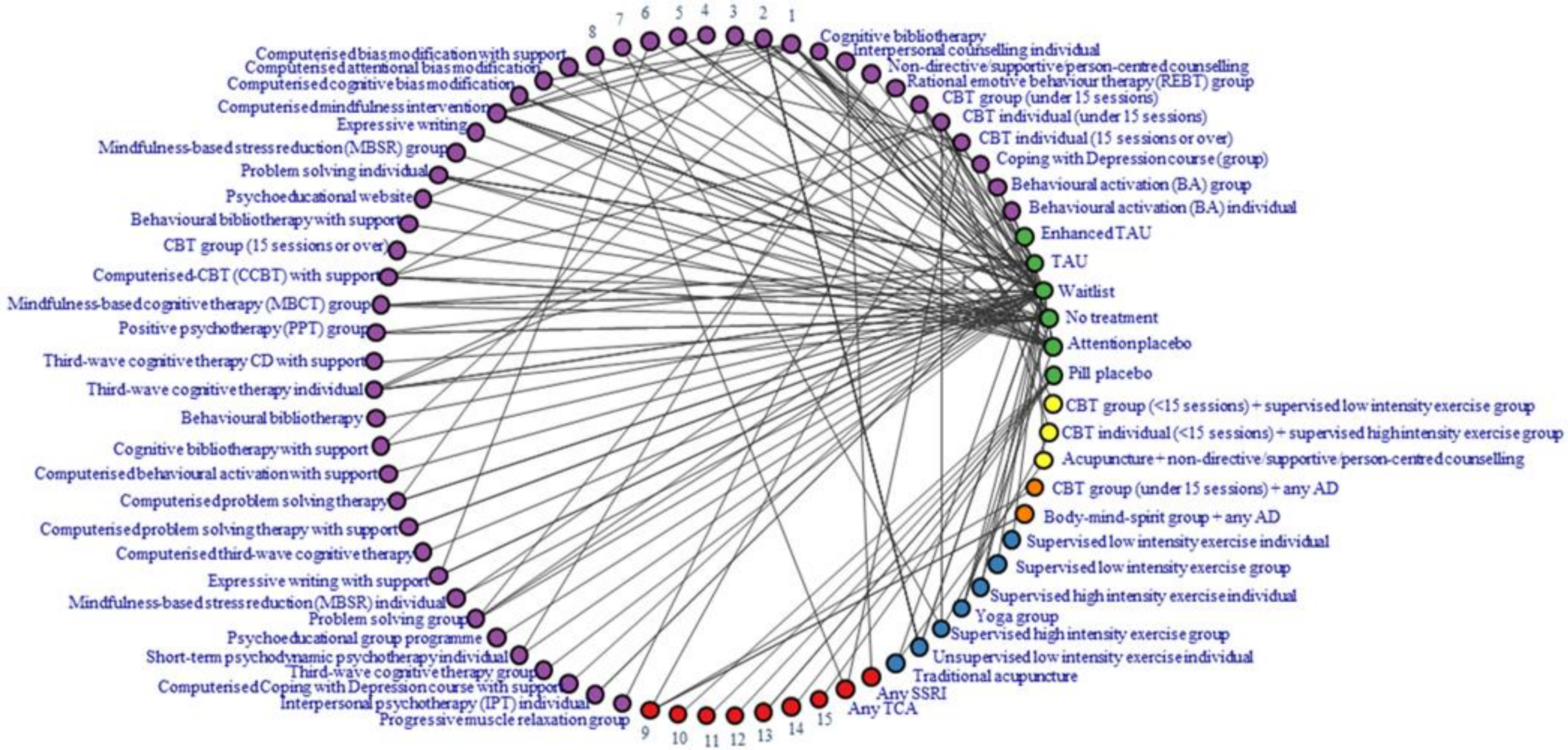

AD: antidepressant; CBT: cognitive behavioural therapy; CT: cognitive therapy; IPT: interpersonal psychotherapy; MBCT: mindfulness-based cognitive therapy; SSRI: selective serotonin uptake inhibitor; TAU: treatment as usual; TCA: tricyclic antidepressant

1 Computerised CBT (CCBT), 2 Computerised behavioural activation, 3 Computerised Coping with Depression course, 4 Computerised expressive writing, 5 Computerised positive psychological intervention, 6 Computerised third wave cognitive therapy with support, 7 Mindfulness meditation group, 8 Progressive muscle relaxation individual, 9 Any AD, 10 Amitriptyline, 11 Citalopram, 12 Fluoxetine, 13 Imipramine, 14 Lofepramine, 15 Sertraline.

Without the use of a class model Pill placebo, Interpersonal counselling individual, Amitriptyline, Any SSRI, Citalopram, Fluoxetine, Imipramine, Lofepramine and Sertraline would be disconnected from the rest of the network.

Classes, interventions and numbers of participants tested on each

The NMA included 120 RCTs, 75 interventions grouped in 34 treatment classes and 19,433 participants.

Treatment lass	N	Intervention	N	Variance Sharing*
Waitlist	3785	Waitlist	3785	
Placebo	621	Pill placebo	621	
Attention placebo	795	Attention placebo	795	
No treatment	1713	No treatment	1713	
TAU	1005	TAU	1005	
Enhanced TAU	96	Enhanced TAU	96	
Behavioural therapies individual	153	Behavioural activation (BA) individual	153	1
Behavioural therapies group	373	Behavioural activation (BA) group	107	1
		Coping with Depression course (group)	266	
CT/CBT individual	663	CBT individual (15 sessions or over)	90	1
		CBT individual (under 15 sessions)	402	
		Third-wave CT individual	171	
CT/CBT group	483	CBT group (15 sessions or over)	47	2
		CBT group (under 15 sessions)	283	
		Positive psychotherapy (PPT) group	89	
		Rational emotive behaviour therapy (REBT) group	15	
		Third-wave CT group	49	
Problem solving individual	159	Problem solving individual	159	1
Problem solving group	168	Problem solving group	168	1
Counselling individual	125	Non-directive/supportive/person-centred counselling	125	1
IPT individual	135	Interpersonal counselling individual	27	1
		IPT individual	108	
Psychoeducation group	23	Psychoeducational group programme	23	1
Self-help	5733	Behavioural bibliotherapy	13	3
		Cognitive bibliotherapy	427	
		Computerised-CBT (CCBT)	3173	
		Computerised attentional bias modification	154	
		Computerised behavioural activation	159	
		Computerised cognitive bias modification	76	
		Computerised Coping with Depression course	292	
		Computerised expressive writing	44	
		Computerised mindfulness intervention	645	
		Computerised positive psychological intervention	440	
		Computerised problem solving therapy	101	
		Computerised third-wave CT	31	
		Expressive writing	13	
		Psychoeducational website	165	
Self-help with support	1391	Behavioural bibliotherapy with support	67	4
		Cognitive bias modification with support	32	
		Cognitive bibliotherapy with support	125	

		Computerised-CBT (CCBT) with support	428	
		Computerised behavioural activation with support	41	
		Computerised Coping with Depression course with support	36	
		Computerised problem solving therapy with support	124	
		Computerised third-wave CT with support	82	
		Expressive writing with support	125	
		Third-wave CT CD with support	331	
Short-term PDPT individual	53	Short-term PDPT individual	53	1
Mindfulness or meditation individual	20	Mindfulness-based stress reduction (MBSR) individual	20	1
Mindfulness or meditation group	375	MBCT group	167	5
		Mindfulness-based stress reduction (MBSR) group	70	
		Mindfulness meditation group	138	
Relaxation individual	15	Progressive muscle relaxation individual	15	1
Relaxation group	63	Progressive muscle relaxation group	63	2
SSRIs	462	Any SSRI	28	6
		Citalopram	27	
		Fluoxetine	81	
		Sertraline	326	
TCAs	208	Amitriptyline	90	7
		Any TCA	13	
		Imipramine	73	
		Lofepramine	32	
Any AD	107	Any AD	107	8
Acupuncture	40	Traditional acupuncture	40	1
Exercise individual	235	Supervised high intensity exercise individual	39	9
		Supervised low intensity exercise individual	61	
		Unsupervised low intensity exercise individual	135	
Exercise group	181	Supervised high intensity exercise group	121	4
		Supervised low intensity exercise group	60	
Yoga group	78	Yoga group	78	2
CT/CBT group + AD	35	CBT group (under 15 sessions) + any AD	35	1
Mindfulness or meditation group + AD	44	Body-mind-spirit group + any AD	44	1
Acupuncture + counselling individual	40	Traditional acupuncture + non-directive/supportive/person-centred counselling	40	1
CT/CBT individual + exercise group	21	CBT individual (under 15 sessions) + supervised high intensity exercise group	21	1
CT/CBT group + exercise group	35	CBT group (under 15 sessions) + supervised low intensity exercise group	35	1

CBT: cognitive behavioural therapy; CT: cognitive therapy; IPT: interpersonal psychotherapy; MBCT: mindfulness-based cognitive therapy; PDPT: psychodynamic psychotherapy; SSRIs: selective serotonin uptake inhibitors; TAU: treatment as usual; TCAs: tricyclic antidepressants

** Classes with the same number share a common class variance*

Treatment discontinuation due to side effects from medication, in those who discontinued treatment

Network - treatment class level

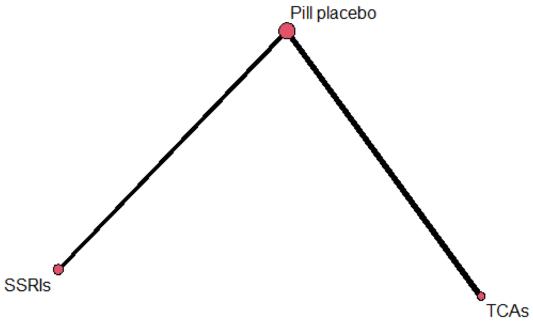

SSRIs: selective serotonin uptake inhibitors; TCAs: tricyclic antidepressants

Network - intervention level

There were insufficient studies and interventions available to be able to fit a NMA with random class effects. Therefore, a simpler fixed class model was fitted, in which all interventions within a class were assumed to have the same effect.

Classes, interventions and numbers of participants tested on each

The NMA included 5 RCTs, 5 interventions grouped in 3 treatment classes and 138 participants.

Class	N	Intervention	N	Variance Sharing*
Placebo	66	Pill placebo	66	
SSRIs	31	Fluoxetine	22	NA
		Citalopram	9	
TCAs	40	Amitriptyline	28	NA
		Imipramine	12	

NA: non-applicable
** non-applicable, as a fixed class model was used.*

Response in treatment completers

Network - treatment class level

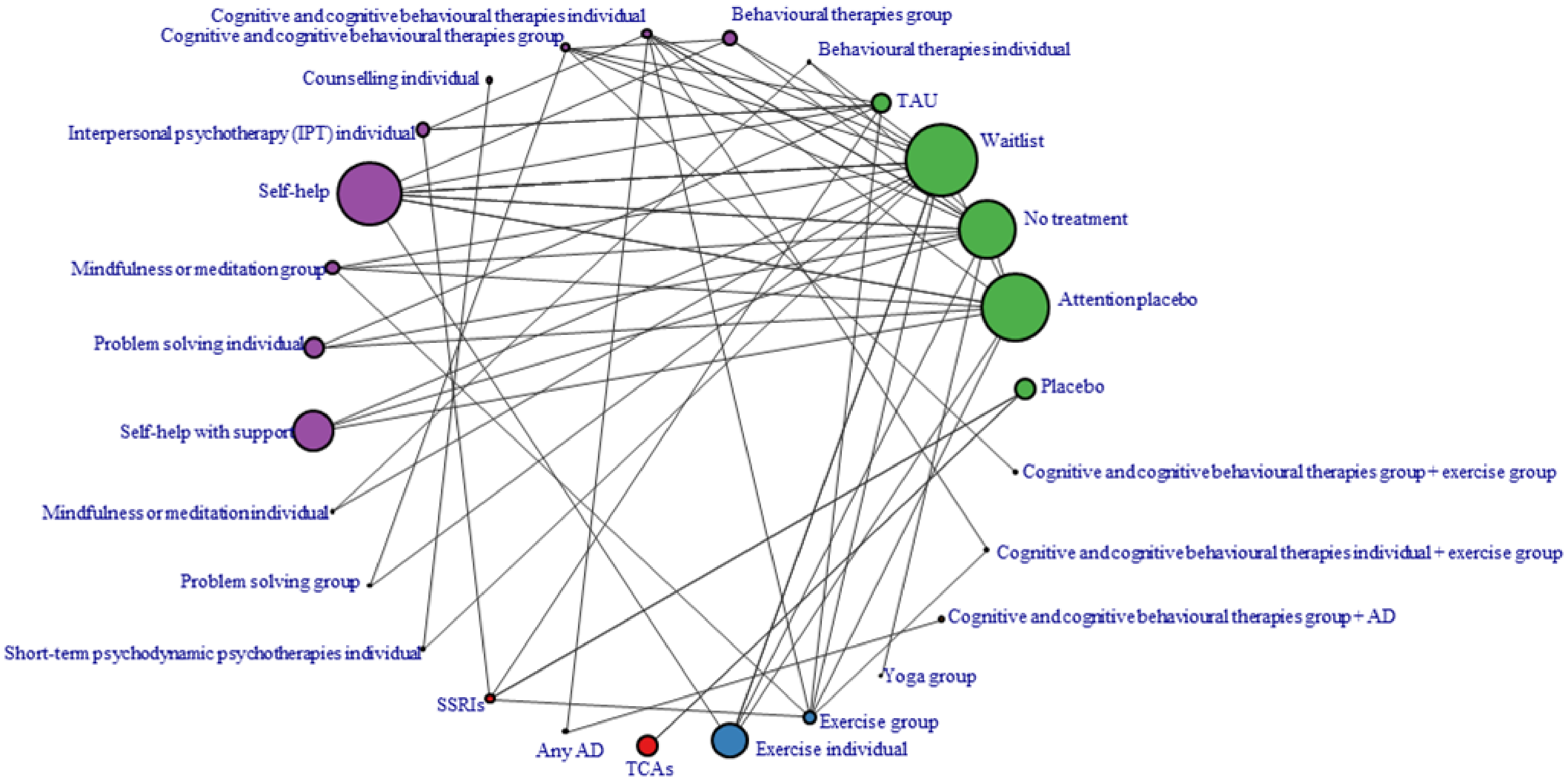

AD: antidepressant; SSRIs: selective serotonin uptake inhibitors; TAU: treatment as usual; TCAs: tricyclic antidepressants

Network - intervention level

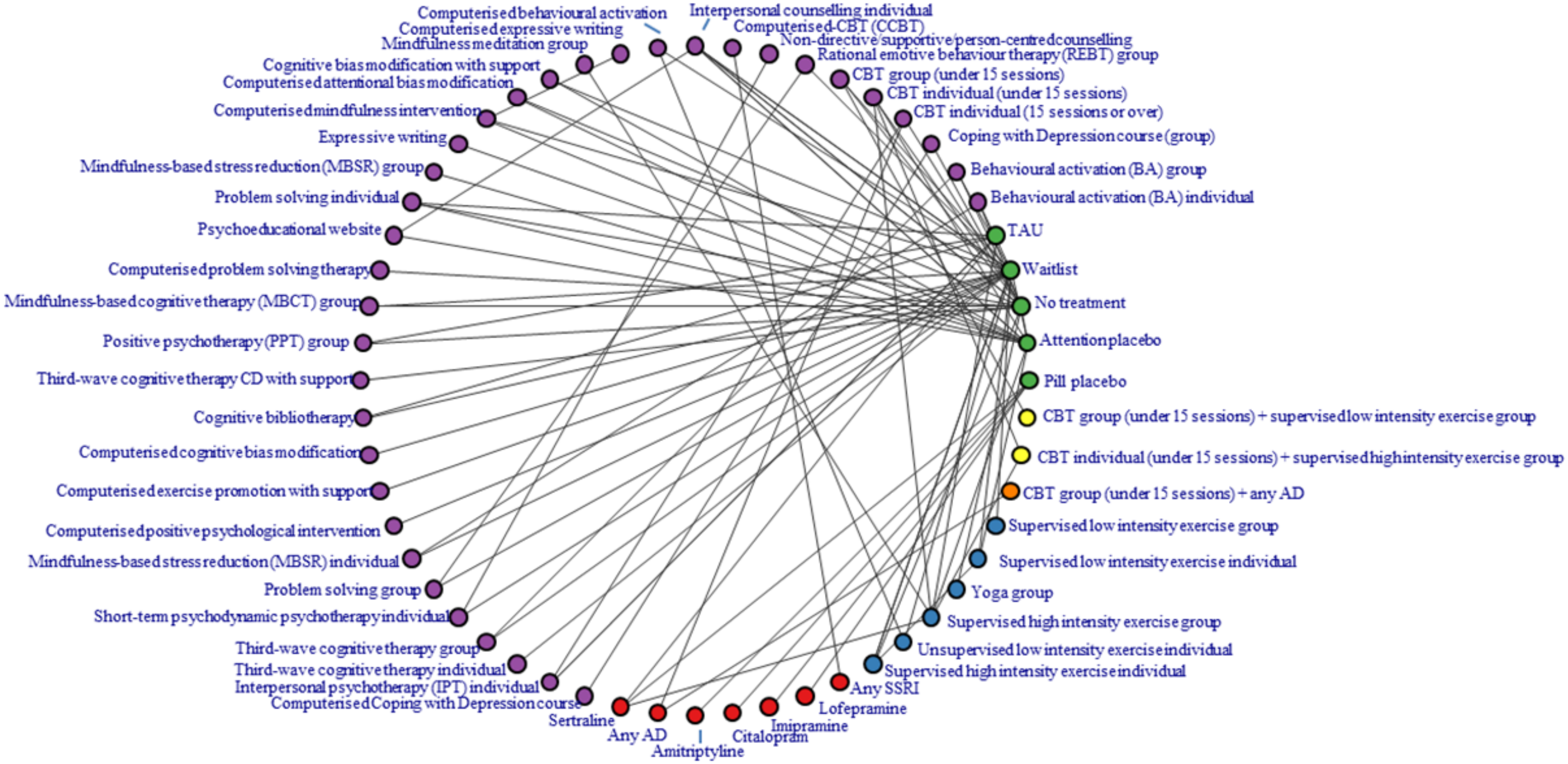

AD: antidepressant

Without the use of a class network Interpersonal counselling individual and Any SSRI would be disconnected from the rest of the network and would have to be excluded from the analysis.

Classes, interventions and numbers of participants tested on each
The NMA included 76 RCTs, 56 interventions grouped in 27 treatment classes and 6,788 participants. Of the 76 RCTs, 12 reported dichotomous response data, 8 reported CFB depression symptom score data; and 56 reported baseline and endpoint depression symptom score data.

Class	N	Intervention	N	Variance Sharing*
Waitlist	772	Waitlist	772	
Placebo	219	Pill placebo	219	
Attention placebo	417	Attention placebo	417	
No treatment	1033	No treatment	1033	
TAU	395	TAU	395	
Behavioural therapies individual	111	Behavioural activation (BA) individual	111	1
Behavioural therapies group	171	Behavioural activation (BA) group	47	1
		Coping with Depression course (group)	124	
CT/CBT individual	361	CBT individual (15 sessions or over)	68	1
		CBT individual (under 15 sessions)	233	
		Third-wave CT individual	60	
CT/CBT group	164	CBT group (under 15 sessions)	59	1
		Positive psychotherapy (PPT) group	76	
		Rational emotive behaviour therapy (REBT) group	14	
		Third-wave CT group	15	
Problem solving individual	98	Problem solving individual	98	1
Problem solving group	15	Problem solving group	15	1
Counselling individual	39	Non-directive/supportive/person-centred counselling	39	1
IPT individual	142	Interpersonal counselling individual	17	1
		IPT individual	125	
Self-help	1508	Cognitive bibliotherapy	137	2
		Computerised-CBT (CCBT)	607	
		Computerised attentional bias modification	76	
		Computerised behavioural activation	122	
		Computerised cognitive bias modification	20	
		Computerised Coping with Depression course	67	
		Computerised expressive writing	36	
		Computerised mindfulness intervention	174	
		Computerised positive psychological intervention	95	
		Computerised problem solving therapy	25	
		Expressive writing	13	
		Psychoeducational website	136	
Self-help with support	327	Cognitive bias modification with support	20	3
		Computerised exercise promotion with support	24	
		Third-wave CT CD with support	283	
Short-term PDPT individual	43	Short-term PDPT individual	43	1
Mindfulness or meditation individual	18	Mindfulness-based stress reduction (MBSR) individual	18	1
Mindfulness or meditation group	179	MBCT group	73	1
		Mindfulness-based stress reduction (MBSR) group	15	

		Mindfulness meditation group	91	
SSRIs	98	Any SSRI	24	4
		Citalopram	24	
		Sertraline	50	
TCAs	146	Amitriptyline	62	4
		Imipramine	61	
		Lofepramine	23	
Any AD	50	Any AD	50	4
Exercise individual	189	Supervised high intensity exercise individual	43	3
		Supervised low intensity exercise individual	25	
		Unsupervised low intensity exercise individual	121	
Exercise group	178	Supervised high intensity exercise group	136	3
		Supervised low intensity exercise group	42	
Yoga group	40	Yoga group	40	1
CT/CBT group + AD	32	CBT group (under 15 sessions) + any AD	32	1
CT/CBT individual + exercise group	18	CBT individual (under 15 sessions) + supervised high intensity exercise group	18	1
CT/CBT group + exercise group	25	CBT group (under 15 sessions) + supervised low intensity exercise group	25	1

CBT: cognitive behavioural therapy; CT: cognitive therapy; IPT: interpersonal psychotherapy; MBCT: mindfulness-based cognitive therapy; SSRIs: selective serotonin uptake inhibitors; TAU: treatment as usual; TCAs: tricyclic antidepressants
** Classes with the same number share a common class variance*

Remission in treatment completers

Network - treatment class level

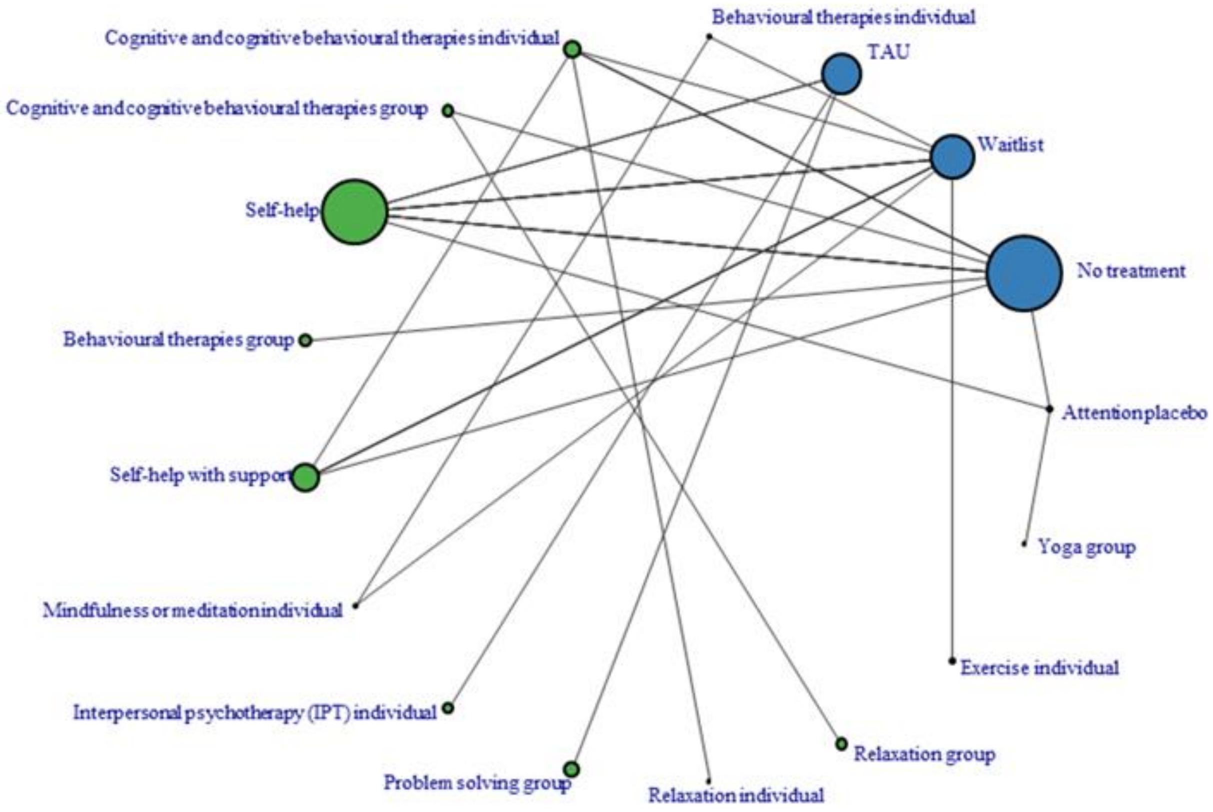

TAU: treatment as usual

Network - intervention level

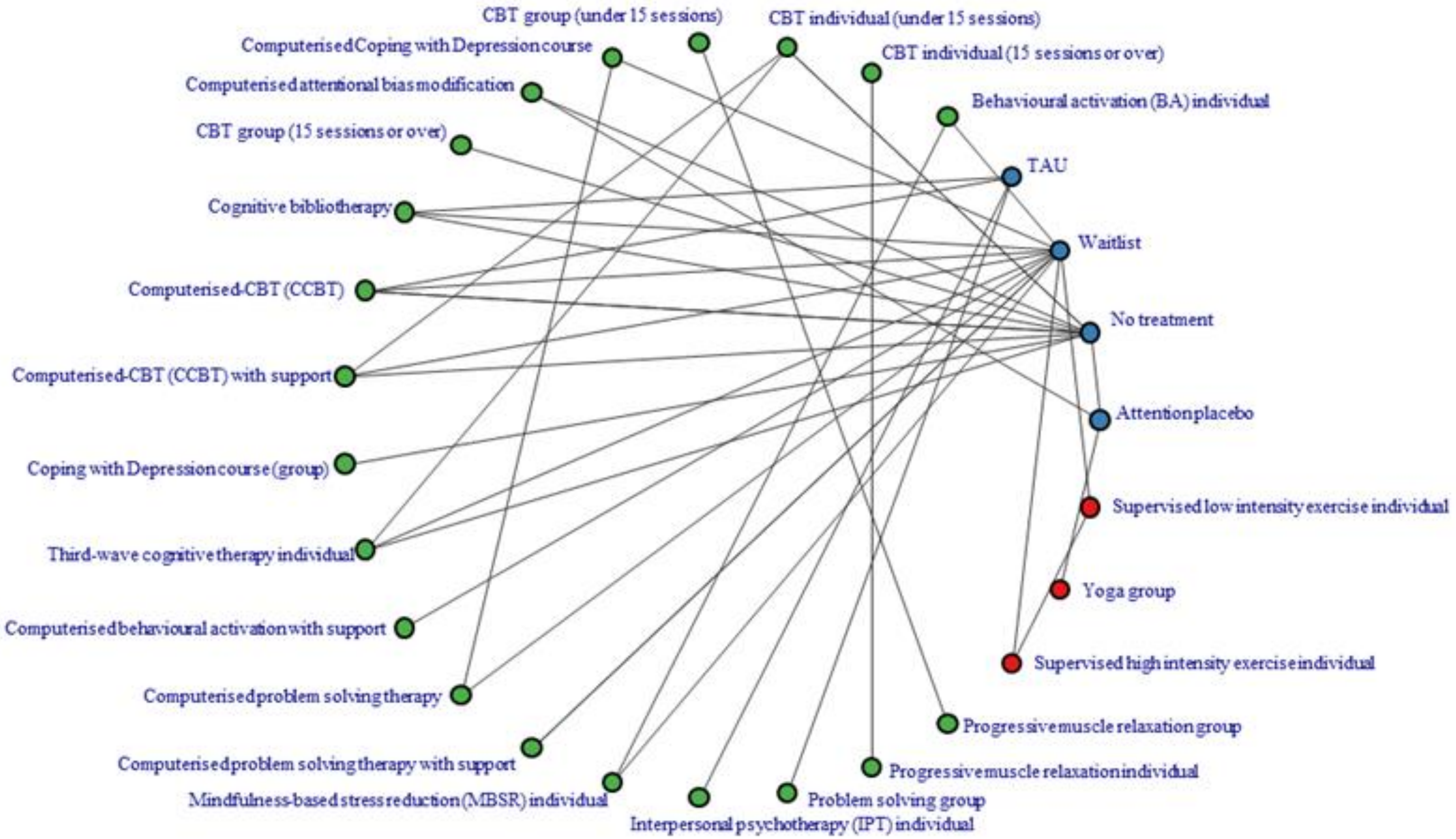

CBT: cognitive behavioural therapy; TAU: treatment as usual
Without the use of a class network CBT group (under 15 sessions), CBT individual (15 sessions or over), progressive muscle relaxation group and progressive muscle relaxation individual would be disconnected from the rest of the network and would have to be excluded from the analysis.

Classes, interventions and numbers of participants tested on each
The NMA included 27 RCTs, 27 interventions grouped in 17 treatment classes, and 3,208 participants.

Treatment class	N	Intervention	N	Variance Sharing*
Waitlist	414	Waitlist	414	
Attention placebo	38	Attention placebo	38	
No treatment	671	No treatment	671	
TAU	371	TAU	371	
Behavioural therapies individual	15	Behavioural activation (BA) individual	15	1
Behavioural therapies group	61	Coping with Depression course (group)	61	1
CT/CBT individual	194	CBT individual (15 sessions or over)	12	1
		CBT individual (under 15 sessions)	89	
		Third-wave CT individual	93	
CT/CBT group	107	CBT group (15 sessions or over)	42	1
		CBT group (under 15 sessions)	65	
Problem solving group	86	Problem solving group	86	1
IPT individual	58	IPT individual	58	1
Self-help	795	Cognitive bibliotherapy	205	2
		Computerised-CBT (CCBT)	460	
		Computerised attentional bias modification	28	
		Computerised Coping with Depression course	51	
		Computerised problem solving therapy	51	
Self-help with support	263	Computerised-CBT (CCBT) with support	133	1
		Computerised behavioural activation with support	40	
		Computerised problem solving therapy with support	90	
Mindfulness or meditation individual	18	Mindfulness-based stress reduction (MBSR) individual	18	1
Relaxation individual	12	Progressive muscle relaxation individual	12	1
Relaxation group	61	Progressive muscle relaxation group	61	1
Exercise individual	29	Supervised high intensity exercise individual	14	1
		Supervised low intensity exercise individual	15	
Yoga group	15	Yoga group	15	1

CBT: cognitive behavioural therapy; CT: cognitive therapy; IPT: interpersonal psychotherapy; PDPT: psychodynamic psychotherapy; SSRIs: selective serotonin uptake inhibitors; TAU: treatment as usual; TCAs: tricyclic antidepressants
** Classes with the same number share a common class variance*

More severe depression
SMD of depressive symptom scale change scores

Network - treatment class level

See Figure 2 in main article

Network - intervention level

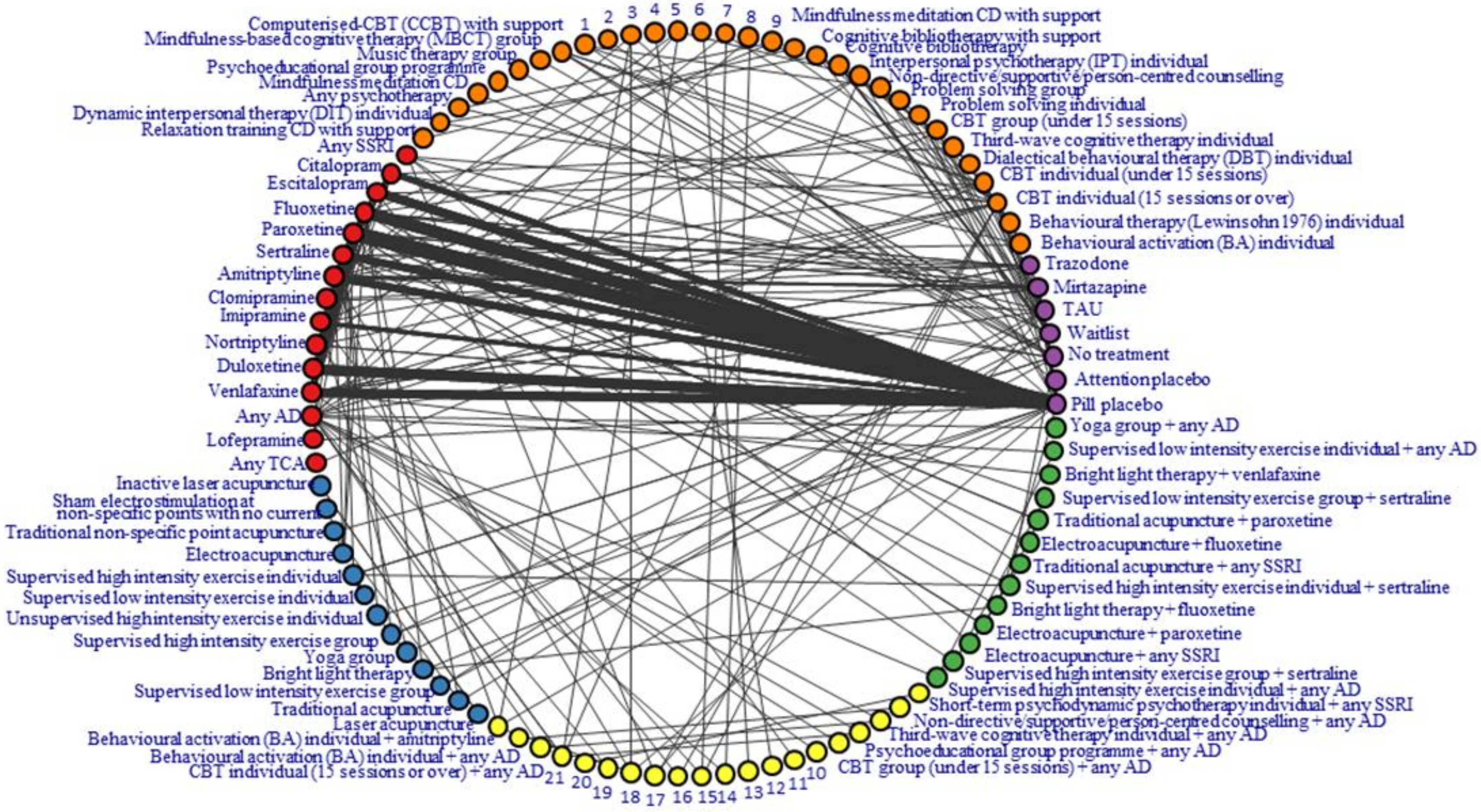

AD: antidepressant; CBT: cognitive behavioural therapy; IPT: interpersonal psychotherapy; MBCT: mindfulness-based cognitive therapy; SSRI: selective serotonin uptake inhibitor; TAU: treatment as usual; TCA: tricyclic antidepressant

1 Computerised-CBT (C-CBT); 2 Computerised attentional bias modification; 3 Progressive muscle relaxation individual + pill placebo; 4 Non-directive/supportive/person-centred counselling + pill placebo; 5 Interpersonal psychotherapy individual + pill placebo; 6 CBT individual (<15 sessions) + pill placebo; 7 CBT individual (≥15 sessions) + pill placebo; 8 Peer support group; 9 Short-term psychodynamic psychotherapy individual; 10 Interpersonal counselling individual + venlafaxine; 11 CBT individual (<15 sessions) + escitalopram; 12 Non-directive/supportive/person-centred counselling + fluoxetine; 13 CBT individual (<15 sessions) + sertraline; 14 Peer support group + any AD; 15 CBT individual (≥15 sessions) + nortriptyline; 16 CBT individual (≥15 sessions) + imipramine; 17 CBT individual (≥15 sessions) + any SSRI; 18 Progressive muscle relaxation therapy + amitriptyline; 19 Short-term psychodynamic psychotherapy individual + any AD; 20 Non-directive/supportive/person-centred counselling + any SSRI; 21 Interpersonal psychotherapy individual + any AD

Without the use of a class network the following interventions would be disconnected from the rest of the network and would have to be excluded from the analysis: Mindfulness meditation CD with support, CBT individual (<15 sessions) + pill placebo, Non-directive/supportive/person-centred counselling + pill placebo, Inactive laser acupuncture, Behavioural activation individual + any AD, Non-directive/supportive/person-centred counselling + any SSRI, Relaxation training CD with support, CBT individual (<15 sessions) + sertraline, Non-directive/supportive/person-centred counselling + fluoxetine, Laser acupuncture, Non-directive/supportive/person-centred counselling + any AD, and Short-term psychodynamic psychotherapy individual + any SSRI

Classes, interventions and numbers of participants tested on each

The NMA included 352 RCTs, 99 interventions grouped in 50 treatment classes, and 59,350 participants. Of the 352 RCTs, 146 reported change from baseline (CFB) depression symptom score data; 172 reported baseline and endpoint depression symptom score data; and 34 reported dichotomous response data and baseline symptom scores.

Treatment class	N	Intervention	N	Variance Sharing*
Pill placebo	12,554	Pill placebo	12,554	
Attention placebo	61	Attention placebo	61	
No treatment	504	No treatment	504	
Waitlist	526	Waitlist	526	
TAU	220	TAU	220	
Sham acupuncture	108	Inactive laser acupuncture	34	1
		Sham electrostimulation at non-specific points with no current	22	
		Traditional non-specific point acupuncture	52	
Self-help without or with minimal support	344	Cognitive bibliotherapy	159	2
		Computerised-CBT (CCBT)	120	
		Computerised attentional bias modification	26	
		Mindfulness meditation CD	39	
Self-help with support	267	Cognitive bibliotherapy with support	66	3
		Computerised-CBT (CCBT) with support	164	
		Mindfulness meditation CD with support	19	
		Relaxation training CD with support	18	
Behavioural therapies individual	378	Behavioural activation (BA) individual	368	4
		Behavioural therapy (Lewinsohn 1976) individual	10	
CT/CBT individual	1,044	CBT individual (15 sessions or over)	626	4
		CBT individual (under 15 sessions)	369	
		Dialectical behavioural therapy (DBT) individual	10	
		Third-wave CT individual	39	
CT/CBT group	165	CBT group (under 15 sessions)	165	4
Problem solving individual	367	Problem solving individual	367	4
Problem solving group	47	Problem solving group	47	4
Counselling individual	404	Non-directive/supportive/person-centred counselling	404	4
IPT individual	146	IPT individual	146	4
Short-term PDPT individual	233	Dynamic interpersonal therapy (DIT) individual	73	4
		Short-term PDPT individual	160	
Psychoeducation group	44	Psychoeducational group programme	44	4
Music therapy group	12	Music therapy group	12	4
Mindfulness or meditation group	15	MBCT group	15	4
Peer support group	39	Peer support group	39	4
Any psychotherapy	37	Any psychotherapy	37	4
CT/CBT individual + pill placebo	61	CBT individual (15 sessions or over) + pill placebo	17	4
		CBT individual (under 15 sessions) + pill placebo	44	
IPT + pill placebo	69	IPT individual + pill placebo	69	4
Counselling individual + pill placebo	26	Non-directive/supportive/person-centred counselling + pill placebo	26	4
Relaxation individual + pill placebo	11	Progressive muscle relaxation individual + pill placebo	11	4
SSRIs	22,018	Any SSRI	207	5

		Citalopram	2,195	
		Escitalopram	4,930	
		Fluoxetine	6,031	
		Paroxetine	5,861	
		Sertraline	2,794	
TCAs	4,524	Amitriptyline	2,462	6
		Any TCA	21	
		Clomipramine	345	
		Imipramine	1,306	
		Lofepramine	145	
		Nortriptyline	245	
SNRIs	9,538	Duloxetine	5,269	5
		Venlafaxine	4,269	
Mirtazapine	1,884	Mirtazapine	1,884	
Trazodone	1,072	Trazodone	1,072	
Any AD	452	Any AD	452	7
Acupuncture	264	Electroacupuncture	110	8
		Laser acupuncture	39	
		Traditional acupuncture	115	
Exercise individual	298	Supervised high intensity exercise individual	128	9
		Supervised low intensity exercise individual	117	
		Unsupervised high intensity exercise individual	53	
Exercise group	106	Supervised high intensity exercise group	69	3
		Supervised low intensity exercise group	37	
Yoga group	65	Yoga group	65	4
Light therapy	32	Bright light therapy	32	8
Behavioural therapies individual + AD	22	Behavioural activation (BA) individual + amitriptyline	12	10
		Behavioural activation (BA) individual + any AD	10	
CT/CBT individual + AD	192	CBT individual (15 sessions or over) + any AD	10	10
		CBT individual (15 sessions or over) + any SSRI	43	
		CBT individual (15 sessions or over) + imipramine	25	
		CBT individual (15 sessions or over) + nortriptyline	18	
		CBT individual (under 15 sessions) + escitalopram	48	
		CBT individual (under 15 sessions) + sertraline	38	
		Third-wave CT individual + any AD	10	
CT/CBT group + AD	63	CBT group (under 15 sessions) + any AD	63	10
IPT individual + AD	99	IPT individual + any AD	87	10
		Interpersonal counselling individual + venlafaxine	12	
Counselling individual + AD	57	Non-directive/supportive/person-centred counselling + any AD	15	11
		Non-directive/supportive/person-centred counselling + any SSRI	17	
		Non-directive/supportive/person-centred counselling + fluoxetine	25	
Short-term PDPT individual + AD	131	Short-term PDPT individual + any AD	113	10
		Short-term PDPT individual + any SSRI	18	
Psychoeducation group + AD	27	Psychoeducational group programme + any AD	27	10

Peer support group + AD	42	Peer support group + any AD	42	10
Relaxation individual + AD	10	Progressive muscle relaxation individual + amitriptyline	10	10
Exercise individual + AD	40	Supervised high intensity exercise individual + any AD	14	12
		Supervised high intensity exercise individual + sertraline	15	
		Supervised low intensity exercise individual + any AD	11	
Exercise group + AD	79	Supervised high intensity exercise group + sertraline	42	10
		Supervised low intensity exercise group + sertraline	37	
Yoga group + AD	15	Yoga group + any AD	15	10
Acupuncture + AD	584	Electroacupuncture + any SSRI	160	13
		Electroacupuncture + fluoxetine	46	
		Electroacupuncture + paroxetine	71	
		Traditional acupuncture + any SSRI	206	
		Traditional acupuncture + paroxetine	101	
Light therapy + AD	54	Bright light therapy + fluoxetine	29	8
		Bright light therapy + venlafaxine	25	

AD: antidepressant; CBT: cognitive behavioural therapy; CT: cognitive therapy; IPT: interpersonal psychotherapy; MBCT: mindfulness-based cognitive therapy; PDPT: psychodynamic psychotherapy; SNRIs: serotonin and norepinephrine reuptake inhibitors; SSRIs: selective serotonin uptake inhibitors; TAU: treatment as usual; TCAs: tricyclic antidepressants
** Classes with the same number share a common class variance*

Response in those randomised

Network - treatment class level

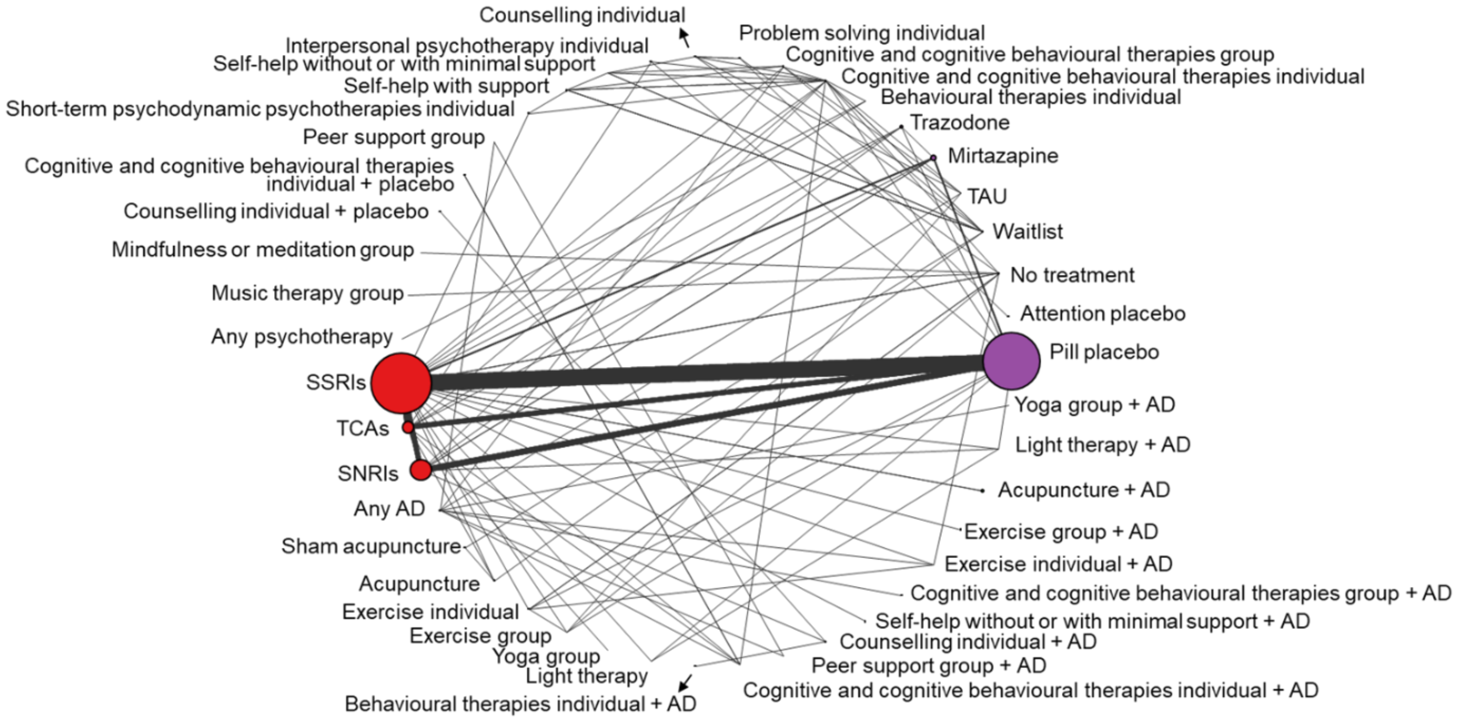

AD: antidepressant; CBT: cognitive behavioural therapy; SNRIs: serotonin and norepinephrine reuptake inhibitors; SSRIs: selective serotonin uptake inhibitors; TAU: treatment as usual; TCAs: tricyclic antidepressants

Network - intervention level

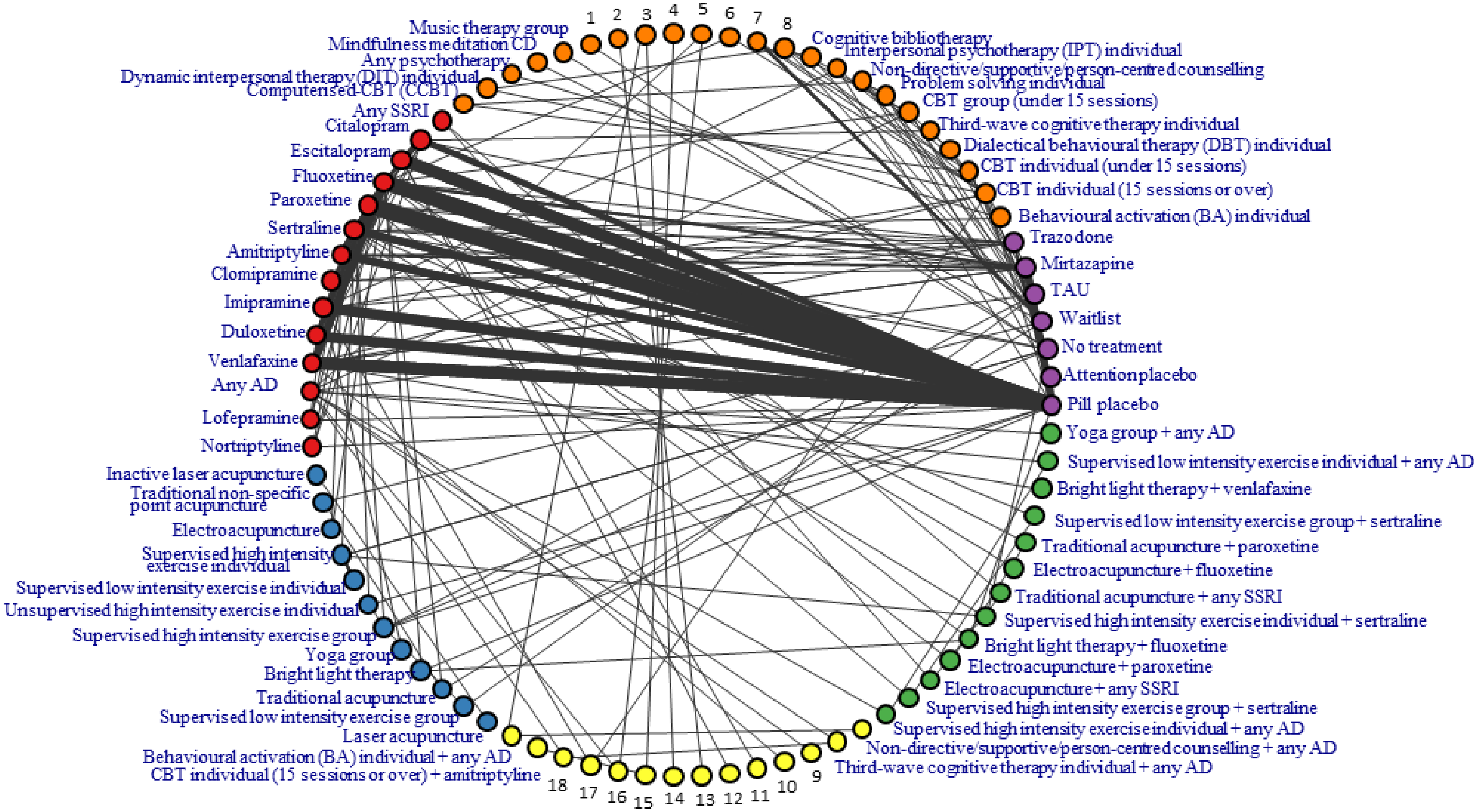

1 Mindfulness-based cognitive therapy (MBCT) group; 2 Non-directive/supportive/person-centred counselling + pill placebo; 3 CBT individual (under 15 sessions) + pill placebo; 4 CBT individual (15 sessions or over) + pill placebo; 5 Peer support; 6 Short-term psychodynamic therapy individual; 7 Computerised-CBT (CCBT) with support; 8 Cognitive bibliotherapy with support; 9 CBT group (under 15 sessions) + any AD; 10 Interpersonal counselling individual + venlafaxine; 11 Cognitive bibliotherapy + escitalopram; 12 CBT individual (under 15 sessions) + citalopram; 13 Non-directive/supportive/person-centred counselling + fluoxetine; 14 CBT individual (under 15 sessions) + sertraline; 15 Peer support group + any AD; 16 CBT individual (15 sessions or over) + trazodone; 17 CBT individual (15 sessions or over) + imipramine; 18 CBT individual (15 sessions or over) + any AD

Without the use of a class network the following interventions would be disconnected from the rest of the network and would have to be excluded from the analysis: CBT individual (15 sessions or over) + pill placebo, CBT individual (under 15 sessions) + pill placebo, Non-directive/supportive/person-centred counselling + pill placebo, Any SSRI, Inactive laser acupuncture, Behavioural activation (BA) individual + any AD, CBT individual (15 sessions or over) + amitriptyline, Electroacupuncture + any SSRI, CBT individual (15 sessions or over) + trazodone, CBT individual (under 15 sessions) + sertraline, Non-directive/supportive/person-centred counselling + fluoxetine, Traditional acupuncture + any SSRI, Laser acupuncture, and Non-directive/supportive/person-centred counselling + any AD

Classes, interventions and numbers of participants tested on each

The NMA included 364 RCTs, 83 interventions grouped in 43 treatment classes and 68,073 participants. Of the 364 RCTs, 280 reported dichotomous response data, 31 reported CFB depression symptom score data; and 53 reported baseline and endpoint depression symptom score data.

Treatment class	N	Intervention	N	Variance Sharing*
Pill placebo	15,384	Pill placebo	15,384	
Attention placebo	36	Attention placebo	36	
No treatment	441	No treatment	441	
Waitlist	349	Waitlist	349	
TAU	176	TAU	176	
Sham acupuncture	74	Inactive laser acupuncture	22	6
		Traditional non-specific point acupuncture	52	
Self-help without or with minimal support	168	Cognitive bibliotherapy	32	2
		Computerised-CBT (CCBT)	97	
		Mindfulness meditation CD	39	
Self-help with support	274	Cognitive bibliotherapy with support	66	1
		Computerised-CBT (CCBT) with support	208	
Behavioural therapies individual	368	Behavioural activation (BA) individual	368	1
CT/CBT individual	779	CBT individual (15 sessions or over)	470	1
		CBT individual (under 15 sessions)	260	
		Dialectical behavioural therapy (DBT) individual	10	
		Third-wave CT individual	39	
CT/CBT group	155	CBT group (under 15 sessions)	155	1
Problem solving individual	338	Problem solving individual	338	1
Counselling individual	421	Non-directive/supportive/person-centred counselling	421	1
IPT individual	61	IPT individual	61	1
Short-term PDPT individual	217	Dynamic interpersonal therapy (DIT) individual	73	1
		Short-term PDPT individual	144	
Music therapy group	12	Music therapy group	12	1
Mindfulness or meditation group	15	MBCT group	15	1
Peer support group	39	Peer support group	39	1
Any psychotherapy	22	Any psychotherapy	22	1
CT/CBT + pill placebo	58	CBT individual (15 sessions or over) + pill placebo	14	1
		CBT individual (under 15 sessions) + pill placebo	44	
Counselling individual + pill placebo	26	Non-directive/supportive/person-centred counselling + pill placebo	26	1
SSRIs	26,961	Any SSRI	156	3
		Citalopram	3,242	
		Escitalopram	5,863	
		Fluoxetine	7,732	
		Paroxetine	6,661	
		Sertraline	3,307	
TCAs	54,37	Amitriptyline	2,519	4
		Clomipramine	414	
		Imipramine	2,061	
		Lofepramine	242	
		Nortriptyline	201	
SNRIs	10,469	Duloxetine	5,472	3
		Venlafaxine	4,997	
Mirtazapine	2,629	Mirtazapine	2,629	

Trazodone	1,181	Trazodone	1,181	
Any AD	188	Any AD	188	5
Acupuncture	217	Electroacupuncture	77	6
		Laser acupuncture	25	
		Traditional acupuncture	115	
Exercise individual	273	Supervised high intensity exercise individual	114	7
		Supervised low intensity exercise individual	106	
		Unsupervised high intensity exercise individual	53	
Exercise group	126	Supervised high intensity exercise group	106	1
		Supervised low intensity exercise group	20	
Yoga group	45	Yoga group	45	1
Light therapy	32	Bright light therapy	32	6
Behavioural therapies individual + AD	10	Behavioural activation (BA) individual + any AD	10	8
CT/CBT individual + AD	158	CBT individual (15 sessions or over) + amitriptyline	12	8
		CBT individual (15 sessions or over) + any AD	10	
		CBT individual (15 sessions or over) + imipramine	25	
		CBT individual (15 sessions or over) + trazodone	11	
		CBT individual (under 15 sessions) + escitalopram	52	
		CBT individual (under 15 sessions) + sertraline	38	
		Third-wave CT individual + any AD	10	
CT/CBT group + AD	20	CBT group (under 15 sessions) + any AD	20	8
Counselling individual + AD	52	Interpersonal counselling individual + venlafaxine	12	8
		Non-directive/supportive/person-centred counselling + any AD	15	
		Non-directive/supportive/person-centred counselling + fluoxetine	25	
Self-help + AD	79	Cognitive bibliotherapy + escitalopram	79	8
Peer support group + AD	42	Peer support group + any AD	42	8
Exercise individual + AD	40	Supervised high intensity exercise individual + any AD	14	8
		Supervised high intensity exercise individual + sertraline	15	
		Supervised low intensity exercise individual + any AD	11	
Exercise group + AD	79	Supervised high intensity exercise group + sertraline	42	8
		Supervised low intensity exercise group + sertraline	37	
Yoga group + AD	15	Yoga group + any AD	15	8
Acupuncture + AD	553	Electroacupuncture + any SSRI	160	9
		Electroacupuncture + fluoxetine	48	
		Electroacupuncture + paroxetine	80	
		Traditional acupuncture + any SSRI	161	
		Traditional acupuncture + paroxetine	104	
Light therapy + AD	54	Bright light therapy + fluoxetine	29	6
		Bright light therapy + venlafaxine	25	

AD: antidepressant; CBT: cognitive behavioural therapy; CT: cognitive therapy; IPT: interpersonal psychotherapy; MBCT: mindfulness-based cognitive therapy; PDPT: psychodynamic psychotherapy; SNRIs: serotonin and norepinephrine reuptake inhibitors; SSRIs: selective serotonin uptake inhibitors; TAU: treatment as usual; TCAs: tricyclic antidepressants
** Classes with the same number share a common class variance*

Remission in those randomised

Network - treatment class level

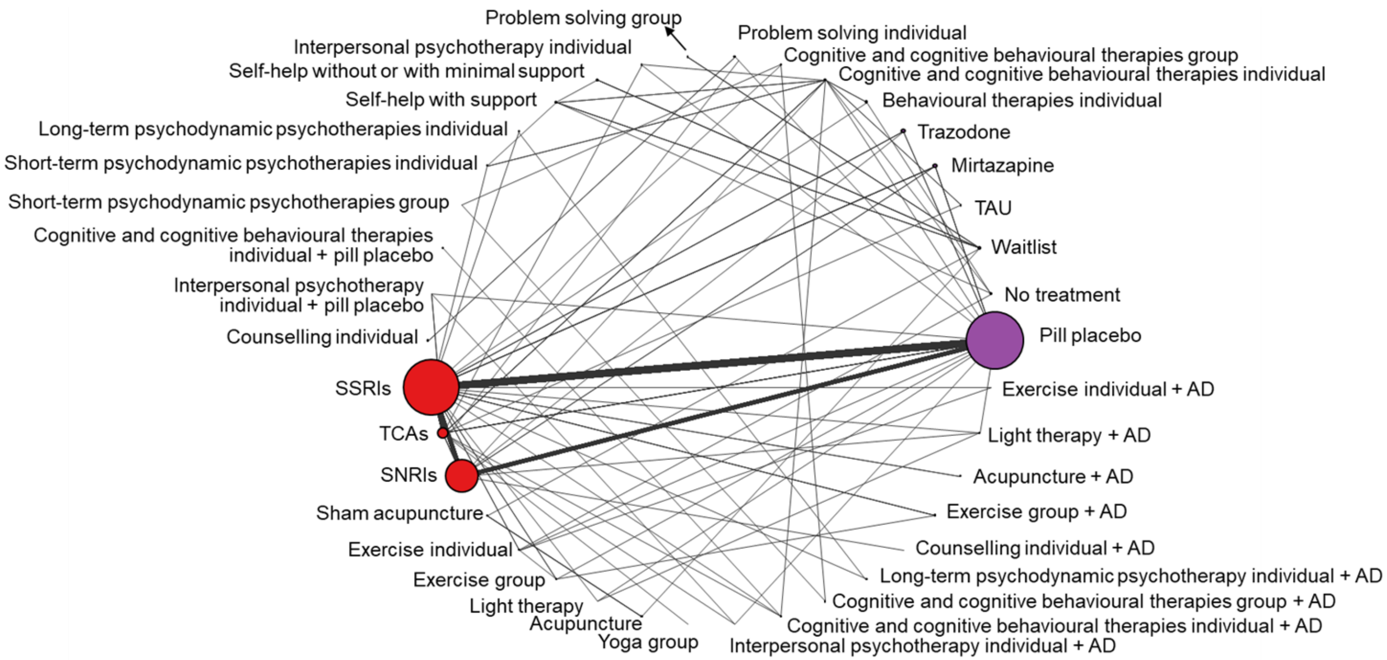

AD: antidepressant; SNRIs: serotonin and norepinephrine reuptake inhibitors; SSRIs: selective serotonin uptake inhibitors; TAU: treatment as usual; TCAs: tricyclic antidepressants

Network - intervention level

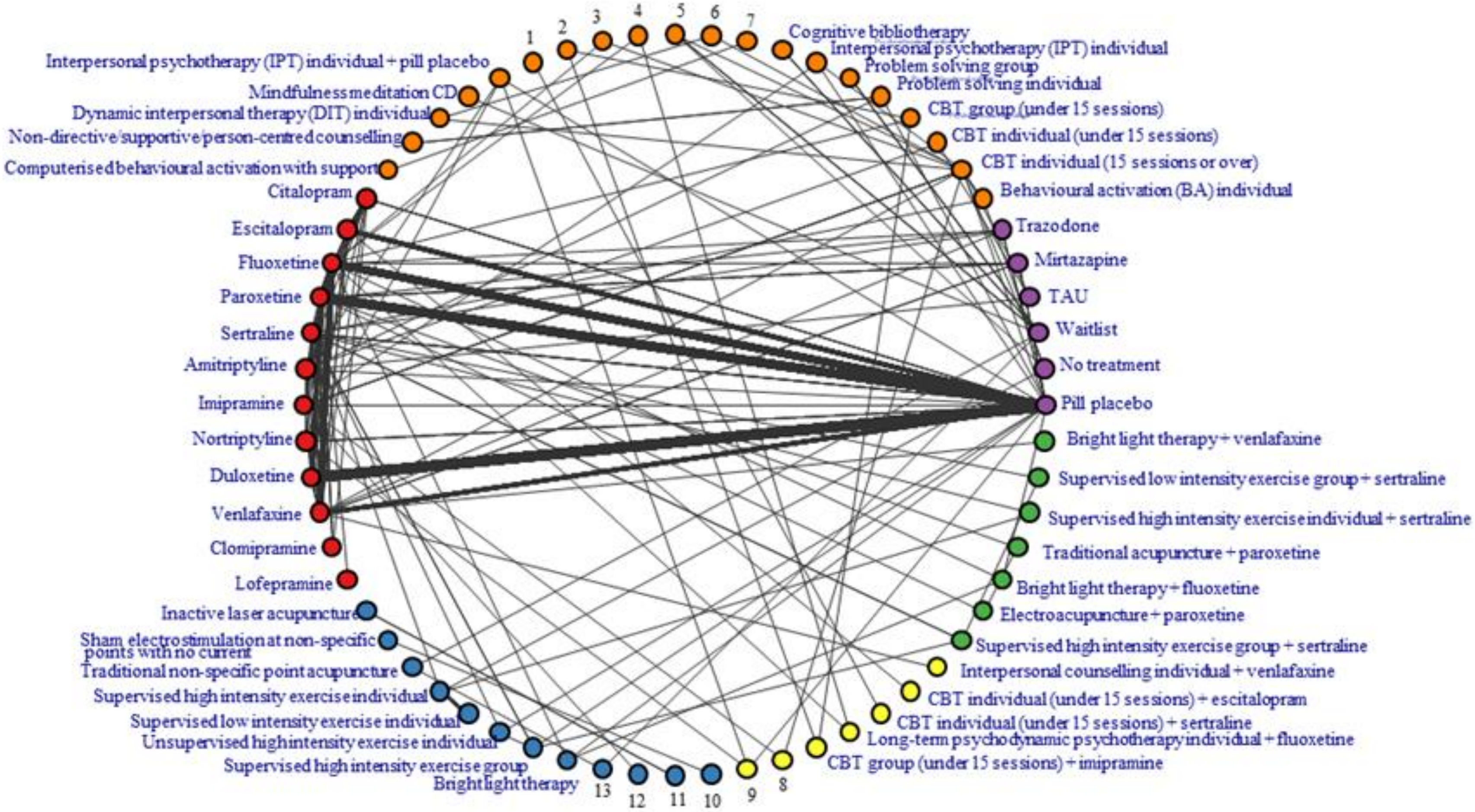

1 CBT individual (under 15 sessions) + pill placebo; 2 Short-term psychodynamic therapy group; 3 Short term psychodynamic psychotherapy individual; 4 Long-term psychodynamic psychotherapy individual; 5 Computerised CBT (CCBT) with support; 6 Cognitive bibliotherapy with support; 7 Psychoeducational website; 8 CBT individual (15 sessions or over) + imipramine; 9 Interpersonal psychotherapy (IPT) individual + nortriptyline; 10 Electroacupuncture; 11 Laser acupuncture; 12 Yoga therapy; 13 Traditional acupuncture
Without the use of a class network the following interventions would be disconnected from the rest of the network and would have to be excluded from the analysis: Psychoeducational website, CBT individual (under 15 sessions) + pill placebo, Inactive laser acupuncture, Sham electrostimulation at non-specific points with no current, Computerised behavioural activation with support, CBT individual (under 15 sessions) + sertraline, Laser acupuncture, and Electroacupuncture

Classes, interventions and numbers of participants tested on each
The NMA included 202 RCTs reporting dichotomous remission data, 64 interventions grouped in 38 treatment classes and 40,066 participants.

Treatment class	N	Intervention	N	Variance Sharing*
Pill placebo	8,376	Pill placebo	8,376	
No treatment	353	No treatment	353	
Waitlist	338	Waitlist	338	
TAU	60	TAU	60	
Sham acupuncture	117	Inactive laser acupuncture	36	1
		Sham electrostimulation at non-specific points with no current	29	
		Traditional non-specific point acupuncture	52	
Self-help without or with minimal support	349	Cognitive bibliotherapy	156	1
		Mindfulness meditation CD	39	
		Psychoeducational website	154	
Self-help with support	416	Cognitive bibliotherapy with support	54	1
		Computerised-CBT (CCBT) with support	203	
		Computerised behavioural activation with support	159	
Behavioural therapies individual	354	Behavioural activation (BA) individual	354	1
CT/CBT individual	451	CBT individual (15 sessions or over)	421	1
		CBT individual (under 15 sessions)	30	
CT/CBT group	65	CBT group (under 15 sessions)	65	1
Problem solving individual	232	Problem solving individual	232	1
Problem solving group	58	Problem solving group	58	1
Counselling individual	124	Non-directive/supportive/person-centred counselling	124	1
IPT individual	63	IPT individual	63	1
Long-term PDPT individual	90	Long-term PDPT individual	90	1
Short-term PDPT individual	129	Dynamic interpersonal therapy (DIT) individual	73	1
		Short-term PDPT individual	56	
Short-term PDPT group	24	Short-term PDPT group	24	1
CT/CBT individual + pill placebo	39	CBT individual (under 15 sessions) + pill placebo	39	1
IPT individual + pill placebo	48	IPT individual + pill placebo	48	1
SSRIs	15,203	Citalopram	1,676	2
		Escitalopram	3,818	
		Fluoxetine	3,981	
		Paroxetine	4,571	
		Sertraline	1,157	
TCAs	1,747	Amitriptyline	666	3
		Clomipramine	184	
		Imipramine	562	
		Lofepramine	68	
		Nortriptyline	267	
SNRIs	8,727	Duloxetine	5,472	2
		Venlafaxine	3,255	
Mirtazapine	726	Mirtazapine	726	
Trazodone	742	Trazodone	742	
Acupuncture	122	Electroacupuncture	28	1
		Laser acupuncture	41	
		Traditional acupuncture	53	
Exercise individual	336	Supervised high intensity exercise individual	177	4
		Supervised low intensity exercise individual	106	
		Unsupervised high intensity exercise individual	53	

Exercise group	104	Supervised high intensity exercise group	104	1
Yoga group	15	Yoga group	15	1
Light therapy	32	Bright light therapy	32	1
CT/CBT individual + AD	117	CBT individual (15 sessions or over) + imipramine	25	1
		CBT individual (under 15 sessions) + escitalopram	52	
		CBT individual (under 15 sessions) + sertraline	40	
CT/CBT group + AD	34	CBT group (under 15 sessions) + imipramine	34	1
Long-term PDPT + AD	91	Long-term PDPT individual + fluoxetine	91	1
IPT individual + AD	16	IPT individual + nortriptyline	16	1
Counselling individual + AD	13	Interpersonal counselling individual + venlafaxine	13	1
Exercise individual + AD	55	Supervised high intensity exercise individual + sertraline	55	1
Exercise group + AD	134	Supervised high intensity exercise group + sertraline	97	1
		Supervised low intensity exercise group + sertraline	37	
Acupuncture + AD	112	Electroacupuncture + paroxetine	58	1
		Traditional acupuncture + paroxetine	54	
Light therapy + AD	54	Bright light therapy + fluoxetine	29	1
		Bright light therapy + venlafaxine	25	

AD: antidepressant; CBT: cognitive behavioural therapy; CT: cognitive therapy; IPT: interpersonal psychotherapy; PDPT: psychodynamic psychotherapy; SNRIs: serotonin and norepinephrine reuptake inhibitors; SSRIs: selective serotonin uptake inhibitors; TAU: treatment as usual; TCAs: tricyclic antidepressants
** Classes with the same number share a common class variance*

Treatment discontinuation for any reason

Network - treatment class level

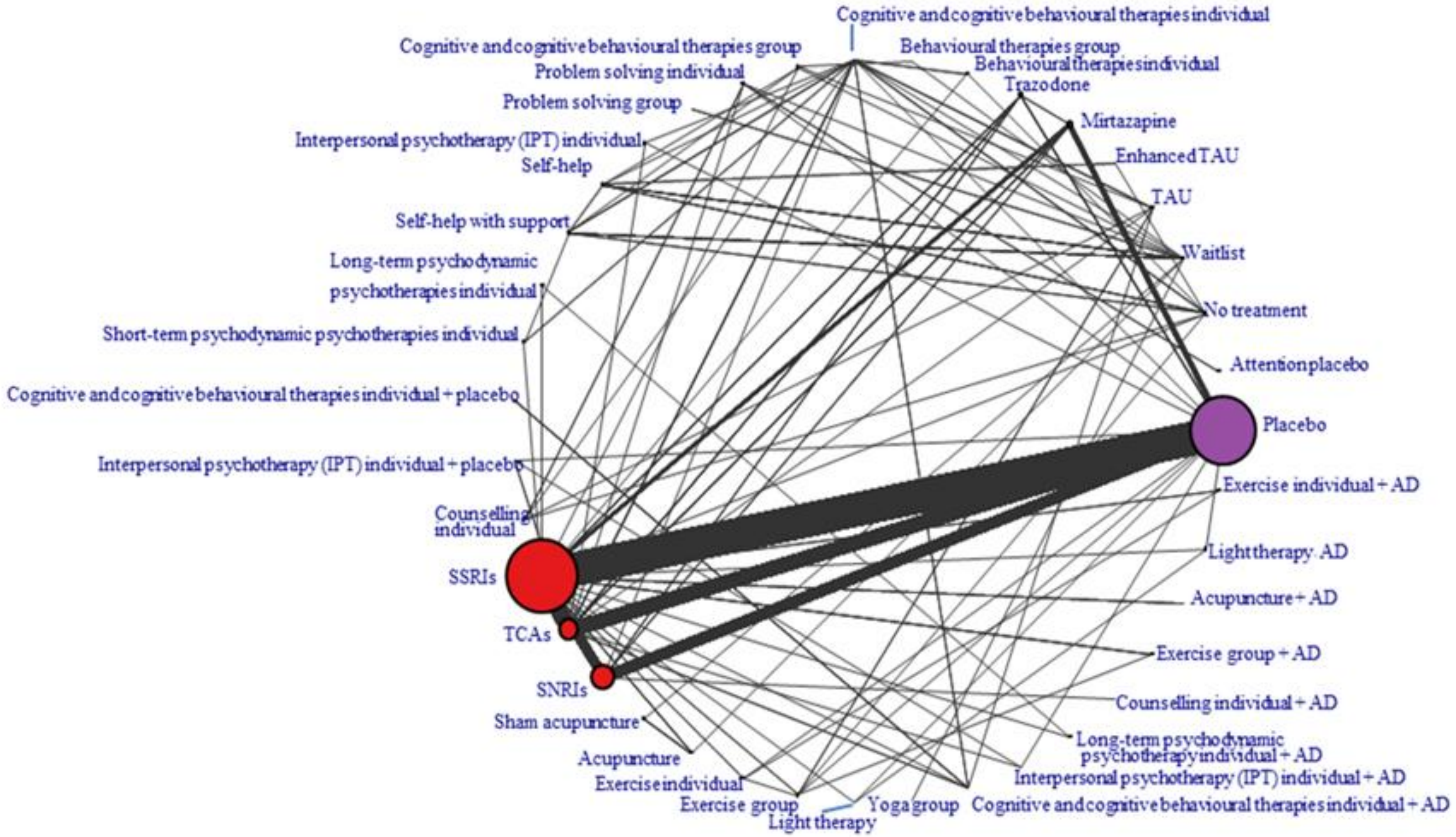

AD: antidepressant; SNRIs: serotonin and norepinephrine reuptake inhibitors; SSRIs: selective serotonin uptake inhibitors; TAU: treatment as usual; TCAs: tricyclic antidepressants

Network - intervention level

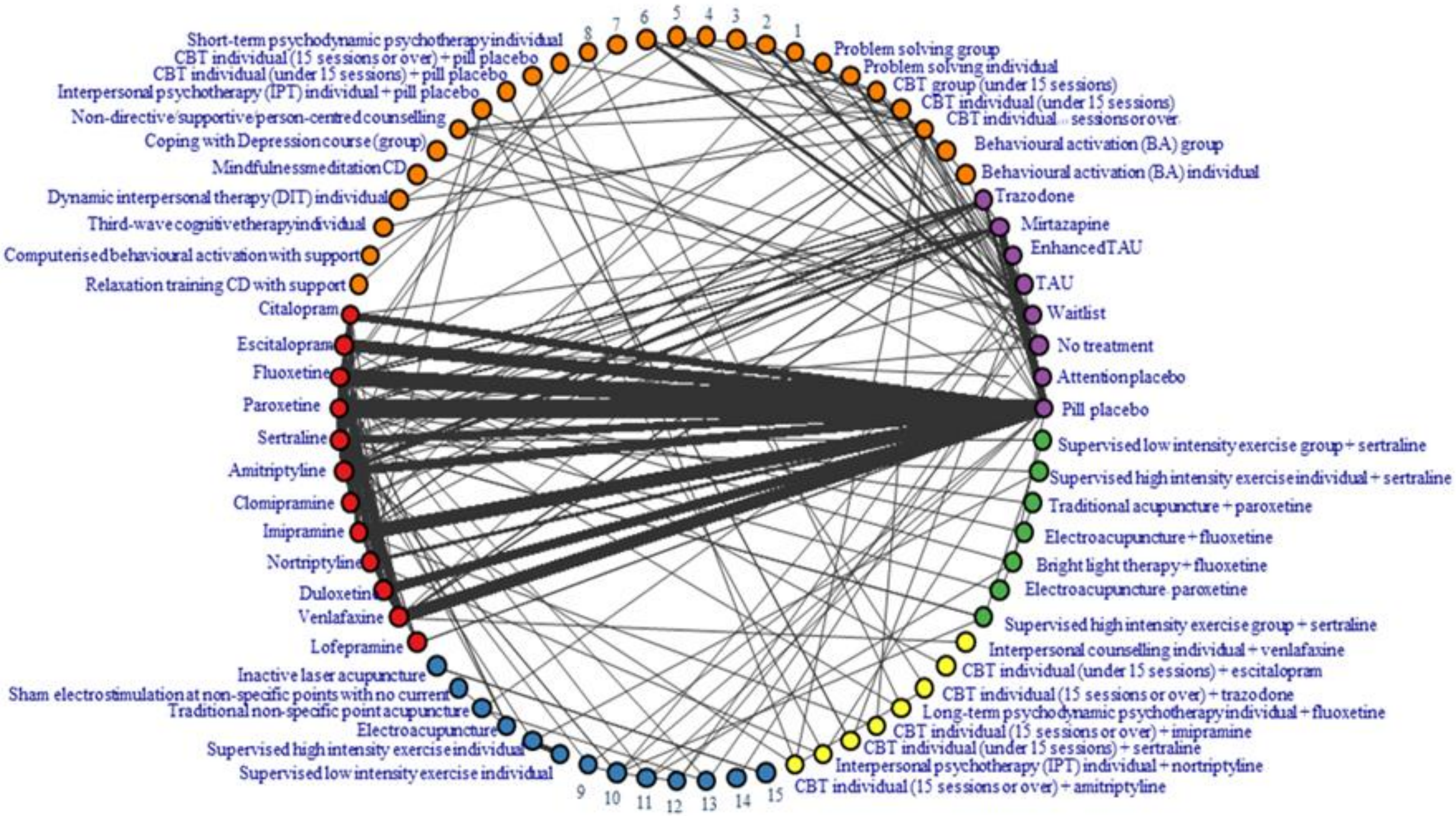

1 Interpersonal psychotherapy (IPT) individual; 2 Cognitive bibliotherapy; 3 Computerised CBT (CCBT); 4 Psychoeducational website; 5 Cognitive bibliotherapy with support; 6 Computerised CBT with support; 7 Mindfulness meditation CD with support; 8 Long-term psychodynamic therapy individual; 9 Unsupervised high intensity exercise individual; 10 Supervised high intensity exercise group; 11 Supervised low intensity exercise group; 12 Bright light therapy; 13 Traditional acupuncture; 14 Yoga group; 15 Laser acupuncture

Without the use of a class network the following treatments would be disconnected from the rest of the network and would have to be excluded from the analysis: Psychoeducational website, Mindfulness meditation CD with support, Inactive laser acupuncture, Computerised behavioural activation with support, Relaxation training CD with support, and Laser acupuncture

Classes, interventions and numbers of participants tested on each
The NMA included 402 RCTs, 74 interventions grouped in 39 treatment classes and 74,406 participants.

Class	N	Intervention	N	Variance Sharing*
Placebo	16577	Pill placebo	16577	
Attention placebo	36	Attention placebo	36	
No treatment	764	No treatment	764	
Waitlist	580	Waitlist	580	
TAU	266	TAU	266	
Enhanced TAU	37	Enhanced TAU	37	
Mirtazapine	2637	Mirtazapine	2637	
Trazodone	1430	Trazodone	1430	
Behavioural therapies individual	595	Behavioural activation (BA) individual	595	1
Behavioural therapies group	46	Behavioural activation (BA) group	15	1
		Coping with Depression course (group)	31	
CT/CBT individual	771	CBT individual (15 sessions or over)	461	1
		CBT individual (under 15 sessions)	287	
		Third-wave CT individual	23	
CT/CBT group	162	CBT group (under 15 sessions)	162	1
Problem solving individual	448	Problem solving individual	448	1
Problem solving group	58	Problem solving group	58	1
Counselling individual	332	Non-directive/supportive/person-centred counselling	332	1
IPT individual	63	IPT individual	63	1
Self-help	477	Cognitive bibliotherapy	169	2
		Computerised-CBT (CCBT)	115	
		Mindfulness meditation CD	39	
		Psychoeducational website	154	
Self-help with support	556	Cognitive bibliotherapy with support	67	3
		Computerised-CBT (CCBT) with support	290	
		Computerised behavioural activation with support	159	
		Mindfulness meditation CD with support	20	
		Relaxation training CD with support	20	
Long-term PDPT individual	90	Long-term PDPT individual	90	1
Short-term PDPT individual	129	Dynamic interpersonal therapy (DIT) individual	73	1
		Short-term PDPT individual	56	
CT/CBT individual + placebo	97	CBT individual (15 sessions or over) + pill placebo	14	1
		CBT individual (under 15 sessions) + pill placebo	83	
IPT individual + placebo	48	IPT individual + pill placebo	48	1
SSRIs	28464	Citalopram	3523	4
		Escitalopram	5627	
		Fluoxetine	7766	
		Paroxetine	8362	

		Sertraline	3186	
TCAs	7782	Amitriptyline	3778	5
		Clomipramine	601	
		Imipramine	2585	
		Lofepramine	296	
		Nortriptyline	522	
SNRIs	10251	Duloxetine	5226	4
		Venlafaxine	5025	
Sham acupuncture	117	Inactive laser acupuncture	36	1
		Sham electrostimulation at non-specific points with no current	29	
		Traditional non-specific point acupuncture	52	
Acupuncture	255	Electroacupuncture	112	1
		Laser acupuncture	41	
		Traditional acupuncture	102	
Exercise individual	336	Supervised high intensity exercise individual	162	3
		Supervised low intensity exercise individual	121	
		Unsupervised high intensity exercise individual	53	
Exercise group	167	Supervised high intensity exercise group	124	3
		Supervised low intensity exercise group	43	
Yoga group	30	Yoga group	30	1
Light therapy	32	Bright light therapy	32	1
CT/CBT individual + AD	246	CBT individual (15 sessions or over) + amitriptyline	50	6
		CBT individual (15 sessions or over) + imipramine	25	
		CBT individual (15 sessions or over) + trazodone	11	
		CBT individual (under 15 sessions) + escitalopram	52	
		CBT individual (under 15 sessions) + sertraline	108	
Long-term PDPT individual + AD	91	Long-term PDPT individual + fluoxetine	91	6
IPT individual + AD	16	IPT individual + nortriptyline	16	6
Counselling individual + AD	13	Interpersonal counselling individual + venlafaxine	13	6
Exercise individual + AD	84	Supervised high intensity exercise individual + sertraline	84	6
Exercise group + AD	134	Supervised high intensity exercise group + sertraline	97	6
		Supervised low intensity exercise group + sertraline	37	
Acupuncture + AD	160	Electroacupuncture + fluoxetine	48	1
		Electroacupuncture + paroxetine	58	
		Traditional acupuncture + paroxetine	54	
Light therapy + AD	29	Bright light therapy + fluoxetine	29	1

AD: antidepressant; CBT: cognitive behavioural therapy; CT: cognitive therapy; IPT: interpersonal psychotherapy; MBCT: mindfulness-based cognitive therapy; PDPT: psychodynamic psychotherapy; SNRIs: serotonin and norepinephrine reuptake inhibitors; SSRIs: selective serotonin uptake inhibitors; TAU: treatment as usual; TCAs: tricyclic antidepressants
** Classes with the same number share a common class variance*

Treatment discontinuation due to side effects from medication, in those who discontinued treatment

Network - treatment class level

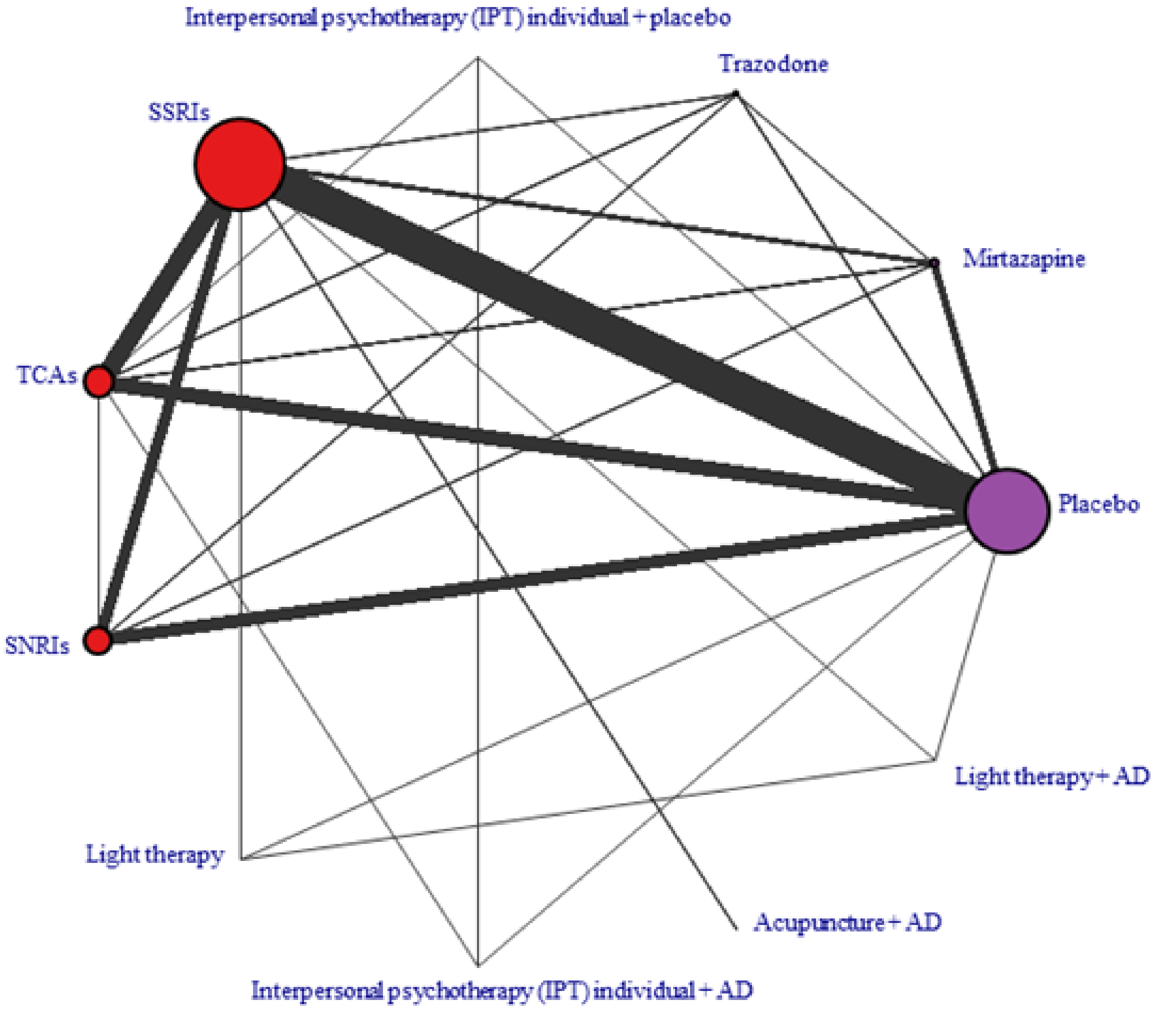

AD: antidepressant; SNRIs: serotonin and norepinephrine reuptake inhibitors; SSRIs: selective serotonin uptake inhibitors; TCAs: tricyclic antidepressants

Network - intervention level

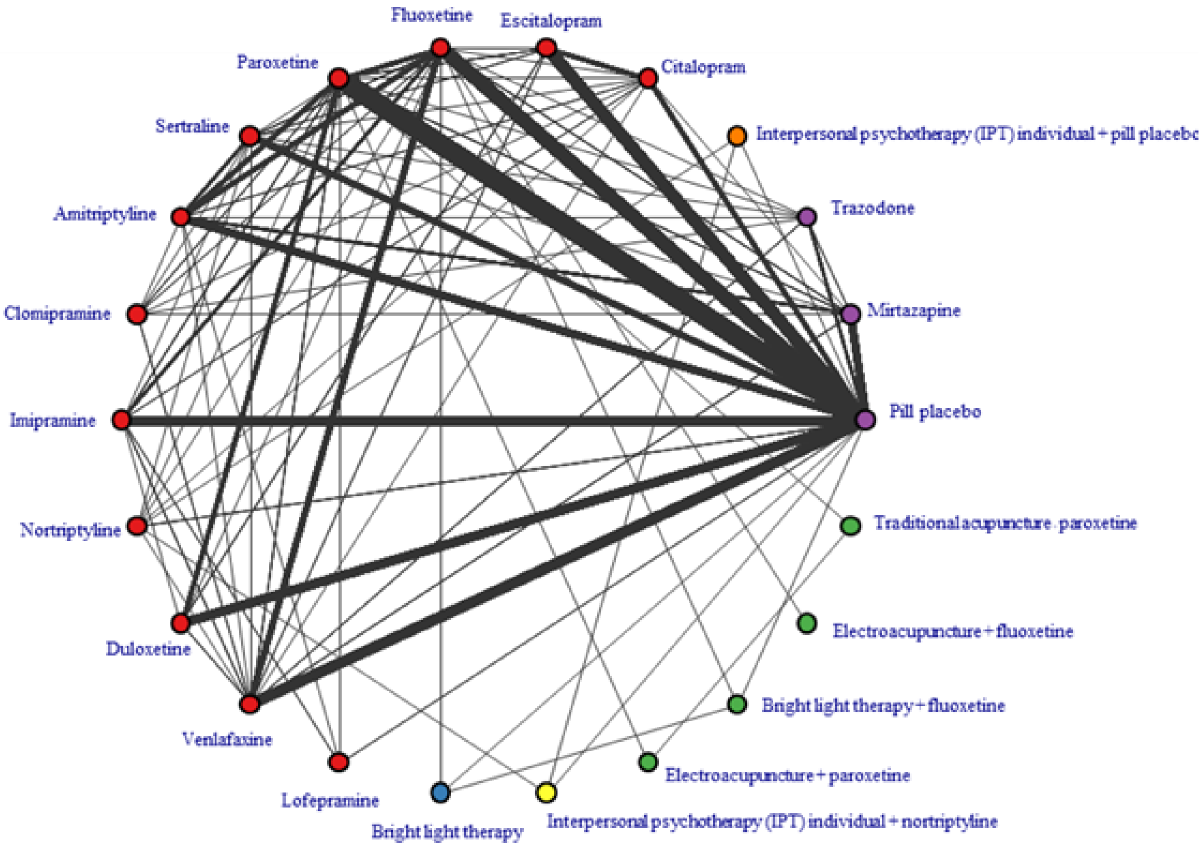

Classes, interventions and numbers of participants tested on each
The NMA included 278 RCTs, 22 interventions grouped in 11 treatment classes and 16,354 participants.

Class	N	Intervention	N	Variance Sharing*
Placebo	4231	Pill placebo	4231	
Mirtazapine	692	Mirtazapine	692	
Trazodone	365	Trazodone	365	
IPT individual + placebo	17	IPT individual + pill placebo	17	1
SSRIs	6445	Citalopram	661	1
		Escitalopram	1108	
		Fluoxetine	1831	
		Paroxetine	2082	
		Sertraline	763	
TCAs	2096	Amitriptyline	963	2
		Clomipramine	174	
		Imipramine	759	
		Lofepramine	80	
		Nortriptyline	120	
SNRIs	2478	Duloxetine	1272	1
		Venlafaxine	1206	
Light therapy	4	Bright light therapy	4	Max(1,2)
IPT individual + AD	10	IPT individual + nortriptyline	10	Max(1,2)
Acupuncture + AD	14	Electroacupuncture + fluoxetine	2	Max(1,2)
		Electroacupuncture + paroxetine	9	
		Traditional acupuncture + paroxetine	3	
Light therapy + AD	2	Bright light therapy + fluoxetine	2	Max(1,2)

AD: antidepressant; IPT: interpersonal psychotherapy; SNRIs: serotonin and norepinephrine reuptake inhibitors; SSRIs: selective serotonin uptake inhibitors; TCAs: tricyclic antidepressants
** Classes with the same number share a common class variance*

Response in treatment completers

Network - treatment class level

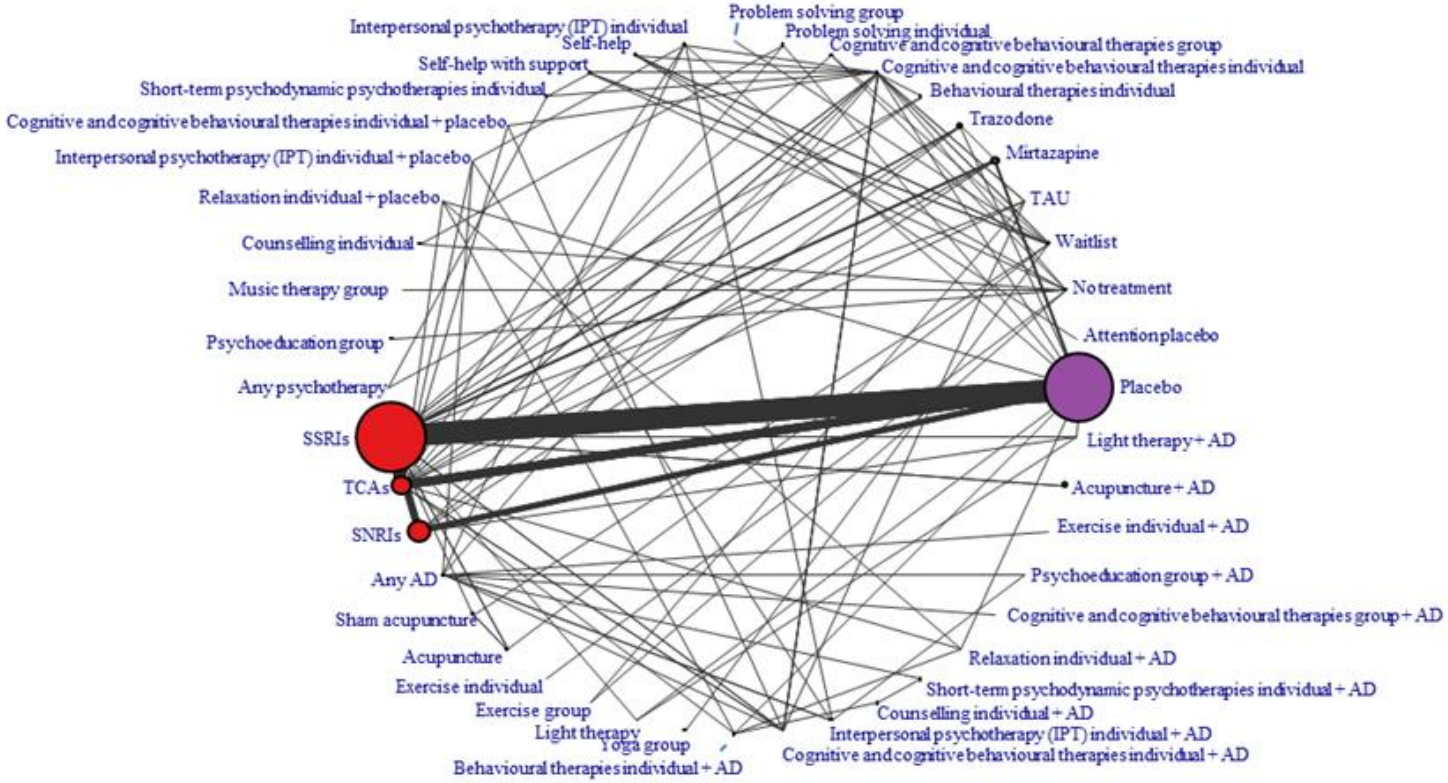

AD: antidepressant; SNRIs: serotonin and norepinephrine reuptake inhibitors; SSRIs: selective serotonin uptake inhibitors; TAU: treatment as usual; TCAs: tricyclic antidepressants

Network - intervention level

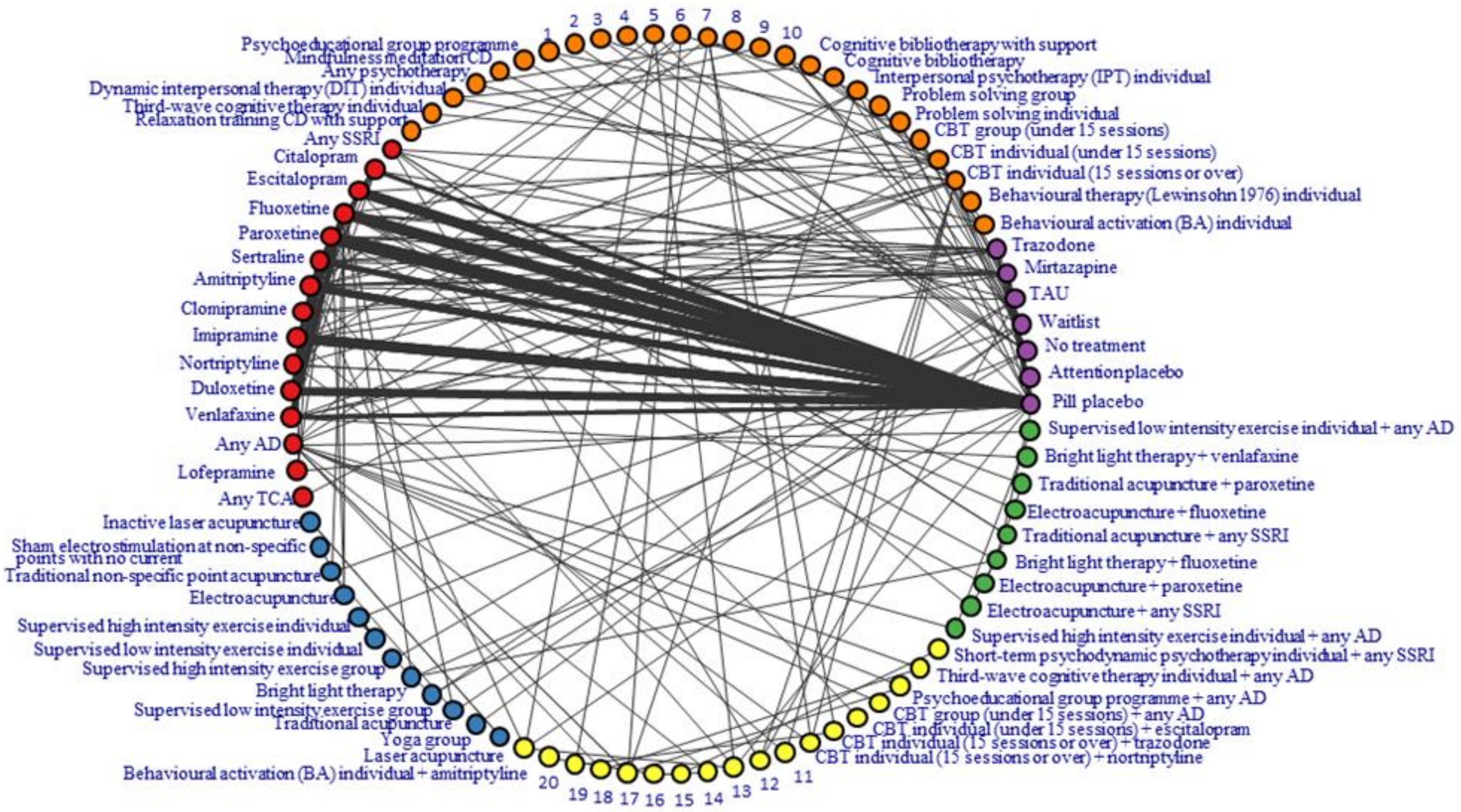

1 Non-directive/supportive/ person-centred counselling; 2 Music therapy group; 3 Computerised CBT (CCBT); 4 Computerised attentional bias modification; 5 Progressive muscle relaxation individual +pill placebo; 6 Interpersonal psychotherapy (IPT) individual +pill placebo; 7 CBT individual (15 sessions or over) + pill placebo; 8 Short-term psychodynamic psychotherapy individual; 9 Mindfulness meditation CD with support; 10 Computerised-CBT (CCBT) with support; 11 CBT individual (15 sessions or over) + imipramine; 12 CBT individual (15 sessions or over) + any SSRI; 13 Progressive muscle relaxation individual + amitriptyline; 14 Short-term psychodynamic psychotherapy individual + any AD; 15 Non-directive/supportive/ person-centred counselling + any SSRI; 16 Non-directive/supportive/ person-centred counselling + any AD; 17 Interpersonal psychotherapy (IPT) individual + any AD; 18 CBT individual (15 sessions or over) + any AD; 19 CBT individual (15 sessions or over) + amitriptyline; 20 Behavioural activation (BA) individual + any AD

Without the use of a class network the following interventions would be disconnected from the rest of the network and would have to be excluded from the analysis: Attention placebo, Mindfulness meditation CD with support, Inactive laser acupuncture, Non-directive/supportive/person-centred counselling + any SSRI, Computerised attentional bias modification, Relaxation training CD with support, Laser acupuncture, and Short-term psychodynamic psychotherapy individual + any SSRI

Classes, interventions and numbers of participants tested on each
The NMA included 327 RCTs, 87 interventions grouped in 44 treatment classes and 44,614 participants. Of the 327 RCTs, 250 reported dichotomous response data, 21 reported CFB depression symptom score data; and 56 reported baseline and endpoint depression symptom score data.

Class	N	Intervention	N	Variance Sharing*
Placebo	9333	Pill placebo	9333	
Attention placebo	25	Attention placebo	25	
No treatment	266	No treatment	266	
Waitlist	371	Waitlist	371	
TAU	64	TAU	64	
Mirtazapine	1845	Mirtazapine	1845	
Trazodone	1003	Trazodone	1003	
Behavioural therapies individual	320	Behavioural activation (BA) individual	310	1
		Behavioural therapy (Lewinsohn 1976) individual	10	
CT/CBT individual	507	CBT individual (15 sessions or over)	348	1
		CBT individual (under 15 sessions)	141	
		Third-wave CT individual	18	
CT/CBT group	64	CBT group (under 15 sessions)	64	1
Problem solving individual	123	Problem solving individual	123	1
Problem solving group	47	Problem solving group	47	1
Counselling individual	216	Non-directive/supportive/person-centred counselling	216	1
IPT individual	132	IPT individual	132	1
Psychoeducation group	44	Psychoeducational group programme	44	1
Self-help	231	Cognitive bibliotherapy	147	2
		Computerised-CBT (CCBT)	23	
		Computerised attentional bias modification	26	
		Mindfulness meditation CD	35	
Self-help with support	189	Cognitive bibliotherapy with support	38	3
		Computerised-CBT (CCBT) with support	114	
		Mindfulness meditation CD with support	19	
		Relaxation training CD with support	18	
Short-term PDPT individual	75	Dynamic interpersonal therapy (DIT) individual	59	1
		Short-term PDPT individual	16	
Music therapy group	12	Music therapy group	12	1
Any psychotherapy	27	Any psychotherapy	27	1
CT/CBT individual + placebo	26	CBT individual (15 sessions or over) + pill placebo	26	1
IPT individual + placebo	69	IPT individual + pill placebo	69	1
Relaxation individual + placebo	11	Progressive muscle relaxation individual + pill placebo	11	1
SSRIs	16720	Any SSRI	201	4

		Citalopram	1762	
		Escitalopram	3396	
		Fluoxetine	4804	
		Paroxetine	4291	
		Sertraline	2266	
TCAs	4233	Amitriptyline	2222	4
		Any TCA	21	
		Clomipramine	297	
		Imipramine	1247	
		Lofepramine	188	
		Nortriptyline	258	
SNRIs	6569	Duloxetine	3700	4
		Venlafaxine	2869	
Any AD	286	Any AD	286	4
Sham acupuncture	188	Inactive laser acupuncture	33	1
		Sham electrostimulation at non-specific points with no current	22	
		Traditional non-specific point acupuncture	133	
Acupuncture	249	Electroacupuncture	83	1
		Laser acupuncture	36	
		Traditional acupuncture	130	
Exercise individual	88	Supervised high intensity exercise individual	47	3
		Supervised low intensity exercise individual	41	
Exercise group	55	Supervised high intensity exercise group	18	3
		Supervised low intensity exercise group	37	
Yoga group	20	Yoga group	20	1
Light therapy	28	Bright light therapy	28	1
Behavioural therapies individual + AD	22	Behavioural activation (BA) individual + amitriptyline	12	5
		Behavioural activation (BA) individual + any AD	10	
CT/CBT individual + AD	157	CBT individual (15 sessions or over) + amitriptyline	10	5
		CBT individual (15 sessions or over) + any AD	10	
		CBT individual (15 sessions or over) + any SSRI	43	
		CBT individual (15 sessions or over) + imipramine	16	
		CBT individual (15 sessions or over) + nortriptyline	18	
		CBT individual (15 sessions or over) + trazodone	10	
		CBT individual (under 15 sessions) + escitalopram	40	
		Third-wave CT individual + any AD	10	
CT/CBT group + AD	43	CBT group (under 15 sessions) + any AD	43	5
IPT individual + AD	87	IPT individual + any AD	87	5
Counselling individual + AD	71	Non-directive/supportive/person-centred counselling + any AD	55	5

		Non-directive/supportive/person-centred counselling + any SSRI	16	
Short-term PDPT individual + AD	168	Short-term PDPT individual + any AD	152	5
		Short-term PDPT individual + any SSRI	16	
Psychoeducation group + AD	27	Psychoeducational group programme + any AD	27	5
Relaxation individual + AD	10	Progressive muscle relaxation individual + amitriptyline	10	5
Exercise individual + AD	22	Supervised high intensity exercise individual + any AD	13	5
		Supervised low intensity exercise individual + any AD	9	
Acupuncture + AD	519	Electroacupuncture + any SSRI	138	1
		Electroacupuncture + fluoxetine	46	
		Electroacupuncture + paroxetine	49	
		Traditional acupuncture + any SSRI	185	
		Traditional acupuncture + paroxetine	101	
Light therapy + AD	52	Bright light therapy + fluoxetine	27	1
		Bright light therapy + venlafaxine	25	

AD: antidepressant; CBT: cognitive behavioural therapy; CT: cognitive therapy; IPT: interpersonal psychotherapy; PDPT: psychodynamic psychotherapy; SNRIs: serotonin and norepinephrine reuptake inhibitors; SSRIs: selective serotonin uptake inhibitors; TAU: treatment as usual; TCAs: tricyclic antidepressants
** Classes with the same number share a common class variance*

Remission in treatment completers

Network - treatment class level

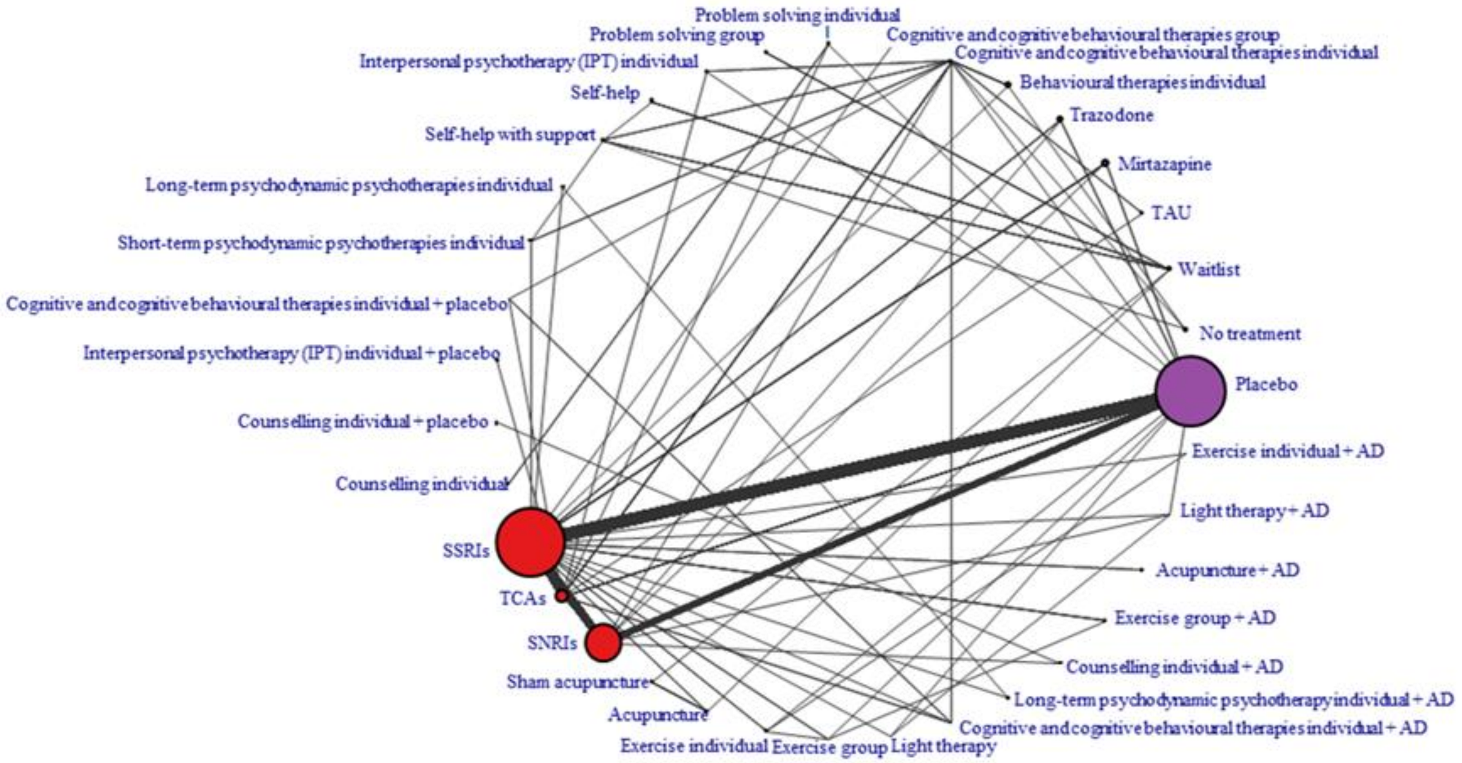

AD: antidepressant; SNRIs: serotonin and norepinephrine reuptake inhibitors; SSRIs: selective serotonin uptake inhibitors; TAU: treatment as usual; TCAs: tricyclic antidepressants

Network - intervention level

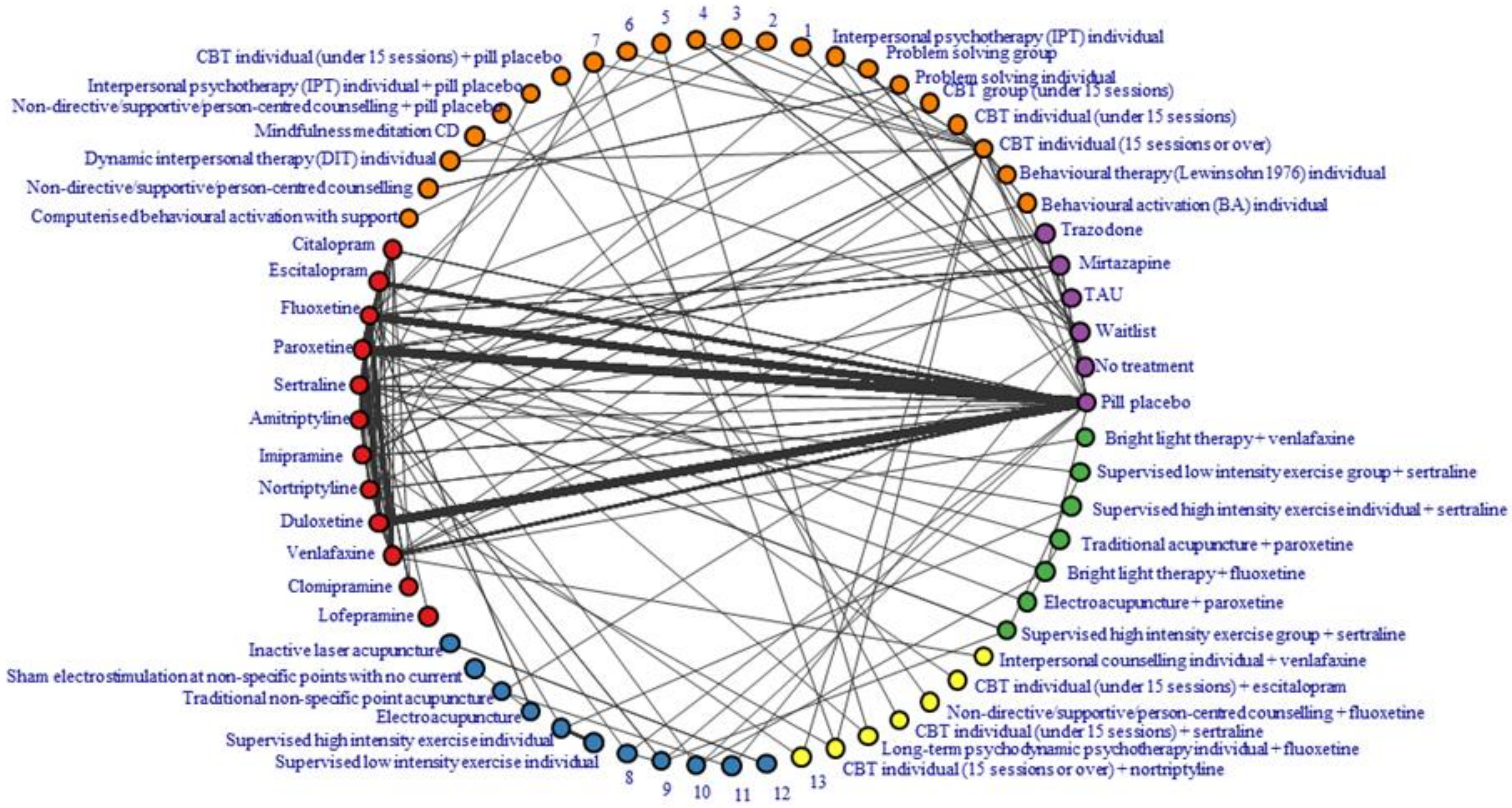

1 Cognitive bibliotherapy; 2 Psychoeducational website; 3 Cognitive bibliotherapy with support; 4 Computerised CBT (CCBT) with support; 5 Long-term psychodynamic psychotherapy individual; 6 Short-term psychodynamic psychotherapy individual; 7 CBT individual (15 sessions or over) + pill placebo; 8 Unsupervised high intensity exercise individual; 9 Supervised high intensity exercise group; 10 Bright light therapy; 11 Traditional acupuncture; 12 Laser acupuncture; 13 CBT individual (15 sessions or over) + imipramine
Without the use of a class network the following treatments would be disconnected from the rest of the network and would have to be excluded from the analysis: Psychoeducational website, CBT individual (under 15 sessions) + pill placebo, Non-directive/supportive/person-centred counselling + pill placebo, Inactive laser acupuncture, Computerised behavioural activation with support, CBT individual (under 15 sessions) + sertraline, Non-directive/supportive/person-centred counselling + fluoxetine, and Laser acupuncture

Classes, interventions and numbers of participants tested on each
The NMA included 185 RCTs, 65 interventions grouped in 35 treatment classes, and 28,432 participants.

Class	N	Intervention	N	Variance Sharing*
Placebo	5850	Pill placebo	5850	
No treatment	299	No treatment	299	
Waitlist	309	Waitlist	309	
TAU	45	TAU	45	
Mirtazapine	645	Mirtazapine	645	
Trazodone	552	Trazodone	552	
Behavioural therapies individual	330	Behavioural activation (BA) individual	320	1
		Behavioural therapy (Lewinsohn 1976) individual	10	
CT/CBT individual	440	CBT individual (15 sessions or over)	391	1
		CBT individual (under 15 sessions)	49	
CT/CBT group	32	CBT group (under 15 sessions)	32	1
Problem solving individual	191	Problem solving individual	191	1
Problem solving group	47	Problem solving group	47	1
Counselling individual	103	Non-directive/supportive/person-centred counselling	103	1
IPT individual	89	IPT individual	89	1
Self-help	327	Cognitive bibliotherapy	147	1
		Mindfulness meditation CD	35	
		Psychoeducational website	145	
Self-help with support	323	Cognitive bibliotherapy with support	38	1
		Computerised-CBT (CCBT) with support	165	
		Computerised behavioural activation with support	120	
Long-term PDPT individual	73	Long-term PDPT individual	73	1
Short-term PDPT individual	101	Dynamic interpersonal therapy (DIT) individual	59	1
		Short-term PDPT individual	42	
CT/CBT individual + placebo	38	CBT individual (15 sessions or over) + pill placebo	17	1
		CBT individual (under 15 sessions) + pill placebo	21	
IPT individual + placebo	22	IPT individual + pill placebo	22	1
Counselling individual + placebo	11	Non-directive/supportive/person-centred counselling + pill placebo	11	1
SSRIs	10361	Citalopram	1041	2
		Escitalopram	2457	
		Fluoxetine	3001	
		Paroxetine	3110	
		Sertraline	752	
TCAs	1204	Amitriptyline	486	3
		Clomipramine	135	
		Imipramine	318	
		Lofepramine	55	

		Nortriptyline	210	
SNRIs	5949	Duloxetine	3674	2
		Venlafaxine	2275	
Sham acupuncture	100	Inactive laser acupuncture	33	4
		Sham electrostimulation at non-specific points with no current	22	
		Traditional non-specific point acupuncture	45	
Acupuncture	145	Electroacupuncture	67	4
		Laser acupuncture	36	
		Traditional acupuncture	42	
Exercise individual	242	Supervised high intensity exercise individual	109	5
		Supervised low intensity exercise individual	83	
		Unsupervised high intensity exercise individual	50	
Exercise group	80	Supervised high intensity exercise group	80	1
Light therapy	28	Bright light therapy	28	4
CT/CBT individual + AD	100	CBT individual (15 sessions or over) + imipramine	16	6
		CBT individual (15 sessions or over) + nortriptyline	18	
		CBT individual (under 15 sessions) + escitalopram	40	
		CBT individual (under 15 sessions) + sertraline	26	
Long-term PDPT individual + AD	62	Long-term PDPT individual + fluoxetine	62	6
Counselling individual + AD	24	Interpersonal counselling individual + venlafaxine	11	6
		Non-directive/supportive/person-centred counselling + fluoxetine	13	
Exercise individual + AD	44	Supervised high intensity exercise individual + sertraline	44	6
Exercise group + AD	114	Supervised high intensity exercise group + sertraline	82	6
		Supervised low intensity exercise group + sertraline	32	
Acupuncture + AD	100	Electroacupuncture + paroxetine	49	4
		Traditional acupuncture + paroxetine	51	
Light therapy + AD	52	Bright light therapy + fluoxetine	27	4
		Bright light therapy + venlafaxine	25	

AD: antidepressant; CBT: cognitive behavioural therapy; CT: cognitive therapy; IPT: interpersonal psychotherapy; PDPT: psychodynamic psychotherapy; SNRIs: serotonin and norepinephrine reuptake inhibitors; SSRIs: selective serotonin uptake inhibitors; TAU: treatment as usual; TCAs: tricyclic antidepressants
** Classes with the same number share a common class variance*
