# Supplementary material for: A systematic review and network meta-analysis of psychological, psychosocial, pharmacological, physical and combined treatments for adults with a new episode of depression
Source: eClinicalMedicine. 2024 Aug 16;75:102780. doi: 10.1016/j.eclinm.2024.102780 (PMC11377144; doi:10.1016/j.eclinm.2024.102780)
Supplement: Appendix 6 Results [file mmc6.pdf]

APPENDIX 6

CONTENTS

Network meta-analysis model fit statistics ... 3
Less severe depression ... 3
SMD of depression scale change scores ... 3
Response in those randomised ... 3
Remission in those randomised ... 3
Treatment discontinuation for any reason ... 3
Treatment discontinuation due to side effects from medication, in those who discontinued treatment ... 3
Response in treatment completers ... 4
Remission in treatment completers ... 4
More severe depression ... 5
SMD of depression scale change scores ... 5
Response in those randomised ... 5
Remission in those randomised ... 5
Treatment discontinuation for any reason ... 5
Treatment discontinuation due to side effects from medication, in those who discontinued treatment ... 5
Response in treatment completers ... 6
Remission in treatment completers ... 6
Network meta-analysis inconsistency checks and bias adjustment models - results ... 7
Less severe depression ... 7
SMD of depression scale change scores ... 7
Response in those randomised ... 9
Remission in those randomised ... 10
Treatment discontinuation for any reason ... 11
Treatment discontinuation due to side effects from medication, in those who discontinued treatment ... 13
Response in treatment completers ... 13
Remission in treatment completers ... 15
More severe depression ... 16
SMD of depression scale change scores ... 16
Response in those randomised ... 18
Remission in those randomised ... 19
Treatment discontinuation for any reason ... 20
Treatment discontinuation due to side effects from medication, in those who discontinued treatment ... 22
Response in treatment completers ... 23
Remission in treatment completers ... 25
Risk of bias of studies included in the NMA ... 26
Less severe depression ... 26
More severe depression ... 26
Quality assessment of the evidence - results ... 27

Less severe depression 27
Risk of bias 27
Model goodness of fit and inconsistency 27
Selective outcome reporting and publication bias 27
Indirectness 27
Imprecision 28
Overall rating of the quality of the evidence 28
More severe depression 29
Risk of bias 29
Model goodness of fit and inconsistency 29
Selective outcome reporting and publication bias 29
Indirectness 29
Imprecision 30
Overall rating of the quality of the evidence 30
Network meta-analysis results for all outcomes at the treatment class level. Relative effects versus the reference treatment. 31
Less severe depression 31
Standardised mean difference (SMD) of depression scale change scores 31
Response in those randomised 36
Remission in those randomised 38
Discontinuation for any reason 39
Discontinuation due to side effects 40
Response in treatment completers 41
Remission in treatment completers 42
More severe depression 43
Standardised mean difference (SMD) of depression scale change scores 43
Response in those randomised 49
Remission in those randomised 51
Discontinuation for any reason 53
Discontinuation due to side effects 55
Response in treatment completers 56
Remission in treatment completers 58

Network meta-analysis model fit statistics

Less severe depression

SMD of depression scale change scores

Model	Between Study Heterogeneity - SD (95% CrI)	Posterior total residual deviance[a]	DIC[b]
RE – random class effect	0·49 (0·40, 0·60)	343·0	1247
RE – inconsistency	0·49 (0·38, 0·62)	345·1	1259
RE – random class effect: bias adjustment	0·44 (0·27, 0·57)	283·9	1192

Abbreviations: CrI, credible interval; DIC, deviance information criteria; RE, random study-specific treatment effects; SD, standard deviation
a Posterior mean residual deviance compared to 279 total data points
b Lower values of DIC preferred

Response in those randomised

Model	Between Study Heterogeneity - SD (95% CrI)	Posterior total residual deviance[a]	DIC[b]
RE – random class effect	0·76 (0·55, 1·02)	172·4	729·4
RE – inconsistency	0·93 (0·67, 1·26)	173·9	741·5

Abbreviations: CrI, credible interval; DIC, deviance information criteria; RE, random study-specific treatment effects; SD, standard deviation
a Posterior mean residual deviance compared to 166 total data points
b Lower values of DIC preferred

Remission in those randomised

Model	Between Study Heterogeneity - SD (95% CrI)	Posterior total residual deviance[a]	DIC[b]
RE – random class effect	0·45 (0·04, 1·04)	59·31	334·5
RE – inconsistency	0·46 (0·04, 1·14)	59·18	335·1

Abbreviations: CrI, credible interval; DIC, deviance information criteria; RE, random study-specific treatment effects; SD, standard deviation
a Posterior mean residual deviance compared to 56 total data points
b Lower values of DIC preferred

Treatment discontinuation for any reason

Model	Between Study Heterogeneity - SD (95% CrI)	Posterior total residual deviance[a]	DIC[b]
RE – random class effect	0·53 (0·38, 0·70)	265·2	1380
RE – inconsistency	0·54 (0·38, 0·74)	269·1	1400
RE – random class effect: bias adjustment	0·50 (0·35, 0·68)	259·4	1380

Abbreviations: CrI, credible interval; DIC, deviance information criteria; RE, random study-specific treatment effects; SD, standard deviation
a Posterior mean residual deviance compared to 263 total data points
b Lower values of DIC preferred

Treatment discontinuation due to side effects from medication, in those who discontinued treatment

NMA was not conducted for this outcome in this population.

Response in treatment completers

Model	Between Study Heterogeneity - SD (95% CrI)	Posterior total residual deviance[a]	DIC[b]
RE – random class effect	0·96 (0·71, 1·28)	168·2	166
RE – inconsistency	0·62 (0·30, 0·98)	168·5	166
RE – random class effect: bias adjustment	0·56 (0·03, 1·13)	167·5	166

Abbreviations: CrI, credible interval; DIC, deviance information criteria; RE, random study-specific treatment effects; SD, standard deviation
a Posterior mean residual deviance compared to 166 total data points
b Lower values of DIC preferred

Remission in treatment completers

Model	Between Study Heterogeneity - SD (95% CrI)	Posterior total residual deviance[a]	DIC[b]
RE – random class effect	0·35 (0·02, 0·89)	61·2	338·5
RE – inconsistency	0·37 (0·02, 1·05)	61·2	340·8

Abbreviations: CrI, credible interval; DIC, deviance information criteria; RE, random study-specific treatment effects; SD, standard deviation
a Posterior mean residual deviance compared to 59 total data points
b Lower values of DIC preferred

More severe depression

SMD of depression scale change scores

Model	Between Study Heterogeneity - SD (95% CrI)	Posterior total residual deviance[a]	DIC[b]
RE – random class effect	0·25 (0·22, 0·29)	896·3	3299
RE – inconsistency	0·22 (0·18, 0·25)	899·7	3317
RE – random class effect: bias adjustment	0·19 (0·15, 0·23)	830·9	3255

Abbreviations: CrI, credible interval; DIC, deviance information criteria; RE, random study-specific treatment effects; SD, standard deviation
a Posterior mean residual deviance compared to 811 total data points
b Lower values of DIC preferred

Response in those randomised

Model	Between Study Heterogeneity - SD (95% CrI)	Posterior total residual deviance[a]	DIC[b]
RE – random class effect	0·26 (0·21, 0·31)	927·4	4816
RE – inconsistency	0·22 (0·17, 0·27)	926·5	4827

Abbreviations: CrI, credible interval; DIC, deviance information criteria; RE, random study-specific treatment effects; SD, standard deviation
a Posterior mean residual deviance compared to 835 total data points
b Lower values of DIC preferred

Remission in those randomised

Model	Between Study Heterogeneity - SD (95% CrI)	Posterior total residual deviance[a]	DIC[b]
RE – random class effect	0·27 (0·20, 0·34)	471·2	2833
RE – inconsistency	0·24 (0·16, 0·32)	472·0	2846

Abbreviations: CrI, credible interval; DIC, deviance information criteria; RE, random study-specific treatment effects; SD, standard deviation
a Posterior mean residual deviance compared to 456 total data points
b Lower values of DIC preferred

Treatment discontinuation for any reason

Model	Between Study Heterogeneity - SD (95% CrI)	Posterior total residual deviance[a]	DIC[b]
RE – random class effect	0·31 (0·26, 0·36)	950·4	5403
RE – inconsistency	0·25 (0·20, 0·30)	958·3	5415
RE – random class effect: bias adjustment	0·28 (0·22, 0·33)	915·7	5388

Abbreviations: CrI, credible interval; DIC, deviance information criteria; RE, random study-specific treatment effects; SD, standard deviation
a Posterior mean residual deviance compared to 923 total data points
b Lower values of DIC preferred

Treatment discontinuation due to side effects from medication, in those who discontinued treatment

Model	Between Study Heterogeneity - SD (95% CrI)	Posterior total residual deviance[a]	DIC[b]
RE – random class effect	0·44 (0·33, 0·55)	659·0	2975
RE – inconsistency	0·37 (0·26, 0·48)	671·3	2987

Abbreviations: CrI, credible interval; DIC, deviance information criteria; RE, random study-specific treatment effects; SD, standard deviation

a Posterior mean residual deviance compared to 633 total data points
b Lower values of DIC preferred

Response in treatment completers

Model	Between Study Heterogeneity - SD (95% CrI)	Posterior total residual deviance[a]	DIC[b]
RE – random class effect	0·65 (0·58, 0·74)	763·8	4146
RE – inconsistency	0·67 (0·59, 0·76)	775·9	4180
RE – random class effect: bias adjustment	0·60 (0·52, 0·68)	750·0	4138

Abbreviations: CrI, credible interval; DIC, deviance information criteria; RE, random study-specific treatment effects; SD, standard deviation
a Posterior mean residual deviance compared to 752 total data points
b Lower values of DIC preferred

Remission in treatment completers

Model	Between Study Heterogeneity - SD (95% CrI)	Posterior total residual deviance[a]	DIC[b]
RE – random class effect	0·14 (0·02, 0·24)	445·6	2475
RE – inconsistency	0·10 (0·00, 0·21)	451·7	2496

Abbreviations: CrI, credible interval; DIC, deviance information criteria; RE, random study-specific treatment effects; SD, standard deviation
a Posterior mean residual deviance compared to 422 total data points
b Lower values of DIC preferred

Network meta-analysis inconsistency checks and bias adjustment models - results

Less severe depression

SMD of depression scale change scores

No evidence of inconsistency was identified with the NMA model having a slightly lower DIC, and similar between study heterogeneity (see Model fit statistics in this Appendix). The inconsistency model did not predict the data substantially better for any data points (Figure 1). Between study heterogeneity was lower in the bias-adjusted model that accounted for small study effects (performed as a pre-specified sensitivity analysis) (see Model fit statistics). The negative bias parameter (-2·96; 95%CrI: -5·11 to -0·91) indicated that smaller studies had larger effects favouring active interventions versus control interventions or counselling. Reported results are therefore based on the bias-adjusted random-effects NMA model, assuming consistency (Figure 2). Moderate between trials heterogeneity was found relative to the size of the treatment effect estimates following bias adjustment [τ=0·44 (95% CrI 0·27 to 0·57)].

Figure 1: Deviance contributions for the random effects consistency and inconsistency models. SMD of depression scale change scores – less severe depression

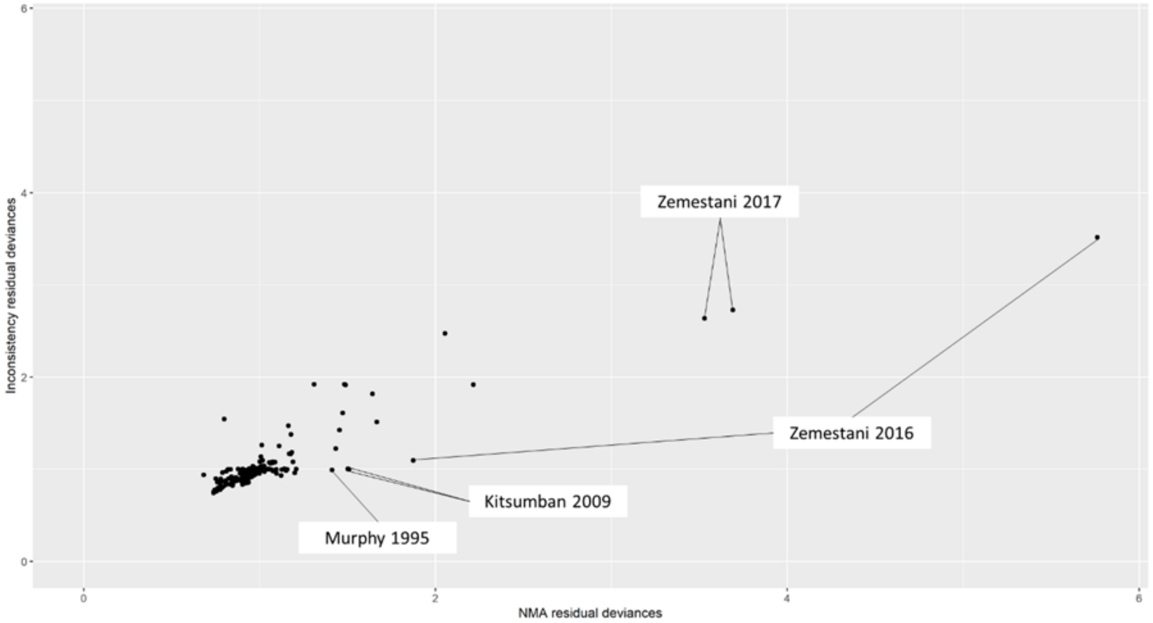

Figure 2: Forest plot for bias-adjusted versus base-case NMA of the SMD of depression scale change scores, for each class versus TAU – less severe depression. Bias-adjusted model was selected.

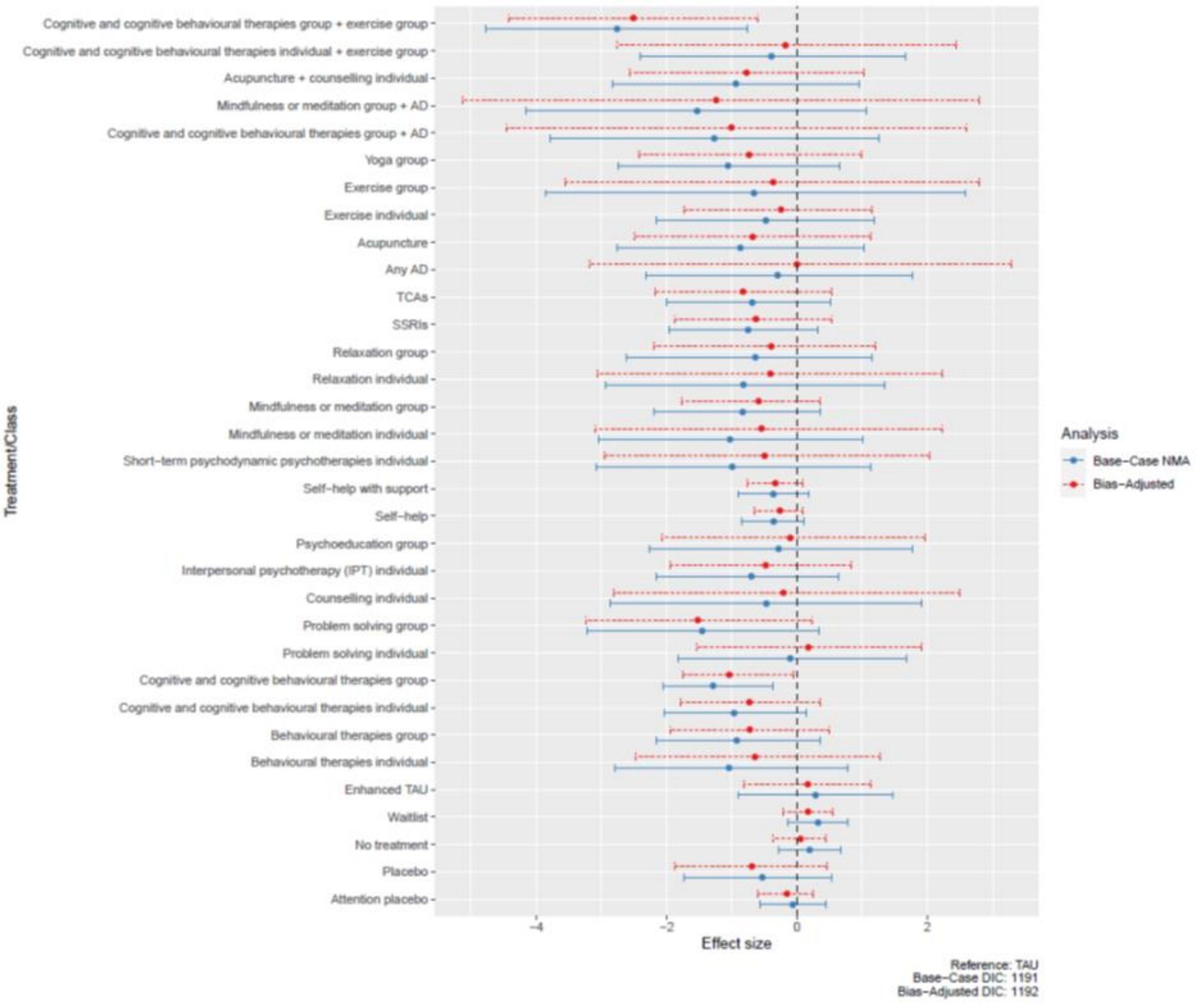

Response in those randomised

No evidence of inconsistency was identified with the NMA model having a similar posterior mean residual deviance and lower DIC and between study heterogeneity (see Model fit statistics). The inconsistency model did not predict the data substantially better for any data points, although both consistency and inconsistency models provided a poor fit for Zemestani 2016, which compared Waitlist, Behavioural activation (BA) group and Third-wave cognitive therapy group (Figure 3). Reported results are therefore based on the random-effects NMA model, assuming consistency. High between trials heterogeneity was found relative to the size of the treatment effect estimates [τ=0·76 (95% CrI 0·55 to 1·02)].

Figure 3: Deviance contributions for the random effects consistency and inconsistency models. Response in those randomised – less severe depression

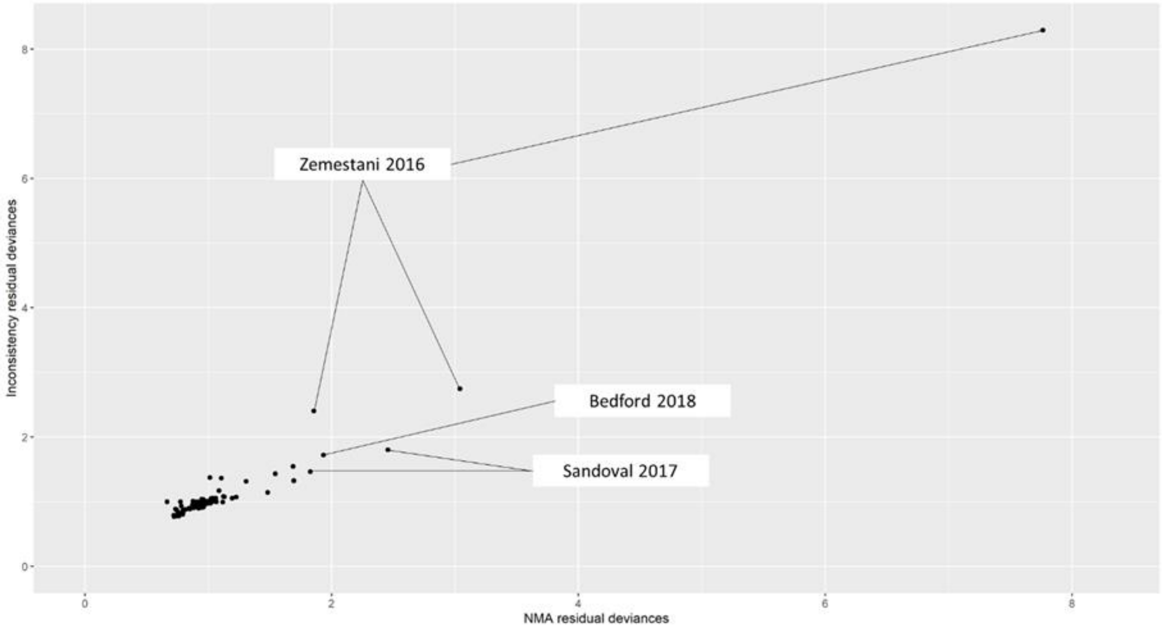

Remission in those randomised
Posterior mean residual deviances and DIC were similar in the NMA random effects consistency model and the inconsistency model, and there was no clear improvement in the prediction of data in individual studies by the inconsistency model. This suggested that there was no evidence of inconsistency (see Model fit statistics). However, both models poorly predicted data from two studies (Yang 2015, Rosso 2017), both of which investigated No treatment compared to an intervention from the Self-help class (Figure 4). The between-study heterogeneity was very similar in consistency and inconsistency models. Reported results are therefore based on the random-effects NMA model, assuming consistency. Moderate between trials heterogeneity was observed relative to the size of the intervention effect estimates [τ=0·45 (95% CrI 0·04 to 1·04)].

Figure 4: Deviance contributions for the random effects consistency and inconsistency models. Remission in those randomised – less severe depression

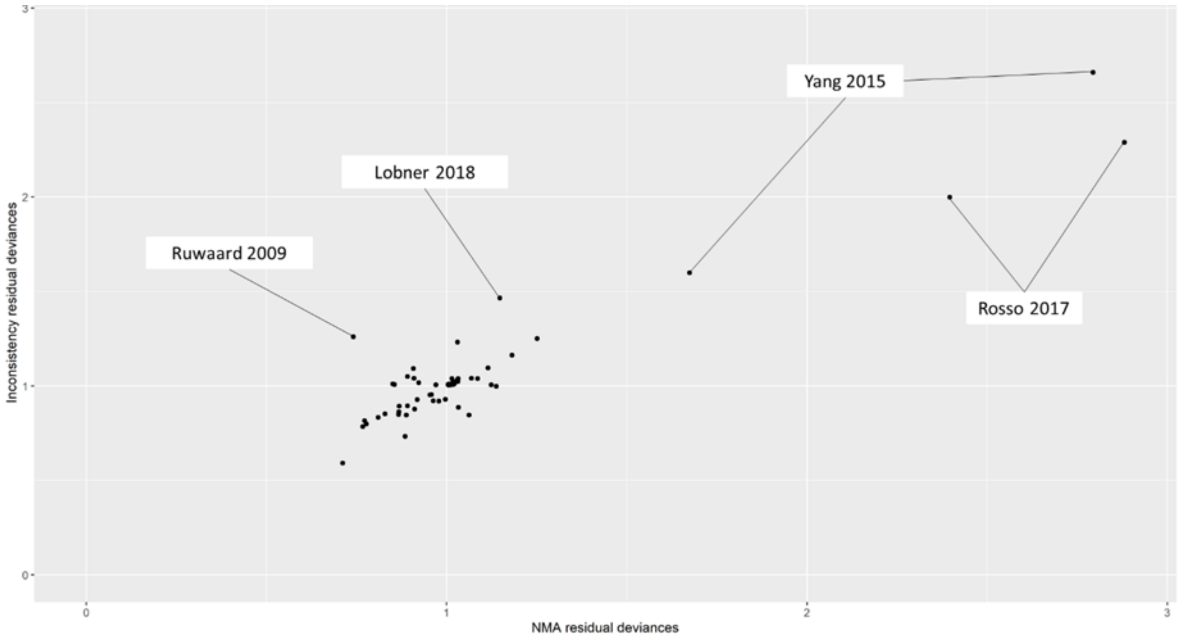

Treatment discontinuation for any reason
Lower posterior mean residual deviance and DIC values in the NMA random effects consistency model, as well as minimal improvement in the prediction of data in individual studies by the inconsistency model, suggested that there was no evidence of inconsistency (see Model fit statistics). The inconsistency model did not predict the data substantially better for any data points (Figure 5). The between-study heterogeneity was very similar in consistency and inconsistency models. As a pre-specified sensitivity analysis, a bias-adjusted model that accounted for small-study effects was fitted. The bias parameter for comparisons with active versus control or counselling treatments was estimated to be 0·14 (95%CrI -0·26, 0·58). Although the between study heterogeneity was slightly reduced (see Model fit statistics), the DIC remained the same as in the base-case consistency model. Reported results are therefore based on the random-effects NMA model, assuming consistency (Figure 6). Moderate between trials heterogeneity was observed relative to the size of the treatment effect estimates [(τ=0·53 (95% CrI 0·38 to 0·70)].

Figure 5: Deviance contributions for the random effects consistency and inconsistency models. Treatment discontinuation for any reason – less severe depression

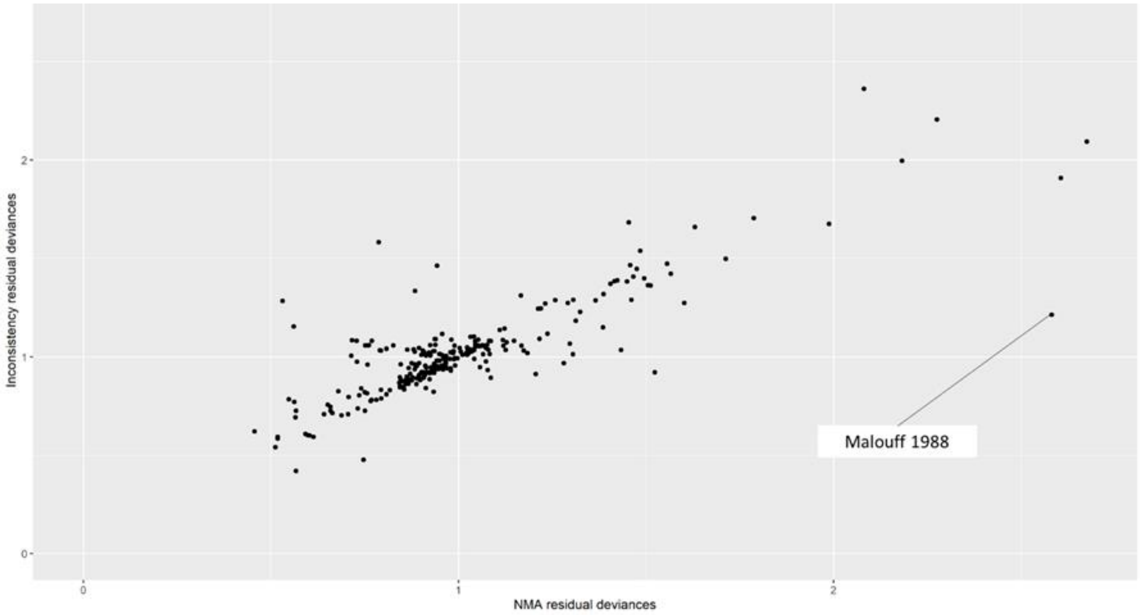

Figure 6: Forest plot for bias-adjusted versus base-case NMA of treatment discontinuation for any reason for each class versus TAU – less severe depression. Base-case model was selected.

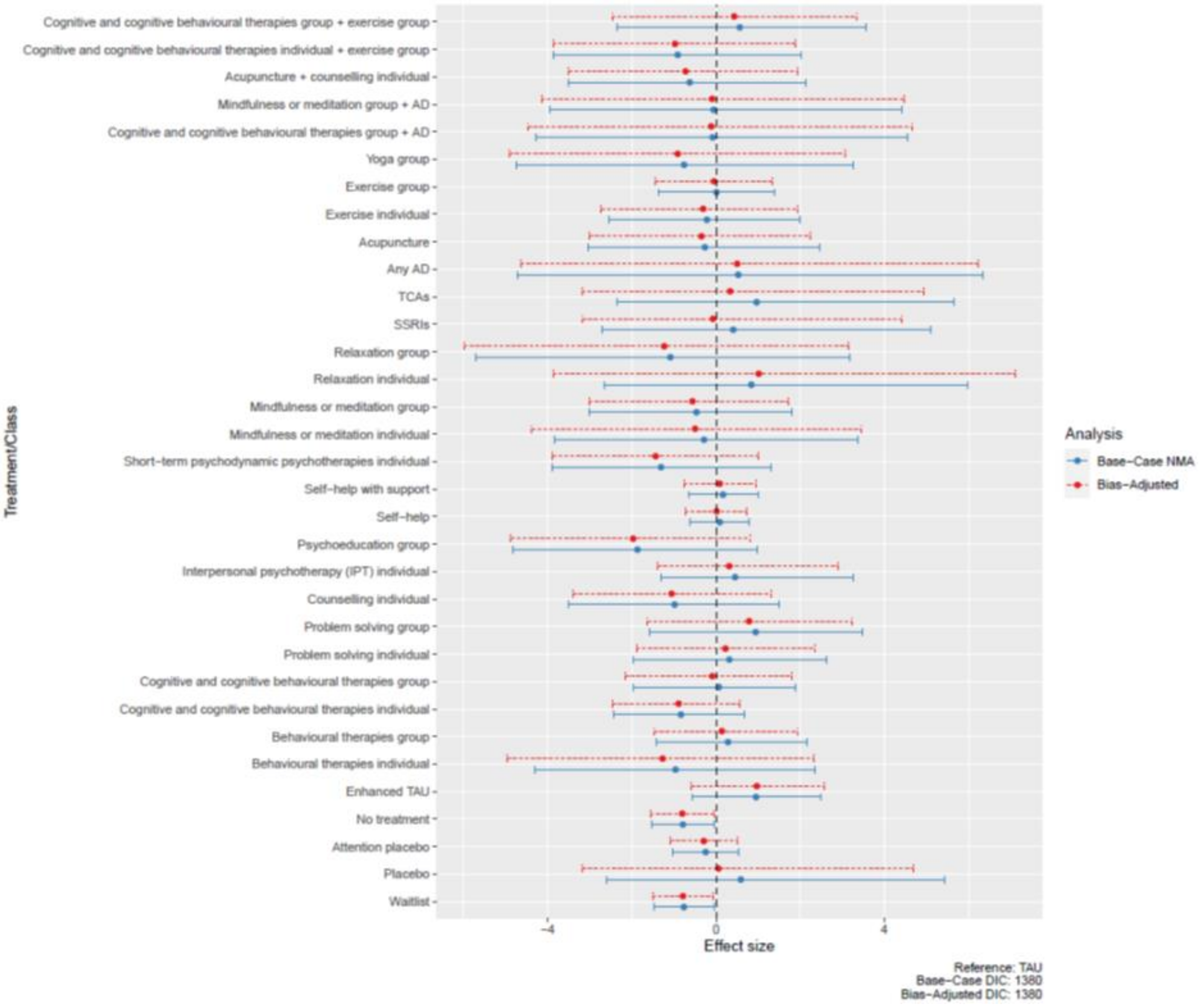

Treatment discontinuation due to side effects from medication, in those who discontinued treatment
NMA was not conducted for this outcome in this population.

Response in treatment completers
Although posterior mean residual deviances were very similar between the random-effects NMA consistency model and the inconsistency model, between-study heterogeneity was considerably lower in the inconsistency model, and prediction of some data points was substantially improved in the inconsistency model (see Model fit statistics). These were strongly suggestive of inconsistency, particularly in 4 studies comparing Waitlist, No treatment, Behavioural activation (BA) group and CBT group (under 15 sessions) (Zemestani 2016, Yang 2018, Gordon 1987, Zemstani 2017) (Figure 7). As a pre-specified sensitivity analysis, a bias-adjusted model that accounted for small-study effects was fitted. The bias parameter for comparisons with active versus control or counselling treatments was estimated to be 0·66 (95%CrI -0·95, 2·35). The between study heterogeneity was substantially reduced (see Model fit statistics), though it had a wide 95%CrI, and the prediction of data points improved such that these were similar between the bias-adjusted consistency NMA and the inconsistency model. This suggests that heterogeneity and inconsistency could be explained by small study effects. However, the residual deviance and DIC were similar between the base-case and bias-adjusted models, and for this reason the base-case model was selected. Results are therefore based on the random-effects consistency NMA model (Figure 8). High between trials heterogeneity was found relative to the size of the treatment effect estimates [τ=0·96 (95% CrI 0·71 to 1·28)].

Figure 7: Deviance contributions for the random effects consistency and inconsistency models. Response in treatment completers – less severe depression

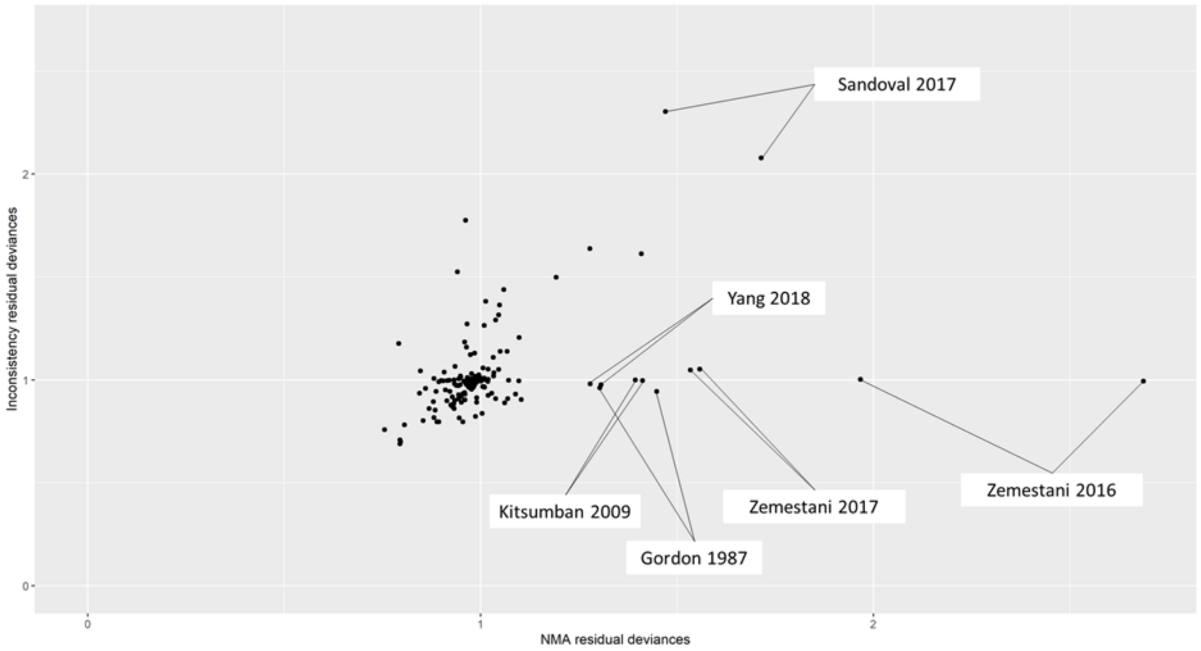

Figure 8: Forest plot for bias-adjusted versus base-case NMA of response in treatment completers for each class versus TAU – less severe depression. Base-case model was selected.

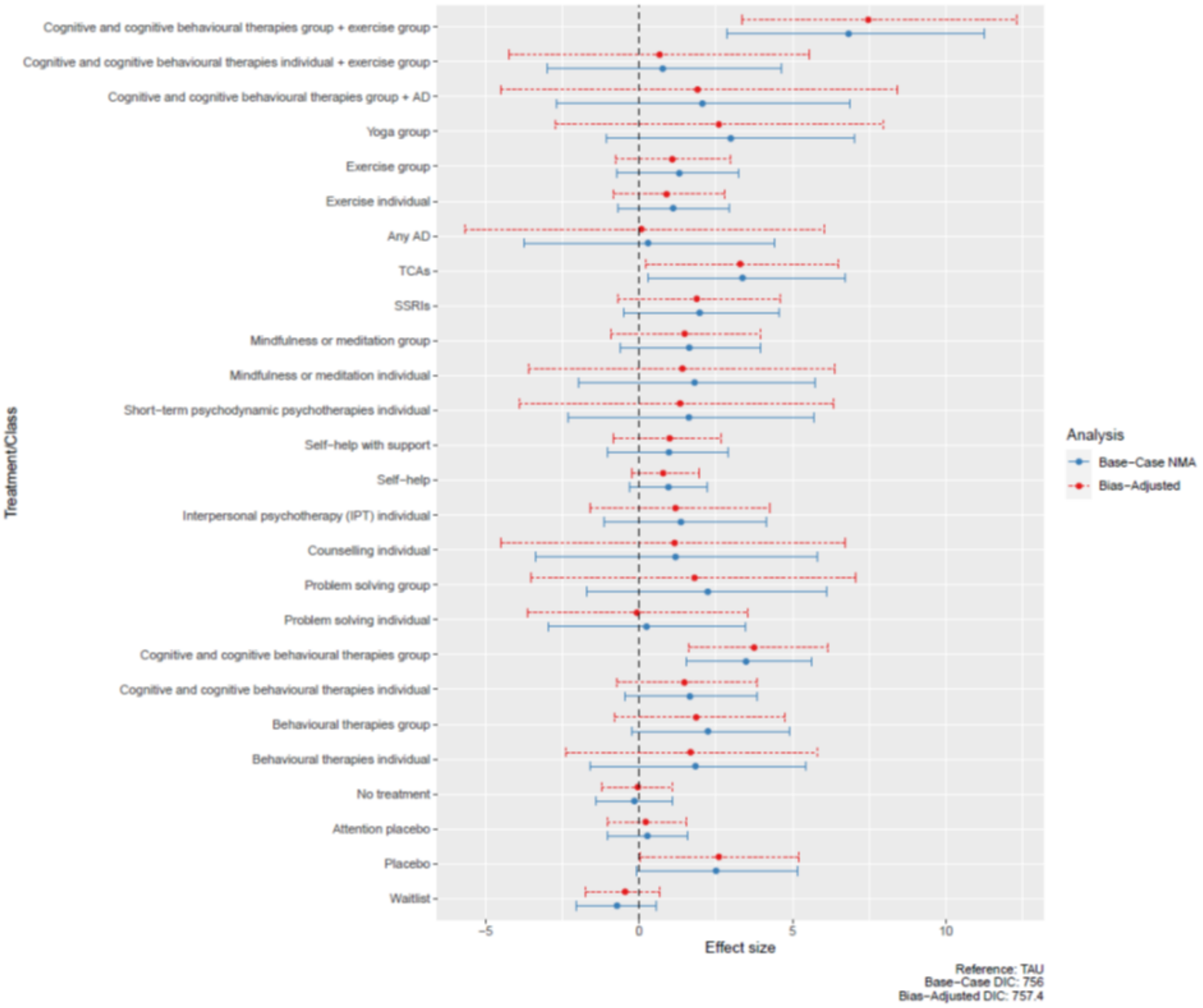

Remission in treatment completers

Posterior mean residual deviances were the same in the NMA random effects consistency model and the inconsistency model, and DIC was slightly lower. In addition to minimal improvement in the prediction of data in individual studies by the inconsistency model, this suggested that there was no evidence of inconsistency (see Model fit statistics). However, both models poorly predicted data from two studies (Yang 2015, Rosso 2017), both of which investigated No treatment compared to an intervention from the Self-help class (Figure 9). The between-study heterogeneity was very similar in consistency and inconsistency models (see Model fit statistics). Reported results are therefore based on the random-effects NMA model, assuming consistency. Moderate between trials heterogeneity was observed relative to the size of the treatment effect estimates [τ=0·35 (95% CrI 0·02 to 0·89)].

Figure 9: Deviance contributions for the random effects consistency and inconsistency models. Remission in treatment completers – less severe depression

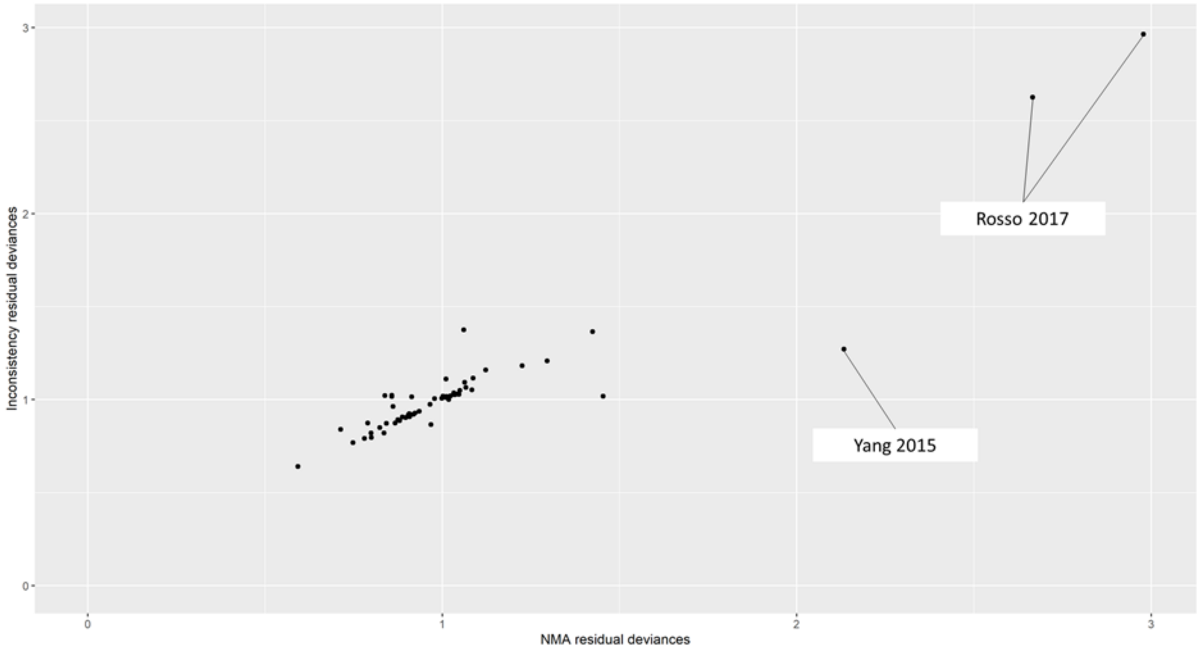

More severe depression

SMD of depression scale change scores
Between-study heterogeneity and posterior mean residual deviance were slightly lower in the inconsistency model than in the random effects consistency model (see Model fit statistics). The model was a reasonable fit to the data, with the exception of two very poorly fitting studies (Schweitzer 1991 and Sahranavard 2018). Schweitzer 1991 compared different regimens of venlafaxine, which may explain the poor fit for this study. The inconsistency model notably predicted the data in three studies much better than the consistency model, further adding evidence of inconsistency (Figure 10). As a pre-specified sensitivity analysis, a bias-adjusted model that accounted for small-study effects was fitted. The posterior mean residual deviance, DIC and between study heterogeneity was substantially reduced compared to the base-case consistency model (see Model fit statistics), and the bias parameter was negative (-2·57; 95%CrI -3·65 to -1·51), indicating that smaller studies tended to favour active interventions versus inactive controls or counselling. Reported results are therefore based on the bias-adjusted random-effects NMA model (Figure 11). Moderate between trials heterogeneity was found relative to the size of the treatment effect estimates [τ=0·20 (95% CrI 0·16 to 0·24)].

Figure 10: Deviance contributions for the random effects consistency and inconsistency models. SMD of depression scale change scores – more severe depression

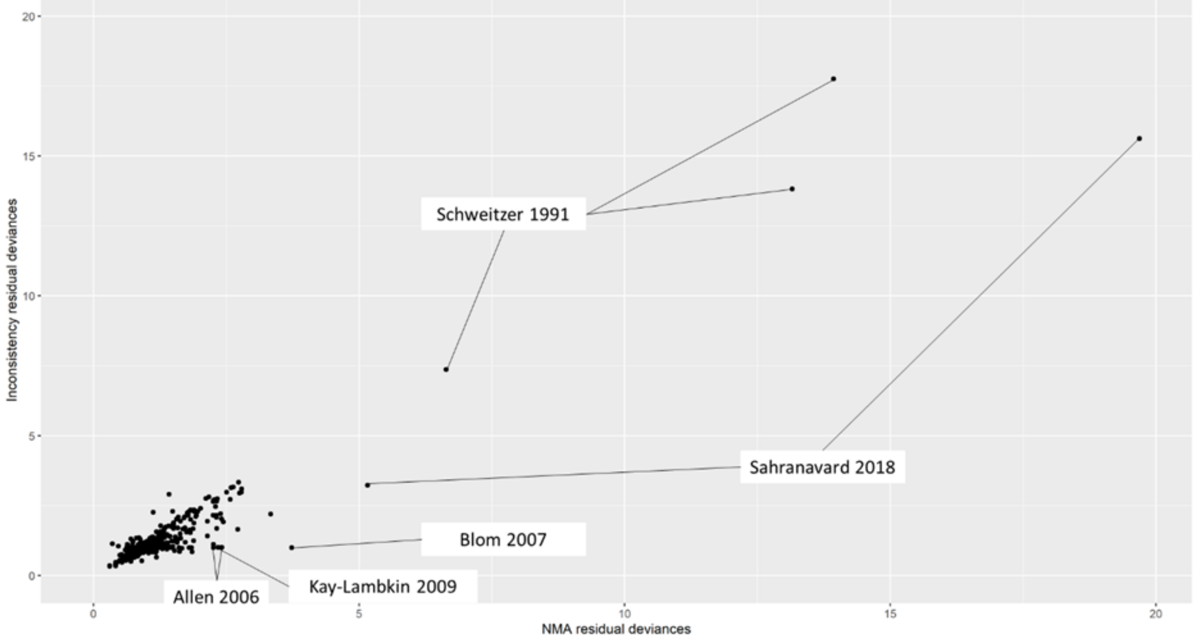

Figure 11: Forest plot for bias-adjusted versus base-case NMA of the SMD of depression scale change scores for each class versus pill placebo – more severe depression. Bias-adjusted model was selected.

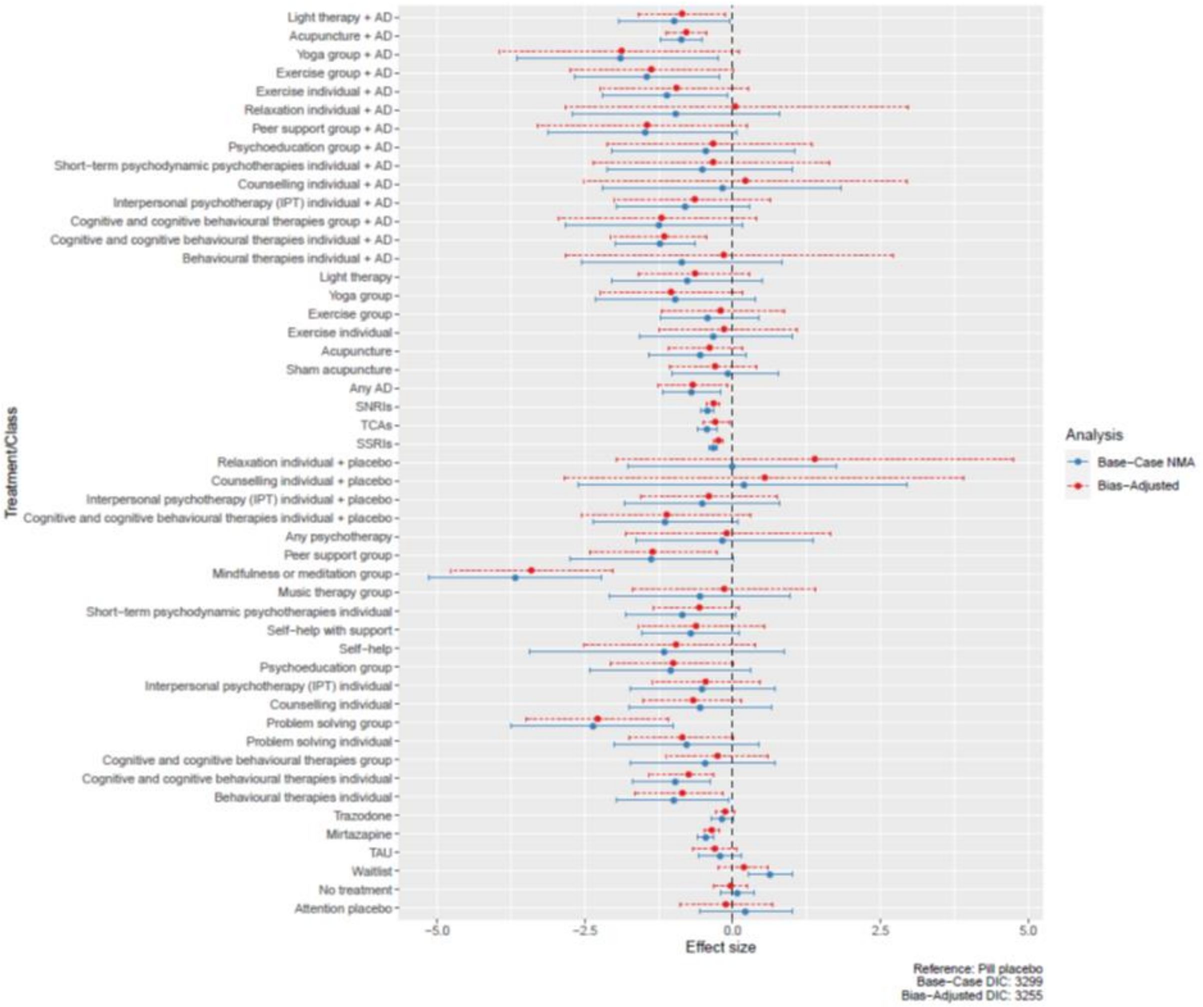

Response in those randomised
Lower posterior mean residual deviance and between study heterogeneity in the inconsistency model suggested evidence of inconsistency (see Model fit statistics). The inconsistency model notably predicted the data in one study (Sahranavard 2018) much better than the consistency model, further adding evidence of inconsistency (Figure 12). This study compared Waitlist, Dialectical behavioural therapy (DBT) individual and CBT group (under 15 sessions). Reported results are based on the random-effects NMA model, assuming consistency but should be interpreted with caution due to the identification of potential inconsistency. Relative to the size of the treatment effect estimates, moderate between trial heterogeneity was observed for this outcome [τ=0·26 (95% CrI 0·21 to 0·31)].

Figure 12: Deviance contributions for the random effects consistency and inconsistency models. Response in those randomised – more severe depression

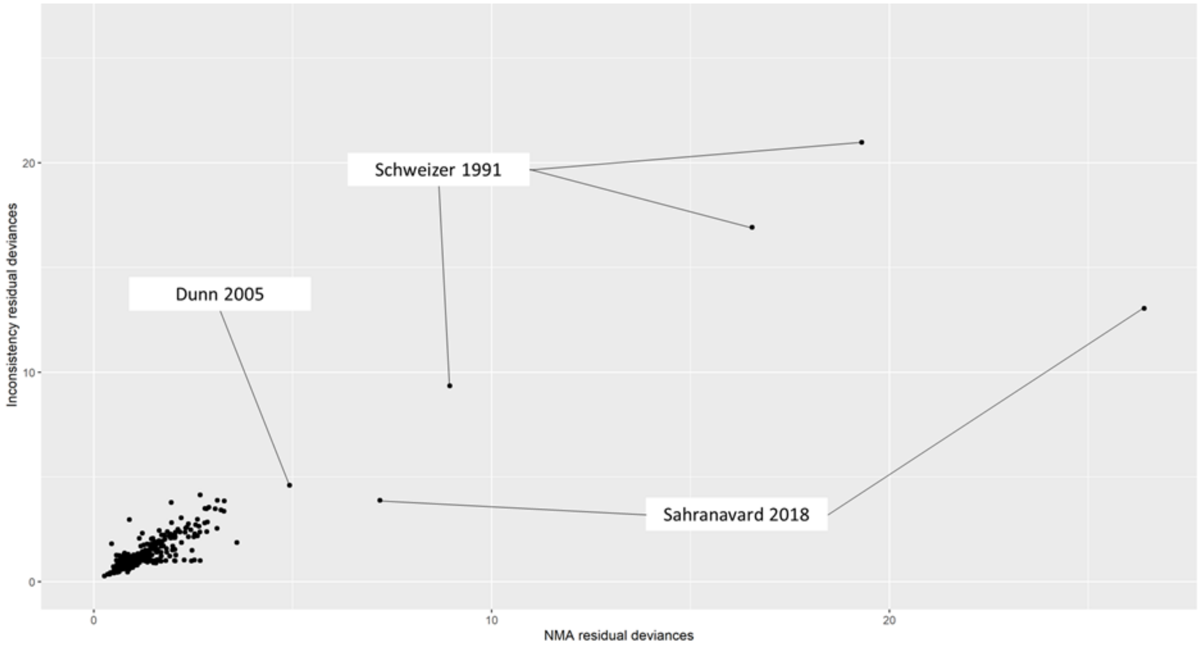

Remission in those randomised
No meaningful differences were observed in posterior mean residual deviance, though DIC was slightly lower in the random effects consistency model, and between-study heterogeneity slightly lower in the inconsistency model (see Model fit statistics). The prediction of several individual studies was worse in the consistency model, suggesting some evidence of inconsistency. These studies investigated Behavioural activation (BA) individual, CBT individual (15 sessions or over), Sertraline, Impiramine and Venafalxine (Figure 13). Reported results are based on the random-effects NMA model, assuming consistency. There was moderate between trial heterogeneity observed for this outcome [τ=0·27 (95% CrI 0·20 to 0·34)].

Figure 13: Deviance contributions for the random effects consistency and inconsistency models. Remission in those randomised – more severe depression

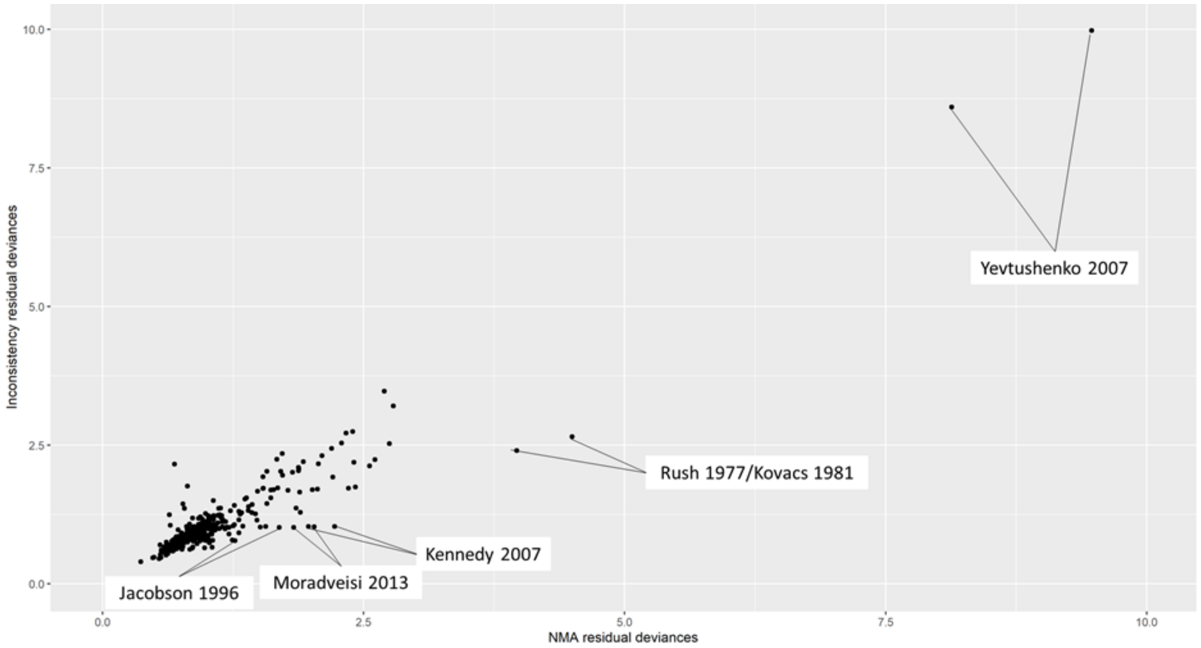

Treatment discontinuation for any reason

Although there was lower posterior mean residual deviance and DIC values in the NMA random effects consistency model, the between-study heterogeneity was lower in the inconsistency model (see Model fit statistics). The prediction of individual studies was similar in both models, apart from for one study (Sun 2013). This was for a zero arm to which a continuity correction had been added (Figure 14). As a pre-specified sensitivity analysis, a bias-adjusted model that accounted for small-study effects was fitted. The bias parameter for comparisons with active versus control or counselling treatments was estimated to be -0·35 (95%CrI -0·76, 0·04). The between study heterogeneity was slightly reduced and the DIC was lower than in the base-case consistency model (see Model fit statistics). Reported results are based on the bias-adjusted random effects NMA model, assuming consistency (Figure 15). Moderate between trials heterogeneity was observed relative to the size of the treatment effect estimates [τ=0·28 (95% CrI 0·22 to 0·33)].

Figure 14: Deviance contributions for the random effects consistency and inconsistency models. Treatment discontinuation for any reason – more severe depression

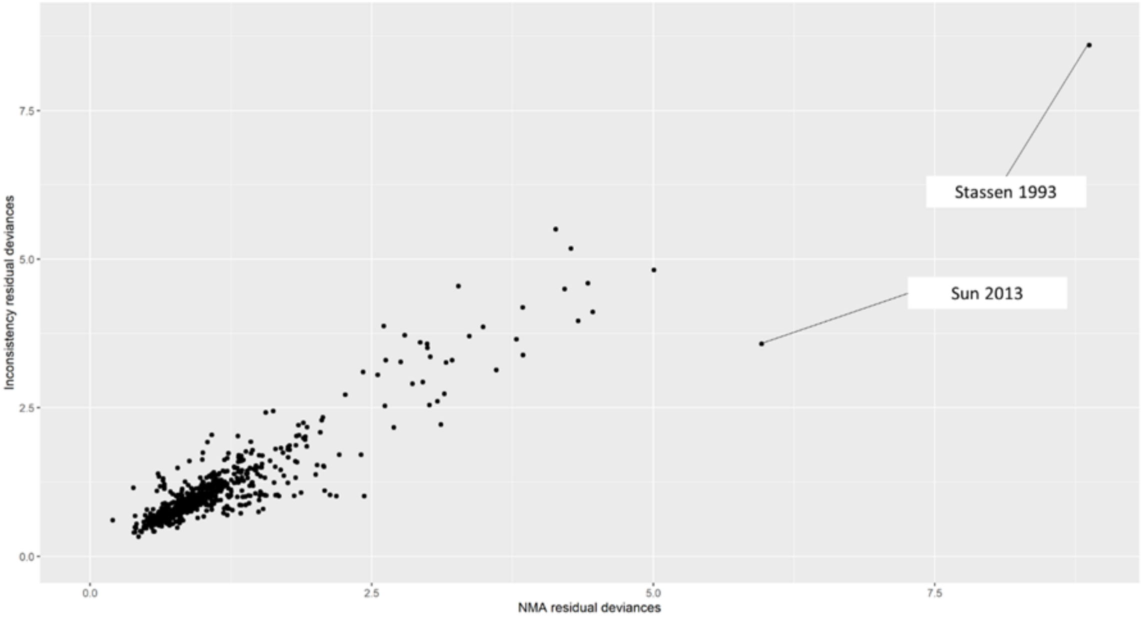

Figure 15: Forest plot for bias-adjusted versus base-case NMA of treatment discontinuation for any reason for each class versus pill placebo – more severe depression. Bias-adjusted model was selected.

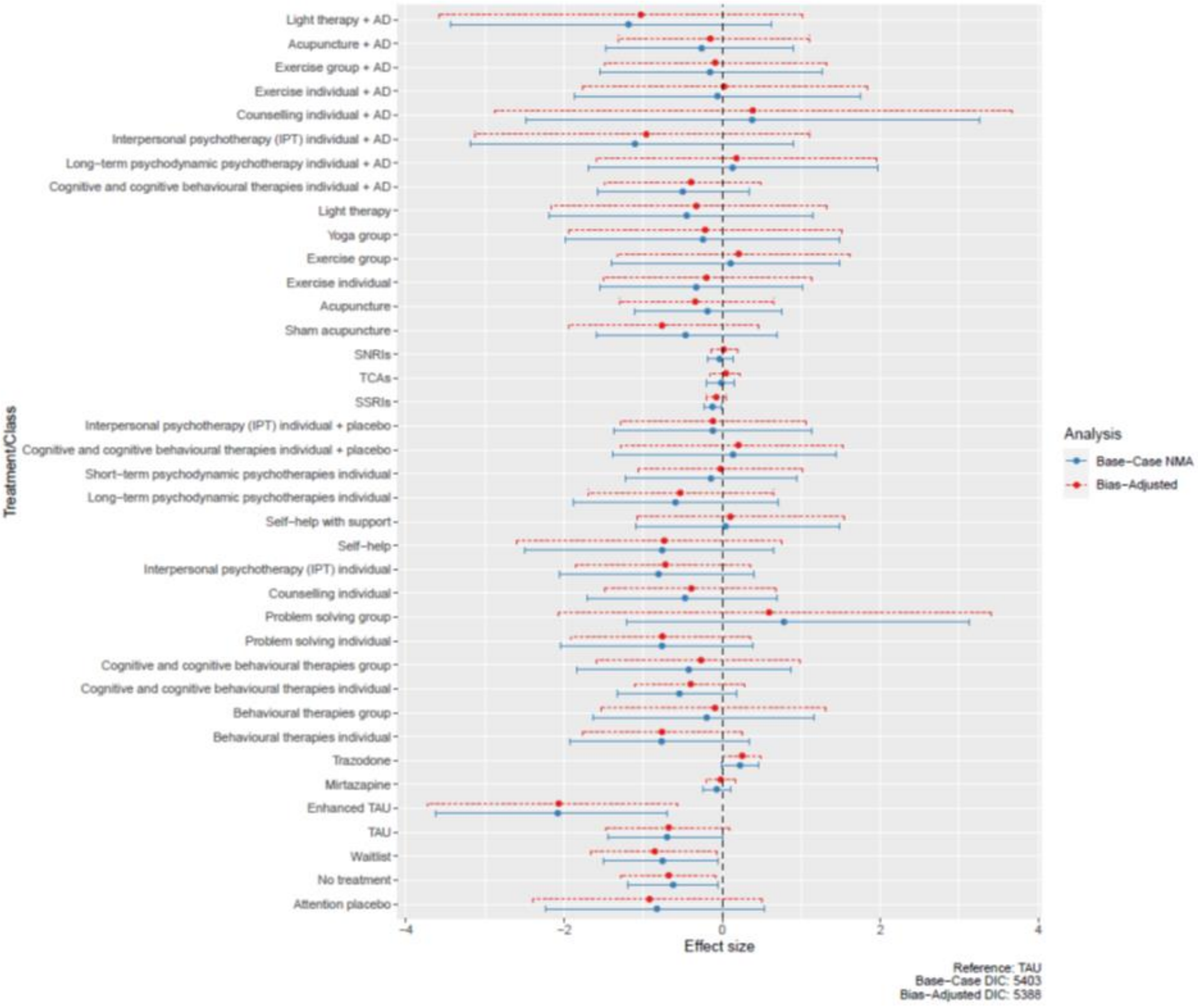

Treatment discontinuation due to side effects from medication, in those who discontinued treatment
Although there was lower posterior mean residual deviance and DIC values in the NMA random effects consistency model, the between-study heterogeneity was lower in the inconsistency model (see Model fit statistics). However, the prediction of individual studies was similar in both models (Figure 16). Reported results are therefore based on the random-effects NMA model, assuming consistency. Moderate between trials heterogeneity was observed relative to the size of the treatment effect estimates [τ=0·44 (95% CrI 0·33 to 0·55)].

Figure 16: Deviance contributions for the random effects consistency and inconsistency models. Treatment discontinuation due to side effects in those who discontinued treatment – more severe depression

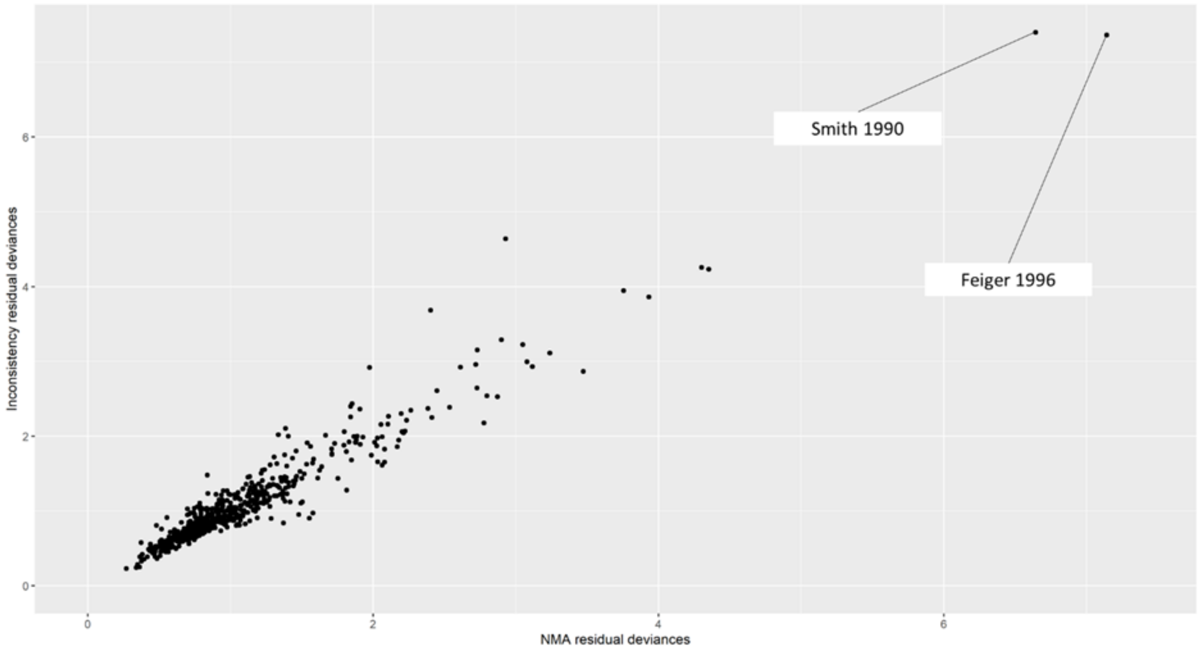

Response in treatment completers

Posterior mean residual deviances, DIC and between-study heterogeneity were all lower in the random-effects NMA consistency model than in the inconsistency model (see Model fit statistics). Prediction of data points were largely similar in both models, although for one study (Moradveisi 2013) the fit was substantially poorer in the consistency model, due to one arm in which the number of responders was equal to the number of completers (Figure 17). As a pre-specified sensitivity analysis, a bias-adjusted model that accounted for small-study effects was fitted. The bias parameter for comparisons with active versus control or counselling interventions was estimated to be 0·86 (95%CrI 0·33, 1·42). This indicated that smaller studies were likely to be biased in favour of active interventions versus control or counselling interventions. The posterior mean residual deviance, DIC and between study heterogeneity were substantially reduced compared to the base-case consistency model (see Model fit statistics). Reported results are therefore based on the bias-adjusted random-effects NMA model (Figure 18). Moderate between trials heterogeneity was found relative to the size of the treatment effect estimates [τ=0·60 (95% CrI 0·52 to 0·68)].

Figure 17: Deviance contributions for the random effects consistency and inconsistency models. Response in treatment completers – more severe depression

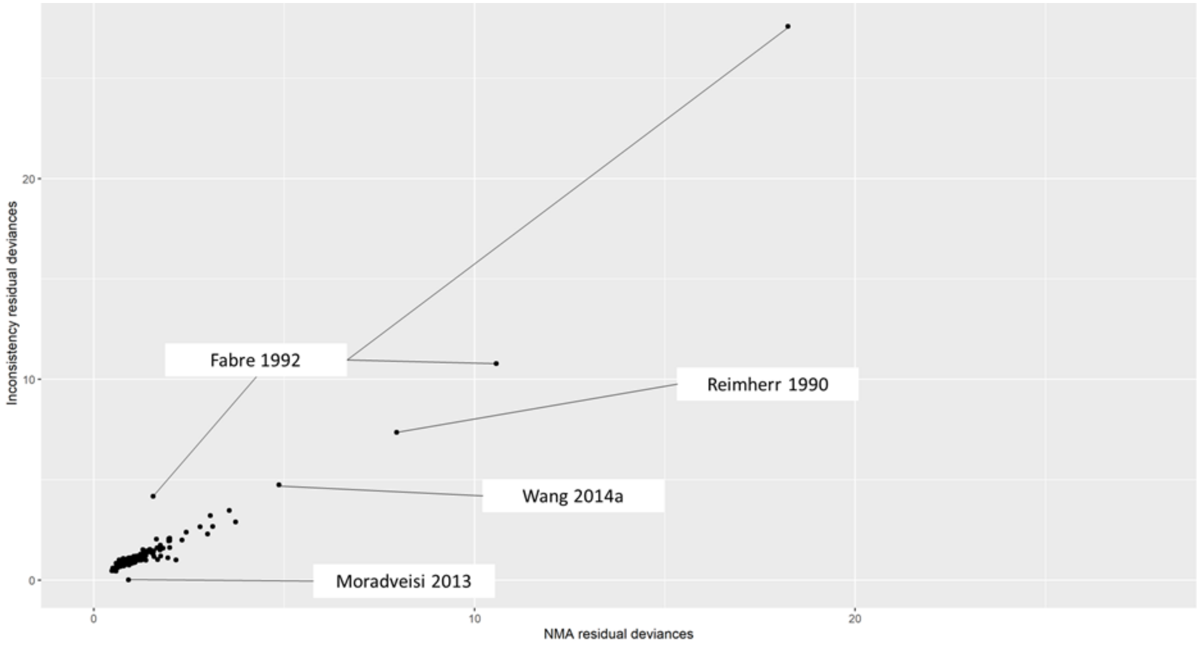

Figure 18: Forest plot for bias-adjusted versus base-case NMA of response in treatment completers for each class versus pill placebo – more severe depression. Bias-adjusted model was selected.

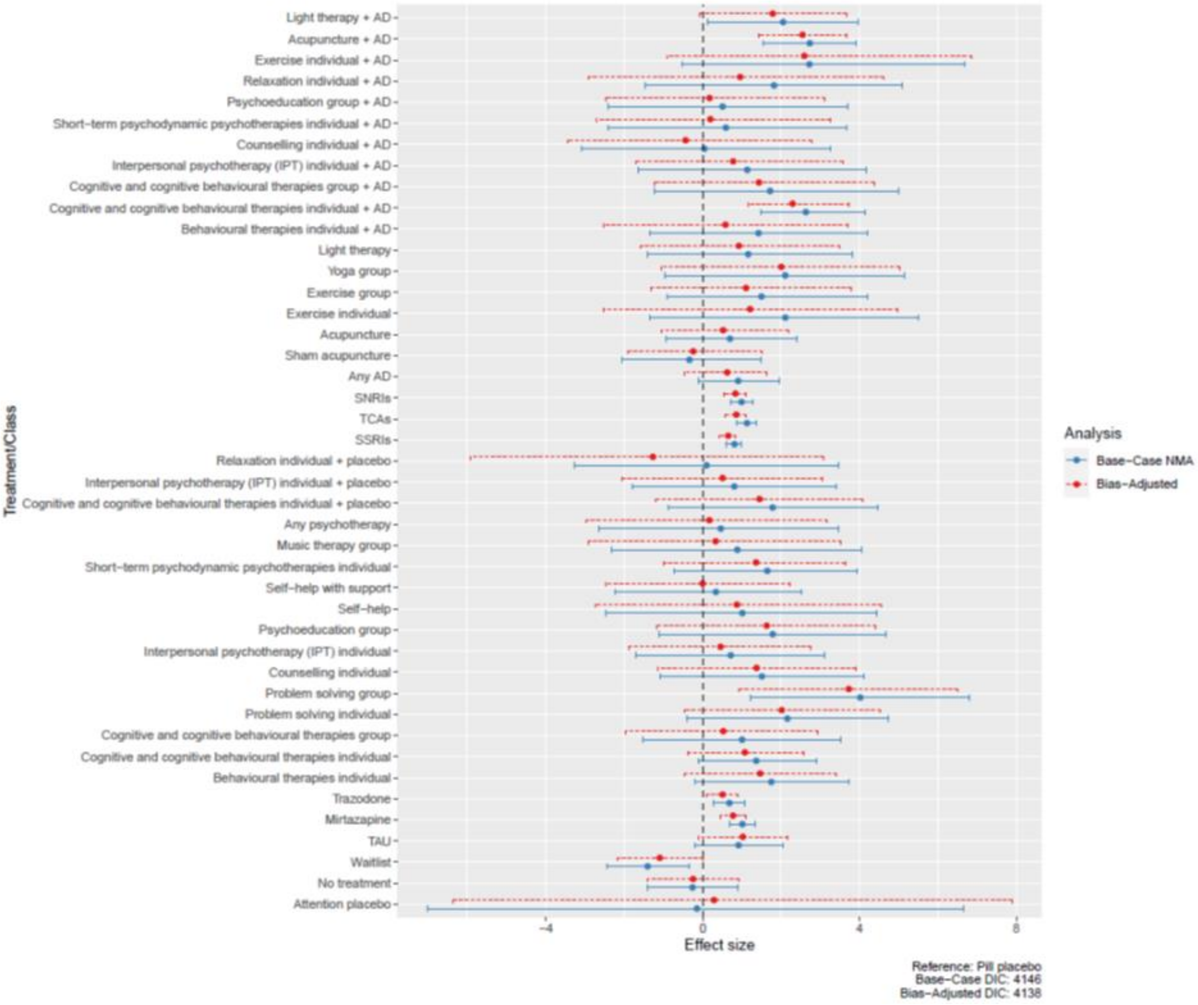

Remission in treatment completers

Although there was lower posterior mean residual deviance and DIC values in the NMA random effects consistency model, the between-study heterogeneity was lower in the inconsistency model (see Model fit statistics). The prediction of individual studies was notably worse in one study (Rush 1977/Kovacs 1981), which investigated CBT individual (15 sessions or over) versus Impiramine (Figure 19). Results are based on the random-effects NMA model, assuming consistency. Low between trial heterogeneity was observed for this outcome [τ=0·14 (95% CrI 0·02 to 0·24)].

Figure 19: Deviance contributions for the random effects consistency and inconsistency models. Remission in treatment completers – more severe depression

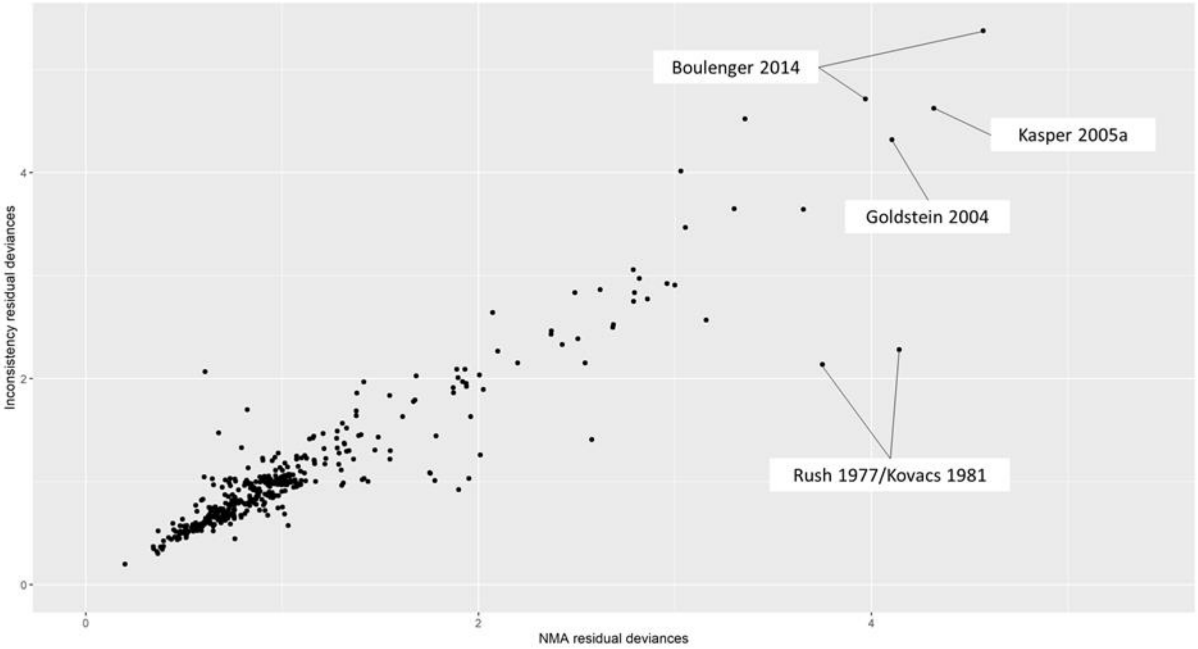

Risk of bias of studies included in the NMA

Less severe depression

Figure 20. Risk of bias graph: reviewers' judgements about each risk-of-bias item presented as percentages across all included studies.

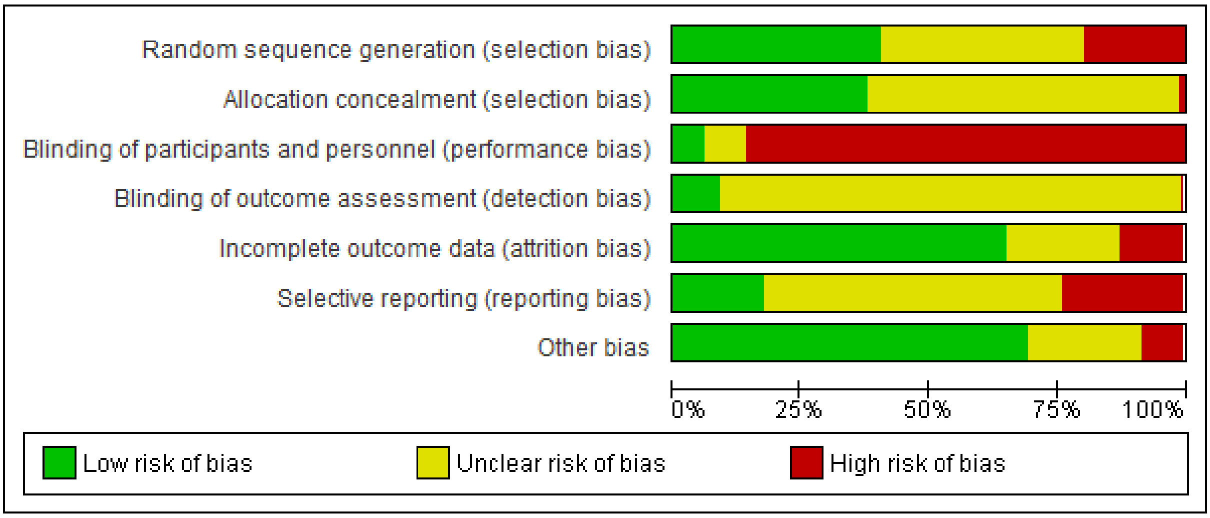

For reviewers' risk-of-bias judgements presented by study see Appendix 2, 'LS depression -included studies' sheet.

More severe depression

Figure 21 Risk of bias graph: reviewers' judgements about each risk-of-bias item presented as percentages across all included studies.

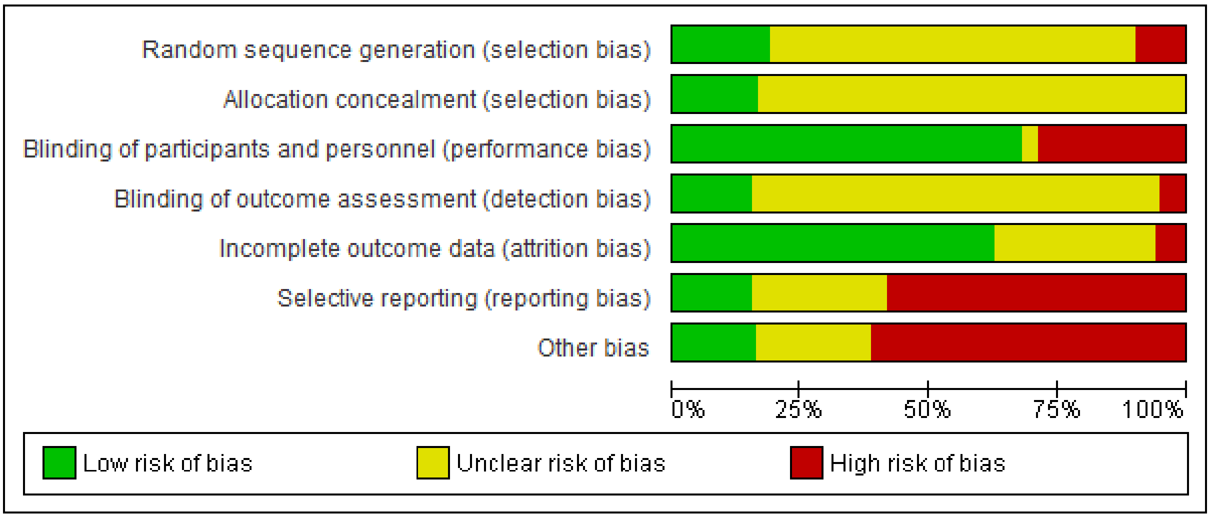

For reviewers' risk-of-bias judgements presented by study see Appendix 2, 'MS depression -included studies' sheet.

Quality assessment of the evidence - results

Less severe depression

Risk of bias

Generally the standard of reporting in studies was quite low, as demonstrated by the risk of bias summary diagram. Of the 142 studies included in the NMAs for less severe depression, 56 were at low risk of bias for allocation method and 53 were at low risk of bias for allocation concealment. Trials of psychological therapies were typically considered at high risk of bias for participant and provider blinding, although it is difficult to quantify in risk of bias ratings it is also important to bear in mind that the rate of side effects may also make it difficult to maintain blinding in pharmacological trials. Across interventions, eight trials were at low risk of bias for blinding participants and providers. Assessor blinding was considered for all trials including those using self-report measures: 14 were at low risk of bias, 127 were unclear, and high risk in one trial. For attrition bias, 90 trials were at low risk of bias, unclear risk in 33 trials, and 19 trials were at high risk of bias. Other sources of bias, potential or actual (for instance, potential conflicts of interest associated with funding), were identified in 45 RCTs.

Model goodness of fit and inconsistency

Evidence of inconsistency was identified in the analysis of response in completers; some evidence of local-level inconsistency was identified across all other analyses.

Selective outcome reporting and publication bias

Bias adjustment models on the SMD of depression scale change scores, treatment discontinuation for any reason and response in completers were developed to assess potential bias associated with small study size. Between study heterogeneity and posterior mean residual deviance were lower in the bias-adjusted model that accounted for small study effects for the SMD outcome, suggesting some evidence of small study bias in comparisons of active versus inactive interventions or counselling.

Indirectness

In the context of the NMA, indirectness refers to potential differences across the populations, interventions and outcomes of interest, and those included in the relevant studies that informed the NMA.

A key assumption when conducting NMA is that the populations included in all RCTs considered in the NMA are similar. However, participants in pharmacological and non-pharmacological (psychological or physical intervention) trials may differ to the extent that some participants find different interventions more or less acceptable in light of their personal circumstances and preferences (so that they might be willing to participate in a pharmacological trial but not a psychological one and vice versa). Similarly, self-help trials may recruit participants who would not seek or accept face-to-face interventions. However, a number of trials included in the NMA have successfully recruited participants who are willing to be randomised to either pharmacological or psychological intervention and to either self-help or face-to-face treatment. The NMAs have assumed that service users are willing to accept any of the interventions included in the analyses; in practice, treatment decisions may be influenced by individual values and goals, and people’s preferences for different types of interventions. These factors were taken into account when formulating recommendations.

In addition, to explore the transitivity assumption in the context of participants in pharmacological and non-pharmacological trials, a sensitivity analysis on the SMD outcome was conducted after excluding trials with at least one pharmacological or combined intervention arm, where the combined intervention included a pharmacological element. The purpose was to compare the relative effects and rankings of non-psychological treatments between this sensitivity analysis and the base-case analysis. The comparison suggested only small changes after exclusion of pharmacological trials, probably because there were not many pharmacological trials included in this dataset (treatments for a new episode of less severe depression).

A post-hoc sensitivity analysis that included only RCTs rated as being at low risk of bias was conducted on the SMD outcome, which was the primary efficacy outcome. Such analysis was only possible to conduct for the domain of ‘attrition’ in the risk of bias tool, as this was the only domain that included a sufficient number of RCTs at low risk of bias, and a relatively wide range of treatment classes. This sub-group analysis showed no substantial difference in treatment effects compared with the base-case analysis, suggesting that bias from attrition was unlikely to be an effect modifier in this population.

Interventions of similar type were grouped in classes following the committee’s advice and considered in class models. These models allowed interventions within each class to have similar, but not identical, effects around a

class mean effect. Classes and interventions assessed in the NMAs were directly relevant to the classes and interventions of interest.

Outcomes reported in included studies were also the primary outcomes of interest, as agreed by the committee.

Imprecision

There were wide 95%CrI around mean effects and rankings, for most treatment classes versus the reference treatment (TAU) across all NMA outcomes. For the vast majority of treatment classes, the 95%CrI around relative effects versus TAU crossed the line of no effect.

Overall rating of the quality of the evidence

Based on the narrative assessment of the quality of the evidence using the domains considered as per a standard GRADE approach, the quality of the evidence was considered to be low.

More severe depression

Risk of bias

Generally the standard of reporting in studies was quite low, as demonstrated by the risk of bias summary diagram. Of the studies included in the NMAs for more severe depression, 106 were at low risk for allocation method, and 86 were at low risk of bias for allocation concealment. Trials of psychological therapies were typically considered at high risk of bias for participant and provider blinding, although it is difficult to quantify in risk of bias ratings it is also important to bear in mind that the rate of side effects may also make it difficult to maintain blinding in pharmacological trials. Across interventions, 364 trials were at low risk of bias for blinding participants and providers. Most reported outcomes were investigator-rated, and assessor blinding was considered for all trials: 82 were at low risk of bias, 423 were unclear, and high risk in 29 trials. For attrition bias, 330 trials were at low risk of bias, unclear risk in 173 trials, and 31 trials were at high risk of bias. For selective reporting bias, 77 trials were at low risk of bias, unclear risk in 143 trials, and 314 trials were at high risk of bias. Other sources of bias, predominantly potential conflict of interest based on the source of funding, were identified in 455 RCTs.

Model goodness of fit and inconsistency

Evidence of inconsistency was identified across most analyses, including analyses of the following outcomes:

- SMD of depression scale change scores
- Response in those randomised
- Remission in those randomised
- Treatment discontinuation for any reason
- Remission in treatment completers

Selective outcome reporting and publication bias

Bias adjustment models on the SMD of depression scale change scores, treatment discontinuation for any reason and response in completers were developed to assess potential bias associated with small study size. The posterior mean residual deviance, DIC and between study heterogeneity was substantially reduced in the bias adjustment model compared to the base-case consistency model for the SMD and response in completers outcomes, suggesting strong evidence of small study bias in comparisons of active versus inactive interventions or counselling. Regarding treatment discontinuation for any reason, the between study heterogeneity was slightly reduced and the DIC was lower compared with the base-case consistency model, suggesting some evidence of small study bias in comparisons of active versus inactive interventions or counselling.

Indirectness

In the context of the NMA, indirectness refers to potential differences across the populations, interventions and outcomes of interest, and those included in the relevant studies that informed the NMA.

A key assumption when conducting NMA is that the populations included in all RCTs considered in the NMA are similar. However, participants in pharmacological and non-pharmacological (psychological or physical intervention) trials may differ to the extent that some participants find different interventions more or less acceptable in light of their personal circumstances and preferences (so that they might be willing to participate in a pharmacological trial but not a psychological one and vice versa). Similarly, self-help trials may recruit participants who would not seek or accept face-to-face interventions. However, a number of trials included in the NMA have successfully recruited participants who are willing to be randomised to either pharmacological or psychological intervention and to either self-help or face-to-face treatment. The NMAs have assumed that service users are willing to accept any of the interventions included in the analyses; in practice, treatment decisions may be influenced by individual values and goals, and people’s preferences for different types of interventions. These factors were taken into account when formulating recommendations.

In addition, to explore the transitivity assumption in the context of participants in pharmacological and non-pharmacological trials, a sensitivity analysis on the SMD outcome was conducted after excluding trials with at least one pharmacological or combined intervention arm, where the combined intervention included a pharmacological element. The purpose was to compare the relative effects and rankings of non-psychological treatments between this sensitivity analysis and the base-case analysis. The comparison suggested some changes in effects and rankings after exclusion of pharmacological trials, and higher uncertainty in the effects, apparently because the majority of the evidence came from pharmacological trials in this dataset (treatments for a new episode of more severe depression).

A post-hoc sensitivity analysis that included only RCTs rated as being at low risk of bias was conducted on the SMD outcome, which was the primary efficacy outcome. Such analysis was only possible to conduct for the

domain of ‘attrition’ in the risk of bias tool, as this was the only domain that included a sufficient number of RCTs at low risk of bias, and a relatively wide range of treatment classes. This sub-group analysis showed no substantial difference in treatment effects compared with the base-case analysis, suggesting that bias from attrition was unlikely to be an effect modifier in this population.

Interventions of similar type were grouped in classes following the committee’s advice and considered in class models. These models allowed interventions within each class to have similar, but not identical, effects around a class mean effect. Classes and interventions assessed in the NMAs were directly relevant to the classes and interventions of interest.

Outcomes reported in included studies were also the primary outcomes of interest, as agreed by the committee.

Imprecision

There were wide 95%CrI around mean effects and rankings, for most treatment classes versus the reference treatment (pill placebo) across all NMA outcomes. For several treatment classes, the 95%CrI around relative effects versus pill placebo crossed the line of no effect.

Overall rating of the quality of the evidence

Based on the narrative assessment of the quality of the evidence using the domains considered as per a standard GRADE approach, the quality of the evidence was considered to be low-to-moderate.

Network meta-analysis results for all outcomes at the treatment class level. Relative effects versus the reference treatment.

Less severe depression

Standardised mean difference (SMD) of depression scale change scores

Bias-adjusted results

See Figure 3 in main article for the respective forest plots

Bias-adjusted results of the network meta-analysis of standardised mean difference (SMD) of depression scale change scores in adults with a new episode of less severe depression: posterior effects (mean SMD, 95%CrI) of all treatment classes versus treatment as usual (TAU) and treatment class rankings

Treatment class	N	SMD vs TAU (mean, 95% CrI)	Rank (mean, 95% CrI)
CT/CBT group + exercise group	25	**-2·51 (-4·42 to -0·61)**	2·92 (1 to 14)
Problem solving group	104	-1·52 (-3·24 to 0·23)	6·61 (1 to 26)
CT/CBT group	480	**-1·01 (-1·76 to -0·06)**	9·55 (3 to 22)
Mindfulness or meditation group + AD	15	-1·23 (-5·14 to 2·80)	12·22 (1 to 32)
Behavioural therapies group	340	-0·73 (-1·95 to 0·50)	13·09 (3 to 28)
CT/CBT individual	481	-0·73 (-1·78 to 0·36)	13·14 (4 to 27)
TCAs	136	-0·83 (-2·18 to 0·53)	13·27 (3 to 29)
CT/CBT group + AD	32	-1·00 (-4·47 to 2·61)	13·34 (1 to 32)
Acupuncture + counselling individual	40	-0·78 (-2·57 to 1·02)	13·37 (2 to 31)
Yoga group	73	-0·73 (-2·43 to 0·98)	13·83 (2 to 31)
Acupuncture	40	-0·69 (-2·50 to 1·13)	14·26 (2 to 31)
Mindfulness or meditation group	376	-0·62 (-1·77 to 0·35)	14·47 (4 to 28)
Behavioural therapies individual	147	-0·63 (-2·48 to 1·28)	14·72 (2 to 31)
Pill placebo	301	-0·69 (-1·87 to 0·45)	15·09 (4 to 29)
SSRIs	207	-0·64 (-1·87 to 0·53)	15·90 (4 to 30)
Mindfulness or meditation individual	20	-0·52 (-3·10 to 2·22)	16·09 (1 to 32)
Short-term PDPT individual	49	-0·48 (-2·96 to 2·03)	16·49 (2 to 32)
IPT individual	153	-0·5 (-1·94 to 0·83)	16·93 (4 to 30)
Relaxation group	63	-0·42 (-2·19 to 1·20)	17·84 (3 to 32)
Exercise group	199	-0·37 (-3·56 to 2·79)	17·91 (1 to 32)
Self-help with support	1,286	-0·33 (-0·77 to 0·08)	18·22 (11 to 25)
Relaxation individual	13	-0·41 (-3·07 to 2·23)	18·39 (1 to 32)
Counselling individual	55	-0·20 (-2·82 to 2·5)	19·20 (2 to 32)
Exercise individual	250	-0·26 (-1·73 to 1·15)	19·43 (4 to 31)
Self-help without/with minimal support	4,922	-0·27 (-0·66 to 0·09)	19·51 (13 to 25)
CT/CBT individual + exercise group	18	-0·18 (-2·75 to 2·44)	19·78 (2 to 32)
Psychoeducation group	22	-0·09 (-2·07 to 1·96)	20·80 (3 to 32)
Attention placebo	935	-0·16 (-0·61 to 0·25)	21·52 (14 to 28)
Problem solving individual	98	0·17 (-1·53 to 1·91)	24·28 (6 to 32)
TAU	815	Reference	24·35 (18 to 30)
Enhanced TAU	36	0·16 (-0·81 to 1·13)	24·90 (11 to 32)
Waitlist	3,555	0·17 (-0·21 to 0·54)	26·56 (21 to 31)

Treatment classes ordered from best to worst, according to mean ranking. Negative effect values indicate a favourable outcome for treatment classes compared with TAU. Results where 95% CrI do not cross the no effect line are shown in bold.
AD: antidepressant; CBT: cognitive behavioural therapy; CrI: credible intervals; CT: cognitive therapy; IPT: interpersonal psychotherapy; PDPT: psychodynamic psychotherapy; SMD: standardised mean difference; SSRIs: selective serotonin uptake inhibitors; TAU: treatment as usual; TCAs: tricyclic antidepressants

Base-case results

Base-case forest plots of standardised mean difference (SMD) of depression scale change scores in adults with a new episode of less severe depression: effects of treatment classes versus treatment as usual (TAU, N=815) Values on the left side of the vertical axis indicate better effect compared with TAU. Effects are shown only for treatment classes with N ≥ 50, plus short-term psychodynamic psychotherapy (N=49).

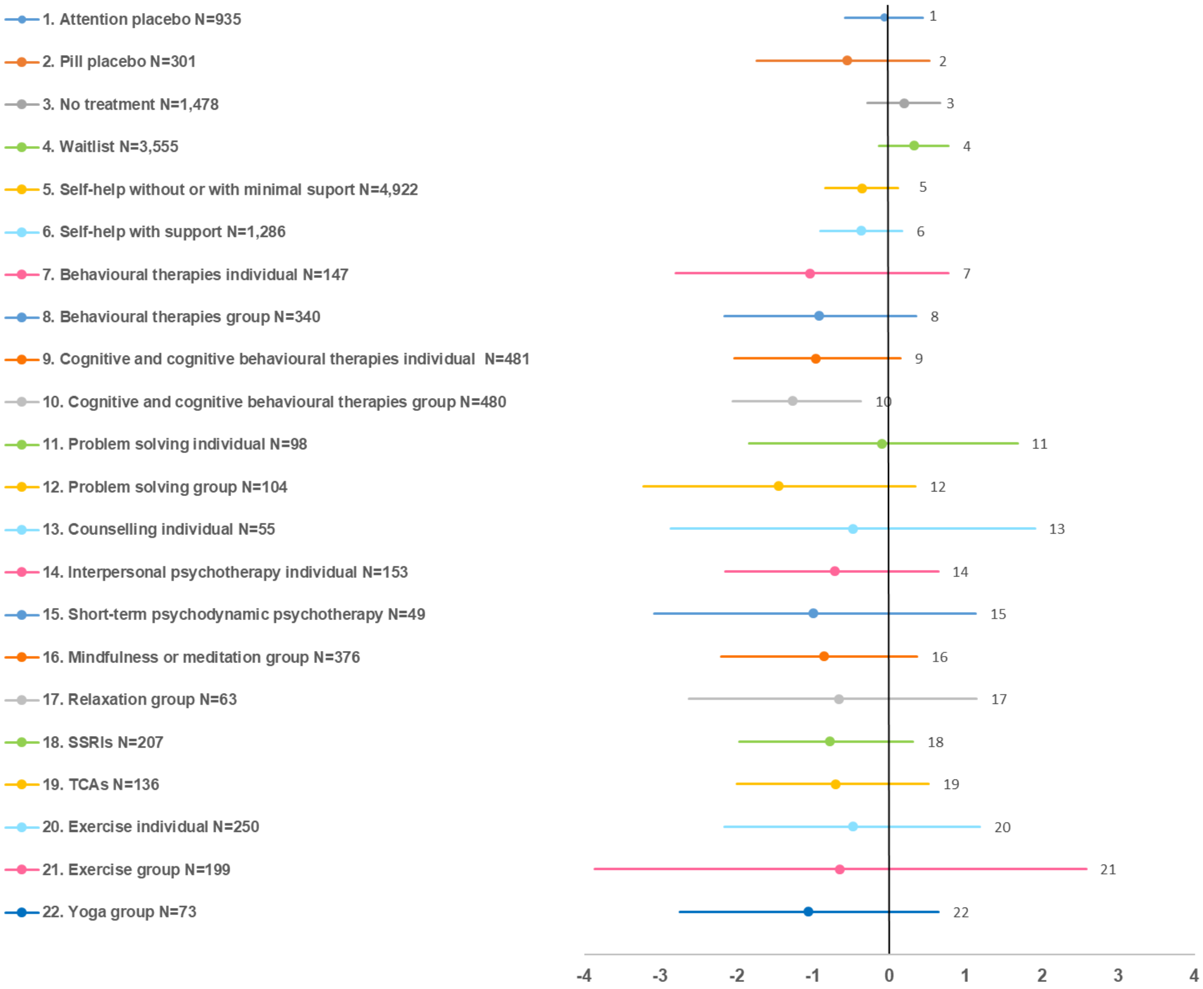

SSRIs: selective serotonin uptake inhibitors; TCAs: tricyclic antidepressants

Base-case results of the network meta-analysis of standardised mean difference (SMD) of depression scale change scores in adults with a new episode of less severe depression: posterior effects (mean SMD, 95%CrI) of all treatment classes versus treatment as usual (TAU) and treatment class rankings

Treatment class	N	SMD vs TAU (mean, 95% CrI)	Rank (mean, 95% CrI)
CT/CBT group + exercise group	25	**-2·76 (-4·77 to -0·77)**	2·76 (1 to 14)
Problem solving group	104	-1·45 (-3·22 to 0·35)	8·65 (1 to 28)
CT/CBT group	480	**-1·27 (-2·05 to -0·38)**	8·92 (3 to 20)
Mindfulness or meditation group + AD	15	-1·54 (-4·17 to 1·07)	9·95 (1 to 31)
CT/CBT group + AD	32	-1·27 (-3·79 to 1·26)	11·87 (1 to 32)
Yoga group	73	-1·06 (-2·75 to 0·65)	12·18 (2 to 31)
Behavioural therapies individual	147	-1·04 (-2·80 to 0·77)	12·46 (2 to 30)
CT/CBT individual	481	-0·96 (-2·03 to 0·14)	12·64 (4 to 26)
Mindfulness or meditation individual	20	-1·03 (-3·04 to 1·01)	13·04 (2 to 31)
Behavioural therapies group	340	-0·92 (-2·16 to 0·36)	13·36 (3 to 28)
Short-term PDPT individual	49	-0·99 (-3·08 to 1·14)	13·50 (2 to 31)
Acupuncture + counselling individual	40	-0·94 (-2·84 to 0·95)	13·88 (2 to 31)
Mindfulness or meditation group	376	-0·85 (-2·20 to 0·36)	14·21 (3 to 29)
Acupuncture	40	-0·87 (-2·77 to 1·03)	14·67 (2 to 31)
Relaxation individual	13	-0·82 (-2·94 to 1·35)	15·28 (2 to 32)
SSRIs	207	-0·77 (-1·97 to 0·31)	15·35 (4 to 29)
IPT individual	153	-0·71 (-2·15 to 0·64)	16·21 (4 to 30)
TCAs	136	-0·70 (-2·00 to 0·52)	16·29 (4 to 30)
Exercise group	199	-0·65 (-3·86 to 2·58)	16·75 (1 to 32)
Relaxation group	63	-0·66 (-2·63 to 1·15)	16·99 (2 to 32)
Pill placebo	301	-0·55 (-1·74 to 0·53)	18·45 (5 to 30)
Counselling individual	55	-0·47 (-2·87 to 1·91)	18·70 (2 to 32)
Exercise individual	250	-0·48 (-2·16 to 1·18)	18·88 (3 to 32)
CT/CBT individual + exercise group	18	-0·39 (-2·40 to 1·67)	19·69 (3 to 32)
Self-help with support	1,286	-0·36 (-0·90 to 0·17)	20·82 (14 to 27)
Psychoeducation group	22	-0·27 (-2·26 to 1·77)	20·86 (3 to 32)
Self-help without/with minimal support	4,922	-0·36 (-0·84 to 0·11)	20·86 (15 to 26)
Problem solving individual	98	-0·10 (-1·83 to 1·68)	23·20 (5 to 32)
Attention placebo	935	-0·06 (-0·57 to 0·44)	25·24 (19 to 30)
TAU	815	Reference	25·95 (19 to 31)
Enhanced TAU	36	0·28 (-0·90 to 1·47)	27·20 (13 to 32)
Waitlist	3,555	0·32 (-0·13 to 0·78)	29·20 (25 to 32)

Treatment classes ordered from best to worst, according to mean ranking. Negative effect values indicate a favourable outcome for treatment classes compared with TAU. Results where 95% CrI do not cross the no effect line are shown in bold.
AD: antidepressant; CBT: cognitive behavioural therapy; CrI: credible intervals; CT: cognitive therapy; IPT: interpersonal psychotherapy; PDPT: psychodynamic psychotherapy; SMD: standardised mean difference; SSRIs: selective serotonin uptake inhibitors; TAU: treatment as usual; TCAs: tricyclic antidepressants

Sensitivity analysis – inclusion of non-pharmacological RCTs

Forest plot comparing class-level results following exclusion of pharmacological trials from the NMA, results from bias-adjusted NMA, and results of the NMA base-case analysis: standardised mean difference (SMD) of depression scale change scores in adults with a new episode of less severe depression. Values on the left side of the vertical axis indicate better effect compared with TAU.

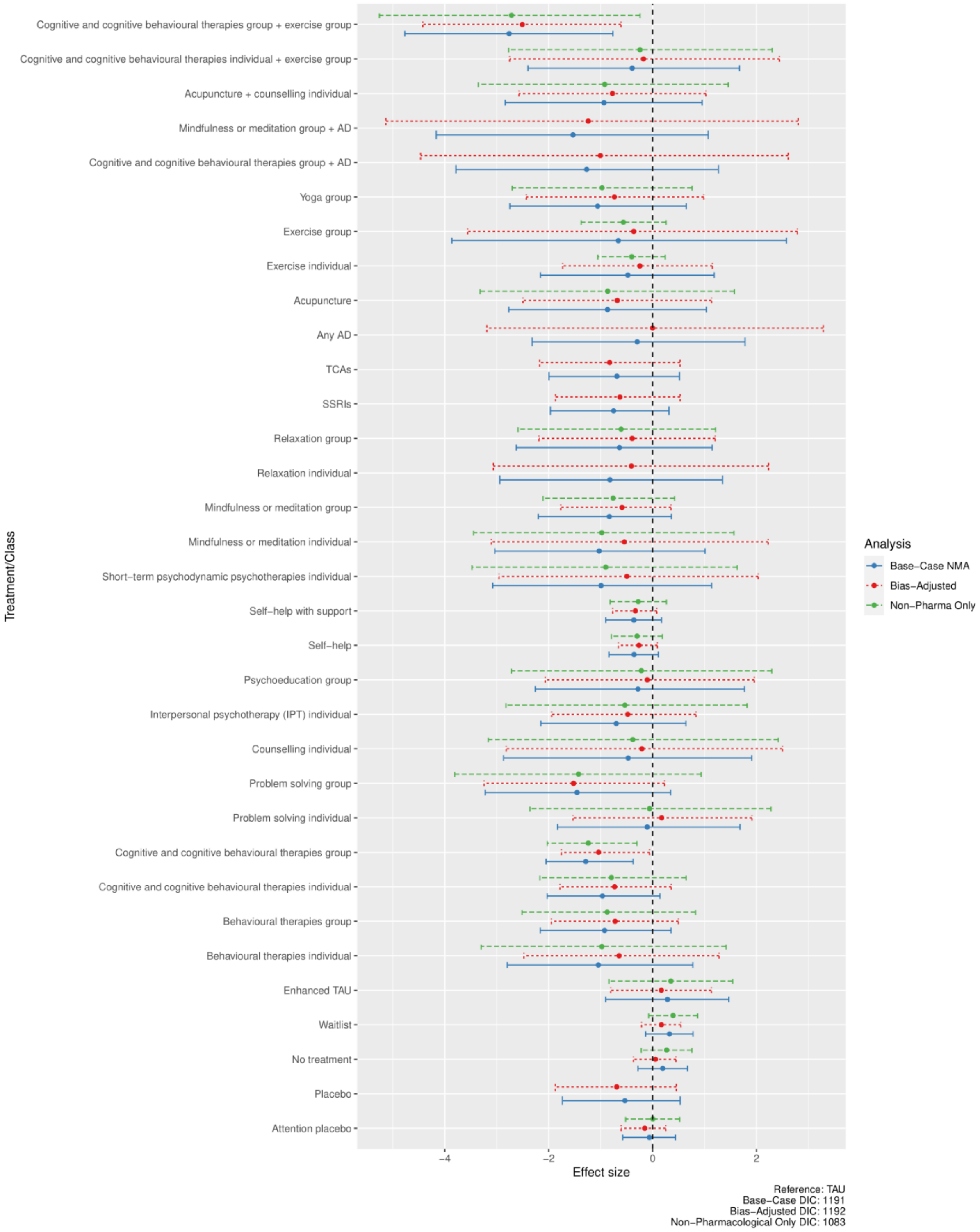

Comparison of results following exclusion of pharmacological trials from the NMA and results of the NMA base-case analysis: standardised mean difference (SMD) of depression scale change scores in adults with a new episode of less severe depression

Non-pharmacological dataset			Full dataset – base-case analysis		
**Treatment class**	**N**	**Effect vs TAU (mean SMD, 95%CrI)**	**Treatment class**	**N**	**Effect vs TAU (mean SMD, 95%CrI)**
CT/CBT group + exercise group	25	**-2·72 (-5·26 to -0·24)**	CT/CBT group + exercise group	25	**-2·76 (-4·77 to -0·77)**
CT/CBT group	480	**-1·22 (-2·03 to -0·30)**	Problem solving group	104	-1·45 (-3·22 to 0·35)
Problem solving group	104	-1.43 (-3.81 to 0.93)	CT/CBT group	480	**-1.27 (-2.05 to -0.38)**
Yoga group	73	-0.97 (-2.70 to 0.76)	Yoga group	73	-1.06 (-2.75 to 0.65)
Behavioural therapies individual	147	-0.97 (-3.30 to 1.41)	Behavioural therapies individual	147	-1.04 (-2.80 to 0.77)
Mindfulness or meditation individual	20	-0.97 (-3.45 to 1.56)	CT/CBT individual	481	-0.96 (-2.03 to 0.14)
Behavioural therapies group	340	-0.86 (-2.51 to 0.82)	Mindfulness or meditation individual	20	-1.03 (-3.04 to 1.01)
Acupuncture + counselling individual	40	-0.93 (-3.35 to 1.45)	Behavioural therapies group	340	-0.92 (-2.16 to 0.36)
Short-term PDPT individual	49	-0.91 (-3.48 to 1.63)	Short-term PDPT individual	49	-0.99 (-3.08 to 1.14)
CT/CBT individual	450	-0.79 (-2.17 to 0.64)	Acupuncture + counselling individual	40	-0.94 (-2.84 to 0.95)
Mindfulness or meditation group	376	-0.78 (-2.11 to 0.42)	Mindfulness or meditation group	376	-0.85 (-2.20 to 0.36)
Acupuncture	40	-0.87 (-3.32 to 1.57)	Acupuncture	40	-0.87 (-2.77 to 1.03)
Relaxation group	63	-0.63 (-2.59 to 1.21)	IPT individual	153	-0.71 (-2.15 to 0.64)
Exercise group	185	-0.56 (-1.38 to 0.26)	Exercise group	199	-0.65 (-3.86 to 2.58)
IPT individual	136	-0.53 (-2.82 to 1.82)	Relaxation group	63	-0.66 (-2.63 to 1.15)
Exercise individual	250	-0·40 (-1·06 to 0·24)	Counselling individual	55	-0·47 (-2·87 to 1·91)
Counselling individual	55	-0·39 (-3·16 to 2·42)	Exercise individual	250	-0·48 (-2·16 to 1·18)
CT/CBT individual + exercise group	18	-0·24 (-2·77 to 2·30)	CT/CBT individual + exercise group	18	-0·39 (-2·40 to 1·67)
Self-help without/with minimal support	4,922	-0·30 (-0·79 to 0·19)	Self-help with support	1,286	-0·36 (-0·90 to 0·17)
Psychoeducation group	22	-0·21 (-2·72 to 2·29)	Psychoeducation group	22	-0·27 (-2·26 to 1·77)
Self-help with support	1,286	-0·28 (-0·82 to 0·26)	Self-help without/with minimal support	4,922	-0·36 (-0·84 to 0·11)
Problem solving individual	98	-0·06 (-2·36 to 2·28)	Problem solving individual	98	-0·10 (-1·83 to 1·68)

Treatment classes ordered from best to worst, according to mean ranking in each analysis. Negative effect values indicate a favourable outcome for treatment classes compared with TAU. Results where 95% CrI do not cross the no effect line are shown in bold.

CBT: cognitive behavioural therapy; CrI: credible intervals; CT: cognitive therapy; IPT: interpersonal psychotherapy; PDPT: psychodynamic psychotherapy; SMD: standardised mean difference; TAU: treatment as usual

Response in those randomised

Forest plots of response in those randomised in adults with a new episode of less severe depression: effects of treatment classes versus treatment as usual (TAU, N=623) Values on the right side of the vertical axis indicate better effect compared with TAU. Results are expressed as log-odds ratios (LORs). Effects are shown only for treatment classes with N ≥ 50.

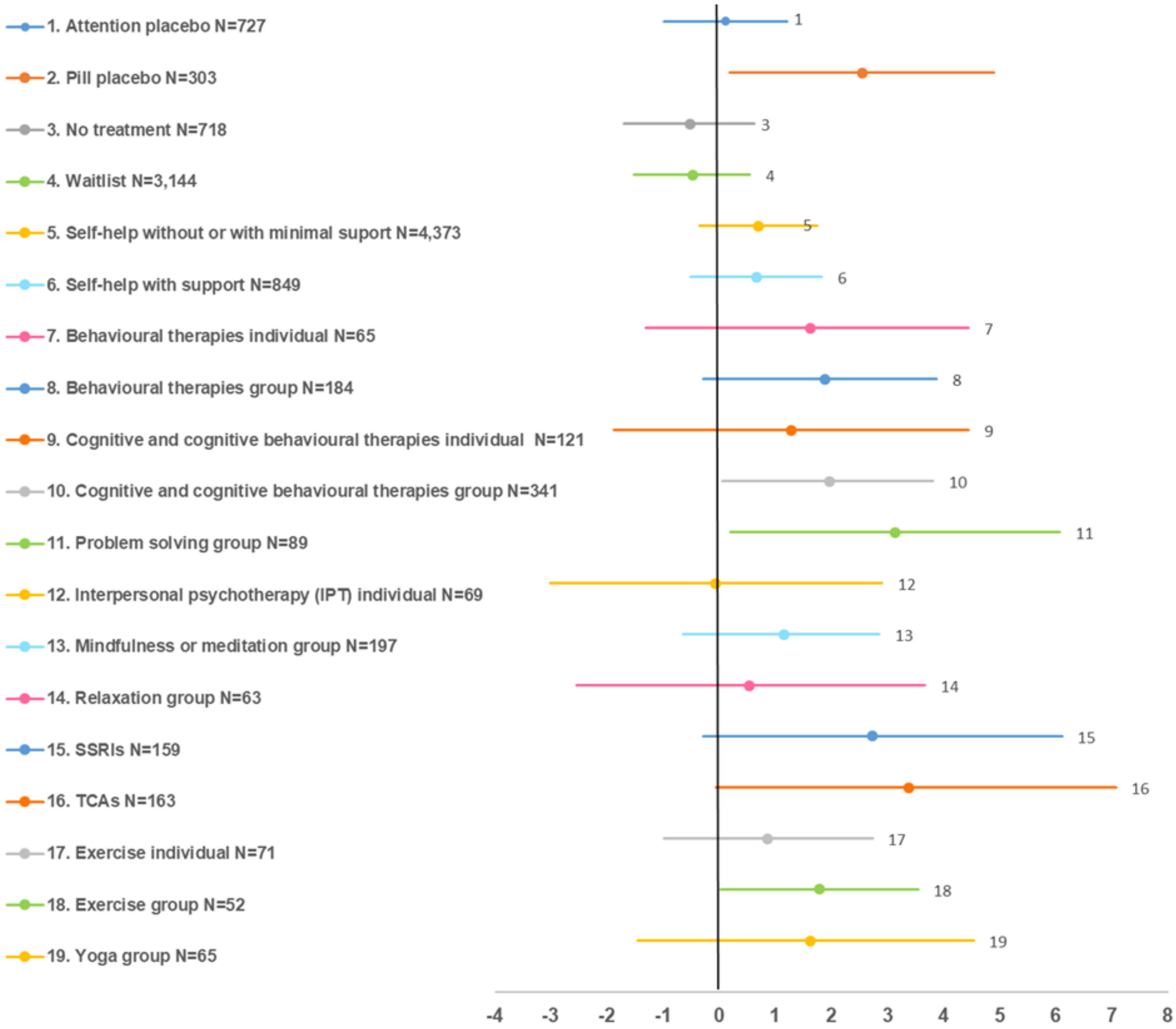

SSRIs: selective serotonin uptake inhibitors; TCAs: tricyclic antidepressants

Results of the network meta-analysis of response in those randomised in adults with a new episode of less severe depression: posterior effects (mean log-odds ratio [LOR], 95%CrI) of all treatment classes versus treatment as usual (TAU) and treatment class rankings

Treatment class	N	LOR vs TAU (mean, 95%CrI)	Rank (mean, 95% CrI)
TCAs	163	3·37 (-0·05 to 7·07)	4·54 (1 to 20)
Problem solving group	89	**3·14 (0·21 to 6·07)**	4·86 (1 to 18)
SSRIs	159	2·74 (-0·27 to 6·11)	6·27 (1 to 21)
Pill placebo	303	**2·55 (0·19 to 4·90)**	6·75 (2 to 19)
CT/CBT group	341	**1·96 (0·06 to 3·81)**	8·32 (2 to 18)
Behavioural therapies group	184	1·88 (-0·29 to 3·88)	8·86 (2 to 20)
Exercise group	52	**1·79 (0·02 to 3·54)**	9·27 (2 to 20)
Acupuncture + counselling individual	40	1·70 (-1·26 to 4·69)	10·30 (1 to 24)
Behavioural therapies individual	65	1·63 (-1·30 to 4·44)	10·40 (1 to 23)
Yoga group	65	1·63 (-1·45 to 4·54)	10·51 (1 to 24)
Acupuncture	40	1·59 (-1·39 to 4·60)	10·81 (1 to 24)
Mindfulness or meditation individual	20	1·56 (-1·75 to 4·74)	11·06 (1 to 24)
CT/CBT individual	121	1·29 (-1·87 to 4·44)	12·16 (1 to 24)
Mindfulness or meditation group	197	1·15 (-0·64 to 2·85)	12·76 (4 to 22)
Exercise individual	71	0·87 (-0·97 to 2·73)	14·24 (5 to 23)
Self-help without/with minimal support	4,373	0·71 (-0·35 to 1·75)	15·23 (10 to 19)
Psychoeducation group	22	0·61 (-2·71 to 3·81)	15·36 (2 to 25)
Self-help with support	849	0·66 (-0·52 to 1·83)	15·62 (10 to 21)
Relaxation group	63	0·55 (-2·54 to 3·67)	15·91 (2 to 25)
IPT individual	69	-0·06 (-3·01 to 2·90)	18·48 (4 to 25)
Attention placebo	727	0·13 (-0·98 to 1·21)	19·07 (14 to 23)
TAU	623	Reference	19·61 (14 to 24)
Enhanced TAU	36	-0·49 (-2·56 to 1·59)	20·98 (11 to 25)
Relaxation individual	15	-2·30 (-9·68 to 3·16)	21·53 (4 to 25)
Waitlist	3,144	-0·47 (-1·51 to 0·55)	22·09 (18 to 25)

Treatment classes ordered from best to worst, according to mean ranking. Positive effect values indicate a favourable outcome for treatment classes compared with TAU. Results where 95% CrI do not cross the no effect line are shown in bold.
CBT: cognitive behavioural therapy; CrI: credible intervals; CT: cognitive therapy; IPT: interpersonal psychotherapy; LOR: log-odds ratio; SSRIs: selective serotonin uptake inhibitors; TAU: treatment as usual; TCAs: tricyclic antidepressants

Remission in those randomised

Forest plots of remission in those randomised in adults with a new episode of less severe depression: effects of treatment classes versus treatment as usual (TAU, N=437) Values on the right side of the vertical axis indicate better effect compared with TAU. Only classes with N ≥ 50 are shown.

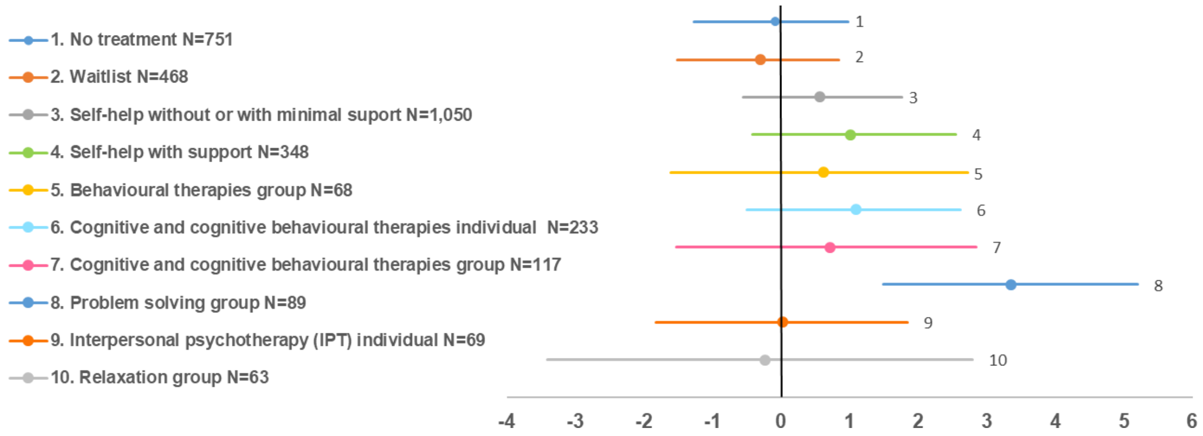

Results of the network meta-analysis of remission in those randomised in adults with a new episode of less severe depression: posterior effects (mean log-odds ratio [LOR], 95%CrI) of all treatment classes versus treatment as usual (TAU) and treatment class rankings

Treatment class	N	LOR vs TAU (mean, 95%CrI)	Rank (mean, 95% CrI)
Problem solving group	89	**3·36 (1·50 to 5·20)**	1·59 (1 to 5)
Yoga group	20	2·02 (-2·04 to 6·54)	4·58 (1 to 14)
CT/CBT individual	233	1·09 (-0·49 to 2·62)	5·38 (2 to 11)
Behavioural therapies individual	16	1·25 (-1·35 to 3·95)	5·45 (1 to 13)
Self-help with support	348	1·01 (-0·42 to 2·55)	5·72 (2 to 10)
Mindfulness or meditation individual	20	0·91 (-1·65 to 3·53)	6·57 (2 to 14)
CT/CBT group	117	0·72 (-1·53 to 2·85)	7·02 (2 to 13)
Behavioural therapies group	68	0·62 (-1·60 to 2·73)	7·49 (2 to 14)
Self-help without/with minimal support	1,050	0·56 (-0·55 to 1·77)	7·74 (4 to 11)
IPT individual	69	0·02 (-1·82 to 1·84)	9·81 (3 to 15)
TAU	437	Reference	10·27 (5 to 14)
Relaxation group	63	-0·23 (-3·41 to 2·79)	10·48 (2 to 15)
Waitlist	468	-0·3 (-1·51 to 0·84)	11·60 (8 to 14)
Attention placebo	46	-1·14 (-4·11 to 1·59)	12·67 (5 to 15)
Relaxation individual	15	-3·08 (-10·48 to 1·51)	13·64 (5 to 15)

Treatment classes ordered from best to worst, according to mean ranking. Positive effect values indicate a favourable outcome for treatment classes compared with TAU. Results where 95% CrI do not cross the no effect line are shown in bold.
CBT: cognitive behavioural therapy; CrI: credible intervals; CT: cognitive therapy; IPT: interpersonal psychotherapy; LOR: log-odds ratio; TAU: treatment as usual

Discontinuation for any reason

Results of the network meta-analysis of discontinuation for any reason in adults with a new episode of less severe depression: posterior effects (median log-odds ratio [LOR], 95%CrI) of all treatment classes versus treatment as usual (TAU) and treatment class rankings

Treatment class	N	LOR vs TAU (median, 95%CrI)	Rank (mean, 95% CrI)
Psychoeducation group	23	-1·87 (-4·84 to 0·99)	6·10 (1 to 25)
Short-term PDPT individual	53	-1·31 (-3·90 to 1·29)	8·16 (1 to 27)
Waitlist	3785	**-0·77 (-1·46 to -0·05)**	9·89 (5 to 16)
CT/CBT individual	663	-0·83 (-2·44 to 0·67)	9·94 (3 to 23)
Counselling individual	125	-0·99 (-3·50 to 1·51)	10·01 (1 to 28)
CT/CBT individual + exercise group	21	-0·91 (-3·86 to 2·02)	11·13 (1 to 30)
Relaxation group	63	-1·09 (-5·72 to 3·17)	11·37 (1 to 32)
Behavioural therapies individual	153	-0·96 (-4·30 to 2·34)	11·41 (1 to 31)
Yoga group	78	-0·76 (-4·76 to 3·25)	12·59 (1 to 32)
Acupuncture + counselling individual	40	-0·63 (-3·50 to 2·14)	13·17 (1 to 31)
Mindfulness or meditation group	375	-0·47 (-3·01 to 1·79)	13·49 (2 to 30)
Attention placebo	795	-0·25 (-1·02 to 0·54)	15·15 (9 to 23)
Acupuncture	40	-0·27 (-3·05 to 2·46)	15·74 (2 to 31)
Mindfulness or meditation individual	20	-0·29 (-3·83 to 3·37)	15·81 (1 to 32)
Exercise individual	235	-0·22 (-2·55 to 1·99)	15·84 (2 to 31)
CT/CBT group + AD	35	-0·08 (-4·27 to 4·56)	16·61 (1 to 32)
Mindfulness or meditation group + AD	44	-0·06 (-3·94 to 4·42)	16·78 (1 to 32)
TAU	1005	Reference	18·07 (10 to 26)
Exercise group	181	0·01 (-1·37 to 1·40)	18·21 (6 to 29)
CT/CBT group	483	0·07 (-1·96 to 1·87)	18·34 (4 to 31)
Self-help	5733	0·09 (-0·62 to 0·79)	19·28 (13 to 26)
SSRIs	462	0·41 (-2·71 to 5·10)	19·75 (2 to 32)
Self-help with support	1391	0·17 (-0·65 to 0·99)	20·17 (12 to 28)
Problem solving individual	159	0·32 (-1·98 to 2·63)	20·72 (4 to 31)
Pill placebo	621	0·59 (-2·61 to 5·44)	20·78 (2 to 32)
Behavioural therapies group	373	0·28 (-1·41 to 2·15)	20·82 (7 to 31)
CT/CBT group + exercise group	35	0·56 (-2·37 to 3·55)	21·57 (3 to 32)
IPT individual	135	0·45 (-1·31 to 3·27)	21·82 (6 to 32)
Relaxation individual	15	0·84 (-2·66 to 5·97)	21·87 (2 to 32)
TCAs	208	0·96 (-2·35 to 5·65)	23·18 (3 to 32)
Problem solving group	168	0·94 (-1·57 to 3·47)	24·70 (6 to 32)
Enhanced TAU	96	0·95 (-0·55 to 2·48)	25·54 (13 to 32)

Treatment classes ordered from best to worst, according to mean ranking. Negative effect values indicate a favourable outcome for treatment classes compared with TAU. Results where 95% CrI do not cross the no effect line are shown in bold.
AD: antidepressant; CBT: cognitive behavioural therapy; CrI: credible intervals; CT: cognitive therapy; IPT: interpersonal psychotherapy; LOR: log-odds ratio; PDPT: psychodynamic psychotherapy; SSRIs: selective serotonin uptake inhibitors; TAU: treatment as usual; TCAs: tricyclic antidepressants

Discontinuation due to side effects

Results of the indirect comparison of discontinuation due to side effects from medication in those discontinuing treatment for any reason, in adults with a new episode of less severe depression: posterior effects (mean log-odds ratio [LOR], 95%CrI) of all pharmacological treatment classes versus pill placebo and treatment class rankings

Treatment class	N	LOR vs pill placebo (mean, 95%CrI)	Rank (mean, 95% CrI)
Pill placebo	64	Reference	1·42 (1 to 2)
SSRIs	40	0·26 (-2·22 to 2·83)	1·61 (1 to 3)
TCAs	31	**3·61 (1·15 to 6·23)**	2·97 (2 to 3)

Treatment classes ordered from best to worst, according to mean ranking. Negative effect values indicate a favourable outcome for treatment classes compared with pill placebo. Results where 95% CrI do not cross the no effect line are shown in bold.

CrI: credible intervals; LOR: log-odds ratio; SSRIs: selective serotonin uptake inhibitors; TCAs: tricyclic antidepressants

Response in treatment completers

Results of the network meta-analysis of response in completers in adults with a new episode of less severe depression: posterior effects (median log-odds ratio [LOR], 95%CrI) of all treatment classes versus treatment as usual (TAU) and treatment class rankings

Treatment class	N	LOR vs TAU (median, 95%CrI)	Rank (mean, 95% CrI)
CT/CBT group + exercise group	25	**6·83 (2·88 to 11·25)**	1·54 (1 to 6)
CT/CBT group	164	**3·49 (1·55 to 5·60)**	5·04 (2 to 12)
TCAs	146	**3·37 (0·3 to 6·72)**	6·10 (1 to 19)
Yoga group	40	2·99 (-1·04 to 7·01)	8·08 (1 to 24)
Pill placebo	219	2·51 (-0·06 to 5·18)	9·17 (3 to 21)
Behavioural therapies group	171	2·25 (-0·22 to 4·92)	9·72 (2 to 21)
Problem solving group	15	2·24 (-1·68 to 6·11)	10·55 (2 to 25)
SSRIs	98	1·98 (-0·49 to 4·56)	11·27 (3 to 23)
CT/CBT group + AD	32	2·07 (-2·69 to 6·88)	11·57 (1 to 25)
Behavioural therapies individual	111	1·84 (-1·59 to 5·44)	11·91 (2 to 24)
Mindfulness or meditation individual	18	1·81 (-1·97 to 5·75)	12·17 (2 to 25)
CT/CBT individual	361	1·66 (-0·45 to 3·86)	12·37 (4 to 22)
Mindfulness or meditation group	179	1·64 (-0·59 to 3·96)	12·51 (4 to 22)
Short-term PDPT individual	43	1·63 (-2·29 to 5·69)	12·90 (2 to 25)
IPT individual	142	1·37 (-1·13 to 4·16)	13·93 (4 to 24)
Exercise group	178	1·31 (-0·71 to 3·23)	14·28 (6 to 23)
Counselling individual	39	1·19 (-3·38 to 5·80)	14·59 (2 to 25)
Exercise individual	189	1·11 (-0·69 to 2·96)	15·27 (7 to 23)
Self-help with support	327	0·98 (-1·01 to 2·90)	16·06 (7 to 24)
Self-help	1508	0·96 (-0·31 to 2·23)	16·20 (10 to 21)
CT/CBT individual + exercise group	18	0·77 (-2·98 to 4·64)	16·33 (3 to 25)
Problem solving individual	98	0·25 (-2·94 to 3·48)	18·73 (5 to 25)
Attention placebo	417	0·28 (-1·04 to 1·58)	20·06 (15 to 24)
TAU	395	Reference	21·09 (15 to 25)
Waitlist	772	-0·71 (-2·03 to 0·57)	23·59 (20 to 25)

Treatment classes ordered from best to worst, according to mean ranking. Positive effect values indicate a favourable outcome for treatment classes compared with TAU. Results where 95% CrI do not cross the no effect line are shown in bold.
CBT: cognitive behavioural therapy; CrI: credible intervals; CT: cognitive therapy; IPT: interpersonal psychotherapy; LOR: log-odds ratio; SSRIs: selective serotonin uptake inhibitors; TAU: treatment as usual; TCAs: tricyclic antidepressants

Remission in treatment completers

Results of the network meta-analysis of remission in completers in adults with a new episode of less severe depression: posterior effects (median log-odds ratio [LOR], 95%CrI) of all treatment classes versus treatment as usual (TAU) and treatment class rankings

Treatment class	N	LOR vs TAU (median, 95%CrI)	Rank (mean, 95% CrI)
Problem solving group	86	**3·53 (1·49 to 5·55)**	1·80 (1 to 6)
Exercise individual	29	2·37 (-0·06, 5·09)	3·49 (1 to 10)
Yoga group	15	2·13 (-1·93, 6·75)	5·18 (1 to 15)
Self-help with support	263	1·36 (-0·09, 2·92)	5·69 (2 to 11)
CT/CBT individual	194	1·24 (-0·46, 2·78)	6·17 (3 to 12)
Behavioural therapies individual	15	1·31 (-1·35 to 4·02)	6·41 (1 to 15)
Mindfulness or meditation individual	18	1·02 (-1·55 to 3·73)	7·26 (2 to 15)
Self-help	795	0·69 (-0·36 to 2·08)	8·28 (4 to 12)
Behavioural therapies group	61	0·66 (-1·73 to 2·83)	8·70 (3 to 15)
CT/CBT group	107	0·53 (-2·02 to 2·74)	8·94 (3 to 15)
IPT individual	58	0·00 (-2·00 to 2·02)	10·87 (3 to 16)
TAU	371	Reference	11·34 (7 to 15)
Relaxation group	61	-0·57 (-4·30 to 2·75)	12·48 (3 to 16)
Waitlist	414	-0·3 (-1·36 to 0·72)	12·72 (9 to 15)
Relaxation individual	12	-1·27 (-7·65 to 2·84)	13·03 (3 to 16)
Attention placebo	38	-0·98 (-3·80 to 1·80)	13·65 (6 to 16)

Treatment classes ordered from best to worst, according to mean ranking. Positive effect values indicate a favourable outcome for treatment classes compared with TAU. Results where 95% CrI do not cross the no effect line are shown in bold.
CBT: cognitive behavioural therapy; CrI: credible intervals; CT: cognitive therapy; IPT: interpersonal psychotherapy; LOR: log-odds ratio; TAU: treatment as usual

More severe depression

Standardised mean difference (SMD) of depression scale change scores

Bias-adjusted results

See Figure 4 in main article for the respective forest plots

Bias-adjusted results of the network meta-analysis of standardised mean difference (SMD) of depression symptom change scores in adults with a new episode of more severe depression: posterior effects (mean SMD, 95%CrI) of all treatment classes versus pill placebo and treatment class rankings

Treatment class	N	SMD vs pill placebo (mean, 95% CrI)	Rank (mean, 95% CrI)
Mindfulness or meditation group	15	**-3·40 (-4·77 to -2·03)**	1·41 (1 to 4)
Problem solving group	47	**-2·29 (-3·49 to -1·10)**	3·76 (1 to 12)
Yoga group + AD	15	-1·89 (-3·95 to 0·10)	7·82 (1 to 38)
Peer support group	39	**-1·35 (-2·42 to -0·26)**	9·83 (3 to 30)
Peer support group + AD	42	-1·47 (-3·30 to 0·25)	10·42 (2 to 39)
Exercise group + AD	79	-1·37 (-2·75 to 0·01)	10·63 (2 to 37)
CT/CBT individual + AD	192	**-1·18 (-2·07 to -0·44)**	11·09 (4 to 24)
CT/CBT group + AD	63	-1·23 (-2·95 to 0·41)	12·86 (2 to 40)
Psychoeducation group	44	-1·01 (-2·06 to 0·00)	14·18 (3 to 36)
Yoga group	65	-1·04 (-2·25 to 0·17)	14·26 (3 to 39)
Self-help without/with minimal support	344	-0·98 (-2·52 to 0·39)	14·99 (3 to 41)
Behavioural therapies individual	378	**-0·86 (-1·65 to -0·16)**	15·97 (5 to 33)
Exercise individual + AD	40	-0·96 (-2·25 to 0·27)	15·98 (3 to 40)
Light therapy + AD	54	**-0·86 (-1·59 to -0·12)**	16·07 (5 to 34)
Problem solving individual	367	-0·86 (-1·75 to 0·01)	16·22 (5 to 36)
Acupuncture + AD	584	**-0·78 (-1·12 to -0·44)**	16·88 (9 to 26)
CT/CBT individual	1,044	**-0·78 (-1·42 to -0·33)**	17·28 (8 to 27)
Counselling individual	404	-0·67 (-1·53 to 0·15)	19·96 (7 to 39)
Light therapy	32	-0·64 (-1·60 to 0·29)	20·89 (6 to 40)
Self-help with support	267	-0·60 (-1·61 to 0·54)	21·32 (6 to 41)
IPT individual + AD	99	-0·66 (-2·02 to 0·63)	21·32 (4 to 42)
Short-term PDPT individual	233	-0·58 (-1·35 to 0·10)	22·08 (8 to 38)
IPT individual	146	-0·45 (-1·36 to 0·47)	25·01 (8 to 41)
Acupuncture	264	-0·40 (-1·08 to 0·16)	26·35 (12 to 39)
Short-term PDPT individual + AD	131	-0·34 (-2·36 to 1·64)	26·51 (3 to 43)
Psychoeducation group + AD	27	-0·35 (-2·13 to 1·35)	26·59 (4 to 43)
Mirtazapine	1,884	**-0·35 (-0·48 to -0·22)**	27·04 (20 to 34)
Behavioural therapies individual + AD	22	-0·13 (-2·82 to 2·71)	28·06 (2 to 43)
SNRIs	9,538	**-0·32 (-0·43 to -0·22)**	28·07 (22 to 34)
Sham acupuncture	108	-0·31 (-1·07 to 0·41)	28·47 (12 to 41)
TAU	220	-0·30 (-0·67 to 0·06)	28·96 (19 to 38)
Relaxation individual + AD	10	0·05 (-2·82 to 2·96)	29·23 (2 to 43)
TCAs	4,524	**-0·29 (-0·50 to -0·05)**	29·34 (21 to 37)
Music therapy group	12	-0·14 (-1·69 to 1·41)	29·54 (5 to 43)
CT/CBT group	165	-0·26 (-1·12 to 0·60)	29·59 (11 to 42)
Exercise group	106	-0·19 (-1·20 to 0·87)	30·60 (10 to 42)
SSRIs	22,018	**-0·24 (-0·32 to -0·16)**	31·21 (25 to 37)
Exercise individual	298	-0·13 (-1·24 to 1·10)	31·75 (9 to 43)
Counselling individual + AD	57	0·21 (-2·52 to 2·96)	32·21 (4 to 43)

Attention placebo	61	-0·12 (-0·90 to 0·67)	32·27 (15 to 42)
Trazodone	1,072	-0·13 (-0·29 to 0·04)	34·14 (27 to 40)
Placebo	12,554	Reference	37·00 (32 to 41)
Waitlist	526	0·19 (-0·24 to 0·61)	38·83 (31 to 43)

Treatment classes ordered from best to worst, according to mean ranking. Negative effect values indicate a favourable outcome for treatment classes compared with pill placebo. Results where 95% CrI do not cross the no effect line are shown in bold.
AD: antidepressant; CBT: cognitive behavioural therapy; CrI: credible intervals; CT: cognitive therapy; IPT: interpersonal psychotherapy; PDPT: psychodynamic psychotherapy; SMD: standardised mean difference; SNRIs: serotonin and norepinephrine reuptake inhibitors; SSRIs: selective serotonin uptake inhibitors; TAU: treatment as usual; TCAs: tricyclic antidepressants

Base-case results

Base-case forest plots of standardised mean difference (SMD) of depression scale change scores in adults with a new episode of more severe depression: effects of treatment classes versus pill placebo (N=12,554)
Values on the left side of the vertical axis indicate better effect compared with pill placebo. Effects are shown only for treatment classes with N ≥ 50.

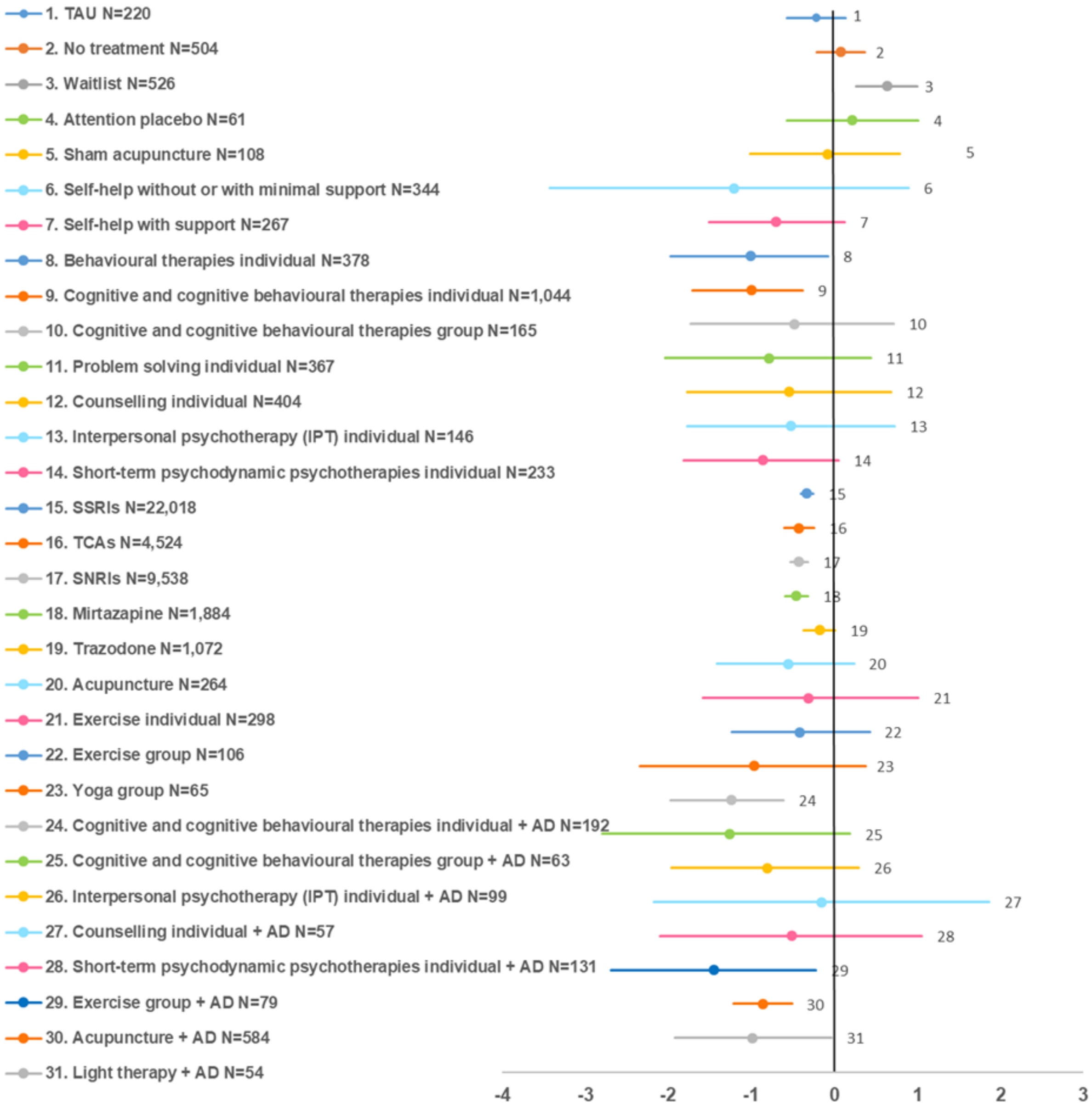

AD: antidepressant; SNRIs: serotonin and norepinephrine reuptake inhibitors; SSRIs: selective serotonin uptake inhibitors; TAU: treatment as usual; TCAs: tricyclic antidepressants

Base-case results of the network meta-analysis of standardised mean difference (SMD) of depression symptom change scores in adults with a new episode of more severe depression: posterior effects (mean SMD, 95%CrI) of all treatment classes versus pill placebo and treatment class rankings

Treatment class	N	SMD vs pill placebo (mean, 95% CrI)	Rank (mean, 95% CrI)
Mindfulness or meditation group	15	**-3·69 (-5·16 to -2·23)**	1·33 (1 to 4)
Problem solving group	47	**-2·37 (-3·76 to -1·00)**	4·05 (1 to 15)
Yoga group + AD	15	**-1·91 (-3·64 to -0·24)**	7·58 (1 to 33)
Exercise group + AD	79	**-1·46 (-2·69 to -0·22)**	10·64 (2 to 33)
Peer support group + AD	42	-1·49 (-3·10 to 0·04)	11·14 (2 to 38)
CT/CBT individual + AD	192	**-1·25 (-1·97 to -0·62)**	11·86 (4 to 23)
Peer support group	39	-1·37 (-2·75 to 0·03)	12·05 (2 to 37)
CT/CBT group + AD	63	-1·27 (-2·80 to 0·19)	13·65 (2 to 39)
Exercise individual + AD	40	**-1·13 (-2·21 to -0·09)**	14·73 (3 to 36)
Self-help without/with minimal support	344	-1·21 (-3·43 to 0·89)	15·21 (2 to 43)
CT/CBT individual	1,044	**-1·00 (-1·71 to -0·38)**	15·89 (6 to 29)
Behavioural therapies individual	378	**-1·01 (-1·98 to -0·08)**	16·21 (4 to 36)
Psychoeducation group	44	-1·05 (-2·41 to 0·31)	16·52 (3 to 40)
Light therapy + AD	54	**-0·99 (-1·92 to -0·04)**	16·59 (4 to 37)
Yoga group	65	-0·97 (-2·34 to 0·38)	17·77 (3 to 41)
Acupuncture + AD	584	**-0·87 (-1·22 to -0·51)**	17·88 (10 to 27)
Relaxation individual + AD	10	-0·96 (-2·68 to 0·78)	18·69 (2 to 42)
Short-term PDPT individual	233	-0·86 (-1·82 to 0·05)	18·99 (5 to 38)
IPT individual + AD	99	-0·81 (-1·96 to 0·29)	20·18 (5 to 40)
Behavioural therapies individual + AD	22	-0·85 (-2·51 to 0·83)	20·21 (3 to 42)
Problem solving individual	367	-0·79 (-2·04 to 0·44)	20·68 (4 to 41)
Light therapy	32	-0·77 (-2·06 to 0·52)	21·14 (4 to 41)
Self-help with support	267	-0·70 (-1·51 to 0·13)	21·74 (8 to 39)
Music therapy group	12	-0·56 (-2·10 to 0·97)	24·87 (4 to 43)
Acupuncture	264	-0·56 (-1·42 to 0·23)	25·13 (9 to 40)
Counselling individual	404	-0·55 (-1·78 to 0·68)	25·17 (6 to 42)
Short-term PDPT + AD	131	-0·51 (-2·10 to 1·06)	25·60 (4 to 43)
IPT individual	146	-0·52 (-1·77 to 0·72)	25·66 (6 to 42)
Psychoeducation group + AD	27	-0·47 (-2·05 to 1·04)	26·47 (5 to 43)
CT/CBT group	165	-0·48 (-1·73 to 0·71)	26·51 (6 to 42)
Mirtazapine	1,884	**-0·45 (-0·59 to -0·32)**	27·12 (21 to 34)
TCAs	4,524	**-0·43 (-0·60 to -0·24)**	27·80 (21 to 35)
Exercise group	106	-0·42 (-1·24 to 0·42)	27·84 (11 to 41)
SNRIs	9,538	**-0·43 (-0·54 to -0·32)**	27·95 (22 to 34)
Exercise individual	298	-0·32 (-1·59 to 1·01)	29·69 (7 to 43)
Counselling individual + AD	57	-0·16 (-2·18 to 1·87)	30·10 (4 to 43)
SSRIs	22,018	**-0·33 (-0·40 to -0·26)**	31·28 (26 to 36)
TAU	220	-0·22 (-0·57 to 0·13)	33·39 (24 to 40)
Sham acupuncture	108	-0·08 (-1·01 to 0·79)	34·18 (15 to 43)
Trazodone	1,072	-0·18 (-0·37 to 0·01)	34·47 (28 to 39)
Pill placebo	12,554	Reference	37·72 (33 to 41)
Attention placebo	61	0·21 (-0·57 to 1·01)	38·36 (25 to 43)
Waitlist	526	0·63 (0·26 to 1·00)	41·97 (39 to 43)

Treatment classes ordered from best to worst, according to mean ranking. Negative effect values indicate a favourable outcome for treatment classes compared with pill placebo. Results where 95% CrI do not cross the no effect line are shown in bold.
AD: antidepressant; CBT: cognitive behavioural therapy; CrI: credible intervals; CT: cognitive therapy; IPT: interpersonal psychotherapy; PDPT: psychodynamic psychotherapy; SMD: standardised mean difference; SNRIs: serotonin and norepinephrine reuptake inhibitors; SSRIs: selective serotonin uptake inhibitors; TAU: treatment as usual; TCAs: tricyclic antidepressants

Sensitivity analysis – inclusion of non-pharmacological RCTs

Forest plot comparing class-level results following exclusion of pharmacological trials from the NMA and results of the NMA base-case analysis: standardised mean difference (SMD) of depression scale change scores in adults with a new episode of more severe depression. Values on the left side of the vertical axis indicate better effect compared with TAU. TAU is used as the reference treatment, as the non-pharmacological dataset does not include pill placebo.

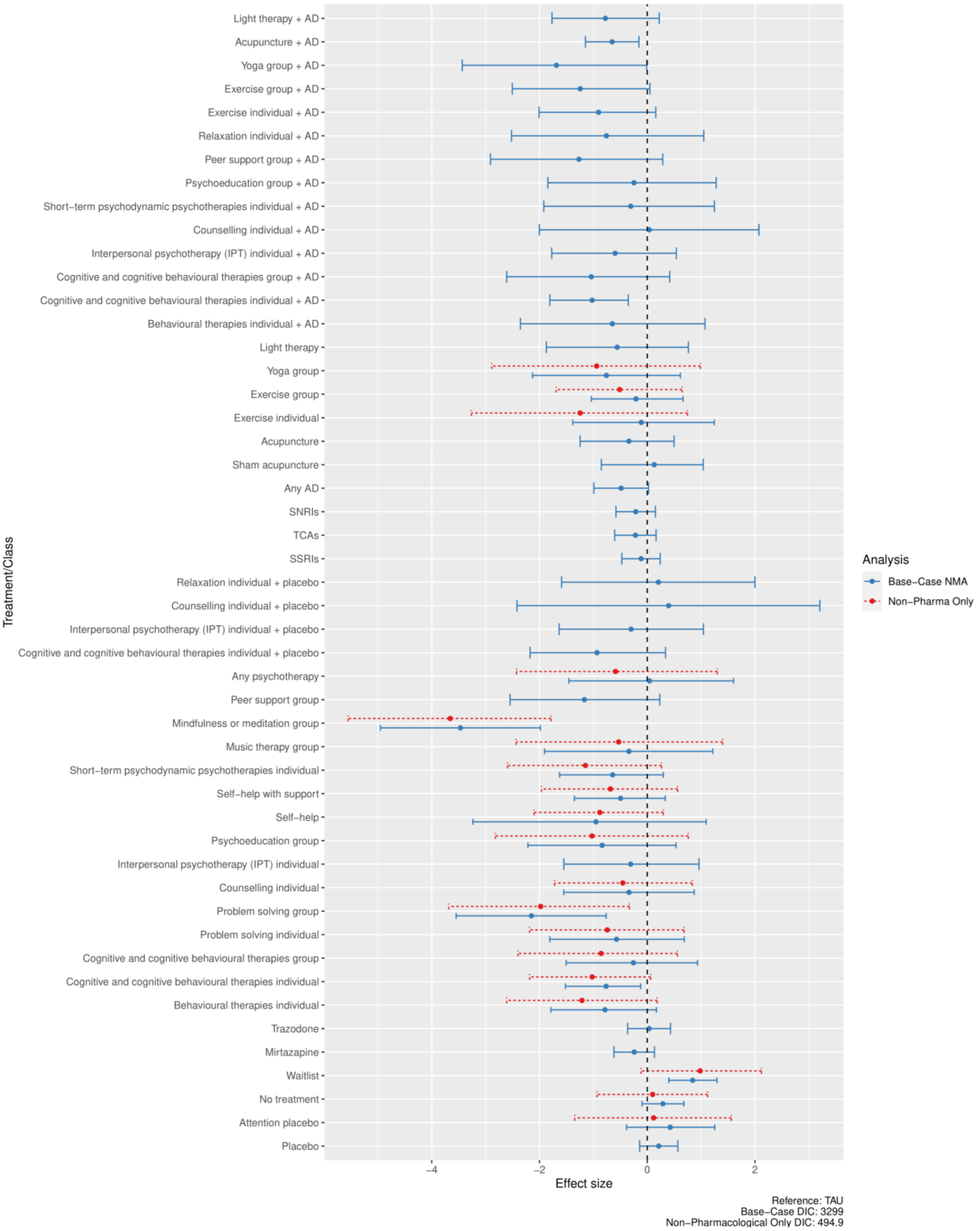

Comparison of results following exclusion of pharmacological trials from the NMA and results of the NMA base-case analysis: standardised mean difference (SMD) of depression scale change scores in adults with a new episode of more severe depression. TAU is used as the reference treatment, as the non-pharmacological dataset does not include pill placebo.

Non-pharmacological dataset			Full dataset – base-case analysis			
**Treatment class**	**N**	**Effect vs TAU (mean SMD, 95%CrI)**	**Treatment class**	**N**	**Effect vs pill placebo (mean SMD, 95%CrI)**	**Effect vs TAU (mean SMD, 95%CrI)**
Mindfulness or meditation group	15	**-3·66 (-5·55 to -1·79)**	Mindfulness or meditation group	15	**-3·69 (-5·16 to -2·23)**	**-3·47 (-4·95 to -1·99)**
Problem solving group	47	**-1·98 (-3·68 to -0·33)**	Problem solving group	47	**-2·37 (-3·76 to -1·00)**	**-2·15 (-3·55 to -0·76)**
Behavioural therapies individual	328	-1·21 (-2·61 to 0·19)	Self-help	344	-1·21 (-3·43 to 0·89)	-1·00 (-3·24 to 1·10)
Short-term PDPT individual	207	-1·15 (-2·60 to 0·27)	CT/CBT individual	1,044	**-1·00 (-1·71 to -0·38)**	**-0·78 (-1·52 to -0·12)**
Exercise individual	230	-1·25 (-3·27 to 0·75)	Behavioural therapies individual	378	**-1·01 (-1·98 to -0·08)**	-0·79 (-1·79 to 0·17)
CT/CBT individual	701	-1·03 (-2·18 to 0·06)	Psychoeducation group	44	-1·05 (-2·41 to 0·31)	-0·84 (-2·22 to 0·53)
Psychoeducation group	44	-1·03 (-2·82 to 0·76)	Yoga group	65	-0·97 (-2·34 to 0·38)	-0·76 (-2·13 to 0·62)
Yoga group	50	-0·94 (-2·89 to 0·99)	Short-term PDPT individual	233	-0·86 (-1·82 to 0·05)	-0·65 (-1·63 to 0·30)
Self-help without/with minimal support	344	-0·89 (-2·10 to 0·30)	Problem solving individual	367	-0·79 (-2·04 to 0·44)	-0·57 (-1·81 to 0·69)
CT/CBT group	42	-0·87 (-2·40 to 0·56)	Self-help with support	267	-0·70 (-1·51 to 0·13)	-0·50 (-1·35 to 0·33)
Problem solving individual	338	-0·74 (-2·18 to 0·68)	Music therapy group	12	-0·56 (-2·10 to 0·97)	-0·34 (-1·91 to 1·22)
Self-help with support	267	-0·69 (-1·96 to 0·57)	Counselling individual	404	-0·55 (-1·78 to 0·68)	-0·34 (-1·55 to 0·87)
Music therapy group	12	-0·53 (-2·44 to 1·40)	CT/CBT group	165	-0·48 (-1·73 to 0·71)	-0·27 (-1·51 to 0·93)
Exercise group	55	-0·52 (-1·69 to 0·65)	Exercise group	106	-0·42 (-1·24 to 0·42)	-0·21 (-1·04 to 0·66)
Counselling individual	404	-0·45 (-1·72 to 0·84)	Exercise individual	298	-0·32 (-1·59 to 1·01)	-0·10 (-1·38 to 1·24)

Treatment classes ordered from best to worst, according to mean ranking in each analysis. Negative effect values indicate a favourable outcome for treatment classes compared with pill placebo. Results where 95% CrI do not cross the no effect line are shown in bold.
CBT: cognitive behavioural therapy; CrI: credible intervals; CT: cognitive therapy; PDPT: psychodynamic psychotherapy; SMD: standardised mean difference; TAU: treatment as usual

Response in those randomised

Forest plots of response in those randomised in adults with a new episode of more severe depression: effects of treatment classes versus pill placebo (N=15,384) Values on the right side of the vertical axis indicate better effect compared with pill placebo. Results are expressed as log-odds ratios (LORs). Effects are shown only for treatment classes with N ≥ 50.

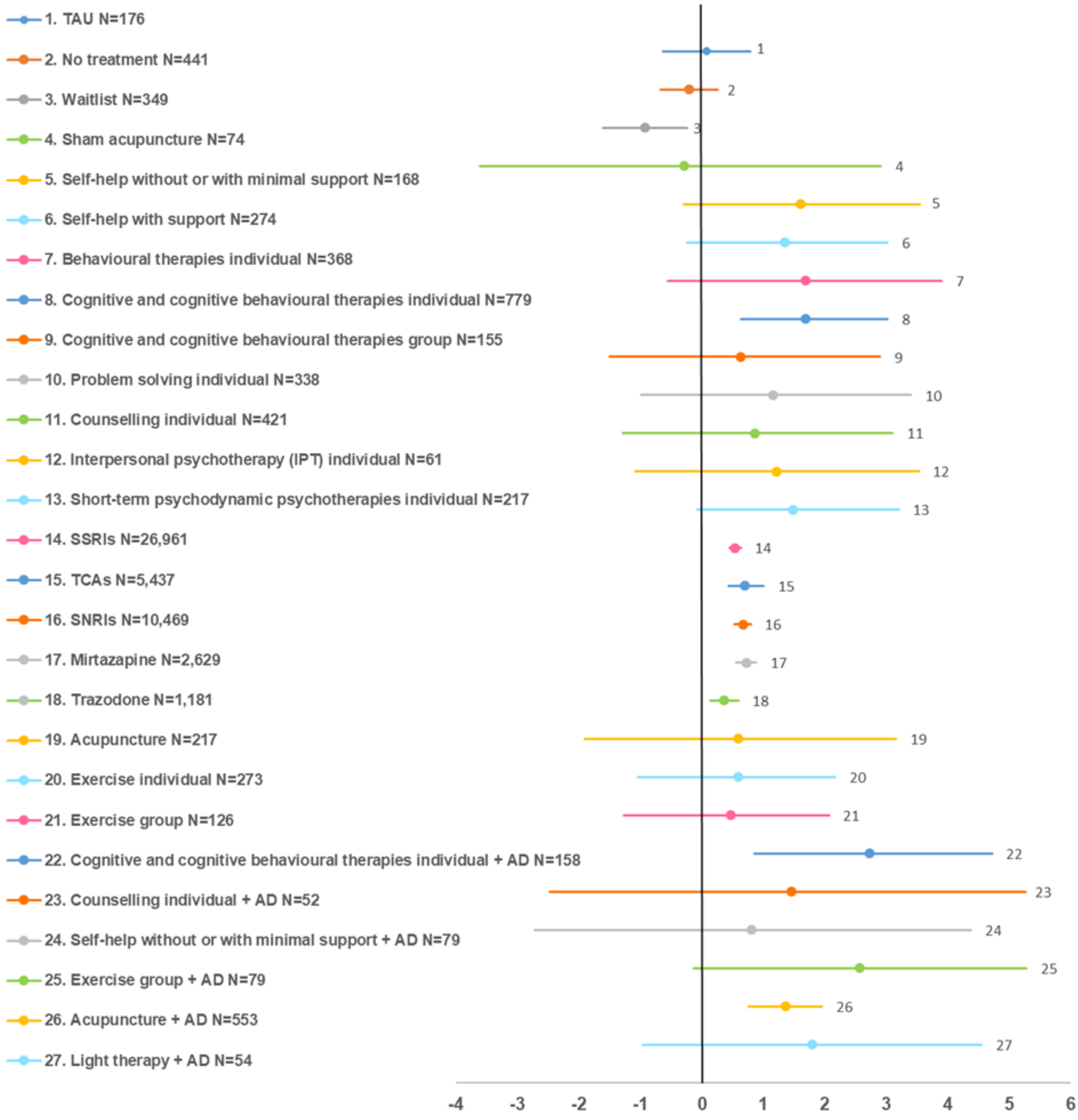

AD: antidepressant; SNRIs: serotonin and norepinephrine reuptake inhibitors; SSRIs: selective serotonin uptake inhibitors; TAU: treatment as usual; TCAs: tricyclic antidepressants

Results of the network meta-analysis of response in those randomised in adults with a new episode of more severe depression: posterior effects (mean log-odds ratio [LOR], 95%CrI) of all treatment classes versus pill placebo and treatment class rankings

Treatment class	N	LOR vs pill placebo (mean, 95% CrI)	Rank (mean, 95% CrI)
Mindfulness or meditation group	15	**6·61 (4·03 to 9·19)**	1·48 (1 to 4)
Yoga group + AD	15	3·68 (-0·07 to 7·63)	6·91 (1 to 32)
Exercise individual + AD	40	**2·86 (0·58 to 5·23)**	8·25 (2 to 25)
CT/CBT individual + AD	158	**2·73 (0·86 to 4·72)**	8·39 (2 to 21)
Peer support group	39	**2·71 (0·28 to 5·21)**	9·03 (2 to 29)
Peer support group + AD	42	2·91 (-0·66 to 6·66)	9·64 (1 to 35)
Exercise group + AD	79	2·56 (-0·14 to 5·28)	10·21 (2 to 33)
CT/CBT group + AD	20	2·78 (-0·83 to 6·55)	10·36 (2 to 36)
Behavioural therapies individual + AD	10	2·86 (-3·78 to 9·24)	12·55 (1 to 38)
CT/CBT individual	779	**1·69 (0·63 to 3·02)**	13·92 (6 to 24)
Light therapy + AD	54	1·79 (-0·97 to 4·55)	14·44 (3 to 36)
Behavioural therapies individual	368	1·68 (-0·55 to 3·89)	14·87 (4 to 35)
Self-help	168	1·61 (-0·30 to 3·55)	15·07 (4 to 34)
Short-term PDPT individual	217	1·48 (-0·09 to 3·20)	16·16 (5 to 32)
Acupuncture + AD	553	**1·36 (0·76 to 1·95)**	16·29 (10 to 23)
Self-help with support	274	1·34 (-0·25 to 3·01)	17·34 (6 to 33)
Counselling individual + AD	52	1·46 (-2·47 to 5·26)	17·97 (3 to 38)
IPT individual	61	1·21 (-1·09 to 3·53)	18·9 (5 to 36)
Problem solving individual	338	1·15 (-0·99 to 3·39)	19·43 (5 to 36)
Light therapy	32	1·05 (-2·78 to 4·92)	20·52 (2 to 38)
Music therapy group	12	0·92 (-1·70 to 3·59)	21·57 (5 to 38)
Counselling individual	421	0·86 (-1·29 to 3·10)	22·14 (6 to 37)
Self-help + AD	79	0·80 (-2·72 to 4·37)	22·42 (3 to 38)
Mirtazapine	2629	**0·72 (0·56 to 0·88)**	22·98 (18 to 28)
Yoga group	45	0·69 (-2·12 to 3·47)	23·32 (5 to 38)
TCAs	5437	**0·70 (0·43 to 1·00)**	23·45 (18 to 29)
SNRIs	10469	**0·66 (0·53 to 0·79)**	24·03 (19 to 29)
CT/CBT group	155	0·63 (-1·50 to 2·89)	24·44 (7 to 37)
Acupuncture	217	0·59 (-1·91 to 3·15)	24·51 (6 to 38)
Exercise individual	273	0·59 (-1·05 to 2·17)	24·77 (10 to 37)
Exercise group	126	0·47 (-1·27 to 2·06)	25·93 (11 to 37)
SSRIs	26961	**0·54 (0·45 to 0·63)**	26·53 (22 to 31)
Trazodone	1181	**0·36 (0·13 to 0·59)**	28·71 (24 to 33)
Sham acupuncture	74	-0·29 (-3·62 to 2·91)	30·33 (7 to 38)
TAU	176	0·08 (-0·64 to 0·79)	30·90 (23 to 36)
Pill placebo	15384	Reference	32·04 (28 to 36)
Attention placebo	36	-0·76 (-2·05 to 0·54)	35·03 (27 to 38)
Waitlist	349	**-0·93 (-1·61 to -0·25)**	36·17 (33 to 38)

Treatment classes ordered from best to worst, according to mean ranking. Positive effect values indicate a favourable outcome for treatment classes compared with pill placebo. Results where 95% CrI do not cross the no effect line are shown in bold.
AD: antidepressant; CBT: cognitive behavioural therapy; CrI: credible intervals; CT: cognitive therapy; IPT: interpersonal psychotherapy; LOR: log-odds ratio; PDPT: psychodynamic psychotherapy; SNRIs: serotonin and norepinephrine reuptake inhibitors; SSRIs: selective serotonin uptake inhibitors; TAU: treatment as usual; TCAs: tricyclic antidepressants

Remission in those randomised

Forest plots of remission in those randomised in adults with a new episode of more severe depression: effects of treatment classes versus pill placebo (N=8,376) Values on the right side of the vertical axis indicate better effect compared with pill placebo. Only classes with N ≥ 50 are shown.

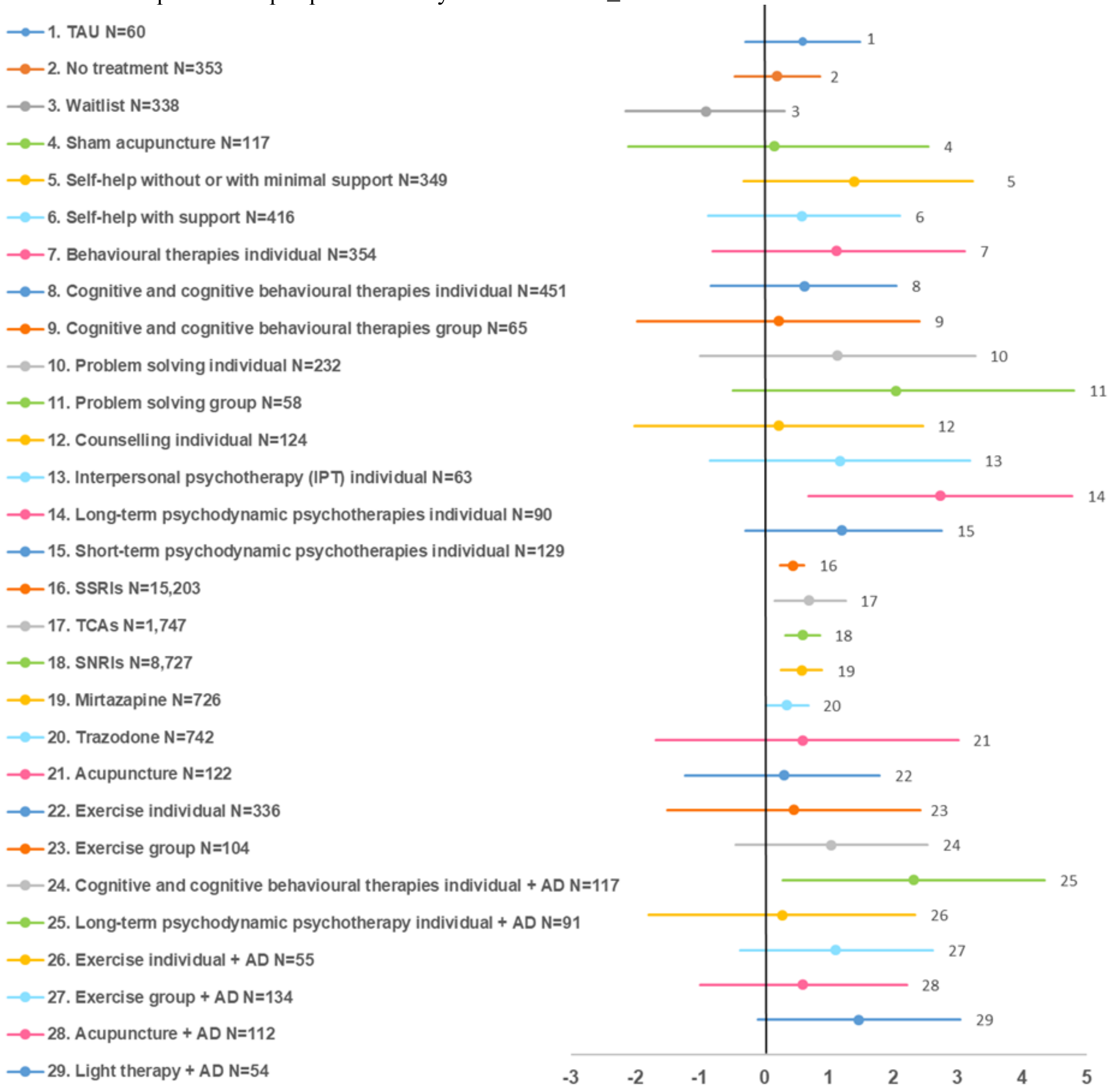

AD: antidepressant; SNRIs: serotonin and norepinephrine reuptake inhibitors; SSRIs: selective serotonin uptake inhibitors; TAU: treatment as usual; TCAs: tricyclic antidepressants

Results of the network meta-analysis of remission in those randomised in adults with a new episode of more severe depression: posterior effects (mean log-odds ratio [LOR], 95%CrI) of all treatment classes versus pill placebo and treatment class rankings

Treatment class	N	LOR vs pill placebo (mean, 95% CrI)	Rank (mean, 95% CrI)
Long-term PDPT individual	90	**2·73 (0·69 to 4·78)**	3·87 (1 to 17)
Long-term PDPT individual + AD	91	**2·32 (0·29 to 4·35)**	5·54 (1 to 24)
Problem solving group	58	2·05 (-0·49 to 4·81)	8·18 (1 to 31)
Light therapy + AD	54	1·47 (-0·10 to 3·04)	10·09 (2 to 28)
IPT individual + AD	16	1·54 (-0·72 to 3·84)	11·00 (1 to 32)
Self-help without/with minimal support	349	1·39 (-0·32 to 3·24)	11·28 (2 to 29)
Short-term PDPT individual	129	1·21 (-0·29 to 2·76)	12·50 (2 to 30)
Exercise group + AD	134	1·11 (-0·38 to 2·62)	13·42 (3 to 30)
IPT individual	63	1·17 (-0·84 to 3·19)	13·48 (2 to 32)
Behavioural therapies individual	354	1·12 (-0·80 to 3·11)	13·84 (2 to 32)
Problem solving individual	232	1·13 (-0·99 to 3·27)	13·96 (2 to 33)
CT/CBT individual + AD	117	1·04 (-0·44 to 2·53)	14·17 (3 to 31)
Light therapy	32	1·05 (-1·06 to 3·18)	14·77 (2 to 33)
Counselling individual + AD	13	0·88 (-1·53 to 3·29)	16·43 (1 to 34)
TCAs	1,747	**0·70 (0·16 to 1·26)**	17·28 (9 to 27)
Acupuncture	122	0·60 (-1·68 to 3·01)	18·64 (2 to 33)
SNRIs	8,727	**0·60 (0·33 to 0·86)**	18·76 (12 to 25)
CT/CBT individual	451	0·62 (-0·83 to 2·05)	18·84 (5 to 32)
TAU	60	0·60 (-0·29 to 1·49)	19·14 (8 to 31)
Mirtazapine	726	**0·58 (0·26 to 0·90)**	19·15 (12 to 26)
Acupuncture + AD	112	0·60 (-0·99 to 2·21)	19·19 (4 to 33)
Self-help with support	416	0·58 (-0·87 to 2·10)	19·56 (5 to 32)
Exercise group	104	0·46 (-1·50 to 2·42)	20·59 (4 to 34)
SSRIs	15,203	**0·44 (0·25 to 0·62)**	21·81 (16 to 27)
Exercise individual + AD	55	0·28 (-1·79 to 2·34)	22·13 (4 to 34)
CT/CBT group	65	0·23 (-1·97 to 2·41)	22·30 (4 to 34)
Counselling individual	124	0·22 (-2·01 to 2·46)	22·35 (4 to 34)
Yoga group	15	0·17 (-2·39 to 2·72)	22·36 (3 to 35)
Sham acupuncture	117	0·16 (-2·11 to 2·55)	22·55 (4 to 34)
Exercise individual	336	0·31 (-1·23 to 1·79)	22·69 (6 to 33)
CT/CBT group + AD	34	0·12 (-2·32 to 2·57)	22·90 (3 to 34)
Trazodone	742	**0·35 (0·03 to 0·68)**	23·11 (16 to 29)
Pill placebo	8376	Reference	27·78 (23 to 32)
Waitlist	338	-0·91 (-2·15 to 0·32)	32·01 (25 to 35)
Short-term PDPT group	24	**-3·22 (-7·00 to -0·06)**	34·32 (28 to 35)

Treatment classes ordered from best to worst, according to mean ranking. Positive effect values indicate a favourable outcome for treatment classes compared with pill placebo. Results where 95% CrI do not cross the no effect line are shown in bold.
AD: antidepressant; CBT: cognitive behavioural therapy; CrI: credible intervals; CT: cognitive therapy; IPT: interpersonal psychotherapy; LOR: log-odds ratio; PDPT: psychodynamic psychotherapy; SNRIs: serotonin and norepinephrine reuptake inhibitors; SSRIs: selective serotonin uptake inhibitors; TAU: treatment as usual; TCAs: tricyclic antidepressants

Discontinuation for any reason

Bias-adjusted results

Bias-adjusted results of the network meta-analysis of discontinuation for any reason in adults with a new episode of more severe depression: posterior effects (median log-odds ratio [LOR], 95%CrI) of all treatment classes versus pill placebo and treatment class rankings

Treatment class	N	LOR vs pill placebo (median, 95% CrI)	Rank (mean, 95% CrI)
Enhanced TAU	37	**-2·08 (-3·77 to -0·56)**	2·75 (1 to 12)
Waitlist	580	**-0·86 (-1·71 to -0·06)**	9·32 (3 to 20)
Attention placebo	36	-0·95 (-2·45 to 0·50)	10·25 (1 to 32)
Light therapy + AD	29	-1·06 (-3·51 to 1·03)	10·77 (1 to 35)
IPT individual + AD	16	-0·98 (-3·15 to 1·14)	11·15 (1 to 35)
Behavioural therapies individual	595	-0·75 (-1·75 to 0·25)	11·32 (2 to 29)
Problem solving individual	448	-0·76 (-1·92 to 0·33)	11·42 (2 to 30)
IPT individual	63	-0·72 (-1·83 to 0·36)	12·05 (2 to 31)
TAU	266	-0·67 (-1·49 to 0·13)	12·10 (3 to 27)
Self-help	477	-0·74 (-2·60 to 0·80)	12·24 (1 to 34)
Sham acupuncture	117	-0·74 (-1·97 to 0·52)	12·27 (2 to 32)
Long-term PDPT individual	90	-0·54 (-1·70 to 0·62)	14·77 (2 to 33)
CT/CBT individual	771	-0·41 (-1·10 to 0·29)	16·26 (6 to 30)
CT/CBT individual + AD	246	-0·40 (-1·51 to 0·51)	16·54 (3 to 33)
Counselling individual	332	-0·40 (-1·49 to 0·67)	17·05 (4 to 33)
Light therapy	32	-0·36 (-2·18 to 1·35)	17·88 (2 to 36)
Acupuncture	255	-0·33 (-1·33 to 0·68)	18·27 (5 to 34)
CT/CBT group	162	-0·25 (-1·59 to 1·00)	19·50 (3 to 35)
Yoga group	30	-0·23 (-2·00 to 1·54)	19·77 (2 to 36)
Exercise individual	336	-0·20 (-1·48 to 1·14)	20·10 (3 to 35)
Acupuncture + AD	160	-0·14 (-1·31 to 1·05)	21·13 (4 to 35)
Exercise group + AD	134	-0·09 (-1·54 to 1·35)	21·73 (3 to 36)
SSRIs	28,464	-0·07 (-0·19 to 0·04)	21·90 (15 to 28)
Behavioural therapies group	46	-0·09 (-1·52 to 1·34)	21·92 (4 to 36)
Exercise individual + AD	84	0·02 (-1·82 to 1·87)	23·09 (3 to 36)
Short-term PDPT individual	129	-0·03 (-1·07 to 1·04)	23·18 (6 to 35)
Mirtazapine	2,637	-0·02 (-0·21 to 0·17)	23·86 (16 to 31)
Pill placebo	16,577	Reference	24·52 (18 to 30)
Counselling individual + AD	13	0·48 (-2·83 to 3·76)	24·96 (1 to 36)
Long-term PDPT individual + AD	91	0·17 (-1·65 to 2·02)	25·13 (3 to 36)
SNRIs	10,251	0·02 (-0·14 to 0·19)	25·15 (18 to 31)
Self-help with support	556	0·10 (-1·05 to 1·63)	25·25 (7 to 36)
TCAs	7,782	0·05 (-0·15 to 0·22)	25·91 (18 to 32)
Exercise group	167	0·20 (-1·31 to 1·64)	25·98 (4 to 36)
Problem solving group	58	0·56 (-2·07 to 3·41)	26·59 (2 to 36)
Trazodone	1,430	0·25 (0·01 to 0·50)	29·92 (23 to 34)

Treatment classes ordered from best to worst, according to mean ranking. Negative effect values indicate a favourable outcome for treatment classes compared with pill placebo. Results where 95% CrI do not cross the no effect line are shown in bold.
AD: antidepressant; CBT: cognitive behavioural therapy; CrI: credible intervals; CT: cognitive therapy; IPT: interpersonal psychotherapy; LOR: log-odds ratio; PDPT: psychodynamic psychotherapy; SNRIs: serotonin and norepinephrine reuptake inhibitors; SSRIs: selective serotonin uptake inhibitors; TAU: treatment as usual; TCAs: tricyclic antidepressants

Base-case results

Base-case results of the network meta-analysis of discontinuation for any reason in adults with a new episode of more severe depression: posterior effects (median log-odds ratio [LOR], 95%CrI) of all treatment classes versus pill placebo and treatment class rankings

Treatment class	N	LOR vs pill placebo (median, 95% CrI)	Rank (mean, 95% CrI)
Enhanced TAU	37	**-2·07 (-3·59 to -0·70)**	2·53 (1 to 11)
Light therapy + AD	29	-1·19 (-3·45 to 0·67)	9·19 (1 to 34)
IPT individual + AD	16	-1·09 (-3·17 to 0·89)	9·96 (1 to 34)
Waitlist	580	**-0·75 (-1·49 to -0·04)**	10·97 (4 to 22)
IPT individual	63	-0·80 (-2·04 to 0·40)	11·37 (2 to 32)
Behavioural therapies individual	595	-0·77 (-1·91 to 0·35)	11·52 (2 to 31)
Problem solving individual	448	-0·76 (-2·00 to 0·40)	11·78 (2 to 31)
Attention placebo	36	-0·83 (-2·24 to 0·53)	11·79 (1 to 33)
TAU	266	-0·69 (-1·45 to 0·02)	11·96 (3 to 25)
Self-help	477	-0·75 (-2·48 to 0·63)	12·12 (1 to 33)
CT/CBT individual	771	-0·54 (-1·31 to 0·19)	14·51 (5 to 29)
Long-term PDPT individual	90	-0·59 (-1·88 to 0·73)	14·63 (2 to 34)
CT/CBT individual + AD	246	-0·50 (-1·56 to 0·34)	15·29 (3 to 32)
Counselling individual	332	-0·47 (-1·69 to 0·69)	16·43 (3 to 34)
Sham acupuncture	117	-0·45 (-1·58 to 0·74)	16·75 (3 to 34)
CT/CBT group	162	-0·43 (-1·84 to 0·84)	17·11 (2 to 34)
Light therapy	32	-0·42 (-2·13 to 1·16)	17·47 (2 to 35)
Exercise individual	336	-0·32 (-1·54 to 1·03)	18·87 (3 to 35)
Acupuncture + AD	160	-0·23 (-1·40 to 0·94)	20·23 (4 to 35)
Yoga group	30	-0·22 (-1·96 to 1·49)	20·30 (2 to 36)
Behavioural therapies group	46	-0·21 (-1·61 to 1·21)	20·80 (3 to 35)
Exercise group + AD	134	-0·16 (-1·53 to 1·24)	21·32 (3 to 35)
Acupuncture	255	-0·17 (-1·12 to 0·76)	21·58 (6 to 34)
SSRIs	28,464	-0·12 (-0·23 to -0·02)	21·81 (15 to 28)
Short-term PDPT individual	129	-0·13 (-1·2 to 0·91)	22·00 (5 to 35)
Exercise individual + AD	84	-0·06 (-1·87 to 1·75)	22·53 (2 to 36)
Mirtazapine	2,637	-0·07 (-0·25 to 0·11)	23·58 (16 to 31)
SNRIs	10,251	-0·03 (-0·19 to 0·14)	24·80 (17 to 31)
Long-term PDPT individual + AD	91	0·13 (-1·67 to 1·94)	25·11 (3 to 36)
Self-help with support	556	0·05 (-1·05 to 1·47)	25·23 (7 to 36)
Counselling individual + AD	13	0·40 (-2·48 to 3·28)	25·26 (1 to 36)
Exercise group	167	0·11 (-1·38 to 1·48)	25·33 (4 to 36)
TCAs	7,782	-0·01 (-0·20 to 0·15)	25·34 (18 to 31)
Placebo	16,577	Reference	25·97 (20 to 31)
Trazodone	1,430	0·23 (-0·01 to 0·46)	30·19 (24 to 35)
Problem solving group	58	0·79 (-1·20 to 3·19)	30·33 (6 to 36)

Treatment classes ordered from best to worst, according to mean ranking. Negative effect values indicate a favourable outcome for treatment classes compared with pill placebo. Results where 95% CrI do not cross the no effect line are shown in bold.
AD: antidepressant; CBT: cognitive behavioural therapy; CrI: credible intervals; CT: cognitive therapy; IPT: interpersonal psychotherapy; LOR: log-odds ratio; PDPT: psychodynamic psychotherapy; SNRIs: serotonin and norepinephrine reuptake inhibitors; SSRIs: selective serotonin uptake inhibitors; TAU: treatment as usual; TCAs: tricyclic antidepressants

Discontinuation due to side effects

Results of the network meta-analysis of discontinuation due to side effects from medication in those discontinuing treatment for any reason, in adults with a new episode of more severe depression: posterior effects (median log-odds ratio [LOR], 95%CrI) of all pharmacological and combined treatment classes versus pill placebo and treatment class rankings

Treatment class	N	LOR vs pill placebo (median, 95%CrI)	Rank (mean, 95% CrI)
Pill placebo	4,231	Reference	2.22 (1 to 4)
Light therapy	4	-0·08 (-3·97 to 2·98)	3·47 (1 to 10)
IPT individual + AD	10	0·40 (-3·06 to 2·81)	4·20 (1 to 10)
SSRIs	6,445	**0·85 (0·58 to 1·11)**	4·59 (2 to 7)
Mirtazapine	692	**0·88 (0·56 to 1·21)**	4·82 (2 to 7)
Light therapy + AD	2	1·26 (-2·69 to 5·43)	6·14 (1 to 10)
Trazodone	365	**1·11 (0·67 to 1·56)**	6·26 (3 to 9)
SNRIs	2,478	**1·25 (0·84 to 1·64)**	7·04 (4 to 9)
Acupuncture + AD	14	1·99 (-0·47 to 5·48)	7·89 (2 to 10)
TCAs	2,096	**1·54 (1·08 to 2·00)**	8·37 (6 to 10)

Treatment classes ordered from best to worst, according to mean ranking. Negative effect values indicate a favourable outcome for treatment classes compared with pill placebo. Results where 95% CrI do not cross the no effect line are shown in bold.
AD: antidepressant; CrI: credible intervals; IPT: interpersonal psychotherapy; LOR: log-odds ratio; SNRIs: serotonin and norepinephrine reuptake inhibitors; SSRIs: selective serotonin uptake inhibitors; TCAs: tricyclic antidepressants

Response in treatment completers

Bias-adjusted results

Bias-adjusted results of the network meta-analysis of response in completers in adults with a new episode of more severe depression: posterior effects (median log-odds ratio [LOR], 95%CrI) of all treatment classes versus pill placebo and treatment class rankings

Treatment class	N	LOR vs pill placebo (median, 95% CrI)	Rank (mean, 95% CrI)
Problem solving group	47	**3·74 (0·99 to 6·44)**	3·77 (1 to 17)
Acupuncture + AD	519	**2·55 (1·40 to 3·67)**	6·39 (2 to 15)
CT/CBT individual + AD	157	**2·34 (1·24 to 3·85)**	7·24 (2 to 15)
Exercise individual + AD	22	2·60 (-0·84 to 7·08)	9·33 (1 to 34)
Problem solving individual	123	2·01 (-0·50 to 4·52)	11·21 (1 to 33)
Light therapy + AD	52	1·79 (-0·06 to 3·65)	12·03 (2 to 31)
Yoga group	20	1·99 (-1·04 to 4·98)	12·14 (1 to 35)
Psychoeducation group	44	1·63 (-1·13 to 4·44)	14·22 (1 to 35)
Behavioural therapies individual	320	1·51 (-0·42 to 3·44)	14·31 (3 to 32)
CT/CBT group + AD	43	1·44 (-1·28 to 4·47)	15·33 (1 to 36)
Short-term PDPT individual	75	1·37 (-0·99 to 3·66)	15·87 (2 to 35)
Counselling individual	216	1·38 (-1·10 to 3·88)	15·92 (2 to 36)
Exercise group	55	1·13 (-1·20 to 3·81)	17·63 (2 to 36)
CT/CBT individual	507	1·08 (-0·36 to 2·59)	17·70 (6 to 32)
Exercise individual	88	1·18 (-2·42 to 4·88)	17·95 (1 to 38)
TAU	64	1·05 (-0·09 to 2·20)	17·97 (8 to 31)
TCAs	4,233	**0·87 (0·57 to 1·12)**	19·26 (13 to 26)
Light therapy	28	0·91 (-1·59 to 3·49)	19·71 (3 to 37)
SNRIs	6,569	**0·84 (0·55 to 1·11)**	19·78 (13 to 27)
Relaxation individual + AD	10	0·90 (-2·88 to 4·62)	19·86 (1 to 38)
Self-help	231	0·86 (-2·69 to 4·54)	20·13 (2 to 37)
IPT individual + AD	87	0·79 (-1·79 to 3·64)	20·63 (3 to 37)
Mirtazapine	1,845	**0·78 (0·44 to 1·10)**	20·78 (13 to 28)
Behavioural therapies individual + AD	22	0·53 (-2·50 to 3·69)	22·46 (3 to 38)
CT/CBT group	64	0·53 (-1·89 to 2·96)	23·00 (4 to 37)
SSRIs	16,720	**0·65 (0·43 to 0·84)**	23·12 (16 to 29)
Attention placebo	25	0·24 (-6·35 to 7·57)	23·34 (1 to 38)
Acupuncture	249	0·50 (-1·07 to 2·15)	23·65 (8 to 36)
IPT individual	132	0·47 (-1·88 to 2·77)	23·68 (5 to 37)
Music therapy group	12	0·30 (-2·88 to 3·49)	24·15 (3 to 38)
Trazodone	1,003	**0·51 (0·1 to 0·91)**	24·82 (17 to 32)
Short-term PDPT individual + AD	168	0·15 (-2·78 to 3·22)	25·33 (4 to 38)
Psychoeducation group + AD	27	0·15 (-2·57 to 3·19)	25·61 (4 to 38)
Self-help with support	189	0·00 (-2·48 to 2·17)	28·02 (9 to 38)
Counselling individual + AD	71	-0·48 (-3·56 to 2·80)	29·34 (6 to 38)
Pill placebo	9,333	Reference	29·89 (24 to 35)
Sham acupuncture	188	-0·27 (-1·94 to 1·51)	30·09 (13 to 38)
Waitlist	371	-1·10 (-2·20 to 0·01)	35·35 (30 to 38)

Treatment classes ordered from best to worst, according to mean ranking. Positive effect values indicate a favourable outcome for treatment classes compared with pill placebo. Results where 95% CrI do not cross the no effect line are shown in bold.
AD: antidepressant; CBT: cognitive behavioural therapy; CrI: credible intervals; CT: cognitive therapy; IPT: interpersonal psychotherapy; LOR: log-odds ratio; PDPT: psychodynamic psychotherapy; SNRIs: serotonin and norepinephrine reuptake inhibitors; SSRIs: selective serotonin uptake inhibitors; TAU: treatment as usual; TCAs: tricyclic antidepressants

Base-case results

Base-case results of the network meta-analysis of response in completers in adults with a new episode of more severe depression: posterior effects (median log-odds ratio [LOR], 95%CrI) of all treatment classes versus pill placebo and treatment class rankings

Treatment class	N	LOR vs pill placebo (median, 95% CrI)	Rank (mean, 95% CrI)
Problem solving group	47	**4·01 (1·21 to 6·77)**	3·87 (1 to 18)
Acupuncture + AD	519	**2·73 (1·53 to 3·91)**	7·01 (2 to 16)
CT/CBT individual + AD	157	**2·63 (1·47 to 4·16)**	7·34 (2 to 16)
Exercise individual + AD	22	2·74 (-0·50 to 6·85)	9·80 (1 to 34)
Problem solving individual	123	2·15 (-0·42 to 4·73)	12·21 (1 to 34)
Light therapy + AD	52	**2·05 (0·14 to 3·98)**	12·23 (2 to 31)
Yoga group	20	2·09 (-0·98 to 5·11)	13·31 (1 to 35)
Exercise individual	88	2·13 (-1·28 to 5·49)	13·33 (1 to 36)
Behavioural therapies individual	320	1·75 (-0·21 to 3·74)	14·57 (3 to 33)
Relaxation individual + AD	10	1·82 (-1·46 to 5·15)	15·16 (1 to 37)
Psychoeducation group	44	1·77 (-1·13 to 4·68)	15·27 (1 to 36)
CT/CBT group + AD	43	1·72 (-1·23 to 4·97)	15·49 (1 to 36)
Short-term PDPT individual	75	1·64 (-0·72 to 3·94)	15·84 (2 to 35)
Exercise group	55	1·49 (-0·92 to 4·18)	16·89 (2 to 36)
Counselling individual	216	1·51 (-1·10 to 4·12)	17·12 (2 to 36)
CT/CBT individual	507	1·36 (-0·11 to 2·90)	17·62 (6 to 32)
Behavioural therapies individual + AD	22	1·44 (-1·33 to 4·20)	17·66 (2 to 36)
TCAs	4,233	**1·13 (0·87 to 1·36)**	19·13 (13 to 26)
Light therapy	28	1·17 (-1·46 to 3·81)	19·83 (3 to 37)
IPT individual + AD	87	1·14 (-1·65 to 4·15)	19·88 (2 to 37)
Mirtazapine	1,845	**1·01 (0·70 to 1·33)**	20·94 (14 to 28)
Self-help	231	1·00 (-2·54 to 4·53)	21·11 (2 to 37)
CT/CBT group	64	1·00 (-1·51 to 3·50)	21·20 (3 to 37)
SNRIs	6,569	**0·99 (0·70 to 1·27)**	21·31 (14 to 28)
Music therapy group	12	0·86 (-2·31 to 4·05)	22·03 (2 to 38)
TAU	64	0·92 (-0·22 to 2·04)	22·32 (11 to 33)
IPT individual	132	0·71 (-1·72 to 3·13)	23·65 (5 to 37)
Short-term PDPT individual + AD	168	0·61 (-2·39 to 3·69)	23·89 (3 to 38)
Acupuncture	249	0·68 (-0·95 to 2·39)	24·26 (8 to 36)
SSRIs	16,720	**0·81 (0·61 to 0·99)**	24·27 (18 to 30)
Psychoeducation group + AD	27	0·50 (-2·42 to 3·69)	24·71 (3 to 38)
Trazodone	1,003	**0·68 (0·28 to 1·08)**	25·70 (18 to 32)
Attention placebo	25	-0·17 (-7·27 to 6·84)	26·92 (1 to 38)
Self-help with support	189	0·32 (-2·21 to 2·47)	27·34 (8 to 38)
Counselling individual + AD	71	0·06 (-3·07 to 3·27)	27·61 (5 to 38)
Placebo	9,333	Reference	31·60 (26 to 36)
Sham acupuncture	188	-0·34 (-2·07 to 1·51)	31·92 (15 to 38)
Waitlist	371	**-1·42 (-2·47 to -0·37)**	36·67 (33 to 38)

Treatment classes ordered from best to worst, according to mean ranking. Positive effect values indicate a favourable outcome for treatment classes compared with pill placebo. Results where 95% CrI do not cross the no effect line are shown in bold.
AD: antidepressant; CBT: cognitive behavioural therapy; CrI: credible intervals; CT: cognitive therapy; IPT: interpersonal psychotherapy; LOR: log-odds ratio; PDPT: psychodynamic psychotherapy; SNRIs: serotonin and norepinephrine reuptake inhibitors; SSRIs: selective serotonin uptake inhibitors; TAU: treatment as usual; TCAs: tricyclic antidepressants

Remission in treatment completers

Results of the network meta-analysis of remission in those randomised in adults with a new episode of more severe depression: posterior effects (median log-odds ratio [LOR], 95%CrI) of all treatment classes versus pill placebo and treatment class rankings

Treatment class	N	LOR vs pill placebo (median, 95% CrI)	Rank (mean, 95% CrI)
Long-term PDPT individual + AD	62	**4·88 (2·81 to 7·44)**	1·65 (1 to 4)
Long-term PDPT individual	73	**3·77 (1·05 to 6·54)**	3·22 (1 to 13)
Problem solving group	47	**3·13 (0·05 to 6·55)**	4·94 (1 to 24)
CT/CBT individual + AD	100	**1·51 (0·50 to 2·64)**	9·36 (4 to 20)
Short-term PDPT individual	101	1·52 (-0·41 to 3·54)	10·62 (3 to 28)
Light therapy + AD	52	1·53 (-0·57 to 3·62)	10·62 (3 to 29)
Exercise group + AD	114	**1·35 (0·05 to 2·75)**	11·10 (4 to 25)
Self-help	327	1·28 (-0·58 to 3·66)	12·27 (3 to 28)
Counselling individual + AD	24	1·22 (-1·06 to 3·42)	13·42 (3 to 30)
TCAs	1,204	**0·97 (0·40 to 1·51)**	13·67 (8 to 22)
Problem solving individual	191	1·13 (-1·58 to 3·87)	13·98 (2 to 31)
Light therapy	28	1·10 (-1·76 to 4·05)	14·32 (2 to 31)
IPT individual	89	0·98 (-1·62, 3·63)	15·07 (3 to 31)
Self-help with support	323	0·88 (-0·79, 2·83)	15·62 (4 to 29)
SNRIs	5,949	**0·76 (0·56 to 0·96)**	16·06 (11 to 21)
Acupuncture + AD	100	0·69 (-1·44 to 2·78)	17·29 (4 to 31)
CT/CBT individual	440	0·69 (-1·23 to 2·54)	17·55 (4 to 30)
Acupuncture	145	0·61 (-1·59 to 2·84)	17·55 (4 to 30)
Behavioural therapies individual	330	0·72 (-1·45 to 2·72)	17·66 (4 to 31)
Exercise group	80	0·64 (-2·01 to 3·21)	17·88 (3 to 31)
Mirtazapine	645	**0·61 (0·33 to 0·89)**	18·43 (13 to 24)
Trazodone	552	**0·54 (0·26 to 0·82)**	19·57 (14 to 25)
SSRIs	10,361	**0·51 (0·36 to 0·65)**	20·21 (15 to 25)
Counselling individual	103	0·27 (-2·49 to 3·09)	20·22 (4 to 31)
CT/CBT group	32	0·26 (-2·49 to 3·05)	20·64 (4 to 31)
TAU	45	0·37 (-0·65 to 1·33)	21·06 (10 to 29)
Sham acupuncture	100	0·11 (-2·15 to 2·36)	21·71 (5 to 31)
Exercise individual + AD	44	0·19 (-1·67 to 2·02)	22·28 (6 to 31)
Exercise individual	242	0·15 (-1·46 to 1·79)	22·92 (7 to 31)
Placebo	5,850	Reference	25·54 (21 to 29)
Waitlist	309	-1·03 (-2·21 to 0·13)	29·54 (25 to 31)

Treatment classes ordered from best to worst, according to mean ranking. Positive effect values indicate a favourable outcome for treatment classes compared with pill placebo. Results where 95% CrI do not cross the no effect line are shown in bold.
AD: antidepressant; CBT: cognitive behavioural therapy; CrI: credible intervals; CT: cognitive therapy; IPT: interpersonal psychotherapy; LOR: log-odds ratio; PDPT: psychodynamic psychotherapy; SNRIs: serotonin and norepinephrine reuptake inhibitors; SSRIs: selective serotonin uptake inhibitors; TAU: treatment as usual; TCAs: tricyclic antidepressants
