# Supplementary material for: A systematic review and network meta-analysis of psychological, psychosocial, pharmacological, physical and combined treatments for adults with a new episode of depression
Source: eClinicalMedicine. 2024 Aug 16;75:102780. doi: 10.1016/j.eclinm.2024.102780 (PMC11377144; doi:10.1016/j.eclinm.2024.102780)

APPENDIX 9 – 2023 REVIEW UPDATE RESULTS

CONTENTS

Flow diagram of study selection 2

Included and excluded studies identified from the search update 3

Characteristics of included studies, and full references 3

List of excluded studies with reasons for exclusion, and full references 10

Updated SMD networks, treatment classes, interventions and numbers of participants tested 22

Less severe depression 22

Network - treatment class level 22

Network - intervention level 23

Classes, interventions and numbers of participants tested on each 24

More severe depression 26

Network - treatment class level 26

Network - intervention level 27

Classes, interventions and numbers of participants tested on each 28

Model fit statistics – original and updated bias-adjusted SMD analyses 31

Less severe depression 31

More severe depression 31

Risk of bias of new studies included in the NMA (reviewer's judgements) 32

Less severe depression 32

More severe depression 33

Updated bias-adjusted results: SMDs versus the reference treatment 34

Less severe depression 34

Treatment class level 34

Intervention level 35

More severe depression 38

Treatment class level 38

Intervention level 40

Comparison between original and updated results (SMD versus reference) 43

Less severe depression 43

Treatment class level 43

Intervention level 44

More severe depression 45

Treatment class level 45

Intervention level 46

Flow diagram of study selection

Figure 1: Flow diagram of study selection for the review update (only new records identified between June 2020 – November 2023 included)

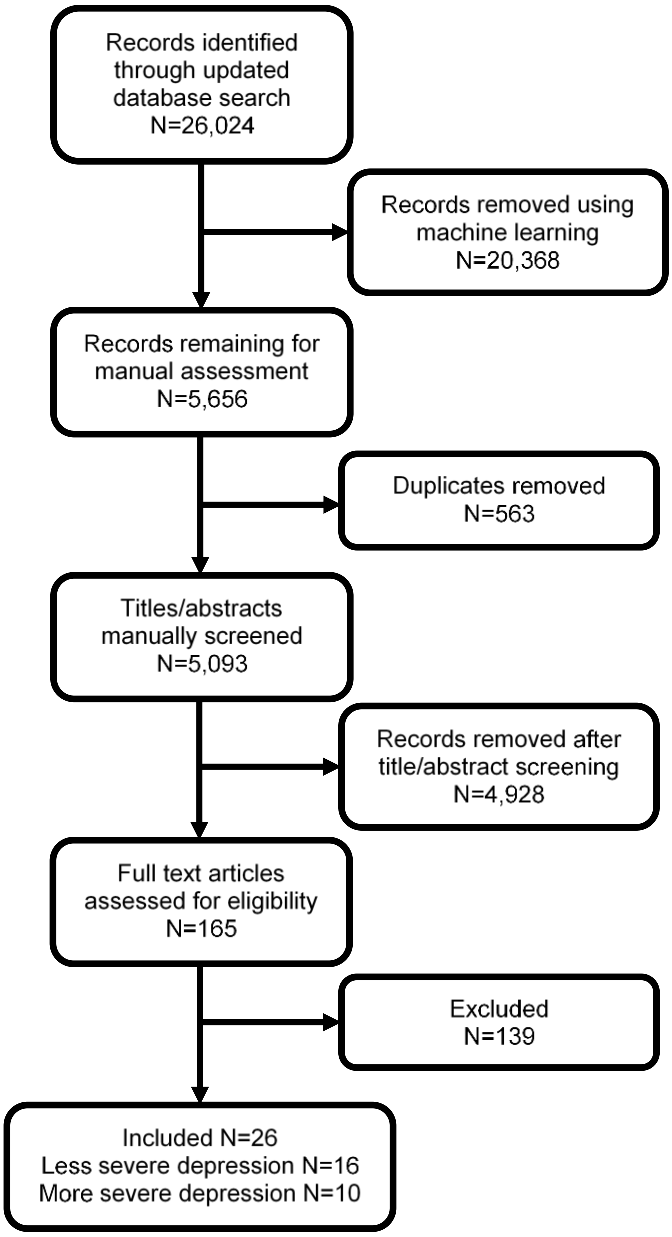

Included and excluded studies identified from the search update

Characteristics of included studies, and full references

Less severe depression										
	**Study ID**	**NMA node: interventions**	**Inclusion criteria**	**N**	**Country**	**Mean age in years (SD)**	**Sex (% female)**	**Baseline depression scale**	**Mean baseline depression score (SD)**	**Reference**
1	Armat 2022	1. Laughter yoga group<br>2. No treatment	Female retired educators; aged 50-70 years; BDI score=10-19; BAI score=8-26; not engaged in any regular physical exercises or sport programmes; receiving any antidepressants, anti-anxiety medication, or CBT	62	Iran	58.3 (5.0)	100	BDI	13.47 (4.67)	Armat, M. R., Emami Zeydi, A., Mokarami, H., Nakhlband, A., & Hojjat, S. K. (2022). The impact of laughter yoga on depression and anxiety among retired women: a randomized controlled clinical trial. Journal of women & aging, 34(1), 31-42.
2	Braun 2021	1. Computerised-CBT (CCBT) with support<br>2. No treatment	Insurance policy holder in a green profession; aged at least 18 years; PHQ-9 score≥5; no current psychotherapy	360	Germany	50.6 (10.2)	58	QIDS	10.01 (4.42)	Braun, L., Titzler, I., Terhorst, Y., Freund, J., Thielecke, J., Ebert, D. D., & Baumeister, H. (2021). Effectiveness of guided internet-based interventions in the indicated prevention of depression in green professions (PROD-A): results of a pragmatic randomized controlled trial. Journal of Affective Disorders, 278, 658-671.
3	Brush 2022	1. Supervised high intensity exercise individual<br>2. Supervised low intensity exercise individual	University students; current depression diagnosis; no regular exercise; no current treatment beyond stable (≥6 weeks) SSRI treatment	66	US	20.2 (2.4)	74	BDI-II	21.26 (7.57)	Brush, C. J., Hajcak, G., Bocchine, A. J., Ude, A. A., Muniz, K. M., Foti, D., & Alderman, B. L. (2022). A randomized trial of aerobic exercise for major depression: examining neural indicators of reward and cognitive control as predictors and treatment targets. Psychological medicine, 52(5), 893-903.

**Less severe depression**										
4	Cody 2023	1. Computerised exercise promotion with support<br>2. Attention placebo	Inpatients aged 18-65 years; met ICD-10 criteria for a diagnosis of MDD; BDI score ≥17; insufficiently physically active prior to inpatient treatment	244	Switzerlan d	40.9 (12.6)	52	HAMD	13.37 (5.31)	Cody, R., Beck, J., Brand, S., Donath, L., Faude, O., Hatzinger, M., ... & Gerber, M. (2023). Short-term outcomes of physical activity counseling in in-patients with Major Depressive Disorder: Results from the PACINPAT randomized controlled trial. Frontiers in psychiatry, 13, 1045158.
5	Demir 2022	1. CBT group (under 15 sessions)<br>2. No treatment	Nursing students; BDI score=10-20; no diagnosis of depressive disorder; no use of psychotropic medication; not receiving other counselling or therapy	80	Turkey	20.1 (1.5)	94	BDI	13.49 (3.22)	Demir, S., & Ercan, F. (2022). The effectiveness of cognitive behavioral therapy-based group counseling on depressive symptomatology, anxiety levels, automatic thoughts, and coping ways Turkish nursing students: A randomized controlled trial. Perspectives in Psychiatric Care, 58(4), 2394-2406.
6	He 2022	1. Computerised-CBT (CCBT)<br>2. Psychoeducational website<br>3. Attention placebo	University students aged 17-34 years; College Students Mental Health Screening Scale (CSMHSS) depression subscale score = 2-3; not taking a psychiatric medication	148	China	18.8 (0.9)	37	PHQ-9	10.02 (3.18)	He, Y., Yang, L., Zhu, X., Wu, B., Zhang, S., Qian, C., & Tian, T. (2022). Mental health chatbot for young adults with depressive symptoms during the COVID-19 pandemic: single-blind, three-arm randomized controlled trial. Journal of Medical Internet Research, 24(11), e40719.
7	Kannampal lil 2023	1. Computerised problem solving therapy<br>2. Waitlist	Outpatients aged at least 18 years; PHQ score=10-19 and/or GAD-7 score=10-14; not receiving current pharmacotherapy or psychotherapy (individual or professionally led group therapy) for depression or anxiety	63	US	37.8 (12.4)	68	HADS-D	7.3 (3)	Kannampallil, T., Ajilore, O. A., Lv, N., Smyth, J. M., Wittels, N. E., Ronneberg, C. R., ... & Ma, J. (2023). Effects of a virtual voice-based coach delivering problem-solving treatment on emotional distress and brain function: a pilot RCT in depression and anxiety. Translational psychiatry, 13(1), 166.
8	Kramer 2021	1. Computerised behavioural	Outpatients aged at least 18 years, on a	136	Germany	36.3 (11.9)	78	PHQ-9	15.16 (4.32)	Kramer, L. V., Gruenzig, S. D., Baumeister, H., Ebert, D. D., &

**Less severe depression**										
		activation with support<br>2. Waitlist	waiting list for psychotherapy (wait of at least 2 months); CES-D score>22							Bengel, J. (2021). Effectiveness of a guided web-based intervention to reduce depressive symptoms before outpatient psychotherapy: A pragmatic randomized controlled trial. Psychotherapy and Psychosomatics, 90(4), 233-242.
9	Lopez 2020b	1. CBT group (under 15 sessions)<br>2. Behavioural activation (BA) group<br>3. TAU	Informal caregiver; CES-D score >16; do not meet DSM-5 criteria for a current major depressive episode; no history of major depression; not received psychological or pharmacological treatment in the last 2 months	219	Spain	54.0 (10.8)	91	CES-D	22.7 (6.3)	Lopez, L., Vázquez, F. L., Torres, Á. J., Otero, P., Blanco, V., Díaz, O., & Páramo, M. (2020). Long-term effects of a cognitive behavioral conference call intervention on depression in non-professional caregivers. International Journal of Environmental Research and Public Health, 17(22), 8329.
10	MacLean 2020	1. Computerised problem solving therapy with support<br>2. Waitlist	Outpatients aged at least 16 years; symptoms of depression or dysthymia; on a waiting list for psychiatric treatment (waitlist 9 months to 1 year)	95	Canada	44.2 (12.9)	70	PHQ-9	15.15 (5.68)	MacLean, S., Corsi, D. J., Litchfield, S., Kucharski, J., Genise, K., Selaman, Z., ... & Hatcher, S. (2020). Coach-facilitated web-based therapy compared with information about web-based resources in patients referred to secondary mental health care for depression: randomized controlled trial. Journal of Medical Internet Research, 22(6), e15001.
11	Mathiasen 2022	1. Computerised-CBT (CCBT) with support<br>2. CBT individual (under 15 sessions)	Adults aged at least 18 years; met DSM-IV-TR criteria for MDD (assessed with MINI); PHQ-9 score ≥5; not concurrently receiving psychological treatment for depression	76	Denmark	35.0 (13.96)	74	PHQ-9	15.25 (4.04)	Mathiasen, K., Andersen, T. E., Lichtenstein, M. B., Ehlers, L. H., Riper, H., Kleiboer, A., & Roessler, K. K. (2022). The clinical effectiveness of blended cognitive behavioral therapy compared with face-to-face cognitive behavioral therapy for adult depression: randomized controlled noninferiority trial. Journal of medical Internet research, 24(9), e36577.

**Less severe depression**										
12	Shih 2021	1. Mindfulness-based cognitive therapy (MBCT) group<br>2. Supervised low intensity exercise group	Community-dwelling adults aged at least 60 years; HAMD score=8-18; normal cognitive functioning (score of ≥22 on Montreal Cognitive Assessment [MoCA]); does not practise meditation or mindfulness regularly	57	Hong Kong	70.3 (6.6)	88	HAMD	12.75 (3.45)	Shih, V. W., Chan, W. C., Tai, O. K., Wong, H. L., Cheng, C. P., & Wong, C. S. (2021). Mindfulness-based cognitive therapy for late-life depression: a randomised controlled trial. East Asian archives of psychiatry, 31(2), 27-35.
13	Vázquez 2023	1. Computerised-CBT (CCBT)<br>2. CBT group (under 15 sessions)<br>3. Attention placebo	Non-professional caregiver of a dependent person; CES-D score ≥16; do not meet DSM-5 criteria for a current or past major depressive episode; not received psychological or pharmacological treatment in the past 2 months	175	Spain	50.0 (9.8)	93	CES-D	25.43 (8.74)	Vázquez, F. L., Blanco, V., Hita, I., Torres, Á. J., Otero, P., Páramo, M., & Salmerón, M. (2023). Efficacy of a cognitive behavioral intervention for the prevention of depression in nonprofessional caregivers administered through a smartphone app: a randomized controlled trial. Journal of Clinical Medicine, 12(18), 5872.
14	Wu 2021	1. Self-administered positive psychological intervention<br>2. No treatment	Undergraduate students; displaying 1-2 of the 9 DSM-5 indicators of depression; BDI score=9-13; no previous history of depressive disorders, treatment with medication, or psychotherapy for depression	264	China	21.3 (2.5)	80	BDI	12.39 (2.18)	Wu, S. (2021). Affect mediates the influence of the. Social Behavior and Personality: an international journal, 49(11), 1-16.
15	Ying 2023	1. Computerised-CBT (CCBT)<br>2. CBT group (under 15 sessions)<br>3. Waitlist	Chinese resident aged at least 18 years; CES-D score ≥16; no medication for depression during the last month; did not	329	China	41.3 (13.4)	66	PHQ-9	12.41 (2.45)	Ying, Y., Ji, Y., Kong, F., Wang, M., Chen, Q., Wang, L., ... & Ruan, L. (2023). Efficacy of an internet-based cognitive behavioral therapy for subthreshold depression among Chinese adults: a randomized

Less severe depression

			meet DSM-IV criteria for a diagnosis of major depressive episode; no current or past month participation in psychotherapy							controlled trial. Psychological medicine, 53(9), 3932-3942.
16	Zhang 2022	1. Music therapy group<br>2. No treatment	University students aged 18-20 years; BDI score ≥10; Difficulties in Emotion Regulation Scale (DERS) score ≥101; not currently receiving music therapy or antidepressant treatment	75	China	18.3 (0.5)	52	BDI	16.23 (5.02)	Zhang, M., Ding, Y., Zhang, J., Jiang, X., Xu, N., Zhang, L., & Yu, W. (2022). Effect of group impromptu music therapy on emotional regulation and depressive symptoms of college students: a randomized controlled study. Frontiers in Psychology, 13, 851526.

More severe depression

	Study ID	NMA node: interventions	Inclusion criteria	N	Country	Mean age in years (SD)	Sex (% female)	Baseline depression scale	Mean baseline depression score (SD)	Reference
1	Asghar 2022	1. Escitalopram<br>2. Nortriptyline	Adults aged 20-50 years; met DSM-V criteria for a major depressive episode; newly diagnosed and treatment-naïve	500	Pakistan	NR	64	HAMD	22.4 (9.49)	Asghar, J., Tabasam, M., Althobaiti, M. M., Adnan Ashour, A., Aleid, M. A., Ibrahim Khalaf, O., & Aldhyani, T. H. (2022). A randomized clinical trial comparing two treatment strategies, evaluating the meaningfulness of HAM-D rating scale in patients with major depressive disorder. Frontiers in psychiatry, 13, 873693.
2	Bibi 2020	1. Computerised cognitive bias modification<br>2. Attention placebo	University students aged at least 18 years; QIDS score ≥6; not currently receiving treatment for a psychiatric condition	55	Pakistan	22.5 (1.6)	64	QIDS	12.23 (2.67)	Bibi, A., Margraf, J., & Blackwell, S. E. (2020). Positive imagery cognitive bias modification for symptoms of depression among university students in Pakistan: A pilot study. Journal of Experimental

**More severe depression**										
										Psychopathology, 11(2), 2043808720918030.
3	Giosan 2020*	1. CBT individual (under 15 sessions)<br>2. CBT individual (under 15 sessions)	Adults who met DSM-IV criteria for MDD (assessed with SCID); BDI-II score >13; not receiving medication	97	Romania	31.5 (10.6)	79	BDI-II	30.21 (10.87)	Giosan, C., Cobeanu, O., Wyka, K., Muresan, V., Mogoase, C., Szentagotai, A., ... & Moldovan, R. (2020). Cognitive evolutionary therapy versus standard cognitive therapy for depression: A single-blinded randomized clinical trial. Journal of Clinical Psychology, 76(10), 1818-1831.
4	Kadam 2020	1. Escitalopram<br>2. Amitriptyline	Newly diagnosed patients aged 18-60 years; met DSM V criteria for MDD; HAMD score ≥22; did not have severe depression (where psychiatrist judged pharmacotherapy alone likely to be insufficient); had not received ECT in past 3 months	40	India	36.7 (12.8)	45	MADRS	26.88 (2.07)	Kadam, R. L., Sontakke, S. D., Tiple, P., Motghare, V. M., Bajait, C. S., & Kalikar, M. V. (2020). Comparative evaluation of efficacy and tolerability of vilazodone, escitalopram, and amitriptyline in patients of major depressive disorder: a randomized, parallel, open-label clinical study. Indian Journal of Pharmacology, 52(2), 79-85.
5	Kim 2021	1. Electroacupuncture<br>2. Sham electrostimulation at non-specific points with no current	Adults aged 19-65 years; met DSM-IV criteria for MDD; HAMD score=7-24; not received any type of psychotropic drugs, psychotherapy, electroshock therapy, or transcranial magnetic stimulation in past month	30	Korea	50.3 (12.4)	77	HAMD	17.9 (2.7)	Kim, M., Choi, E. J., Kwon, O. J., Park, H. J., Kim, A. R., Seo, B. N., ... & Kim, J. H. (2021). Electroacupuncture plus moxibustion for major depressive disorder: A randomized, sham-controlled, pilot clinical trial. Integrative medicine research, 10(3), 100727.
6	Kramer 2022	1. Computerised-CBT (CCBT) with support<br>2. Computerised-CBT (CCBT)<br>3. Waitlist	Adults aged 18-65 years; met ICD-10 criteria for MDD or dysthymia; HAMD score ≥8; BDI-II score ≥13	401	Germany	37.1 (11.0)	83	BDI-II	30.5 (9.5)	Krämer, R., Köhne-Volland, L., Schumacher, A., & Köhler, S. (2022). Efficacy of a web-based intervention for depressive disorders: three-arm randomized controlled trial comparing guided

More severe depression										
										and unguided self-help with waitlist control. JMIR formative research, 6(4), e34330.
7	Kumar 2022	1. Amitriptyline<br>2. Fluoxetine	Met DSM-III-R criteria for a diagnosis of MDD; duration of current episode at least 1 month; HAMD score >17	50	India	41.1 (9.1)	50	HAMD	26.05 (5.9)	Kumar, S., Gaur, R. K., Azmi, S. A., & Akhouri, D. (2022). Effect Of Amitriptyline and Fluoxetine in Patients Presenting with Mixed Anxiety and Depression: Comparative Study. International Journal of Toxicological and Pharmacological Research, 12(2), 47-53
8	Rani 2021	1. Fluoxetine<br>2. Venlafaxine	Newly diagnosed patients aged 18-69 years; met ICD-10 criteria for a diagnosis of moderate or severe depression; not receiving any other medications	52	India	34.9 (12.0)	37	HAMD	20.34 (2.61)	Rani, S., Sindhu, N., Saini, R., Pandey, A.K., Narwat, A., & Garg, S. (2021). Comparison of the effects of antidepressants on cognition functions in patients of major depressive disorders in tertiary care hospital in haryana. Asian Journal of Pharmaceutical and Clinical Research, 14(3), 134-140.
9	Wang 2021a	1. Escitalopram<br>2. Escitalopram<br>3. Pill placebo	Outpatients aged 18-65 years; met DSM-IV criteria for a diagnosis of MDD; HAMD score >20; good physical health	390	China	NR	NR	MADRS	30.24 (6.11)	Wang, X., Fan, Y., Li, G., & Li, H. (2021). The efficacy of escitalopram in major depressive disorder: a multicenter randomized, placebo-controlled double-blind study. International Clinical Psychopharmacology, 36(3), 133-139.
10	Wang 2022a	1. Traditional acupuncture + fluoxetine<br>2. Fluoxetine	Adults aged 19-75 years; met DSM-IV criteria for a diagnosis of depression; HAMD score=17-24	160	China	47.1 (6.9)	69	HAMD	19.94 (2.29)	Wang, Y., Huang, Y.W., Ablikim, D., Lu, Q., Zhang, A.J., Dong, Y.Q., ... & Hu, Z.H. (2022). Efficacy of acupuncture at ghost points combined with fluoxetine in treating depression: A randomized study. World Journal of Clinical Cases, 10(3), 929.

*Giosan 2020 data were not included in the NMA due to severe convergence issues caused by the fact that the study compared the same intervention in both arms, but effects were very different between arms, leading to considerable heterogeneity compared with other relevant evidence in the dataset.

List of excluded studies with reasons for exclusion, and full references

	Study ID	Reference	Reason for exclusion
1	Alexopoulos 2021	Alexopoulos, G. S., Raue, P. J., Banerjee, S., Marino, P., Renn, B. N., Solomonov, N., ... & Areán, P. A. (2021). Comparing the streamlined psychotherapy “Engage” with problem-solving therapy in late-life major depression. A randomized clinical trial. Molecular psychiatry, 26(9), 5180-5189.	Population: <80% first-line treatment<br>*42% were on antidepressants during the trial*
2	Almeida 2021	Almeida, O. P., Patel, H., Kelly, R., Ford, A., Flicker, L., Robinson, S., ... & Thompson, S. (2021). Preventing depression among older people living in rural areas: a randomised controlled trial of behavioural activation in collaborative care. International journal of geriatric psychiatry, 36(4), 530-539.	Population: Not depression<br>*Below sub-threshold level (PHQ-9=7.4)*
3	Amano 2023	Amano, M., Katayama, N., Umeda, S., Terasawa, Y., Tabuchi, H., Kikuchi, T., ... & Nakagawa, A. (2023). The effect of cognitive behavioral therapy on future thinking in patients with major depressive disorder: A randomized controlled trial. Frontiers in Psychiatry, 14, 997154.	Population: <80% non-chronic depression<br>*53% had chronic depression (MDD≥2 years)*
4	Andersson 2023	Andersson, G., Käll, A., Juhlin, S., Wahlström, C., de Fine Licht, E., Färdeman, S., ... & Berg, M. (2023). Free choice of treatment content, support on demand and supervision in internet-delivered CBT for adults with depression: A randomized factorial design trial. Behaviour Research and Therapy, 162, 104265.	Study design: Dismantling study<br>*8 arms in a factorial design, all participants received CCBT and factors varied according to self-/therapist-chosen content, regular therapist support/support-on-demand, and supervision available/not available*
5	Anuwatgasem 2020	Anuwatgasem, C., Awirutworakul, T., Vallibhakara, S. A. O., Kaisa-ard, P., Yamnim, T., Phadermphol, K., ... & Jullagate, S. (2020). The Effects of Mindfulness and Self-Compassion-Based Group Therapy for Major Depressive Disorder: A Randomized Controlled Trial. Journal of the Medical Association of Thailand, 103(9).	Paper unavailable
6	Ara 2023	Ara, J., Deeba, F., & Dobson, K. S. (2023). A randomized controlled trial of the effectiveness of cognitive behavior therapy for major depression in Bangladesh. International journal of cognitive therapy, 16(2), 222-236.	Outcome measure(s) outside protocol<br>*Depression Scale (DS; Uddin & Rahman, 2005)*
7	Arroll 2022	Arroll, B., Frischtak, H., Roskvist, R., Mount, V., Sundram, F., Fletcher, S., ... & van der Werf, B. (2022). FACT effectiveness in primary care; a single visit RCT for depressive symptoms. The International Journal of Psychiatry in Medicine, 57(2), 91-102.	Intervention: Psychological intervention not of interest<br>*Information and support/MI type single session intervention*
8	Barkham 2021	Barkham, M., Saxon, D., Hardy, G. E., Bradburn, M., Galloway, D., Wickramasekera, N., ... & Brazier, J. E. (2021). Person-centred experiential therapy versus cognitive behavioural therapy delivered in the English Improving Access to Psychological Therapies service for the treatment of moderate or severe depression (PRaCTICED): a pragmatic, randomised, non-inferiority trial. The Lancet Psychiatry, 8(6), 487-499.	Population: <80% first-line treatment<br>*58% using psychotropic medication at baseline*
9	Bieber 2021	Bieber, M., Görgülü, E., Schmidt, D., Zabel, K., Etyemez, S., Friedrichs, B., ... & Oertel, V. (2021). Effects of body-oriented yoga: a RCT study for patients with major depressive disorder. European archives of psychiatry and clinical neuroscience, 271, 1217-1229.	Population: <80% first-line treatment<br>*76% receiving medication at baseline and 93% psychotherapy*
10	Bisby 2023	Bisby, M. A., Balakumar, T., Scott, A. J., Titov, N., & Dear, B. F. (2024). An online therapist-guided ultra-brief treatment for depression and anxiety: a randomized controlled trial. Psychological Medicine, 54(5), 902-913.	Population: <80% first-line treatment<br>*43% receiving medication at baseline*
11	Bo 2023	Bø, R., Kraft, B., Pedersen, M. L., Joormann, J., Jonassen, R., Osnes, K., ... & Landrø, N. I. (2023). The effect of attention bias modification on depressive symptoms in a comorbid sample: a randomized controlled trial. Psychological Medicine, 53(13), 6389-6396.	Population: <80% first-line treatment<br>*27% on current SSRI at baseline*

	Study ID	Reference	Reason for exclusion
12	Bolinski 2022	Bolinski, F., Kleiboer, A., Neijenhuijs, K., Karyotaki, E., Wiers, R., de Koning, L., ... & Riper, H. (2022). Challenges in recruiting university students for web-based indicated prevention of depression and anxiety: results from a randomized controlled trial (ICare Prevent). Journal of medical Internet research, 24(12), e40892.	Sample size N<10 per arm<br>*Post-test assessment completed by <10 in 2 of the 3 arms*
13	Boucher 2023	Boucher, V. G., Haight, B. L., Hives, B. A., Zumbo, B. D., Merali-Dewji, A., Hutton, S., ... & Puterman, E. (2023). Effects of 12 weeks of At-Home, application-based Exercise on Health Care workers' depressive symptoms, Burnout, and absenteeism: a Randomized Clinical Trial. JAMA psychiatry, 80(11), 1101-1109.	Data cannot be extracted<br>*Only effect size reported*
14	Brose 2023	Brose, A., Heinrich, M., Bohn, J., Kampisiou, C., Zagorscak, P., & Knaevelsrud, C. (2023). Sequencing effects of behavioral activation and cognitive restructuring in an Internet-based intervention for depressed adults are negligible: Results from a randomized controlled trial. Journal of Consulting and Clinical Psychology, 91(3), 122.	Population: <80% first-line treatment<br>*24% receiving antidepressant medication at baseline*
15	Bur 2022	Bur, O. T., Krieger, T., Moritz, S., Klein, J. P., & Berger, T. (2022). Optimizing the context of support of web-based self-help in individuals with mild to moderate depressive symptoms: a randomized full factorial trial. Behaviour research and therapy, 152, 104070.	Study design: Dismantling study<br>*16 condition factorial design*
16	Cardozo-Batista 2020	Cardozo-Batista, L., & Tucci, A. M. (2020). Effectiveness of an alternative intervention in the treatment of depressive symptoms. Journal of Affective Disorders, 276, 562-569.	Intervention: Combination intervention not of interest<br>*Combination intervention using Mindfulness Meditation, Reiki, Acupuncture and Auriculotherapy*
17	Chen 2021	Chen, Z., Yan, L., Nong, X., Chen, F., Gan, F., Tang, P., ... & Huang, L. (2021). Impact of citalopram combined with mindfulness-based stress reduction on symptoms, cognitive functions and self-confidence in patients with depression. Tropical Journal of Pharmaceutical Research, 20(10), 2135-2142.	Design: Non-RCT<br>*No mention of randomisation*
18	Ciccolo 2022	Ciccolo, J. T., Louie, M. E., SantaBarbara, N. J., Webster, C. T., Whitworth, J. W., Nosrat, S., ... & Busch, A. M. (2022). Resistance training for Black men with depressive symptoms: a pilot randomized controlled trial to assess acceptability, feasibility, and preliminary efficacy. BMC psychiatry, 22(1), 283.	Population: <80% first-line treatment<br>*28% receiving psychiatric treatment for depression at baseline*
19	Cruwys 2022	Cruwys, T., Haslam, C., Rathbone, J. A., Williams, E., Haslam, S. A., & Walter, Z. C. (2022). Groups 4 Health versus cognitive–behavioural therapy for depression and loneliness in young people: randomised phase 3 non-inferiority trial with 12-month follow-up. The British Journal of Psychiatry, 220(3), 140-147.	Outcome measure(s) outside protocol<br>*DASS-21*
20	Cullen 2021	Cullen, B., Eichel, K., Lindahl, J. R., Rahrig, H., Kini, N., Flahive, J., & Britton, W. B. (2021). The contributions of focused attention and open monitoring in mindfulness-based cognitive therapy for affective disturbances: A 3-armed randomized dismantling trial. PLoS One, 16(1), e0244838.	Data cannot be extracted<br>*Depression outcome on IDS cannot be extracted and DASS-21 outside protocol*
21	Cully 2023	Cully, J. A., Hundt, N. E., Fletcher, T., Sansgiry, S., Zeno, D., Kauth, M. R., ... & Sorocco, K. (2024). Brief cognitive-behavioral therapy for Depression in Community clinics: a hybrid effectiveness-implementation trial. Psychiatric services, 75(3), 237-245.	Paper unavailable
22	Daengruan 2021	Daengruan, P., Chairat, R., Jenraumjit, R., Chinwong, D., Oon-Arom, A., Klaphajone, J., & Arunmanakul, P. (2021). Effectiveness of receptive music therapy with imbedded 10 Hz binaural beats compared with standard care for patients with major depressive disorder: a randomized controlled trial. Complementary Therapies in Medicine, 61, 102765.	Sample size N<10 per arm<br>*N=9 in each arm*
23	Dafsari 2023	Dafsari, F. S., Bewernick, B., Böhringer, S., Domschke, K., Elsaesser, M., Löbner, M., ... & Jessen, F. (2023). Cognitive Behavioral Therapy for Late-Life Depression (CBTlate): Results of a Multicenter, Randomized, Observer-Blinded, Controlled Trial. Psychotherapy and Psychosomatics, 92(3), 180-192.	Population: <80% first-line treatment<br>*45% current use of psychopharmacological drugs at baseline*

	Study ID	Reference	Reason for exclusion
24	Davis 2023	Davis, C. H., Twohig, M. P., & Levin, M. E. (2023). Choosing ACT or CBT: A preliminary test of incorporating client preferences for depression treatment with college students. Journal of Affective Disorders, 325, 413-420.	Population: <80% first-line treatment<br>*37% current psychiatric medication at baseline*
25	Deady 2022	Deady, M., Glozier, N., Calvo, R., Johnston, D., Mackinnon, A., Milne, D., ... & Harvey, S. B. (2022). Preventing depression using a smartphone app: a randomized controlled trial. Psychological medicine, 52(3), 457-466.	Data cannot be extracted<br>*PHQ-9 means not reported at baseline or post-intervention*
26	D'Elia 2020	D'Elia, A., Bawor, M., Dennis, B. B., Bhatt, M., Litke, K., McCabe, K., ... & Samaan, Z. (2020). Feasibility of behavioral activation group therapy in reducing depressive symptoms and improving quality of life in patients with depression: the BRAVE pilot trial. Pilot and feasibility studies, 6, 1-11.	Population: Further-line treatment<br>*All participants currently receiving treatment for depression at the clinic*
27	Dunn 2023	Dunn, B. D., Widnall, E., Warbrick, L., Warner, F., Reed, N., Price, A., ... & Kuyken, W. (2023). Preliminary clinical and cost effectiveness of augmented depression therapy versus cognitive behavioural therapy for the treatment of anhedonic depression (ADepT): a single-centre, open-label, parallel-group, pilot, randomised, controlled trial. EClinicalMedicine, 61.	Population: <80% first-line treatment<br>*66% taking antidepressants at baseline*
28	Ejiri 2023	Ejiri, H., Uchida, H., Tsuchiya, K., Fujiwara, K., Kikuchi, S., & Hirao, K. (2023). Immediate Effects of Mobile Phone App for Depressed Mood in Young Adults with Subthreshold Depression: A Pilot Randomized Controlled Trial. Neuropsychiatric Disease and Treatment, 1695-1707.	Outcome measure(s) outside protocol<br>*Depression-dejection on the Profile of Mood States 2nd Edition-Adult Short (POMS 2-A Short)*
29	Ezeudu 2020	Ezeudu, F. O., Eya, N. M., Nwafor, S. C., & Ogbonna, C. S. (2020). Intervention for depression among chemistry education undergraduates in a Nigerian university. Journal of International Medical Research, 48(1), 0300060519865064.	Design: Non-RCT<br>*N=72 eligible but only the 'first' 23 randomised*
30	Falkenström 2022	Falkenström, F., & Holmqvist, R. (2022). Therapist in-session feelings predict change in depressive symptoms in interpersonal and brief relational psychotherapy. Psychotherapy Research, 32(5), 571-584.	Data cannot be extracted
31	Fawcett 2020	Fawcett, E., Neary, M., Ginsburg, R., & Cornish, P. (2020). Comparing the effectiveness of individual and group therapy for students with symptoms of anxiety and depression: A randomized pilot study. Journal of American College Health, 68(4), 430-437.	Data cannot be extracted
32	Fernandes 2022	Fernandes, B. M., Siqueira, C. C., Vieira, R. M., Moreno, R. A., & Soeiro-de-Souza, M. G. (2022). Physical activity as an adjuvant therapy for depression and influence on peripheral inflammatory markers: A randomized clinical trial. Mental Health and Physical Activity, 22, 100442.	Secondary analysis with no new relevant or usable data<br>*Secondary analysis of Siqueira 2016*
33	Funderburk 2021	Funderburk, J. S., Pigeon, W. R., Shepardson, R. L., Wade, M., Acker, J., Fivecoat, H., ... & Maisto, S. A. (2021). Treating depressive symptoms among veterans in primary care: A multi-site RCT of brief behavioral activation. Journal of affective disorders, 283, 11-19.	Population: <80% first-line treatment<br>*34% current psychotropic medication at baseline*
34	Gilbody 2022	Gilbody, S., Brabyn, S., Mitchell, A., Ekers, D., McMillan, D., Bailey, D., ... & Bosanquet, K. (2022). Can we prevent depression in at-risk older adults using self-help? the UK SHARD trial of behavioral activation. The American Journal of Geriatric Psychiatry, 30(2), 197-207.	Population: Not depression<br>*Below subthreshold level (PHQ-9=8.17)*
35	Gili 2020	Gili, M., Castro, A., García-Palacios, A., Garcia-Campayo, J., Mayoral-Cleries, F., Botella, C., ... & Baños, R. M. (2020). Efficacy of three low-intensity, internet-based psychological interventions for the treatment of depression in primary care: randomized controlled trial. Journal of medical Internet research, 22(6), e15845.	Data cannot be extracted

	Study ID	Reference	Reason for exclusion
36	Graham 2020	Graham, A. K., Greene, C. J., Kwasny, M. J., Kaiser, S. M., Lieponis, P., Powell, T., & Mohr, D. C. (2020). Coached mobile app platform for the treatment of depression and anxiety among primary care patients: a randomized clinical trial. JAMA psychiatry, 77(9), 906-914.	Population: <80% first-line treatment<br>*56% current pscyhotropic medication at baseline*
37	Gruhn 2021	Gruhn, M. A., Phan, K. L., Klumpp, H., Ajilore, O., & Gorka, S. M. (2021). Early life adversity as a moderator of symptom change following selective serotonin reuptake inhibitors and cognitive behavioral therapy. Cognitive Therapy and Research, 45, 343-354	Intervention: Psychological intervention not of interest<br>*Treatment tailored to principal diagnosis and only 44% MDD*
38	Hadjistavropoulos 2022	Hadjistavropoulos, H. D., Peynenburg, V., Thiessen, D. L., Nugent, M., Karin, E., Dear, B. F., & Titov, N. (2022). A randomized factorial trial of internet-delivered cognitive behavioural therapy: An 8-week program with or without extended support and booster lesson. Internet Interventions, 27, 100499.	Study design: Dismantling study<br>*Factorial design with all participants receiving CCBT, and components of interest were with/without extended support and with/without booster lesson*
39	He 2022	He, X. (2022). Physical activity in the treatment of depression in college students. Revista Brasileira de Medicina do Esporte, 28, 68-71.	Data cannot be extracted<br>*Unclear what scale used to measure depression and only subscales reported*
40	Heim 2021	Heim, E., Abi Ramia, J., Abi Hana, R., Burchert, S., Carswell, K., Cornelisz, I., ... & Van't Hof, E. (2021). Step-by-step: feasibility randomised controlled trial of a mobile-based intervention for depression among populations affected by adversity in Lebanon. Internet interventions, 24, 100380.	Completion data <50%/>50% left treatment early<br>*20% of those eligible/randomised completed post-treatment assessment*
41	Heinzel 2022	Heinzel, S., Schwefel, M., Sanchez, A., Heinen, D., Fehm, L., Henze, R., ... & Heissel, A. (2022). Physical exercise training as preceding treatment to cognitive behavioral therapy in mild to moderate major depressive disorder: A randomized controlled trial. Journal of Affective Disorders, 319, 90-98.	Population: <80% first-line treatment<br>*40% receiving antidepressant medication at baseline*
42	Hidalgo 2021	Hidalgo, J. L. T., & Sotos, J. R. (2021). Effectiveness of physical exercise in older adults with mild to moderate depression. The Annals of Family Medicine, 19(4), 302-309.	Completion data <50%/>50% left treatment early<br>*49% completion*
43	Hill 2021	Hill, K. R., Gardus, J. D., Bartlett, E. A., Perlman, G., Parsey, R. V., & DeLorenzo, C. (2021). Measuring brain glucose metabolism in order to predict response to antidepressant or placebo: A randomized clinical trial. NeuroImage: Clinical, 32, 102858.	Data cannot be extracted<br>*Study concerned with clinical prediction of antidepressant response and only results reported for placebo arm are remission*
44	Hirokawa-Ueda 2023	Hirokawa-Ueda, H., Sawamura, Y., Kawakami, T., Sakane, H., Teramoto, K., Yamamoto, A., ... & Ono, H. (2023). Interpersonal counseling versus active listening in the treatment of mild depression: a randomized controlled trial. Journal of Physical Therapy Science, 35(7), 533-537.	Outcome measure(s) outside protocol<br>*Self-rating Depression Scale (SDS)*
45	Hsu Kean 2022	Hsu, K. J., Shumake, J., Caffey, K., Risom, S., Labrada, J., Smits, J. A., ... & Beevers, C. G. (2022). Efficacy of attention bias modification training for depressed adults: A randomized clinical trial. Psychological medicine, 52(16), 3865-3873.	Population: <80% first-line treatment<br>*23% on psychiatric medication at baseline*
46	Janssen 2023	Janssen, N. P., Lucassen, P., Huibers, M. J., Ekers, D., Broekman, T., Bosmans, J. E., ... & Hendriks, G. J. (2023). Behavioural activation versus treatment as usual for depressed older adults in primary care: a pragmatic cluster-randomised controlled trial. Psychother Psychosom, 92, 255-266.	Population: <80% first-line treatment<br>*32% receiving antidepressant medication at baseline*
47	Jian-Ke 2022	Jian-ke, G., Hai-long, L., Zun, Z., & Jie, L. (2022). Clinical Study on Therapeutic Effect of Acupuncture Combined with SSRI Drugs in the Treatment of Depression and Anxiety. International Journal of Clinical Acupuncture, 31(3).	Paper unavailable

	Study ID	Reference	Reason for exclusion
48	Jonsson 2020	Jonsson, G., Franzén, L., Nyström, M. B., & Davis, P. A. (2020). Integrating yoga with psychological group-treatment for mixed depression and anxiety in primary healthcare: An explorative pilot study. Complementary Therapies in Clinical Practice, 41, 101250.	Design: Non-RCT
49	Kang 2021	Kang, H., & Jang, S. (2021). Effect of mindfulness yoga on depression severity, self-esteem, and quality of life in middle-aged men. Iranian Journal of Public Health, 50(7), 1334.	Design: Non-RCT
50	Karimpour-Vazifehkhorani 2020	Karimpour-Vazifehkhorani, A., Rudsari, A. B., Rezvanizadeh, A., Kehtary-Harzang, L., & Hasanzadeh, K. (2020). Behavioral activation therapy on reward seeking behaviors in depressed people: An experimental study. Journal of Caring Sciences, 9(4), 195.	Design: Non-RCT
51	Karlsson-Good 2023	Karlsson-Good, M., Kaldo, V., Lundberg, L., Kraepelien, M., Anthony, S. A., & Holländare, F. (2023). Increasing the accessibility to internet-based cognitive behavioural therapy for depression: A single-blind randomized controlled trial of condensed versus full-text versions. Internet Interventions, 34, 100678.	Population: <80% first-line treatment<br>*36% taking antidepressant medication at baseline*
52	Karyotaki 2022	Karyotaki, E., Klein, A. M., Ciharova, M., Bolinski, F., Krijnen, L., de Koning, L., ... & Cuijpers, P. (2022). Guided internet-based transdiagnostic individually tailored Cognitive Behavioral Therapy for symptoms of depression and/or anxiety in college students: A randomized controlled trial. Behaviour research and therapy, 150, 104028.	Population: Not depression<br>*Below subthreshold level (PHQ-9=8.23)*
53	Katayama 2022	Katayama, N., Nakagawa, A., Umeda, S., Terasawa, Y., Abe, T., Kurata, C., ... & Mimura, M. (2022). Cognitive behavioral therapy effects on frontopolar cortex function during future thinking in major depressive disorder: A randomized clinical trial. Journal of Affective Disorders, 298, 644-655.	Population: <80% first-line treatment<br>*92% receiving at least 1 antidepressant medication at baseline*
54	Keller-Varady 2023	Keller-Varady, K., Haufe, S., Schieffer, E., Kerling, A., Tegtbur, U., & Kahl, K. G. (2023). Personalized training as a promoter for physical activity in people with depressive disorder—a randomized controlled trial in Germany. Frontiers in Psychiatry, 14, 1158705.	Population: <80% first-line treatment<br>*58% receiving antidepressants at baseline*
55	Khalili 2022	Khalili, D., Khalili, N., & Jafari, E. (2022). Effectiveness of positive psychotherapy on depression and alexithymia in women applying for a divorce. Depression research and treatment, 2022.	Design: Non-RCT
56	Kingston 2020	Kingston, J., Becker, L., Woeginger, J., & Ellett, L. (2020). A randomised trial comparing a brief online delivery of mindfulness-plus-values versus values only for symptoms of depression: Does baseline severity matter?. Journal of Affective Disorders, 276, 936-944.	Outcome measure(s) outside protocol<br>*DASS-21*
57	Klos 2021	Klos, M. C., Escoredo, M., Joerin, A., Lemos, V. N., Rauws, M., & Bunge, E. L. (2021). Artificial intelligence–based chatbot for anxiety and depression in university students: pilot randomized controlled trial. JMIR formative research, 5(8), e20678.	Data cannot be extracted<br>*Only medians reported for depression*
58	Kratzer 2021	Kratzer, A., Luttenberger, K., Karg-Hefner, N., Weiss, M., & Dorscht, L. (2021). Bouldering psychotherapy is effective in enhancing perceived self-efficacy in people with depression: Results from a multicenter randomized controlled trial. BMC psychology, 9, 1-14.	Population: <80% first-line treatment<br>*50% receiving antidepressants at baseline*
59	La Rocque 2021	La Rocque, C. L., Mazurka, R., Stuckless, T. J., Pyke, K., & Harkness, K. L. (2021). Randomized controlled trial of bikram yoga and aerobic exercise for depression in women: Efficacy and stress-based mechanisms. Journal of Affective Disorders, 280, 457-466.	Population: <80% first-line treatment<br>*45% receiving current treatment at baseline*
60	Latif 2021	Latif, M., Awan, F., Gul, M., Husain, M. O., Husain, M. I., Sayyed, K., ... & Naeem, F. (2021). Preliminary evaluation of a culturally adapted CBT-based online programme for depression and anxiety from a lower middle-income country. the Cognitive Behaviour Therapist, 14, e36.	Baseline severity cannot be categorised

	Study ID	Reference	Reason for exclusion
61	Lavretsky 2022	Lavretsky, H., Milillo, M. M., Kilpatrick, L., Grzenda, A., Wu, P., Nguyen, S. A., ... & Siddarth, P. (2022). A randomized controlled trial of Tai Chi Chih or health education for geriatric depression. The American Journal of Geriatric Psychiatry, 30(3), 392-403.	Population: <80% non-chronic depression<br>*70% have chronic depression (>2 years)*
62	Lechinger 2021	Lechinger, J., Koch, J., Weinhold, S. L., Seeck-Hirschner, M., Stingele, K., Kropp-Näf, C., ... & Göder, R. (2021). REM density is associated with treatment response in major depression: Antidepressant pharmacotherapy vs. psychotherapy. Journal of psychiatric research, 133, 67-72.	Data cannot be extracted<br>*Depression outcome data in figure*
63	Lee 2022	Lee, E., Han, Y., Cha, Y. J., Oh, J. H., Hwang, N. R., Seo, H. J., & Choi, K. H. (2022). Community-based multi-site randomized controlled trial of behavioral activation for patients with depressive disorders. Community Mental Health Journal, 1-13.	Population: <80% first-line treatment<br>*89% receiving medication at baseline*
64	Lin 2023	Lin, Z., Cheng, L., Han, X., Wang, H., Liao, Y., Guo, L., ... & McIntyre, R. S. (2023). The Effect of Internet-Based Cognitive Behavioral Therapy on Major Depressive Disorder: Randomized Controlled Trial. Journal of Medical Internet Research, 25, e42786.	Population: <80% first-line treatment<br>*57% using AD medication at baseline*
65	Lopes 2023	Lopes, R. T., da Rocha, G. C., Svacina, M. A., Meyer, B., Šipka, D., & Berger, T. (2023). Effectiveness of an internet-based self-guided program to treat depression in a sample of Brazilian users: Randomized controlled trial. JMIR formative research, 7(1), e46326.	Population: <80% first-line treatment<br>*40% receiving treatment at baseline (pharmacology, psychotherapy or both)*
66	Lu 2021	Lu, S. H., Assudani, H. A., Kwek, T. R., Ng, S. W., Teoh, T. E., & Tan, G. C. (2021). A randomised controlled trial of clinician-guided internet-based cognitive behavioural therapy for depressed patients in Singapore. Frontiers in Psychology, 12, 668384.	Population: <80% first-line treatment<br>*49% taking antidepressant medication at baseline*
67	Lukas 2021	Lukas, C. A., Eskofier, B., & Berking, M. (2021). A gamified smartphone-based intervention for depression: randomized controlled pilot trial. JMIR Mental Health, 8(7), e16643.	Population: <80% first-line treatment<br>*34% receiving treatment at baseline*
68	Luttenberger 2022	Luttenberger, K., Karg-Hefner, N., Berking, M., Kind, L., Weiss, M., Kornhuber, J., & Dorscht, L. (2022). Bouldering psychotherapy is not inferior to cognitive behavioural therapy in the group treatment of depression: A randomized controlled trial. British Journal of Clinical Psychology, 61(2), 465-493.	Population: <80% first-line treatment<br>*56% receiving antidepressant medication at baseline*
69	Mason 2023a	Mason, M. J., Coatsworth, J. D., Zaharakis, N., Russell, M., Brown, A., & McKinstry, S. (2023). Testing Mechanisms of Change for Text Message–Delivered Cognitive Behavioral Therapy: Randomized Clinical Trial for Young Adult Depression. JMIR mHealth and uHealth, 11, e45186.	Data cannot be extracted
70	Mason 2023b	Mason, M. J., Coatsworth, J. D., Zaharakis, N., Russell, M., Wallis, D., Brown, A., & Hale, C. (2023). Treating young adult depression with text-delivered cognitive behavioral therapy: a pilot randomized clinical trial. Behavior Therapy, 54(2), 315-329.	Data cannot be extracted
71	Meganck 2023	Meganck, R., Desmet, M., Van Nieuwenhove, K., De Smet, M., Hennissen, V., Truijens, F., ... & Vanheule, S. (2023). The Ghent psychotherapy study: A pragmatic, stratified, randomized parallel trial into the differential efficacy of psychodynamic and cognitive-behavioral interventions in dependent and self-critical depressive patients. Psychotherapy and Psychosomatics, 92(4), 267-278.	Population: <80% first-line treatment<br>*86% treatment before therapy (including 38% antidepressants)*
72	Meleppurakkal 2021	Meleppurakkal, S., Sunitha, K., & Jayan, D. (2021). Effect of selected yoga techniques along with ashwagandha and vacha in depression in the age group 65-75 years. Asian Journal of Pharmaceutical and Clinical Research, 14(11), 65-68	Intervention: Pharmacological intervention not of interest (only 1 arm meets inclusion criteria)<br>*Ayurvedic powder (churna) was provided to participants on its own or in addition to yoga in 2 of the 3 study arms*

	Study ID	Reference	Reason for exclusion
73	Meyer 2022	Meyer, J. D., Perkins, S. L., Brower, C. S., Lansing, J. E., Slocum, J. A., Thomas, E. B., ... & Wade, N. G. (2022). Feasibility of an exercise and CBT intervention for treatment of depression: a pilot randomized controlled trial. Frontiers in Psychiatry, 13, 799600.	Sample size N<10 per arm
74	Moloud 2022	Moloud, R., Saeed, Y., Mahmonir, H., & Rasool, G. A. (2022). Cognitive-behavioral group therapy in major depressive disorder with focus on self-esteem and optimism: an interventional study. BMC psychiatry, 22(1), 299.	Outcome(s) not of interest<br>*No depression outcome reported*
75	Morris 2023	Morris, L., Lovell, K., McEvoy, P., Emsley, R., Carter, L. A., Edge, D., ... & Mansell, W. (2023). A brief transdiagnostic group (the take control course) compared to individual low-intensity CBT for depression and anxiety: a randomized non-inferiority trial. Cognitive Behaviour Therapy, 52(3), 176-197.	Population: <80% first-line treatment<br>*66% taking psychiatric medication at baseline*
76	Moskowitz 2021	Moskowitz, J. T., Addington, E. L., Shiu, E., Bassett, S. M., Schuette, S., Kwok, I., ... & Cheung, E. O. (2021). Facilitator contact, discussion boards, and virtual badges as adherence enhancements to a web-based, self-guided, positive psychological intervention for depression: randomized controlled trial. Journal of Medical Internet Research, 23(9), e25922.	Study design: Dismantling study<br>*9 arms examining the effects of 3 enhancements (facilitator contact, an online discussion board, and virtual badges) on adherence*
77	Moskowitz 2023	Moskowitz, J. T., Jackson, K., Freedman, M. E., Grote, V. E., Kwok, I., Schuette, S. A., ... & Addington, E. L. (2023). Positive psychological intervention effects on depression: Positive emotion does not mediate intervention impact in a sample with elevated depressive symptoms. Affective Science, 4(1), 163-173.	Study design: Dismantling study<br>*Randomised into 9 arms but reports aggregated intervention versus control*
78	Murphy 2021	Murphy, S. T., Cooper, A. A., Hollars, S. N., & Strunk, D. R. (2021). Who benefits from a cognitive vs. behavioral approach to treating depression? A pilot study of prescriptive predictors. Behavior Therapy, 52(6), 1433-1448.	Population: <80% first-line treatment<br>*29% taking antidepressant medication*
79	Murri 2021	Murri, M. B., Amore, M., Menchetti, M., Toni, G., Neviani, F., Cerri, M., ... & Safety and Efficacy of Exercise for Depression in Seniors (SEEDS) Study Group. (2021). Physical exercise for late-life major depression. Focus, 19(3), 365-373.	Non-primary paper<br>*Reprint of Murri 2015*
80	Musa 2021	Musa, Z. A., Soh, K. L., Mukhtar, F., Soh, K. Y., Oladele, T. O., & Soh, K. G. (2021). Impact of Mindfulness-Based Cognitive Therapy on Depressive Symptoms Reduction among Depressed Patients in Nigeria: A Randomized Controlled Trial. Issues in mental health nursing, 42(7), 667-675.	Population: Relapse prevention<br>*Inclusion criterion current remission from MDD for at least 1 month*
81	Narayan 2022	Narayan, G. A., Hill, K. R., Wengler, K., He, X., Wang, J., Yang, J., ... & DeLorenzo, C. (2022). Does the change in glutamate to GABA ratio correlate with change in depression severity? A randomized, double-blind clinical trial. Molecular psychiatry, 27(9), 3833-3841.	Population: <80% first-line treatment<br>*22% on 'ineffective' medication at enrollment*
82	Nezafat 2023	Ferizi, J. N., Ashouri, A., Gharraee, B., & Farid, A. A. A. (2023). Comparison of the Effectiveness of Interpersonal Counseling and Interpersonal Psychotherapy in Emotional Expression, Social Skills, and Depression Symptoms in Students. Progress in Psychiatry and Behavioral Sciences, (In Press).	Data cannot be extracted
83	Oehler 2020	Oehler, C., Görges, F., Rogalla, M., Rummel-Kluge, C., & Hegerl, U. (2020). Efficacy of a guided web-based self-management intervention for depression or dysthymia: randomized controlled trial with a 12-month follow-up using an active control condition. Journal of medical Internet research, 22(7), e15361.	Population: <80% first-line treatment<br>*64% taking antidepressants and 55% receiving psychotherapy at baseline*
84	Okeke 2023	Okeke, N. M., Onah, B. O., Ekwealor, N. E., Ekwueme, S. C., Ezugwu, J. O., Edeh, E. N., ... & Obeagu, E. I. (2023). Effect of a randomized group intervention for depression among Nigerian pre-service adult education teachers. Medicine, 102(27), e34159.	Intervention: Psychological intervention not of interest
85	Pandey 2023	Pandey, R., Tiwari, G. K., & Rai, P. K. (2023). Understanding the efficacy of self-affirmation intervention for subclinical depression among young adults. Practice in Clinical Psychology, 11(1), 23-34.	Outcome measure(s) outside protocol<br>*Depressive episodes and depressive tendencies scale (Aarø et al. 2011)*

	Study ID	Reference	Reason for exclusion
86	Paterniti 2022	Paterniti, S., Raab, K., Sterner, I., Collimore, K. C., Dalton, C., & Bisserbe, J. C. (2022). Individual mindfulness-based cognitive therapy in major depression: A feasibility study. Mindfulness, 13(11), 2845-2856.	Population: <80% first-line treatment<br>*85% taking antidepressants at baseline*
87	Pott 2022	Pott, S. L., Kellett, S., Green, S., Daughters, S., & Delgadillo, J. (2022). Behavioral activation for depression delivered by drug and alcohol treatment workers: A pilot randomized controlled trial. Journal of Substance Abuse Treatment, 139, 108769.	Population: <80% first-line treatment<br>*47% prescribed antidepressants at baseline*
88	Raevuori 2021	Raevuori, A., Vahlberg, T., Korhonen, T., Hilgert, O., Aittakumpu-Hyden, R., & Forman-Hoffman, V. (2021). A therapist-guided smartphone app for major depression in young adults: a randomized clinical trial. Journal of Affective Disorders, 286, 228-238.	Population: <80% first-line treatment<br>*56% on antidepressants at baseline*
89	Rafiei 2022	Rafiei Alhosaini, N., Zarrin, H., & Rezaei-Jamalouei, H. (2022). Pilot Randomized Controlled Trial of Interpersonal Psychotherapy for Women With Depression in Iran. American Journal of Psychotherapy, 75(4), 181-185.	Data cannot be extracted<br>*Post-intervention BDI-II only in figure*
90	Rao 2020	Rao, U. T., Noronha, J. A., & Adiga, K. (2020). Effect of aerobic exercises on depressive symptoms, anxiety, self-esteem, and quality of life among adults with depression. Clinical Epidemiology and Global Health, 8(4), 1147-1151.	Design: Non-RCT
91	Rauen 2020	Rauen, K., Vetter, S., Eisele, A., Biskup, E., Delsignore, A., Rufer, M., & Weidt, S. (2020). Internet cognitive behavioral therapy with or without face-to-face psychotherapy: a 12-weeks clinical trial of patients with depression. Frontiers in Digital Health, 2, 544177.	Design: Non-RCT<br>*No mention of randomisation*
92	Raza 2023	Raza, S., Ahmed, S., Islam, R., Ahmed, M., Ashraf, S., Islam, H., ... & Mumtaz, H. (2023). Sertraline versus escitalopram in South Asians with moderate to severe major depressive disorder:(SOUTH-DEP) a double-blind, parallel, randomized controlled trial. Annals of Medicine and Surgery, 85(10), 4851-4859.	Data cannot be extracted<br>*Duration of drug treatment not clear 8 or 16 weeks but unclear at what timepoint measurements taken and what criteria were for shorter or longer duration*
93	Richards 2020	Richards, D., Enrique, A., Eilert, N., Franklin, M., Palacios, J., Duffy, D., ... & Timulak, L. (2020). A pragmatic randomized waitlist-controlled effectiveness and cost-effectiveness trial of digital interventions for depression and anxiety. NPJ digital medicine, 3(1), 85.	Population: <80% first-line treatment<br>*44% taking psychotropic medication at baseline*
94	Ruehlman 2023	Ruehlman, L., & Karoly, P. (2023). A pilot test of Internet-delivered brief interactive training sessions for depression: Evaluating dropout, uptake, adherence, and outcome. Journal of American college health, 71(7), 2131-2139.	Intervention: Psychological intervention not of interest
95	Saisanan Na 2020	Saisanan Na Ayudhaya, W., Pityaratstian, N., & Jiamjarasrangsi, W. (2020). Effectiveness of behavioral activation in treating Thai older adults with subthreshold depression residing in the community. Clinical Interventions in Aging, 2363-2374.	Outcome measure(s) outside protocol<br>*Thai-GDS & DASS*
96	Sakata 2022	Sakata, M., Toyomoto, R., Yoshida, K., Luo, Y., Nakagami, Y., Uwatoko, T., ... & Furukawa, T. A. (2022). Components of smartphone cognitive-behavioural therapy for subthreshold depression among 1093 university students: a factorial trial. BMJ Ment Health, 25(e1), e18-e25.	Study design: Dismantling study<br>*Factorial design with all participants receiving CCBT with different components*
97	Samaan 2021	Samaan, M., Diefenbacher, A., Schade, C., Dambacher, C., Pontow, I. M., Pakenham, K., & Fydrich, T. (2021). A clinical effectiveness trial comparing ACT and CBT for inpatients with depressive and mixed mental disorders. Psychotherapy Research, 31(3), 372-385.	Population: <80% first-line treatment<br>*28% current psychotherapy at baseline*
98	Savari 2021	Savari, Y., Mohagheghi, H., & Petrocchi, N. (2021). A preliminary investigation on the effectiveness of compassionate mind training for students with major depressive disorder: A randomized controlled trial. Mindfulness, 12, 1159-1172.	Intervention: Psychological intervention not of interest<br>*Compassionate mind training group*

	Study ID	Reference	Reason for exclusion
99	Scazufca 2022	Scazufca, M., Nakamura, C. A., Seward, N., Moreno-Agostino, D., Van De Ven, P., Hollingworth, W., ... & Araya, R. (2022). A task-shared, collaborative care psychosocial intervention for improving depressive symptomatology among older adults in a socioeconomically deprived area of Brazil (PROACTIVE): a pragmatic, two-arm, parallel-group, cluster-randomised controlled trial. The Lancet Healthy Longevity, 3(10), e690-e702.	Data cannot be extracted<br>*No endpoint data, 17-week intervention but first assessment at 8-months after baseline*
100	Schaich 2023	Schaich, A., Outzen, J., Assmann, N., Gebauer, C., Jauch-Chara, K., Alvarez-Fischer, D., ... & Fassbinder, E. (2023). The effectiveness of metacognitive therapy compared to behavioral activation for severely depressed outpatients: a single-center randomized trial. Psychotherapy and Psychosomatics, 92(1), 38-48.	Population: >20% have coexisting personality disorder<br>*57% have personality disorder*
101	Schneider 2024	Schneider, B. C., Veckenstedt, R., Karamatskos, E., Ahlf-Schumacher, J., Gehlenborg, J., Schultz, J., ... & Jelinek, L. (2024). Efficacy and moderators of metacognitive training for depression in older adults (MCT-Silver): A randomized controlled trial. Journal of Affective Disorders, 345, 320-334.	Population: <80% non-chronic depression<br>*49% dysthymia*
102	Schwarzkopf 2021	Schwarzkopf, L., Dorscht, L., Kraus, L., & Luttenberger, K. (2021). Is bouldering-psychotherapy a cost-effective way to treat depression when compared to group cognitive behavioral therapy–results from a randomized controlled trial. BMC Health Services Research, 21, 1-13.	Population: <80% first-line treatment<br>*Same RCT as Kratzer 2021*
103	Sirey 2021	Sirey, J. A., Solomonov, N., Guillod, A., Zanotti, P., Lee, J., Soliman, M., & Alexopoulos, G. S. (2021). PROTECT: a novel psychotherapy for late-life depression in elder abuse victims. International psychogeriatrics, 33(5), 521-525.	Intervention: Psychological intervention not of interest<br>*Intervention focused on abuse*
104	Six 2022	Six, S. G., Byrne, K. A., Aly, H., & Harris, M. W. (2022). The effect of mental health app customization on depressive symptoms in college students: randomized controlled trial. JMIR Mental Health, 9(8), e39516.	Study design: Dismantling study<br>*Effects of avatar customisation*
105	Song 2023	Song, J., Litvin, B., Allred, R., Chen, S., Hull, T. D., & Areán, P. A. (2023). Comparing message-based psychotherapy to once-weekly, video-based psychotherapy for moderate depression: randomized controlled trial. Journal of Medical Internet Research, 25, e46052.	Comparison not relevant<br>*Details of specfic therapies provided not reported, comparison is concerned with format*
106	Strauss 2023	Strauss, C., Bibby-Jones, A. M., Jones, F., Byford, S., Heslin, M., Parry, G., ... & Cavanagh, K. (2023). Clinical effectiveness and cost-effectiveness of supported mindfulness-based cognitive therapy self-help compared with supported cognitive behavioral therapy self-help for adults experiencing depression: the Low-Intensity Guided Help Through Mindfulness (LIGHTMind) randomized clinical trial. JAMA psychiatry, 80(5), 415-424.	Population: <80% first-line treatment<br>*49% using psychotropic medication*
107	Stuart 2022	Stuart, R., Fischer, H., Leitzke, A. S., Becker, D., Saheba, N., & Coleman, K. J. (2022). The effectiveness of internet-based cognitive behavioral therapy for the treatment of depression in a large real-world primary care practice: a randomized trial. The Permanente Journal, 26(3), 53.	Population: <80% first-line treatment<br>*74% taking antidepressants at baseline*
108	Swartz 2023	Swartz, H. A., Bylsma, L. M., Fournier, J. C., Girard, J. M., Spotts, C., Cohn, J. F., & Morency, L. P. (2023). Randomized trial of brief interpersonal psychotherapy and cognitive behavioral therapy for depression delivered both in-person and by telehealth. Journal of Affective Disorders, 333, 543-552.	Population: <80% first-line treatment<br>*48% taking antidepressants at baseline*
109	Sweet 2021	Sweet, A. M., Pearlstein, S. L., Paulus, M. P., Stein, M. B., & Taylor, C. T. (2021). Computer-delivered behavioural activation and approach-avoidance training in major depression: Proof of concept and initial outcomes. British Journal of Clinical Psychology, 60(3), 357-374.	Data cannot be extracted<br>*Results not reported by arm*
110	Tan 2023	Tan, S., Ismail, M. A. B., Daud, T. I. M., Hod, R., & Ahmad, N. (2023). A randomized controlled trial on the effect of smartphone-based mental health application among outpatients with depressive and anxiety symptoms: A pilot study in Malaysia. Indian Journal of Psychiatry, 65(9), 934-940.	Population: Further-line treatment<br>*'All participants recruited were already on treatment by the psychiatrist-in-charge'*

	Study ID	Reference	Reason for exclusion
111	Taylor 2023	Taylor, R. W., Male, R., Economides, M., Bolton, H., & Cavanagh, K. (2023). Feasibility and Preliminary Efficacy of Digital Interventions for Depressive Symptoms in Working Adults: Multiarm Randomized Controlled Trial. JMIR Formative Research, 7, e41590.	Population: Not depression<br>*Below sub-threshold level (PHQ-8=8.7)*
112	Thielecke 2022	Thielecke, J., Buntrock, C., Titzler, I., Braun, L., Freund, J., Berking, M., ... & Ebert, D. D. (2022). Telephone coaching for the prevention of depression in farmers: Results from a pragmatic randomized controlled trial. Journal of Telemedicine and Telecare, 1357633X221106027.	Intervention: Psychological intervention not of interest<br>*No fixed procedures or standardised manuals were applied in the coaching. Coaching methods varied depending on the coach's background*
113	Ting 2022	Ting, X. (2022). Clinical Evaluation on Therapeutic Effect of Acupuncture Combined with Paroxetine on Patients with Anxiety and Depression. International Journal of Clinical Acupuncture, 31(4).	Paper unavailable
114	Uebelacker 2023	Uebelacker, L. A., Epstein-Lubow, G., Sillice, M. A., O'Keeffe, B., Kraines, M., Battle, C. L., ... & Abrantes, A. M. (2023). Project MOVE: A randomized controlled trial of interventions for initiating and maintaining physical activity in depressed individuals. Mental health and physical activity, 24, 100508.	Outcome(s) not of interest<br>*Depression outcome not reported*
115	Veloz 2022	Veloz, A. P. M., Padilla, C. P., & Villa, M. F. V. (2022). Effectiveness of physical exercise in the elderly with depression. NeuroQuantology, 20(13), 183.	Outcome measure(s) outside protocol<br>*Only remission reported*
116	Verhoeven 2023	Verhoeven, J. E., Han, L. K., Lever-van Milligen, B. A., Hu, M. X., Révész, D., Hoogendoorn, A. W., ... & Penninx, B. W. (2023). Antidepressants or running therapy: Comparing effects on mental and physical health in patients with depression and anxiety disorders. Journal of affective disorders, 329, 19-29.	Sample size N<10 per arm<br>*Partially randomised patient preference design and N<10 randomised in antidepressant arm*
117	Victor-Aigbodion 2023	Victor-Aigbodion, V., Eseadi, C., Ardi, Z., Sewagegn, A. A., Ololo, K., Abonor, L. B., ... & Effanga, O. A. (2023). Effectiveness of rational emotive behavior therapy in reducing depression among undergraduate medical students. Medicine, 102(4), e32724.	Outcome measure(s) outside protocol<br>*Zung Depression Inventory (ZDI)*
118	Vollbehr 2022	Vollbehr, N. K., Hoenders, H. J., Bartels-Velthuis, A. A., Nauta, M. H., Castelein, S., Schroevers, M. J., ... & Ostafin, B. D. (2022). Mindful yoga intervention as add-on to treatment as usual for young women with major depressive disorder: Results from a randomized controlled trial. Journal of Consulting and Clinical Psychology, 90(12), 925.	Population: <80% first-line treatment<br>*Participants in ongoling treatment at point of randomisation*
119	Wang 2021b	Wang, H., Liu, X. R., Wu, X. J., He, T. Z., Miao, D., Jiang, J. F., ... & Sun, Z. L. (2021). Additional value of auricular intradermal acupuncture alongside selective serotonin reuptake inhibitors: a single-blinded, randomized, sham-controlled preliminary clinical study. Acupuncture in Medicine, 39(6), 596-602.	Population: <80% first-line treatment<br>*Unclear how many participants already receiving SSRI at baseline and for whom acupuncture is further-line*
120	Wang 2021c	Wang, Y., Fu, C., Liu, Y., Li, D., Wang, C., Sun, R., & Song, Y. (2021). A study on the effects of mindfulness-based cognitive therapy and loving-kindness mediation on depression, rumination, mindfulness level and quality of life in depressed patients. American journal of translational research, 13(5), 4666.	Data cannot be extracted<br>*HAMD results only in figure*
121	Wang 2022b	Wang, J., & Li, Z. (2022). Effect of physical exercise on medical rehabilitation treatment of depression. Revista Brasileira de Medicina do Esporte, 28, 174-176.	Data cannot be extracted<br>*Details of intervention unclear. Paper does not report if exercise is individual or in groups and if it is supervised or not. Exercise combined with*

	Study ID	Reference	Reason for exclusion
			'Biyoujie 20mg' antidepressant but unclear what this is
122	Wang 2022c	Wang, Y., Luo, B., Wu, X., Li, X., & Liao, S. (2022). Comparison of the effects of Tai Chi and general aerobic exercise on weight, blood pressure and glycemic control among older persons with depressive symptoms: a randomized trial. BMC geriatrics, 22(1), 401.	Outcome(s) not of interest<br>*No depression outcome reported*
123	Wang 2023	Wang, C., Wang, C., Wang, J., Yu, N. X., Tang, Y., Liu, Z., & Chen, T. (2023). Effectiveness of solution-focused group counseling on depression and cognition among Chinese older adults: A cluster randomized controlled trial. Research on Social Work Practice, 33(5), 530-543.	Population: Not depression<br>*Below subthreshold depression (CES-D=8.42)*
124	Ward 2021	Ward, E. C., Brown, R. L., Sullivan-Wade, L., & Sainvilmar, S. (2021). A culturally adapted Depression intervention for african american adults: an efficacy trial. WMJ: official publication of the State Medical Society of Wisconsin, 120(4), 273.	Data cannot be extracted
125	Watkins 2023	Watkins, E., Newbold, A., Tester-Jones, M., Collins, L. M., & Mostazir, M. (2023). Investigation of active ingredients within internet-delivered cognitive behavioral therapy for depression: a randomized optimization trial. JAMA psychiatry, 80(9), 942-951.	Study design: Dismantling study<br>*32-condition fractional factorial optimisation experiment*
126	Watkins-Martin 2022	Watkins-Martin, K., Bolanis, D., Richard-Devantoy, S., Pennestri, M. H., Malboeuf-Hurtubise, C., Philippe, F., ... & Geoffroy, M. C. (2022). The effects of walking in nature on negative and positive affect in adult psychiatric outpatients with major depressive disorder: A randomized-controlled study. Journal of Affective Disorders, 318, 291-298.	Population: <80% first-line treatment<br>*34% receiving current psychiatric medication at baseline*
127	Weller 2022	Weller, S., Schroeder, P. A., & Plewnia, C. (2022). Gamification improves antidepressant effects of cognitive control training—A pilot trial. Frontiers in Digital Health, 4, 994484.	Population: <80% first-line treatment<br>*44% medication and 47% psychotherapy*
128	Williams 2022	Williams, C., McClay, C. A., Martinez, R., Morrison, J., Haig, C., Jones, R., & Farrand, P. (2022). Online Cognitive Behavioral Therapy (CBT) Life Skills Program for Depression: Pilot Randomized Controlled Trial. JMIR Formative Research, 6(2), e30489.	Population: <80% first-line treatment<br>*28% antidepressant use at baseline*
129	Wong 2021	Wong, Y. K., Wu, J. M., Zhou, G., Zhu, F., Zhang, Q., Yang, X. J., ... & Zhang, Z. J. (2021). Antidepressant monotherapy and combination therapy with acupuncture in depressed patients: a resting-state functional near-infrared spectroscopy (fNIRS) study. Neurotherapeutics, 18(4), 2651-2663.	Population: <80% first-line treatment<br>*Inclusion criteria included regular intake of antidepressants at a fixed dose for at least 3 months before baseline and during the study period*
130	Wright 2022	Wright, J. H., Owen, J., Eells, T. D., Antle, B., Bishop, L. B., Girdler, R., ... & Ali, S. (2022). Effect of computer-assisted cognitive behavior therapy vs usual care on depression among adults in primary care: a randomized clinical trial. JAMA Network Open, 5(2), e2146716-e2146716.	Population: <80% first-line treatment<br>*24% current antidepressant use at baseline*
131	Wu 2023	Wu, J., Song, J., He, Y., Li, Z., Deng, H., Huang, Z., ... & Chan, C. C. (2023). Effect of Tai Chi on Young Adults with Subthreshold Depression via a Stress–Reward Complex: A Randomized Controlled Trial. Sports Medicine-Open, 9(1), 90.	Data cannot be extracted<br>*Data reported as medians and IQRs*
132	Xu 2023	Xu, L., Wang, F., Yuan, J., Wu, Y., Wang, X., Meng, J. Y., ... & Yang, J. Z. (2023). A randomized controlled trial of mindfulness-based cognitive therapy (MBCT) for major depressive disorder in undergraduate students in China: The efficacy, serum proinflammatory cytokines and brain-derived neurotrophic factor. Asian journal of psychiatry, 88, 103718.	Study design: Letter to the editor<br>*Supplement appendix provides further details of trial but unclear if peer-reviewed and protocol reported is for single-arm trial*

	Study ID	Reference	Reason for exclusion
133	Yu 2023	Yu, D. J., Yu, A. P., Leung, C. K., Chin, E. C., Fong, D. Y., Cheng, C. P., ... & Siu, P. M. (2023). Comparison of moderate and vigorous walking exercise on reducing depression in middle-aged and older adults: A pilot randomized controlled trial. European journal of sport science, 23(6), 1018-1027.	Data cannot be extracted
134	Zhao 2022	Zhao, C., Wampold, B. E., Ren, Z., Zhang, L., & Jiang, G. (2022). The efficacy and optimal matching of an Internet-based acceptance and commitment therapy intervention for depressive symptoms among university students: A randomized controlled trial in China. Journal of Clinical Psychology, 78(7), 1354-1375.	Design: Non-RCT
135	Zhao 2023	Zhao, Y., Wang, W., Wang, M., Gao, F., Hu, C., Cui, B., ... & Ren, H. (2023). Personalized individual-based exercise prescriptions are effective in treating depressive symptoms of college students during the COVID-19: a randomized controlled trial in China. Frontiers in psychiatry, 13, 1015725.	Outcome measure(s) outside protocol<br>*Zung self-rating scale*
136	Ziapour 2023	Ziapour, A., Hajiazizi, A., Ahmadi, M., & Dehghan, F. (2023). Effect of short-term dynamic psychotherapy on sexual function and marital satisfaction in women with depression: Clinical trial study. Health Science Reports, 6(6), e1370.	Design: Non-RCT
137	Liu 2022	Liu, H., Peng, H., Song, X., Xu, C., & Zhang, M. (2022). Using AI chatbots to provide self-help depression interventions for university students: A randomized trial of effectiveness. Internet Interventions, 27, 100495.	Data could not be synthesised for the SMD outcome: baseline data were for the whole sample, but endpoint data were available for completers only
138	Dawood 2023	Dawood, S., Mir, G., & West, R. M. (2023). Randomized control trial of a culturally adapted behavioral activation therapy for Muslim patients with depression in Pakistan. World Journal of Psychiatry, 13(8), 551.	Data could not be synthesised for the SMD outcome: baseline data were for the whole sample, but endpoint data were available for completers only
139	Lindegaard 2021	Lindegaard, T., Seaton, F., Halaj, A., Berg, M., Kashoush, F., Barchini, R., ... & Andersson, G. (2021). Internet-based cognitive behavioural therapy for depression and anxiety among Arabic-speaking individuals in Sweden: a pilot randomized controlled trial. Cognitive behaviour therapy, 50(1), 47-66.	Data could not be synthesised for the SMD outcome: baseline data were for the whole sample, but endpoint data were available for completers only

Updated SMD networks, treatment classes, interventions and numbers of participants tested

Less severe depression

Network - treatment class level

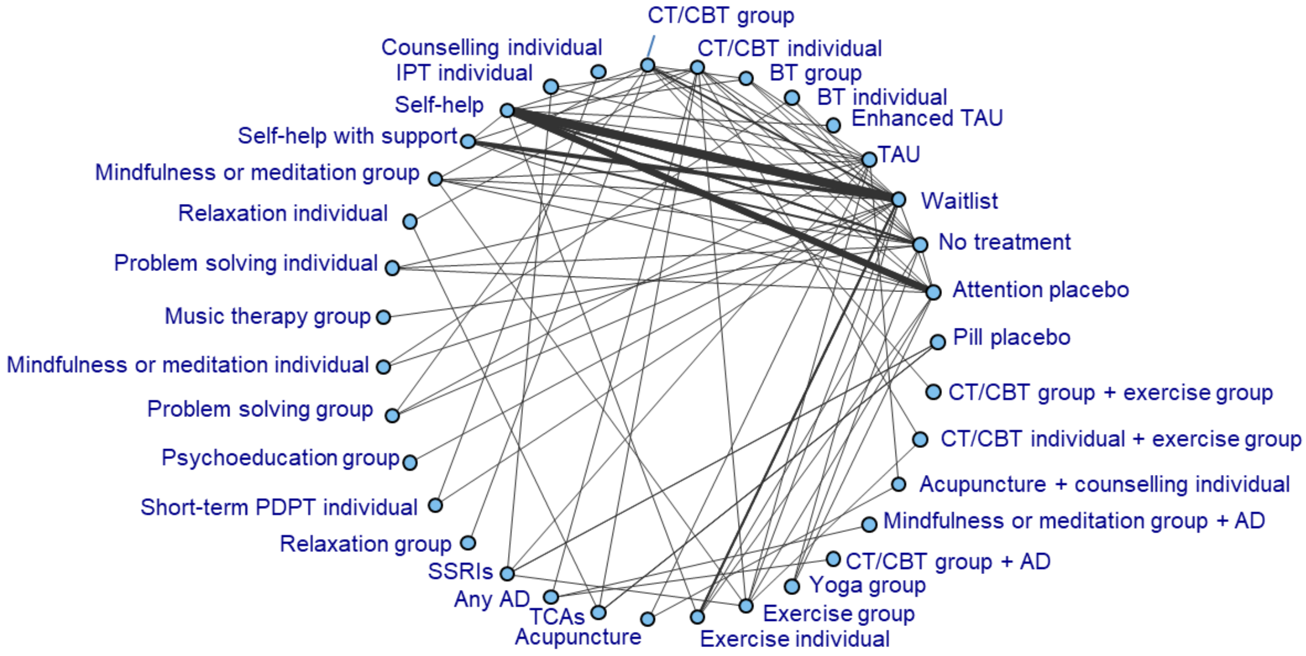

AD: antidepressant; BT: behavioural therapies; CBT: cognitive behavioural therapy; CT: cognitive therapy; IPT: interpersonal psychotherapy; PDPT: psychodynamic psychotherapy; SSRIs: selective serotonin uptake inhibitors; TAU: treatment as usual; TCAs: tricyclic antidepressants

Network - intervention level

1	Mindfulness meditation group
2	Computerised third-wave cognitive therapy with support
3	Computerised CBT (CCBT)
4	Computerised positive psychological intervention
5	Computerised expressive writing
6	Computerised behavioural activation
7	Computerised attentional bias modification
8	Computerised Coping with Depression course
9	Cognitive bibliotherapy
10	Lofepramine
11	Imipramine
12	Fluoxetine
13	Citalopram
14	Amitriptyline
15	Any AD
16	Sertraline
17	Progressive muscle relaxation group
18	Meditation-relaxation group

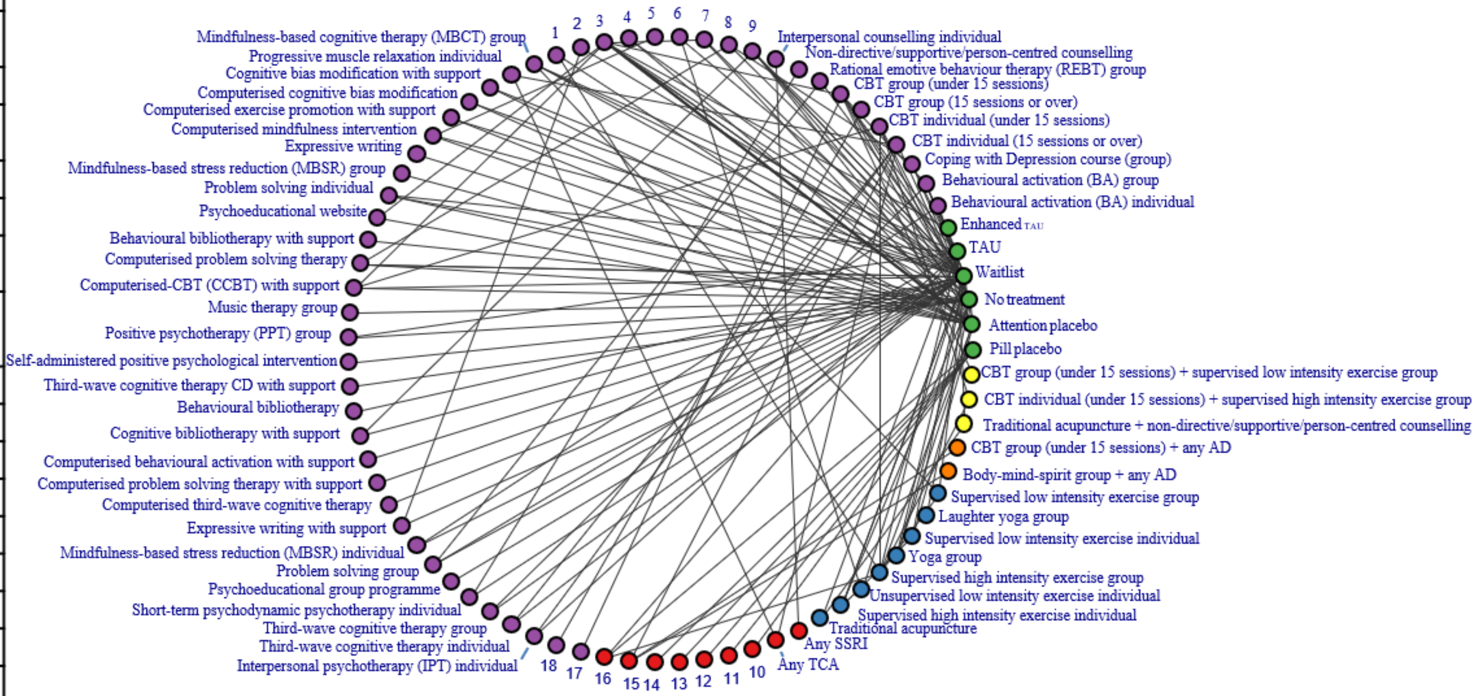

Classes, interventions and numbers of participants tested on each

The updated NMA following the 2023 search included 143 RCTs (16 additional RCTs compared with the original analysis), 79 interventions (3 additional interventions compared with the original analysis) grouped in 35 treatment classes (1 additional treatment class compared with the original analysis), and 19,118 participants (up from 16,829 participants in the original analysis). Of the 143 RCTs, 12 (up from 10 in the original analysis) reported change from baseline (CFB) depression symptom score data; 129 (up from 115 in the original analysis) reported baseline and endpoint depression symptom score data; and 2 (as in the original analysis) reported dichotomous response data and baseline symptom scores.

Treatment class	N	Intervention	N	Variance Sharing*
Waitlist	3785	Waitlist	3785	
Placebo	301	Pill placebo	301	
Attention placebo	1146	Attention placebo	1146	
No treatment	1863	No treatment	1863	
TAU	895	TAU	895	
Enhanced TAU	36	Enhanced TAU	36	
Self-help	5,334	Behavioural bibliotherapy	13	3
		Cognitive bibliotherapy	516	
		Computerised-CBT (CCBT)	2824	
		Computerised attentional bias modification	230	
		Computerised behavioural activation	122	
		Computerised cognitive bias modification	75	
		Computerised Coping with Depression course	257	
		Computerised expressive writing	36	
		Computerised mindfulness intervention	174	
		Computerised positive psychological intervention	439	
		Computerised problem solving therapy	272	
		Computerised third-wave CT	31	
		Excpressive writing	13	
		Psychoeducational website	204	
		Self-administered positive psychological intervention	128	
Self-help with support	1721	Behavioural bibliotherapy with support	67	4
		Cognitive bibliotherapy with support	125	
		Computerised-CBT (CCBT) with support	604	
		Computerised behavioural activation with support	107	
		Computerised exercise promotion with support	137	
		Computerised problem solving therapy with support	171	
		Computerised third-wave CT with support	283	
		Cognitive bias modification with support	20	
		Computerised exercise promotion with support	137	
		Expressive writing with support	125	
Behavioural therapies individual	147	Behavioural activation (BA) individual	147	1
Behavioural therapies group	410	Behavioural activation (BA) group	187	1
		Coping with Depression course (group)	223	
CT/CBT individual	519	CBT individual (15 sessions or over)	123	1
		CBT individual (under 15 sessions)	271	
		Third-wave CT individual	125	

CT/CBT group	719	CBT group (15 sessions or over)	10	2
		CBT group (under 15 sessions)	555	
		Positive psychotherapy (PPT) group	76	
		Rational emotive behaviour therapy (REBT) group	14	
		Third-wave CT group	64	
Problem solving individual	98	Problem solving individual	98	1
Problem solving group	104	Problem solving group	104	1
Counselling individual	55	Non-directive/supportive/person-centred counselling	55	1
IPT individual	153	IPT individual	136	1
		Interpersonal counselling individual	17	
Short-term PDPT individual	49	Short-term PDPT individual	49	1
Psychoeducation group	22	Psychoeducational group programme	22	1
Mindfulness or meditation individual	20	Mindfulness-based stress reduction (MBSR) individual	20	1
Mindfulness or meditation group	404	Meditation-relaxation group	13	5
		MBCT group	177	
		Mindfulness-based stress reduction (MBSR) group	85	
		Mindfulness meditation group	129	
Relaxation individual	13	Progressive muscle relaxation individual	13	1
Relaxation group	63	Progressive muscle relaxation group	63	2
SSRIs	207	Any SSRI	24	6
		Fluoxetine	78	
		Citalopram	24	
		Sertraline	81	
TCAs	136	Any TCA	10	7
		Amitriptyline	67	
		Lofepramine	23	
		Imipramine	36	
Any AD	65	Any AD	65	8
Acupuncture	40	Traditional acupuncture	40	1
Exercise individual	316	Supervised high intensity exercise individual	78	9
		Supervised low intensity exercise individual	117	
		Unsupervised low intensity exercise individual	121	
Exercise group	228	Supervised high intensity exercise group	147	4
		Supervised low intensity exercise group	81	
Yoga group	103	Yoga group	73	2
		Laughter yoga group	30	
CT/CBT group + AD	32	CBT group (under 15 sessions) + any AD	32	1
Mindfulness or meditation group + AD	15	Body-mind-spirit group + any AD	15	1
Acupuncture + counselling individual	40	Traditional acupuncture + non-directive/supportive/person-centred counselling	40	1
CT/CBT individual + exercise group	18	CBT individual (under 15 sessions) + supervised high intensity exercise group	18	1
CT/CBT group + exercise group	25	CBT group (under 15 sessions) + supervised low intensity exercise group	25	1

AD: antidepressant; CBT: cognitive behavioural therapy; CT: cognitive therapy; IPT: interpersonal psychotherapy; MBCT: mindfulness-based cognitive therapy; PDPT: psychodynamic psychotherapy; SSRIs: selective serotonin uptake inhibitors; TAU: treatment as usual; TCAs: tricyclic antidepressants

** Classes with the same number share a common class variance*

More severe depression

Network - treatment class level

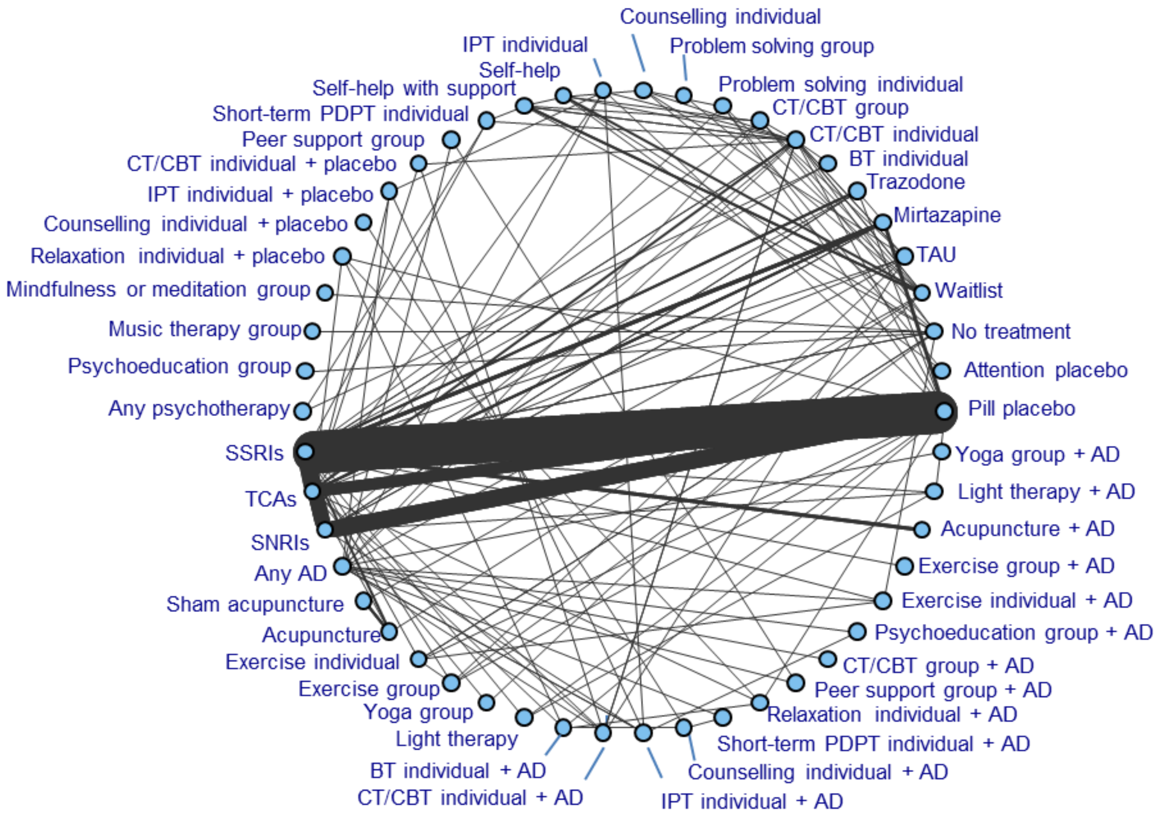

AD: antidepressant; BT: behavioural therapies; CBT: cognitive behavioural therapy; CT: cognitive therapy; IPT: interpersonal psychotherapy; PDPT: psychodynamic psychotherapy; SNRIs: serotonin and norepinephrine reuptake inhibitors; SSRIs: selective serotonin uptake inhibitors; TAU: treatment as usual; TCAs: tricyclic antidepressants

Network - intervention level

1	Computerised-CBT (CCBT) with support
2	Computerised cognitive bias modification
3	Computerised attentional bias modification
4	Progressive muscle relaxation individual + pill placebo
5	Interpersonal psychotherapy (IPT) individual + pill placebo
6	CBT individual (15 sessions or over) + pill placebo
7	CBT individual (15 sessions or over) + pill placebo
8	Peer support group
9	Non-directive/supportive/person-centred counselling + pill placebo
10	Short-term psychodynamic psychotherapy individual
11	Mindfulness meditation CD with support
12	Cognitive bibliotherapy with support
13	Interpersonal counselling individual + venlafaxine
14	Non-directive/supportive/person-centred counselling + fluoxetine
15	Peer support group + any AD
16	CBT individual (under 15 sessions) + escitalopram
17	CBT individual (under 15 sessions) + sertraline
18	CBT individual (15 sessions or over) + imipramine
19	CBT individual (15 sessions or over) + nortriptyline
20	CBT individual (15 sessions or over) + any SSRI
21	Progressive muscle relaxation individual + amitriptyline
22	Short-term psychodynamic psychotherapy individual + any AD
23	Non-directive/supportive/person-centred counselling + any SSRI

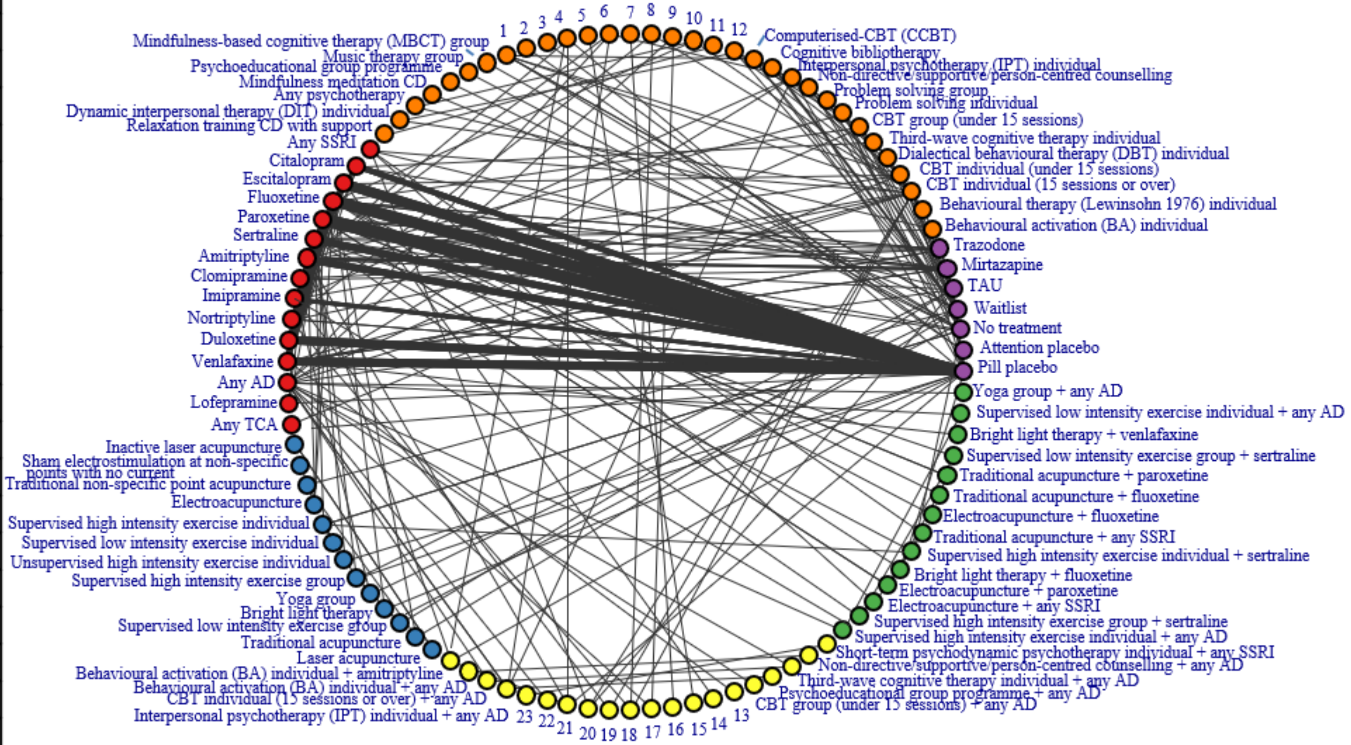

Classes, interventions and numbers of participants tested on each

The updated NMA following the 2023 search included 361 RCTs (9 additional RCTs compared with the original analysis), 101 interventions (2 additional interventions compared with the original analysis) grouped in 50 treatment classes (as in the original analysis), and 61,103 participants (up from 59,350 participants in the original analysis). Of the 361 RCTs, 149 (up from 146 in the original analysis) reported change from baseline (CFB) depression symptom score data; 178 (up from 172 in the original analysis) reported baseline and endpoint depression symptom score data; and 34 (as in the original analysis) reported dichotomous response data and baseline symptom scores. One study (Giosan 2020) identified from the update search was eligible for inclusion in the review but its data were not possible to include in the NMA, due to severe convergence issues caused by the fact that the study compared the same intervention in both arms, but effects were very different between arms, leading to considerable heterogeneity compared with other relevant evidence in the dataset.

Treatment class	N	Intervention	N	Variance Sharing*
Pill placebo	12,684	Pill placebo	12,684	
Attention placebo	87	Attention placebo	87	
No treatment	504	No treatment	504	
Waitlist	626	Waitlist	626	
TAU	220	TAU	220	
Sham acupuncture	124	Inactive laser acupuncture	34	1
		Sham electrostimulation at non-specific points with no current	38	
		Traditional non-specific point acupuncture	52	
Self-help without or with minimal support	523	Cognitive bibliotherapy	159	2
		Computerised-CBT (CCBT)	270	
		Computerised attentional bias modification	26	
		Computerised cognitive bias modification	29	
		Mindfulness meditation CD	39	
Self-help with support	418	Cognitive bibliotherapy with support	66	3
		Computerised-CBT (CCBT) with support	315	
		Mindfulness meditation CD with support	19	
		Relaxation training CD with support	18	
Behavioural therapies individual	378	Behavioural activation (BA) individual	368	4
		Behavioural therapy (Lewinsohn 1976) individual	10	
CT/CBT individual	1,044	CBT individual (15 sessions or over)	626	4
		CBT individual (under 15 sessions)	369	
		Dialectical behavioural therapy (DBT) individual	10	
		Third-wave CT individual	39	
CT/CBT group	165	CBT group (under 15 sessions)	165	4
Problem solving individual	367	Problem solving individual	367	4
Problem solving group	47	Problem solving group	47	4
Counselling individual	404	Non-directive/supportive/person-centred counselling	404	4
IPT individual	146	IPT individual	146	4

Short-term PDPT individual	233	Dynamic interpersonal therapy (DIT) individual	73	4
		Short-term PDPT individual	160	
Psychoeducation group	44	Psychoeducational group programme	44	4
Music therapy group	12	Music therapy group	12	4
Mindfulness or meditation group	15	MBCT group	15	4
Peer support group	39	Peer support group	39	4
Any psychotherapy	37	Any psychotherapy	37	4
CT/CBT individual + pill placebo	61	CBT individual (15 sessions or over) + pill placebo	17	4
		CBT individual (under 15 sessions) + pill placebo	44	
IPT + pill placebo	69	IPT individual + pill placebo	69	4
Counselling individual + pill placebo	26	Non-directive/supportive/person-centred counselling + pill placebo	26	4
Relaxation individual + pill placebo	11	Progressive muscle relaxation individual + pill placebo	11	4
SSRIs	22,675	Any SSRI	207	5
		Citalopram	2,195	
		Escitalopram	5,456	
		Fluoxetine	6,162	
		Paroxetine	5,861	
		Sertraline	2,794	
TCAs	4,816	Amitriptyline	2,504	6
		Any TCA	21	
		Clomipramine	345	
		Imipramine	1,306	
		Lofepramine	145	
		Nortriptyline	495	
SNRIs	9,646	Duloxetine	5,269	5
		Venlafaxine	4,377	
Mirtazapine	1,884	Mirtazapine	1,884	
Trazodone	1,072	Trazodone	1,072	
Any AD	452	Any AD	452	7
Acupuncture	278	Electroacupuncture	124	8
		Laser acupuncture	39	
		Traditional acupuncture	115	
Exercise individual	298	Supervised high intensity exercise individual	128	9
		Supervised low intensity exercise individual	117	
		Unsupervised high intensity exercise individual	53	
Exercise group	106	Supervised high intensity exercise group	69	3
		Supervised low intensity exercise group	37	
Yoga group	65	Yoga group	65	4
Light therapy	32	Bright light therapy	32	8
Behavioural therapies individual + AD	22	Behavioural activation (BA) individual + amitriptyline	12	10
		Behavioural activation (BA) individual + any AD	10	
CT/CBT individual + AD	192	CBT individual (15 sessions or over) + any AD	10	10
		CBT individual (15 sessions or over) + any SSRI	43	
		CBT individual (15 sessions or over) + imipramine	25	
		CBT individual (15 sessions or over) + nortriptyline	18	

		CBT individual (under 15 sessions) + escitalopram	48	
		CBT individual (under 15 sessions) + sertraline	38	
		Third-wave CT individual + any AD	10	
CT/CBT group + AD	63	CBT group (under 15 sessions) + any AD	63	10
IPT individual + AD	99	IPT individual + any AD	87	10
		Interpersonal counselling individual + venlafaxine	12	
Counselling individual + AD	57	Non-directive/supportive/person-centred counselling + any AD	15	11
		Non-directive/supportive/person-centred counselling + any SSRI	17	
		Non-directive/supportive/person-centred counselling + fluoxetine	25	
Short-term PDPT individual + AD	131	Short-term PDPT individual + any AD	113	10
		Short-term PDPT individual + any SSRI	18	
Psychoeducation group + AD	27	Psychoeducational group programme + any AD	27	10
Peer support group + AD	42	Peer support group + any AD	42	10
Relaxation individual + AD	10	Progressive muscle relaxation individual + amitriptyline	10	10
Exercise individual + AD	40	Supervised high intensity exercise individual + any AD	14	12
		Supervised high intensity exercise individual + sertraline	15	
		Supervised low intensity exercise individual + any AD	11	
Exercise group + AD	79	Supervised high intensity exercise group + sertraline	42	10
		Supervised low intensity exercise group + sertraline	37	
Yoga group + AD	15	Yoga group + any AD	15	10
Acupuncture + AD	664	Electroacupuncture + any SSRI	160	13
		Electroacupuncture + fluoxetine	46	
		Electroacupuncture + paroxetine	71	
		Traditional acupuncture + any SSRI	206	
		Traditional acupuncture + fluoxetine	80	
		Traditional acupuncture + paroxetine	101	
Light therapy + AD	54	Bright light therapy + fluoxetine	29	8
		Bright light therapy + venlafaxine	25	

AD: antidepressant; CBT: cognitive behavioural therapy; CT: cognitive therapy; IPT: interpersonal psychotherapy; MBCT: mindfulness-based cognitive therapy; PDPT: psychodynamic psychotherapy; SNRIs: serotonin and norepinephrine reuptake inhibitors; SSRIs: selective serotonin uptake inhibitors; TAU: treatment as usual; TCAs: tricyclic antidepressants
** Classes with the same number share a common class variance*

Model fit statistics – original and updated bias-adjusted SMD analyses

Less severe depression

Model<br>RE – random class effect: bias adjustment	Between Study Heterogeneity - SD (95% CrI)	Posterior total residual deviance[a]	DIC[b]
2020 – original analysis	0·44 (0·27, 0·57)	283·9	1192
2023 – updated analysis	0·46 (0·31, 0·57)	319·4	1313

Abbreviations: CrI, credible interval; DIC, deviance information criteria; RE, random study-specific treatment effects; SD, standard deviation
a Posterior mean residual deviance compared to 279 (2020) and 315 (2023) total data points.
b Lower values of DIC preferred

More severe depression

Model<br>RE – random class effect: bias adjustment	Between Study Heterogeneity - SD (95% CrI)	Posterior total residual deviance[a]	DIC[b]
2020 – original analysis	0·20 (0·16, 0·24)	830·4	3255
2023 – updated analysis	0·30 (0·26, 0·33)	836·9	3392

Abbreviations: CrI, credible interval; DIC, deviance information criteria; RE, random study-specific treatment effects; SD, standard deviation
a Posterior mean residual deviance compared to 811 (2020) and 832 (2023) total data points
b Lower values of DIC preferred

Risk of bias of new studies included in the NMA (reviewer's judgements)

Less severe depression

	Random sequence generation (selection bias)	Allocation concealment (selection bias)	Blinding of participants and personnel (performance bias)	Blinding of outcome assessment (detection bias)	Incomplete outcome data (attrition bias)	Selective reporting (reporting bias)	Other bias
Armat 2022	+	?	–	?	+	+	+
Braun 2021	+	+	–	?	+	+	+
Brush 2022	+	?	–	?	+	?	+
Cody 2023	?	?	–	–	+	+	+
Demir 2022	+	?	–	?	+	+	+
He 2022	+	+	?	?	+	+	–
Kannampallil 2023	?	?	–	?	+	+	?
Kramer 2021	?	+	–	?	+	+	+
Lopez 2020b	?	?	–	?	+	?	+
MacLean 2020	–	+	–	?	+	+	+
Mathiasen 2022	–	+	–	?	+	+	+
Shih 2021	+	?	–	?	+	?	+
Vázquez 2023	–	+	–	?	+	+	+
Wu 2021	?	?	–	?	+	?	?
Ying 2023	+	+	–	?	–	?	+
Zhang 2022	?	?	–	?	?	?	+

More severe depression

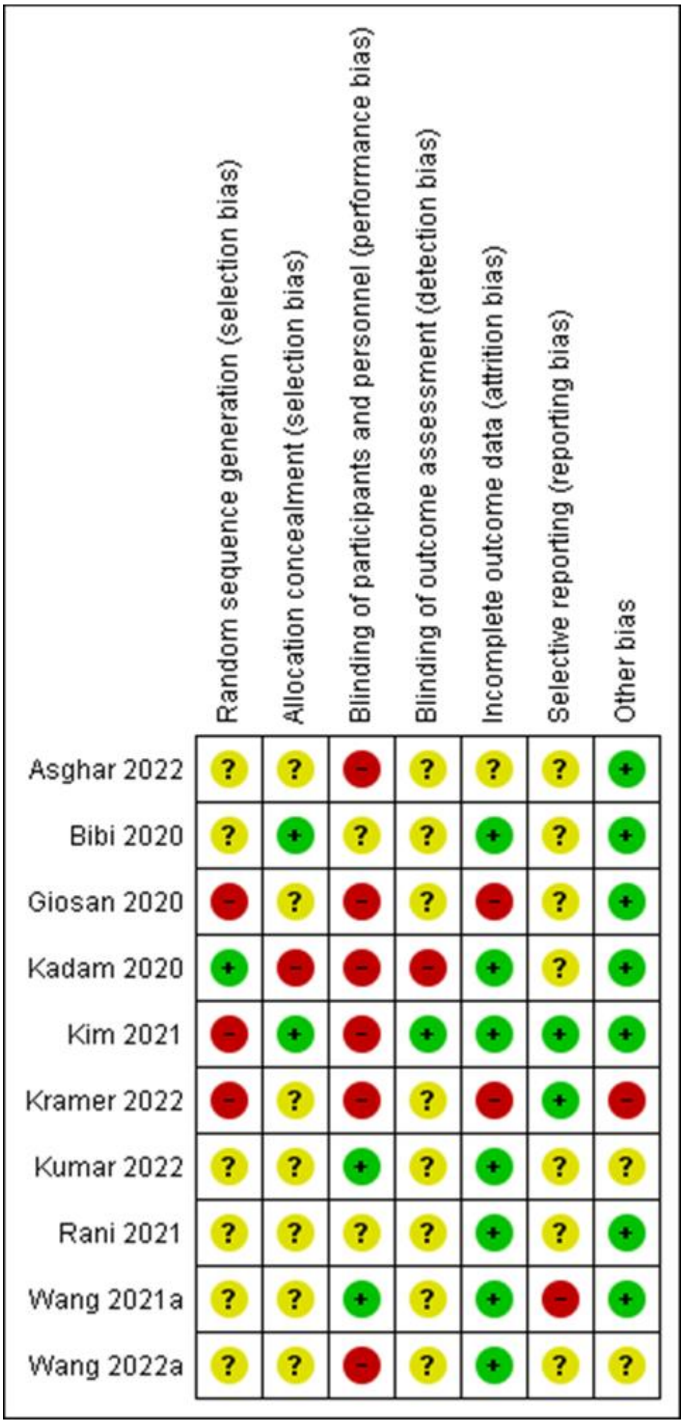

Updated bias-adjusted results: SMDs versus the reference treatment

Less severe depression

Treatment class level

Review update: Bias-adjusted results of the network meta-analysis of standardised mean difference (SMD) of depression scale change scores in adults with a new episode of less severe depression: posterior effects (mean SMD, 95%CrI) of all treatment classes versus treatment as usual (TAU) and treatment class rankings

Treatment class	N	SMD vs TAU (mean, 95% CrI)	Rank (mean, 95% CrI)
CT/CBT group + exercise group	25	**-2.40 (-4.19 to -0.65)**	3.33 (1 to 16)
Yoga group	103	**-1.78 (-3.34 to -0.41)**	5.62 (1 to 19)
Problem solving group	104	-1.45 (-3.03 to 0.12)	8.18 (1 to 27)
CT/CBT group	719	**-1.06 (-1.98 to -0.07)**	10.89 (3 to 25)
Mindfulness or meditation group + AD	15	-1.46 (-4.35 to 1.37)	10.97 (1 to 33)
CT/CBT group + AD	32	-1.19 (-3.84 to 1.46)	12.62 (1 to 33)
CT/CBT individual	519	-0.91 (-1.85 to 0.07)	12.76 (4 to 26)
Behavioural therapies group	410	-0.91 (-1.94 to 0.22)	12.88 (4 to 28)
Behavioural therapies individual	147	-0.90 (-2.47 to 0.73)	13.73 (2 to 31)
Mindfulness or meditation group	404	-0.81 (-1.96 to 0.25)	14.18 (3 to 29)
Mindfulness or meditation individual	20	-0.90 (-3.07 to 1.29)	14.53 (1 to 33)
Acupuncture + counselling individual	40	-0.84 (-2.54 to 0.84)	14.68 (2 to 32)
Short-term PDPT individual	49	-0.82 (-2.93 to 1.38)	15.14 (1 to 32)
Acupuncture	40	-0.77 (-2.47 to 0.95)	15.49 (2 to 32)
TCAs	136	-0.71 (-2.58 to 1.12)	16.39 (2 to 33)
SSRIs	207	-0.69 (-2.11 to 0.62)	16.41 (3 to 32)
Pill placebo	301	-0.77 (-2.08 to 0.47)	17.13 (4 to 31)
IPT individual	153	-0.61 (-1.92 to 0.60)	17.53 (4 to 31)
Relaxation individual	13	-0.54 (-2.79 to 1.75)	18.11 (2 to 33)
Self-help without/with minimal support	5334	**-0.50 (-0.94 to -0.07)**	18.51 (12 to 24)
Counselling individual	55	-0.44 (-2.78 to 2.05)	19.00 (2 to 33)
Exercise individual	316	-0.47 (-2.13 to 1.13)	19.13 (3 to 33)
Exercise group	228	-0.41 (-1.10 to 0.29)	20.35 (10 to 30)
Relaxation group	63	-0.32 (-2.38 to 1.70)	20.64 (2 to 33)
CT/CBT individual + exercise group	18	-0.30 (-2.36 to 1.76)	20.78 (3 to 33)
Psychoeducation group	22	-0.29 (-2.12 to 1.57)	21.04 (3 to 33)
Self-help with support	1721	-0.36 (-0.84 to 0.12)	21.14 (14 to 28)
Music therapy group	36	-0.24 (-2.28 to 1.80)	21.48 (3 to 33)
Attention placebo	1146	-0.27 (-0.76 to 0.20)	23.09 (16 to 29)
Enhanced TAU	36	-0.03 (-1.19 to 1.13)	24.75 (9 to 33)
Problem solving individual	98	0.04 (-1.49 to 1.59)	25.45 (7 to 33)
TAU	895	Reference	26.77 (20 to 32)
Waitlist	3785	0.12 (-0.32 to 0.56)	28.33 (23 to 32)

Treatment classes ordered from best to worst, according to mean ranking. Negative effect values indicate a favourable outcome for treatment classes compared with TAU. Results where 95% CrI do not cross the no effect line are shown in bold. AD: antidepressant; CBT: cognitive behavioural therapy; CrI: credible intervals; CT: cognitive therapy; IPT: interpersonal psychotherapy; PDPT: psychodynamic psychotherapy; SMD: standardised mean difference; SSRIs: selective serotonin uptake inhibitors; TAU: treatment as usual; TCAs: tricyclic antidepressants

Intervention level

Posterior effects (mean SMD, 95%CrI) of all interventions versus treatment as usual (TAU). Only interventions of interest belonging to classes with N ≥50 have been included in the table, plus short-term psychodynamic psychotherapy

Treatment class	N	SMD vs TAU (mean, 95% CrI)	Intervention	N	SMD vs TAU (mean, 95% CrI)
**Self-help without or with minimal support**	**5,334**	**-0.50 (-0.94 to -0.07)**	Behavioural bibliotherapy	13	-0.50 (-1.01 to 0.01)
			Cognitive bibliotherapy	516	-0.45 (-0.91 to 0.01)
			**Computerised Coping with Depression course**	**257**	**-0.51 (-1.01 to -0.04)**
			Computerised attentional bias modification	230	-0.47 (-0.95 to 0.01)
			**Computerised behavioural activation**	**122**	**-0.54 (-1.06 to -0.06)**
			Computerised cognitive bias modification	75	-0.48 (-0.97 to 0.02)
			Computerised expressive writing	36	-0.50 (-1.01 to 0.01)
			Computerised mindfulness intervention	174	-0.49 (-0.97 to 0.00)
			Computerised positive psychological intervention	439	-0.46 (-0.93 to 0.03)
			**Computerised problem solving therapy**	**272**	**-0.53 (-1.03 to -0.06)**
			**Computerised third-wave cognitive therapy**	**31**	**-0.51 (-1.03 to -0.01)**
			**Computerised-CBT (CCBT)**	**2824**	**-0.53 (-0.98 to -0.09)**
			**Expressive writing**	**13**	**-0.51 (-1.04 to 0.00)**
			**Psychoeducational website**	**204**	**-0.51 (-1.00 to -0.03)**
			**Self-administered positive psychological intervention**	**128**	**-0.57 (-1.14 to -0.07)**
Self-help with support	1,721	-0.36 (-0.84 to 0.12)	Behavioural bibliotherapy with support	67	-0.35 (-0.89 to 0.21)
			Cognitive bias modification with support	20	-0.40 (-0.97 to 0.14)
			Cognitive bibliotherapy with support	125	-0.40 (-0.95 to 0.13)
			Computerised behavioural activation with support	107	-0.40 (-0.95 to 0.13)
			Computerised exercise promotion with support	137	-0.35 (-0.89 to 0.19)
			Computerised problem solving therapy with support	171	-0.34 (-0.85 to 0.19)
			Computerised third-wave cognitive therapy with support	82	-0.38 (-0.94 to 0.15)
			Computerised-CBT (CCBT) with support	604	-0.34 (-0.83 to 0.16)
			Expressive writing with support	125	-0.35 (-0.87 to 0.19)
			Third-wave cognitive therapy CD with support	283	-0.39 (-0.95 to 0.14)
Behavioural therapies individual	147	-0.90 (-2.47 to 0.73)	**Behavioural activation (BA) individual**	**147**	**-0.89 (-1.76 to 0.00)**

Behavioural therapies group	410	-0.91 (-1.94 to 0.22)	**Behavioural activation (BA) group**	**187**	**-1.21 (-1.78 to -0.65)**
			Coping with Depression course (group)	223	-0.62 (-1.31 to 0.11)
CT/CBT individual	519	-0.91 (-1.85 to 0.07)	**CBT individual (15 sessions or over)**	**123**	**-0.90 (-1.61 to -0.16)**
			**CBT individual (under 15 sessions)**	**271**	**-0.94 (-1.62 to -0.26)**
			**Third-wave cognitive therapy individual**	**125**	**-0.90 (-1.63 to -0.15)**
**CT/CBT group**	**719**	**-1.06 (-1.98 to -0.07)**	CBT group (15 sessions or over)	10	-0.82 (-2.05 to 0.74)
			**CBT group (under 15 sessions)**	**555**	**-1.18 (-1.62 to -0.74)**
			**Positive psychotherapy (PPT) group**	**76**	**-0.92 (-1.57 to -0.22)**
			**Rational emotive behaviour therapy (REBT) group**	**14**	**-1.22 (-2.34 to -0.14)**
			**Third-wave cognitive therapy group**	**64**	**-1.20 (-1.98 to -0.44)**
Problem solving individual	98	0.04 (-1.49 to 1.59)	Problem solving individual	98	0.04 (-0.69 to 0.78)
Problem solving group	104	-1.45 (-3.03 to 0.12)	**Problem solving group**	**104**	**-1.42 (-2.25 to -0.59)**
Counselling individual	55	-0.44 (-2.78 to 2.05)	Non-directive/supportive/person-centred counselling	55	-0.46 (-2.35 to 1.46)
IPT individual	153	-0.61 (-1.92 to 0.60)	Interpersonal counselling individual	17	-0.68 (-2.09 to 0.57)
			Interpersonal psychotherapy (IPT) individual	136	-0.54 (-1.18 to 0.10)
Short-term PDPT individual	49	-0.82 (-2.93 to 1.38)	Short-term PDPT individual	49	-0.84 (-2.41 to 0.73)
Mindfulness or meditation group	404	-0.81 (-1.96 to 0.25)	Mindfulness-based stress reduction (MBSR) individual	20	-0.88 (-2.41 to 0.67)
			Meditation-relaxation group	13	-1.00 (-2.59 to 0.18)
			**Mindfulness meditation group**	**129**	**-0.84 (-1.6 to -0.10)**
			**Mindfulness-based cognitive therapy (MBCT) group**	**177**	**-0.87 (-1.45 to -0.3)**
			Mindfulness-based stress reduction (MBSR) group	85	-0.57(-1.31 to 0.31)
Relaxation group	63	-0.32 (-2.38 to 1.70)	Progressive muscle relaxation group	63	-0.32 (-1.46 to 0.82)
SSRIs	207	-0.69 (-2.11 to 0.62)	Citalopram	24	-0.66 (-2.00 to 0.61)
			Fluoxetine	78	-0.84 (-2.26 to 0.38)
			Sertraline	81	-0.68 (-1.69 to 0.28)
TCAs	136	-0.71 (-2.58 to 1.12)	Amitriptyline	67	-1.17 (-2.80 to 0.26)
			Imipramine	36	-0.81 (-2.37 to 0.63)
			Lofepramine	23	-0.63 (-2.12 to 0.82)
Exercise individual	316	-0.47 (-2.13 to 1.13)	Supervised high intensity exercise individual	78	-0.70 (-1.49 to 0.04)
			Supervised low intensity exercise individual	117	-0.42 (-1.16 to 0.32)
			Unsupervised low intensity exercise individual	121	-0.31 (-1.04 to 0.46)

Exercise group	228	-0.41 (-1.10 to 0.29)	Supervised high intensity exercise group	147	-0.40 (-1.06 to 0.25)
			Supervised low intensity exercise group	81	-0.37 (-1.04 to 0.30)
**Yoga group**	**103**	**-1.78 (-3.34 to -0.41)**	**Laughter yoga group**	**30**	**-2.30 (-3.63 to -1.09)**
			**Yoga group**	**73**	**-1.26 (-2.17 to -0.34)**

Negative effect values indicate a favourable outcome for treatment classes and interventions compared with TAU. Results where 95% CrI do not cross the no effect line are shown in bold.

CBT: cognitive behavioural therapy; CrI: credible intervals; CT: cognitive therapy; IPT: interpersonal psychotherapy; MBCT: mindfulness-based cognitive therapy; PDPT: psychodynamic psychotherapy; SMD: standardised mean difference; SSRIs: selective serotonin uptake inhibitors; TAU: treatment as usual; TCAs: tricyclic antidepressants

More severe depression

Treatment class level

Review update: bias-adjusted results of the network meta-analysis of standardised mean difference (SMD) of depression symptom change scores in adults with a new episode of more severe depression: posterior effects (mean SMD, 95%CrI) of all treatment classes versus pill placebo and treatment class rankings

Treatment class	N	SMD vs pill placebo (mean, 95% CrI)	Rank (mean, 95% CrI)
Mindfulness or meditation group	15	**-3.48 (-5.09 to -1.91)**	1.63 (1 to 5)
Problem solving group	47	**-2.14 (-3.64 to -0.71)**	4.85 (1 to 19)
Yoga group + AD	15	**-1.83 (-3.71 to -0.05)**	7.84 (1 to 35)
Exercise group + AD	79	**-1.41 (-2.70 to -0.12)**	10.22 (2 to 34)
Peer support group + AD	42	-1.41 (-3.11 to 0.20)	11.02 (2 to 38)
CT/CBT individual + AD	192	**-1.19 (-2.00 to -0.53)**	11.19 (4 to 23)
Peer support group	39	-1.29 (-2.77 to 0.19)	11.76 (2 to 38)
CT/CBT group + AD	63	-1.18 (-2.77 to 0.31)	13.30 (2 to 39)
Behavioural therapies individual	378	**-0.96 (-2.02 to -0.01)**	15.02 (4 to 36)
Psychoeducation group	44	-1.02 (-2.48 to 0.41)	15.25 (3 to 40)
Problem solving individual	367	-0.90 (-2.24 to 0.38)	16.38 (3 to 40)
CT/CBT individual	1044	**-0.85 (-1.76 to -0.28)**	16.65 (6 to 29)
Light therapy + AD	54	-0.91 (-2.54 to 0.72)	16.66 (3 to 41)
Yoga group	65	-0.89 (-2.38 to 0.54)	17.17 (3 to 41)
Self-help without/with minimal support	523	-0.79 (-1.84 to 0.16)	17.99 (5 to 38)
Exercise individual + AD	40	-0.89 (-3.60 to 1.71)	18.61 (1 to 43)
Acupuncture + AD	664	**-0.69 (-1.05 to -0.32)**	19.24 (10 to 29)
Short-term PDPT individual	233	-0.70 (-1.77 to 0.24)	20.20 (6 to 39)
IPT individual + AD	99	-0.71 (-1.92 to 0.43)	20.36 (5 to 40)
Light therapy	32	-0.69 (-2.97 to 1.54)	20.82 (2 to 43)
Self-help with support	418	-0.62 (-1.56 to 0.40)	21.21 (6 to 40)
Counselling individual	404	-0.51 (-1.83 to 0.77)	24.06 (5 to 42)
Interpersonal psychotherapy (IPT) individual	146	-0.43 (-1.75 to 0.89)	25.46 (6 to 42)
Short-term PDPT individual + AD	131	-0.41 (-2.23 to 1.34)	25.58 (4 to 43)
Relaxation individual + AD	10	-0.35 (-2.57 to 1.93)	25.74 (3 to 43)
Acupuncture	278	-0.44 (-2.01 to 0.84)	25.87 (4 to 42)
Psychoeducation group + AD	27	-0.36 (-2.00 to 1.20)	26.69 (5 to 43)
SNRIs	9646	**-0.37 (-0.55 to -0.19)**	27.03 (19 to 35)
Sham acupuncture	124	-0.27 (-2.61 to 2.25)	27.18 (3 to 43)
Mirtazapine	1884	**-0.35 (-0.52 to -0.19)**	27.62 (20 to 35)
Music therapy group	12	-0.25 (-1.96 to 1.42)	28.02 (5 to 43)
CT/CBT group	165	-0.31 (-1.65 to 0.97)	28.21 (7 to 42)
TCAs	4816	**-0.33 (-0.51 to -0.14)**	28.36 (21 to 36)
Behavioural therapies individual + AD	22	-0.10 (-2.43 to 2.34)	28.54 (3 to 43)
Exercise group	106	-0.27 (-1.23 to 0.74)	29.04 (10 to 42)
TAU	220	-0.29 (-0.69 to 0.11)	29.51 (19 to 39)
SSRIs	22675	**-0.30 (-0.42 to -0.18)**	29.69 (23 to 36)
Attention placebo	87	-0.22 (-0.98 to 0.61)	30.15 (14 to 42)
Exercise individual	298	-0.18 (-1.43 to 1.19)	30.54 (8 to 43)
Counselling individual + AD	57	0.23 (-4.02 to 4.48)	30.55 (1 to 43)
Trazodone	1072	-0.12 (-0.34 to 0.10)	33.97 (27 to 40)
Pill placebo	12684	Reference	36.48 (31 to 41)

Waitlist	626	0.45 (0.04 to 0.86)	40.36 (36 to 43)

Treatment classes ordered from best to worst, according to mean ranking. Negative effect values indicate a favourable outcome for treatment classes compared with pill placebo. Results where 95% CrI do not cross the no effect line are shown in bold.
AD: antidepressant; CBT: cognitive behavioural therapy; CrI: credible intervals; CT: cognitive therapy; IPT: interpersonal psychotherapy; PDPT: psychodynamic psychotherapy; SMD: standardised mean difference; SNRIs: serotonin and norepinephrine reuptake inhibitors; SSRIs: selective serotonin uptake inhibitors; TAU: treatment as usual; TCAs: tricyclic antidepressants

Intervention level

Posterior effects (mean SMD, 95%CrI) of all interventions versus pill placebo. Only interventions of interest belonging to classes with N ≥50 have been included in the table.

Class	N	SMD vs pill placebo (mean, 95% CrI)	Intervention	N	SMD vs pill placebo (mean, 95% CrI)
Self-help without/with minimal support	523	-0.79 (-1.84 to 0.16)	**Cognitive bibliotherapy**	**159**	**-0.93 (-1.54 to -0.38)**
			Computerised attentional bias modification	26	-0.64 (-1.58 to 0.50)
			Computerised cognitive bias modification	29	-0.49 (-1.33 to 0.63)
			**Computerised-CBT (CCBT)**	**270**	**-0.65 (-1.12 to -0.18)**
			**Mindfulness meditation CD**	**39**	**-1.26 (-3.17 to -0.19)**
Self-help with support	418	-0.62 (-1.56 to 0.40)	Cognitive bibliotherapy with support	66	-0.57 (-1.20 to 0.16)
			**Computerised-CBT (CCBT) with support**	**315**	**-0.69 (-1.12 to -0.26)**
			Mindfulness meditation CD with support	19	-0.53 (-1.76 to 1.02)
			Relaxation training CD with support	18	-0.72 (-2.16 to 0.6)
**Behavioural therapies individual**	**378**	**-0.96 (-2.02 to -0.01)**	**Behavioural activation (BA) individual**	**368**	**-0.85 (-1.38 to -0.32)**
			**Behavioural therapy (Lewinsohn 1976) individual**	**10**	**-1.08 (-1.99 to -0.32)**
**CT/CBT individual**	**1,044**	**-0.85 (-1.76 to -0.28)**	**CBT individual (15 sessions or over)**	**626**	**-0.63 (-0.92 to -0.32)**
			**CBT individual (under 15 sessions)**	**369**	**-0.71 (-1.07 to -0.37)**
			**Dialectical behavioural therapy (DBT) individual**	**10**	**-1.24 (-2.91 to -0.40)**
			**Third-wave cognitive therapy individual**	**39**	**-0.82 (-1.45 to -0.30)**
CT/CBT group	165	-0.31 (-1.65 to 0.97)	CBT group (under 15 sessions)	165	-0.31 (-0.77 to 0.15)
Problem solving individual	367	-0.90 (-2.24 to 0.38)	**Problem solving individual**	**367**	**-0.90 (-1.41 to -0.40)**
Counselling individual	404	-0.51 (-1.83 to 0.77)	**Non-directive/supportive/person-centred counselling**	**404**	**-0.51 (-0.94 to -0.08)**
IPT individual	146	-0.43 (-1.75 to 0.89)	IPT individual	146	-0.43 (-0.97 to 0.10)
Short-term PDPT individual	233	-0.70 (-1.77 to 0.24)	**Dynamic interpersonal therapy (DIT) individual**	**73**	**-0.88 (-1.82 to -0.14)**
			Short-term PDPT individual	160	-0.51 (-1.03 to 0.02)
**SSRIs**	**22,675**	**-0.30 (-0.42 to -0.18)**	**Citalopram**	**2195**	**-0.28 (-0.41 to -0.15)**
			**Escitalopram**	**5456**	**-0.41 (-0.53 to -0.28)**
			**Fluoxetine**	**6162**	**-0.25 (-0.35 to -0.15)**
			**Paroxetine**	**5861**	**-0.28 (-0.37 to -0.18)**
			**Sertraline**	**2794**	**-0.28 (-0.39 to -0.16)**

**TCAs**	**4,816**	**-0.33 (-0.51 to -0.14)**	**Amitriptyline**	**2504**	**-0.38 (-0.51 to -0.25)**
			**Clomipramine**	**345**	**-0.33 (-0.53 to -0.10)**
			**Imipramine**	**1306**	**-0.32 (-0.46 to -0.16)**
			**Lofepramine**	**145**	**-0.36 (-0.62 to -0.13)**
			**Nortriptyline**	**495**	**-0.28 (-0.47 to -0.02)**
**SNRIs**	**9,646**	**-0.37 (-0.55 to -0.19)**	**Duloxetine**	**5269**	**-0.37 (-0.49 to -0.26)**
			**Venlafaxine**	**4377**	**-0.37 (-0.48 to -0.26)**
**Mirtazapine**	**1,884**	**-0.35 (-0.52 to -0.19)**	**Mirtazapine**	**1,884**	**-0.35 (-0.52 to -0.19)**
Trazodone	1,072	-0.12 (-0.34 to 0.10)	Trazodone	1,072	-0.12 (-0.34 to 0.10)
Acupuncture	278	-0.44 (-2.01 to 0.84)	Electroacupuncture	124	-0.46 (-1.00 to 0.04)
			Laser acupuncture	39	-0.69 (-2.61 to 0.83)
			Traditional acupuncture	115	-0.17 (-0.64 to 0.34)
Exercise individual	298	-0.18 (-1.43 to 1.19)	Supervised high intensity exercise individual	128	-0.24 (-0.82 to 0.35)
			Supervised low intensity exercise individual	117	-0.05 (-0.75 to 0.77)
			Unsupervised high intensity exercise individual	53	-0.24 (-0.79 to 0.30)
Exercise group	106	-0.27 (-1.23 to 0.74)	Supervised high intensity exercise group	69	-0.34 (-0.85 to 0.18)
			Supervised low intensity exercise group	37	-0.21 (-0.84 to 0.49)
Yoga group	65	-0.89 (-2.38 to 0.54)	**Yoga group**	**65**	**-0.89 (-1.77 to -0.03)**
**CT/CBT individual + AD**	**192**	**-1.19 (-2.00 to -0.53)**	**CBT individual (15 sessions or over) + any AD**	**10**	**-1.36 (-2.51 to -0.47)**
			**CBT individual (15 sessions or over) + any SSRI**	**43**	**-0.84 (-1.45 to -0.18)**
			**CBT individual (15 sessions or over) + imipramine**	**25**	**-1.13 (-2.11 to -0.21)**
			**CBT individual (15 sessions or over) + nortriptyline**	**18**	**-0.98 (-1.90 to -0.02)**
			**CBT individual (under 15 sessions) + escitalopram**	**48**	**-0.79 (-1.40 to -0.11)**
			**CBT individual (under 15 sessions) + sertraline**	**38**	**-1.34 (-2.75 to -0.24)**
			**Third-wave cognitive therapy individual + any AD**	**10**	**-1.91 (-3.20 to -0.82)**
CT/CBT group + AD	63	-1.18 (-2.77 to 0.31)	**CBT group (under 15 sessions) + any AD**	**63**	**-1.18 (-1.89 to -0.47)**
IPT individual + AD	99	-0.71 (-1.92 to 0.43)	**IPT individual + any AD**	**87**	**-0.68 (-1.33 to -0.03)**
			Interpersonal counselling individual + venlafaxine	12	-0.74 (-1.78 to 0.24)
Counselling individual + AD	57	0.23 (-4.02 to 4.48)	Non-directive/supportive/person-centred counselling + any AD	15	0.56 (-2.16 to 3.46)
			Non-directive/supportive/person-centred counselling + any SSRI	17	-0.09 (-2.45 to 2.19)
			Non-directive/supportive/person-centred counselling + fluoxetine	25	0.23 (-6.72 to 6.98)

Short-term PDPT individual + AD	131	-0.41 (-2.23 to 1.34)	Short-term psychodynamic psychotherapy individual + any AD	113	-0.46 (-1.76 to 0.83)
			Short-term psychodynamic psychotherapy individual + any SSRI	18	-0.37 (-2.52 to 1.74)
**Exercise group + AD**	**79**	**-1.41 (-2.70 to -0.12)**	**Supervised high intensity exercise group + sertraline**	**42**	**-1.52 (-2.47 to -0.58)**
			**Supervised low intensity exercise group + sertraline**	**37**	**-1.30 (-2.28 to -0.32)**
**Acupuncture + AD**	**664**	**-0.69 (-1.05 to -0.32)**	**Electroacupuncture + any SSRI**	**160**	**-0.74 (-1.19 to -0.33)**
			**Electroacupuncture + fluoxetine**	**46**	**-0.67 (-1.14 to -0.16)**
			**Electroacupuncture + paroxetine**	**71**	**-0.79 (-1.25 to -0.41)**
			**Traditional acupuncture + any SSRI**	**206**	**-0.62 (-0.99 to -0.19)**
			**Traditional acupuncture + fluoxetine**	**80**	**-0.60 (-1.02 to -0.07)**
			**Traditional acupuncture + paroxetine**	**101**	**-0.71 (-1.11 to -0.34)**
Light therapy + AD	54	-0.91 (-2.54 to 0.72)	**Bright light therapy + fluoxetine**	**29**	**-1.00 (-1.67 to -0.36)**
			**Bright light therapy + venlafaxine**	**25**	**-0.81 (-1.52 to -0.04)**

Negative effect values indicate a favourable outcome for treatment classes and interventions compared with pill placebo. Results where 95% CrI do not cross the no effect line are shown in bold.
AD: antidepressant; CBT: cognitive behavioural therapy; CrI: credible intervals; CT: cognitive therapy; IPT: interpersonal psychotherapy; PDPT: psychodynamic psychotherapy; SMD: standardised mean difference; SNRIs: serotonin and norepinephrine reuptake inhibitors; SSRIs: selective serotonin uptake inhibitors; TAU: treatment as usual; TCAs: tricyclic antidepressants

Comparison between original and updated results (SMD versus reference)

The graphs below illustrate, for each level of depression symptom severity, the effects (mean SMD, 95%CrI) of each treatment class and intervention versus the relevant reference treatment (a) in the original bias-adjusted analysis that informed the NICE guideline (in red dotted lines) and (b) in the 2023 updated bias-adjusted analysis.

Less severe depression

Treatment class level

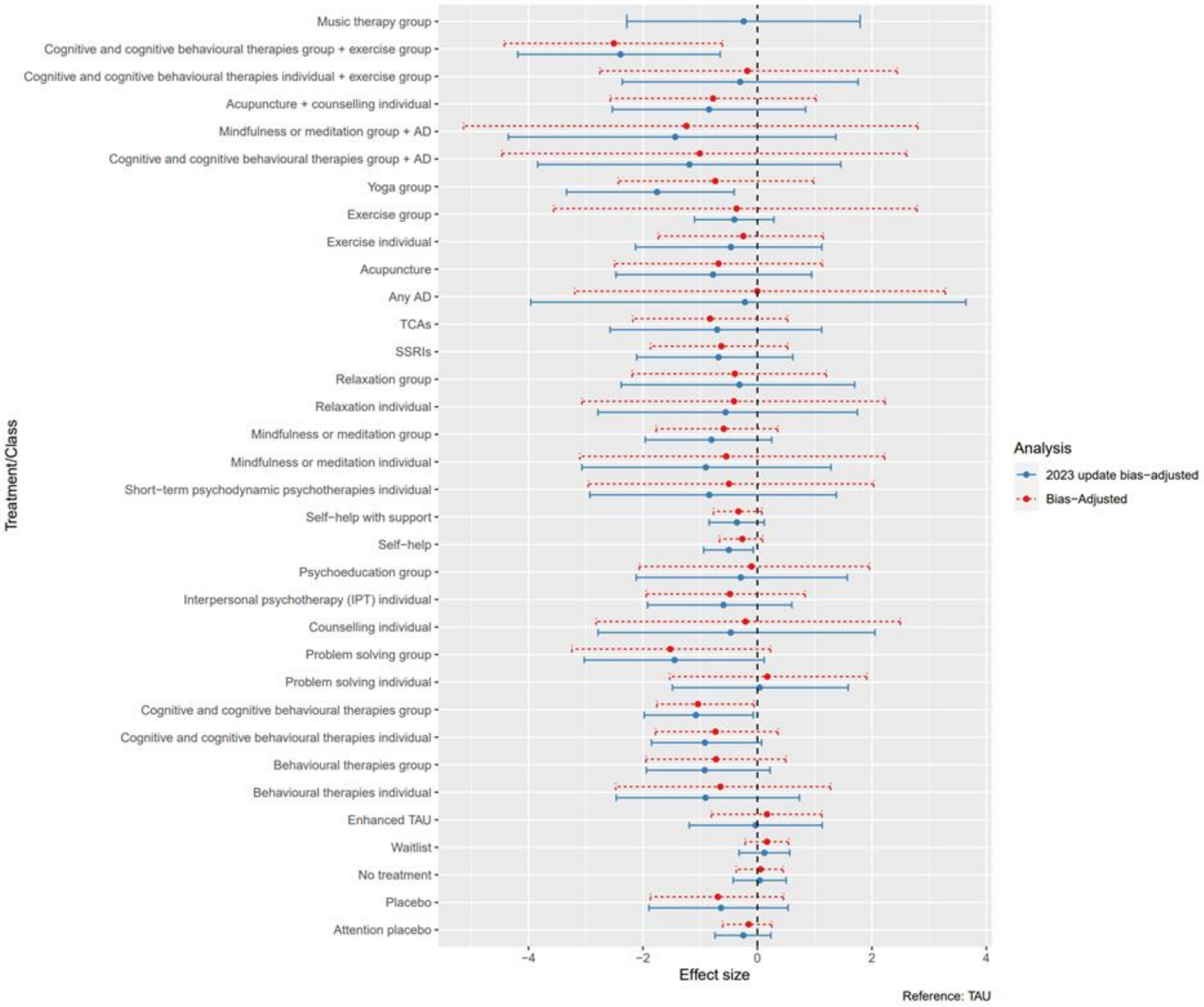

Intervention level

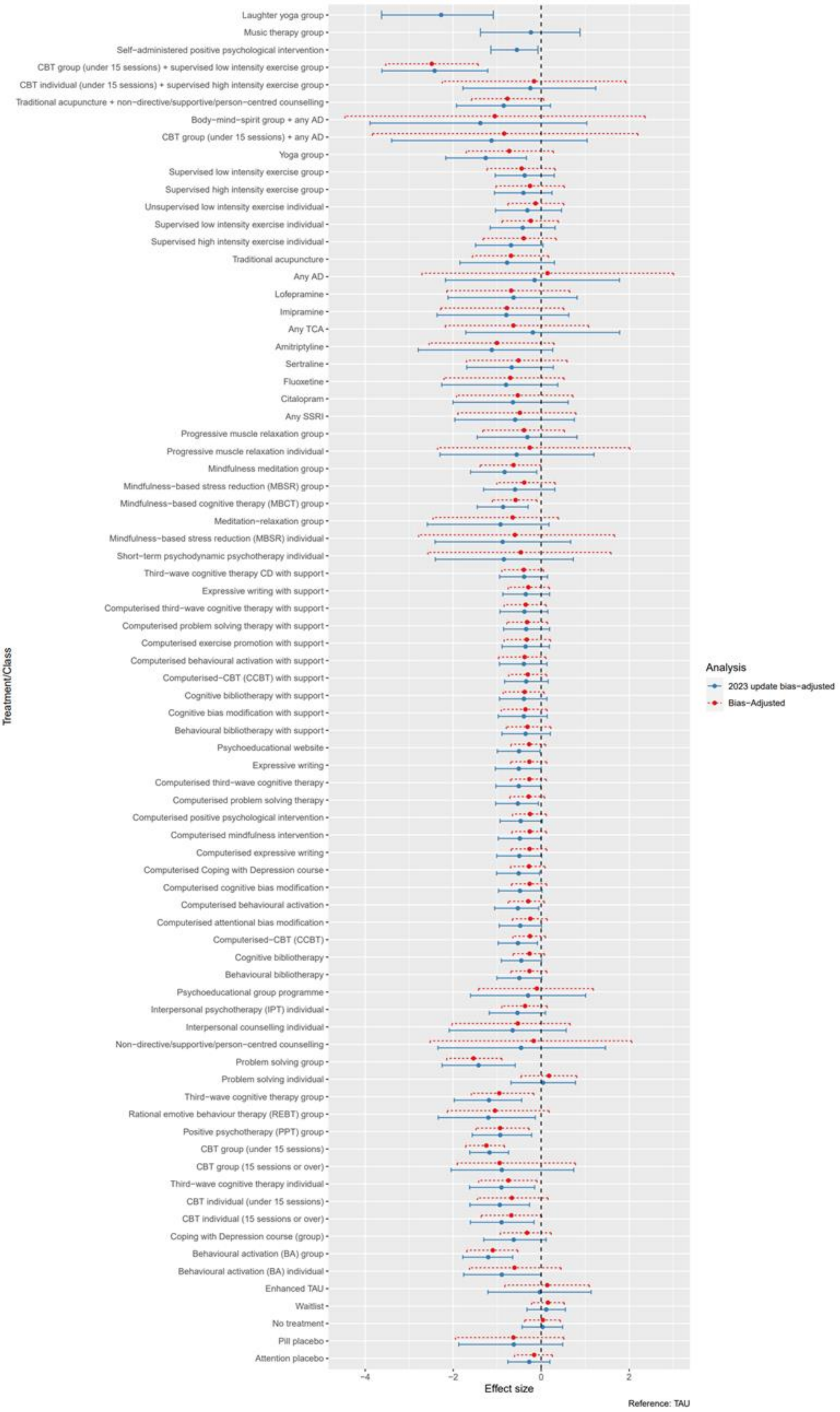

More severe depression

Treatment class level

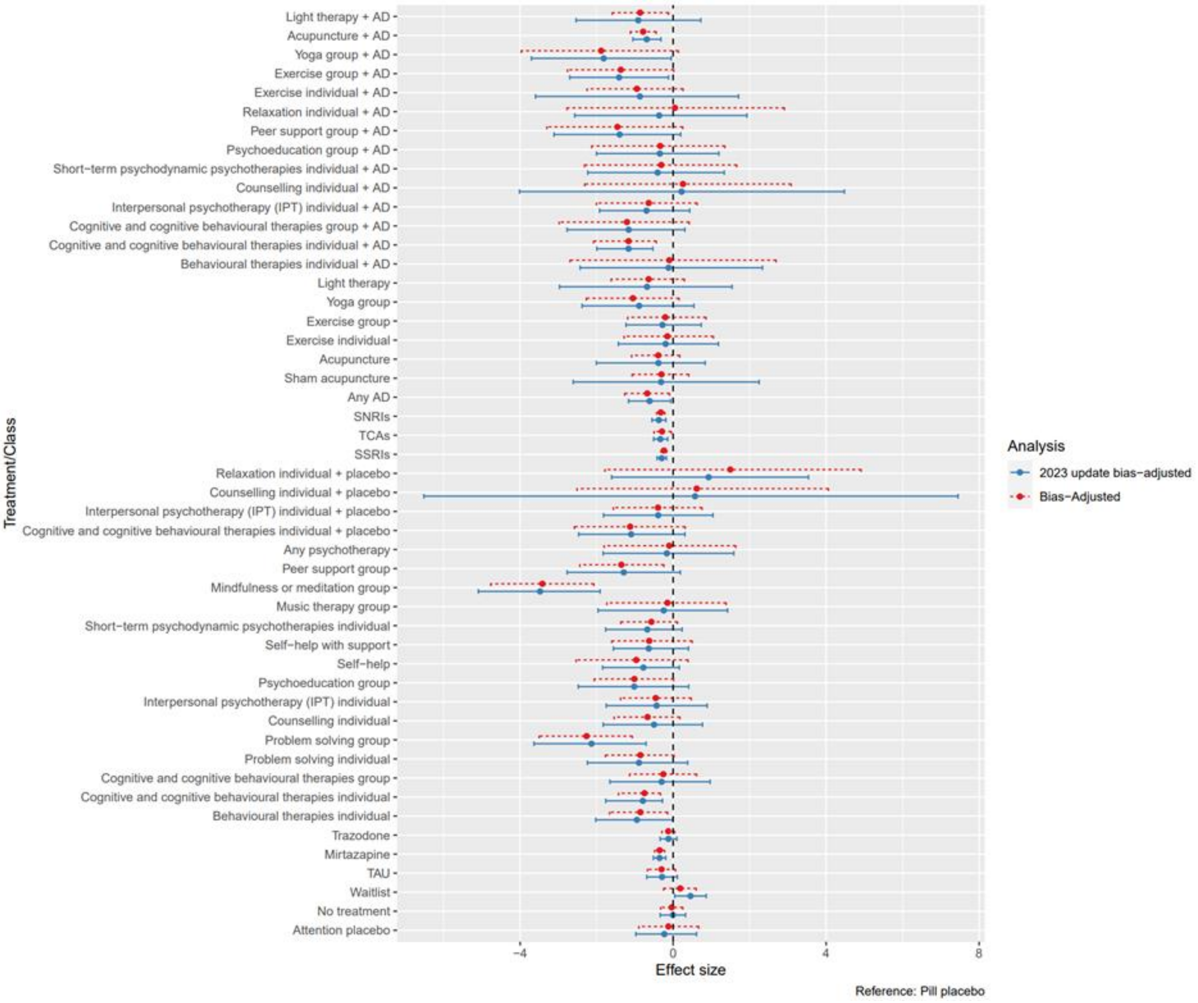

Intervention level

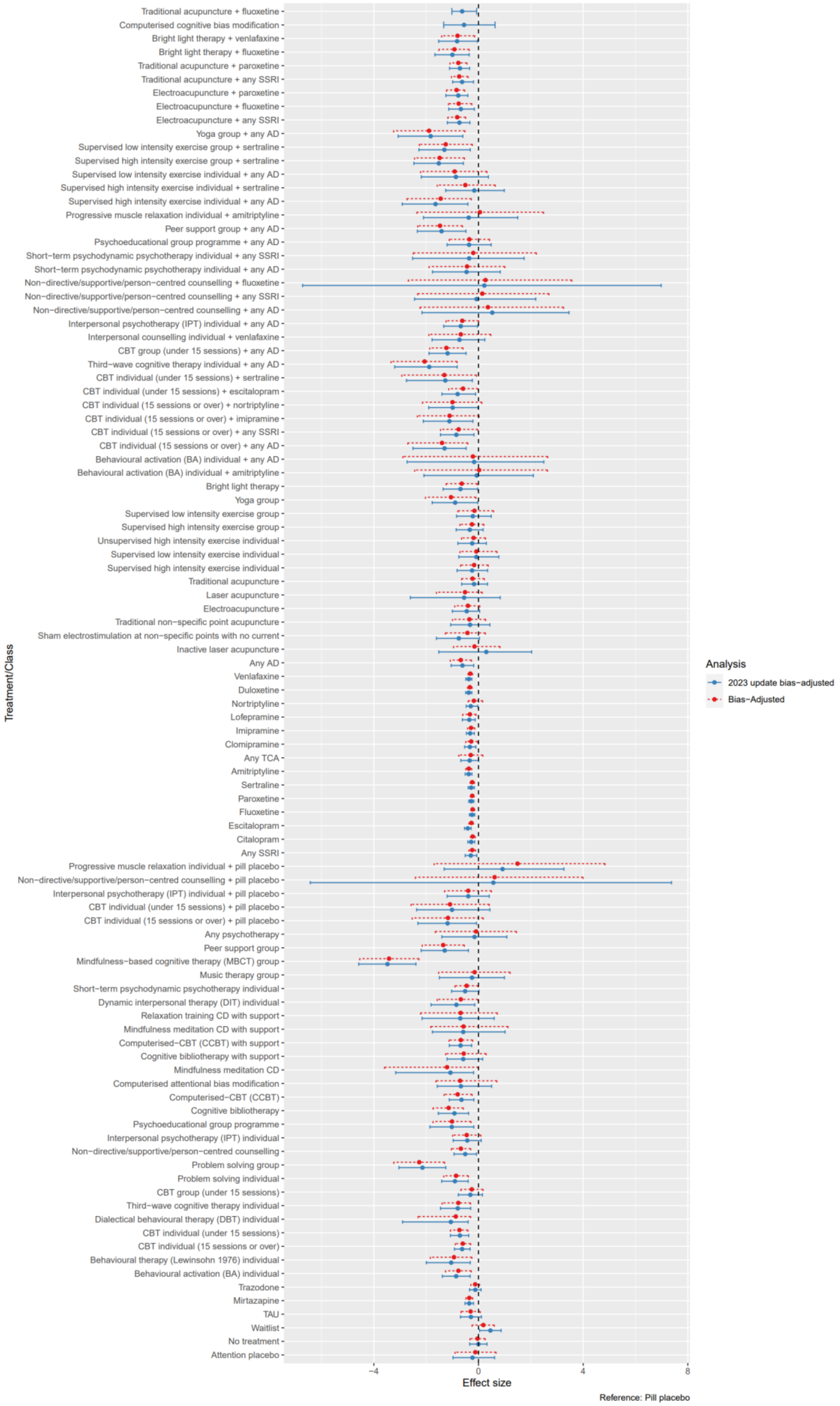

Supplement: Appendix 9 2023 Review update results [file mmc9.pdf]
