# Supplementary material for: A systematic review and network meta-analysis of psychological, psychosocial, pharmacological, physical and combined treatments for adults with a new episode of depression
Source: eClinicalMedicine. 2024 Aug 16;75:102780. doi: 10.1016/j.eclinm.2024.102780 (PMC11377144; doi:10.1016/j.eclinm.2024.102780)
Supplement: Appendix 11 2024 Search update results [file mmc11.pdf]

APPENDIX 11 – 2024 SEARCH UPDATE RESULTS

CONTENTS

Flow diagram of study selection from the 2024 search update..2

Eligible and non-eligible studies identified from the 2024 search update.......................3

Eligible studies: treatments assessed, overview of findings and full references ...3

List of non-eligible studies, with reasons for non-eligibility, and full references....6

Flow diagram of study selection from the 2024 search update

Figure 1: Flow diagram of study selection from the 2024 search update (only new records identified between November 2023 – July 2024 included)

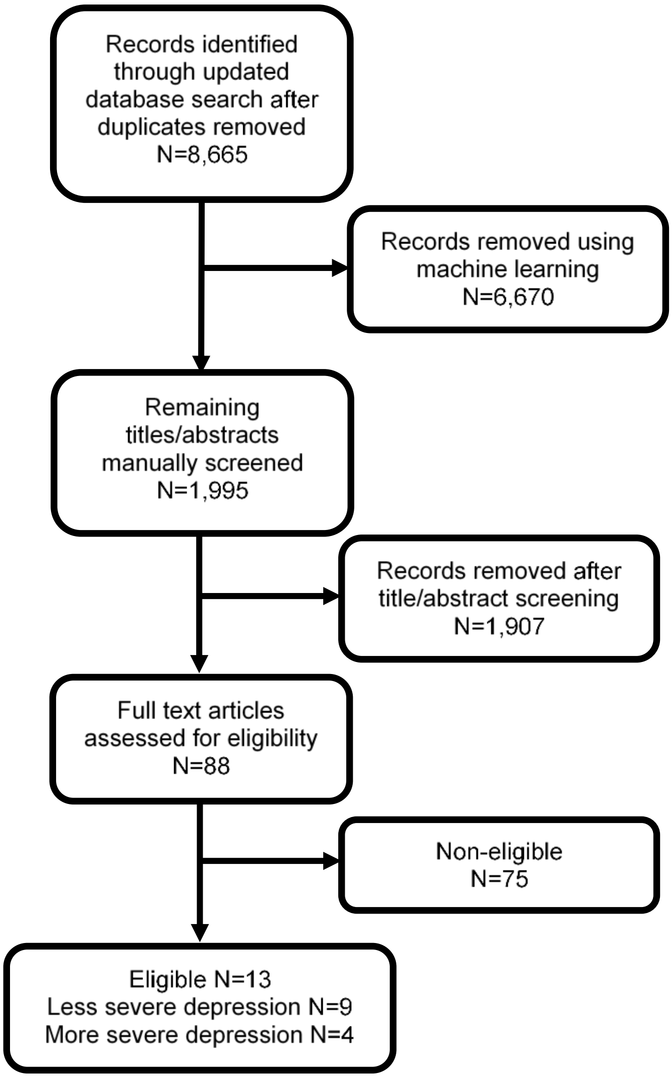

Eligible and non-eligible studies identified from the 2024 search update

Eligible studies: treatments assessed, overview of findings and full references

Less severe depression			
	**Full study reference**	**Treatments and comparators assessed**	**N randomized**<br>**Estimated SMD change score**<br>**Author conclusions**
1.	Alfurjani, A. M., Al-Hammouri, M. M., Rababah, J. A., Alhawatmeh, H. N., & Hall, L. A. (2024). The effect of a mindfulness-based intervention on stress overload, depression, and mindfulness among nurses: A randomized controlled trial. Worldviews on Evidence-Based Nursing, 21(1), 34-44.	Class: Mindfulness or meditation group<br>Specific intervention: Mindfulness-based stress reduction (MBSR) group [2 arms: conducted inside or outside]<br><br>Waitlist	N=195 (130 intervention; 65 control)<br><br>SMD change score: -2.10 [-2.46, -1.73]<br><br>MBSR significantly reduced depression symptoms (CES-D) relative to waitlist
2.	Liu, W., Yuan, J., Wu, Y., Xu, L., Wang, X., Meng, J., ... & Yang, J. Z. (2024). A randomized controlled trial of mindfulness-based cognitive therapy for major depressive disorder in undergraduate students: Dose-response effect, inflammatory markers and BDNF. Psychiatry Research, 331, 115671.	Class: Mindfulness or meditation group<br>Specific intervention: Mindfulness-based cognitive therapy (MBCT) group<br><br>Waitlist	N=56 (26 intervention; 30 control)<br><br>SMD change score: -4.09 [-5.03, -3.14]<br><br>MBCT significantly reduced depression symptoms (PHQ-9) relative to waitlist
3.	Chen, G., Chen, P., Yang, Z., Ma, W., Yan, H., Su, T., ... & Wang, Y. (2024). Increased functional connectivity between the midbrain and frontal cortex following bright light therapy in subthreshold depression: A randomized clinical trial. American Psychologist, 79(3), 437.	Class: Light therapy<br>Specific intervention: Bright light therapy<br><br>Attention placebo	N=74 (38 intervention; 36 control)<br><br>SMD change score: -0.85 [-1.33, -0.38]<br><br>Bright light therapy reduced depression symptoms (HAMD-24) relative to placebo
4.	James Vibin, A., Niharika, N., Valliappan, V., Lamo, P., Parajuli, N., Jat, M., ... & Sharma, G. (2024). Effect of Integrated Yoga as an add-on therapy in adults with clinical depression–A randomized controlled trial. International Journal of Social Psychiatry, 70(4), 709-719.	Class: Yoga group (+TAU)<br>Specific intervention: Yoga group (+ TAU)<br><br>Attention placebo (+TAU)	N=65 [completer analysis] (30 intervention; 35 control)<br><br>SMD change score: -0.81 [-1.32, -0.30]<br><br>Yoga group reduced depression symptoms (BDI-II) relative to an attention placebo condition
5.	Kiraz, S., & Yıldırım, S. (2023). The effect of regular exercise on depression, anxiety, treatment motivation and mindfulness in addiction: a randomized controlled trial. Journal of Substance Use, 28(4), 643-650.	Class: Exercise group (+TAU)<br>Specific intervention: Supervised high intensity exercise group (+ TAU)<br><br>No treatment (+ TAU)	N=60 [completer analysis] (30 intervention; 30 control)<br><br>SMD change score: -2.07 [-2.70, -1.43]<br><br>Exercise (+ TAU) reduced depression symptoms (PHQ-9) relative to no exercise (+ TAU) condition

Less severe depression			
6.	Kong, F., Yu, L., Hou, Y., Zhu, L., Zhou, J., Huang, L., ... & Ying, Y. (2024). Efficacy of Internet-Based Cognitive Behavioral Therapy for Subthreshold Depression Among Older Adults in Institutional Long-Term Care Settings: Pragmatic Randomized Controlled Trial. Journal of Medical Internet Research, 26, e40187.	Class: Self-help<br>Specific intervention: Computerised-CBT (CCBT)<br><br>Class: Cognitive and cognitive behavioural therapies group<br>Specific intervention: CBT group (under 15 sessions)<br><br>Waitlist	N=315 [‘completer’ analysis] (105 CCBT; 104 CBT group; 106 waitlist)<br><br>SMD change score:<br>CCBT vs waitlist: -0.36 [-0.64, -0.09]<br>CBT group vs waitlist: 0.14 [-0.13, 0.41]<br>CCBT vs CBT group: -0.50 [-0.78, -0.23]<br><br>CCBT had small but significant effect on depression symptoms (PHQ-9) relative to waitlist<br>CBT group did not significantly reduce depression symptoms (PHQ-9) relative to waitlist<br>CCBT reduced depression symptoms (PHQ-9) relative to group CBT
7.	Srivastava, K., Chatterjee, K., Prakash, J., Yadav, A., & Chaudhury, S. (2024). Comparative efficacy of cognitive behavior therapy and interpersonal therapy in the treatment of depression: A randomized controlled study. Industrial Psychiatry Journal, 33(1), 160-167.	Class: Cognitive and cognitive behavioural therapies individual (+TAU)<br>Specific intervention: CBT individual (under 15 sessions) (+ TAU)<br><br>Class: Interpersonal psychotherapy (IPT) individual (+ TAU)<br>Specific intervention: Interpersonal psychotherapy (IPT) individual (+TAU)	N=50 [completer analysis] (24 CBT; 26 IPT)<br><br>SMD change score: 3.71 [2.77, 4.65]<br><br>IPT individual reduced depression symptoms (MADRS) relative to CBT individual, but both interventions showed improvement from baseline
8.	Young, D. K. W., Carlbring, P., Cheng, D. Y. T., Ng, S. M., Ng, P. Y. N., & Chen, J. Q. R. (2024). Guided Online Mindfulness-Based Intervention for Community Residents with Emotional Distress during the COVID-19 Pandemic. Social Work, 69(2), 158-166.	Class: Self-help with support<br>Specific intervention: *Computerised MBCT with support*<br><br>Waitlist	N=64 (32 in each arm)<br><br>SMD change score: -0.85 [-1.36, -0.34]<br><br>Self-help with support reduced depression symptoms (BDI-II) relative to waitlist
9.	Zarski, A. C., Weisel, K. K., Berger, T., Krieger, T., Schaub, M. P., Berking, M., ... & Ebert, D. D. (2024). Efficacy of an Internet-and Mobile-Based Intervention for Subclinical Anxiety and Depression (ICare Prevent) with Two Guidance Formats: Results from a Three-Armed Randomized Controlled Trial. Psychotherapy and psychosomatics, 1-14.	Class: Self-help<br>Specific intervention: Computerised-CBT (CCBT)<br><br>Class: Self-help with support<br>Specific intervention: Computerised-CBT (CCBT) with support<br><br>Waitlist	N=577 (186 self-help with support; 189 self-help; 191 waitlist)<br><br>SMD change score:<br>Self-help vs to waitlist: -0.51 [-0.71, -0.30]<br>Self-help with support vs waitlist: -0.69 [-0.89, -0.48]<br>Self-help with support vs self-help: -0.17 [-0.37, 0.03]<br><br>Self-help and self-help with support reduced depression symptoms (QIDS) relative to waitlist<br>No significant difference between self-help with support and self-help

More severe depression			
	**Full study reference**	**Treatments and comparators assessed**	**N randomized**<br>**Estimated SMD change score**<br>**Author conclusions**
1.	El-Haj-Mohamad, R., Böttche, M., Vöhringer, M., Specht, F., Stammel, N., Nesterko, Y., ... & Wagner, B. (2024). An internet-based cognitive behavioural intervention for adults with depression in Arabic-speaking countries: A randomized controlled trial. Stress and Health, e3432.	Class: Self-help with support<br>Specific intervention: Computerised-CBT (CCBT) with support<br><br>Waitlist	N=259 (128 intervention; 131 control)<br><br>SMD change score: -0.70 [-0.95, -0.45]<br><br>CCBT reduced depression symptoms (BDI-II) relative to waitlist
2.	Harald, C. M. W. J. B., & Krämera, B. L. V. (2023). Effects of a Web-Based Behavioral Activation Intervention on Depressive Symptoms, Activation, Motivation, and Volition: Results of a Randomized Controlled Trial. Psychother Psychosom, 92, 367-378.	Class: Self-help with support (+ TAU)<br>Specific intervention: Computerised behavioural activation with support (+ TAU)<br><br>Waitlist (+ TAU)	N=128 (64 intervention; 64 control)<br><br>SMD change score: -0.99 [-1.36, -0.63]<br><br>Computerised BA reduced depression symptoms (QIDS) relative to waitlist
3.	Mo, Y., Lei, Z., Chen, M., Deng, H., Liang, R., Yu, M., & Huang, H. (2023). Effects of self-help mindfulness-based cognitive therapy on mindfulness, symptom change, and suicidal ideation in patients with depression: a randomized controlled study. Frontiers in psychology, 14, 1287891.	Class: Self-help (+ TAU)<br>Specific intervention: *Self-administered MBCT (+ TAU)*<br><br>Waitlist (+ TAU)	N=97 (48 intervention; 49 control)<br><br>SMD change score: -1.25 [-1.68, -0.81]<br><br>Self-administered MBCT reduced depression symptoms (HAMD) relative to waitlist
4.	Shamabadi, A., Karimi, H., Fallahzadeh, M. A., Vaseghi, S., Bahri, R. A., Fallahpour, B., ... & Akhondzadeh, S. (2024). Sex-controlled differences in sertraline and citalopram efficacies in major depressive disorder: a randomized, double-blind trial. International Clinical Psychopharmacology, 10-1097.	Class: SSRIs<br>Specific intervention: Sertraline<br><br>Class: SSRIs<br>Specific intervention: Citalopram	N=80 (40 in each arm)<br><br>SMD change score: -0.14 [-0.58, 0.30]<br><br>No significant difference between sertraline and citalopram (within-class comparison) in depression symptom change scores (HAMD), both showed improved depression symptoms

List of non-eligible studies, with reasons for non-eligibility, and full references

	Full study reference	Reason for non-eligibility
1.	Alvarez, C., Aryal, S., Vrany, E., Quiles, R., Escobar-Acosta, L., & Hill-Briggs, F. (2024). Remote Delivery of the Cuidándome Telehealth Intervention for Self-Management of Depression and Anxiety Among Latina Immigrant Women: Randomized Controlled Trial. JMIR Formative Research, 8(1), e52969.	Outcome measure(s) outside protocol
2.	Aminoff, V., Bobeck, J., Hjort, S., Sörliden, E., Ludvigsson, M., Berg, M., & Andersson, G. (2023). Tailored internet-based psychological treatment for psychological problems during the COVID-19 pandemic: A randomized controlled trial. Internet interventions, 34, 100662.	Data cannot be extracted (ITT at baseline and completer at endpoint)
3.	Bademli, K., Lök, N., & Lök, S. (2023). The Effect of a Physical Activity Intervention on Burden and Depressive Symptoms in Depressed Family Caregivers of Patients With Schizophrenia: A Randomized Controlled Trial. Journal of Physical Activity and Health, 20(12), 1109-1115.	Paper unavailable
4.	Balci, S., Küchler, A. M., Ebert, D. D., & Baumeister, H. (2023). An online mindfulness intervention for international students: a randomized controlled feasibility trial. Clinical Psychology in Europe, 5(2).	Outcome measure(s) outside protocol
5.	Bantjes, J., Hunt, X., Cuijpers, P., Kazdin, A. E., Kennedy, C. J., Luedtke, A., ... & Kessler, R. C. (2024). Comparative effectiveness of remote digital gamified and group CBT skills training interventions for anxiety and depression among college students: Results of a three-arm randomised controlled trial. Behaviour Research and Therapy, 178, 104554.	Outcome measure(s) outside protocol
6.	Bartels, G. C., Cheavens, J. S., & Strunk, D. R. (2024). Entrée or sampler? A randomized controlled trial of two approaches to single session internet-based interventions. Cognitive Therapy and Research, 48(2), 350-360.	Data cannot be extracted (baseline/change scores not reported for depression)
7.	Bell, I., Arnold, C., Gilbertson, T., D'Alfonso, S., Castagnini, E., Chen, N., ... & Alvarez-Jimenez, M. (2023). A Personalized, Transdiagnostic Smartphone Intervention (Mello) Targeting Repetitive Negative Thinking in Young People With Depression and Anxiety: Pilot Randomized Controlled Trial. Journal of Medical Internet Research, 25, e47860.	Outcome measure(s) outside protocol
8.	Bisby, M. A., Barrett, V., Staples, L. G., Nielssen, O., Dear, B. F., & Titov, N. (2024). Things You Do: a randomized controlled trial of an unguided ultra-brief intervention to reduce symptoms of depression and anxiety. Journal of Anxiety Disorders, 102882.	<80% first-line treatment
9.	Brasser, M., Frühholz, S., Schneeberger, A. R., Trevor, C., Ruschetti, G. G., Held, F. E., ... & Studer-Luethi, B. (2024). The effectiveness of cognitive training in people in psychiatry with depressive symptoms–A randomized controlled study. Journal of Affective Disorders Reports, 17, 100805.	Further-line treatment
10.	Chen, M. L., Wu, Y. J., Lee, M. J., Hsieh, S. L., Tseng, I. J., Chen, L. S., & Gardenhire, D. S. (2023). Effects of Resistance Exercise on Cognitive Performance and Depressive Symptoms in Community-Dwelling Older Chinese Americans: A Pilot Randomized Controlled Trial. Behavioral Sciences, 13(3), 241.	Outcome measure(s) outside protocol
11.	Vezmar, M., Ćirović, N., Sudar, B., Buzejić, J., Jovanović, T., Radanović, A., & Vezmar, M. (2024). The effectiveness of group art therapy in a clinically heterogenous sample: Randomized controlled trial. The Arts in Psychotherapy, 89, 102150.	Outcome measure(s) outside protocol
12.	de Oliveira Rodrigues, D. M., Menezes, P. R., Silotto, A. E. M. R., Heps, A., Sanches, N. M. P., Schveitzer, M. C., & Faisal-Cury, A. (2023). Efficacy and Safety of Auricular Acupuncture for Depression: A Randomized Clinical Trial. JAMA Network Open, 6(11), e2345138-e2345138.	Data cannot be extracted (depression symptom scores reported as medians)
13.	Deady, M., Collins, D. A., Lavender, I., Mackinnon, A., Glozier, N., Bryant, R., ... & Harvey, S. B. (2023). Selective Prevention of Depression in Workers Using a Smartphone App: Randomized Controlled Trial. Journal of Medical Internet Research, 25, e45963.	Not depression (below sub-threshold level)
14.	Dong, M., Li, Y., & Zhang, Y. (2023). The effect of mindfulness training on executive function in youth with depression. Acta Psychologica, 235, 103888.	Outcome(s) not of interest

15.	Ekeblad, A., Holmqvist, R., Andersson, G., & Falkenström, F. (2023). Change in reflective functioning in interpersonal psychotherapy and cognitive behavioral therapy for major depressive disorder. Psychotherapy Research, 33(3), 342-349.	Outcome(s) not of interest
16.	Elgendy, H., Shalaby, R., Agyapong, B., Lesage, D., Paulsen, L., Delday, A., ... & Agyapong, V. I. O. (2024). Effectiveness of Group Physical Exercise in Treating Major Depressive Disorder: An Analysis of Secondary Data from an Aborted Randomized Trial. Behavioral Sciences, 14(3), 219.	Non-RCT
17.	Farhadi, M., Rahimi, H., Paydar, M. R. Z., & Vasel, M. Y. (2023). The Effectiveness of Self-Compassion-Focused Therapy on Cognitive Vulnerability to Depression. Iranian Journal of Psychiatry, 18(2), 134.	Data cannot be extracted (depression symptom scores not reported)
18.	Forman-Hoffman, V. L., Sihvonen, S., Wielgosz, J., Kuhn, E., Nelson, B. W., Peiper, N. C., & Gould, C. E. (2024). Therapist-supported digital mental health intervention for depressive symptoms: A randomized clinical trial. Journal of Affective Disorders, 349, 494-501.	<80% first-line treatment
19.	Gaines, A. N., Constantino, M. J., Coyne, A. E., Atkinson, L. R., Bagby, R. M., Ravitz, P., & McBride, C. (2023). Change in satisfaction with social support as a common outcome in interpersonal psychotherapy and cognitive behavioral therapy for depression. Journal of Psychotherapy Integration, 33(4), 457.	Data cannot be extracted (depression symptom scores not reported)
20.	Gallo, G. G., Curado, D. F., Machado, M. P. A., Espíndola, M. I., Scattone, V. V., & Noto, A. R. (2023). A randomized controlled trial of mindfulness: effects on university students' mental health. International Journal of Mental Health Systems, 17(1), 32.	Data cannot be extracted (depression symptom scores not reported)
21.	Gellert, P., Lech, S., Hoppmann, F., O'Sullivan, J. L., & Kessler, E. M. (2024). Effectiveness of Psychotherapy for Community-Dwelling Vulnerable Older Adults with Depression and Care Needs: Findings from the PSY-CARE Trial. Clinical Gerontologist, 1-15.	Outcome measure(s) outside protocol
22.	Ghosh, A., Cherian, R. J., Wagle, S., Sharma, P., Kannan, K. R., Bajpai, A., & Gupta, N. (2023). An unguided, computerized cognitive behavioral therapy intervention (TreadWill) in a lower middle-income country: Pragmatic randomized controlled trial. Journal of Medical Internet Research, 25, e41005.	Completion data <50%/>50% left treatment early
23.	Große, J., Huppertz, C., Röh, A., Oertel, V., Andresen, S., Schade, N., ... & Ströhle, A. (2024). Step away from depression—results from a multicenter randomized clinical trial with a pedometer intervention during and after inpatient treatment of depression. European Archives of Psychiatry and Clinical Neuroscience, 274(3), 709-721.	Data cannot be extracted (ITT at baseline and completer at endpoint)
24.	Guertler, D., Krause, K., Moehring, A., Bischof, G., Batra, A., Freyer-Adam, J., ... & Meyer, C. (2023). E-Health intervention for subthreshold depression: Reach and two-year effects of a randomized controlled trial. Journal of Affective Disorders, 339, 33-42.	Outcome measure(s) outside protocol
25.	Haakana, R., Rosenström, T., Parkkinen, L., Tuomisto, M. T., & Isometsä, E. (2024). Effectiveness of an add-on brief group behavioral activation treatment for depression in psychiatric care: a randomized clinical trial. Frontiers in psychiatry, 15, 1284363.	Data cannot be extracted (ITT at baseline and completer at endpoint)
26.	Hallford, D. J., Rusanov, D., Yeow, J. J. E., Austin, D. W., D'Argembeau, A., Fuller-Tyszkiewicz, M., & Raes, F. (2023). Reducing anhedonia in major depressive disorder with future event specificity training (FEST): a randomized controlled trial. Cognitive Therapy and Research, 47(1), 20-37.	Data cannot be extracted (ITT at baseline and completer at endpoint)
27.	Harra, R. C., & Vargas, I. (2023). A peer-based mentoring program for reducing anxiety and depression symptoms among college students: A preliminary study. Journal of American college health, 1-8.	Outcome measure(s) outside protocol
28.	Hirokawa-Ueda, H., Sawamura, Y., Kawakami, T., Sakane, H., Teramoto, K., Yamamoto, A., ... & Ono, H. (2023). Interpersonal counseling versus active listening in the treatment of mild depression: a randomized controlled trial. Journal of Physical Therapy Science, 35(7), 533-537.	Outcome measure(s) outside protocol
29.	Jallalian, M., Majd, A., & Ghalehnoei, H. (2023). Comparison of Serum BDNF changes in Women with Major Depressive Disorder Treated with Fluoxetine and Cognitive Behavioral Therapy: A Randomized Clinical Trial. Journal of Mazandaran University of Medical Sciences, 33(1), 97-107.	Non-English language paper
30.	Jeong, S., Ca, C., Nam, S., & Song, J. (2024). The effects of mobile technology-based support on young women with depressive symptoms: A block randomized controlled trial. Medicine, 103(1), e36748.	Dismantling study

31.	Joubert, A. E., Grierson, A. B., Li, I., Sharrock, M. J., Moulds, M. L., Werner-Seidler, A., ... & Newby, J. M. (2023). Managing Rumination and worry: A randomised controlled trial of an internet intervention targeting repetitive negative thinking delivered with and without clinician guidance. Behaviour Research and Therapy, 168, 104378.	<80% first-line treatment
32.	Kalde, J., Atik, E., Stricker, J., Schückes, M., Neudeck, P., Pittig, A., & Pietrowsky, R. (2023). Enhancing the effectiveness of CBT for patients with unipolar depression by integrating digital interventions into treatment: A pilot randomized controlled trial. Psychotherapy Research, 1-16.	Data cannot be extracted (ITT at baseline and completer at endpoint)
33.	Karkosz, S., Szymański, R., Sanna, K., & Michałowski, J. (2024). Effectiveness of a Web-based and Mobile Therapy Chatbot on Anxiety and Depressive Symptoms in Subclinical Young Adults: Randomized Controlled Trial. JMIR formative research, 8(1), e47960.	Data cannot be extracted (ITT at baseline and completer at endpoint)
34.	Katayama, N., Nakagawa, A., Umeda, S., Terasawa, Y., Shinagawa, K., Kikuchi, T., ... & Mimura, M. (2023). Functional connectivity changes between frontopolar cortex and nucleus accumbens following cognitive behavioral therapy in major depression: A randomized clinical trial. Psychiatry Research: Neuroimaging, 332, 111643.	<80% first-line treatment
35.	Kemmeren, L. L., van Schaik, A., Draisma, S., Kleiboer, A., Riper, H., & Smit, J. H. (2023). Effectiveness of Blended Cognitive Behavioral Therapy Versus Treatment as Usual for Depression in Routine Specialized Mental Healthcare: E-COMPARED Trial in the Netherlands. Cognitive Therapy and Research, 47(3), 386-398.	<80% first-line treatment
36.	Kim, H., Lee, K., Lee, Y. H., Park, Y., Park, Y., Yu, Y., ... & Noh, S. (2023). The effectiveness of a mobile phone–based physical activity program for treating depression, stress, psychological well-being, and quality of life among adults: Quantitative study. JMIR mHealth and uHealth, 11, e46286.	Not depression (below sub-threshold level)
37.	Kleinau, E., Lamba, T., Jaskiewicz, W., Gorentz, K., Hungerbuehler, I., Rahimi, D., ... & Kapps, M. (2024). Effectiveness of a chatbot in improving the mental wellbeing of health workers in Malawi during the COVID-19 pandemic: A randomized, controlled trial. Plos one, 19(5), e0303370.	Not depression (below sub-threshold level)
38.	Koelen, J., Klein, A., Wolters, N., Bol, E., De Koning, L., Roetink, S., ... & Wiers, R. (2024). Web-Based, Human-Guided, or Computer-Guided Transdiagnostic Cognitive Behavioral Therapy in University Students With Anxiety and Depression: Randomized Controlled Trial. JMIR Mental Health, 11, e50503.	Completion data <50%/>50% left treatment early
39.	Kopf-Beck, J., Müller, C. L., Tamm, J., Fietz, J., Rek, N., Just, L., ... & Egli, S. (2024). Effectiveness of schema therapy versus cognitive behavioral therapy versus supportive therapy for depression in inpatient and day clinic settings: A randomized clinical trial. Psychotherapy and Psychosomatics, 93(1), 24-35.	<80% first-line treatment
40.	Krause-Sorio, B., Siddarth, P., Milillo, M. M., Kilpatrick, L., Ercoli, L., Narr, K. L., & Lavretsky, H. (2023). Grey matter volume predicts improvement in geriatric depression in response to Tai Chi compared to Health Education. International Psychogeriatrics, 1-9.	Secondary analysis/study
41.	Kung, P. H., Davey, C. G., Harrison, B. J., Jamieson, A. J., Felmingham, K. L., & Steward, T. (2023). Frontoamygdalar effective connectivity in youth depression and treatment response. Biological Psychiatry, 94(12), 959-968.	Secondary analysis/study
42.	Langenecker, S. A., Schreiner, M. W., Bessette, K. L., Roberts, H., Thomas, L., Dillahunt, A., ... & Watkins, E. R. (2024). Rumination-Focused Cognitive Behavioral Therapy Reduces Rumination and Targeted Cross-network Connectivity in Youth With a History of Depression: Replication in a Preregistered Randomized Clinical Trial. Biological Psychiatry Global Open Science, 4(1), 1-10.	Mean age <18 years
43.	Lee, Y. H., Kim, H., Hwang, J., & Noh, S. (2024). Effectiveness of Mobile-Based Progressive and Fixed Physical Activity on Depression, Stress, Anxiety, and Quality of Life Outcomes Among Adults in South Korea: Randomized Controlled Trial. JMIR mHealth and uHealth, 12(1), e55578.	Not depression (below sub-threshold level)
44.	Li, G., Sit, H. F., Chen, W., Wu, K., Sou, E. K. L., Wong, M., ... & Hall, B. J. (2024). A WHO digital intervention to address depression among young Chinese adults: a type 1 effectiveness-implementation randomized controlled trial. Translational psychiatry, 14(1), 102.	Data cannot be extracted

45.	Liu, J., Cheung, E. S. L., Lou, Y., & Wu, B. (2024). A peer mentoring program for Chinese American dementia caregivers: a pilot randomized controlled trial. Aging & Mental Health, 1-9.	Outcome measure(s) outside protocol
46.	Lorenzo-Luaces, L., & Howard, J. (2023). Efficacy of an unguided, digital single-session intervention for internalizing symptoms in web-based workers: Randomized controlled trial. Journal of Medical Internet Research, 25, e45411.	Outcome measure(s) outside protocol
47.	Lu, Y. E., Li, Y., Huang, Y., Zhang, X., Wang, J., Wu, L., & Cao, F. (2023). Effects and mechanisms of a web-and mobile-based acceptance and commitment therapy intervention for anxiety and depression symptoms in nurses: Fully decentralized randomized controlled trial. Journal of Medical Internet Research, 25, e51549.	Data cannot be extracted
48.	Mak, W. W., Tong, A. C., Fu, A. C., Leung, I. W., Jung, O. H., Watkins, E. R., & Lui, W. W. (2024). Efficacy of Internet-based rumination-focused cognitive behavioral therapy and mindfulness-based intervention with guided support in reducing risks of depression and anxiety: A randomized controlled trial. Applied Psychology: Health and Well-Being, 16(2), 696-722.	Data cannot be extracted (ITT at baseline and completer at endpoint)
49.	Medina, J. C., Paz, C., Salla, M., Aguilera, M., Montesano, A., Compan, V., & Feixas, G. (2023). The effect of two cognitive therapies on subjective wellbeing of individuals with depression: results from a randomised controlled trial. Journal of Mental Health, 32(3), 655-661.	<80% first-line treatment
50.	Moghimi, E., Stephenson, C., Agarwal, A., Nikjoo, N., Malakouti, N., Layzell, G., ... & Alavi, N. (2023). Efficacy of an Electronic Cognitive Behavioral Therapy Program Delivered via the Online Psychotherapy Tool for Depression and Anxiety Related to the COVID-19 Pandemic: Pre-Post Pilot Study. JMIR Mental Health, 10(1), e51102.	Non-RCT
51.	Nyer, M. B., Hopkins, L. B., Nagaswami, M., Norton, R., Streeter, C. C., Hoeppner, B. B., ... & Mischoulon, D. (2023). A Randomized Controlled Trial of Community-Delivered Heated Hatha Yoga for Moderate-to-Severe Depression. The Journal of clinical psychiatry, 84(6), 49607.	Outcome measure(s) outside protocol
52.	O'Sullivan, D., Gordon, B. R., Lyons, M., Meyer, J. D., & Herring, M. P. (2023). Effects of resistance exercise training on depressive symptoms among young adults: A randomized controlled trial. Psychiatry Research, 326, 115322.	Secondary analysis/study
53.	Potsch, L., & Rief, W. (2024). Effectiveness of behavioral activation and mindfulness in increasing reward sensitivity and reducing depressive symptoms-A randomized controlled trial. Behaviour Research and Therapy, 173, 104455.	Data cannot be extracted
54.	Powers, A., Lathan, E. C., McAfee, E., Mekawi, Y., Dixon, H. D., Lopez, E., ... & Kaslow, N. J. (2024). Feasibility and acceptability of a virtual mindfulness intervention for Black adults with PTSD and depression: Randomized controlled trial. Journal of Mood & Anxiety Disorders, 5, 100048.	Data cannot be extracted
55.	Purdie, D. R., Federman, M., Chin, A., Winston, D., Bursch, B., Olmstead, R., ... & Irwin, M. R. (2023). Hybrid delivery of mindfulness meditation and perceived stress in pediatric resident physicians: a randomized clinical trial of in-person and digital mindfulness meditation. Journal of clinical psychology in medical settings, 30(2), 425-434.	Not depression (below sub-threshold level)
56.	Rahgozar, S., & Giménez-Llort, L. (2024). Design and effectiveness of an online group logotherapy intervention on the mental health of Iranian international students in European countries during the COVID-19 pandemic. Frontiers in psychiatry, 15, 1323774.	Data cannot be extracted
57.	Rastogi, S., Kumar, R., Hussain, M. S., & Rastogi, V. (2023). Effect of Short Term Raj Yoga Meditation on Psychological Well Being in Young Adults. International Journal of Pharmaceutical and Clinical Research, 15(10), 1-5	<80% first-line treatment
58.	Rezapour-Mirsaleh, Y., Amini, R., Rezai, Z., & Javadian, F. S. (2023). Effectiveness of Existential Therapy Based on Ontological Core Schemas on Rumination and Mindfulness of Depressed Women: A Randomized Clinical Trial Design. Journal of Contemporary Psychotherapy, 53(2), 181-190.	Outcome(s) not of interest
59.	Rubin, M., Fischer, C. M., & Telch, M. J. (2024). Efficacy of a single session mindfulness based intervention: A randomized clinical trial. Plos one, 19(3), e0299300.	Outcome measure(s) outside protocol
60.	Ruzickova, T., Carson, J., Argabright, S., Gillespie, A., Guinea, C., Pearse, A., ... & Harmer, C. J. (2023). Online behavioural activation during the COVID-19 pandemic decreases depression and negative affective bias. Psychological medicine, 53(3), 795-804.	Data cannot be extracted

61.	Scazufca, M., Nakamura, C. A., Seward, N., Didone, T. V. N., Moretti, F. A., Oliveira da Costa, M., ... & Araya, R. (2024). Self-help mobile messaging intervention for depression among older adults in resource-limited settings: a randomized controlled trial. Nature Medicine, 30(4), 1127-1133.	Data cannot be extracted (ITT at baseline and completer at endpoint)
62.	Shapiro, M. O., Allan, N. P., Raines, A. M., & Schmidt, N. B. (2023). A randomized control trial examining the initial efficacy of an intolerance of uncertainty focused psychoeducation intervention. Journal of Psychopathology and Behavioral Assessment, 45(2), 379-390.	Data cannot be extracted (ITT at baseline and completer at endpoint)
63.	Shareh, H., & Yazdanian, M. (2023). The effectiveness of dialectical behavior group therapy on stress, depression, and cognitive emotion regulation in mothers of intellectually disabled students: A randomized clinical trial. Clinical Child Psychology and Psychiatry, 28(3), 1092-1108.	Data cannot be extracted (ITT at baseline and completer at endpoint)
64.	Stecher, C., Pagni, B. A., Cloonan, S., Vink, S., Hill, E., Ogbeama, D., ... & Braden, B. B. (2024). App-based meditation habits maintain reductions in depression symptoms among autistic adults. Autism, 28(6), 1487-1502.	Dismantling study
65.	Thomas, E. B. K., Sagorac Gruichich, T., Maronge, J. M., Hoel, S., Victory, A., Stowe, Z. N., & Cochran, A. (2023). Mobile acceptance and commitment therapy with distressed first-generation college students: microrandomized trial. JMIR Mental Health, 10, e43065.	Data cannot be extracted
66.	Wang, C., Si, H., Bian, Y., Qiao, X., Ji, L., Liu, Q., ... & Jin, Y. (2024). Effectiveness of subjective support-focused cognitive behavioral therapy on depressive symptoms among (pre) frail community-dwelling older adults: A randomized controlled trial. Journal of Affective Disorders, 347, 92-100.	Outcome measure(s) outside protocol
67.	Wang, Y., Yao, J., Koszycki, D., Jiang, W., Fang, F., Wang, M., ... & Qiu, J. (2023). Efficacy of dynamic interpersonal therapy for major depressive disorder in China: results of a multicentered, three-arm, randomized, controlled trial. Psychological Medicine, 53(15), 7242-7254.	Data cannot be extracted (ITT at baseline and completer at endpoint)
68.	Xiang, X., Kayser, J., Turner, S., Ash, S., & Himle, J. A. (2024). Layperson-Supported, Web-Delivered Cognitive Behavioral Therapy for Depression in Older Adults: Randomized Controlled Trial. Journal of Medical Internet Research, 26, e53001.	<80% first-line treatment
69.	Xie, Q., Guan, Y., Hofmann, S. G., Jiang, T., & Liu, X. (2023). The potential mediating role of anxiety sensitivity in the impact of mindfulness training on anxiety and depression severity and impairment: A randomized controlled trial. Scandinavian journal of psychology, 64(1), 21-29.	Outcome measure(s) outside protocol
70.	Xu, Q., Xu, B., & Lin, D. (2024). Assessing dance movement therapy in Chinese undergraduates with depression and anxiety: An initial randomized controlled trial. The Arts in Psychotherapy, 89, 102147.	Outcome measure(s) outside protocol
71.	Yaşar, A. B., Gündoğmuş, İ., Taşdelen, R., Taygar, A. S., Uludağ, E., Akça, E., ... & Türkçapar, M. H. (2024). A randomized controlled trial of the effect of cognitive behavioral therapy-based self-help psychotherapy books on anxiety and depressive symptoms: A bibliotherapy study. Dusunen Adam-The Journal of Psychiatry and Neurological Sciences.	Further-line treatment
72.	Zetsche, U., Neumann, P., Bürkner, P. C., Renneberg, B., Koster, E. H., & Hoorelbeke, K. (2024). Computerized cognitive control training to reduce rumination in major depression: A randomized controlled trial. Behaviour Research and Therapy, 177, 104521.	<80% non-chronic depression
73.	Zhang, Y., & Jiang, X. (2023). The effect of Baduanjin exercise on the physical and mental health of college students: a randomized controlled trial. Medicine, 102(34), e34897.	Outcome measure(s) outside protocol
74.	Zhang, Z. J., Zhang, S. Y., Yang, X. J., Qin, Z. S., Xu, F. Q., Jin, G. X., ... & Rong, P. J. (2023). Transcutaneous electrical cranial-auricular acupoint stimulation versus escitalopram for mild-to-moderate depression: An assessor-blinded, randomized, non-inferiority trial. Psychiatry and Clinical Neurosciences, 77(3), 168-177.	Data cannot be extracted (ITT at baseline and completer at endpoint)
75.	Zuo, Z., & Zhang, X. (2023). A randomized controlled trial of group CBT with positive psychotherapy intervention for university students with maladaptive perfectionism in China. Frontiers in Psychology, 14, 1161575.	Outcome measure(s) outside protocol
